# The sugar substitute Stevia shortens the lifespan of *Aedes aegypti* potentially by N-linked protein glycosylation

**DOI:** 10.1038/s41598-020-63050-3

**Published:** 2020-04-10

**Authors:** Arvind Sharma, Jeremiah Reyes, David Borgmeyer, Cuauhtemoc Ayala-Chavez, Katie Snow, Fiza Arshad, Andrew Nuss, Monika Gulia-Nuss

**Affiliations:** 1 Department of Biochemistry and Molecular Biology, University of Nevada, Reno, USA; 2 Department of Biology, University of California, San Diego, USA; 3 Department of Agriculture, Veterinary, and Rangeland Sciences, University of Nevada, Reno, USA

**Keywords:** RNA, Transcriptomics, Animal physiology

## Abstract

Adult male and female mosquitoes consume sugar as floral and extrafloral nectar. Earlier work demonstrated that mosquito populations and their vector potential are dependent upon the availability of sugar sources. Thus, a novel method of vector control may involve targeting sugar-feeding mosquitoes. Multiple human-safe sugar substitutes are already approved by the U.S. Food and Drug Administration and are readily available. However, plant-based sugar substitutes such as stevia (erythritol) have been shown to affect lifespan in other flies. Therefore, the current study was carried out to test the potential of commercially available sugar substitutes to adversely affect the survival, fecundity, and metabolism of adult *Aedes aegypti* mosquitoes. Of the four sugar substitutes tested, erythritol (Stevia), sucralose (Splenda), aspartame (Equal), and saccharin (Sweet’N Low), only erythritol negatively affected mosquito longevity and fecundity. The effect on fecundity was probably due in part to a corresponding decrease in glycogen and lipid levels over time in mosquitoes fed on erythritol. Comparative mosquito head transcriptomes indicated upregulation of a gene in the mannose biosynthesis pathway in females fed on erythritol, suggesting that N-linked glycosylation might be responsible for the negative impact of erythritol feeding in mosquitoes. Mosquitoes preferred sucrose when a choice was given but were not averse to erythritol. Our results suggest the possibility of using erythritol alone or in combination with sucrose as a component of attractive toxic sugar baits for a human-safe approach for mosquito control.

## Introduction

Mosquitoes are one of the most significant vectors of human pathogens and are a major burden to global health^1,2^. Widespread vector-borne disease incidence coupled with a limited number of effective vaccines or drugs to treat infections makes mosquito control an essential component of mosquito-borne disease management. Mosquito control primarily relies on insecticides, but the rapid and widespread resistance of mosquitoes to approved synthetic insecticides has created the need for novel, human-safe approaches for mosquito control^3–5^.

Most work to identify novel mosquito control targets has focused on female mosquitoes because males do not blood feed. Vertebrate blood provides a rich and abundant nutritional resource that female mosquitoes can allocate to egg production. However, all mosquitoes, including autogenous species^6^, regardless of sex, require a sugar meal shortly after emergence and throughout their lives^7^. The availability and type of sugar source in nature can alter the biting behavior of mosquitoes^8^ and have a significant impact on their vectorial capacity^9^. This reliance on sugar sources provides an opportunity to utilize this behavior for mosquito control^7^.

Attractive-toxic sugar baits (ATSB) are promoted as one of the new vector control paradigms because this method kills both female and male mosquitoes questing for essential sugar sources in the outdoor environment^10,11^. ATSB solutions have three main components: (1) fruit or flower scent as an attractant, (2) sugar solution as a feeding stimulant, and (3) oral toxin to kill the mosquitoes^11^. The ATSB’s may further be simplified by using non-nutritive sugar substitutes instead of sugar solution and oral toxin. Erythritol sweeteners are human-safe and are widely used in food products as natural sweeteners. The sweetener Truvia, and its main component erythritol, reduced *Drosophila melanogaster* survival in a dose-dependent manner^12–14^. A recent study in *Ae. aegypti* also demonstrated the negative impact of erythritol on both larvae and adults^15^. Considering the importance of sugar feeding in mosquitoes and its potential to be used in a human- and environmentally-safe approach for their control, the current study was designed to analyze the effects of four major, commercially available, sugar substitutes on mosquito physiology.

## Results

### Mosquitoes fed on erythritol had a shorter lifespan

**Figure 1:**
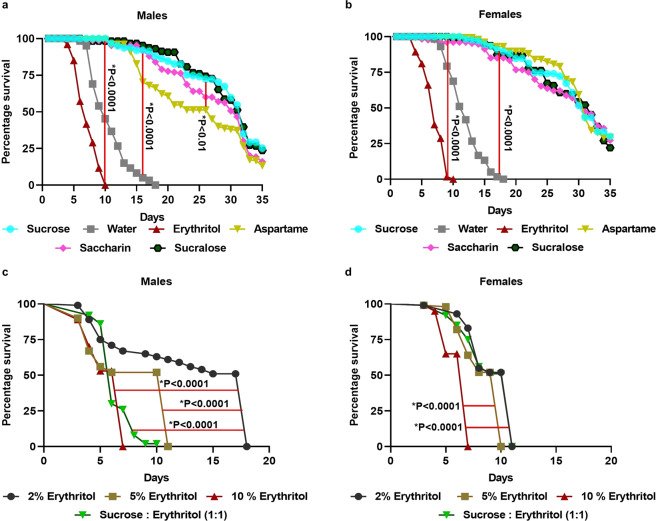
Effect of sugar substitute feeding on *Aedes aegypti* lifespan. Male (**a**) and female (**b**) adults were fed either 10% sucrose (control), water only (negative control), or 10% of a sugar substitute (erythritol, aspartame, saccharin, or sucralose) via cotton-soaked pads and mortality was recorded daily. Different concentrations of erythritol (2, 5, and 10%) and a mix of 1:1 (5% each) sucrose and erythritol were used to test the effect on mortality in males (**c**) and females (**d**). N = 50. Kaplan-Meier survival analysis was carried out for all treatments and Log-rank (Mantel-Cox) test was performed for the pairwise comparisons among groups. Vertical (a and b) and horizontal (c and d) red bars between two groups show statistical significance. (**a**) sucrose vs. water: χ^2^ = 185.3 p < 0.0001; sucrose vs. erythritol: χ^2^ = 189.6 p < 0.0001, sucrose vs. aspartame: χ^2^ = 5.6 p < 0.01; (**b)** sucrose vs. water: χ^2^ = 187.7 p < 0.0001; sucrose vs. erythritol: χ^2^ = 181 p < 0.0001; (**c**) 2% erythritol vs. 5% erythritol: χ^2^ = 51.5 p < 0.0001, 2% erythritol vs. 10% erythritol: χ^2^ = 67.1 p < 0.0001, and (**d**) 2% erythritol vs. 5% erythritol: χ^2^ = 32.5 p < 0.0001, 2% erythritol vs. 10% erythritol: χ^2^ = 111.2 p < 0.0001.

Both males and females fed on erythritol had a significantly shorter lifespan compared to sucrose-fed (males, χ^2^ = 189.9, p < 0.0001; females, χ^2^ = 187.7, p < 0.0001) and water only individuals (males, χ^2^ = 8.38, p < 0.001; females, χ^2^ = 121.3, p < 0.0001) (Fig. 1a,b). Survival of erythritol-fed males and females declined sharply from day 4 post-eclosion, and by day 10 all erythritol-fed mosquitoes (both sexes) were dead. In contrast, no water only control males died until day 7, but by day 18 all mosquitoes were dead in this treatment. For all other sugar substitutes and sucrose, mortality was first observed on day 12 in males and day 15 in females. Mortality increased gradually, and approximately 15% of males and 20% of females survived over 34 days post-eclosion when fed on sucrose and sugar substitutes other than erythritol (Fig. 1a,b). There was no significant difference in mortality between the sucrose, saccharin, and sucralose treatments in either males or females. However, a significant increase in mortality was observed in males, but not females, fed on aspartame (χ^2^ = 5.6, p < 0.01).

To test the dose-dependent effect of erythritol on mosquito mortality, we fed both males and females on different concentrations of erythritol. Mortality increased with an increase in erythritol concentration. Both males and females survived significantly longer when fed on 2% erythritol compared to 5% (males, χ^2^ = 51.5, p < 0.0001; females, χ^2^ = 32.5, p < 0.0001) and 10% (males, χ^2^ = 67.1, p < 0.0001; females, χ^2^ = 112.2, p < 0.0001) erythritol. Male mortality was first observed on day 4, and on days 7, 11, and 18, all males were dead in 10%, 5%, and 2% erythritol, respectively (Fig. 1c). Mortality in females was first observed on day 5 in 10% erythritol and on day 6 in both 5% and 2% erythritol. All females were dead on day 7, 10, and 11 in 10%, 5%, and 2% erythritol, respectively (Fig. 1d). All sucrose-fed mosquitoes (male and female) survived for at least 15 days, but at day 16, two males and one female died (data not shown). Because all the mosquitoes were dead in erythritol and 1:1 sucrose- erythritol samples, we did not follow with controls in this experiment after 18 days.

When sucrose was mixed with erythritol (1:1 concentration, 5% each), males feeding on the sucrose-erythritol mixture had a similar mortality rate to that fed on 5% erythritol-only. However, males fed on the mixture of erythritol-sucrose had more gradual mortality, and all males died at day 10, whereas in the erythritol-only (5%) treatment, 50% of the males survived until day 10. Survival sharply declined afterward and at day 11 all males were dead (Fig. 1c). By day 11, all males fed on 1:1 erythritol and sucrose were dead. In females, the sucrose–erythritol mixture also did not rescue lifespan compared to 5% erythritol-only treatments (Fig. 1d).

### Sucrose was preferred over sugar substitutes

**Figure 2:**
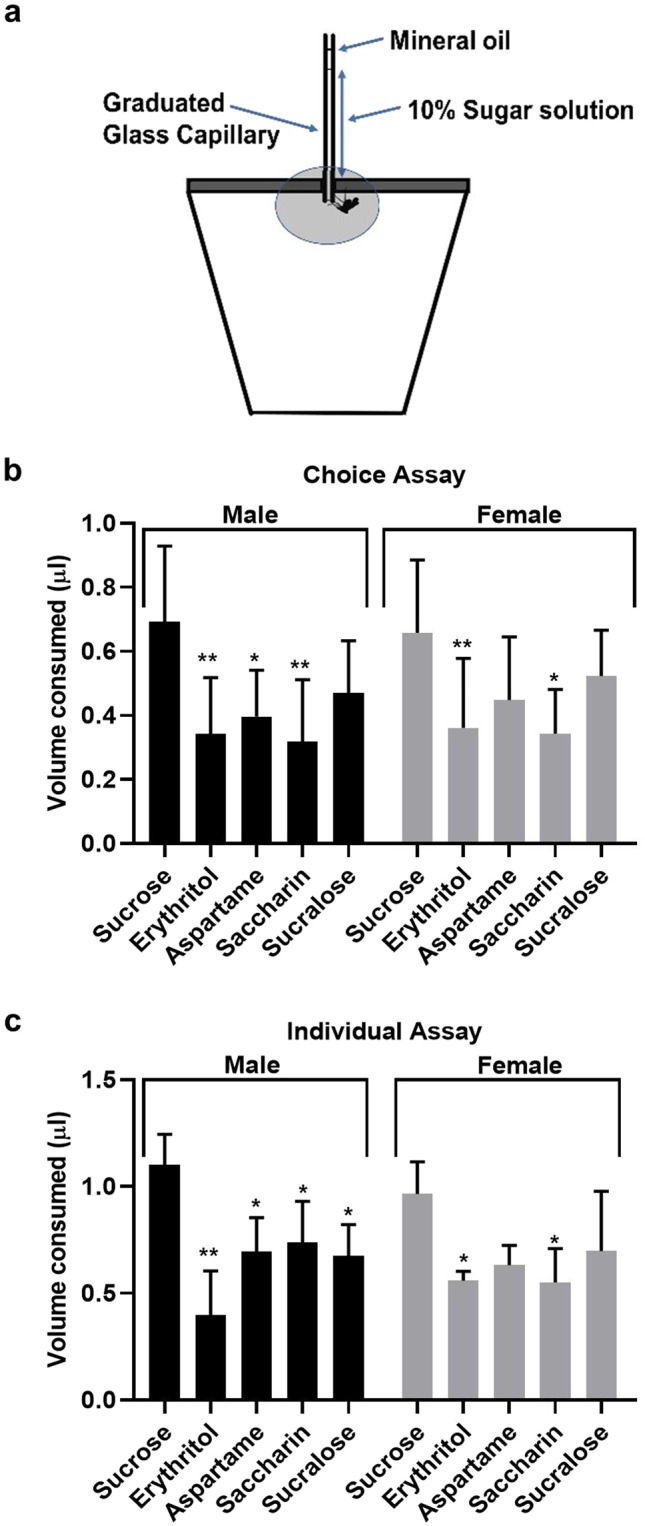
Feeding preference of *Aedes aegypti* for sugar and sugar substitutes. (**a**) A CAFE assay was used to determine the feeding preference of mosquitoes towards different sugar substitutes wherein an individual mosquito was offered liquids via a capillary tube. In the choice assay (**b**), adult males and females were starved for 24 h and then given a choice of all sugar and sugar substitutes for 24 h. In the individual feeding assay (**c**), 24 h starved mosquitoes were provided with only a single sugar or sugar substitute for 24 h. N = 10. One-way ANOVA [A: F (9, 106) = 5.905; B: F (9, 40) = 7.266] and Tukey’s multiple comparison were used to determine significance (*=p < 0.01; **=p < 0.001).

To test the preference of *Ae. aegypti* for sugar substitutes and sucrose, we performed two different experiments: 1) a choice experiment where adults were given access to all sugar and sugar substitutes at the same time, and 2) an individual feeding experiment where mosquitoes were only allowed to feed on a single sugar/substitute (Fig. 2a). Both males and females preferred sucrose and sucralose over other sugar substitutes when given access to all at the same time (Fig. 2b). Females also fed equally well on aspartame whereas both sexes least preferred erythritol and saccharin (Fig. 2b). When sugar and sugar substitutes were given individually, the trend was similar to the choice assay. Males consumed a significantly higher volume of sucrose compared to the sugar substitutes (Fig. 2c). Females consumed equal volumes of sucrose, aspartame, and sucralose, whereas a significantly lower volume of erythritol and saccharin was consumed (Fig. 2c).

### Mosquitoes fed on water and erythritol had comparatively lower levels of stored lipids and glycogen

**Figure 3:**
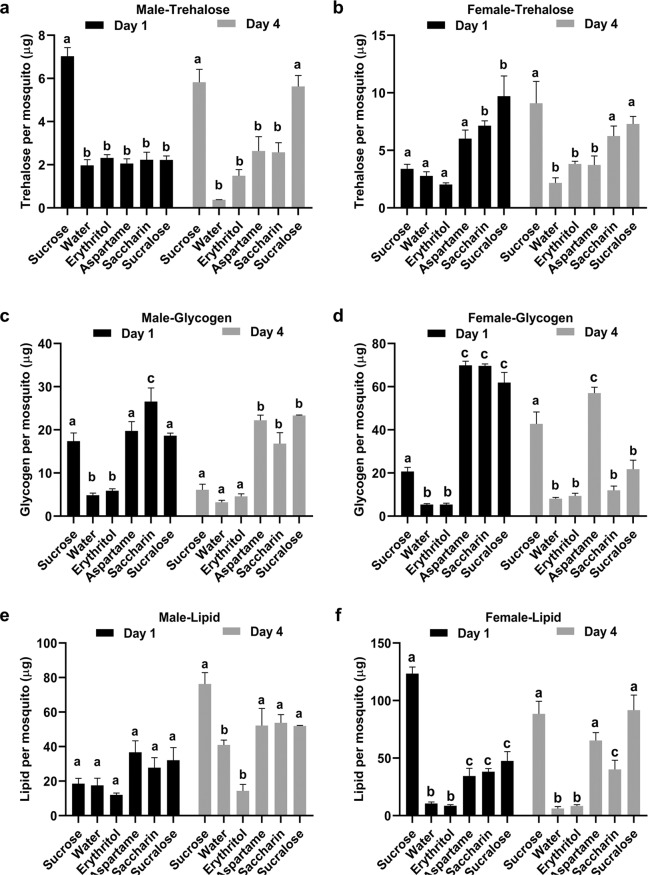
Effect of sugar substitute feeding on nutrient storage. Individual mosquitoes fed on sugar, water only, or sugar substitutes for 1 or 4 days were assayed for trehalose [males (**a**) and females (**b**)], glycogen [males (**c**) and females (**d**)], and lipid [males (**e**) and females (**f**)] using anthrone and vanillin assays (average ± SE). The experiment was repeated with two cohorts and three replicates within each cohort per time point (N = 6). Data were analyzed with a One-way ANOVA using Tukey’s multiple comparison tests between different groups. Significance was calculated as 95% confidence interval on the difference between means (p < 0.05). Means with different letters are significantly different.

To test the effect of different sugar substitutes on nutrient storage, we examined the levels of circulating carbohydrates (trehalose), glycogen, and lipid in mosquitoes fed on sugar or sugar substitutes *ad libitum* for 1 or 4 days (Fig. 3). In males, trehalose levels were significantly higher in day 1 sucrose-fed samples compared to water only and all other sugar substitutes. At day 4, trehalose levels in sucralose-fed samples were equal to the sucrose-fed males; however, all other were significantly lower (Fig. 3a). In females, trehalose levels at day 1 were significantly higher in the saccharin- and sucralose-fed individuals compared to the other treatments. At day 4, trehalose levels in sucrose-, saccharin-, and sucralose- fed individuals were similar, but significantly lower levels were recorded in water-, erythritol-, and aspartame-fed individuals (Fig. 3b).

Glycogen levels in males at day 1 were similar in sucrose-, aspartame-, and sucralose-fed mosquitoes, whereas water and erythritol-fed individuals had significantly lower glycogen levels. Saccharin-fed individuals had significantly higher levels of glycogen than all other treatments. At day 4 in males, glycogen level remained significantly high in aspartame-, saccharin-, and sucralose-fed compared to other treatments, including sucrose-fed (Fig. 3c). In females, at day 1, glycogen levels were approximately 3-fold higher in aspartame-, saccharin-, and sucralose-fed compared to the sucrose-fed females, whereas water and erythritol- fed females had significantly lower levels of glycogen than all other treatments (Fig. 3d). At day 4, glycogen levels in saccharin- and sucralose-fed fell to levels similar in water and erythritol-fed individuals whereas in sucrose- and aspartame-fed females levels remained significantly high (Fig. 3d).

Lipid levels in day 1 males were not significantly different between treatments. At day 4, lipid levels increased in all treatments except water and erythritol-fed (Fig. 3e). In females, at day 1, lipids levels were significantly higher in sucrose-fed mosquitoes compared to all other treatments. The least amount of lipids were detected in water and erythritol-fed females. All other sugar substitutes had significantly higher level of lipids compared to water and erythritol-fed but lower than sucrose-fed females. At day 4, lipid levels in aspartame- and sucralose-fed were similar to the sucrose-fed females. Saccharin-fed individuals had significantly lower amounts of lipid at day 4 compared to sucrose, aspartame, and sucralose-fed but significantly higher than water and erythritol-fed females (Fig. 3f).

### Erythritol feeding prior to a blood meal significantly reduced fecundity and hatchability

**Figure 4:**
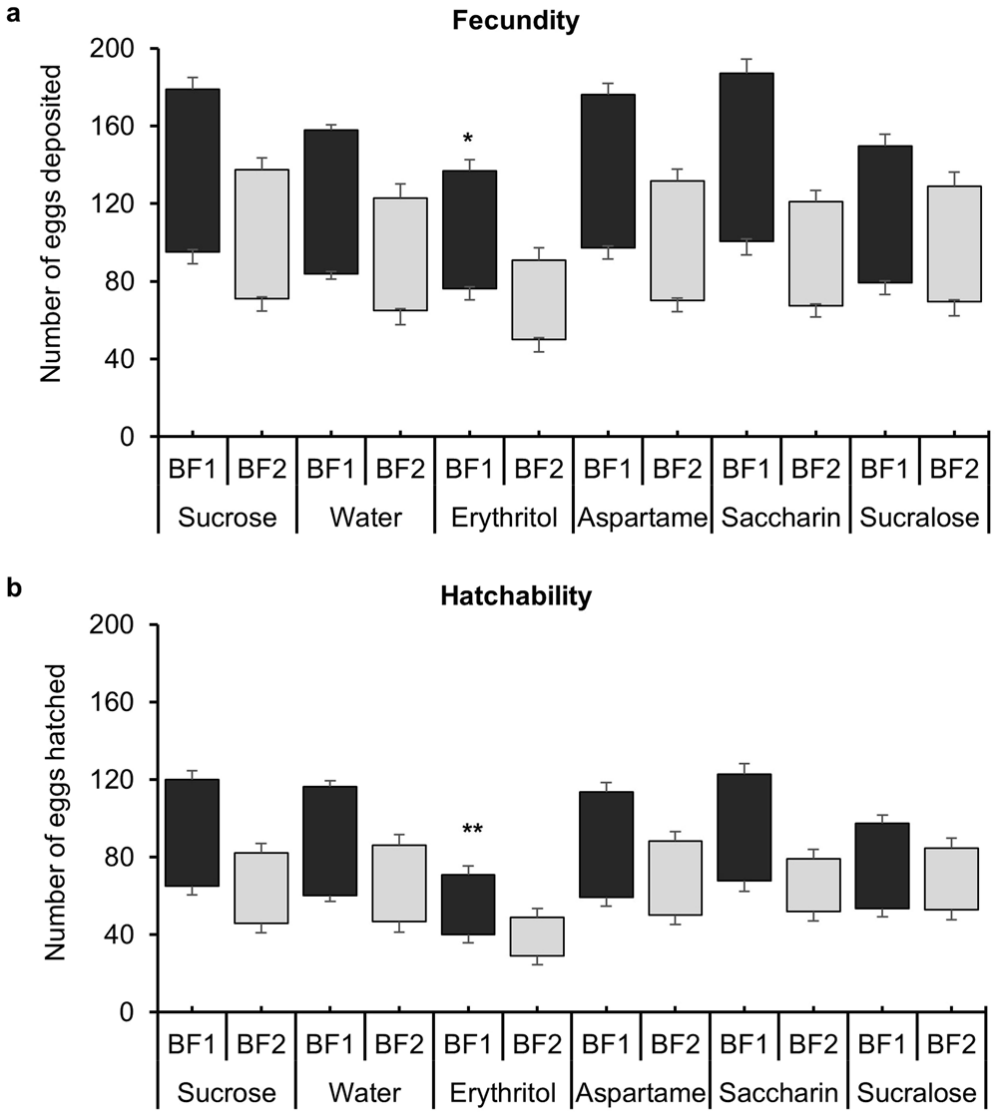
Sugar substitute impact on fecundity and egg hatchability. (**a**) Number of eggs laid by female *Ae. aegypti* that were fed sugar, water only, or sugar substitutes for 4 days post emergence, then blood fed (first gonotrophic cycle = BF1). Once eggs were deposited, a second blood meal was provided to test the effect on the second gonotrophic cycle (BF2). (**b**) Number of larvae hatched from the eggs from the first and second gonotrophic cycles (BF1 and BF2). Black boxes represent first blood meal and gray boxes depict second blood meal data. * = p < 0.05, ** = p < 0.01.

Fecundity in females maintained on sugar substitutes was recorded for two consecutive gonotrophic cycles. During the first gonotrophic cycle, the number of eggs deposited was significantly lower in individuals fed on erythritol before a blood meal in comparison to sucrose, water, and other sugar substitutes. However, there was no significant difference in the number of eggs laid in the second gonotrophic cycle (Fig. 4a). A similar pattern was observed for hatchability where egg hatching was significantly lower in the first gonotrophic cycle of erythritol-fed females but not in the second gonotrophic cycle (Fig. 4b).

### **Mannose biosynthesis pathway gene was upregulated in heads of females fed on erythritol**

To investigate a possible mechanism of action of mortality in erythritol-fed mosquitoes, RNA was isolated from a pool of 10 female heads per sample. Three libraries for each treatment were subjected to Tag-seq. An average of 7,569,145 reads per sample were obtained, and 82% of reads aligned with the *Ae. aegypti* L5 genome^16^. 61% aligned uniquely with annotated genes. After filtering out low expressed genes, 10,900 genes were recovered. Differential expression analysis detected only one statistically significant, differentially expressed gene: mannose-1-phosphate guanyltransferase (AAEL011912) (adjusted p-value <0.05) between erythritol and water controls at day 1 (Supplemental Table). Three more genes in the mannose biosynthesis pathway [dolichyl glycosyltransferase (AAEL002996), a putative dolichyl-phosphate-mannose-protein mannosyltransferase, PMT2 (AAEL010227), and dolichyl-phosphate b-D-mannosyltransferase, DPM1 (AAEL003084)] were identified below the cutoff but were not statistically significant (Supplementary data).

**Figure 5:**
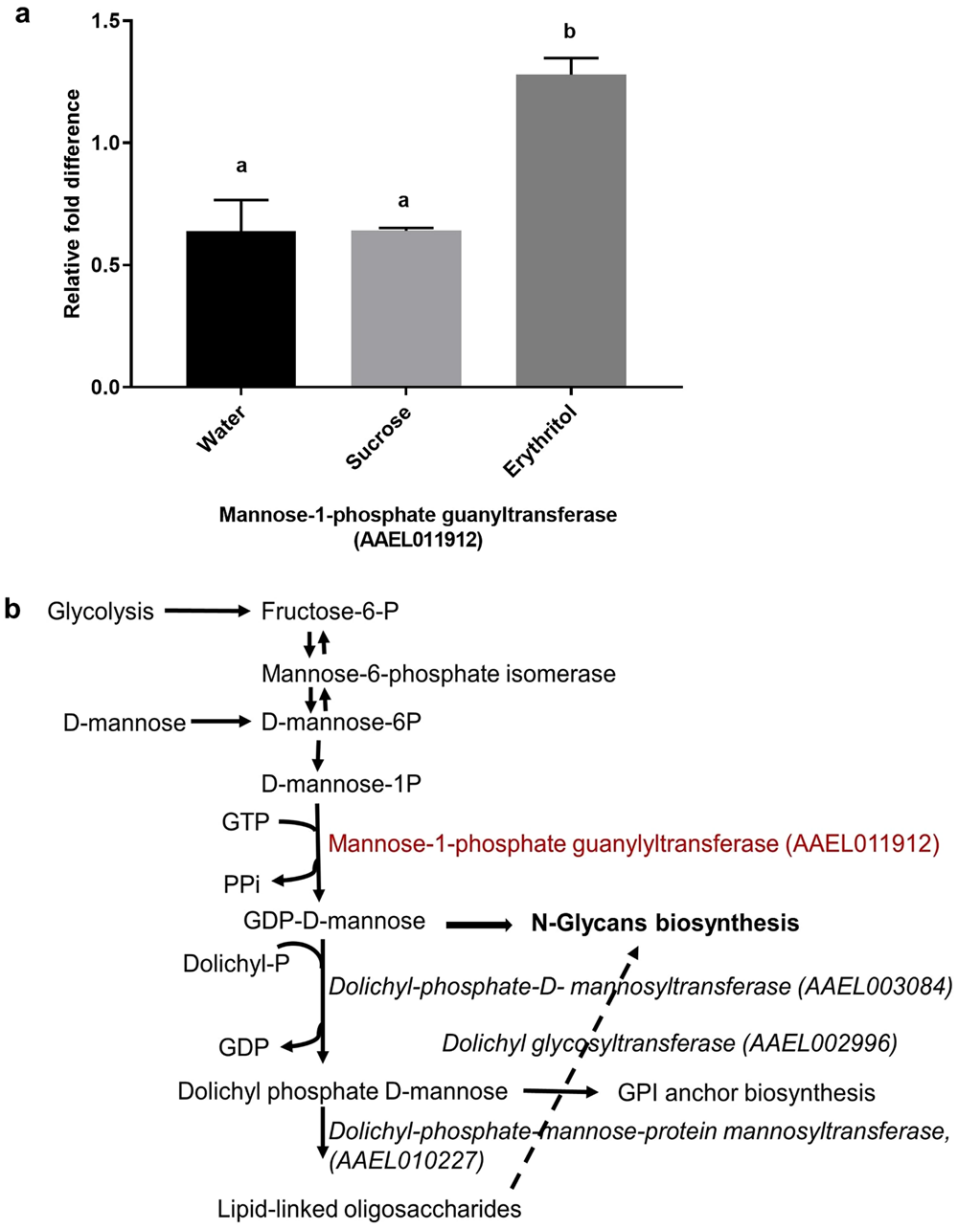
Erythritol feeding impacts the N-glycan pathway. (**a**) Real time PCR validation of mannose-1-phosphate guanylyltransferase (AAEL011912) expression differences in heads of *Ae. aegypti* females fed water, sucrose, or erythritol (N = 2). Significance determined by 1 way ANOVA, [F (2, 3) = 19.3] and Tukey’s multiple comparison between groups (p < 0.05). (**b**) Schematic of the N-linked glycosylation pathway^23,24^. The only significantly differentially expressed gene, mannose-1-phosphate guanylyl-transferase, is highlighted in red font. The additional three genes in this pathway are shown in italics.

Validation by qRT-PCR also supported the 3′ Tag Seq results for mannose-1-phosphate guanylyltransferase (Fig. 5a). This gene is essential for the protein glycosylation pathway and is involved in N-glycans biosynthesis, mannose metabolism, and GPI anchor biosynthesis (Fig. 5b). No other treatments resulted in any significant difference in gene expression.

## Discussion

Our data strongly suggest that the erythritol-based sugar substitute with erythritol as an active compound is toxic when ingested by both male and female *Ae. aegypti* (Fig. 1). Other sugar substitutes tested did not affect mosquito lifespan, reproduction, or physiology. Previous studies on *D. melanogaster*^12–14^, *D. suzukii*^17^, and *Bactrocera dorsalis*^18^ also showed a similar insecticidal effect of erythritol in flies, although erythritol ingestion had no toxic effect on the housefly, *Musca domestica*^19^. Adult fruit flies fed on aspartame and saccharin also had significantly higher mortality compared to those fed on sucrose^18^. However, in another report, except for Truvia (another brand name of erythritol-based sweetener), there was no effect of sugar-substitutes [Equal (aspartame), Splenda (sucralose), Sweet’N Low (saccharin), and PureVia (erythritol)] on *D. melanogaster* lifespan^13^. Our data agree with Baudier *et al*.^13^, in that sugar substitutes other than erythritol did not affect lifespan, fecundity, and egg hatching in mosquitoes. In *D. melanogaster*, longevity due to erythritol ingestion was dose-dependent. Similarly, both male and female mosquitoes survived longer on 2% erythritol compared to 5% and 10% erythritol in our study (Fig. 1c,d). When mosquitoes were fed on 5% each of sucrose and erythritol combined, sucrose had no protective effect when ingested with erythritol (Fig. 1c,d). A recent study in *Ae. aegypti* showed that in adult females, the combination of erythritol and sucrose was more lethal to females than the erythritol alone^15^. Similar results were also observed with *D. melanogaster* fed on a combination of erythritol and sucrose^13^.

Our CAFE assay with choice and individual feeding experiments suggested that both males and females ingested all sugar substitutes. Sucrose and sucralose were more readily consumed, but mosquitoes consumed sugar substitutes even in the presence of sucrose (Fig. 2b,c). *D. suzukii*, when given a choice between sucrose and erythritol, preferred sucrose^17^ whereas *D. melanogaster* preferred erythritol suggesting differences in preference between different fly species^13^.

Water and erythritol-fed mosquitoes consistently had the lowest levels of trehalose, glycogen, and lipids compared to the other treatments and were in many instances significantly lower. Similarly, in *D. suzukii*, trehalose and glycogen levels were significantly higher in sucrose-fed female flies when compared with water control and erythritol-fed flies^17^. No other study examined lipid levels. Α-glucosidase from the gut of *Ae. aegypti* catalyzes the hydrolysis of terminal, non-reducing α1–4-linked glucose residues from aryl-glucosides (as p-nitrophenyl-α-D-glucoside)^20^. The physiological role of the midgut α-glucosidase is thought to be the release of glucose from oligosaccharides^21^. Erythritol is a competitive inhibitor of α-glucosidase^22^. Our data with 1:1 sucrose and erythritol feeding support this previous finding that the ingestion of erythritol inhibits sucrose metabolism and this inability to metabolize sugars might be responsible for death due to starvation.

Lower metabolite levels in females fed on erythritol for 4 days before a blood meal resulted in significantly fewer eggs deposited during the first gonotrophic cycle. However, there was no significant effect of previous erythritol feeding on egg numbers during the second gonotrophic cycle compared to the other treatments (Fig. 4a). We maintained the mosquitoes on the water between the first and second feedings and it is, therefore, possible that the mosquitoes cleared the erythritol from the body by excretion, metabolism, or sequestration prior to the second blood- feeding, permitting recovery of metabolic function. Interestingly, water-fed females deposited an equal number of eggs as females fed sucrose and other sugar substitutes suggesting that the blood meal alone provided sufficient nutrients to mature a batch of eggs.

Previous studies have shown a negative correlation between feeding on erythritol and motor coordination in flies^13–15,17,18^. Therefore, we hypothesized that gene expression in the brain (head) of water, sucrose, and sugar substitutes-fed mosquitoes would differ and that these differences might help pinpoint the mechanism of erythritol toxicity in insects. Interestingly, there were no differences in head transcriptome between water, sucrose, and sugar substitutes other than erythritol, further supporting our physiology data. One gene, mannose-1-phosphate guanyltransferase, was significantly upregulated in erythritol-fed females compared to water and sucrose (Fig. 5). Three additional genes in the mannose biosynthesis pathway (dolichyl glycosyltransferase, a putative dolichyl-phosphate-mannose-protein mannosyltransferase, and dolichyl-phosphate β-D-mannosyltransferase) had increased expression in erythritol-fed mosquitoes, but these were outside the significance cutoff value (p < 0.05) (Supplemental Data S1). The very few differentially expressed genes detected in our data are in part due to the use of more robust 3′ Tag Seq instead of standard RNASeq. While both RNASeq and 3′ Tag Seq are able to quantify gene expression, the advantage of RNAseq is in high sequence coverage, whereas the strength of 3′ Tag Seq lies in highly sensitive gene expression quantification. It is also possible that gene expression differs more strongly in other tissues, such as the fat body, instead of the head. In future experiments, we plan to examine fat body transcriptomes to better understand the differences in metabolic genes and pathways induced by erythritol.

Mannose-1-phosphate guanyltransferase (and the other three genes mentioned above) function in the glycosylation pathway (Fig. 5b)^23,24^. In higher organisms, glycosylation is one of the most common and evolutionarily conserved posttranslational modifications of proteins and regulates numerous developmental and physiological processes. Glycosylation pathways influence cellular decisions, for instance for proliferation and apoptosis. Glycosylation in the nervous system is thought to play a particularly important role in both vertebrates and invertebrates given the extraordinary complexity of synaptic connections^25^. Increasing evidence indicates that glycosyltransferases play a critical role in the metabolism of many drugs and active components of various natural products^26^, and might also be involved in the metabolism and detoxification of erythritol in mosquitoes.

Our findings demonstrate that erythritol is toxic to *Ae. aegypti* and suggest a role of protein glycosylation in the toxicity of erythritol. The decreased lifespan in mosquitoes to less than 10 days after adult emergence could have a significant impact on pathogen transmission because most pathogens require an extrinsic incubation time of 12–15 days after initial ingestion of the pathogens within a blood meal until the pathogen can be transmitted by the mosquito vector^27^. These data set the stage for investigating erythritol as an effective and human-safe approach for mosquito control. Further understanding of the molecular mechanisms involved would help identify additional novel targets for vector control using ATSB.

## Materials and Methods

### Mosquito Rearing

*Ae. aegypti* (UGAL strain) colonies were maintained at 27 °C and 78% relative humidity (RH) with a photoperiod of 16 h light and 8 h dark^28^ in a dedicated insectary. Eggs were hatched overnight in small plastic cups with deionized water. First instar larvae were counted (150 per pan) and reared in 500 ml of deionized water on a powdered fish food diet (Tetramin^®^, Melle, Germany) as described in detail by Pooraiiouby *et al*.^29^. Pupae were collected from rearing pans and were transferred to adult emergence cages. Adults were provided with water and 10% sucrose solution *ad libitum* for regular colony maintenance.

### Sugar substitutes

Four commercially available sugar substitutes [Erythritol (Stevia), aspartame (Equal), saccharin (Sweet’N Low), and sucralose (Splenda)] were purchased from a local grocery store.

### Survival assays

An equal number of male and female pupae were kept in separate, clear plastic emergence cages for adult emergence. Adults were provided with either 10% sucrose (positive control), 10% sugar substitute (unless otherwise stated), or water only (negative control). Mortality was recorded every day until all mosquitoes died. Moribund and dead mosquitoes were observed under a microscope to confirm death (i.e., motionless even when probed). The experiment was replicated thrice with a different biological cohort of mosquitoes (90 males and 90 females per biological replicate, 3 replicates: total 270 mosquitoes of each sex; N = 3). Dead mosquitoes were recorded daily and percent survival was calculated individually for each biological cohort and an average of all three cohorts was plotted. Kaplan-Meier survival analysis was used to determine significance.

To test for a dose-dependent effect on mortality, mosquitoes were fed on different concentrations of erythritol (2, 5, and 10%). A 1:1 mixture of 5% sucrose and 5% erythritol was also used to test the effect of erythritol in the presence of sucrose. These subsequent experiments were performed on a single biological cohort with 50 individuals of each sex (N = 1).

Survivorship (S) was calculated each day by using the formula:

$${\rm{S}}({\rm{Day}}\,{\rm{X}})=\{{\rm{Number}}\,{\rm{of}}\,{\rm{alive}}\,{\rm{mosquito}}\,{\rm{at}}\,{\rm{day}}\,{\rm{X}}/{\rm{Total}}\,{\rm{number}}\,{\rm{of}}\,{\rm{mosquito}}\,{\rm{alive}}\,{\rm{at}}\,{\rm{day}}\,0\}\times 100$$

### Capillary feeder assay (CAFE assay)

To determine *Ae. aegypti* preference for sugar and sugar substitutes, a CAFE assay was carried out according to the established protocols for *D. melanogaster*^13^ (Fig. 2a) in two different setups:

#### Choice assay

Newly emerged adult *Ae. aegypti* were starved for 24 h before they were provided with a choice of all sugar and sugar substitutes (10% solutions each) at the same time in 5 µl graduated capillaries (VWR international, PA, USA). A single adult was tested at a time in a 6 oz. cup, and the capillaries were attached to the top of the cup. The position of the capillaries was randomized in the cups to avoid any positional bias. A decrease in the level of sugar solutions was recorded after 24 h. Control capillaries without any mosquitoes were kept along with experimental cups to measure evaporation. The volume of each sugar/sugar substitute ingested by the mosquitoes was calculated by subtracting the evaporation volume from the experimental sample volume. The experiment was repeated with 5 technical replicates (males and females) and two different cohorts of mosquitoes (N = 10 for each sex).

#### Individual assay

Newly emerged adults were starved for 24 h and then were given access to a single sugar solution for 24 h in a similar setting as described above. A single adult mosquito was introduced into each cup containing a single capillary tube filled either with 10% sucrose or a 10% sugar substitute. The volume of sugar/sugar substitute consumed was calculated as above. The experiment was repeated with 5 technical replicates (males and females) and two different cohorts of mosquitoes (N = 10 for each sex). The distention of the crop was also recorded to confirm sugar ingestion.

### Metabolic bioassays

Both male and female mosquitoes were fed on water only, sucrose, or individual sugar substitutes *ad libitum*. Trehalose, glycogen, and lipid levels were quantified from these mosquitoes at day 1 and day 4 post-eclosion. Two mosquitoes per sample were collected in triplicate at each time point per treatment. Samples were processed for micro-separation of metabolites from both males and females according to previously established protocols^29^. Levels of trehalose and glycogen were estimated using the anthrone assay and lipids were measured with a vanillin assay^28^. In females, the experiment was replicated twice with different biological cohorts (N = 6), whereas in males the assay was performed with a single biological cohort and three technical replicates (N = 3).

### Fecundity & hatchability

Four-day old females reared on different sugar substitutes were fed on vertebrate blood via an artificial membrane feeder. Ten females per sugar/sugar substitute treatment were kept individually in 250 ml plastic cups lined with a moist paper towel for egg laying. The number of eggs deposited by each female was counted 5 days post blood meal (PBM). After drying for three days, eggs were kept for hatching in 100 ml H_2_O in plastic specimen cups and hatched under a vacuum. First instar larvae that hatched from the eggs were counted. Females were provided with only water between the blood meals. At 6 days PBM, females were blood-fed again for a second gonotrophic cycle. The experiment was replicated twice with different biological cohorts of mosquitoes (N = 40).

### 3′ Tag RNA Sequencing

Mosquitoes were fed *ad libitum* on either sucrose, water, or sugar substitutes for 1 or 4 days. Heads were collected from a pool of ten females from day 1 and day 4 samples from three different biological cohorts (N = 3). Total RNA was isolated with Trizol and the Zymo RNA extraction kit (Zymo Research, Irvine, CA, USA). Total RNA samples were run on a 1.2% TBE agarose gel and an Agilent chip (Agilent, Santa Clara, CA, USA) for quality control. All RNA samples were run on a Qubit fluorometer (Invitrogen, Carlsbad, CA, USA) and had RIN values of 7.8–8.2. Total RNA was submitted to the UC Davis Genomics Core for Illumina sequencing using 3′ Tag Seq library preparation and ran on an Illumina HiSeq. 2500.

Standard RNA sequencing provides full-length, whole-mRNA data which is generally not required for estimates of relative gene expression. 3′ Tag Seq is an alternative method that focuses on the 3′ end of mRNAs, thereby reducing the sequencing depth per sample. 3′ Tag Seq was preferred in this situation because it has been shown to measure RNA distribution more accurately than standard RNAseq, especially for transcripts of moderate to low abundance^30^.

### 3′ Tag RNA Sequencing data analysis and data validation

Low expressed genes (less than two counts per million in all samples) were filtered out prior to analysis. Differential expression was conducted using the limma-voom^31^ Bioconductor pipeline and functional annotations were downloaded from Ensembl.

The one gene that was significantly differentially expressed in day 1 head samples in erythritol-fed females was validated with qRT-PCR. For this, two biological sets of mosquitoes were fed on water, 10% sucrose (positive control), and 10% erythritol for one day. Ten females per cohort were collected from each set for total RNA isolation. RNA was isolated with Trizol reagent (Invitrogen). 5 µg of total RNA was used for DNase treatment (Sigma, St. Louis, MO, USA) according to the manufacturers’ protocol. DNase-treated RNA samples were re-purified with Trizol. DNase-treated RNA (1 μg) was used for cDNA synthesis with iScript Reverse Transcription Supermix (BioRad, Hercules, CA, USA). cDNA was diluted 10x in water before using as a template in qRT-PCR experiments. qRT-PCR was performed on a CFX Touch Real-Time PCR Detection System (BioRad, Hercules, CA, USA), using the SYBR green master mix (BioRad). Gene-specific primers for mannose-1-phosphate guanyltransferase were designed (AaM1-PGT Fwd: 5′TATGGGAACGGAAGCAACCC3′ and AaM1-PGTRev: 5′ACGTACACGCCACAGTTGAT3′). A ribosomal protein S7 gene was used as a housekeeping control^29^. Relative expression was calculated using the 2^−ΔΔCt^ method.

### Statistical analysis

Statistical analysis was carried out using GraphPad Prism 8 software (La Jolla, CA, USA). All data (trehalose, glycogen, lipids, CAFE assay, mannose biosynthesis pathway genes validation) were analyzed by one-way ANOVA and Tukey’s multiple comparison tests between different groups. The metabolic assays (Trehalose, Glycogen, and Lipids) data were compared separately for day 1 and day 4 [Control (sucrose-fed) vs. treatments (water and all four substitutes)]. For fecundity and hatchability data, Dunnet’s comparison was used with one-way ANOVA. For mortality data, the average survival was first calculated by summing all biological replicates data for each day. Then Kaplan-Meier survival analysis was performed on all the treatments and a Log-rank (Mantel-Cox) test was performed to do the pairwise comparisons among groups.

## Supplementary information

Supplementary information.

## Data Availability

All data are available in the text and supplementary materials. 3′ Tag sequencing data has been submitted to the National Center for Biotechnology Information and available as a BioProject accession number: PRJNA598930 for free download.
