# Author Correction: The sugar substitute Stevia shortens the lifespan of *Aedes aegypti* potentially by N-linked protein glycosylation

**DOI:** 10.1038/s41598-021-84560-8

**Published:** 2021-03-10

**Authors:** Arvind Sharma, Jeremiah Reyes, David Borgmeyer, Cuauhtemoc Ayala-Chavez, Katie Snow, Fiza Arshad, Andrew Nuss, Monika Gulia-Nuss

**Affiliations:** 1 Department of Biochemistry and Molecular Biology, University of Nevada, Reno, USA; 2 Department of Biology, University of California, San Diego, USA; 3 Department of Agriculture, Veterinary, and Rangeland Sciences, University of Nevada, Reno, USA

Correction to: *Scientific Reports* 10.1038/s41598-020-63050-3, published online 10 April 2020

The original version of this Article contained an error in the title of the paper, where the word “erythritol” was used instead of “Stevia”. This has now been corrected in the PDF and HTML versions of the Article, and in the accompanying Supplementary Information file.

Additionally, the original version of this Article contained an error in the Introduction section.

“The ATSB’s may further be simplified by using non-nutritive sugar substitutes instead of sugar solution and oral toxin. Erythritol, derived from the South American plant *Stevia rebaudiana Bertoni* and commonly known as stevia, is up to 300 times sweeter than sucrose^12^. Erythritol sweeteners are human-safe and are widely used in food products as natural sweeteners.”

now reads:

“The ATSB’s may further be simplified by using non-nutritive sugar substitutes instead of sugar solution and oral toxin. Erythritol sweeteners are human-safe and are widely used in food products as natural sweeteners.”

As a result of the changes above, one Reference was removed and is listed below:

Lemus-Mondaca, R., Vega-Gálvez, A., Zura-Bravo, L. & Kong, A. H. Stevia rebaudiana Bertoni, source of a high-potency natural sweetener: A comprehensive review on the biochemical, nutritional and functional aspects. Food Chemistry 132, 1121–1132 (2012).

Consequently, References 12–32 were incorrectly listed as References 13–33. This has now been corrected in the PDF and HTML versions of the Article.

