# Supplementary material for: The sugar substitute Stevia shortens the lifespan of Aedes aegypti potentially by N-linked protein glycosylation
Source: Sci Rep. 2020 Apr 10;10:6195. doi: 10.1038/s41598-020-63050-3 (PMC7148303; doi:10.1038/s41598-020-63050-3)
Supplement: Supplementary file 1 — Supplementary information. [file 41598_2020_63050_MOESM1_ESM.pdf]

**The sugar substitute Stevia shortens the lifespan of *Aedes aegypti* potentially by N-linked protein glycosylation**

Arvind Sharma[1], Jeremiah Reyes[1], David Borgmeyer[2], Cuauhtemoc Ayala-Chavez[1], Katie Snow[1], Fiza Arshad[1], Andrew Nuss[1, 3 *], Monika Gulia-Nuss[1*]

[1]Department of Biochemistry and Molecular Biology, University of Nevada, Reno, USA

[2]Department of Biology, University of California, San Diego, USA

[3]Department of Agriculture, Veterinary, and Rangeland Sciences, University of Nevada, Reno, USA

*Corresponding authors

Email: nuss@cabnr.unr.edu; mgulianuss@unr.edu

| ensembl_gene_ | external_g | logFC | AveExpr | P.Value | adj.P.Val | description |
|---|---|---|---|---|---|---|
| AAEL011912 | | 1.7719 | 4.4555 | 0.0000 | 0.0324 | mannose-1-phosphate guanyltransferase [Source:VB Community Annotation] |
| AAEL002996 | | 1.7914 | 4.8373 | 0.0000 | 0.0947 | dolichyl glycosyltransferase [Source:VB Community Annotation] |
| AAEL004879 | | 1.8312 | 6.4762 | 0.0000 | 0.1039 | |
| AAEL007809 | | 1.7939 | 5.7865 | 0.0001 | 0.2000 | potassium channel regulator [Source:VB Community Annotation] |
| AAEL021232 | NA | 1.4837 | 5.7125 | 0.0001 | 0.2000 | NA |
| AAEL000505 | | 2.4779 | 3.6011 | 0.0003 | 0.5925 | |
| AAEL026962 | NA | 1.8808 | 3.2726 | 0.0006 | 0.8994 | NA |
| AAEL022931 | NA | 1.3078 | 6.9726 | 0.0008 | 0.9636 | NA |
| AAEL013065 | | 1.2056 | 4.2723 | 0.0008 | 0.9636 | thiamin pyrophosphokinase [Source:VB Community Annotation] |
| AAEL004444 | | 1.5753 | 5.0878 | 0.0014 | 0.9996 | zinc finger protein [Source:VB Community Annotation] |
| AAEL003624 | | -1.5043 | 5.4025 | 0.0015 | 0.9996 | sodium/chloride dependent amino acid transporter [Source:VB Community Annotation] |
| AAEL006643 | | 1.9204 | 1.6132 | 0.0015 | 0.9996 | |
| AAEL003084 | | 1.4561 | 5.5846 | 0.0017 | 0.9996 | dolichyl-phosphate beta-D-mannosyltransferase, putative [Source:VB Community Annotation] |
| AAEL010227 | | 1.5422 | 6.3285 | 0.0020 | 0.9996 | dolichol-phosphate mannosyltransferase [Source:VB Community Annotation] |
| AAEL012973 | | 1.1026 | 4.2275 | 0.0025 | 0.9996 | uracil phosphoribosyltransferase [Source:VB Community Annotation] |
| AAEL002331 | | 1.2345 | 6.8832 | 0.0026 | 0.9996 | Mannose-6-phosphate isomerase [Source:UniProtKB/TrEMBL;Acc:Q17IJ3] |
| AAEL001705 | | 1.3412 | 5.9986 | 0.0029 | 0.9996 | odorant response protein ODR-4, putative [Source:VB Community Annotation] |
| AAEL007041 | | -0.8277 | 5.3507 | 0.0030 | 0.9996 | low-density lipoprotein receptor (ldl) [Source:VB Community Annotation] |
| AAEL020651 | NA | 1.6869 | 4.8062 | 0.0031 | 0.9996 | NA |
| AAEL021998 | NA | 2.9562 | -1.4008 | 0.0033 | 0.9996 | NA |
| AAEL000885 | | 3.4205 | -0.4401 | 0.0033 | 0.9996 | cytoplasmic dynein heavy chain [Source:VB Community Annotation] |
| AAEL024500 | NA | 1.3169 | 5.8929 | 0.0034 | 0.9996 | NA |
| AAEL011638 | | 1.1104 | 3.7478 | 0.0034 | 0.9996 | cyclic-nucleotide-gated cation channel [Source:VB Community Annotation] |
| AAEL004334 | | 1.2432 | 3.3345 | 0.0036 | 0.9996 | |
| AAEL008216 | | -0.9338 | 6.2544 | 0.0040 | 0.9996 | aconitase [Source:VB Community Annotation] |
| AAEL007646 | | -1.0733 | 4.2189 | 0.0040 | 0.9996 | |
| AAEL013828 | | 1.3944 | 3.2779 | 0.0043 | 0.9996 | translation initiation factor eif-2b [Source:VB Community Annotation] |
| AAEL022615 | NA | 1.3825 | 2.9367 | 0.0043 | 0.9996 | NA |
| AAEL014761 | | 1.6852 | 4.8002 | 0.0044 | 0.9996 | |
| AAEL003130 | | 1.2749 | 5.2459 | 0.0046 | 0.9996 | bcr-associated protein, bap [Source:VB Community Annotation] |
| AAEL019900 | NA | 1.6031 | 2.1565 | 0.0050 | 0.9996 | NA |
| AAEL027864 | NA | 2.6780 | -2.0379 | 0.0050 | 0.9996 | NA |
| AAEL003885 | | -3.2399 | -1.1481 | 0.0053 | 0.9996 | |
| AAEL027249 | NA | 1.0591 | 5.3661 | 0.0056 | 0.9996 | NA |
| AAEL006269 | | 1.9429 | 2.5591 | 0.0059 | 0.9996 | |
| AAEL010384 | | 2.1976 | 3.0392 | 0.0063 | 0.9996 | aldehyde oxidase [Source:VB Community Annotation] |
| AAEL004487 | | -2.0549 | 0.4714 | 0.0064 | 0.9996 | valacyclovir hydrolase [Source:VB Community Annotation] |
| AAEL013098 | | 1.2651 | 6.0494 | 0.0067 | 0.9996 | Coatomer subunit alpha [Source:UniProtKB/TrEMBL;Acc:Q16K68] |
| AAEL001314 | | 1.7268 | 5.9820 | 0.0067 | 0.9996 | |

| | | | | | | |
|---|---|---|---|---|---|---|
| AAEL001601 | | 0.9731 | 4.6695 | 0.0068 | 0.9996 | |
| AAEL012777 | | 2.1551 | 4.8537 | 0.0069 | 0.9996 | serine protease snake, putative [Source:VB Community Annotation] |
| AAEL023595 | NA | 2.2794 | 1.2278 | 0.0070 | 0.9996 | NA |
| AAEL025769 | NA | 1.6571 | 5.2159 | 0.0077 | 0.9996 | NA |
| AAEL027123 | NA | 1.3897 | 2.0844 | 0.0081 | 0.9996 | NA |
| AAEL009510 | | 1.6570 | 5.0512 | 0.0083 | 0.9996 | glucosamine-fructose-6-phosphate aminotransferase [Source:VB Community Annotation] |
| AAEL009584 | | 2.4924 | 3.0285 | 0.0084 | 0.9996 | elg, drosophila [Source:VB Community Annotation] |
| AAEL000407 | | 1.1486 | 5.2284 | 0.0085 | 0.9996 | Protein kish [Source:UniProtKB/TrEMBL;Acc:Q17P87] |
| AAEL001930 | | 0.6951 | 6.2361 | 0.0086 | 0.9996 | pra1 protein [Source:VB Community Annotation] |
| AAEL025801 | NA | 2.4969 | 1.9855 | 0.0086 | 0.9996 | NA |
| AAEL012748 | | 2.6309 | -0.4157 | 0.0086 | 0.9996 | |
| AAEL011217 | | 0.9389 | 3.6290 | 0.0087 | 0.9996 | |
| AAEL025042 | NA | 1.2621 | 4.1149 | 0.0088 | 0.9996 | NA |
| AAEL007461 | | 1.1311 | 1.5419 | 0.0090 | 0.9996 | |
| AAEL013092 | | 0.9850 | 4.7940 | 0.0091 | 0.9996 | cation chloride cotransporter [Source:VB Community Annotation] |
| AAEL026717 | NA | 1.9173 | 4.1787 | 0.0091 | 0.9996 | NA |
| AAEL003384 | | 1.7386 | 5.0123 | 0.0093 | 0.9996 | |
| AAEL026223 | NA | -1.1959 | 4.8575 | 0.0095 | 0.9996 | NA |
| AAEL001516 | | 1.4646 | 3.8488 | 0.0096 | 0.9996 | vesicle associated protein, putative [Source:VB Community Annotation] |
| AAEL006270 | | 1.6485 | 3.8243 | 0.0096 | 0.9996 | Sugar transporter SWEET [Source:UniProtKB/TrEMBL;Acc:Q176S9] |
| AAEL002933 | | 1.4021 | 6.2118 | 0.0098 | 0.9996 | |
| AAEL022395 | NA | -3.2892 | 0.1446 | 0.0098 | 0.9996 | NA |
| AAEL014763 | | 2.4626 | 0.7303 | 0.0102 | 0.9996 | tak1 binding protein-1 [Source:VB Community Annotation] |
| AAEL023900 | NA | 2.3231 | 0.1329 | 0.0105 | 0.9996 | NA |
| AAEL004694 | | -0.5575 | 5.7280 | 0.0105 | 0.9996 | munc13-4 [Source:VB Community Annotation] |
| AAEL023327 | NA | 3.0390 | 0.8284 | 0.0107 | 0.9996 | NA |
| AAEL013388 | | 1.3991 | 1.3338 | 0.0109 | 0.9996 | |
| AAEL008613 | | 0.7619 | 4.7542 | 0.0111 | 0.9996 | TraB, putative [Source:VB Community Annotation] |
| AAEL001472 | | 1.9207 | 2.8003 | 0.0113 | 0.9996 | phosphatidyltransferase [Source:VB Community Annotation] |
| AAEL014697 | | 1.0477 | 3.0669 | 0.0113 | 0.9996 | |
| AAEL025246 | NA | 2.5259 | 3.9702 | 0.0113 | 0.9996 | NA |
| AAEL011778 | | 1.4763 | 4.4637 | 0.0116 | 0.9996 | WD-repeat protein [Source:VB Community Annotation] |
| AAEL001566 | | -1.0241 | 4.1605 | 0.0117 | 0.9996 | bone morphogenetic protein [Source:VB Community Annotation] |
| AAEL000128 | | 0.9174 | 5.4322 | 0.0119 | 0.9996 | P130 [Source:VB Community Annotation] |
| AAEL022106 | NA | -2.5139 | 0.8186 | 0.0120 | 0.9996 | NA |
| AAEL004774 | | 1.0985 | 3.4209 | 0.0133 | 0.9996 | |
| AAEL011347 | | 0.9212 | 5.2810 | 0.0133 | 0.9996 | |
| AAEL012109 | | 2.2773 | 7.0792 | 0.0136 | 0.9996 | sucrose transport protein [Source:VB Community Annotation] |
| AAEL005856 | | 0.7904 | 5.7078 | 0.0137 | 0.9996 | signal recognition particle receptor alpha subunit (sr-alpha) [Source:VB Community Annotation] |
| AAEL002309 | TPX4 | 1.0996 | 8.4297 | 0.0139 | 0.9996 | Thioredoxin Peroxidase. [Source:VB Community Annotation] |

| | | | | | | |
|---|---|---|---|---|---|---|
| AAEL013866 | | 1.1970 | 4.6786 | 0.0139 | 0.9996 | WD-repeat protein [Source:VB Community Annotation] |
| AAEL022827 | NA | 1.4663 | 1.8574 | 0.0139 | 0.9996 | NA |
| AAEL021826 | NA | 0.8420 | 4.4684 | 0.0142 | 0.9996 | NA |
| AAEL000305 | | 1.5561 | 0.9025 | 0.0146 | 0.9996 | |
| AAEL012394 | | 1.1587 | 6.4808 | 0.0151 | 0.9996 | glucosidase ii beta subunit [Source:VB Community Annotation] |
| AAEL005472 | | 0.9668 | 4.1026 | 0.0151 | 0.9996 | calpain [Source:VB Community Annotation] |
| AAEL009438 | | -3.8002 | 3.0054 | 0.0155 | 0.9996 | regulator of telomere elongation helicase 1 rtel1 [Source:VB Community Annotation] |
| AAEL007009 | | 0.8892 | 4.2118 | 0.0157 | 0.9996 | |
| AAEL007439 | | 1.9329 | 6.9624 | 0.0157 | 0.9996 | myosin light chain 1, [Source:VB Community Annotation] |
| AAEL027966 | NA | 4.0064 | 0.5555 | 0.0157 | 0.9996 | NA |
| AAEL015113 | | 2.8325 | -1.1227 | 0.0158 | 0.9996 | prophenoloxidase [Source:VB Community Annotation] |
| AAEL013248 | | 0.9689 | 2.8084 | 0.0159 | 0.9996 | |
| AAEL013208 | | 1.1414 | 2.1392 | 0.0160 | 0.9996 | |
| AAEL004570 | | 0.7866 | 3.5942 | 0.0162 | 0.9996 | |
| AAEL006438 | | 0.9348 | 3.6743 | 0.0162 | 0.9996 | dolichyl glycosyltransferase [Source:VB Community Annotation] |
| AAEL009647 | | 1.2994 | 5.6451 | 0.0163 | 0.9996 | |
| AAEL008500 | | -0.8598 | 6.7823 | 0.0164 | 0.9996 | DEAD box ATP-dependent RNA helicase [Source:VB Community Annotation] |
| AAEL011626 | | 0.7091 | 3.0087 | 0.0165 | 0.9996 | DNA cross-link repair protein pso2/snm1 [Source:VB Community Annotation] |
| AAEL014204 | | 2.1348 | 3.9242 | 0.0169 | 0.9996 | 6-phosphogluconolactonase [Source:VB Community Annotation] |
| AAEL011949 | Tf2 | 0.8782 | 4.2254 | 0.0172 | 0.9996 | transferrin [Source:VB Community Annotation] |
| AAEL010324 | | 0.8453 | 4.6876 | 0.0173 | 0.9996 | |
| AAEL000118 | | 1.6218 | 3.5171 | 0.0176 | 0.9996 | cleavage and polyadenylation specificity factor [Source:VB Community Annotation] |
| AAEL009497 | | 1.4109 | 3.5981 | 0.0177 | 0.9996 | Phosphomannomutase [Source:UniProtKB/TrEMBL;Acc:Q0IEM8] |
| AAEL006655 | | -2.7512 | -0.6686 | 0.0178 | 0.9996 | chromatin regulatory protein sir2 [Source:VB Community Annotation] |
| AAEL005341 | hk | 1.3785 | 3.1291 | 0.0179 | 0.9996 | Protein hook [Source:UniProtKB/Swiss-Prot;Acc:Q17AF4 |
| AAEL013336 | | 2.1642 | 1.8281 | 0.0180 | 0.9996 | |
| AAEL020848 | NA | 1.2461 | 3.2440 | 0.0180 | 0.9996 | NA |
| AAEL000559 | | 0.8031 | 4.6393 | 0.0183 | 0.9996 | glycosyl transferase [Source:VB Community Annotation] |
| AAEL005046 | | 0.6203 | 5.8597 | 0.0187 | 0.9996 | splicing factor [Source:VB Community Annotation] |
| AAEL026439 | NA | 1.0195 | 3.3863 | 0.0188 | 0.9996 | NA |
| AAEL019579 | NA | -0.6464 | 5.5628 | 0.0192 | 0.9996 | NA |
| AAEL028705 | NA | -2.0634 | 0.7998 | 0.0194 | 0.9996 | NA |
| AAEL004678 | | 2.1493 | 4.3354 | 0.0196 | 0.9996 | rhomboid [Source:VB Community Annotation] |
| AAEL011513 | | -2.1195 | 1.6843 | 0.0198 | 0.9996 | |
| AAEL027989 | NA | -2.2174 | -0.4086 | 0.0199 | 0.9996 | NA |
| AAEL009883 | | -1.2609 | 2.8597 | 0.0199 | 0.9996 | 26S protease (S4) regulatory subunit, putative [Source:VB Community Annotation] |
| AAEL018041 | | 2.3599 | 1.2555 | 0.0202 | 0.9996 | UDP-glucuronosyltransferase [Source:UniProtKB/TrEMBL;Acc:A0A1S4G7B6] |
| AAEL007191 | | 2.2733 | 5.3233 | 0.0203 | 0.9996 | amino acid transporter [Source:VB Community Annotation] |
| AAEL014507 | | 1.4379 | 2.4484 | 0.0204 | 0.9996 | peptide n-glycanase (pngase) [Source:VB Community Annotation] |
| AAEL007304 | | 0.6230 | 4.2784 | 0.0204 | 0.9996 | |

| | | | | | | |
|---|---|---|---|---|---|---|
| AAEL003986 | | 1.1800 | 2.6683 | 0.0206 | 0.9996 | |
| AAEL002969 | | -0.8428 | 5.3902 | 0.0208 | 0.9996 | brain chitinase and chia [Source:VB Community Annotation] |
| AAEL002010 | | 0.8665 | 3.8684 | 0.0211 | 0.9996 | |
| AAEL008674 | | 0.9364 | 5.2124 | 0.0213 | 0.9996 | |
| AAEL007299 | | 1.1265 | 2.2678 | 0.0215 | 0.9996 | cadherin, putative [Source:VB Community Annotation] |
| AAEL011413 | | -3.3123 | -0.8402 | 0.0215 | 0.9996 | |
| AAEL003542 | | 0.9338 | 2.3638 | 0.0215 | 0.9996 | |
| AAEL006436 | | 1.3588 | 3.8616 | 0.0216 | 0.9996 | phosphorylase b kinase beta, kpbb [Source:VB Community Annotation] |
| AAEL007202 | HR96 | 1.0084 | 3.1475 | 0.0218 | 0.9996 | Hormone receptor-like in 96  nuclear receptor [Source:VB Community Annotation] |
| AAEL002953 | | 2.4897 | 3.3468 | 0.0222 | 0.9996 | actin 3 isoform, putative [Source:VB Community Annotation] |
| AAEL001331 | | 1.0542 | 4.8409 | 0.0231 | 0.9996 | mannose-1-phosphate guanyltransferase [Source:VB Community Annotation] |
| AAEL003911 | | 0.9738 | 3.9769 | 0.0233 | 0.9996 | |
| AAEL018707 | | 1.3879 | 0.8166 | 0.0233 | 0.9996 | |
| AAEL012551 | | 1.6248 | 1.3041 | 0.0234 | 0.9996 | |
| AAEL007229 | | 0.6767 | 4.3308 | 0.0234 | 0.9996 | Derlin [Source:UniProtKB/TrEMBL;Acc:Q172Y9] |
| AAEL014822 | | 1.1621 | 2.4160 | 0.0234 | 0.9996 | |
| AAEL020159 | NA | 1.9131 | 0.6132 | 0.0235 | 0.9996 | NA |
| AAEL021114 | NA | 2.7069 | -1.4575 | 0.0236 | 0.9996 | NA |
| AAEL024267 | NA | 1.0482 | 2.6829 | 0.0236 | 0.9996 | NA |
| AAEL026423 | NA | 2.2662 | 0.6346 | 0.0242 | 0.9996 | NA |
| AAEL003543 | | 2.0825 | 1.8343 | 0.0243 | 0.9996 | |
| AAEL012310 | | 0.9278 | 4.9327 | 0.0244 | 0.9996 | actin [Source:VB Community Annotation] |
| AAEL007717 | | 0.6134 | 5.1065 | 0.0244 | 0.9996 | alpha-endosulfine, putative [Source:VB Community Annotation] |
| AAEL012020 | | 0.9161 | 3.0008 | 0.0245 | 0.9996 | |
| AAEL009727 | | 1.0852 | 2.7690 | 0.0248 | 0.9996 | |
| AAEL025564 | NA | -2.3123 | 0.1430 | 0.0249 | 0.9996 | NA |
| AAEL027458 | NA | -1.0890 | 2.7160 | 0.0251 | 0.9996 | NA |
| AAEL002504 | | -1.4951 | 6.0878 | 0.0254 | 0.9996 | ATP synthase delta chain, mitochondrial [Source:VB Community Annotation] |
| AAEL002178 | | -1.0396 | 6.0477 | 0.0255 | 0.9996 | |
| AAEL013494 | | 0.6261 | 6.6431 | 0.0259 | 0.9996 | |
| AAEL018710 | | 1.0799 | 5.1936 | 0.0261 | 0.9996 | |
| AAEL010355 | | 2.0066 | 1.7789 | 0.0261 | 0.9996 | |
| AAEL002272 | | -0.9745 | 5.7349 | 0.0267 | 0.9996 | splicing factor yt521-b [Source:VB Community Annotation] |
| AAEL003921 | | -0.8350 | 4.5285 | 0.0269 | 0.9996 | ubiquitin, putative [Source:VB Community Annotation] |
| AAEL007238 | | 1.8272 | 2.9676 | 0.0271 | 0.9996 | |
| AAEL022757 | NA | 0.7362 | 5.1697 | 0.0277 | 0.9996 | NA |
| AAEL000451 | | 0.6695 | 6.0872 | 0.0279 | 0.9996 | beta1,4 mannosyltransferase [Source:VB Community Annotation] |
| AAEL004448 | | 0.7145 | 4.6738 | 0.0279 | 0.9996 | torsin a [Source:VB Community Annotation] |
| AAEL005962 | | -1.5994 | 0.9472 | 0.0279 | 0.9996 | Condensin complex subunit 2 [Source:UniProtKB/TrEMBL;Acc:Q178C0] |
| AAEL002512 | | 0.6196 | 4.2216 | 0.0281 | 0.9996 | |

| | | | | | | |
|---|---|---|---|---|---|---|
| AAEL024677 | NA | 2.0213 | 0.1752 | 0.0283 | 0.9996 | NA |
| AAEL005631 | | 2.4416 | 3.5924 | 0.0284 | 0.9996 | |
| AAEL005585 | | 1.0468 | 3.0461 | 0.0284 | 0.9996 | |
| AAEL010697 | | 1.8471 | 7.4336 | 0.0285 | 0.9996 | 3-ketoacyl-coa thiolase, mitochondrial (beta- ketothiolase) (acetyl-coa acyltransferase) (mitochondrial 3-oxoacyl- coa thiolase) [Source:V |
| AAEL023684 | NA | 0.6490 | 4.7065 | 0.0287 | 0.9996 | NA |
| AAEL024025 | NA | 2.2921 | -1.2419 | 0.0288 | 0.9996 | NA |
| AAEL009429 | | 0.7578 | 3.8058 | 0.0288 | 0.9996 | |
| AAEL009579 | | 1.5960 | 2.7777 | 0.0289 | 0.9996 | NBP2b protein, putative [Source:VB Community Annotation] |
| AAEL010682 | | 1.3439 | 1.5080 | 0.0292 | 0.9996 | armc4 [Source:VB Community Annotation] |
| AAEL002777 | | 1.4824 | 3.5818 | 0.0293 | 0.9996 | |
| AAEL006311 | | 0.7422 | 4.6274 | 0.0294 | 0.9996 | nucleoporin, putative [Source:VB Community Annotation] |
| AAEL006473 | | 0.7381 | 6.0367 | 0.0294 | 0.9996 | arginine/serine-rich splicing factor [Source:VB Community Annotation] |
| AAEL001821 | | 1.0446 | 1.7181 | 0.0295 | 0.9996 | |
| AAEL008263 | | -0.6666 | 4.8908 | 0.0295 | 0.9996 | |
| AAEL001647 | | 1.3058 | 4.9267 | 0.0298 | 0.9996 | |
| AAEL021071 | NA | 1.0895 | 4.9153 | 0.0300 | 0.9996 | NA |
| AAEL007683 | | -0.8197 | 4.3976 | 0.0301 | 0.9996 | prokaryotic DNA topoisomerase [Source:VB Community Annotation] |
| AAEL000084 | | 0.6666 | 6.6218 | 0.0305 | 0.9996 | elongin b [Source:VB Community Annotation] |
| AAEL001312 | CYP9M6 | 0.9664 | 4.9664 | 0.0307 | 0.9996 | cytochrome P450 [Source:VB Community Annotation] |
| AAEL000194 | | 0.6951 | 6.8107 | 0.0310 | 0.9996 | phosphatidylinositol 4-kinase [Source:VB Community Annotation] |
| AAEL002913 | | -0.5747 | 6.0435 | 0.0310 | 0.9996 | peroxisomal membrane protein 70 abcd3 [Source:VB Community Annotation] |
| AAEL028047 | NA | 2.0472 | 3.8427 | 0.0310 | 0.9996 | NA |
| AAEL025051 | NA | -0.5240 | 6.3593 | 0.0313 | 0.9996 | NA |
| AAEL027113 | NA | 1.3974 | 2.6921 | 0.0314 | 0.9996 | NA |
| AAEL004333 | | -0.9721 | 1.6064 | 0.0321 | 0.9996 | pax neighbour protein [Source:VB Community Annotation] |
| AAEL006080 | | 1.2488 | 2.7700 | 0.0321 | 0.9996 | DNA repair protein rad51 [Source:VB Community Annotation] |
| AAEL000994 | | 0.6653 | 5.5683 | 0.0322 | 0.9996 | |
| AAEL019950 | NA | -1.0509 | 6.1962 | 0.0324 | 0.9996 | NA |
| AAEL001573 | | 0.8964 | 5.1866 | 0.0326 | 0.9996 | |
| AAEL016071 | tRNA-Ser | -1.9354 | -0.1718 | 0.0329 | 0.9996 | |
| AAEL010405 | | -0.6863 | 5.6078 | 0.0330 | 0.9996 | alkyldihydroxyacetonephosphate synthase [Source:VB Community Annotation] |
| AAEL026797 | NA | 2.1874 | -0.8382 | 0.0333 | 0.9996 | NA |
| AAEL008319 | | 0.8602 | 3.8963 | 0.0333 | 0.9996 | protein disulfide isomerase [Source:VB Community Annotation] |
| AAEL006526 | | -0.5885 | 9.0844 | 0.0336 | 0.9996 | neurotransmitter gated ion channel [Source:VB Community Annotation] |
| AAEL026169 | NA | 0.6699 | 4.7864 | 0.0336 | 0.9996 | NA |
| AAEL002555 | | -0.7978 | 3.9163 | 0.0337 | 0.9996 | sodium/solute symporter [Source:VB Community Annotation] |
| AAEL006166 | | 2.2376 | 2.1410 | 0.0337 | 0.9996 | DNA (cytosine-5)-methyltransferase [Source:VB Community Annotation] |
| AAEL025241 | NA | 2.4400 | -0.3471 | 0.0340 | 0.9996 | NA |
| AAEL004276 | | 1.9107 | 0.9776 | 0.0342 | 0.9996 | |
| AAEL000113 | | -2.1408 | -0.8933 | 0.0342 | 0.9996 | |

| | | | | | | |
|---|---|---|---|---|---|---|
| AAEL001339 | | 0.5519 | 5.5610 | 0.0343 | 0.9996 | |
| AAEL006039 | | 0.7921 | 4.4168 | 0.0344 | 0.9996 | dehydrodolichyl diphosphate synthase (dedol-pp synthase) [Source:VB Community Annotation] |
| AAEL025202 | NA | 0.6853 | 4.4969 | 0.0345 | 0.9996 | NA |
| AAEL004985 | | 0.9551 | 7.4821 | 0.0350 | 0.9996 | house keepingprotein, putative [Source:VB Community Annotation] |
| AAEL012899 | | 1.0768 | 2.0008 | 0.0351 | 0.9996 | meiotic coiled-coil protein, putative [Source:VB Community Annotation] |
| AAEL024085 | NA | 2.2822 | 0.1115 | 0.0351 | 0.9996 | NA |
| AAEL010764 | | 1.4322 | 5.5232 | 0.0352 | 0.9996 | aldehyde dehydrogenase [Source:VB Community Annotation] |
| AAEL012038 | | 1.2280 | 3.6793 | 0.0353 | 0.9996 | |
| AAEL005339 | | 0.7328 | 5.1477 | 0.0353 | 0.9996 | hepatocyte growth factor-regulated tyrosine kinase substrate (hgs) [Source:VB Community Annotation] |
| AAEL006856 | | 1.5052 | 1.2634 | 0.0354 | 0.9996 | |
| AAEL026062 | NA | 1.4392 | 1.9950 | 0.0354 | 0.9996 | NA |
| AAEL008757 | | -1.7949 | 3.5638 | 0.0354 | 0.9996 | Carboxylic ester hydrolase (Fragment) [Source:UniProtKB/TrEMBL;Acc:Q16XU6] |
| AAEL009351 | | -1.2852 | 6.3939 | 0.0355 | 0.9996 | thioredoxin, putative [Source:VB Community Annotation] |
| AAEL001891 | | 2.1872 | 1.4758 | 0.0357 | 0.9996 | |
| AAEL012465 | | 1.0222 | 2.5928 | 0.0361 | 0.9996 | |
| AAEL008583 | | -0.7290 | 2.9851 | 0.0362 | 0.9996 | |
| AAEL003045 | Or41 | 2.2648 | -1.0544 | 0.0362 | 0.9996 | Odorant receptor [Source:UniProtKB/TrEMBL;Acc:Q0IGC4] |
| AAEL010794 | | 0.5249 | 6.1541 | 0.0365 | 0.9996 | prenylated rab acceptor [Source:VB Community Annotation] |
| AAEL018207 | CTL8 | -1.6483 | 0.8123 | 0.0366 | 0.9996 | C-Type lectin (CTL) [Source:VB Community Annotation] |
| AAEL006321 | | -1.0682 | 4.9540 | 0.0368 | 0.9996 | 1-acylglycerol-3-phosphate acyltransferase [Source:VB Community Annotation] |
| AAEL007811 | | 0.9498 | 2.5450 | 0.0369 | 0.9996 | short-chain dehydrogenase [Source:VB Community Annotation] |
| AAEL002753 | | 0.9336 | 1.9156 | 0.0370 | 0.9996 | zinc finger protein [Source:VB Community Annotation] |
| AAEL019796 | NA | 0.6796 | 4.1749 | 0.0372 | 0.9996 | NA |
| AAEL006928 | | 1.9450 | 6.5989 | 0.0373 | 0.9996 | dihydrolipoamide dehydrogenase [Source:VB Community Annotation] |
| AAEL005037 | | 0.5941 | 5.7525 | 0.0376 | 0.9996 | seryl-tRNA synthetase [Source:VB Community Annotation] |
| AAEL007842 | | 0.6328 | 5.3514 | 0.0377 | 0.9996 | |
| AAEL011566 | | -1.2156 | 5.3062 | 0.0378 | 0.9996 | cell adhesion molecule [Source:VB Community Annotation] |
| AAEL006025 | | 0.5091 | 5.4320 | 0.0378 | 0.9996 | |
| AAEL003075 | | 0.7820 | 3.8623 | 0.0378 | 0.9996 | |
| AAEL021695 | NA | 2.3558 | 2.6419 | 0.0379 | 0.9996 | NA |
| AAEL022617 | NA | 1.7849 | 4.0241 | 0.0382 | 0.9996 | NA |
| AAEL008806 | | 0.9958 | 5.7056 | 0.0383 | 0.9996 | testis development protein prtd [Source:VB Community Annotation] |
| AAEL001295 | | -0.9855 | 5.8727 | 0.0383 | 0.9996 | |
| AAEL000137 | | -0.7884 | 2.6723 | 0.0384 | 0.9996 | |
| AAEL000203 | | 1.7611 | -0.7581 | 0.0385 | 0.9996 | trypsin [Source:VB Community Annotation] |
| AAEL007891 | | 1.4134 | 4.9430 | 0.0387 | 0.9996 | xtp3-transactivated protein b [Source:VB Community Annotation] |
| AAEL021061 | NA | 1.3156 | 1.8576 | 0.0387 | 0.9996 | NA |
| AAEL009192 | SCRASP1 | -1.3406 | 4.8423 | 0.0390 | 0.9996 | Class A Scavenger Receptor (SRCR domain) with Serine Protease domain. [Source:VB Community Annotation] |
| AAEL012595 | | 0.8848 | 5.1382 | 0.0390 | 0.9996 | |
| AAEL009100 | | 1.8192 | 0.4774 | 0.0393 | 0.9996 | mitochondrial oxodicarboxylate carrier [Source:VB Community Annotation] |

| | | | | | | |
|---|---|---|---|---|---|---|
| AAEL002465 | | 0.7591 | 3.7132 | 0.0393 | 0.9996 | |
| AAEL007725 | | 0.5728 | 4.9513 | 0.0394 | 0.9996 | reticulocalbin [Source:VB Community Annotation] |
| AAEL008291 | | 0.7438 | 7.1320 | 0.0396 | 0.9996 | |
| AAEL013995 | APG18B | 0.7553 | 6.0299 | 0.0396 | 0.9996 | autophagy related gene [Source:VB Community Annotation] |
| AAEL011078 | CTLGA1 | -1.0781 | 7.9197 | 0.0399 | 0.9996 | C-Type Lectin (CTL) - galactose binding. [Source:VB Community Annotation] |
| AAEL019663 | NA | 0.8637 | 1.7686 | 0.0403 | 0.9996 | NA |
| AAEL008587 | | 1.4684 | 6.5565 | 0.0405 | 0.9996 | glutamate receptor, ionotropic, N-methyl d-aspartate [Source:VB Community Annotation] |
| AAEL007136 | | 2.3446 | 2.5653 | 0.0407 | 0.9996 | sugar transporter [Source:VB Community Annotation] |
| AAEL001124 | | 0.8655 | 3.3568 | 0.0408 | 0.9996 | |
| AAEL021573 | NA | -0.5998 | 4.3788 | 0.0408 | 0.9996 | NA |
| AAEL020585 | NA | 2.0646 | -1.4433 | 0.0409 | 0.9996 | NA |
| AAEL027116 | NA | 1.2905 | 2.5183 | 0.0410 | 0.9996 | NA |
| AAEL021984 | NA | -2.1037 | 0.3213 | 0.0410 | 0.9996 | NA |
| AAEL000677 | | 0.9121 | 4.3791 | 0.0411 | 0.9996 | DNA-directed RNA polymerase subunit rpb8 [Source:VB Community Annotation] |
| AAEL026241 | NA | 1.1681 | 2.3731 | 0.0412 | 0.9996 | NA |
| AAEL002351 | | 0.8898 | 3.8314 | 0.0415 | 0.9996 | DEAD box ATP-dependent RNA helicase [Source:VB Community Annotation] |
| AAEL001670 | | 0.6534 | 5.7073 | 0.0417 | 0.9996 | |
| AAEL026958 | NA | 2.2789 | -0.9106 | 0.0419 | 0.9996 | NA |
| AAEL009477 | | 1.7029 | 0.4489 | 0.0420 | 0.9996 | |
| AAEL008814 | | 0.8397 | 3.1820 | 0.0421 | 0.9996 | |
| AAEL003562 | | 0.7861 | 2.6588 | 0.0423 | 0.9996 | polypeptide of 976 aa, putative [Source:VB Community Annotation] |
| AAEL001800 | | 0.5997 | 4.7890 | 0.0425 | 0.9996 | hsp70 binding protein [Source:VB Community Annotation] |
| AAEL012881 | | 0.9709 | 2.4834 | 0.0427 | 0.9996 | |
| AAEL000214 | | 0.6025 | 3.3905 | 0.0427 | 0.9996 | vacuolar protein sorting (vps33) [Source:VB Community Annotation] |
| AAEL008788 | | 0.6318 | 3.2434 | 0.0432 | 0.9996 | es2 protein [Source:VB Community Annotation] |
| AAEL008021 | | 1.3002 | -0.6038 | 0.0432 | 0.9996 | specifically Rac-associated protein, putative [Source:VB Community Annotation] |
| AAEL009044 | | -2.0437 | -0.4666 | 0.0433 | 0.9996 | amine oxidase [Source:VB Community Annotation] |
| AAEL000928 | | -1.8561 | 2.1091 | 0.0438 | 0.9996 | Fatty acyl-CoA reductase [Source:UniProtKB/TrEMBL;Acc:Q17MY9] |
| AAEL000851 | | -0.7470 | 5.6452 | 0.0438 | 0.9996 | predicted G-protein coupled receptor (GPCR) [Source:VB Community Annotation] |
| AAEL010788 | | -1.0958 | 1.8014 | 0.0439 | 0.9996 | metalloprotease [Source:VB Community Annotation] |
| AAEL011702 | | 0.9953 | 3.7635 | 0.0440 | 0.9996 | |
| AAEL009811 | | 1.3471 | 4.2208 | 0.0442 | 0.9996 | |
| AAEL015222 | | -1.4863 | 0.7741 | 0.0443 | 0.9996 | adult cuticle protein, putative [Source:VB Community Annotation] |
| AAEL028005 | NA | 0.9507 | 1.9201 | 0.0443 | 0.9996 | NA |
| AAEL014183 | | 0.4211 | 5.4336 | 0.0445 | 0.9996 | |
| AAEL002564 | | -2.1411 | -0.2391 | 0.0447 | 0.9996 | |
| AAEL022059 | NA | 2.1055 | -0.9024 | 0.0448 | 0.9996 | NA |
| AAEL010015 | | 0.8142 | 5.4434 | 0.0448 | 0.9996 | |
| AAEL010123 | | 1.2043 | 5.1995 | 0.0449 | 0.9996 | equilibrative nucleoside transporter [Source:VB Community Annotation] |
| AAEL027174 | NA | 0.6944 | 3.8684 | 0.0450 | 0.9996 | NA |

| | | | | | | |
|---|---|---|---|---|---|---|
| AAEL008700 | | 1.0813 | 4.4536 | 0.0451 | 0.9996 | |
| AAEL007298 | | -0.9402 | 7.4799 | 0.0451 | 0.9996 | Fatty acyl-CoA reductase [Source:UniProtKB/TrEMBL;Acc:Q172Q8] |
| AAEL012767 | LRIM5 | -2.1929 | 0.2536 | 0.0451 | 0.9996 | leucine-rich immune protein (Short) [Source:VB Community Annotation] |
| AAEL006547 | | 0.8700 | 4.1771 | 0.0453 | 0.9996 | THO complex, putative [Source:VB Community Annotation] |
| AAEL007660 | | -0.8365 | 6.0200 | 0.0453 | 0.9996 | suppressor of actin (sac) [Source:VB Community Annotation] |
| AAEL004352 | | 1.9183 | 1.7850 | 0.0455 | 0.9996 | |
| AAEL003634 | | -0.5080 | 6.5186 | 0.0455 | 0.9996 | Hsp70-interacting protein, putative [Source:VB Community Annotation] |
| AAEL003336 | | 0.9174 | 5.2673 | 0.0455 | 0.9996 | tRNA-splicing ligase RtcB homolog [Source:UniProtKB/Swiss-Prot;Acc:Q17FP1] |
| AAEL003203 | | -0.6925 | 11.5678 | 0.0456 | 0.9996 | fatty acid desaturase, putative [Source:VB Community Annotation] |
| AAEL006145 | | 0.4692 | 5.0171 | 0.0457 | 0.9996 | dynactin [Source:VB Community Annotation] |
| AAEL009567 | | 0.8897 | 2.3469 | 0.0458 | 0.9996 | apolipoprotein D, putative [Source:VB Community Annotation] |
| AAEL013839 | | 0.7173 | 3.8877 | 0.0458 | 0.9996 | |
| AAEL012709 | | 1.4799 | 2.5500 | 0.0459 | 0.9996 | |
| AAEL017566 | | 0.9447 | 2.3912 | 0.0459 | 0.9996 | DNA ligase [Source:UniProtKB/TrEMBL;Acc:A0A1S4G6J7] |
| AAEL010467 | | -0.5772 | 7.2370 | 0.0460 | 0.9996 | heterogeneous nuclear ribonucleoprotein [Source:VB Community Annotation] |
| AAEL008041 | | 0.5253 | 5.3359 | 0.0460 | 0.9996 | bleomycin hydrolase [Source:VB Community Annotation] |
| AAEL019699 | NA | 1.1607 | 3.4711 | 0.0461 | 0.9996 | NA |
| AAEL006847 | | -0.7996 | 5.4625 | 0.0461 | 0.9996 | Defective in cullin neddylation protein [Source:UniProtKB/TrEMBL;Acc:Q174N9 |
| AAEL027706 | NA | 1.2790 | 3.1469 | 0.0466 | 0.9996 | NA |
| AAEL006155 | | -1.1183 | 4.3622 | 0.0468 | 0.9996 | Nicalin [Source:UniProtKB/TrEMBL;Acc:Q177H2] |
| AAEL002103 | | -2.3843 | -2.0008 | 0.0470 | 0.9996 | histone H1, putative [Source:VB Community Annotation] |
| AAEL004327 | | 0.4976 | 5.6040 | 0.0471 | 0.9996 | valyl-tRNA synthetase [Source:VB Community Annotation] |
| AAEL012105 | | 0.9219 | 4.8337 | 0.0472 | 0.9996 | Zinc finger protein-like 1 homolog [Source:UniProtKB/Swiss-Prot;Acc:Q16N38] |
| AAEL006698 | RpL31 | -0.9867 | 9.9218 | 0.0473 | 0.9996 | 60S ribosomal protein L31 [Source:UniProtKB/Swiss-Prot;Acc:Q9GN74] |
| AAEL011140 | | -0.5080 | 6.4323 | 0.0474 | 0.9996 | |
| AAEL018048 | | 1.3291 | 1.2418 | 0.0474 | 0.9996 | |
| AAEL006805 | CYP9J2 | 1.5692 | 2.9501 | 0.0479 | 0.9996 | cytochrome P450 [Source:VB Community Annotation] |
| AAEL008647 | | 2.1288 | 0.5029 | 0.0481 | 0.9996 | no-mechanoreceptor potential a [Source:VB Community Annotation] |
| AAEL005701 | | -0.6635 | 3.7651 | 0.0484 | 0.9996 | retinaldehyde binding protein [Source:VB Community Annotation] |
| AAEL007822 | | 0.8062 | 5.1538 | 0.0484 | 0.9996 | ubiquitin-conjugating enzyme E2 g [Source:VB Community Annotation] |
| AAEL020827 | NA | -2.6621 | -0.5391 | 0.0484 | 0.9996 | NA |
| AAEL006016 | | -0.4325 | 6.0715 | 0.0484 | 0.9996 | |
| AAEL010214 | | 1.4366 | 0.4272 | 0.0484 | 0.9996 | |
| AAEL009039 | | 1.7719 | 3.5312 | 0.0484 | 0.9996 | AP-3 complex subunit beta [Source:UniProtKB/TrEMBL;Acc:A0A1S4FLH9] |
| AAEL003401 | | 2.3881 | 2.7706 | 0.0485 | 0.9996 | DNA-directed RNA polymerase II 19 kDa polypeptide rpb7 [Source:VB Community Annotation] |
| AAEL006374 | | 0.9978 | 3.6416 | 0.0486 | 0.9996 | |
| AAEL019770 | NA | -0.5859 | 4.2488 | 0.0486 | 0.9996 | NA |
| AAEL003260 | | -0.7431 | 4.7430 | 0.0487 | 0.9996 | |
| AAEL013074 | | 0.5775 | 6.2041 | 0.0487 | 0.9996 | Adenylyl cyclase-associated protein [Source:UniProtKB/TrEMBL;Acc:Q16K93] |
| AAEL007656 | | -1.2943 | 2.4560 | 0.0489 | 0.9996 | receptor for activated C kinase, putative [Source:VB Community Annotation] |

| | | | | | | |
|---|---|---|---|---|---|---|
| AAEL009817 | | 1.1241 | 3.9405 | 0.0489 | 0.9996 | |
| AAEL002607 | | 1.7848 | 0.6402 | 0.0489 | 0.9996 | |
| AAEL003503 | | 0.6410 | 6.8395 | 0.0491 | 0.9996 | ras-related protein Rab-7 [Source:VB Community Annotation] |
| AAEL002215 | | 0.7152 | 3.6651 | 0.0491 | 0.9996 | |
| AAEL007022 | | 0.5789 | 5.8119 | 0.0491 | 0.9996 | tfiia large subunit [Source:VB Community Annotation] |
| AAEL025705 | NA | -1.9406 | -0.5879 | 0.0492 | 0.9996 | NA |
| AAEL020700 | NA | -1.2958 | 2.3100 | 0.0493 | 0.9996 | NA |
| AAEL009915 | | -1.9320 | -1.2550 | 0.0494 | 0.9996 | centrosomal protein [Source:VB Community Annotation] |
| AAEL003149 | | 1.3223 | 1.3954 | 0.0495 | 0.9996 | |
| AAEL028097 | NA | -1.8229 | 2.6890 | 0.0498 | 0.9996 | NA |
| AAEL024958 | NA | 0.6208 | 2.6950 | 0.0499 | 0.9996 | NA |
| AAEL013250 | | 0.6744 | 3.4034 | 0.0500 | 0.9996 | |
| AAEL011850 | CYP315A1 | 0.7737 | 3.9416 | 0.0501 | 0.9996 | cytochrome P450 [Source:VB Community Annotation] |
| AAEL013240 | | 0.8025 | 2.7222 | 0.0501 | 0.9996 | |
| AAEL010951 | | 0.8916 | 3.7731 | 0.0503 | 0.9996 | glutamate decarboxylase [Source:VB Community Annotation] |
| AAEL009644 | | 1.1506 | 3.9668 | 0.0503 | 0.9996 | SAGA-associated factor 11 [Source:UniProtKB/TrEMBL;Acc:Q16V87] |
| AAEL010877 | | -1.3972 | 0.2207 | 0.0503 | 0.9996 | |
| AAEL008767 | | -2.3993 | -2.2340 | 0.0506 | 0.9996 | serine protease [Source:VB Community Annotation] |
| AAEL001971 | | 0.6674 | 3.6825 | 0.0506 | 0.9996 | |
| AAEL003518 | | -1.5376 | 2.5801 | 0.0506 | 0.9996 | calcium-transporting ATPase 2 (ATPase 2) [Source:VB Community Annotation] |
| AAEL002995 | | 0.6315 | 5.2261 | 0.0508 | 0.9996 | pyrazinamidase/nicotinamidase [Source:VB Community Annotation] |
| AAEL007268 | | -1.9116 | 2.2959 | 0.0509 | 0.9996 | |
| AAEL008049 | | 0.9813 | 2.1192 | 0.0511 | 0.9996 | |
| AAEL007407 | | 0.6516 | 6.5734 | 0.0515 | 0.9996 | |
| AAEL003277 | | 0.6380 | 3.5323 | 0.0516 | 0.9996 | |
| AAEL005978 | | -1.0885 | 5.6086 | 0.0516 | 0.9996 | |
| AAEL002531 | | 1.7518 | 1.4318 | 0.0516 | 0.9996 | |
| AAEL002835 | | 0.5013 | 4.8542 | 0.0518 | 0.9996 | |
| AAEL001490 | | 1.6656 | 4.0294 | 0.0520 | 0.9996 | acylphosphatase, putative [Source:VB Community Annotation] |
| AAEL023909 | NA | -1.9247 | -1.0005 | 0.0521 | 0.9996 | NA |
| AAEL003889 | GNBPB1 | -1.2390 | 7.6991 | 0.0522 | 0.9996 | Gram-Negative Binding Protein (GNBP) or Beta-1 3-Glucan Binding Protein (BGBP). [Source:VB Community Annotation] |
| AAEL014567 | | -0.6505 | 2.8240 | 0.0527 | 0.9996 | oviductin [Source:VB Community Annotation] |
| AAEL024343 | NA | -0.8922 | 6.8496 | 0.0528 | 0.9996 | NA |
| AAEL007134 | | -1.4996 | 3.7370 | 0.0531 | 0.9996 | |
| AAEL015065 | | 0.4706 | 6.3541 | 0.0531 | 0.9996 | spectrin [Source:VB Community Annotation] |
| AAEL006854 | | -0.5845 | 5.6147 | 0.0533 | 0.9996 | Niemann-Pick Type C-2, putative [Source:VB Community Annotation] |
| AAEL001170 | | 0.6418 | 4.7878 | 0.0534 | 0.9996 | Protein phosphatase methylesterase 1 [Source:UniProtKB/TrEMBL;Acc:Q17M26] |
| AAEL003647 | GPRNPR3 | 1.0678 | 1.2591 | 0.0536 | 0.9996 | GPCR Neurokinin/Tachykinin Family [Source:VB Community Annotation] |
| AAEL009302 | cbc | 0.9911 | 3.0490 | 0.0536 | 0.9996 | Protein CLP1 homolog [Source:UniProtKB/Swiss-Prot;Acc:Q16WA6] |
| AAEL003549 | | 0.9987 | 2.2912 | 0.0538 | 0.9996 | |

| | | | | | | |
|---|---|---|---|---|---|---|
| AAEL011460 | | -1.0293 | 3.0917 | 0.0540 | 0.9996 | |
| AAEL008420 | | 0.9112 | 2.3956 | 0.0542 | 0.9996 | |
| AAEL005279 | | 1.1641 | 1.7016 | 0.0542 | 0.9996 | methionyl-tRNA formyltransferase [Source:VB Community Annotation] |
| AAEL008564 | cue | -0.7285 | 3.7422 | 0.0543 | 0.9996 | Protein cueball Precursor [Source:VB Community Annotation] |
| AAEL010761 | | 0.5089 | 4.7874 | 0.0544 | 0.9996 | GRIP and coiled-coil domain-containing protein 1 , putative [Source:VB Community Annotation] |
| AAEL011745 | | -0.5483 | 4.9119 | 0.0546 | 0.9996 | trisn small nuclear ribonucleoprotein, putative [Source:VB Community Annotation] |
| AAEL015442 | | 0.7525 | 2.0363 | 0.0548 | 0.9996 | |
| AAEL013527 | snRNP-U1- | 0.5743 | 5.3485 | 0.0549 | 0.9996 | U1 small nuclear ribonucleoprotein C [Source:UniProtKB/Swiss-Prot;Acc:Q16IW3] |
| AAEL021579 | NA | 0.8987 | 3.1352 | 0.0549 | 0.9996 | NA |
| AAEL006567 | | -0.9852 | 6.5848 | 0.0550 | 0.9996 | max binding protein, mnt [Source:VB Community Annotation] |
| AAEL013534 | | 1.5111 | 4.9788 | 0.0553 | 0.9996 | tgf-beta resistance-associated protein trag [Source:VB Community Annotation] |
| AAEL011481 | OBP54 | 2.2134 | 0.0192 | 0.0556 | 0.9996 | odorant binding protein OBP43 [Source:VB Community Annotation] |
| AAEL014062 | | 0.8226 | 3.8018 | 0.0557 | 0.9996 | |
| AAEL021771 | NA | 0.7188 | 4.1154 | 0.0559 | 0.9996 | NA |
| AAEL010151 | CYP6N16 | 0.8890 | 5.1847 | 0.0562 | 0.9996 | cytochrome P450 [Source:VB Community Annotation] |
| AAEL015393 | | 1.7270 | 1.7784 | 0.0567 | 0.9996 | |
| AAEL001922 | | -1.3891 | -0.0768 | 0.0567 | 0.9996 | |
| AAEL003729 | | -0.8771 | 2.8058 | 0.0572 | 0.9996 | Probable hydroxyacid-oxoacid transhydrogenase, mitochondrial Precursor (HOT)(EC 1.1.99.24) [Source:VB Community Annotation] |
| AAEL015571 | | 1.3231 | 2.2395 | 0.0573 | 0.9996 | |
| AAEL012359 | | -0.8785 | 8.1214 | 0.0573 | 0.9996 | nucleoside-diphosphate kinase NBR-A, putative [Source:VB Community Annotation] |
| AAEL012549 | | 1.0852 | 1.6343 | 0.0574 | 0.9996 | ATPase, class vi, type 11c [Source:VB Community Annotation] |
| AAEL005471 | | 0.5379 | 8.6488 | 0.0577 | 0.9996 | Sec61 protein complex gamma subunit, putative [Source:VB Community Annotation] |
| AAEL009466 | | -1.6830 | 2.5270 | 0.0577 | 0.9996 | protein kinase c [Source:VB Community Annotation] |
| AAEL025082 | NA | -1.6318 | 3.0523 | 0.0579 | 0.9996 | NA |
| AAEL008656 | | 0.8537 | 3.5609 | 0.0579 | 0.9996 | UDP-galactose transporter [Source:VB Community Annotation] |
| AAEL028112 | NA | 0.7309 | 3.1250 | 0.0582 | 0.9996 | NA |
| AAEL011095 | | 0.6064 | 4.9605 | 0.0582 | 0.9996 | n-acetylgalactosaminyltransferase [Source:VB Community Annotation] |
| AAEL025966 | NA | 0.5038 | 6.2405 | 0.0584 | 0.9996 | NA |
| AAEL023984 | NA | -1.6226 | 0.5069 | 0.0588 | 0.9996 | NA |
| AAEL012698 | | 0.7357 | 4.9925 | 0.0589 | 0.9996 | ATP-binding cassette sub-family A member 3, putative [Source:VB Community Annotation] |
| AAEL021576 | NA | -1.4284 | 5.4683 | 0.0589 | 0.9996 | NA |
| AAEL013541 | | 0.6109 | 5.1757 | 0.0589 | 0.9996 | syntaxin [Source:VB Community Annotation] |
| AAEL004936 | | 0.5051 | 5.5289 | 0.0591 | 0.9996 | |
| AAEL013999 | | 0.4712 | 5.0669 | 0.0591 | 0.9996 | |
| AAEL024199 | NA | 0.7792 | 2.6017 | 0.0594 | 0.9996 | NA |
| AAEL004938 | | -0.5223 | 4.5375 | 0.0594 | 0.9996 | |
| AAEL002286 | APG5 | -0.4946 | 3.3703 | 0.0595 | 0.9996 | autophagy related gene [Source:VB Community Annotation] |
| AAEL011191 | | -0.8346 | 6.8320 | 0.0595 | 0.9996 | protein phosphatase 1 binding protein [Source:VB Community Annotation] |
| AAEL007484 | | 0.5992 | 7.3471 | 0.0597 | 0.9996 | protein transport protein sec23 [Source:VB Community Annotation] |
| AAEL000178 | | 0.8849 | 3.0081 | 0.0598 | 0.9996 | ubiquinone/menaquinone biosynthesis methyltransferase [Source:VB Community Annotation] |

| | | | | | | |
|---|---|---|---|---|---|---|
| AAEL027012 | NA | 0.8744 | 2.2128 | 0.0599 | 0.9996 | NA |
| AAEL008532 | | -1.5846 | 2.7814 | 0.0600 | 0.9996 | Carboxylic ester hydrolase (Fragment) [Source:UniProtKB/TrEMBL;Acc:Q16YG4] |
| AAEL014705 | | 0.5905 | 3.7694 | 0.0600 | 0.9996 | |
| AAEL006800 | | 0.8818 | 3.1220 | 0.0602 | 0.9996 | sodium/chloride dependent transporter [Source:VB Community Annotation] |
| AAEL001164 | | -0.8981 | 7.4745 | 0.0602 | 0.9996 | NADH:ubiquinone dehydrogenase, putative [Source:VB Community Annotation] |
| AAEL009749 | | 2.1641 | 2.2437 | 0.0603 | 0.9996 | sodium-dependent phosphate transporter [Source:VB Community Annotation] |
| AAEL024222 | NA | -1.4641 | 1.5359 | 0.0603 | 0.9996 | NA |
| AAEL018340 | | -0.5396 | 5.0692 | 0.0603 | 0.9996 | |
| AAEL020352 | NA | -1.4149 | 3.3612 | 0.0604 | 0.9996 | NA |
| AAEL021346 | NA | 2.4119 | -0.5198 | 0.0609 | 0.9996 | NA |
| AAEL003683 | | 0.8782 | 2.6683 | 0.0610 | 0.9996 | tRNA pseudouridine synthase [Source:UniProtKB/TrEMBL;Acc:Q17EU6] |
| AAEL027809 | NA | -0.7422 | 2.6230 | 0.0611 | 0.9996 | NA |
| AAEL023252 | NA | 0.7635 | 2.9126 | 0.0611 | 0.9996 | NA |
| AAEL006615 | | 1.5062 | 1.5527 | 0.0611 | 0.9996 | zinc finger protein [Source:VB Community Annotation] |
| AAEL004873 | | -0.6696 | 8.1087 | 0.0612 | 0.9996 | |
| AAEL002853 | | -2.2471 | 0.4348 | 0.0613 | 0.9996 | ccaat/enhancer binding protein [Source:VB Community Annotation] |
| AAEL002269 | | 2.0182 | 5.1416 | 0.0613 | 0.9996 | Purine nucleoside phosphorylase [Source:UniProtKB/TrEMBL;Acc:Q17IS2] |
| AAEL004957 | | 0.6155 | 5.7011 | 0.0618 | 0.9996 | |
| AAEL027036 | NA | -0.8073 | 3.1739 | 0.0618 | 0.9996 | NA |
| AAEL007813 | | 1.1397 | 2.3039 | 0.0620 | 0.9996 | |
| AAEL008363 | | -0.5746 | 5.1191 | 0.0623 | 0.9996 | transcription initiation factor TFIID subunit 12 [Source:VB Community Annotation] |
| AAEL001167 | | 1.0805 | 3.1402 | 0.0630 | 0.9996 | synaptotagmin [Source:VB Community Annotation] |
| AAEL000468 | Med28 | 0.6662 | 4.9950 | 0.0632 | 0.9996 | mediator of RNA polymerase II transcription subunit 28 (Med28) [Source:VB Community Annotation] |
| AAEL001706 | | -0.6116 | 4.1583 | 0.0633 | 0.9996 | p15-2a protein, putative [Source:VB Community Annotation] |
| AAEL014176 | | -0.8205 | 4.5697 | 0.0636 | 0.9996 | cornichon [Source:VB Community Annotation] |
| AAEL019896 | NA | -0.7880 | 5.2434 | 0.0636 | 0.9996 | NA |
| AAEL012268 | | 2.3868 | -0.6148 | 0.0637 | 0.9996 | brain chitinase and chia [Source:VB Community Annotation] |
| AAEL001254 | | 1.0429 | 3.6311 | 0.0637 | 0.9996 | |
| AAEL000055 | | -0.5837 | 4.9718 | 0.0638 | 0.9996 | |
| AAEL003835 | | -2.5216 | -1.7543 | 0.0638 | 0.9996 | initiation factor 5a [Source:VB Community Annotation] |
| AAEL011324 | | -0.9090 | 2.2807 | 0.0639 | 0.9996 | |
| AAEL004405 | | 1.0474 | 1.7458 | 0.0639 | 0.9996 | |
| AAEL004076 | | 0.5122 | 4.3774 | 0.0642 | 0.9996 | Ubiquitin-like modifier-activating enzyme 5 (Ubiquitin-activating enzyme 5) [Source:VB Community Annotation] |
| AAEL003262 | | -2.8852 | -1.3072 | 0.0643 | 0.9996 | leucine-rich transmembrane protein [Source:VB Community Annotation] |
| AAEL001767 | | 0.6241 | 5.0009 | 0.0646 | 0.9996 | |
| AAEL011124 | | -1.8415 | 0.3857 | 0.0647 | 0.9996 | PHD finger protein [Source:VB Community Annotation] |
| AAEL009645 | | 1.0110 | 6.0536 | 0.0648 | 0.9996 | |
| AAEL010414 | | -0.6999 | 2.5551 | 0.0649 | 0.9996 | set domain protein [Source:VB Community Annotation] |
| AAEL019678 | NA | 1.2628 | 2.7486 | 0.0650 | 0.9996 | NA |
| AAEL008770 | | -0.5645 | 6.2078 | 0.0650 | 0.9996 | proteasome subunit beta type [Source:VB Community Annotation] |

| | | | | | | |
|---|---|---|---|---|---|---|
| AAEL000912 | | -1.4945 | 0.5596 | 0.0651 | 0.9996 | Fatty acyl-CoA reductase [Source:UniProtKB/TrEMBL;Acc:Q17MZ0] |
| AAEL000450 | | 0.7648 | 5.3457 | 0.0653 | 0.9996 | ras GTPase activating protein [Source:VB Community Annotation] |
| AAEL005900 | | 0.8399 | 3.9557 | 0.0658 | 0.9996 | UPF0443 protein AAEL005900 [Source:VB Community Annotation] |
| AAEL001667 | | 1.7670 | 1.0468 | 0.0664 | 0.9996 | multicopper oxidase [Source:VB Community Annotation] |
| AAEL024787 | NA | 0.6805 | 3.0092 | 0.0665 | 0.9996 | NA |
| AAEL003390 | | -0.8257 | 2.4112 | 0.0666 | 0.9996 | |
| AAEL005839 | | 0.8814 | 7.1099 | 0.0667 | 0.9996 | uridine phosphorylase [Source:VB Community Annotation] |
| AAEL014027 | | 1.1361 | 2.5739 | 0.0668 | 0.9996 | |
| AAEL010822 | | 1.0260 | 2.9876 | 0.0670 | 0.9996 | sulphate transporter [Source:VB Community Annotation] |
| AAEL013417 | | -1.3234 | 2.1733 | 0.0672 | 0.9996 | fibrinogen and fibronectin [Source:VB Community Annotation] |
| AAEL011602 | | -1.2979 | 3.6076 | 0.0672 | 0.9996 | |
| AAEL014059 | | 0.5200 | 4.1808 | 0.0673 | 0.9996 | ancient conserved domain protein 2 (cyclin m2) [Source:VB Community Annotation] |
| AAEL014014 | | -1.5724 | 0.6627 | 0.0677 | 0.9996 | ornithine cyclodeaminase [Source:VB Community Annotation] |
| AAEL026665 | NA | -0.7531 | 4.9040 | 0.0677 | 0.9996 | NA |
| AAEL014345 | | -0.7026 | 5.3209 | 0.0678 | 0.9996 | |
| AAEL004295 | | 1.2363 | 4.5219 | 0.0678 | 0.9996 | Glucose-6-phosphatase [Source:UniProtKB/TrEMBL;Acc:Q17D95] |
| AAEL007687 | | -0.4786 | 6.1358 | 0.0679 | 0.9996 | transmembrane 9 superfamily protein member 4 [Source:VB Community Annotation] |
| AAEL024980 | NA | 1.2180 | 4.0001 | 0.0679 | 0.9996 | NA |
| AAEL000600 | | 1.0499 | 1.5515 | 0.0686 | 0.9996 | Wnt10a protein, putative [Source:VB Community Annotation] |
| AAEL025078 | NA | 0.5123 | 5.8513 | 0.0686 | 0.9996 | NA |
| AAEL013184 | | -0.6828 | 4.8721 | 0.0687 | 0.9996 | |
| AAEL009857 | | 0.4962 | 4.3432 | 0.0687 | 0.9996 | |
| AAEL023468 | NA | -1.1525 | 1.5541 | 0.0688 | 0.9996 | NA |
| AAEL005548 | | -1.9890 | -1.0989 | 0.0690 | 0.9996 | tetraspanin, putative [Source:VB Community Annotation] |
| AAEL023187 | NA | -1.0895 | 5.8161 | 0.0691 | 0.9996 | NA |
| AAEL001545 | | 0.7324 | 2.7014 | 0.0693 | 0.9996 | |
| AAEL003740 | | 1.6967 | -0.2440 | 0.0693 | 0.9996 | |
| AAEL001088 | | 0.4691 | 5.1275 | 0.0696 | 0.9996 | beta-1,3-galactosyltransferase [Source:VB Community Annotation] |
| AAEL000652 | GNBPA2 | -1.5316 | 0.0883 | 0.0696 | 0.9996 | Gram-Negative Binding Protein (GNBP) or Beta-1 3-Glucan Binding Protein (BGBP). [Source:VB Community Annotation] |
| AAEL024684 | NA | 1.7202 | 0.8108 | 0.0698 | 0.9996 | NA |
| AAEL007862 | | -0.7792 | 2.8037 | 0.0700 | 0.9996 | |
| AAEL009719 | | 0.5607 | 6.6697 | 0.0704 | 0.9996 | |
| AAEL024200 | NA | 1.0729 | 1.7366 | 0.0706 | 0.9996 | NA |
| AAEL010771 | | -1.0079 | 3.4373 | 0.0706 | 0.9996 | |
| AAEL001191 | | 0.7730 | 3.5438 | 0.0708 | 0.9996 | |
| AAEL005629 | RpL7a | -0.6081 | 10.4317 | 0.0710 | 0.9996 | 60S ribosomal protein L35, putative [Source:VB Community Annotation] |
| AAEL010678 | | 1.7602 | 4.6793 | 0.0711 | 0.9996 | |
| AAEL017443 | | -1.3048 | 0.2766 | 0.0711 | 0.9996 | |
| AAEL022897 | NA | 0.4726 | 5.3343 | 0.0711 | 0.9996 | NA |
| AAEL027114 | NA | 2.0351 | -1.8238 | 0.0712 | 0.9996 | NA |

| | | | | | | |
|---|---|---|---|---|---|---|
| AAEL009090 | | 0.8378 | 2.7187 | 0.0717 | 0.9996 | U6 snRNA phosphodiesterase [Source:UniProtKB/TrEMBL;Acc:Q16WV0] |
| AAEL012297 | | -0.8263 | 5.6348 | 0.0718 | 0.9996 | sterol desaturase [Source:VB Community Annotation] |
| AAEL006929 | | 0.5546 | 5.3745 | 0.0721 | 0.9996 | cullin [Source:VB Community Annotation] |
| AAEL008528 | | -0.5132 | 6.2132 | 0.0722 | 0.9996 | protein tyrosine phosphatase n11 (shp2) [Source:VB Community Annotation] |
| AAEL028088 | NA | 1.1879 | 2.0508 | 0.0722 | 0.9996 | NA |
| AAEL004688 | | 0.6210 | 5.3001 | 0.0725 | 0.9996 | |
| AAEL012172 | | -0.5766 | 5.8789 | 0.0725 | 0.9996 | methylthioadenosine phosphorylase [Source:VB Community Annotation] |
| AAEL010379 | | -1.0707 | 3.3989 | 0.0726 | 0.9996 | ATP-binding cassette transporter [Source:VB Community Annotation] |
| AAEL000425 | | 0.7945 | 2.4527 | 0.0726 | 0.9996 | |
| AAEL003434 | | 2.9446 | -0.0425 | 0.0727 | 0.9996 | alpha-amylase [Source:VB Community Annotation] |
| AAEL019679 | NA | 1.1339 | 2.9863 | 0.0729 | 0.9996 | NA |
| AAEL027425 | NA | 1.4627 | -0.6993 | 0.0732 | 0.9996 | NA |
| AAEL006734 | | 0.5423 | 3.5551 | 0.0734 | 0.9996 | cytochrome oxidase biogenesis protein (oxa1 mitochondrial) [Source:VB Community Annotation] |
| AAEL009679 | | 0.8987 | 3.4792 | 0.0735 | 0.9996 | secretory Phospholipase A2, putative [Source:VB Community Annotation] |
| AAEL007291 | | 1.3807 | 3.4507 | 0.0736 | 0.9996 | replication factor A, 14kD-subunit, putative [Source:VB Community Annotation] |
| AAEL006020 | | 0.5978 | 3.7503 | 0.0737 | 0.9996 | |
| AAEL010627 | | -1.2894 | 1.2652 | 0.0738 | 0.9996 | |
| AAEL020987 | NA | -0.6294 | 4.5435 | 0.0738 | 0.9996 | NA |
| AAEL001745 | | 1.8921 | 0.6065 | 0.0740 | 0.9996 | candidate tumor suppressor protein [Source:VB Community Annotation] |
| AAEL001183 | | -0.3750 | 5.4221 | 0.0740 | 0.9996 | zinc binding dehydrogenase [Source:VB Community Annotation] |
| AAEL007875 | | -2.0361 | -1.3694 | 0.0741 | 0.9996 | |
| AAEL010924 | | 0.6837 | 3.7945 | 0.0742 | 0.9996 | ca-activated cl channel protein [Source:VB Community Annotation] |
| AAEL001687 | | 0.8874 | 2.1203 | 0.0743 | 0.9996 | adenylate cyclase [Source:VB Community Annotation] |
| AAEL010076 | | 0.7486 | 3.3972 | 0.0743 | 0.9996 | |
| AAEL023393 | NA | 1.0910 | 3.0304 | 0.0744 | 0.9996 | NA |
| AAEL018051 | | 0.7952 | 3.7992 | 0.0745 | 0.9996 | |
| AAEL027044 | NA | -1.1035 | 1.8073 | 0.0746 | 0.9996 | NA |
| AAEL021869 | NA | 2.2400 | -1.2960 | 0.0747 | 0.9996 | NA |
| AAEL005239 | | 0.6181 | 2.4502 | 0.0750 | 0.9996 | gh regulated tbc protein-1 [Source:VB Community Annotation] |
| AAEL009890 | | 0.5089 | 4.4092 | 0.0751 | 0.9996 | |
| AAEL007959 | | 0.7058 | 4.6062 | 0.0754 | 0.9996 | |
| AAEL002079 | | 0.8678 | 1.8491 | 0.0754 | 0.9996 | TATA binding protein, putative [Source:VB Community Annotation] |
| AAEL025541 | NA | -1.9219 | -0.9141 | 0.0755 | 0.9996 | NA |
| AAEL026434 | NA | -1.9158 | 0.0625 | 0.0755 | 0.9996 | NA |
| AAEL007288 | | 0.7044 | 6.4252 | 0.0758 | 0.9996 | dynamin [Source:VB Community Annotation] |
| AAEL022248 | NA | 2.5626 | -0.9628 | 0.0758 | 0.9996 | NA |
| AAEL012745 | | 0.6858 | 2.7095 | 0.0758 | 0.9996 | |
| AAEL005363 | | 0.6586 | 3.1173 | 0.0764 | 0.9996 | galactosyltransferase [Source:VB Community Annotation] |
| AAEL009723 | | -0.6411 | 3.9451 | 0.0765 | 0.9996 | fibrinogen and fibronectin [Source:VB Community Annotation] |
| AAEL004583 | | -0.5022 | 6.3620 | 0.0765 | 0.9996 | |

| | | | | | | |
|---|---|---|---|---|---|---|
| AAEL009615 | GPROP8 | 0.8838 | 10.0982 | 0.0772 | 0.9996 | ultraviolet wavelength sensitive opsin [Source:VB Community Annotation] |
| AAEL009253 | | -0.6215 | 3.7816 | 0.0776 | 0.9996 | |
| AAEL020957 | NA | 1.0336 | 1.6983 | 0.0777 | 0.9996 | NA |
| AAEL019540 | NA | -0.9135 | 0.9071 | 0.0777 | 0.9996 | NA |
| AAEL028015 | NA | 0.5191 | 6.5682 | 0.0778 | 0.9996 | NA |
| AAEL008020 | | 0.5528 | 5.6886 | 0.0779 | 0.9996 | sorting nexin [Source:VB Community Annotation] |
| AAEL023006 | NA | -1.9883 | 1.3108 | 0.0786 | 0.9996 | NA |
| AAEL008651 | | -0.7374 | 6.2315 | 0.0788 | 0.9996 | |
| AAEL019725 | NA | -0.7931 | 6.3429 | 0.0789 | 0.9996 | NA |
| AAEL013750 | | -1.3755 | 0.8393 | 0.0789 | 0.9996 | |
| AAEL010847 | | 0.6071 | 2.9650 | 0.0789 | 0.9996 | zinc finger protein [Source:VB Community Annotation] |
| AAEL011830 | | 0.7236 | 5.9162 | 0.0791 | 0.9996 | Probable methylthioribulose-1-phosphate dehydratase (MTRu-1-P dehydratase)(EC 4.2.1.109) [Source:VB Community Annotation] |
| AAEL019628 | NA | -1.6794 | 0.7193 | 0.0791 | 0.9996 | NA |
| AAEL019576 | NA | -0.5071 | 4.8606 | 0.0793 | 0.9996 | NA |
| AAEL020739 | NA | 0.5206 | 6.6466 | 0.0793 | 0.9996 | NA |
| AAEL001513 | | 1.0111 | 1.5277 | 0.0794 | 0.9996 | WD-repeat protein [Source:VB Community Annotation] |
| AAEL005047 | | 0.8158 | 2.0879 | 0.0803 | 0.9996 | |
| AAEL006646 | | 0.4857 | 3.4750 | 0.0805 | 0.9996 | |
| AAEL011292 | | -1.1006 | 4.6278 | 0.0807 | 0.9996 | protease m1 zinc metalloprotease [Source:VB Community Annotation] |
| AAEL017139 | | -1.0692 | 4.7237 | 0.0808 | 0.9996 | |
| AAEL011567 | | 0.7086 | 4.4184 | 0.0808 | 0.9996 | protein phosphatase 2c [Source:VB Community Annotation] |
| AAEL012419 | | 0.4710 | 5.0388 | 0.0809 | 0.9996 | 26S proteasome subunit S9 [Source:VB Community Annotation] |
| AAEL020499 | NA | -1.3066 | 1.1924 | 0.0811 | 0.9996 | NA |
| AAEL025682 | NA | 1.7463 | -0.8743 | 0.0812 | 0.9996 | NA |
| AAEL015052 | | 0.4841 | 3.9125 | 0.0813 | 0.9996 | |
| AAEL000933 | | -0.4540 | 4.7503 | 0.0814 | 0.9996 | |
| AAEL012384 | | 0.7420 | 4.9246 | 0.0816 | 0.9996 | amalgam protein, putative [Source:VB Community Annotation] |
| AAEL024438 | NA | -0.7243 | 4.7338 | 0.0819 | 0.9996 | NA |
| AAEL004519 | | 0.8893 | 1.8779 | 0.0821 | 0.9996 | |
| AAEL004803 | | 0.8698 | 3.6469 | 0.0821 | 0.9996 | |
| AAEL005103 | | 0.5587 | 3.2957 | 0.0821 | 0.9996 | |
| AAEL012825 | | 1.3272 | 5.4479 | 0.0822 | 0.9996 | bifunctional purine biosynthesis protein [Source:VB Community Annotation] |
| AAEL008547 | | 0.8813 | 1.5078 | 0.0823 | 0.9996 | |
| AAEL013210 | | 0.6747 | 3.6165 | 0.0823 | 0.9996 | |
| AAEL007481 | | 0.8698 | 3.3985 | 0.0823 | 0.9996 | GDP-fucose transporter, putative [Source:VB Community Annotation] |
| AAEL023060 | NA | -2.2459 | -0.9007 | 0.0824 | 0.9996 | NA |
| AAEL011117 | | -0.6662 | 5.2498 | 0.0824 | 0.9996 | Histone deacetylase [Source:UniProtKB/TrEMBL;Acc:Q16QZ8] |
| AAEL028004 | NA | 0.4781 | 5.0445 | 0.0827 | 0.9996 | NA |
| AAEL026741 | NA | 0.8260 | 7.4484 | 0.0829 | 0.9996 | NA |
| AAEL014655 | | -1.2477 | -0.0677 | 0.0830 | 0.9996 | |

| | | | | | | |
|---|---|---|---|---|---|---|
| AAEL000821 | OBP6 | -1.6295 | -0.0714 | 0.0831 | 0.9996 | odorant binding protein OBP6 [Source:VB Community Annotation] |
| AAEL024508 | NA | 0.4392 | 5.2563 | 0.0831 | 0.9996 | NA |
| AAEL012546 | | 1.3442 | 1.0149 | 0.0832 | 0.9996 | DNA replication licensing factor MCM6 [Source:VB Community Annotation] |
| AAEL028073 | NA | 1.3063 | 0.7111 | 0.0834 | 0.9996 | NA |
| AAEL014699 | | 0.4171 | 5.2660 | 0.0835 | 0.9996 | ABC transporter [Source:VB Community Annotation] |
| AAEL000226 | | -0.6257 | 4.0196 | 0.0839 | 0.9996 | |
| AAEL027424 | NA | -0.5137 | 3.8699 | 0.0840 | 0.9996 | NA |
| AAEL002261 | | -1.0800 | 5.2103 | 0.0841 | 0.9996 | GTP cyclohydrolase i [Source:VB Community Annotation] |
| AAEL022657 | NA | -1.0224 | 2.5815 | 0.0845 | 0.9996 | NA |
| AAEL000883 | | 0.5921 | 4.5750 | 0.0845 | 0.9996 | |
| AAEL005776 | Orco | 0.9118 | 5.9231 | 0.0850 | 0.9996 | odorant receptor obligate co-receptor (Orco) [Source:VB Community Annotation] |
| AAEL013121 | | 0.7229 | 5.4852 | 0.0851 | 0.9996 | zeta-coat protein [Source:VB Community Annotation] |
| AAEL014657 | | -0.7328 | 5.2248 | 0.0856 | 0.9996 | |
| AAEL011469 | | 0.9235 | 5.0771 | 0.0860 | 0.9996 | |
| AAEL002554 | | 1.1198 | 1.2248 | 0.0861 | 0.9996 | anosmin, putative [Source:VB Community Annotation] |
| AAEL013179 | | -0.7565 | 1.9213 | 0.0862 | 0.9996 | 8-oxoguanine DNA glycosylase [Source:VB Community Annotation] |
| AAEL006238 | | 1.9286 | -0.5097 | 0.0862 | 0.9996 | |
| AAEL009154 | | 0.5899 | 3.2867 | 0.0862 | 0.9996 | Glutathione synthetase [Source:UniProtKB/TrEMBL;Acc:Q0IEN7] |
| AAEL011952 | | -0.7366 | 3.9596 | 0.0863 | 0.9996 | kelch repeat protein [Source:VB Community Annotation] |
| AAEL009211 | | 1.1796 | 1.1484 | 0.0863 | 0.9996 | |
| AAEL021113 | NA | 0.6630 | 4.7959 | 0.0864 | 0.9996 | NA |
| AAEL025600 | NA | -0.6980 | 3.3845 | 0.0865 | 0.9996 | NA |
| AAEL025367 | NA | -1.7097 | 6.2616 | 0.0865 | 0.9996 | NA |
| AAEL008863 | | 2.0313 | -0.3473 | 0.0869 | 0.9996 | protein regulator of cytokinesis 1 prc1 [Source:VB Community Annotation] |
| AAEL024740 | NA | 1.4607 | 1.7747 | 0.0870 | 0.9996 | NA |
| AAEL008714 | | 0.6576 | 4.7537 | 0.0874 | 0.9996 | |
| AAEL009646 | | -0.3942 | 6.6750 | 0.0874 | 0.9996 | |
| AAEL012974 | | 1.8584 | 4.7669 | 0.0875 | 0.9996 | glycine cleavage system h protein [Source:VB Community Annotation] |
| AAEL013682 | | 0.8099 | 2.2933 | 0.0877 | 0.9996 | zinc finger protein [Source:VB Community Annotation] |
| AAEL005134 | | -1.2128 | 0.3933 | 0.0877 | 0.9996 | arylsulfatase b [Source:VB Community Annotation] |
| AAEL007261 | | 1.0637 | 3.8976 | 0.0877 | 0.9996 | |
| AAEL021387 | NA | -1.8810 | -1.4186 | 0.0881 | 0.9996 | NA |
| AAEL022916 | NA | 1.2943 | 0.7404 | 0.0882 | 0.9996 | NA |
| AAEL015455 | | 1.5119 | 1.7521 | 0.0883 | 0.9996 | |
| AAEL008911 | | 1.0752 | 2.9768 | 0.0883 | 0.9996 | |
| AAEL024446 | NA | 1.6885 | -0.2434 | 0.0884 | 0.9996 | NA |
| AAEL023847 | NA | -1.0126 | 1.1754 | 0.0885 | 0.9996 | NA |
| AAEL012341 | | -0.8655 | 5.3594 | 0.0885 | 0.9996 | lysosomal acid lipase, putative [Source:VB Community Annotation] |
| AAEL027860 | NA | -0.5646 | 7.2272 | 0.0886 | 0.9996 | NA |
| AAEL012839 | | 1.3053 | 0.5895 | 0.0887 | 0.9996 | |

| | | | | | | |
|---|---|---|---|---|---|---|
| AAEL006036 | | 1.4163 | 2.8552 | 0.0889 | 0.9996 | phosphatase SSU72, putative [Source:VB Community Annotation] |
| AAEL004308 | | -0.3770 | 5.3972 | 0.0890 | 0.9996 | Proteasome subunit alpha type [Source:UniProtKB/TrEMBL;Acc:Q1HQN1] |
| AAEL009918 | | 0.9400 | 2.1892 | 0.0891 | 0.9996 | |
| AAEL011415 | | 0.7106 | 3.1861 | 0.0892 | 0.9996 | lysine-specific histone demethylase [Source:VB Community Annotation] |
| AAEL010219 | | -0.7173 | 6.8383 | 0.0892 | 0.9996 | sugar transporter [Source:VB Community Annotation] |
| AAEL009153 | | 1.2236 | 1.8984 | 0.0892 | 0.9996 | M-type 9 protein, putative [Source:VB Community Annotation] |
| AAEL026300 | NA | -1.8699 | 3.9289 | 0.0892 | 0.9996 | NA |
| AAEL011394 | | 0.5246 | 5.1163 | 0.0893 | 0.9996 | |
| AAEL026160 | NA | 0.5042 | 3.8869 | 0.0895 | 0.9996 | NA |
| AAEL019669 | NA | -0.6901 | 8.6416 | 0.0895 | 0.9996 | NA |
| AAEL010931 | | -1.1052 | 3.2385 | 0.0896 | 0.9996 | |
| AAEL025213 | NA | 1.7096 | 0.2196 | 0.0897 | 0.9996 | NA |
| AAEL004625 | | 0.5797 | 2.5268 | 0.0900 | 0.9996 | |
| AAEL022163 | NA | 1.0117 | 0.7957 | 0.0900 | 0.9996 | NA |
| AAEL013905 | | 0.8387 | 3.7564 | 0.0901 | 0.9996 | |
| AAEL009479 | | -0.5879 | 4.4967 | 0.0901 | 0.9996 | amino acid transporter [Source:VB Community Annotation] |
| AAEL019487 | NA | 0.5078 | 6.2667 | 0.0901 | 0.9996 | NA |
| AAEL004973 | | 0.6218 | 7.0999 | 0.0906 | 0.9996 | golgi phosphoprotein 3 (coat-protein GPP34) [Source:VB Community Annotation] |
| AAEL013472 | | -0.4727 | 4.5501 | 0.0907 | 0.9996 | |
| AAEL007065 | Arf1 | -0.5155 | 9.1512 | 0.0910 | 0.9996 | ADP-ribosylation factor 1 [Source:VB Community Annotation] |
| AAEL008493 | | 0.9166 | 2.7373 | 0.0910 | 0.9996 | zinc finger protein [Source:VB Community Annotation] |
| AAEL011536 | | 1.0457 | 3.0875 | 0.0911 | 0.9996 | phosphoglucomutase [Source:VB Community Annotation] |
| AAEL003704 | mRpL13 | -0.4356 | 5.6999 | 0.0911 | 0.9996 | 39S ribosomal protein L13, mitochondrial [Source:VB Community Annotation] |
| AAEL025742 | NA | -1.7481 | -0.1936 | 0.0913 | 0.9996 | NA |
| AAEL010102 | | 0.5806 | 5.8080 | 0.0913 | 0.9996 | tetraspanin, putative [Source:VB Community Annotation] |
| AAEL009085 | | -0.4663 | 6.4246 | 0.0920 | 0.9996 | rho/rac/cdc GTPase-activating protein [Source:VB Community Annotation] |
| AAEL015356 | | 0.7149 | 2.4769 | 0.0921 | 0.9996 | |
| AAEL005813 | | 0.8205 | 2.1410 | 0.0923 | 0.9996 | |
| AAEL002124 | CLIPD6 | -0.8732 | 8.2276 | 0.0925 | 0.9996 | Clip-Domain Serine Protease  family D [Source:VB Community Annotation] |
| AAEL010800 | | 0.6828 | 3.7235 | 0.0925 | 0.9996 | |
| AAEL002655 | | -1.4943 | 3.8890 | 0.0932 | 0.9996 | matrix metalloproteinase [Source:VB Community Annotation] |
| AAEL022593 | NA | -1.4528 | 0.4909 | 0.0932 | 0.9996 | NA |
| AAEL028021 | NA | -0.5225 | 8.2603 | 0.0933 | 0.9996 | NA |
| AAEL004691 | | -0.6478 | 7.0841 | 0.0937 | 0.9996 | ring finger [Source:VB Community Annotation] |
| AAEL007667 | | -1.8317 | 2.7202 | 0.0938 | 0.9996 | |
| AAEL004825 | | -0.5406 | 3.6820 | 0.0938 | 0.9996 | potassium-dependent sodium-calcium exchanger, putative [Source:VB Community Annotation] |
| AAEL027626 | NA | -0.5570 | 4.3619 | 0.0939 | 0.9996 | NA |
| AAEL021035 | NA | 0.7476 | 4.9507 | 0.0939 | 0.9996 | NA |
| AAEL026664 | NA | -0.5359 | 4.2433 | 0.0940 | 0.9996 | NA |
| AAEL003618 | | 1.2456 | 2.2513 | 0.0941 | 0.9996 | sodium/chloride dependent amino acid transporter [Source:VB Community Annotation] |

| | | | | | | |
|---|---|---|---|---|---|---|
| AAEL008497 | | 0.8694 | 3.9634 | 0.0942 | 0.9996 | |
| AAEL010864 | | 0.4811 | 4.4556 | 0.0945 | 0.9996 | TRP-phosphatase, putative [Source:VB Community Annotation] |
| AAEL005968 | | 0.6625 | 6.1464 | 0.0945 | 0.9996 | Ubiquitin-fold modifier-conjugating enzyme 1 (Ufm1-conjugating enzyme 1) [Source:VB Community Annotation] |
| AAEL011934 | GSTZ1 | 0.4882 | 5.1867 | 0.0946 | 0.9996 | glutathione transferase [Source:VB Community Annotation] |
| AAEL007910 | | 0.5504 | 3.3405 | 0.0950 | 0.9996 | |
| AAEL002254 | | -0.7647 | 6.7378 | 0.0951 | 0.9996 | |
| AAEL006051 | | -0.6882 | 5.2222 | 0.0951 | 0.9996 | beta-sarcoglycan [Source:VB Community Annotation] |
| AAEL020800 | NA | 0.5880 | 5.3519 | 0.0953 | 0.9996 | NA |
| AAEL027356 | NA | -1.5684 | 0.5014 | 0.0955 | 0.9996 | NA |
| AAEL024264 | NA | 2.5798 | 2.4424 | 0.0955 | 0.9996 | NA |
| AAEL013206 | | 0.5091 | 6.2076 | 0.0959 | 0.9996 | fgf receptor activating protein [Source:VB Community Annotation] |
| AAEL009372 | | 1.0978 | 4.0118 | 0.0959 | 0.9996 | |
| AAEL027099 | NA | -0.4802 | 5.4880 | 0.0960 | 0.9996 | NA |
| AAEL022411 | NA | 1.2656 | 0.0225 | 0.0961 | 0.9996 | NA |
| AAEL003316 | | -0.6041 | 7.1511 | 0.0962 | 0.9996 | |
| AAEL020012 | NA | 0.6052 | 2.6186 | 0.0964 | 0.9996 | NA |
| AAEL000011 | | -1.0666 | -0.2202 | 0.0964 | 0.9996 | |
| AAEL007869 | | 0.5896 | 3.2479 | 0.0969 | 0.9996 | |
| AAEL006488 | | 1.1099 | 1.7716 | 0.0970 | 0.9996 | nucleoporin, p88, putative [Source:VB Community Annotation] |
| AAEL003996 | | 1.0305 | 1.3648 | 0.0971 | 0.9996 | |
| AAEL008505 | | 1.2069 | 1.1580 | 0.0971 | 0.9996 | |
| AAEL003883 | | -0.5250 | 4.7023 | 0.0972 | 0.9996 | |
| AAEL010173 | | 1.0267 | 3.1950 | 0.0974 | 0.9996 | |
| AAEL009483 | | -1.4740 | 3.0278 | 0.0974 | 0.9996 | |
| AAEL020835 | NA | 2.0426 | -1.2734 | 0.0976 | 0.9996 | NA |
| AAEL019818 | NA | 1.1830 | 2.3088 | 0.0977 | 0.9996 | NA |
| AAEL002896 | | 0.6905 | 2.9618 | 0.0978 | 0.9996 | |
| AAEL009690 | | 1.2786 | 0.5253 | 0.0983 | 0.9996 | |
| AAEL003476 | | -0.7828 | 2.3489 | 0.0984 | 0.9996 | calpain-c [Source:VB Community Annotation] |
| AAEL008014 | | -0.5635 | 2.9732 | 0.0984 | 0.9996 | ef hand protein [Source:VB Community Annotation] |
| AAEL013397 | CycC | -0.7005 | 3.8759 | 0.0986 | 0.9996 | cyclin C [Source:VB Community Annotation] |
| AAEL005725 | | 0.5195 | 3.8524 | 0.0986 | 0.9996 | |
| AAEL002031 | CYP12F7 | 0.9568 | 6.3720 | 0.0987 | 0.9996 | cytochrome P450 [Source:VB Community Annotation] |
| AAEL013078 | | 0.6569 | 6.5511 | 0.0989 | 0.9996 | glycosyltransferase [Source:VB Community Annotation] |
| AAEL004414 | | 0.9493 | 4.1825 | 0.0994 | 0.9996 | |
| AAEL013176 | | 0.7951 | 4.1036 | 0.0994 | 0.9996 | |
| AAEL011710 | | 0.5999 | 4.1830 | 0.0995 | 0.9996 | |
| AAEL010332 | | 0.6548 | 1.9647 | 0.0996 | 0.9996 | |
| AAEL012874 | | 0.9221 | 2.0263 | 0.0996 | 0.9996 | rrp4 [Source:VB Community Annotation] |
| AAEL022149 | NA | 0.7566 | 4.1221 | 0.0996 | 0.9996 | NA |

| | | | | | | |
|---|---|---|---|---|---|---|
| AAEL001569 | | -0.4803 | 7.2385 | 0.0999 | 0.9996 | |
| AAEL007669 | | -1.7496 | 1.8264 | 0.0999 | 0.9996 | oxidoreductase [Source:VB Community Annotation] |
| AAEL012875 | | 0.4939 | 5.2517 | 0.1001 | 0.9996 | snare protein sec22 [Source:VB Community Annotation] |
| AAEL004325 | RpL5 | 0.4505 | 10.4258 | 0.1001 | 0.9996 | 60S ribosomal protein L5 [Source:UniProtKB/Swiss-Prot;Acc:Q1HQU2] |
| AAEL016999 | white | 1.0468 | 4.3403 | 0.1002 | 0.9996 | white protein; eye pigment transporter [Source:VB Community Annotation] |
| AAEL020720 | NA | -0.7071 | 3.4336 | 0.1002 | 0.9996 | NA |
| AAEL006176 | OBP27 | 1.1670 | 8.7007 | 0.1003 | 0.9996 | odorant binding protein OBP27 [Source:VB Community Annotation] |
| AAEL011083 | | 0.6272 | 4.0133 | 0.1004 | 0.9996 | |
| AAEL011463 | | 0.9405 | 1.7240 | 0.1004 | 0.9996 | cytochrome P450 [Source:VB Community Annotation] |
| AAEL013220 | | -0.8166 | 5.3078 | 0.1005 | 0.9996 | |
| AAEL020828 | NA | -1.8913 | -1.1948 | 0.1006 | 0.9996 | NA |
| AAEL001799 | | 0.4729 | 5.7287 | 0.1010 | 0.9996 | |
| AAEL013071 | | 0.4557 | 6.9906 | 0.1012 | 0.9996 | ribophorin [Source:VB Community Annotation] |
| AAEL012140 | | 0.5617 | 3.1736 | 0.1012 | 0.9996 | tRNA-dihydrouridine synthase [Source:VB Community Annotation] |
| AAEL001492 | | 0.5640 | 3.0675 | 0.1013 | 0.9996 | cgmp-dependent 3,5-cyclic phosphodiesterase [Source:VB Community Annotation] |
| AAEL014934 | | 1.6975 | 0.3194 | 0.1015 | 0.9996 | |
| AAEL011204 | | 0.6583 | 3.2794 | 0.1016 | 0.9996 | xenotropic and polytropic murine leukemia virus receptor xpr1 [Source:VB Community Annotation] |
| AAEL028086 | NA | 1.1241 | 0.5719 | 0.1018 | 0.9996 | NA |
| AAEL023230 | NA | 0.8435 | 3.0394 | 0.1019 | 0.9996 | NA |
| AAEL011216 | | -0.6206 | 5.8010 | 0.1028 | 0.9996 | |
| AAEL006864 | | 0.6971 | 2.9537 | 0.1030 | 0.9996 | |
| AAEL020284 | NA | -1.4651 | 1.1871 | 0.1032 | 0.9996 | NA |
| AAEL024410 | NA | -0.4344 | 5.1623 | 0.1032 | 0.9996 | NA |
| AAEL009307 | | 0.4793 | 4.8878 | 0.1033 | 0.9996 | |
| AAEL013394 | | -0.3713 | 5.8110 | 0.1036 | 0.9996 | |
| AAEL014022 | | -1.2069 | 4.9334 | 0.1036 | 0.9996 | |
| AAEL003371 | | -0.4532 | 6.1048 | 0.1037 | 0.9996 | F-box and WD-40 domain protein [Source:VB Community Annotation] |
| AAEL017082 | | 0.5242 | 6.5348 | 0.1039 | 0.9996 | |
| AAEL020342 | NA | -1.3826 | 5.9974 | 0.1042 | 0.9996 | NA |
| AAEL004472 | | -0.4502 | 7.7559 | 0.1042 | 0.9996 | |
| AAEL003763 | CYP329B1 | 0.5652 | 3.3400 | 0.1045 | 0.9996 | cytochrome P450 [Source:VB Community Annotation] |
| AAEL010136 | | 0.5433 | 3.2440 | 0.1048 | 0.9996 | |
| AAEL009333 | | -0.7191 | 4.4112 | 0.1050 | 0.9996 | |
| AAEL025723 | NA | -1.9735 | -1.3348 | 0.1050 | 0.9996 | NA |
| AAEL022726 | NA | -0.6494 | 5.2110 | 0.1053 | 0.9996 | NA |
| AAEL005465 | | 1.7193 | 0.1424 | 0.1053 | 0.9996 | |
| AAEL009362 | | -0.9665 | 3.2088 | 0.1053 | 0.9996 | cationic amino acid transporter [Source:VB Community Annotation] |
| AAEL024516 | NA | 1.6376 | 0.6483 | 0.1055 | 0.9996 | NA |
| AAEL005678 | | 0.4163 | 5.5286 | 0.1055 | 0.9996 | UDP-galactose transporter [Source:VB Community Annotation] |
| AAEL000874 | ARK | 0.9563 | 1.9945 | 0.1057 | 0.9996 | Apaf-1 Related Killer [Source:VB Community Annotation] |

| | | | | | | |
|---|---|---|---|---|---|---|
| AAEL014609 | CYP9J26 | -0.7651 | 5.5798 | 0.1059 | 0.9996 | cytochrome P450 [Source:VB Community Annotation] |
| AAEL010455 | | 0.5093 | 4.3033 | 0.1060 | 0.9996 | cxyorf1 [Source:VB Community Annotation] |
| AAEL009636 | | -0.4827 | 4.7839 | 0.1061 | 0.9996 | |
| AAEL009058 | | 1.2635 | 0.8544 | 0.1061 | 0.9996 | |
| AAEL015271 | | 1.8758 | 3.6250 | 0.1065 | 0.9996 | rac GTPase [Source:VB Community Annotation] |
| AAEL003238 | | 0.4648 | 4.5110 | 0.1068 | 0.9996 | Palmitoyltransferase [Source:UniProtKB/TrEMBL;Acc:Q17FV8] |
| AAEL013491 | | -0.4962 | 6.5020 | 0.1069 | 0.9996 | short-chain dehydrogenase [Source:VB Community Annotation] |
| AAEL018286 | | 0.4269 | 4.6142 | 0.1072 | 0.9996 | |
| AAEL022074 | NA | 0.8438 | 1.3887 | 0.1073 | 0.9996 | NA |
| AAEL001881 | | 0.7480 | 1.6347 | 0.1073 | 0.9996 | |
| AAEL014349 | CLIPB15 | -0.9112 | 4.4143 | 0.1074 | 0.9996 | Clip-Domain Serine Protease  family B. [Source:VB Community Annotation] |
| AAEL006786 | | 0.4324 | 4.6817 | 0.1077 | 0.9996 | GTPase_rho [Source:VB Community Annotation] |
| AAEL011009 | | -1.0448 | 1.7887 | 0.1077 | 0.9996 | fibrinogen and fibronectin [Source:VB Community Annotation] |
| AAEL002867 | | 0.5666 | 5.8358 | 0.1080 | 0.9996 | phenylalanyl-tRNA synthetase alpha chain [Source:VB Community Annotation] |
| AAEL000293 | | -0.7556 | 5.3988 | 0.1082 | 0.9996 | ebna2 binding protein P100 [Source:VB Community Annotation] |
| AAEL022387 | NA | 2.4430 | 1.0381 | 0.1082 | 0.9996 | NA |
| AAEL011809 | | -0.7797 | 2.8490 | 0.1084 | 0.9996 | glucose dehydrogenase [Source:VB Community Annotation] |
| AAEL004532 | | 2.0526 | 3.5008 | 0.1086 | 0.9996 | glyoxylate/hydroxypyruvate reductase [Source:VB Community Annotation] |
| AAEL020634 | NA | -0.4532 | 6.6842 | 0.1086 | 0.9996 | NA |
| AAEL002972 | | -0.6793 | 4.7493 | 0.1087 | 0.9996 | brain chitinase and chia [Source:VB Community Annotation] |
| AAEL015066 | | -0.6784 | 4.5872 | 0.1087 | 0.9996 | nebula [Source:VB Community Annotation] |
| AAEL006610 | | 0.9648 | 3.2476 | 0.1089 | 0.9996 | |
| AAEL001939 | | 0.9455 | 3.4144 | 0.1090 | 0.9996 | |
| AAEL002372 | RpS11 | -0.5219 | 11.3715 | 0.1092 | 0.9996 | 40S ribosomal protein S11 [Source:VB Community Annotation] |
| AAEL012501 | | 0.4922 | 3.2042 | 0.1093 | 0.9996 | |
| AAEL010610 | | 0.5309 | 3.5242 | 0.1095 | 0.9996 | serine palmitoyltransferase i [Source:VB Community Annotation] |
| AAEL005254 | | 0.6198 | 3.6757 | 0.1095 | 0.9996 | dynactin P62 subunit [Source:VB Community Annotation] |
| AAEL014836 | mRpS15 | 0.9019 | 3.7697 | 0.1098 | 0.9996 | 28S ribosomal protein S15, mitochondrial precursor [Source:VB Community Annotation] |
| AAEL017145 | | -2.0478 | 1.6541 | 0.1099 | 0.9996 | |
| AAEL019840 | NA | 0.7671 | 1.6138 | 0.1099 | 0.9996 | NA |
| AAEL020968 | NA | 0.7820 | 2.3268 | 0.1100 | 0.9996 | NA |
| AAEL025455 | NA | 0.6006 | 5.0059 | 0.1100 | 0.9996 | NA |
| AAEL006420 | | -1.2751 | 3.4323 | 0.1101 | 0.9996 | |
| AAEL011285 | | 0.7572 | 2.3804 | 0.1101 | 0.9996 | Zinc finger Ran-binding domain-containing protein 2 [Source:UniProtKB/TrEMBL;Acc:Q16QI4] |
| AAEL002250 | | 0.7625 | 2.7358 | 0.1101 | 0.9996 | terminal deoxycytidyl transferase rev1 [Source:VB Community Annotation] |
| AAEL007275 | | 0.8887 | 3.3429 | 0.1103 | 0.9996 | aldo-keto reductase [Source:VB Community Annotation] |
| AAEL010159 | | 0.5571 | 7.2272 | 0.1105 | 0.9996 | importin beta-3 [Source:VB Community Annotation] |
| AAEL002988 | | 0.6523 | 4.3617 | 0.1109 | 0.9996 | |
| AAEL017481 | | -0.4854 | 8.4689 | 0.1109 | 0.9996 | |
| AAEL024038 | NA | 0.7317 | 2.4686 | 0.1110 | 0.9996 | NA |

| | | | | | | |
|---|---|---|---|---|---|---|
| AAEL002998 | | 0.6126 | 3.6281 | 0.1110 | 0.9996 | |
| AAEL005445 | | -1.5005 | 2.4587 | 0.1113 | 0.9996 | |
| AAEL010260 | | -0.7821 | 8.2639 | 0.1113 | 0.9996 | |
| AAEL004261 | | 1.0051 | 1.1923 | 0.1118 | 0.9996 | |
| AAEL010585 | | -1.4232 | 0.2561 | 0.1119 | 0.9996 | spermatogenesis associated factor [Source:VB Community Annotation] |
| AAEL006532 | | -1.7013 | -0.3788 | 0.1120 | 0.9996 | histone transcription regulator [Source:VB Community Annotation] |
| AAEL012217 | | -0.7645 | 4.7986 | 0.1122 | 0.9996 | protease m1 zinc metalloprotease [Source:VB Community Annotation] |
| AAEL015543 | | 0.4963 | 5.0232 | 0.1122 | 0.9996 | |
| AAEL000925 | | 1.3258 | 3.1824 | 0.1125 | 0.9996 | leucine-zipper-like transcriptional regulator 1 (LZTR-1) [Source:VB Community Annotation] |
| AAEL023815 | NA | -1.1474 | 0.7711 | 0.1126 | 0.9996 | NA |
| AAEL006835 | | 0.9503 | 3.8618 | 0.1126 | 0.9996 | transient receptor potential cation channel protein painless [Source:VB Community Annotation] |
| AAEL019937 | NA | -0.3137 | 8.8995 | 0.1128 | 0.9996 | NA |
| AAEL011408 | CTL21 | -1.2213 | 5.3519 | 0.1128 | 0.9996 | C-Type Lectin (CTL21) [Source:VB Community Annotation] |
| AAEL019691 | NA | 0.7572 | 5.9836 | 0.1130 | 0.9996 | NA |
| AAEL000930 | | -0.4157 | 5.8643 | 0.1130 | 0.9996 | |
| AAEL010752 | | 1.1399 | 1.5753 | 0.1131 | 0.9996 | |
| AAEL018056 | | -0.4463 | 5.4689 | 0.1134 | 0.9996 | |
| AAEL005076 | | -0.4493 | 5.3502 | 0.1141 | 0.9996 | alanyl aminopeptidase [Source:VB Community Annotation] |
| AAEL003108 | | 0.4522 | 4.2440 | 0.1142 | 0.9996 | protein-tyrosine phosphatase, non-receptor type nt5 [Source:VB Community Annotation] |
| AAEL021953 | NA | 0.5345 | 3.5062 | 0.1147 | 0.9996 | NA |
| AAEL024317 | NA | 0.8801 | 1.9008 | 0.1147 | 0.9996 | NA |
| AAEL020922 | NA | 1.2753 | 1.4670 | 0.1151 | 0.9996 | NA |
| AAEL022687 | NA | 0.5463 | 2.9735 | 0.1154 | 0.9996 | NA |
| AAEL006407 | | -1.5506 | -0.1044 | 0.1155 | 0.9996 | |
| AAEL009123 | CYP6Z6 | 0.6881 | 5.1724 | 0.1156 | 0.9996 | cytochrome P450 [Source:VB Community Annotation] |
| AAEL004288 | | -0.5182 | 6.0186 | 0.1156 | 0.9996 | protein phsophatase-2a [Source:VB Community Annotation] |
| AAEL000026 | | -0.7341 | 3.3348 | 0.1158 | 0.9996 | dynein light chain, putative [Source:VB Community Annotation] |
| AAEL011890 | | -0.5233 | 8.4376 | 0.1159 | 0.9996 | |
| AAEL007309 | | 1.0395 | 1.8043 | 0.1159 | 0.9996 | |
| AAEL004546 | betaCop | 0.4431 | 5.8916 | 0.1162 | 0.9996 | coatomer beta subunit [Source:VB Community Annotation] |
| AAEL010552 | | -1.6087 | -0.6162 | 0.1162 | 0.9996 | |
| AAEL004920 | | -0.4938 | 4.4999 | 0.1162 | 0.9996 | |
| AAEL007473 | CYP6AH1 | -0.6825 | 7.4957 | 0.1163 | 0.9996 | cytochrome P450 [Source:VB Community Annotation] |
| AAEL007731 | | 0.8885 | 6.5485 | 0.1164 | 0.9996 | Pep12p, putative [Source:VB Community Annotation] |
| AAEL006253 | | 0.7218 | 5.0743 | 0.1164 | 0.9996 | |
| AAEL001087 | | 0.8273 | 5.2240 | 0.1165 | 0.9996 | synaptic vesicle protein [Source:VB Community Annotation] |
| AAEL004023 | | 0.4097 | 4.1514 | 0.1166 | 0.9996 | juvenile hormone-inducible protein, putative [Source:VB Community Annotation] |
| AAEL010900 | | -0.3703 | 6.4556 | 0.1169 | 0.9996 | importin alpha [Source:VB Community Annotation] |
| AAEL004790 | | 0.4981 | 4.2624 | 0.1169 | 0.9996 | ubiquitin-protein ligase [Source:VB Community Annotation] |
| AAEL004700 | | 0.6783 | 2.6218 | 0.1169 | 0.9996 | cdkl1/4 [Source:VB Community Annotation] |

| | | | | | | |
|---|---|---|---|---|---|---|
| AAEL007078 | eIF3-S10 | -1.1142 | 3.3746 | 0.1172 | 0.9996 | Eukaryotic translation initiation factor 3 subunit A (eIF3a)(Eukaryotic translation initiation factor 3 subunit 10) [Source:VB Community An |
| AAEL010402 | | 0.9921 | 3.7270 | 0.1173 | 0.9996 | DEAD box ATP-dependent RNA helicase [Source:VB Community Annotation] |
| AAEL013907 | | 2.0043 | 3.4971 | 0.1174 | 0.9996 | d-alanyl-d-alanine carboxypeptidase [Source:VB Community Annotation] |
| AAEL022213 | NA | 0.7653 | 4.6519 | 0.1174 | 0.9996 | NA |
| AAEL006984 | CYP6AG5 | -0.8077 | 3.5418 | 0.1174 | 0.9996 | cytochrome P450 [Source:VB Community Annotation] |
| AAEL024179 | NA | -0.6490 | 6.4956 | 0.1174 | 0.9996 | NA |
| AAEL013521 | | 0.4084 | 4.6480 | 0.1177 | 0.9996 | tryptophanyl-tRNA synthetase [Source:VB Community Annotation] |
| AAEL006660 | | 1.3307 | -0.0204 | 0.1177 | 0.9996 | DNA replication complex GINS protein PSF2 [Source:UniProtKB/TrEMBL;Acc:Q175H4] |
| AAEL024044 | NA | -1.1351 | 1.6813 | 0.1178 | 0.9996 | NA |
| AAEL004839 | | 0.5053 | 4.0691 | 0.1180 | 0.9996 | cyclin t [Source:VB Community Annotation] |
| AAEL009864 | | -0.6570 | 5.0306 | 0.1183 | 0.9996 | |
| AAEL023193 | NA | -0.6459 | 6.9793 | 0.1183 | 0.9996 | NA |
| AAEL002428 | | 0.4562 | 5.3851 | 0.1187 | 0.9996 | |
| AAEL011705 | | 0.6515 | 2.3549 | 0.1187 | 0.9996 | |
| AAEL022347 | NA | 1.8767 | -0.2408 | 0.1188 | 0.9996 | NA |
| AAEL006137 | SRPN19 | -1.2113 | 0.5756 | 0.1189 | 0.9996 | Serine Protease Inhibitor (serpin) homologue - unlikely to be inhibitory. [Source:VB Community Annotation] |
| AAEL002043 | CYP305A5 | -0.7488 | 2.6648 | 0.1190 | 0.9996 | cytochrome P450 [Source:VB Community Annotation] |
| AAEL004034 | mRpS6 | -0.6175 | 4.6674 | 0.1191 | 0.9996 | mitochondrial ribosomal protein, S6, putative [Source:VB Community Annotation] |
| AAEL003537 | | 0.4917 | 4.5314 | 0.1191 | 0.9996 | nuclear inhibitor of protein phosphatase-1 [Source:VB Community Annotation] |
| AAEL009027 | Med24 | 0.6631 | 2.8479 | 0.1192 | 0.9996 | Mediator of RNA polymerase II transcription subunit 24 (Med24) [Source:VB Community Annotation] |
| AAEL013796 | | 0.5582 | 3.7159 | 0.1192 | 0.9996 | |
| AAEL001507 | | 0.5182 | 3.7849 | 0.1193 | 0.9996 | |
| AAEL018310 | | 1.0762 | 3.2206 | 0.1195 | 0.9996 | |
| AAEL004434 | | 0.7946 | 7.0311 | 0.1196 | 0.9996 | transketolase [Source:VB Community Annotation] |
| AAEL012413 | | 0.4504 | 3.3566 | 0.1198 | 0.9996 | n-acetyltransferase mak3 [Source:VB Community Annotation] |
| AAEL023268 | NA | 2.1676 | 2.6785 | 0.1202 | 0.9996 | NA |
| AAEL005602 | | 2.1082 | -0.3622 | 0.1202 | 0.9996 | |
| AAEL008364 | SRPN9 | -0.6030 | 6.8689 | 0.1202 | 0.9996 | Serine Protease Inhibitor (serpin) likely cleavage at S/S. [Source:VB Community Annotation] |
| AAEL002905 | | 1.4540 | 3.2471 | 0.1203 | 0.9996 | |
| AAEL012249 | | -0.4719 | 4.9228 | 0.1205 | 0.9996 | |
| AAEL024576 | NA | -0.8982 | 2.3725 | 0.1205 | 0.9996 | NA |
| AAEL000102 | | 0.5415 | 6.8785 | 0.1207 | 0.9996 | |
| AAEL019768 | NA | 0.8602 | 5.5873 | 0.1208 | 0.9996 | NA |
| AAEL005023 | | 0.4334 | 5.3202 | 0.1208 | 0.9996 | Palmitoyltransferase [Source:UniProtKB/TrEMBL;Acc:Q17BB7] |
| AAEL008112 | | -0.6981 | 2.1795 | 0.1208 | 0.9996 | |
| AAEL021901 | NA | 1.3497 | 2.8274 | 0.1209 | 0.9996 | NA |
| AAEL014562 | RpL12 | 0.4824 | 10.4445 | 0.1209 | 0.9996 | 60S ribosomal protein L12 [Source:VB Community Annotation] |
| AAEL008465 | | -0.6788 | 2.8526 | 0.1212 | 0.9996 | gamma-tubulin complex component 3 (gcp-3) [Source:VB Community Annotation] |
| AAEL017229 | | 1.5925 | -0.7092 | 0.1212 | 0.9996 | |
| AAEL025045 | NA | -0.6413 | 4.5324 | 0.1212 | 0.9996 | NA |

| | | | | | | |
|---|---|---|---|---|---|---|
| AAEL028153 | NA | 1.5483 | 5.3574 | 0.1213 | 0.9996 | NA |
| AAEL002099 | | 1.7734 | -0.5958 | 0.1214 | 0.9996 | cuticle protein, putative [Source:VB Community Annotation] |
| AAEL003009 | | -0.3522 | 6.0613 | 0.1214 | 0.9996 | zinc finger protein [Source:VB Community Annotation] |
| AAEL024786 | NA | 0.6090 | 4.0062 | 0.1214 | 0.9996 | NA |
| AAEL023123 | NA | 1.8439 | -1.7673 | 0.1215 | 0.9996 | NA |
| AAEL020441 | NA | 0.9903 | 2.9674 | 0.1218 | 0.9996 | NA |
| AAEL003281 | | -0.9316 | 2.2382 | 0.1218 | 0.9996 | ADP-ribosylation factor, arf [Source:VB Community Annotation] |
| AAEL006299 | | 0.3892 | 6.0084 | 0.1220 | 0.9996 | methyltransferase, putative [Source:VB Community Annotation] |
| AAEL009750 | | -2.4221 | -2.3884 | 0.1223 | 0.9996 | |
| AAEL027700 | NA | 2.4981 | 1.0867 | 0.1223 | 0.9996 | NA |
| AAEL011733 | | 0.6416 | 4.9664 | 0.1224 | 0.9996 | transcription elongation regulator 1 (ca150) [Source:VB Community Annotation] |
| AAEL013571 | | 0.8928 | 6.1221 | 0.1227 | 0.9996 | synaptobrevin [Source:VB Community Annotation] |
| AAEL004630 | | -1.1650 | 1.6132 | 0.1227 | 0.9996 | |
| AAEL027749 | NA | -1.3663 | 1.6305 | 0.1229 | 0.9996 | NA |
| AAEL011981 | | -0.5771 | 6.9954 | 0.1229 | 0.9996 | glutamate decarboxylase [Source:VB Community Annotation] |
| AAEL001796 | | 0.9165 | 3.4627 | 0.1230 | 0.9996 | Nuclear hormone receptor (HR78) [Source:VB Community Annotation] |
| AAEL023242 | NA | -0.5373 | 6.4443 | 0.1232 | 0.9996 | NA |
| AAEL022486 | NA | 1.2322 | -0.3316 | 0.1232 | 0.9996 | NA |
| AAEL012826 | | 0.6359 | 2.7677 | 0.1234 | 0.9996 | replication factor a 1, rfa1 [Source:VB Community Annotation] |
| AAEL024010 | NA | -1.2635 | 0.4200 | 0.1234 | 0.9996 | NA |
| AAEL005957 | | -0.7510 | 2.0830 | 0.1235 | 0.9996 | phospholipase b, plb1 [Source:VB Community Annotation] |
| AAEL016973 | | -0.6368 | 6.0770 | 0.1237 | 0.9996 | |
| AAEL021140 | NA | -0.7463 | 7.4498 | 0.1240 | 0.9996 | NA |
| AAEL022169 | NA | -0.8278 | 2.5489 | 0.1240 | 0.9996 | NA |
| AAEL010949 | | 1.0242 | 3.3627 | 0.1242 | 0.9996 | |
| AAEL004668 | | 0.6811 | 4.3814 | 0.1242 | 0.9996 | septin [Source:VB Community Annotation] |
| AAEL009413 | | -0.5074 | 5.2425 | 0.1243 | 0.9996 | |
| AAEL005053 | | 0.6715 | 2.5983 | 0.1245 | 0.9996 | |
| AAEL016559 | tRNA-Glu | 1.4986 | 0.0327 | 0.1247 | 0.9996 | |
| AAEL001305 | | 0.9692 | 4.6735 | 0.1248 | 0.9996 | |
| AAEL020467 | NA | 0.8205 | 2.5112 | 0.1248 | 0.9996 | NA |
| AAEL008171 | | 0.4539 | 6.1740 | 0.1249 | 0.9996 | double-stranded RNA-binding protein zn72d [Source:VB Community Annotation] |
| AAEL003874 | | -1.0358 | 1.7722 | 0.1251 | 0.9996 | |
| AAEL023148 | NA | 1.4511 | 2.9430 | 0.1252 | 0.9996 | NA |
| AAEL013627 | | -0.7633 | 4.4164 | 0.1252 | 0.9996 | |
| AAEL006858 | | 0.5596 | 3.7728 | 0.1252 | 0.9996 | |
| AAEL002758 | | 0.4865 | 5.1539 | 0.1258 | 0.9996 | |
| AAEL013989 | | 1.5358 | 5.6305 | 0.1262 | 0.9996 | protein translocation complex beta subunit, putative [Source:VB Community Annotation] |
| AAEL018215 | | -2.2661 | -0.4890 | 0.1263 | 0.9996 | |
| AAEL019808 | NA | 0.5861 | 2.0030 | 0.1264 | 0.9996 | NA |

| | | | | | | |
|---|---|---|---|---|---|---|
| AAEL006322 | | -0.5711 | 5.9195 | 0.1265 | 0.9996 | |
| AAEL009958 | | 0.5384 | 3.6004 | 0.1268 | 0.9996 | |
| AAEL003865 | | 1.6151 | -0.2137 | 0.1270 | 0.9996 | |
| AAEL000033 | | 1.1539 | 2.5180 | 0.1270 | 0.9996 | |
| AAEL002765 | SVP | 1.1411 | 2.7486 | 0.1270 | 0.9996 | Sevenup nuclear receptor [Source:VB Community Annotation] |
| AAEL012327 | | -0.3731 | 5.8690 | 0.1272 | 0.9996 | PET191 polypeptide, putative [Source:VB Community Annotation] |
| AAEL006063 | | 2.5987 | -0.7600 | 0.1272 | 0.9996 | U3 small nucleolar ribonucleoprotein protein imp4 [Source:VB Community Annotation] |
| AAEL003635 | | 0.4051 | 3.7665 | 0.1273 | 0.9996 | |
| AAEL022704 | NA | -0.8655 | 7.5888 | 0.1275 | 0.9996 | NA |
| AAEL008050 | | -1.3654 | 3.5725 | 0.1275 | 0.9996 | |
| AAEL022692 | NA | 0.5421 | 5.5382 | 0.1276 | 0.9996 | NA |
| AAEL003066 | | 1.5741 | 1.3294 | 0.1276 | 0.9996 | brain chitinase and chia [Source:VB Community Annotation] |
| AAEL017003 | | 1.2322 | 2.8167 | 0.1278 | 0.9996 | Clip-domain serine protease, family B [Source:VB Community Annotation] |
| AAEL004986 | | -0.7103 | 4.1474 | 0.1282 | 0.9996 | smg-7 (suppressor with morphological effect on genitalia protein 7) [Source:VB Community Annotation] |
| AAEL008435 | | 0.8175 | 2.2528 | 0.1282 | 0.9996 | |
| AAEL006675 | | 0.6220 | 6.1783 | 0.1282 | 0.9996 | RNA-binding protein, putative [Source:VB Community Annotation] |
| AAEL026341 | NA | -0.8544 | 1.7653 | 0.1283 | 0.9996 | NA |
| AAEL001287 | | -0.5750 | 7.8481 | 0.1285 | 0.9996 | |
| AAEL004132 | Med31 | -0.5281 | 5.4430 | 0.1287 | 0.9996 | Mediator of RNA polymerase II transcription subunit 31 (Med31) [Source:VB Community Annotation] |
| AAEL023625 | NA | -0.8892 | 1.0379 | 0.1288 | 0.9996 | NA |
| AAEL004449 | | 1.1346 | 3.0151 | 0.1292 | 0.9996 | |
| AAEL008378 | | -1.4596 | 0.0418 | 0.1292 | 0.9996 | preproacrosin, putative [Source:VB Community Annotation] |
| AAEL005901 | RpS3a | -0.5121 | 9.7161 | 0.1296 | 0.9996 | 40S ribosomal protein S3a [Source:UniProtKB/Swiss-Prot;Acc:Q1HRR3] |
| AAEL004356 | | 0.5143 | 4.8560 | 0.1299 | 0.9996 | |
| AAEL007808 | CYP4D39 | 1.6156 | 4.7830 | 0.1300 | 0.9996 | cytochrome P450 [Source:VB Community Annotation] |
| AAEL009595 | | 0.6816 | 3.1270 | 0.1300 | 0.9996 | |
| AAEL009051 | TPX5 | 0.5739 | 3.5063 | 0.1300 | 0.9996 | Thioredoxin Peroxidase. [Source:VB Community Annotation] |
| AAEL007368 | | 0.6186 | 2.8452 | 0.1301 | 0.9996 | |
| AAEL023166 | NA | -0.5087 | 5.4082 | 0.1302 | 0.9996 | NA |
| AAEL023443 | NA | -1.7050 | -1.5686 | 0.1302 | 0.9996 | NA |
| AAEL010046 | | 0.4907 | 3.6267 | 0.1303 | 0.9996 | maintenance of ploidy protein mob1 (mps1 binder 1) [Source:VB Community Annotation] |
| AAEL002012 | | 0.3554 | 4.5720 | 0.1306 | 0.9996 | phosphorylated adaptor for RNA export, putative [Source:VB Community Annotation] |
| AAEL011644 | | 1.8015 | -0.1278 | 0.1306 | 0.9996 | |
| AAEL009964 | | -0.7995 | 8.9967 | 0.1306 | 0.9996 | |
| AAEL021384 | NA | 0.5896 | 2.7652 | 0.1307 | 0.9996 | NA |
| AAEL014729 | | -0.6462 | 4.3400 | 0.1307 | 0.9996 | protein serine/threonine kinase, putative [Source:VB Community Annotation] |
| AAEL007164 | | -0.5154 | 3.8949 | 0.1308 | 0.9996 | |
| AAEL011006 | | 0.5702 | 6.5473 | 0.1308 | 0.9996 | guanylate kinase [Source:VB Community Annotation] |
| AAEL002597 | | 0.4246 | 6.5279 | 0.1309 | 0.9996 | |
| AAEL019771 | NA | -0.8445 | 5.5329 | 0.1309 | 0.9996 | NA |

| | | | | | | |
|---|---|---|---|---|---|---|
| AAEL021738 | NA | -0.4967 | 4.9869 | 0.1313 | 0.9996 | NA |
| AAEL008210 | | -0.3371 | 4.9646 | 0.1313 | 0.9996 | |
| AAEL011264 | | 0.9625 | 5.7006 | 0.1315 | 0.9996 | phosphatidylethanolamine-binding protein [Source:VB Community Annotation] |
| AAEL002129 | | -0.4616 | 5.0550 | 0.1316 | 0.9996 | |
| AAEL003815 | | 0.5532 | 3.5818 | 0.1316 | 0.9996 | zinc finger protein [Source:VB Community Annotation] |
| AAEL001484 | | -0.4505 | 7.7905 | 0.1319 | 0.9996 | chromosome region maintenance protein 1/exportin [Source:VB Community Annotation] |
| AAEL014619 | CYP9J22 | 0.6548 | 5.8970 | 0.1319 | 0.9996 | cytochrome P450 [Source:VB Community Annotation] |
| AAEL017356 | | 0.5584 | 3.4208 | 0.1320 | 0.9996 | |
| AAEL019599 | NA | -1.5144 | 5.3816 | 0.1320 | 0.9996 | NA |
| AAEL008736 | | 0.4507 | 4.4911 | 0.1321 | 0.9996 | p31A, putative [Source:VB Community Annotation] |
| AAEL023398 | NA | 1.3982 | 3.3843 | 0.1321 | 0.9996 | NA |
| AAEL013320 | | 0.6066 | 5.0969 | 0.1325 | 0.9996 | translocon-associated protein, delta subunit [Source:VB Community Annotation] |
| AAEL019589 | NA | 0.4514 | 6.7600 | 0.1325 | 0.9996 | NA |
| AAEL011797 | | -1.3735 | 1.4104 | 0.1327 | 0.9996 | venom allergen [Source:VB Community Annotation] |
| AAEL014082 | OBP34 | 0.7184 | 6.4997 | 0.1328 | 0.9996 | odorant binding protein OBP34 [Source:VB Community Annotation] |
| AAEL019871 | NA | -0.6527 | 4.5350 | 0.1329 | 0.9996 | NA |
| AAEL007566 | | 0.4431 | 5.1137 | 0.1329 | 0.9996 | dynactin subunit P25 [Source:VB Community Annotation] |
| AAEL008489 | | 0.4128 | 6.3918 | 0.1331 | 0.9996 | calcyphosine/tpp [Source:VB Community Annotation] |
| AAEL023095 | NA | 0.6071 | 2.6975 | 0.1332 | 0.9996 | NA |
| AAEL004054 | CYP4G36 | 0.7787 | 5.9367 | 0.1335 | 0.9996 | cytochrome P450 [Source:VB Community Annotation] |
| AAEL022819 | NA | 0.5004 | 7.6100 | 0.1338 | 0.9996 | NA |
| AAEL023465 | NA | -0.6845 | 3.7749 | 0.1340 | 0.9996 | NA |
| AAEL025028 | NA | -0.6862 | 2.7933 | 0.1341 | 0.9996 | NA |
| AAEL013295 | | -0.7410 | 2.8414 | 0.1342 | 0.9996 | |
| AAEL018326 | | -0.6992 | 2.8494 | 0.1343 | 0.9996 | Sodium/nucleoside cotransporter [Source:UniProtKB/TrEMBL;Acc:A0A1S4G7K8 |
| AAEL003005 | | -0.8962 | 3.8135 | 0.1344 | 0.9996 | |
| AAEL007765 | SRPN10 | 0.3175 | 6.2280 | 0.1344 | 0.9996 | Serine Protease Inhibitor (serpin) likely cleavage at K/R. Transcript A. [Source:VB Community Annotation] |
| AAEL001553 | | 1.7137 | -0.9049 | 0.1344 | 0.9996 | |
| AAEL007252 | | -0.4020 | 4.6875 | 0.1345 | 0.9996 | sin3a-associated protein sap130 [Source:VB Community Annotation] |
| AAEL013076 | | -0.4202 | 7.3540 | 0.1346 | 0.9996 | signal recognition particle 54 kda protein [Source:VB Community Annotation] |
| AAEL007329 | | -0.6646 | 5.3771 | 0.1347 | 0.9996 | |
| AAEL024778 | NA | -0.9051 | 2.4146 | 0.1349 | 0.9996 | NA |
| AAEL000181 | | -1.4243 | 0.0765 | 0.1350 | 0.9996 | polybromo-1 [Source:VB Community Annotation] |
| AAEL003995 | | -0.4017 | 5.1496 | 0.1350 | 0.9996 | zinc binding dehydrogenase [Source:VB Community Annotation] |
| AAEL013620 | | 0.5007 | 5.9385 | 0.1352 | 0.9996 | ras-related protein Rab, putative [Source:VB Community Annotation] |
| AAEL018144 | | 0.4859 | 4.6305 | 0.1354 | 0.9996 | |
| AAEL024690 | NA | 0.4197 | 5.1559 | 0.1357 | 0.9996 | NA |
| AAEL001551 | | 0.8602 | 1.9936 | 0.1358 | 0.9996 | |
| AAEL022982 | NA | -0.9529 | 5.8650 | 0.1361 | 0.9996 | NA |
| AAEL002801 | | 0.6757 | 2.7630 | 0.1361 | 0.9996 | |

| | | | | | | |
|---|---|---|---|---|---|---|
| AAEL028080 | NA | 0.9107 | 1.1558 | 0.1366 | 0.9996 | NA |
| AAEL023463 | NA | 1.3875 | 0.6103 | 0.1372 | 0.9996 | NA |
| AAEL019987 | NA | 1.2442 | 1.0686 | 0.1374 | 0.9996 | NA |
| AAEL010866 | | 0.9036 | 4.6629 | 0.1375 | 0.9996 | serine protease [Source:VB Community Annotation] |
| AAEL006936 | | -1.3481 | -0.4432 | 0.1377 | 0.9996 | |
| AAEL024412 | NA | -0.3427 | 5.1068 | 0.1378 | 0.9996 | NA |
| AAEL024164 | NA | 0.6480 | 3.2770 | 0.1378 | 0.9996 | NA |
| AAEL023253 | NA | 1.0743 | 3.7347 | 0.1380 | 0.9996 | NA |
| AAEL005395 | | 0.9240 | 1.6768 | 0.1380 | 0.9996 | |
| AAEL007050 | | -0.4780 | 5.1406 | 0.1381 | 0.9996 | sugar transporter [Source:VB Community Annotation] |
| AAEL002978 | | 0.4402 | 5.5615 | 0.1382 | 0.9996 | leucyl aminopeptidase, putative [Source:VB Community Annotation] |
| AAEL018040 | | -0.5411 | 4.7565 | 0.1383 | 0.9996 | |
| AAEL005058 | | 0.6193 | 4.6045 | 0.1385 | 0.9996 | |
| AAEL027748 | NA | 0.6943 | 2.6655 | 0.1385 | 0.9996 | NA |
| AAEL021026 | NA | 0.8954 | 3.7323 | 0.1386 | 0.9996 | NA |
| AAEL024186 | NA | 1.0780 | 5.6370 | 0.1386 | 0.9996 | NA |
| AAEL011972 | | -0.5025 | 7.3701 | 0.1387 | 0.9996 | actin binding protein, putative [Source:VB Community Annotation] |
| AAEL026446 | NA | 0.6491 | 6.4157 | 0.1388 | 0.9996 | NA |
| AAEL009798 | | 1.2101 | 2.2581 | 0.1389 | 0.9996 | transcription factor IIIA, putative [Source:VB Community Annotation] |
| AAEL013826 | | 1.6199 | 3.5477 | 0.1390 | 0.9996 | serine/threonine protein kinase lats [Source:VB Community Annotation] |
| AAEL008327 | | 1.4021 | 0.7526 | 0.1391 | 0.9996 | huntingtin-interacting protein, putative [Source:VB Community Annotation] |
| AAEL002348 | | -0.7230 | 4.3091 | 0.1392 | 0.9996 | paired box protein pax-6 [Source:VB Community Annotation] |
| AAEL009500 | | 1.3998 | 0.1523 | 0.1392 | 0.9996 | |
| AAEL004457 | | -0.7190 | 10.5548 | 0.1394 | 0.9996 | cytochrome c [Source:VB Community Annotation] |
| AAEL014957 | | -0.4748 | 4.8571 | 0.1394 | 0.9996 | |
| AAEL010371 | | 0.9268 | 5.5396 | 0.1395 | 0.9996 | autotransporter adhesin precursor, putative [Source:VB Community Annotation] |
| AAEL017508 | | 0.8145 | 5.2015 | 0.1398 | 0.9996 | |
| AAEL003087 | | 0.4869 | 3.0140 | 0.1399 | 0.9996 | |
| AAEL021828 | NA | 1.0415 | 2.8622 | 0.1402 | 0.9996 | NA |
| AAEL018259 | | 0.4103 | 4.6705 | 0.1403 | 0.9996 | |
| AAEL015575 | | -0.3663 | 6.1665 | 0.1403 | 0.9996 | mitochondrial import inner membrane translocase subunit tim17 [Source:VB Community Annotation] |
| AAEL023169 | NA | -0.5723 | 5.1960 | 0.1407 | 0.9996 | NA |
| AAEL007167 | | 0.5710 | 2.8809 | 0.1409 | 0.9996 | |
| AAEL006329 | | -1.4314 | 1.4033 | 0.1411 | 0.9996 | |
| AAEL008292 | GPRDIH1 | -0.7719 | 4.6013 | 0.1413 | 0.9996 | GPCR Diuretic Insect Hormone/Kinin/CRF Family [Source:VB Community Annotation] |
| AAEL023247 | NA | -0.8773 | 1.2031 | 0.1414 | 0.9996 | NA |
| AAEL006516 | | 0.4439 | 4.9167 | 0.1415 | 0.9996 | vacuolar ATP synthase subunit h [Source:VB Community Annotation] |
| AAEL005414 | | -0.4090 | 8.0089 | 0.1416 | 0.9996 | |
| AAEL007952 | | -1.0210 | 0.7344 | 0.1417 | 0.9996 | |
| AAEL005201 | | 0.7574 | 2.7311 | 0.1418 | 0.9996 | hydroxymethylglutaryl-coa synthase [Source:VB Community Annotation] |

| | | | | | | |
|---|---|---|---|---|---|---|
| AAEL027666 | NA | -0.4650 | 3.9163 | 0.1419 | 0.9996 | NA |
| AAEL013531 | | 0.6906 | 4.3631 | 0.1420 | 0.9996 | cyclin k [Source:VB Community Annotation] |
| AAEL011592 | | 1.6322 | 1.4293 | 0.1423 | 0.9996 | secreted mucin MUC17, putative [Source:VB Community Annotation] |
| AAEL026819 | NA | -0.7152 | 2.2543 | 0.1423 | 0.9996 | NA |
| AAEL006023 | | 1.0667 | 1.2561 | 0.1424 | 0.9996 | Vanin-like protein 1 precursor, putative [Source:VB Community Annotation] |
| AAEL023449 | NA | -1.0494 | 4.4757 | 0.1426 | 0.9996 | NA |
| AAEL001874 | | -1.2592 | -0.3047 | 0.1428 | 0.9996 | cop9 complex subunit [Source:VB Community Annotation] |
| AAEL006639 | | -0.5961 | 3.4307 | 0.1429 | 0.9996 | |
| AAEL017512 | | -0.3597 | 5.0124 | 0.1429 | 0.9996 | Beta-glucuronidase [Source:UniProtKB/TrEMBL;Acc:A0A1S4G6Y4] |
| AAEL010516 | APG4A | 0.5738 | 3.1122 | 0.1430 | 0.9996 | autophagy related gene [Source:VB Community Annotation] |
| AAEL014248 | | -0.5537 | 5.8708 | 0.1430 | 0.9996 | |
| AAEL010520 | | -0.4446 | 6.1002 | 0.1430 | 0.9996 | |
| AAEL002548 | Tsc1 | -0.5266 | 5.3691 | 0.1430 | 0.9996 | tuberous sclerosis 1 [Source:VB Community Annotation] |
| AAEL007865 | | 0.4501 | 6.0252 | 0.1430 | 0.9996 | |
| AAEL003684 | | 0.6514 | 3.8488 | 0.1431 | 0.9996 | ku P80 DNA helicase [Source:VB Community Annotation] |
| AAEL019436 | NA | -0.6876 | 5.4548 | 0.1436 | 0.9996 | NA |
| AAEL025361 | NA | -2.1296 | -1.4963 | 0.1441 | 0.9996 | NA |
| AAEL001033 | MBD | 0.3856 | 4.9799 | 0.1441 | 0.9996 | methyl-cpg binding protein, mbd [Source:VB Community Annotation] |
| AAEL007495 | | 1.3435 | 4.1207 | 0.1441 | 0.9996 | phosphoglycerate mutase [Source:VB Community Annotation] |
| AAEL005622 | | -2.0180 | -0.1463 | 0.1444 | 0.9996 | |
| AAEL000299 | | 0.5476 | 2.8721 | 0.1445 | 0.9996 | |
| AAEL008854 | | 0.7046 | 6.2968 | 0.1447 | 0.9996 | |
| AAEL009675 | | 0.5597 | 2.8391 | 0.1448 | 0.9996 | |
| AAEL007893 | | 0.4529 | 5.6324 | 0.1449 | 0.9996 | short chain type dehydrogenase [Source:VB Community Annotation] |
| AAEL025344 | NA | 0.3769 | 5.7514 | 0.1450 | 0.9996 | NA |
| AAEL003546 | | 0.6109 | 2.5551 | 0.1451 | 0.9996 | gamma-tubulin complex component 4 (gcp-4) [Source:VB Community Annotation] |
| AAEL009190 | | -0.7270 | 4.6387 | 0.1451 | 0.9996 | |
| AAEL019555 | NA | -0.3916 | 4.9803 | 0.1451 | 0.9996 | NA |
| AAEL002999 | | -0.4530 | 3.7511 | 0.1452 | 0.9996 | GTPase_rho [Source:VB Community Annotation] |
| AAEL004348 | | 0.5345 | 2.5366 | 0.1453 | 0.9996 | NF-180, putative [Source:VB Community Annotation] |
| AAEL001156 | | -1.5473 | -1.2734 | 0.1456 | 0.9996 | |
| AAEL012720 | | 0.4795 | 4.3902 | 0.1456 | 0.9996 | |
| AAEL015586 | | -1.4802 | 0.5500 | 0.1459 | 0.9996 | |
| AAEL014391 | | 0.7339 | 3.9523 | 0.1461 | 0.9996 | map kinase phosphatase [Source:VB Community Annotation] |
| AAEL012263 | | 0.5624 | 2.8470 | 0.1462 | 0.9996 | zinc finger protein [Source:VB Community Annotation] |
| AAEL000044 | | 1.1152 | 3.5031 | 0.1462 | 0.9996 | ornithine decarboxylase [Source:VB Community Annotation] |
| AAEL003142 | | 0.4578 | 5.9351 | 0.1465 | 0.9996 | |
| AAEL002864 | | -0.5986 | 5.0871 | 0.1468 | 0.9996 | |
| AAEL007974 | | 0.5219 | 4.4068 | 0.1468 | 0.9996 | |
| AAEL004040 | | 0.6370 | 4.1698 | 0.1468 | 0.9996 | protein phsophatase-2a [Source:VB Community Annotation] |

| | | | | | | |
|---|---|---|---|---|---|---|
| AAEL004866 | | 0.6940 | 4.1189 | 0.1468 | 0.9996 | protein farnesyltransferase beta subunit [Source:VB Community Annotation] |
| AAEL010360 | | -0.4328 | 4.6927 | 0.1472 | 0.9996 | Cytosolic Fe-S cluster assembly factor NUBP1 homolog [Source:VB Community Annotation] |
| AAEL014339 | | 0.4029 | 5.0922 | 0.1473 | 0.9996 | |
| AAEL025853 | NA | -1.9952 | -2.1037 | 0.1473 | 0.9996 | NA |
| AAEL000272 | | 0.4869 | 3.8674 | 0.1474 | 0.9996 | |
| AAEL005092 | | -0.4771 | 6.9100 | 0.1474 | 0.9996 | n-myristoyl transferase [Source:VB Community Annotation] |
| AAEL002924 | | 0.4241 | 5.1610 | 0.1475 | 0.9996 | |
| AAEL004952 | | -1.5602 | 4.2930 | 0.1477 | 0.9996 | protein N-terminal asparagine amidohydrolase, putative [Source:VB Community Annotation] |
| AAEL004218 | Or85 | -1.2603 | 0.2301 | 0.1478 | 0.9996 | Odorant receptor [Source:UniProtKB/TrEMBL;Acc:Q17DH7] |
| AAEL002016 | | 0.4078 | 3.6533 | 0.1479 | 0.9996 | disulfide oxidoreductase [Source:VB Community Annotation] |
| AAEL020642 | NA | -0.4313 | 4.3019 | 0.1479 | 0.9996 | NA |
| AAEL009099 | | 0.4611 | 5.8018 | 0.1481 | 0.9996 | uridine cytidine kinase i [Source:VB Community Annotation] |
| AAEL024063 | NA | 0.5353 | 6.3621 | 0.1481 | 0.9996 | NA |
| AAEL006947 | GPRTAK2 | 1.7555 | -2.0856 | 0.1483 | 0.9996 | GPCR Neurokinin/Tachykinin Family [Source:VB Community Annotation] |
| AAEL024597 | NA | -0.8683 | 1.6765 | 0.1483 | 0.9996 | NA |
| AAEL013752 | | -1.2224 | 2.4131 | 0.1486 | 0.9996 | rfx5 [Source:VB Community Annotation] |
| AAEL019722 | NA | -0.5753 | 4.4265 | 0.1487 | 0.9996 | NA |
| AAEL023172 | NA | 0.9626 | 1.0049 | 0.1491 | 0.9996 | NA |
| AAEL025775 | NA | -0.7587 | 1.9626 | 0.1492 | 0.9996 | NA |
| AAEL004409 | | -0.7865 | 7.6566 | 0.1492 | 0.9996 | yellow protein, putative [Source:VB Community Annotation] |
| AAEL001962 | | -0.3431 | 4.7962 | 0.1493 | 0.9996 | |
| AAEL013575 | king-tubby | -1.4084 | -0.5964 | 0.1496 | 0.9996 | Protein king tubby 2 [Source:UniProtKB/Swiss-Prot;Acc:Q16IR1] |
| AAEL000403 | | 1.4865 | -0.4115 | 0.1497 | 0.9996 | |
| AAEL003088 | | -1.5505 | -0.3056 | 0.1497 | 0.9996 | |
| AAEL006834 | | -1.0525 | 9.0969 | 0.1497 | 0.9996 | glutamate semialdehyde dehydrogenase [Source:VB Community Annotation] |
| AAEL001194 | FAS1 | -0.6238 | 8.5766 | 0.1498 | 0.9996 | fatty acid synthase [Source:VB Community Annotation] |
| AAEL014610 | CYP9J29 | -0.9347 | 2.3721 | 0.1498 | 0.9996 | cytochrome P450 [Source:VB Community Annotation] |
| AAEL001372 | | -0.3238 | 4.6047 | 0.1500 | 0.9996 | sentrin/sumo-specific protease senp7 [Source:VB Community Annotation] |
| AAEL006048 | | -0.3396 | 5.9403 | 0.1502 | 0.9996 | interleukin enhancer binding factor [Source:VB Community Annotation] |
| AAEL002231 | | -0.9857 | 8.4446 | 0.1503 | 0.9996 | cuticle protein, putative [Source:VB Community Annotation] |
| AAEL019523 | NA | -0.7478 | 7.4195 | 0.1504 | 0.9996 | NA |
| AAEL007382 | | 0.5064 | 5.8535 | 0.1506 | 0.9996 | serine-threonine kinase receptor-associated protein (strap) [Source:VB Community Annotation] |
| AAEL011404 | CTL19 | -1.2750 | 0.2660 | 0.1506 | 0.9996 | C-Type Lectin (CTL19) [Source:VB Community Annotation] |
| AAEL025217 | NA | -0.5511 | 5.4217 | 0.1507 | 0.9996 | NA |
| AAEL010312 | | 0.4297 | 4.9390 | 0.1508 | 0.9996 | |
| AAEL001904 | | 0.5289 | 4.2074 | 0.1511 | 0.9996 | Arp2/3 complex 34 kDa subunit [Source:UniProtKB/TrEMBL;Acc:Q17JV8] |
| AAEL005952 | GPRDOP5 | -1.7641 | 1.2811 | 0.1513 | 0.9996 | GPCR Dopamine Family [Source:VB Community Annotation] |
| AAEL011904 | | 0.5676 | 2.7012 | 0.1513 | 0.9996 | zinc finger protein [Source:VB Community Annotation] |
| AAEL012816 | | 0.4971 | 4.5309 | 0.1515 | 0.9996 | |
| AAEL011522 | | 0.7613 | 2.1382 | 0.1517 | 0.9996 | MRAS2, putative [Source:VB Community Annotation] |

| | | | | | | |
|---|---|---|---|---|---|---|
| AAEL019388 | Metazoa_S | -1.1659 | 5.4185 | 0.1518 | 0.9996 | Metazoan signal recognition particle RNA [Source:RFAM;Acc:RF00017] |
| AAEL014534 | | 0.5133 | 4.1124 | 0.1518 | 0.9996 | |
| AAEL003544 | | 0.6288 | 2.8468 | 0.1521 | 0.9996 | |
| AAEL007059 | | 0.4969 | 4.8194 | 0.1523 | 0.9996 | |
| AAEL008605 | | -0.4158 | 4.9778 | 0.1523 | 0.9996 | inosine triphosphate pyrophosphatase (itpase) (inosine triphosphatase) [Source:VB Community Annotation] |
| AAEL002410 | | -2.5001 | 0.8832 | 0.1525 | 0.9996 | |
| AAEL002904 | | -1.2971 | 2.0442 | 0.1526 | 0.9996 | juvenile hormone-inducible protein, putative [Source:VB Community Annotation] |
| AAEL009791 | | -1.5844 | 1.0862 | 0.1528 | 0.9996 | cuticle protein, putative [Source:VB Community Annotation] |
| AAEL012395 | | 0.5614 | 2.5971 | 0.1530 | 0.9996 | ATP-binding cassette transporter [Source:VB Community Annotation] |
| AAEL000812 | | -0.6258 | 3.5336 | 0.1530 | 0.9996 | |
| AAEL006978 | | 0.9043 | 4.1654 | 0.1531 | 0.9996 | protein-glutamine gamma-glutamyltransferase [Source:VB Community Annotation] |
| AAEL000087 | | -0.9073 | 4.4663 | 0.1532 | 0.9996 | macroglobulin/complement [Source:VB Community Annotation] |
| AAEL002843 | | -0.5599 | 3.1110 | 0.1532 | 0.9996 | |
| AAEL001212 | | -1.8447 | -0.8589 | 0.1532 | 0.9996 | phosphoinositide-binding protein, putative [Source:VB Community Annotation] |
| AAEL012092 | | -1.3470 | 5.4963 | 0.1532 | 0.9996 | leucine-rich repeat [Source:VB Community Annotation] |
| AAEL002238 | | 0.5786 | 4.1862 | 0.1535 | 0.9996 | |
| AAEL027402 | NA | -0.3866 | 7.5829 | 0.1535 | 0.9996 | NA |
| AAEL008458 | | 1.4129 | 3.8867 | 0.1536 | 0.9996 | |
| AAEL003709 | | 0.5701 | 3.2808 | 0.1539 | 0.9996 | crotonobetainyl-CoA-hydratase, putative [Source:VB Community Annotation] |
| AAEL008575 | | 0.8021 | 3.2656 | 0.1539 | 0.9996 | regulator of chromosome condensation [Source:VB Community Annotation] |
| AAEL010911 | dbo | 0.7345 | 3.9094 | 0.1539 | 0.9996 | Kelch-like protein diablo [Source:UniProtKB/Swiss-Prot;Acc:Q16RL8] |
| AAEL002021 | | -1.8535 | -1.7200 | 0.1542 | 0.9996 | protein serine/threonine kinase, putative [Source:VB Community Annotation] |
| AAEL011462 | | -0.5413 | 3.7058 | 0.1543 | 0.9996 | Transcription initiation factor IIE subunit beta [Source:UniProtKB/TrEMBL;Acc:Q16Q16] |
| AAEL012674 | | -0.6124 | 5.4824 | 0.1546 | 0.9996 | d-amino acid oxidase [Source:VB Community Annotation] |
| AAEL010634 | | -1.6436 | 4.5860 | 0.1547 | 0.9996 | |
| AAEL022589 | NA | -1.2536 | 3.2719 | 0.1549 | 0.9996 | NA |
| AAEL004743 | | 0.4376 | 4.4754 | 0.1550 | 0.9996 | multidrug resistance protein 2 (ATP-binding cassette protein c) [Source:VB Community Annotation] |
| AAEL020021 | NA | 0.6300 | 4.6042 | 0.1551 | 0.9996 | NA |
| AAEL008665 | | 1.3378 | -0.8709 | 0.1552 | 0.9996 | |
| AAEL010796 | | 0.4363 | 3.2975 | 0.1552 | 0.9996 | transmembrane protein 1/tmem1b [Source:VB Community Annotation] |
| AAEL006886 | | 1.4074 | 2.5436 | 0.1555 | 0.9996 | |
| AAEL000008 | | 0.5982 | 3.2166 | 0.1556 | 0.9996 | WD and tetratricopeptide repeat protein [Source:VB Community Annotation] |
| AAEL024130 | NA | -1.5307 | -1.2704 | 0.1556 | 0.9996 | NA |
| AAEL001937 | | 0.6225 | 4.1177 | 0.1556 | 0.9996 | selenium-binding protein [Source:VB Community Annotation] |
| AAEL007969 | | 1.9891 | 3.6688 | 0.1557 | 0.9996 | serine protease [Source:VB Community Annotation] |
| AAEL013769 | | 0.5248 | 4.5500 | 0.1560 | 0.9996 | exocyst complex component sec6 [Source:VB Community Annotation] |
| AAEL011090 | | -0.3907 | 5.5960 | 0.1560 | 0.9996 | complement component [Source:VB Community Annotation] |
| AAEL026102 | NA | 1.6548 | -0.9271 | 0.1560 | 0.9996 | NA |
| AAEL014851 | | -0.6700 | 5.7352 | 0.1560 | 0.9996 | mediator complex subunit rgr-1 [Source:VB Community Annotation] |
| AAEL014970 | | -0.4236 | 6.8131 | 0.1562 | 0.9996 | |

| | | | | | | |
|---|---|---|---|---|---|---|
| AAEL006677 | | -0.4390 | 5.9334 | 0.1563 | 0.9996 | phospholipase a-2-activating protein [Source:VB Community Annotation] |
| AAEL001277 | | 1.4134 | 0.9836 | 0.1563 | 0.9996 | arf GTPase-activating protein [Source:VB Community Annotation] |
| AAEL013061 | | -1.0000 | 0.4293 | 0.1563 | 0.9996 | SM protein G, putative [Source:VB Community Annotation] |
| AAEL002362 | | -0.6276 | 3.5635 | 0.1566 | 0.9996 | |
| AAEL003424 | | 1.8597 | -1.6829 | 0.1567 | 0.9996 | zinc carboxypeptidase [Source:VB Community Annotation] |
| AAEL011924 | | -0.4893 | 4.3958 | 0.1568 | 0.9996 | |
| AAEL006934 | Med19 | 0.3507 | 5.0378 | 0.1569 | 0.9996 | Mediator of RNA polymerase II transcription subunit 19 (Med19) [Source:VB Community Annotation] |
| AAEL027209 | NA | -1.1614 | 2.8452 | 0.1569 | 0.9996 | NA |
| AAEL018304 | | -0.7005 | 3.3198 | 0.1571 | 0.9996 | |
| AAEL014899 | | -0.7396 | 4.1451 | 0.1571 | 0.9996 | short chain type dehydrogenase [Source:VB Community Annotation] |
| AAEL002773 | | 1.3888 | -0.7565 | 0.1572 | 0.9996 | |
| AAEL027982 | NA | -0.8334 | 2.1167 | 0.1572 | 0.9996 | NA |
| AAEL012496 | | -1.5680 | -0.6194 | 0.1573 | 0.9996 | |
| AAEL012391 | | 0.7171 | 1.6070 | 0.1574 | 0.9996 | |
| AAEL007539 | | -0.7818 | 4.3041 | 0.1575 | 0.9996 | |
| AAEL010001 | | 0.6759 | 2.4697 | 0.1576 | 0.9996 | |
| AAEL005489 | | -1.2496 | -0.2129 | 0.1579 | 0.9996 | olfactory receptor, putative [Source:VB Community Annotation] |
| AAEL007936 | | -0.3215 | 5.9286 | 0.1579 | 0.9996 | |
| AAEL025807 | NA | 0.3561 | 5.5933 | 0.1580 | 0.9996 | NA |
| AAEL024216 | NA | -0.6222 | 4.8052 | 0.1581 | 0.9996 | NA |
| AAEL006228 | | 0.5253 | 3.9983 | 0.1581 | 0.9996 | |
| AAEL027664 | NA | 0.9571 | 1.6997 | 0.1581 | 0.9996 | NA |
| AAEL024938 | NA | 1.0306 | 2.4343 | 0.1583 | 0.9996 | NA |
| AAEL010956 | | 0.4984 | 5.3463 | 0.1584 | 0.9996 | |
| AAEL006713 | | -0.6007 | 6.6498 | 0.1585 | 0.9996 | U2 snrnp auxiliary factor, small subunit [Source:VB Community Annotation] |
| AAEL002680 | | 0.9511 | 3.3732 | 0.1586 | 0.9996 | AMP dependent ligase [Source:VB Community Annotation] |
| AAEL026651 | NA | 0.5096 | 2.8800 | 0.1589 | 0.9996 | NA |
| AAEL000645 | | -0.6067 | 5.3074 | 0.1589 | 0.9996 | |
| AAEL023529 | NA | 0.6826 | 3.9406 | 0.1591 | 0.9996 | NA |
| AAEL006315 | | 0.3585 | 5.2471 | 0.1595 | 0.9996 | 26S proteasome regulatory subunit rpn1 [Source:VB Community Annotation] |
| AAEL011728 | | -0.5677 | 6.6859 | 0.1597 | 0.9996 | |
| AAEL021813 | NA | 1.6290 | -0.7929 | 0.1599 | 0.9996 | NA |
| AAEL007536 | | -0.3319 | 5.5534 | 0.1599 | 0.9996 | carnitine o-acyltransferase [Source:VB Community Annotation] |
| AAEL007704 | | 0.6659 | 2.6712 | 0.1600 | 0.9996 | lipoma preferred partner/lpp [Source:VB Community Annotation] |
| AAEL007023 | | -0.5089 | 7.3111 | 0.1600 | 0.9996 | estradiol 17 beta-dehydrogenase [Source:VB Community Annotation] |
| AAEL019762 | NA | 0.6322 | 5.1470 | 0.1600 | 0.9996 | NA |
| AAEL000487 | | 0.5316 | 3.2940 | 0.1601 | 0.9996 | |
| AAEL002293 | | -0.6174 | 5.8461 | 0.1603 | 0.9996 | |
| AAEL002854 | | 0.6137 | 2.4982 | 0.1606 | 0.9996 | |
| AAEL009898 | Prosalpha1 | 0.4134 | 5.8912 | 0.1608 | 0.9996 | 26S proteasome alpha 1 subunit [Source:VB Community Annotation] |

| | | | | | | |
|---|---|---|---|---|---|---|
| AAEL004852 | | 0.4447 | 3.9397 | 0.1608 | 0.9996 | |
| AAEL002296 | | 0.6788 | 7.7881 | 0.1608 | 0.9996 | trifunctional enzyme beta subunit (tp-beta) [Source:VB Community Annotation] |
| AAEL023681 | NA | -0.4338 | 10.0168 | 0.1608 | 0.9996 | NA |
| AAEL015458 | Tf1 | -1.0123 | 10.5645 | 0.1610 | 0.9996 | transferrin [Source:VB Community Annotation] |
| AAEL009592 | | 1.1074 | 0.1239 | 0.1612 | 0.9996 | |
| AAEL001954 | | 0.5122 | 3.2080 | 0.1620 | 0.9996 | px serine/threonine kinase (pxk) [Source:VB Community Annotation] |
| AAEL010125 | LRIM17 | -1.4298 | 4.8610 | 0.1621 | 0.9996 | leucine-rich immune protein (Coil-less) [Source:VB Community Annotation] |
| AAEL024051 | NA | 0.8979 | 1.3211 | 0.1621 | 0.9996 | NA |
| AAEL009160 | | -0.3883 | 8.6718 | 0.1622 | 0.9996 | skp1 [Source:VB Community Annotation] |
| AAEL002856 | | -0.7272 | 4.2714 | 0.1622 | 0.9996 | |
| AAEL002240 | | 0.3580 | 5.7970 | 0.1623 | 0.9996 | signal transducing adapter molecule (stam) [Source:VB Community Annotation] |
| AAEL026371 | NA | -1.1611 | 1.3201 | 0.1624 | 0.9996 | NA |
| AAEL013138 | | 0.5799 | 7.1217 | 0.1624 | 0.9996 | |
| AAEL027447 | NA | -0.7944 | 9.2382 | 0.1626 | 0.9996 | NA |
| AAEL004163 | | 0.7534 | 2.0697 | 0.1626 | 0.9996 | O-fucosyltransferase, putative [Source:VB Community Annotation] |
| AAEL022535 | NA | -0.3719 | 6.1391 | 0.1627 | 0.9996 | NA |
| AAEL003245 | IKK1 | 0.5187 | 3.8295 | 0.1627 | 0.9996 | IMD pathway signalling I-Kappa-B Kinase 1 (IKK1 IKK-beta). (ird5 orthologue immune response deficient 5) [Source:VB Community Ann |
| AAEL027326 | NA | -0.7068 | 4.5680 | 0.1629 | 0.9996 | NA |
| AAEL000448 | | 0.7142 | 3.3828 | 0.1631 | 0.9996 | |
| AAEL012689 | | -0.5220 | 8.2871 | 0.1631 | 0.9996 | glucose-methanol-choline (gmc) oxidoreductase [Source:VB Community Annotation] |
| AAEL014355 | | 0.8343 | 3.4882 | 0.1634 | 0.9996 | symbol, putative [Source:VB Community Annotation] |
| AAEL023221 | NA | 0.5336 | 3.1456 | 0.1635 | 0.9996 | NA |
| AAEL000683 | | -1.7360 | -2.0226 | 0.1636 | 0.9996 | |
| AAEL028239 | NA | 0.7355 | 5.5926 | 0.1637 | 0.9996 | NA |
| AAEL009792 | LRIM25 | -0.6796 | 6.1403 | 0.1637 | 0.9996 | leucine-rich immune protein (Coil-less) [Source:VB Community Annotation] |
| AAEL023649 | NA | 1.2647 | 0.8297 | 0.1639 | 0.9996 | NA |
| AAEL021176 | NA | 0.4881 | 6.1559 | 0.1639 | 0.9996 | NA |
| AAEL005001 | | 1.3219 | 3.2257 | 0.1639 | 0.9996 | aquaporin [Source:VB Community Annotation] |
| AAEL008073 | | -0.5306 | 8.3136 | 0.1639 | 0.9996 | |
| AAEL001044 | Aats-tyr | 0.4063 | 6.2518 | 0.1642 | 0.9996 | tyrosyl-tRNA synthetase [Source:VB Community Annotation] |
| AAEL010051 | | -0.6983 | 5.1270 | 0.1642 | 0.9996 | cell cycle progression [Source:VB Community Annotation] |
| AAEL004176 | | 0.3970 | 4.6560 | 0.1646 | 0.9996 | microtubule binding protein, putative [Source:VB Community Annotation] |
| AAEL000122 | | 0.4969 | 2.5952 | 0.1646 | 0.9996 | |
| AAEL010790 | | 1.5236 | 0.4037 | 0.1646 | 0.9996 | |
| AAEL021258 | NA | 0.7428 | 2.0263 | 0.1649 | 0.9996 | NA |
| AAEL013287 | | 0.5481 | 6.9443 | 0.1649 | 0.9996 | |
| AAEL009449 | OBP39 | 0.7035 | 8.1212 | 0.1650 | 0.9996 | odorant binding protein (OBP39) [Source:VB Community Annotation] |
| AAEL000965 | | 0.3736 | 4.7259 | 0.1652 | 0.9996 | |
| AAEL021463 | NA | 1.8262 | -1.6879 | 0.1653 | 0.9996 | NA |
| AAEL013400 | | -0.4252 | 3.4324 | 0.1654 | 0.9996 | DEAD box ATP-dependent RNA helicase [Source:VB Community Annotation] |

| | | | | | | |
|---|---|---|---|---|---|---|
| AAEL009200 | Exo1 | 0.5849 | 3.5745 | 0.1654 | 0.9996 | exocyst complex component 1 [Source:VB Community Annotation] |
| AAEL003891 | | -0.5641 | 1.8894 | 0.1654 | 0.9996 | ctl transporter [Source:VB Community Annotation] |
| AAEL011017 | | 0.3815 | 4.5917 | 0.1654 | 0.9996 | |
| AAEL005521 | | 0.8917 | 5.4751 | 0.1655 | 0.9996 | prolylcarboxypeptidase [Source:VB Community Annotation] |
| AAEL009340 | | 0.7138 | 3.2370 | 0.1656 | 0.9996 | biotin protein ligase [Source:VB Community Annotation] |
| AAEL000896 | | -0.9531 | 1.2290 | 0.1656 | 0.9996 | |
| AAEL008467 | | -1.4238 | 0.5280 | 0.1659 | 0.9996 | cysteine synthase [Source:VB Community Annotation] |
| AAEL027940 | NA | -1.9447 | 0.5353 | 0.1660 | 0.9996 | NA |
| AAEL025196 | NA | -2.6034 | 5.2493 | 0.1661 | 0.9996 | NA |
| AAEL004522 | GAM1 | -1.5141 | 8.4013 | 0.1661 | 0.9996 | gambicin anti-microbial peptide [Source:VB Community Annotation] |
| AAEL003385 | | -1.3510 | 0.7179 | 0.1663 | 0.9996 | |
| AAEL014557 | | 1.7484 | -0.9533 | 0.1665 | 0.9996 | homeobox protein cdx [Source:VB Community Annotation] |
| AAEL013921 | | 0.5482 | 3.0736 | 0.1667 | 0.9996 | |
| AAEL010404 | | 0.3585 | 4.8405 | 0.1667 | 0.9996 | |
| AAEL004081 | | -0.3158 | 6.3521 | 0.1670 | 0.9996 | dj-1 protein (park7) [Source:VB Community Annotation] |
| AAEL027406 | NA | -1.4437 | -0.8332 | 0.1671 | 0.9996 | NA |
| AAEL000483 | | 0.4695 | 4.8948 | 0.1671 | 0.9996 | exostosin-2 [Source:VB Community Annotation] |
| AAEL014317 | | 0.5928 | 2.9706 | 0.1674 | 0.9996 | |
| AAEL023158 | NA | 1.3429 | -0.7073 | 0.1676 | 0.9996 | NA |
| AAEL010782 | | 1.3410 | 3.8967 | 0.1677 | 0.9996 | carboxypeptidase [Source:VB Community Annotation] |
| AAEL000859 | | 1.6460 | 3.0812 | 0.1680 | 0.9996 | |
| AAEL019514 | NA | -0.4802 | 5.5085 | 0.1682 | 0.9996 | NA |
| AAEL005426 | | 0.3904 | 4.7803 | 0.1682 | 0.9996 | annexin x [Source:VB Community Annotation] |
| AAEL011701 | | 0.5146 | 3.3505 | 0.1683 | 0.9996 | |
| AAEL026208 | NA | -1.4468 | -1.7750 | 0.1686 | 0.9996 | NA |
| AAEL005885 | | 0.4062 | 4.4571 | 0.1688 | 0.9996 | arginyltransferase, putative [Source:VB Community Annotation] |
| AAEL008687 | loqs | -1.3116 | 3.3752 | 0.1689 | 0.9996 | loquacious [Source:VB Community Annotation] |
| AAEL008153 | | 0.5033 | 5.4543 | 0.1690 | 0.9996 | |
| AAEL000800 | | -0.8308 | 3.5461 | 0.1693 | 0.9996 | microsomal dipeptidase [Source:VB Community Annotation] |
| AAEL010461 | | -0.4508 | 5.2788 | 0.1696 | 0.9996 | |
| AAEL012841 | | 0.4855 | 5.4709 | 0.1697 | 0.9996 | |
| AAEL027354 | NA | 0.7276 | 2.5753 | 0.1698 | 0.9996 | NA |
| AAEL013115 | | -0.6117 | 3.6252 | 0.1699 | 0.9996 | gpi inositol deacylase pgap1 [Source:VB Community Annotation] |
| AAEL026945 | NA | -0.4636 | 4.7870 | 0.1700 | 0.9996 | NA |
| AAEL002525 | | -0.4039 | 4.7775 | 0.1700 | 0.9996 | amino acids transporter [Source:VB Community Annotation] |
| AAEL026808 | NA | 1.6951 | -0.8502 | 0.1701 | 0.9996 | NA |
| AAEL008600 | | 2.2661 | -0.2727 | 0.1701 | 0.9996 | zinc carboxypeptidase [Source:VB Community Annotation] |
| AAEL010017 | | -0.3610 | 8.0845 | 0.1704 | 0.9996 | cytochrome B5 (cytb5) [Source:VB Community Annotation] |
| AAEL006181 | | -0.5105 | 6.4772 | 0.1705 | 0.9996 | amidase [Source:VB Community Annotation] |
| AAEL007480 | | 0.4557 | 3.8210 | 0.1706 | 0.9996 | glutamate-cysteine ligase, regulatory-subunit, putative [Source:VB Community Annotation] |

| | | | | | | |
|---|---|---|---|---|---|---|
| AAEL019724 | NA | -0.4913 | 5.1414 | 0.1706 | 0.9996 | NA |
| AAEL005042 | | -0.7253 | 1.3187 | 0.1707 | 0.9996 | |
| AAEL003611 | | -0.5258 | 5.6109 | 0.1708 | 0.9996 | stearoyl-coa desaturase [Source:VB Community Annotation] |
| AAEL009272 | | -1.1031 | 0.3893 | 0.1709 | 0.9996 | KTI12 protein, putative [Source:VB Community Annotation] |
| AAEL003945 | | -0.4678 | 5.3802 | 0.1709 | 0.9996 | transcription initiation factor IIF alpha subunit [Source:VB Community Annotation] |
| AAEL022326 | NA | 1.6077 | -0.3403 | 0.1710 | 0.9996 | NA |
| AAEL012635 | | 1.7381 | -0.8641 | 0.1712 | 0.9996 | |
| AAEL018022 | | 0.4279 | 4.8076 | 0.1712 | 0.9996 | |
| AAEL019856 | NA | -0.5238 | 6.1300 | 0.1712 | 0.9996 | NA |
| AAEL001541 | | 0.4265 | 3.6850 | 0.1716 | 0.9996 | component of oligomeric golgi complex [Source:VB Community Annotation] |
| AAEL027541 | NA | 0.6841 | 3.0538 | 0.1717 | 0.9996 | NA |
| AAEL014071 | | 0.4285 | 5.8858 | 0.1718 | 0.9996 | |
| AAEL010991 | | -0.4825 | 6.4241 | 0.1719 | 0.9996 | long-chain-fatty-acid coa ligase [Source:VB Community Annotation] |
| AAEL008391 | | 0.4121 | 3.2810 | 0.1722 | 0.9996 | ptx1 protein [Source:VB Community Annotation] |
| AAEL009424 | | 0.5060 | 5.9231 | 0.1722 | 0.9996 | serine/threonine-protein kinase rio3 (rio kinase 3) [Source:VB Community Annotation] |
| AAEL021135 | NA | 1.1728 | -0.4823 | 0.1724 | 0.9996 | NA |
| AAEL002321 | | 0.7614 | 3.3898 | 0.1725 | 0.9996 | paired box protein pax-6 [Source:VB Community Annotation] |
| AAEL008476 | | 0.6296 | 3.1848 | 0.1725 | 0.9996 | |
| AAEL008249 | | 1.6060 | 4.0682 | 0.1726 | 0.9996 | |
| AAEL011813 | | 0.3387 | 5.2086 | 0.1729 | 0.9996 | |
| AAEL023786 | NA | 0.4764 | 5.3425 | 0.1730 | 0.9996 | NA |
| AAEL014416 | | -1.8582 | -0.6046 | 0.1733 | 0.9996 | pupal cuticle protein 78E, putative [Source:VB Community Annotation] |
| AAEL020363 | NA | 0.4555 | 4.6361 | 0.1733 | 0.9996 | NA |
| AAEL010306 | | 0.3724 | 5.5123 | 0.1733 | 0.9996 | |
| AAEL004902 | | 0.5889 | 7.0137 | 0.1734 | 0.9996 | ras-related protein Rab-2A, putative [Source:VB Community Annotation] |
| AAEL012266 | CYP4C38 | 0.7784 | 5.6226 | 0.1735 | 0.9996 | cytochrome P450 [Source:VB Community Annotation] |
| AAEL025132 | NA | 1.6050 | -0.7260 | 0.1737 | 0.9996 | NA |
| AAEL026403 | NA | -0.5002 | 11.8646 | 0.1738 | 0.9996 | NA |
| AAEL024627 | NA | 1.6241 | -1.1324 | 0.1738 | 0.9996 | NA |
| AAEL019575 | NA | 0.6836 | 6.5104 | 0.1739 | 0.9996 | NA |
| AAEL005045 | | 0.5915 | 2.9213 | 0.1742 | 0.9996 | ATP-dependent bile acid permease [Source:VB Community Annotation] |
| AAEL008559 | | 0.4876 | 2.7173 | 0.1743 | 0.9996 | glutaminase [Source:VB Community Annotation] |
| AAEL011848 | | -0.4459 | 4.6302 | 0.1743 | 0.9996 | |
| AAEL027505 | NA | -1.0485 | 0.8625 | 0.1745 | 0.9996 | NA |
| AAEL006620 | | -0.6039 | 3.2576 | 0.1745 | 0.9996 | |
| AAEL024972 | NA | 1.3678 | -0.1019 | 0.1745 | 0.9996 | NA |
| AAEL022430 | NA | -1.1479 | 3.2660 | 0.1746 | 0.9996 | NA |
| AAEL001351 | | -0.3273 | 8.0709 | 0.1749 | 0.9996 | |
| AAEL017142 | | -2.0984 | 0.3396 | 0.1749 | 0.9996 | |
| AAEL008672 | | -0.5092 | 7.8749 | 0.1750 | 0.9996 | ABC transporter [Source:VB Community Annotation] |

| | | | | | | |
|---|---|---|---|---|---|---|
| AAEL001060 | | -1.8330 | -0.8136 | 0.1750 | 0.9996 | |
| AAEL007004 | GPRBOS1 | 0.4906 | 5.7294 | 0.1752 | 0.9996 | GPCR Bride of Sevenless Family [Source:VB Community Annotation] |
| AAEL019723 | NA | -0.5175 | 5.7178 | 0.1752 | 0.9996 | NA |
| AAEL025594 | NA | 0.5124 | 5.7655 | 0.1753 | 0.9996 | NA |
| AAEL007024 | CYP6AG3 | -0.3665 | 6.9559 | 0.1754 | 0.9996 | cytochrome P450 [Source:VB Community Annotation] |
| AAEL006636 | GPRLLK1_1 | -0.5223 | 3.2835 | 0.1755 | 0.9996 | GPCR Leukokinin Family [Source:VB Community Annotation] |
| AAEL005500 | | 0.6791 | 6.0133 | 0.1760 | 0.9996 | |
| AAEL023997 | NA | -0.5646 | 5.9443 | 0.1761 | 0.9996 | NA |
| AAEL020299 | NA | 0.5517 | 3.0604 | 0.1763 | 0.9996 | NA |
| AAEL009507 | | -0.6170 | 5.9437 | 0.1763 | 0.9996 | Glucose-6-phosphate 1-dehydrogenase [Source:UniProtKB/TrEMBL;Acc:Q0IEL8] |
| AAEL007778 | | -0.6606 | 7.6591 | 0.1763 | 0.9996 | leucine-rich transmembrane protein [Source:VB Community Annotation] |
| AAEL002846 | | 0.5453 | 2.6051 | 0.1764 | 0.9996 | inositol triphosphate 3-kinase c [Source:VB Community Annotation] |
| AAEL009878 | | -0.5197 | 5.9087 | 0.1764 | 0.9996 | |
| AAEL000515 | | 1.0482 | 3.2182 | 0.1764 | 0.9996 | juvenile hormone-inducible protein, putative [Source:VB Community Annotation] |
| AAEL023356 | NA | 1.5432 | 0.0185 | 0.1764 | 0.9996 | NA |
| AAEL002055 | | 0.5774 | 7.9021 | 0.1765 | 0.9996 | neuroendocrine protein 7b2 [Source:VB Community Annotation] |
| AAEL001273 | sec24 | 0.5985 | 6.6827 | 0.1765 | 0.9996 | Sec24B protein, putative [Source:VB Community Annotation] |
| AAEL013356 | | 0.5669 | 5.3093 | 0.1766 | 0.9996 | |
| AAEL010869 | | 0.4915 | 5.8994 | 0.1767 | 0.9996 | |
| AAEL025859 | NA | 1.0174 | -0.1184 | 0.1769 | 0.9996 | NA |
| AAEL003916 | | 0.3727 | 4.7518 | 0.1772 | 0.9996 | splicing factor, putative [Source:VB Community Annotation] |
| AAEL008258 | | 0.3317 | 3.2828 | 0.1774 | 0.9996 | threonine aspartase [Source:VB Community Annotation] |
| AAEL026897 | NA | -1.0972 | 1.9335 | 0.1775 | 0.9996 | NA |
| AAEL001505 | | 1.0262 | 2.4376 | 0.1775 | 0.9996 | |
| AAEL010243 | | 0.6451 | 4.1959 | 0.1775 | 0.9996 | Abnormal X segregation, putative [Source:VB Community Annotation] |
| AAEL004563 | | 0.4060 | 5.7041 | 0.1776 | 0.9996 | 26S protease regulatory subunit S10b [Source:VB Community Annotation] |
| AAEL028061 | NA | 1.1476 | 0.0683 | 0.1778 | 0.9996 | NA |
| AAEL002354 | HPX5 | 1.0332 | 2.2108 | 0.1779 | 0.9996 | heme peroxidase [Source:VB Community Annotation] |
| AAEL010487 | | -0.7544 | 4.6734 | 0.1779 | 0.9996 | |
| AAEL019622 | NA | -0.4444 | 4.9727 | 0.1781 | 0.9996 | NA |
| AAEL008909 | | -0.3591 | 4.5210 | 0.1784 | 0.9996 | |
| AAEL013746 | | 0.5973 | 3.6240 | 0.1784 | 0.9996 | |
| AAEL026811 | NA | 0.4002 | 4.5273 | 0.1784 | 0.9996 | NA |
| AAEL002406 | | -0.9518 | 3.2791 | 0.1785 | 0.9996 | |
| AAEL009235 | | -1.8721 | 0.0735 | 0.1786 | 0.9996 | |
| AAEL023274 | NA | 0.9258 | 0.3278 | 0.1788 | 0.9996 | NA |
| AAEL006577 | | -0.3528 | 6.2121 | 0.1790 | 0.9996 | aspartyl-tRNA synthetase [Source:VB Community Annotation] |
| AAEL008906 | | 0.4439 | 3.5522 | 0.1790 | 0.9996 | |
| AAEL009945 | | 1.1134 | 2.8893 | 0.1791 | 0.9996 | |
| AAEL006330 | | -0.4269 | 5.7509 | 0.1791 | 0.9996 | microtubule associated serine/threonine kinase [Source:VB Community Annotation] |

| | | | | | | |
|---|---|---|---|---|---|---|
| AAEL007328 | | -0.4275 | 4.3619 | 0.1792 | 0.9996 | |
| AAEL009623 | | 0.6409 | 3.6658 | 0.1794 | 0.9996 | RNA 3' terminal phosphate cyclase [Source:VB Community Annotation] |
| AAEL002543 | | 0.4129 | 5.1184 | 0.1795 | 0.9996 | |
| AAEL007807 | CYP4D38 | 0.8856 | 2.5727 | 0.1796 | 0.9996 | cytochrome P450 [Source:VB Community Annotation] |
| AAEL019927 | NA | -0.5366 | 3.0228 | 0.1798 | 0.9996 | NA |
| AAEL018195 | | 0.6154 | 2.8969 | 0.1798 | 0.9996 | |
| AAEL014389 | | 0.4054 | 3.5799 | 0.1802 | 0.9996 | |
| AAEL010345 | | 0.4164 | 4.5706 | 0.1803 | 0.9996 | |
| AAEL001570 | | -0.5306 | 4.2472 | 0.1804 | 0.9996 | |
| AAEL021877 | NA | 0.6071 | 3.4139 | 0.1804 | 0.9996 | NA |
| AAEL027646 | NA | 1.3189 | 4.5036 | 0.1805 | 0.9996 | NA |
| AAEL024563 | NA | 0.7467 | 1.7885 | 0.1807 | 0.9996 | NA |
| AAEL006226 | | 0.4021 | 3.8668 | 0.1808 | 0.9996 | sam/hd domain protein [Source:VB Community Annotation] |
| AAEL004577 | | -0.5808 | 1.9674 | 0.1808 | 0.9996 | pmp22 peroxisomal membrane protein, putative [Source:VB Community Annotation] |
| AAEL001622 | | 0.3910 | 3.8418 | 0.1809 | 0.9996 | dual specificity mitogen-activated protein kinase kinase MAPKK [Source:VB Community Annotation] |
| AAEL020816 | NA | -0.9905 | -0.3246 | 0.1810 | 0.9996 | NA |
| AAEL020265 | NA | 0.5308 | 5.8507 | 0.1813 | 0.9996 | NA |
| AAEL002849 | | 0.5799 | 1.6528 | 0.1814 | 0.9996 | zinc finger protein, putative [Source:VB Community Annotation] |
| AAEL010095 | | -0.4971 | 4.8577 | 0.1816 | 0.9996 | |
| AAEL026383 | NA | 0.9415 | 1.2951 | 0.1816 | 0.9996 | NA |
| AAEL018313 | | 0.4204 | 4.8417 | 0.1816 | 0.9996 | |
| AAEL016726 | tRNA-Gly | -1.0379 | -0.1768 | 0.1817 | 0.9996 | |
| AAEL003936 | | -0.8795 | 4.0615 | 0.1817 | 0.9996 | |
| AAEL027801 | NA | 0.5086 | 4.3945 | 0.1819 | 0.9996 | NA |
| AAEL020124 | NA | 1.0932 | -0.1097 | 0.1820 | 0.9996 | NA |
| AAEL023059 | NA | 1.4224 | -0.6057 | 0.1820 | 0.9996 | NA |
| AAEL000211 | | -0.4892 | 8.6801 | 0.1821 | 0.9996 | |
| AAEL009869 | | -0.7096 | 6.0975 | 0.1822 | 0.9996 | low-density lipoprotein receptor (ldl) [Source:VB Community Annotation] |
| AAEL008042 | | 0.4497 | 3.0836 | 0.1823 | 0.9996 | GPI mannosyltransferase 2 (Fragment) [Source:UniProtKB/TrEMBL;Acc:Q16ZW4] |
| AAEL004804 | | -0.4862 | 4.9304 | 0.1824 | 0.9996 | hexaprenyldihydroxybenzoate methyltransferase [Source:VB Community Annotation] |
| AAEL002836 | | 0.5210 | 5.0621 | 0.1825 | 0.9996 | carbon catabolite repressor protein [Source:VB Community Annotation] |
| AAEL006672 | | -0.6016 | 2.3680 | 0.1825 | 0.9996 | |
| AAEL012656 | | 1.4325 | 2.7190 | 0.1826 | 0.9996 | U2 small nuclear ribonucleoprotein a [Source:VB Community Annotation] |
| AAEL023100 | NA | 0.9067 | 2.3753 | 0.1826 | 0.9996 | NA |
| AAEL010691 | | 0.6312 | 2.5660 | 0.1826 | 0.9996 | ribonucleoside-diphosphate reductase small chain [Source:VB Community Annotation] |
| AAEL010050 | | -1.3105 | -0.5633 | 0.1827 | 0.9996 | |
| AAEL015640 | | -0.3252 | 4.5405 | 0.1829 | 0.9996 | transcription factor IIIA, putative [Source:VB Community Annotation] |
| AAEL023209 | NA | -0.4212 | 6.2294 | 0.1831 | 0.9996 | NA |
| AAEL018052 | | 0.8714 | 1.4085 | 0.1833 | 0.9996 | |
| AAEL013798 | CYP4H33 | -1.5112 | -0.8131 | 0.1834 | 0.9996 | cytochrome P450 [Source:VB Community Annotation] |

| | | | | | | |
|---|---|---|---|---|---|---|
| AAEL003233 | | -0.8218 | 1.0506 | 0.1835 | 0.9996 | |
| AAEL001424 | | -0.3748 | 5.6778 | 0.1836 | 0.9996 | mitochondrial inner membrane protease subunit [Source:VB Community Annotation] |
| AAEL021894 | NA | 0.5624 | 6.5860 | 0.1836 | 0.9996 | NA |
| AAEL013201 | | 0.9069 | 0.6745 | 0.1837 | 0.9996 | |
| AAEL006628 | | 0.3401 | 4.6987 | 0.1837 | 0.9996 | |
| AAEL027783 | NA | 1.6757 | -1.6626 | 0.1838 | 0.9996 | NA |
| AAEL023170 | NA | 2.6972 | -2.4471 | 0.1839 | 0.9996 | NA |
| AAEL010565 | | 0.3365 | 5.4958 | 0.1840 | 0.9996 | |
| AAEL005309 | | -0.5304 | 3.9433 | 0.1841 | 0.9996 | |
| AAEL024055 | NA | -0.6569 | 3.1241 | 0.1842 | 0.9996 | NA |
| AAEL023217 | NA | 0.4361 | 3.6565 | 0.1842 | 0.9996 | NA |
| AAEL027214 | NA | -0.9321 | 2.1483 | 0.1842 | 0.9996 | NA |
| AAEL001968 | | 0.4031 | 5.3095 | 0.1843 | 0.9996 | zinc transporter [Source:VB Community Annotation] |
| AAEL028220 | NA | 0.3801 | 5.1726 | 0.1843 | 0.9996 | NA |
| AAEL011473 | | 0.7445 | 2.2486 | 0.1846 | 0.9996 | chromatin regulatory protein sir2 [Source:VB Community Annotation] |
| AAEL017562 | | 0.3859 | 4.9068 | 0.1846 | 0.9996 | |
| AAEL001869 | | -0.9639 | 3.1484 | 0.1846 | 0.9996 | gmp synthase [Source:VB Community Annotation] |
| AAEL012616 | | -0.5813 | 6.4720 | 0.1851 | 0.9996 | NADP transhydrogenase [Source:VB Community Annotation] |
| AAEL014348 | CASPS8 | 0.7984 | 2.2273 | 0.1852 | 0.9996 | caspase (short) [Source:VB Community Annotation] |
| AAEL005022 | Med15 | 0.5565 | 4.5510 | 0.1859 | 0.9996 | Mediator of RNA polymerase II transcription subunit 15 (Med15) [Source:VB Community Annotation] |
| AAEL008052 | | -0.6213 | 4.6078 | 0.1861 | 0.9996 | |
| AAEL012131 | | -0.4484 | 4.4712 | 0.1861 | 0.9996 | cationic amino acid transporter [Source:VB Community Annotation] |
| AAEL021202 | NA | -2.2046 | -0.7733 | 0.1862 | 0.9996 | NA |
| AAEL011736 | | -0.4202 | 6.2388 | 0.1863 | 0.9996 | cyclohex-1-ene-1-carboxyl-CoA hydratase, putative [Source:VB Community Annotation] |
| AAEL008928 | Kir2A | -0.6416 | 6.5854 | 0.1866 | 0.9996 | inward-rectifying potassium channel [Source:VB Community Annotation] |
| AAEL000320 | CYP325T1 | -1.0113 | 0.6502 | 0.1867 | 0.9996 | cytochrome P450 [Source:VB Community Annotation] |
| AAEL023713 | NA | 0.8098 | 0.8722 | 0.1867 | 0.9996 | NA |
| AAEL009793 | | -0.7383 | 0.1365 | 0.1868 | 0.9996 | cuticle protein, putative [Source:VB Community Annotation] |
| AAEL025993 | NA | -0.6741 | 4.0014 | 0.1869 | 0.9996 | NA |
| AAEL003344 | | 0.3225 | 5.5044 | 0.1870 | 0.9996 | metaxin [Source:VB Community Annotation] |
| AAEL021904 | NA | -1.4383 | 0.9034 | 0.1870 | 0.9996 | NA |
| AAEL020428 | NA | -0.8023 | 2.0519 | 0.1871 | 0.9996 | NA |
| AAEL004138 | | -1.6294 | 4.2102 | 0.1872 | 0.9996 | signal peptide peptidase [Source:VB Community Annotation] |
| AAEL007153 | | 0.3781 | 6.6700 | 0.1874 | 0.9996 | 5'-AMP-activated protein kinase, catalytic alpha-1 chain [Source:VB Community Annotation] |
| AAEL002590 | | -0.5770 | 2.8206 | 0.1875 | 0.9996 | serine protease, putative [Source:VB Community Annotation] |
| AAEL010810 | | 0.4546 | 3.1055 | 0.1875 | 0.9996 | |
| AAEL019423 | NA | 0.4120 | 4.5484 | 0.1876 | 0.9996 | NA |
| AAEL013351 | | 1.4722 | -1.0199 | 0.1876 | 0.9996 | lethal(2)essential for life protein, l2efl [Source:VB Community Annotation] |
| AAEL005819 | | 0.5576 | 4.1557 | 0.1879 | 0.9996 | l-allo-threonine aldolase [Source:VB Community Annotation] |
| AAEL007388 | | 0.3797 | 4.6912 | 0.1883 | 0.9996 | |

| | | | | | | |
|---|---|---|---|---|---|---|
| AAEL019777 | NA | -0.6581 | 4.4537 | 0.1884 | 0.9996 | NA |
| AAEL021538 | NA | -1.2052 | 1.7383 | 0.1885 | 0.9996 | NA |
| AAEL011709 | | 0.8890 | 4.2746 | 0.1885 | 0.9996 | diacylglycerol kinase, epsilon [Source:VB Community Annotation] |
| AAEL027434 | NA | -0.3879 | 4.9373 | 0.1886 | 0.9996 | NA |
| AAEL001329 | | 0.4302 | 5.4002 | 0.1886 | 0.9996 | mitochondrial carrier protein [Source:VB Community Annotation] |
| AAEL008244 | | 0.4546 | 3.4230 | 0.1887 | 0.9996 | thyroid hormone receptor-associated protein [Source:VB Community Annotation] |
| AAEL002284 | Med18 | 0.5518 | 2.8942 | 0.1888 | 0.9996 | mediator of RNA polymerase II transcription subunit 18 (Med18) [Source:VB Community Annotation] |
| AAEL013994 | | -0.4592 | 4.6503 | 0.1889 | 0.9996 | |
| AAEL014510 | | -1.0202 | 6.8520 | 0.1889 | 0.9996 | sprouty [Source:VB Community Annotation] |
| AAEL021708 | NA | -1.5417 | -0.1562 | 0.1890 | 0.9996 | NA |
| AAEL007768 | MYD | 1.3012 | 2.2524 | 0.1890 | 0.9996 | TOLL pathway signalling. [Source:VB Community Annotation] |
| AAEL003738 | | 1.0102 | 1.9309 | 0.1892 | 0.9996 | |
| AAEL020341 | NA | -0.3239 | 5.4511 | 0.1892 | 0.9996 | NA |
| AAEL007058 | | 1.6297 | -0.4153 | 0.1892 | 0.9996 | |
| AAEL007010 | CYP6AG4 | -0.5952 | 4.0503 | 0.1893 | 0.9996 | cytochrome P450 [Source:VB Community Annotation] |
| AAEL009852 | | -0.8561 | 7.8857 | 0.1894 | 0.9996 | |
| AAEL003256 | | -1.7016 | -2.3781 | 0.1896 | 0.9996 | |
| AAEL027289 | NA | 0.9797 | 2.3703 | 0.1897 | 0.9996 | NA |
| AAEL010253 | | 1.3721 | -0.2328 | 0.1898 | 0.9996 | |
| AAEL002840 | | 0.4243 | 3.3675 | 0.1898 | 0.9996 | |
| AAEL007816 | CYP4D23 | 0.8004 | 4.3331 | 0.1899 | 0.9996 | cytochrome P450 [Source:VB Community Annotation] |
| AAEL010474 | | -0.4054 | 5.1805 | 0.1900 | 0.9996 | |
| AAEL027190 | NA | 0.4238 | 5.2553 | 0.1903 | 0.9996 | NA |
| AAEL024456 | NA | -0.4993 | 9.6007 | 0.1904 | 0.9996 | NA |
| AAEL021501 | NA | 0.3756 | 3.6826 | 0.1905 | 0.9996 | NA |
| AAEL007443 | | 1.2565 | 1.9200 | 0.1906 | 0.9996 | serine/threonine protein kinase [Source:VB Community Annotation] |
| AAEL005615 | | 1.5770 | -0.2503 | 0.1907 | 0.9996 | |
| AAEL014028 | | 0.4388 | 5.5557 | 0.1907 | 0.9996 | |
| AAEL006755 | | 0.6317 | 4.1467 | 0.1908 | 0.9996 | |
| AAEL024247 | NA | -1.1631 | -0.2977 | 0.1908 | 0.9996 | NA |
| AAEL002423 | | -1.4815 | -0.1243 | 0.1911 | 0.9996 | |
| AAEL014774 | | 0.6473 | 2.2042 | 0.1912 | 0.9996 | |
| AAEL005748 | | -1.0566 | 3.9252 | 0.1915 | 0.9996 | elastase, putative [Source:VB Community Annotation] |
| AAEL001610 | | 0.8766 | 1.0650 | 0.1915 | 0.9996 | |
| AAEL010905 | | 0.4727 | 5.3385 | 0.1916 | 0.9996 | |
| AAEL015526 | | -1.4099 | 0.0710 | 0.1917 | 0.9996 | |
| AAEL009445 | | 0.4160 | 2.8345 | 0.1918 | 0.9996 | |
| AAEL013960 | | 0.5951 | 8.5241 | 0.1920 | 0.9996 | |
| AAEL004855 | | -0.4718 | 12.2986 | 0.1922 | 0.9996 | ADP,ATP carrier protein [Source:VB Community Annotation] |
| AAEL009625 | | -0.8915 | 5.1355 | 0.1925 | 0.9996 | short-chain dehydrogenase [Source:VB Community Annotation] |

| | | | | | | |
|---|---|---|---|---|---|---|
| AAEL021973 | NA | -0.6038 | 3.3719 | 0.1926 | 0.9996 | NA |
| AAEL026088 | NA | 0.6222 | 3.6541 | 0.1926 | 0.9996 | NA |
| AAEL005411 | | 0.6252 | 3.6744 | 0.1927 | 0.9996 | equilibrative nucleoside transporter [Source:VB Community Annotation] |
| AAEL025717 | NA | 0.5441 | 3.2756 | 0.1927 | 0.9996 | NA |
| AAEL010348 | | -0.4840 | 4.0461 | 0.1931 | 0.9996 | sugar transporter [Source:VB Community Annotation] |
| AAEL024736 | NA | 0.6341 | 4.2011 | 0.1932 | 0.9996 | NA |
| AAEL006988 | | 0.3625 | 4.8857 | 0.1932 | 0.9996 | transcription initiation factor TFIID subunit 6 [Source:VB Community Annotation] |
| AAEL011315 | | 0.3980 | 5.3134 | 0.1933 | 0.9996 | |
| AAEL019455 | NA | -0.3763 | 5.8777 | 0.1934 | 0.9996 | NA |
| AAEL012715 | | -0.9391 | 2.7542 | 0.1934 | 0.9996 | |
| AAEL004513 | | 1.0536 | 1.6122 | 0.1935 | 0.9996 | neurotransmitter gated ion channel [Source:VB Community Annotation] |
| AAEL008491 | | 0.4697 | 5.2020 | 0.1937 | 0.9996 | |
| AAEL021190 | NA | 1.2055 | 1.8300 | 0.1938 | 0.9996 | NA |
| AAEL010745 | | -1.7676 | -0.3343 | 0.1942 | 0.9996 | four-jointed protein, putative [Source:VB Community Annotation] |
| AAEL002772 | | 0.4403 | 3.1982 | 0.1943 | 0.9996 | chromo domain protein [Source:VB Community Annotation] |
| AAEL003065 | | 0.2706 | 4.2686 | 0.1944 | 0.9996 | |
| AAEL010533 | | -0.4211 | 4.2058 | 0.1944 | 0.9996 | |
| AAEL019965 | NA | -0.3411 | 5.5275 | 0.1947 | 0.9996 | NA |
| AAEL008341 | | 0.8137 | 6.7754 | 0.1947 | 0.9996 | Adenosylhomocysteinase [Source:UniProtKB/TrEMBL;Acc:Q1HQR0] |
| AAEL005180 | | 0.6281 | 2.6851 | 0.1950 | 0.9996 | |
| AAEL000593 | | -0.4058 | 2.9303 | 0.1953 | 0.9996 | mitochondrial 18 kda protein (mtp18) [Source:VB Community Annotation] |
| AAEL019769 | NA | 0.3853 | 5.0126 | 0.1954 | 0.9996 | NA |
| AAEL002413 | | -0.9563 | 3.5324 | 0.1956 | 0.9996 | Sphingomyelin phosphodiesterase [Source:UniProtKB/TrEMBL;Acc:Q17IB7] |
| AAEL007160 | Ubqn | -0.8942 | 4.3697 | 0.1957 | 0.9996 | ubiquilin [Source:VB Community Annotation] |
| AAEL004350 | | -0.4507 | 6.3090 | 0.1958 | 0.9996 | janus A, putative [Source:VB Community Annotation] |
| AAEL001206 | | 0.8294 | 6.5444 | 0.1959 | 0.9996 | threonyl-tRNA synthetase [Source:VB Community Annotation] |
| AAEL001204 | | 1.8722 | 3.7495 | 0.1959 | 0.9996 | sterol o-acyltransferase [Source:VB Community Annotation] |
| AAEL000943 | | 1.1719 | 1.9306 | 0.1961 | 0.9996 | |
| AAEL005217 | | 0.3929 | 6.2796 | 0.1962 | 0.9996 | membrin [Source:VB Community Annotation] |
| AAEL024939 | NA | -0.7697 | 1.1804 | 0.1963 | 0.9996 | NA |
| AAEL002813 | | -0.6284 | 8.8233 | 0.1963 | 0.9996 | coupling factor, putative [Source:VB Community Annotation] |
| AAEL010529 | | 0.9210 | 3.9461 | 0.1967 | 0.9996 | |
| AAEL004859 | | -0.5730 | 7.9124 | 0.1968 | 0.9996 | ATP-dependent RNA helicase [Source:VB Community Annotation] |
| AAEL000780 | | 0.3517 | 3.8684 | 0.1969 | 0.9996 | amino acid transporter [Source:VB Community Annotation] |
| AAEL010062 | | -0.4114 | 3.8949 | 0.1969 | 0.9996 | |
| AAEL000981 | | 0.9386 | 2.2760 | 0.1969 | 0.9996 | |
| AAEL000051 | OBP3 | 0.5236 | 5.7931 | 0.1970 | 0.9996 | odorant binding protein OBP3 [Source:VB Community Annotation] |
| AAEL001696 | | -1.0156 | 1.2578 | 0.1970 | 0.9996 | paired box protein, putative [Source:VB Community Annotation] |
| AAEL012740 | | -0.5061 | 12.1118 | 0.1973 | 0.9996 | ATPase subunit, putative [Source:VB Community Annotation] |
| AAEL005176 | | 1.2180 | 2.8099 | 0.1974 | 0.9996 | |

| | | | | | | |
|---|---|---|---|---|---|---|
| AAEL016063 | tRNA-Arg | -0.7349 | 0.9675 | 0.1976 | 0.9996 | |
| AAEL014130 | | 0.7981 | 4.7691 | 0.1976 | 0.9996 | |
| AAEL011901 | | -0.5636 | 4.9801 | 0.1979 | 0.9996 | 1-acyl-sn-glycerol-3-phosphate acyltransferase [Source:VB Community Annotation] |
| AAEL018132 | | 0.7702 | 3.4696 | 0.1980 | 0.9996 | |
| AAEL002248 | | -0.5284 | 4.1213 | 0.1980 | 0.9996 | |
| AAEL012828 | | -1.1794 | 0.6705 | 0.1981 | 0.9996 | |
| AAEL003360 | | 0.5877 | 3.8081 | 0.1982 | 0.9996 | |
| AAEL025888 | NA | 0.4718 | 6.4069 | 0.1984 | 0.9996 | NA |
| AAEL013392 | | 0.5156 | 3.8712 | 0.1985 | 0.9996 | Phosphatidate cytidylyltransferase, mitochondrial [Source:UniProtKB/TrEMBL;Acc:Q16J98] |
| AAEL010722 | | 0.9763 | 1.9267 | 0.1985 | 0.9996 | |
| AAEL027368 | NA | -1.4803 | -1.0204 | 0.1986 | 0.9996 | NA |
| AAEL022003 | NA | -0.7928 | 4.5455 | 0.1986 | 0.9996 | NA |
| AAEL017059 | | -0.4293 | 5.3601 | 0.1986 | 0.9996 | |
| AAEL000348 | | -0.6390 | 4.7586 | 0.1986 | 0.9996 | |
| AAEL009458 | | -0.4439 | 3.1291 | 0.1987 | 0.9996 | syntaxin 4 [Source:VB Community Annotation] |
| AAEL024147 | NA | -0.7582 | 6.5648 | 0.1988 | 0.9996 | NA |
| AAEL020881 | NA | 0.6681 | 1.2771 | 0.1989 | 0.9996 | NA |
| AAEL007777 | | -0.4091 | 7.2996 | 0.1990 | 0.9996 | vacuolar ATP synthase subunit S1 [Source:VB Community Annotation] |
| AAEL027777 | NA | -1.2095 | -0.8917 | 0.1990 | 0.9996 | NA |
| AAEL012383 | | -0.7202 | 2.7696 | 0.1990 | 0.9996 | protein serine/threonine kinase, putative [Source:VB Community Annotation] |
| AAEL009776 | | -0.5052 | 3.2158 | 0.1990 | 0.9996 | |
| AAEL022253 | NA | 1.5865 | -1.3988 | 0.1990 | 0.9996 | NA |
| AAEL004995 | | 0.5170 | 3.8584 | 0.1990 | 0.9996 | caspase-activated nuclease, putative [Source:VB Community Annotation] |
| AAEL005419 | twf | 0.4996 | 4.4772 | 0.1991 | 0.9996 | Twinfilin [Source:UniProtKB/Swiss-Prot;Acc:Q17A58] |
| AAEL008963 | | 2.1493 | 5.6105 | 0.1993 | 0.9996 | tyrosine aminotransferase [Source:VB Community Annotation] |
| AAEL005460 | | -0.4927 | 6.3843 | 0.1993 | 0.9996 | alpha-galactosidase/alpha-n-acetylgalactosaminidase [Source:VB Community Annotation] |
| AAEL006946 | | -0.3683 | 6.7649 | 0.1993 | 0.9996 | chaperonin [Source:VB Community Annotation] |
| AAEL000590 | DNR1 | -0.3148 | 5.3987 | 0.1993 | 0.9996 | Defense repressor 1 [Source:VB Community Annotation] |
| AAEL011811 | | 1.0955 | 0.7535 | 0.1994 | 0.9996 | DNA replication licensing factor MCM3 [Source:VB Community Annotation] |
| AAEL023262 | NA | -0.9658 | 0.5285 | 0.1995 | 0.9996 | NA |
| AAEL019789 | NA | -0.4190 | 10.8409 | 0.1998 | 0.9996 | NA |
| AAEL001381 | | 1.3793 | 1.6288 | 0.1999 | 0.9996 | sphingomyelin synthetase [Source:VB Community Annotation] |
| AAEL002493 | | -0.7134 | 3.2030 | 0.2001 | 0.9996 | short-chain dehydrogenase [Source:VB Community Annotation] |
| AAEL021732 | NA | 0.6968 | 3.4070 | 0.2001 | 0.9996 | NA |
| AAEL003154 | | -0.4180 | 5.2963 | 0.2003 | 0.9996 | aldo-keto reductase [Source:VB Community Annotation] |
| AAEL023750 | NA | -1.1329 | 1.1743 | 0.2003 | 0.9996 | NA |
| AAEL011147 | | 0.4824 | 4.8477 | 0.2006 | 0.9996 | |
| AAEL011697 | | 0.4069 | 4.4567 | 0.2007 | 0.9996 | glycerol kinase [Source:VB Community Annotation] |
| AAEL007800 | | -1.5337 | 2.6458 | 0.2007 | 0.9996 | cytidine and deoxycytidylate deaminase zinc-binding region [Source:VB Community Annotation] |
| AAEL024195 | NA | -0.4076 | 5.8815 | 0.2008 | 0.9996 | NA |

| | | | | | | |
|---|---|---|---|---|---|---|
| AAEL006449 | | 0.4226 | 5.6472 | 0.2008 | 0.9996 | ser/thr protein kinase-lyk4 [Source:VB Community Annotation] |
| AAEL024670 | NA | 0.3953 | 4.3947 | 0.2010 | 0.9996 | NA |
| AAEL003576 | | -0.8504 | 1.7148 | 0.2011 | 0.9996 | |
| AAEL013461 | | 0.4699 | 4.2252 | 0.2012 | 0.9996 | parvin [Source:VB Community Annotation] |
| AAEL012518 | | 1.6679 | 0.4813 | 0.2013 | 0.9996 | |
| AAEL018280 | | -0.4277 | 7.2243 | 0.2014 | 0.9996 | |
| AAEL003161 | | 0.4068 | 5.2859 | 0.2018 | 0.9996 | Adenylosuccinate synthetase [Source:UniProtKB/Swiss-Prot;Acc:Q17G75] |
| AAEL014069 | | -1.0954 | 1.4881 | 0.2018 | 0.9996 | catrin, putative [Source:VB Community Annotation] |
| AAEL009677 | | 0.8716 | 4.8564 | 0.2018 | 0.9996 | membrane-associated protein gex-3 [Source:VB Community Annotation] |
| AAEL026296 | NA | -1.0579 | 0.2047 | 0.2020 | 0.9996 | NA |
| AAEL001495 | | -0.5244 | 2.9817 | 0.2020 | 0.9996 | |
| AAEL000042 | | -0.3934 | 4.5637 | 0.2021 | 0.9996 | |
| AAEL005215 | | 0.4299 | 3.8584 | 0.2022 | 0.9996 | |
| AAEL025916 | NA | -0.8698 | 1.0819 | 0.2022 | 0.9996 | NA |
| AAEL007250 | comm2 | 0.7460 | 1.8336 | 0.2023 | 0.9996 | Protein commissureless 2 homolog [Source:UniProtKB/Swiss-Prot;Acc:Q172W0] |
| AAEL005020 | | 0.4199 | 3.3574 | 0.2024 | 0.9996 | |
| AAEL005229 | | 0.5098 | 4.0377 | 0.2026 | 0.9996 | ptpla domain protein [Source:VB Community Annotation] |
| AAEL017161 | | -0.4678 | 4.1900 | 0.2028 | 0.9996 | |
| AAEL021200 | NA | -1.0599 | 3.0026 | 0.2028 | 0.9996 | NA |
| AAEL020434 | NA | 0.3963 | 5.7608 | 0.2030 | 0.9996 | NA |
| AAEL021559 | NA | 0.5977 | 3.4383 | 0.2032 | 0.9996 | NA |
| AAEL003973 | | 0.3668 | 4.7859 | 0.2033 | 0.9996 | |
| AAEL007848 | | 0.6373 | 5.1331 | 0.2034 | 0.9996 | |
| AAEL025505 | NA | -1.3467 | -0.6081 | 0.2035 | 0.9996 | NA |
| AAEL000545 | | 0.4793 | 4.9790 | 0.2035 | 0.9996 | |
| AAEL001680 | | 0.8505 | 2.8115 | 0.2035 | 0.9996 | |
| AAEL002165 | | -0.6230 | 4.4905 | 0.2037 | 0.9996 | Hydroxypyruvate isomerase [Source:UniProtKB/TrEMBL;Acc:Q17J21] |
| AAEL024669 | NA | -0.8224 | 7.3407 | 0.2038 | 0.9996 | NA |
| AAEL022891 | NA | -0.5811 | 3.2615 | 0.2039 | 0.9996 | NA |
| AAEL010485 | | -0.4341 | 4.2224 | 0.2039 | 0.9996 | sugar transporter [Source:VB Community Annotation] |
| AAEL005939 | | -0.4443 | 3.8490 | 0.2040 | 0.9996 | mpv17 protein [Source:VB Community Annotation] |
| AAEL019554 | NA | -0.6798 | 5.6431 | 0.2041 | 0.9996 | NA |
| AAEL028866 | NA | -0.9442 | 0.9004 | 0.2042 | 0.9996 | NA |
| AAEL013116 | mRpL3 | -0.5819 | 5.3027 | 0.2042 | 0.9996 | mitochondrial 39S ribosomal protein L3 [Source:VB Community Annotation] |
| AAEL013463 | | -0.4613 | 4.1513 | 0.2043 | 0.9996 | nucleolar protein 10 [Source:VB Community Annotation] |
| AAEL003413 | | -0.6303 | 6.2724 | 0.2044 | 0.9996 | F-spondin [Source:VB Community Annotation] |
| AAEL005911 | | 0.8589 | 3.7805 | 0.2045 | 0.9996 | mitochondrial carrier protein [Source:VB Community Annotation] |
| AAEL004960 | | 0.6129 | 1.3139 | 0.2045 | 0.9996 | |
| AAEL018257 | | 0.4051 | 3.1193 | 0.2047 | 0.9996 | |
| AAEL001062 | | 0.6720 | 2.7103 | 0.2049 | 0.9996 | |

| | | | | | | |
|---|---|---|---|---|---|---|
| AAEL004335 | | -1.1818 | 1.3924 | 0.2049 | 0.9996 | secreted ferritin G subunit precursor, putative [Source:VB Community Annotation] |
| AAEL027193 | NA | 0.5800 | 5.6269 | 0.2049 | 0.9996 | NA |
| AAEL003497 | | 0.6539 | 6.0640 | 0.2049 | 0.9996 | farnesyl-pyrophosphate synthetase [Source:VB Community Annotation] |
| AAEL008181 | | 0.4231 | 4.4212 | 0.2050 | 0.9996 | alpha6-fucosyltransferase, putative [Source:VB Community Annotation] |
| AAEL021410 | NA | 1.3096 | -0.4593 | 0.2050 | 0.9996 | NA |
| AAEL014125 | | -1.6043 | -0.7915 | 0.2051 | 0.9996 | nk homeobox protein [Source:VB Community Annotation] |
| AAEL002399 | | 0.4061 | 6.6560 | 0.2053 | 0.9996 | Aspartate aminotransferase [Source:UniProtKB/TrEMBL;Acc:Q5K6H3] |
| AAEL025190 | NA | 0.4387 | 3.8010 | 0.2054 | 0.9996 | NA |
| AAEL018695 | | -0.4043 | 4.3562 | 0.2054 | 0.9996 | |
| AAEL021491 | NA | 0.8473 | 3.2402 | 0.2055 | 0.9996 | NA |
| AAEL009158 | | -0.6135 | 3.0860 | 0.2056 | 0.9996 | |
| AAEL000329 | | -1.1473 | 0.0910 | 0.2058 | 0.9996 | |
| AAEL025815 | NA | 0.4070 | 3.7900 | 0.2058 | 0.9996 | NA |
| AAEL010012 | sar1 | 0.3976 | 7.7828 | 0.2060 | 0.9996 | GTP-binding protein sar1 [Source:VB Community Annotation] |
| AAEL003700 | | 0.5857 | 2.6568 | 0.2060 | 0.9996 | |
| AAEL011483 | | -1.7351 | -2.0369 | 0.2062 | 0.9996 | odorant-binding protein 50c, putative [Source:VB Community Annotation] |
| AAEL019923 | NA | -1.2512 | 1.1168 | 0.2064 | 0.9996 | NA |
| AAEL006762 | | -0.3816 | 4.8168 | 0.2065 | 0.9996 | |
| AAEL002084 | | 0.3851 | 4.4133 | 0.2067 | 0.9996 | suppressor of defective silencing [Source:VB Community Annotation] |
| AAEL002425 | | -0.7317 | 2.3087 | 0.2069 | 0.9996 | ADP ribosylation factor, putative [Source:VB Community Annotation] |
| AAEL014992 | | 0.3691 | 4.3841 | 0.2071 | 0.9996 | rab gdp/GTP exchange factor [Source:VB Community Annotation] |
| AAEL012437 | | -1.4622 | 3.2613 | 0.2073 | 0.9996 | |
| AAEL001094 | | -0.6549 | 6.7170 | 0.2073 | 0.9996 | |
| AAEL004168 | | 1.1242 | 4.4273 | 0.2075 | 0.9996 | syntaxin [Source:VB Community Annotation] |
| AAEL006113 | | 0.3994 | 4.3356 | 0.2076 | 0.9996 | cystinosin [Source:VB Community Annotation] |
| AAEL015567 | OBP63 | -0.4496 | 10.1116 | 0.2078 | 0.9996 | odorant binding protein OBP63 [Source:VB Community Annotation] |
| AAEL012037 | | -0.7716 | 1.8504 | 0.2078 | 0.9996 | sulphate transporter [Source:VB Community Annotation] |
| AAEL017421 | | -0.5493 | 6.0637 | 0.2079 | 0.9996 | |
| AAEL007806 | | 0.3673 | 7.0679 | 0.2080 | 0.9996 | |
| AAEL023323 | NA | 0.4810 | 5.6801 | 0.2082 | 0.9996 | NA |
| AAEL006809 | | 0.4148 | 5.1723 | 0.2083 | 0.9996 | voltage-gated ion channel [Source:VB Community Annotation] |
| AAEL003919 | | 1.3030 | 4.6064 | 0.2083 | 0.9996 | cationic amino acid transporter [Source:VB Community Annotation] |
| AAEL003228 | | 0.4158 | 4.3132 | 0.2083 | 0.9996 | mitotic protein phosphatase 1 regulator, putative [Source:VB Community Annotation] |
| AAEL000802 | | 0.5821 | 4.8876 | 0.2084 | 0.9996 | |
| AAEL001646 | Kir3 | 1.7747 | -1.0804 | 0.2085 | 0.9996 | inward-rectifying potassium channel [Source:VB Community Annotation] |
| AAEL017371 | | -0.4103 | 4.5508 | 0.2087 | 0.9996 | |
| AAEL010812 | | 0.3894 | 3.2406 | 0.2087 | 0.9996 | zinc finger protein [Source:VB Community Annotation] |
| AAEL011254 | | 0.4204 | 3.9331 | 0.2089 | 0.9996 | |
| AAEL013570 | | -1.4470 | -2.3077 | 0.2090 | 0.9996 | |
| AAEL002316 | | 0.7963 | 1.4377 | 0.2093 | 0.9996 | |

| | | | | | | |
|---|---|---|---|---|---|---|
| AAEL004941 | CYP6AK1 | -0.6328 | 4.4115 | 0.2095 | 0.9996 | cytochrome P450 [Source:VB Community Annotation] |
| AAEL026310 | NA | 0.3965 | 5.6632 | 0.2095 | 0.9996 | NA |
| AAEL008738 | | -0.4276 | 7.8532 | 0.2096 | 0.9996 | DEAD box ATP-dependent RNA helicase [Source:VB Community Annotation] |
| AAEL028221 | NA | -0.4841 | 2.0625 | 0.2097 | 0.9996 | NA |
| AAEL005735 | | 0.3548 | 3.8265 | 0.2097 | 0.9996 | |
| AAEL004057 | | 0.5238 | 3.1172 | 0.2099 | 0.9996 | |
| AAEL023958 | NA | -0.6726 | 4.8832 | 0.2099 | 0.9996 | NA |
| AAEL003666 | | 0.4305 | 5.3101 | 0.2101 | 0.9996 | leukotriene a-4 hydrolase [Source:VB Community Annotation] |
| AAEL007224 | LRIM22 | -0.3576 | 7.1077 | 0.2101 | 0.9996 | leucine-rich immune protein (Coil-less) [Source:VB Community Annotation] |
| AAEL003139 | | -0.5038 | 4.0961 | 0.2101 | 0.9996 | short-chain dehydrogenase [Source:VB Community Annotation] |
| AAEL011326 | | 1.2462 | -0.0690 | 0.2101 | 0.9996 | |
| AAEL002065 | | -0.5660 | 4.1980 | 0.2102 | 0.9996 | hydroxymethylglutaryl-coa lyase [Source:VB Community Annotation] |
| AAEL011946 | | -0.5963 | 1.9763 | 0.2104 | 0.9996 | |
| AAEL006955 | | 1.3840 | 0.2726 | 0.2104 | 0.9996 | cadherin [Source:VB Community Annotation] |
| AAEL022833 | NA | -1.9668 | -1.5323 | 0.2105 | 0.9996 | NA |
| AAEL009204 | | 0.7256 | 2.5646 | 0.2105 | 0.9996 | glucose dehydrogenase [Source:VB Community Annotation] |
| AAEL025584 | NA | -0.6028 | 2.5838 | 0.2107 | 0.9996 | NA |
| AAEL003008 | | 0.5598 | 3.3254 | 0.2108 | 0.9996 | |
| AAEL014055 | | 0.4266 | 5.7440 | 0.2108 | 0.9996 | thymidine kinase [Source:VB Community Annotation] |
| AAEL025223 | NA | 0.4059 | 4.1064 | 0.2109 | 0.9996 | NA |
| AAEL020322 | NA | 0.5245 | 3.5395 | 0.2110 | 0.9996 | NA |
| AAEL008032 | | 0.7819 | 1.7490 | 0.2110 | 0.9996 | |
| AAEL000013 | | 0.3538 | 3.3982 | 0.2112 | 0.9996 | peptidyl-prolyl cis-trans isomerase (cyclophilin) [Source:VB Community Annotation] |
| AAEL019544 | NA | -0.4975 | 6.4709 | 0.2113 | 0.9996 | NA |
| AAEL011387 | | 0.3200 | 4.5368 | 0.2114 | 0.9996 | leucine-rich repeat [Source:VB Community Annotation] |
| AAEL027236 | NA | -1.4565 | 1.3699 | 0.2114 | 0.9996 | NA |
| AAEL013525 | | -0.4157 | 5.5543 | 0.2114 | 0.9996 | Timp-3, putative [Source:VB Community Annotation] |
| AAEL001354 | | -0.2523 | 6.9230 | 0.2115 | 0.9996 | |
| AAEL000077 | | 0.4002 | 4.6073 | 0.2117 | 0.9996 | cation efflux protein/ zinc transporter [Source:VB Community Annotation] |
| AAEL025570 | NA | 0.6295 | 3.7454 | 0.2118 | 0.9996 | NA |
| AAEL007934 | | 1.0051 | 1.8840 | 0.2123 | 0.9996 | tRNA (5-methylaminomethyl-2-thiouridylate)-methyltransferase [Source:VB Community Annotation] |
| AAEL021592 | NA | 0.9544 | -0.5164 | 0.2126 | 0.9996 | NA |
| AAEL000863 | | -1.9094 | -0.5436 | 0.2127 | 0.9996 | SPASIC protein, putative [Source:VB Community Annotation] |
| AAEL024039 | NA | -1.3563 | 0.4501 | 0.2127 | 0.9996 | NA |
| AAEL011278 | | -0.4694 | 5.9299 | 0.2129 | 0.9996 | apolipoprotein D, putative [Source:VB Community Annotation] |
| AAEL026418 | NA | 1.9605 | -0.3964 | 0.2134 | 0.9996 | NA |
| AAEL007260 | | 0.6407 | 3.3401 | 0.2135 | 0.9996 | |
| AAEL011593 | CLIPC11 | -1.3782 | 0.7196 | 0.2135 | 0.9996 | Clip-Domain Serine Protease family C. [Source:VB Community Annotation] |
| AAEL025249 | NA | 1.1006 | 0.9489 | 0.2135 | 0.9996 | NA |
| AAEL007548 | | 0.3387 | 5.8338 | 0.2136 | 0.9996 | leukocyte receptor cluster (lrc) member [Source:VB Community Annotation] |

| | | | | | | |
|---|---|---|---|---|---|---|
| AAEL003090 | | 0.6749 | 3.8993 | 0.2136 | 0.9996 | malate synthase [Source:VB Community Annotation] |
| AAEL002155 | | -0.3524 | 5.2773 | 0.2142 | 0.9996 | ras-related protein Rab-10, putative [Source:VB Community Annotation] |
| AAEL019784 | NA | -0.4613 | 5.8733 | 0.2143 | 0.9996 | NA |
| AAEL005897 | | -0.3858 | 7.5789 | 0.2145 | 0.9996 | MICOS complex subunit [Source:UniProtKB/TrEMBL;Acc:Q0IFC3] |
| AAEL010768 | | 0.4076 | 3.4273 | 0.2146 | 0.9996 | |
| AAEL006240 | | -0.3762 | 7.5074 | 0.2146 | 0.9996 | purple acid phosphatase, putative [Source:VB Community Annotation] |
| AAEL025833 | NA | -0.7478 | 0.4146 | 0.2147 | 0.9996 | NA |
| AAEL011857 | | -0.5926 | 3.1720 | 0.2147 | 0.9996 | serine/threonine-protein kinase rio1 (rio kinase 1) [Source:VB Community Annotation] |
| AAEL006251 | | -0.3300 | 7.1184 | 0.2148 | 0.9996 | Ubiquitin-fold modifier 1 Precursor [Source:VB Community Annotation] |
| AAEL001805 | | 0.5660 | 2.7694 | 0.2149 | 0.9996 | |
| AAEL009974 | | -2.0335 | -0.7936 | 0.2150 | 0.9996 | ras-related protein Rab-8A, putative [Source:VB Community Annotation] |
| AAEL022190 | NA | 0.5226 | 5.8134 | 0.2150 | 0.9996 | NA |
| AAEL005590 | | -1.3386 | -1.1001 | 0.2151 | 0.9996 | autotransporter adhesin precursor, putative [Source:VB Community Annotation] |
| AAEL001255 | | 0.4602 | 4.5109 | 0.2152 | 0.9996 | |
| AAEL022578 | NA | -0.7107 | 7.4740 | 0.2152 | 0.9996 | NA |
| AAEL014772 | | 1.0098 | 0.8141 | 0.2152 | 0.9996 | |
| AAEL005162 | mRpL11 | -0.4282 | 6.3767 | 0.2152 | 0.9996 | 39S ribosomal protein L11, mitochondrial [Source:VB Community Annotation] |
| AAEL011753 | | -0.6266 | 2.5759 | 0.2152 | 0.9996 | |
| AAEL004564 | | -1.3971 | 1.5829 | 0.2153 | 0.9996 | |
| AAEL006551 | | -0.5655 | 5.2597 | 0.2158 | 0.9996 | |
| AAEL019509 | NA | 0.3256 | 6.4308 | 0.2160 | 0.9996 | NA |
| AAEL009614 | | 0.3517 | 4.6580 | 0.2164 | 0.9996 | seven in absentia, putative [Source:VB Community Annotation] |
| AAEL022661 | NA | -0.3541 | 6.4208 | 0.2165 | 0.9996 | NA |
| AAEL009480 | | 0.3283 | 5.2789 | 0.2166 | 0.9996 | |
| AAEL010476 | | 0.4506 | 5.1593 | 0.2166 | 0.9996 | sil1 [Source:VB Community Annotation] |
| AAEL021782 | NA | -0.4272 | 2.9233 | 0.2166 | 0.9996 | NA |
| AAEL004415 | | 1.1136 | 3.5474 | 0.2166 | 0.9996 | fuse-binding protein-interacting repressor siahbp1 [Source:VB Community Annotation] |
| AAEL006656 | | 0.2878 | 4.1930 | 0.2167 | 0.9996 | Anoctamin [Source:UniProtKB/TrEMBL;Acc:A0A1S4FED6] |
| AAEL004814 | | -0.9611 | 0.9072 | 0.2168 | 0.9996 | potassium-dependent sodium-calcium exchanger, putative [Source:VB Community Annotation] |
| AAEL002788 | | 0.5480 | 3.1543 | 0.2168 | 0.9996 | |
| AAEL021489 | NA | 1.0223 | 1.6814 | 0.2169 | 0.9996 | NA |
| AAEL003528 | | 0.4715 | 3.6973 | 0.2170 | 0.9996 | cdc73 domain protein [Source:VB Community Annotation] |
| AAEL010780 | | -1.5886 | -1.3537 | 0.2171 | 0.9996 | carboxypeptidase [Source:VB Community Annotation] |
| AAEL018155 | | 0.6172 | 2.2488 | 0.2172 | 0.9996 | |
| AAEL007694 | | 1.5053 | 1.6481 | 0.2172 | 0.9996 | |
| AAEL011103 | | 0.3574 | 3.9100 | 0.2172 | 0.9996 | centromere/kinetochore protein zw10 [Source:VB Community Annotation] |
| AAEL008418 | | 0.4047 | 4.3547 | 0.2173 | 0.9996 | Pyrroline-5-carboxylate reductase [Source:UniProtKB/TrEMBL;Acc:Q16YV4] |
| AAEL009392 | | 0.5644 | 4.2341 | 0.2177 | 0.9996 | chromosome region maintenance protein 5/exportin [Source:VB Community Annotation] |
| AAEL017319 | | 0.3916 | 6.0416 | 0.2179 | 0.9996 | |
| AAEL020918 | NA | 1.1621 | -0.4399 | 0.2180 | 0.9996 | NA |

| | | | | | | |
|---|---|---|---|---|---|---|
| AAEL007944 | | 0.4967 | 5.2523 | 0.2182 | 0.9996 | |
| AAEL020690 | NA | -0.5732 | 7.6648 | 0.2182 | 0.9996 | NA |
| AAEL014246 | | -0.5815 | 9.7840 | 0.2183 | 0.9996 | glucosyl/glucuronosyl transferases [Source:VB Community Annotation] |
| AAEL003216 | | -1.2141 | 4.4829 | 0.2184 | 0.9996 | |
| AAEL002467 | | 2.0829 | -1.2386 | 0.2184 | 0.9996 | |
| AAEL011096 | | 0.7380 | 4.2899 | 0.2184 | 0.9996 | viral IAP-associated factor, putative [Source:VB Community Annotation] |
| AAEL025915 | NA | -0.4073 | 4.1841 | 0.2186 | 0.9996 | NA |
| AAEL002303 | | 0.9758 | 0.4469 | 0.2187 | 0.9996 | |
| AAEL007532 | | 1.3986 | 2.1210 | 0.2187 | 0.9996 | |
| AAEL000001 | | -0.4142 | 5.5002 | 0.2189 | 0.9996 | |
| AAEL009663 | | 0.5515 | 2.6923 | 0.2190 | 0.9996 | |
| AAEL011423 | | 0.9019 | 1.1565 | 0.2193 | 0.9996 | sugar transporter [Source:VB Community Annotation] |
| AAEL009082 | | -0.4173 | 4.5375 | 0.2194 | 0.9996 | |
| AAEL008507 | | -0.6629 | 5.7175 | 0.2196 | 0.9996 | srpk [Source:VB Community Annotation] |
| AAEL004911 | | 0.5757 | 3.4867 | 0.2196 | 0.9996 | DEAD box ATP-dependent RNA helicase [Source:VB Community Annotation] |
| AAEL001048 | | -0.3876 | 5.4870 | 0.2196 | 0.9996 | short-chain dehydrogenase [Source:VB Community Annotation] |
| AAEL022559 | NA | -0.4086 | 5.0954 | 0.2199 | 0.9996 | NA |
| AAEL000701 | RpL4 | 0.3838 | 6.2016 | 0.2200 | 0.9996 | 60S ribosomal protein L4 [Source:VB Community Annotation] |
| AAEL023929 | NA | -1.7198 | -0.1643 | 0.2201 | 0.9996 | NA |
| AAEL017132 | LYSC4 | -0.6831 | 7.4241 | 0.2202 | 0.9996 | C-Type Lysozyme (Lys-C). [Source:VB Community Annotation] |
| AAEL011554 | | 0.2942 | 5.7995 | 0.2204 | 0.9996 | adhesion regulating molecule 1 (110 kda cell membrane glycoprotein) [Source:VB Community Annotation] |
| AAEL027551 | NA | 0.5203 | 4.0569 | 0.2204 | 0.9996 | NA |
| AAEL022538 | NA | 0.3902 | 5.8287 | 0.2205 | 0.9996 | NA |
| AAEL002844 | | 0.3718 | 4.4242 | 0.2206 | 0.9996 | |
| AAEL007982 | | 1.0696 | 2.4276 | 0.2209 | 0.9996 | glycosyltransferase [Source:VB Community Annotation] |
| AAEL021941 | NA | 0.3432 | 5.3140 | 0.2210 | 0.9996 | NA |
| AAEL022986 | NA | 1.2191 | 0.0893 | 0.2210 | 0.9996 | NA |
| AAEL007564 | | 0.9295 | 0.4914 | 0.2212 | 0.9996 | zinc finger protein [Source:VB Community Annotation] |
| AAEL024977 | NA | -0.7022 | 9.4428 | 0.2216 | 0.9996 | NA |
| AAEL013996 | | 0.5550 | 2.6691 | 0.2217 | 0.9996 | |
| AAEL011868 | | 0.4692 | 2.8388 | 0.2217 | 0.9996 | |
| AAEL001851 | | -0.4822 | 5.4083 | 0.2218 | 0.9996 | |
| AAEL008876 | | -1.5492 | 1.2527 | 0.2219 | 0.9996 | deoxyribonuclease I, putative [Source:VB Community Annotation] |
| AAEL002257 | | 1.0943 | 0.1618 | 0.2219 | 0.9996 | |
| AAEL002815 | | -1.5459 | -1.6227 | 0.2220 | 0.9996 | |
| AAEL021971 | NA | -0.7564 | 2.3320 | 0.2220 | 0.9996 | NA |
| AAEL011665 | | 0.4088 | 7.0216 | 0.2222 | 0.9996 | |
| AAEL004229 | GSTT4 | -0.5325 | 6.6562 | 0.2223 | 0.9996 | glutathione transferase [Source:VB Community Annotation] |
| AAEL002463 | | -0.3587 | 5.4333 | 0.2223 | 0.9996 | |
| AAEL001912 | | 1.7685 | 1.6372 | 0.2225 | 0.9996 | forkhead protein/ forkhead protein domain [Source:VB Community Annotation] |

| | | | | | | |
|---|---|---|---|---|---|---|
| AAEL016195 | tRNA-Trp | -0.4482 | 4.6688 | 0.2226 | 0.9996 | |
| AAEL026904 | NA | 0.4109 | 11.0813 | 0.2226 | 0.9996 | NA |
| AAEL003176 | | 1.0034 | -0.0212 | 0.2229 | 0.9996 | |
| AAEL013051 | | 0.6444 | 1.2335 | 0.2231 | 0.9996 | |
| AAEL019640 | NA | 0.3449 | 5.8143 | 0.2234 | 0.9996 | NA |
| AAEL011038 | | -0.5873 | 2.3383 | 0.2234 | 0.9996 | integrin alpha-ps [Source:VB Community Annotation] |
| AAEL007493 | | 0.5085 | 6.8521 | 0.2235 | 0.9996 | |
| AAEL005898 | | 0.6666 | 3.9970 | 0.2235 | 0.9996 | tRNA-dihydrouridine synthase [Source:VB Community Annotation] |
| AAEL011295 | | 0.7375 | 4.9998 | 0.2235 | 0.9996 | |
| AAEL025330 | NA | -1.6959 | -1.1461 | 0.2236 | 0.9996 | NA |
| AAEL010143 | | 0.4045 | 6.6413 | 0.2237 | 0.9996 | Isocitrate dehydrogenase [NAD] subunit, mitochondrial [Source:UniProtKB/TrEMBL;Acc:Q16TS5] |
| AAEL011239 | | 0.3811 | 5.3138 | 0.2237 | 0.9996 | short-chain dehydrogenase [Source:VB Community Annotation] |
| AAEL011187 | | -0.7803 | 2.6787 | 0.2240 | 0.9996 | U520 [Source:VB Community Annotation] |
| AAEL026449 | NA | 1.2900 | 0.0385 | 0.2241 | 0.9996 | NA |
| AAEL001782 | GPRNND2 | 1.1669 | 4.4921 | 0.2241 | 0.9996 | GPCR Orphan/Putative Class D Family [Source:VB Community Annotation] |
| AAEL003942 | RpL44 | -0.3054 | 11.2725 | 0.2241 | 0.9996 | 60S ribosomal protein L44 L41, putative [Source:VB Community Annotation] |
| AAEL021865 | NA | 0.7743 | 0.9536 | 0.2243 | 0.9996 | NA |
| AAEL006855 | | 0.4396 | 6.2539 | 0.2244 | 0.9996 | UDP-galactose transporter [Source:VB Community Annotation] |
| AAEL011470 | | 0.5026 | 4.8408 | 0.2246 | 0.9996 | cis,cis-muconate transport protein MucK, putative [Source:VB Community Annotation] |
| AAEL001810 | | 0.4632 | 5.1805 | 0.2246 | 0.9996 | |
| AAEL009861 | | -0.5473 | 6.7825 | 0.2246 | 0.9996 | |
| AAEL021886 | NA | -0.3849 | 7.7930 | 0.2246 | 0.9996 | NA |
| AAEL014375 | | 0.3397 | 5.7884 | 0.2248 | 0.9996 | clathrin coat assembly protein ap17 [Source:VB Community Annotation] |
| AAEL004083 | | 0.3986 | 4.5906 | 0.2248 | 0.9996 | WD-repeat protein [Source:VB Community Annotation] |
| AAEL015038 | | -0.5571 | 5.7480 | 0.2250 | 0.9996 | palmitoyl-protein thioesterase [Source:VB Community Annotation] |
| AAEL008148 | | -1.4150 | -1.2278 | 0.2251 | 0.9996 | 8-oxoguanine DNA glycosylase [Source:VB Community Annotation] |
| AAEL009862 | | -1.4119 | -0.4048 | 0.2251 | 0.9996 | |
| AAEL000282 | | -0.7549 | 1.0796 | 0.2252 | 0.9996 | syntaxin, putative [Source:VB Community Annotation] |
| AAEL008067 | | 0.4294 | 4.4907 | 0.2253 | 0.9996 | |
| AAEL025509 | NA | -0.4456 | 4.4502 | 0.2253 | 0.9996 | NA |
| AAEL014799 | | 0.2555 | 6.3737 | 0.2254 | 0.9996 | |
| AAEL004422 | | 0.5036 | 2.4795 | 0.2255 | 0.9996 | |
| AAEL001644 | | 1.3182 | -1.1109 | 0.2255 | 0.9996 | |
| AAEL009842 | GALE12 | 0.3453 | 4.2613 | 0.2256 | 0.9996 | Galectin [Source:UniProtKB/TrEMBL;Acc:Q16UP1] |
| AAEL005713 | | 0.4728 | 3.1442 | 0.2257 | 0.9996 | |
| AAEL004533 | GPRMGL2 | 0.4274 | 5.0628 | 0.2258 | 0.9996 | GPCR Metabotropic glutamate Family [Source:VB Community Annotation] |
| AAEL008768 | | 0.3051 | 6.6284 | 0.2261 | 0.9996 | multiprotein bridging factor, putative [Source:VB Community Annotation] |
| AAEL000579 | | 0.3799 | 5.1595 | 0.2262 | 0.9996 | |
| AAEL004050 | | 0.4638 | 2.5183 | 0.2262 | 0.9996 | Diphosphomevalonate decarboxylase [Source:UniProtKB/TrEMBL;Acc:Q17DS1] |
| AAEL019858 | NA | 0.3823 | 4.4256 | 0.2263 | 0.9996 | NA |

| | | | | | | |
|---|---|---|---|---|---|---|
| AAEL000541 | | -0.5681 | 4.6107 | 0.2263 | 0.9996 | fasciclin, putative [Source:VB Community Annotation] |
| AAEL011063 | | -0.3407 | 6.4443 | 0.2263 | 0.9996 | tumor endothelial marker 7 precursor [Source:VB Community Annotation] |
| AAEL013643 | | -0.4945 | 4.3195 | 0.2266 | 0.9996 | mitochondrial transcription factor A, putative [Source:VB Community Annotation] |
| AAEL005790 | | -0.7046 | 8.5377 | 0.2268 | 0.9996 | Malic enzyme [Source:UniProtKB/TrEMBL;Acc:Q178T5] |
| AAEL001914 | | 0.3969 | 4.5881 | 0.2269 | 0.9996 | scavenger receptor, putative [Source:VB Community Annotation] |
| AAEL011496 | | -0.8287 | 1.8333 | 0.2269 | 0.9996 | chitinase [Source:VB Community Annotation] |
| AAEL008070 | | 0.4939 | 5.0049 | 0.2270 | 0.9996 | |
| AAEL004355 | | -0.5051 | 4.6092 | 0.2271 | 0.9996 | |
| AAEL023701 | NA | 0.4508 | 6.3122 | 0.2271 | 0.9996 | NA |
| AAEL012137 | | 0.4685 | 3.5919 | 0.2274 | 0.9996 | |
| AAEL012980 | | 0.6088 | 5.6435 | 0.2276 | 0.9996 | Pescadillo homolog [Source:UniProtKB/Swiss-Prot;Acc:Q0IE95] |
| AAEL006649 | | 1.0931 | 0.3158 | 0.2279 | 0.9996 | tnf receptor associated factor [Source:VB Community Annotation] |
| AAEL019581 | NA | -1.4356 | -0.6716 | 0.2279 | 0.9996 | NA |
| AAEL001202 | | -0.7067 | 1.7364 | 0.2284 | 0.9996 | |
| AAEL011982 | | 0.3937 | 3.3230 | 0.2284 | 0.9996 | cortactin [Source:VB Community Annotation] |
| AAEL006705 | | -1.2801 | -0.9708 | 0.2287 | 0.9996 | |
| AAEL020378 | NA | 0.3774 | 3.8998 | 0.2287 | 0.9996 | NA |
| AAEL013533 | | -0.5888 | 6.9023 | 0.2287 | 0.9996 | |
| AAEL019700 | NA | -0.7554 | 7.2097 | 0.2287 | 0.9996 | NA |
| AAEL009056 | | 0.3825 | 2.9244 | 0.2288 | 0.9996 | |
| AAEL009032 | | -0.4411 | 4.9062 | 0.2290 | 0.9996 | |
| AAEL001584 | | -1.1438 | 1.6200 | 0.2290 | 0.9996 | |
| AAEL014972 | | 1.5436 | -0.1243 | 0.2290 | 0.9996 | |
| AAEL014895 | | 0.4049 | 3.7168 | 0.2291 | 0.9996 | M protein type, putative [Source:VB Community Annotation] |
| AAEL013322 | | 0.5553 | 4.9052 | 0.2292 | 0.9996 | |
| AAEL027054 | NA | 0.4321 | 2.7853 | 0.2292 | 0.9996 | NA |
| AAEL010112 | | 0.3884 | 4.6943 | 0.2293 | 0.9996 | |
| AAEL006789 | | 0.8890 | 3.4361 | 0.2293 | 0.9996 | tbp-associated factor [Source:VB Community Annotation] |
| AAEL011025 | | 1.9401 | 4.0618 | 0.2293 | 0.9996 | vacuolar ATP synthase subunit ac39 [Source:VB Community Annotation] |
| AAEL002940 | | 1.0578 | 2.6078 | 0.2294 | 0.9996 | AMP dependent ligase [Source:VB Community Annotation] |
| AAEL003012 | | -1.5839 | -2.6304 | 0.2295 | 0.9996 | protease m1 zinc metalloprotease [Source:VB Community Annotation] |
| AAEL000836 | | 0.4460 | 2.7779 | 0.2297 | 0.9996 | spermatogenesis associated factor [Source:VB Community Annotation] |
| AAEL011571 | Gr14 | 0.8730 | -0.3536 | 0.2297 | 0.9996 | gustatory receptor Gr14 [Source:VB Community Annotation] |
| AAEL011771 | | -0.7935 | 6.0858 | 0.2298 | 0.9996 | |
| AAEL022109 | NA | -0.6723 | 1.6100 | 0.2298 | 0.9996 | NA |
| AAEL002672 | | -1.2933 | 0.7765 | 0.2299 | 0.9996 | matrix metalloproteinase [Source:VB Community Annotation] |
| AAEL007209 | | -1.2035 | 3.1611 | 0.2301 | 0.9996 | ADP-ribosylation factor, putative [Source:VB Community Annotation] |
| AAEL000428 | Tdo | 0.5056 | 5.1429 | 0.2301 | 0.9996 | tryptophan 2,3-dioxygenase (TDO)(EC 1.13.11.11) [Source:VB Community Annotation] |
| AAEL010182 | | 0.4009 | 4.1404 | 0.2303 | 0.9996 | |
| AAEL011021 | | -0.3929 | 3.5591 | 0.2303 | 0.9996 | |

| | | | | | | |
|---|---|---|---|---|---|---|
| AAEL027134 | NA | 0.6161 | 2.8866 | 0.2303 | 0.9996 | NA |
| AAEL012207 | | 0.9654 | 8.0588 | 0.2303 | 0.9996 | myosin light chain 1, [Source:VB Community Annotation] |
| AAEL021811 | NA | -1.3774 | 0.6582 | 0.2305 | 0.9996 | NA |
| AAEL008101 | | -0.4918 | 2.6980 | 0.2310 | 0.9996 | |
| AAEL013088 | | 0.6502 | 3.2224 | 0.2310 | 0.9996 | |
| AAEL012686 | RpS23 | -0.3282 | 11.0684 | 0.2312 | 0.9996 | 40S ribosomal protein S23 [Source:UniProtKB/TrEMBL;Acc:Q1HRM5] |
| AAEL006859 | | 0.4803 | 6.0167 | 0.2312 | 0.9996 | Myb-interacting protein, putative [Source:VB Community Annotation] |
| AAEL005530 | | -0.6560 | 5.7046 | 0.2314 | 0.9996 | |
| AAEL000330 | | 0.3069 | 5.5841 | 0.2315 | 0.9996 | |
| AAEL005423 | | 0.3301 | 5.1055 | 0.2315 | 0.9996 | |
| AAEL028025 | NA | 0.4695 | 2.3300 | 0.2316 | 0.9996 | NA |
| AAEL019836 | NA | -0.6281 | 6.5644 | 0.2317 | 0.9996 | NA |
| AAEL003462 | | 1.4962 | 2.4708 | 0.2318 | 0.9996 | aromatic amino acid decarboxylase [Source:VB Community Annotation] |
| AAEL020689 | NA | -1.0382 | 6.0612 | 0.2319 | 0.9996 | NA |
| AAEL027897 | NA | 0.5020 | 2.4124 | 0.2319 | 0.9996 | NA |
| AAEL025845 | NA | -1.7553 | 0.1278 | 0.2319 | 0.9996 | NA |
| AAEL025896 | NA | 0.8595 | 0.1342 | 0.2321 | 0.9996 | NA |
| AAEL027109 | NA | 0.9512 | 0.2703 | 0.2325 | 0.9996 | NA |
| AAEL000910 | | -0.9334 | 2.9803 | 0.2325 | 0.9996 | Fatty acyl-CoA reductase [Source:UniProtKB/TrEMBL;Acc:Q17MZ2] |
| AAEL026494 | NA | -1.2702 | 0.0452 | 0.2327 | 0.9996 | NA |
| AAEL026788 | NA | 0.5884 | 2.9389 | 0.2327 | 0.9996 | NA |
| AAEL014556 | | 0.9483 | 4.1680 | 0.2328 | 0.9996 | |
| AAEL026791 | NA | 0.4853 | 4.4380 | 0.2329 | 0.9996 | NA |
| AAEL013898 | | 0.2647 | 5.0846 | 0.2333 | 0.9996 | |
| AAEL005526 | | 0.6329 | 3.2535 | 0.2334 | 0.9996 | |
| AAEL001151 | | -0.9743 | 3.2660 | 0.2334 | 0.9996 | n-acetylgalactosaminyltransferase [Source:VB Community Annotation] |
| AAEL012939 | | -0.3390 | 9.0982 | 0.2335 | 0.9996 | gamma-subunit,methylmalonyl-CoA decarboxylase, putative [Source:VB Community Annotation] |
| AAEL009298 | | -1.0040 | 0.8352 | 0.2335 | 0.9996 | 2-Keto-3-deoxy-(6-phospho-)gluconate aldolase, putative [Source:VB Community Annotation] |
| AAEL005179 | | -0.7645 | 4.1188 | 0.2335 | 0.9996 | |
| AAEL003566 | | 1.5015 | -1.9526 | 0.2335 | 0.9996 | |
| AAEL001414 | LRIM9 | -0.8107 | 9.2013 | 0.2337 | 0.9996 | leucine-rich immune protein (Short) [Source:VB Community Annotation] |
| AAEL028806 | NA | -0.5149 | 2.9297 | 0.2337 | 0.9996 | NA |
| AAEL010486 | | 0.6650 | 4.5081 | 0.2338 | 0.9996 | viral IAP-associated factor, putative [Source:VB Community Annotation] |
| AAEL011492 | | -0.9991 | -0.0371 | 0.2339 | 0.9996 | |
| AAEL005621 | GPROP4 | -1.0275 | 1.7541 | 0.2339 | 0.9996 | long wavelength sensitive opsin [Source:VB Community Annotation] |
| AAEL010803 | | 1.1339 | -0.0159 | 0.2339 | 0.9996 | ncd [Source:VB Community Annotation] |
| AAEL020589 | NA | -0.8420 | 4.0824 | 0.2339 | 0.9996 | NA |
| AAEL012267 | | 0.5622 | 3.8307 | 0.2340 | 0.9996 | macroglobulin/complement [Source:VB Community Annotation] |
| AAEL009909 | | -0.4156 | 4.1477 | 0.2343 | 0.9996 | cln3/battenin [Source:VB Community Annotation] |
| AAEL007109 | | -0.7530 | 0.7252 | 0.2343 | 0.9996 | |

| | | | | | | |
|---|---|---|---|---|---|---|
| AAEL019980 | NA | 0.3465 | 5.5707 | 0.2345 | 0.9996 | NA |
| AAEL023894 | NA | -0.6046 | 3.3661 | 0.2345 | 0.9996 | NA |
| AAEL007762 | mRpL40 | -0.5475 | 5.0336 | 0.2346 | 0.9996 | mitochondrial ribosomal protein, L40, putative [Source:VB Community Annotation] |
| AAEL013253 | | 0.3657 | 5.7398 | 0.2347 | 0.9996 | |
| AAEL008116 | | 0.7025 | 1.6905 | 0.2347 | 0.9996 | |
| AAEL002152 | | 0.4047 | 5.9620 | 0.2347 | 0.9996 | kinesin-associated proteins [Source:VB Community Annotation] |
| AAEL026804 | NA | 0.3921 | 4.6232 | 0.2350 | 0.9996 | NA |
| AAEL003428 | | -0.3230 | 7.0657 | 0.2350 | 0.9996 | |
| AAEL002838 | | -0.6307 | 1.4040 | 0.2351 | 0.9996 | |
| AAEL013063 | APG18A | -0.7968 | 6.3597 | 0.2352 | 0.9996 | autophagy related gene [Source:VB Community Annotation] |
| AAEL010078 | | 0.4262 | 5.3429 | 0.2352 | 0.9996 | |
| AAEL028241 | NA | 1.8798 | 0.5613 | 0.2355 | 0.9996 | NA |
| AAEL005243 | | -1.3111 | 2.2766 | 0.2356 | 0.9996 | |
| AAEL005905 | | 0.4841 | 2.8986 | 0.2357 | 0.9996 | |
| AAEL005145 | | 0.4317 | 3.6157 | 0.2357 | 0.9996 | |
| AAEL025560 | NA | 0.4910 | 4.7685 | 0.2357 | 0.9996 | NA |
| AAEL009273 | | 0.4785 | 3.9745 | 0.2358 | 0.9996 | inosine-5-monophosphate dehydrogenase [Source:VB Community Annotation] |
| AAEL002402 | | 0.6671 | 4.0918 | 0.2358 | 0.9996 | |
| AAEL011776 | | 0.5187 | 4.7961 | 0.2359 | 0.9996 | soluble nsf attachment protein (snap) [Source:VB Community Annotation] |
| AAEL011051 | | -0.2343 | 7.0330 | 0.2360 | 0.9996 | porcupine [Source:VB Community Annotation] |
| AAEL000705 | | -0.5791 | 2.3145 | 0.2361 | 0.9996 | steroid dehydrogenase [Source:VB Community Annotation] |
| AAEL003144 | | 0.7131 | 3.4464 | 0.2363 | 0.9996 | |
| AAEL010205 | | 0.3686 | 9.2943 | 0.2363 | 0.9996 | |
| AAEL028079 | NA | 0.5598 | 2.4355 | 0.2364 | 0.9996 | NA |
| AAEL011440 | | 1.1077 | 0.5161 | 0.2364 | 0.9996 | |
| AAEL000635 | | -0.3789 | 5.3902 | 0.2364 | 0.9996 | cop9 complex subunit 7a [Source:VB Community Annotation] |
| AAEL020101 | NA | 0.3396 | 3.6195 | 0.2370 | 0.9996 | NA |
| AAEL017133 | | -0.5721 | 5.4999 | 0.2371 | 0.9996 | |
| AAEL003812 | | 0.5488 | 5.9348 | 0.2372 | 0.9996 | |
| AAEL003927 | | 0.8491 | 0.2770 | 0.2375 | 0.9996 | |
| AAEL012796 | GPRGHP3 | -0.3900 | 3.7833 | 0.2376 | 0.9996 | GPCR Growth Hormone Releasing Hormone Family [Source:VB Community Annotation] |
| AAEL024360 | NA | 1.6314 | -0.2595 | 0.2377 | 0.9996 | NA |
| AAEL011594 | | 0.4114 | 5.5483 | 0.2378 | 0.9996 | serine/threonine protein kinase [Source:VB Community Annotation] |
| AAEL028037 | NA | -0.6885 | 6.7622 | 0.2379 | 0.9996 | NA |
| AAEL009451 | | -1.3836 | 0.7093 | 0.2380 | 0.9996 | actin [Source:VB Community Annotation] |
| AAEL000152 | | -0.6214 | 1.6069 | 0.2381 | 0.9996 | |
| AAEL000641 | | 0.3737 | 8.8859 | 0.2381 | 0.9996 | protein disulfide isomerase [Source:VB Community Annotation] |
| AAEL010065 | | -0.3060 | 7.0994 | 0.2382 | 0.9996 | protein disulfide-isomerase A6 precursor [Source:VB Community Annotation] |
| AAEL001307 | | 1.8612 | -1.2035 | 0.2383 | 0.9996 | SEC14, putative [Source:VB Community Annotation] |
| AAEL003785 | | -0.3224 | 4.5174 | 0.2383 | 0.9996 | |

| | | | | | | |
|---|---|---|---|---|---|---|
| AAEL004558 | wuho | -0.5031 | 2.2757 | 0.2383 | 0.9996 | tRNA (guanine-N(7)-)-methyltransferase subunit wuho [Source:VB Community Annotation] |
| AAEL008838 | | 0.6010 | 1.7097 | 0.2385 | 0.9996 | |
| AAEL012113 | | 0.3298 | 6.4438 | 0.2391 | 0.9996 | vacuolar ATP synthase proteolipid subunit [Source:VB Community Annotation] |
| AAEL004791 | | -0.7427 | 2.0773 | 0.2393 | 0.9996 | sorting nexin [Source:VB Community Annotation] |
| AAEL021409 | NA | 1.1303 | 0.7988 | 0.2394 | 0.9996 | NA |
| AAEL020622 | NA | 1.3262 | -1.0980 | 0.2400 | 0.9996 | NA |
| AAEL009691 | | -0.6946 | 9.1867 | 0.2401 | 0.9996 | carboxylase:pyruvate/acetyl-coa/propionyl-coa [Source:VB Community Annotation] |
| AAEL020470 | NA | 1.0370 | 1.9367 | 0.2403 | 0.9996 | NA |
| AAEL005013 | | -0.2920 | 6.7039 | 0.2403 | 0.9996 | |
| AAEL000670 | | -0.4870 | 7.6046 | 0.2403 | 0.9996 | methionine sulfoxide reductase [Source:VB Community Annotation] |
| AAEL007560 | | -0.2881 | 4.9925 | 0.2404 | 0.9996 | core 1 udp-galactose:n-acetylgalactosamine-alpha-r beta 1,3- galactosyltransferase [Source:VB Community Annotation] |
| AAEL019774 | NA | -0.3990 | 3.9142 | 0.2405 | 0.9996 | NA |
| AAEL007554 | | -0.5780 | 3.1645 | 0.2405 | 0.9996 | |
| AAEL012820 | | -0.4798 | 6.0213 | 0.2405 | 0.9996 | |
| AAEL006819 | | -0.6130 | 2.8635 | 0.2406 | 0.9996 | |
| AAEL016601 | tRNA-Ala | -0.9584 | 0.5663 | 0.2408 | 0.9996 | |
| AAEL006826 | | -1.4138 | -0.7470 | 0.2409 | 0.9996 | |
| AAEL008790 | | 0.4340 | 3.3353 | 0.2409 | 0.9996 | ATP synthase coupling factor B, putative [Source:VB Community Annotation] |
| AAEL007835 | | 0.6323 | 2.7925 | 0.2410 | 0.9996 | serine/threonine protein kinase [Source:VB Community Annotation] |
| AAEL022370 | NA | 0.3716 | 3.5220 | 0.2410 | 0.9996 | NA |
| AAEL002088 | | -0.5250 | 4.3826 | 0.2411 | 0.9996 | sodium channel, auxiliary protein, putative [Source:VB Community Annotation] |
| AAEL020895 | NA | -0.6427 | 9.2118 | 0.2415 | 0.9996 | NA |
| AAEL013562 | | -0.8441 | 0.5724 | 0.2415 | 0.9996 | zinc finger protein, putative [Source:VB Community Annotation] |
| AAEL013540 | | 0.3675 | 4.1493 | 0.2416 | 0.9996 | |
| AAEL008165 | | 0.6927 | 1.7927 | 0.2417 | 0.9996 | |
| AAEL005896 | | 0.5231 | 4.8129 | 0.2418 | 0.9996 | |
| AAEL021955 | NA | 0.9978 | 2.4555 | 0.2420 | 0.9996 | NA |
| AAEL007729 | | -0.3580 | 4.0817 | 0.2420 | 0.9996 | |
| AAEL011060 | | 0.7375 | 0.5174 | 0.2421 | 0.9996 | |
| AAEL000444 | | 0.3264 | 5.8204 | 0.2421 | 0.9996 | udp-glucose glycoprotein:glucosyltransferase [Source:VB Community Annotation] |
| AAEL005918 | | 1.2786 | 1.5356 | 0.2421 | 0.9996 | ATP-binding cassette transporter [Source:VB Community Annotation] |
| AAEL011925 | mRpS31 | -0.2804 | 4.5622 | 0.2425 | 0.9996 | mitochondrial ribosomal protein, S31, putative [Source:VB Community Annotation] |
| AAEL001455 | | 0.4400 | 3.0109 | 0.2425 | 0.9996 | |
| AAEL018270 | | 0.3617 | 3.6527 | 0.2427 | 0.9996 | |
| AAEL020603 | NA | 0.8236 | 1.2803 | 0.2427 | 0.9996 | NA |
| AAEL011744 | | 0.5116 | 4.5384 | 0.2428 | 0.9996 | DEAD box ATP-dependent RNA helicase [Source:VB Community Annotation] |
| AAEL004503 | RpS21 | -0.5317 | 10.7043 | 0.2429 | 0.9996 | 40S ribosomal protein S21 [Source:UniProtKB/TrEMBL;Acc:Q1HR25] |
| AAEL001859 | | 0.3296 | 5.8441 | 0.2429 | 0.9996 | vesicle protein sorting-associated [Source:VB Community Annotation] |
| AAEL021683 | NA | -0.6624 | 1.3674 | 0.2431 | 0.9996 | NA |
| AAEL013675 | | -0.4434 | 7.8365 | 0.2435 | 0.9996 | eukaryotic translation initiation factor [Source:VB Community Annotation] |

| | | | | | | |
|---|---|---|---|---|---|---|
| AAEL005311 | | 0.3901 | 4.6821 | 0.2437 | 0.9996 | |
| AAEL005660 | | -0.5458 | 3.5936 | 0.2437 | 0.9996 | p60 epidermal growth factor receptor, putative [Source:VB Community Annotation] |
| AAEL006958 | | -1.9230 | -0.6502 | 0.2438 | 0.9996 | cell adhesion molecule [Source:VB Community Annotation] |
| AAEL007634 | | 1.1871 | 3.7770 | 0.2443 | 0.9996 | Protein KRTCAP2 homolog [Source:UniProtKB/Swiss-Prot;Acc:Q1HQF8] |
| AAEL011560 | | -0.3274 | 6.1447 | 0.2444 | 0.9996 | |
| AAEL014993 | | -0.9445 | -1.2768 | 0.2448 | 0.9996 | |
| AAEL011454 | | -0.8419 | 3.8100 | 0.2448 | 0.9996 | |
| AAEL026381 | NA | -0.5312 | 4.1477 | 0.2449 | 0.9996 | NA |
| AAEL001373 | | 0.6346 | 1.1064 | 0.2449 | 0.9996 | lim homeobox protein [Source:VB Community Annotation] |
| AAEL010569 | | -0.6100 | 4.3800 | 0.2450 | 0.9996 | chondroitin synthase [Source:VB Community Annotation] |
| AAEL011192 | | 0.3835 | 6.4933 | 0.2450 | 0.9996 | vacuolar sorting protein vps29 [Source:VB Community Annotation] |
| AAEL008938 | | 0.3760 | 4.3727 | 0.2451 | 0.9996 | |
| AAEL009829 | | 0.3659 | 5.5475 | 0.2452 | 0.9996 | ARL3, putative [Source:VB Community Annotation] |
| AAEL013725 | | -0.6453 | 1.2780 | 0.2454 | 0.9996 | |
| AAEL006788 | | 0.5417 | 2.4003 | 0.2455 | 0.9996 | replication factor C, 37-kDa subunit, putative [Source:VB Community Annotation] |
| AAEL027686 | NA | -0.3333 | 5.8625 | 0.2457 | 0.9996 | NA |
| AAEL000732 | | -2.8470 | 2.5077 | 0.2457 | 0.9996 | |
| AAEL010223 | | -0.3026 | 5.2286 | 0.2458 | 0.9996 | phosphatidylserine decarboxylase [Source:VB Community Annotation] |
| AAEL012272 | | 0.5091 | 5.9450 | 0.2459 | 0.9996 | fk506 binding protein [Source:VB Community Annotation] |
| AAEL020737 | NA | -0.3621 | 6.2245 | 0.2461 | 0.9996 | NA |
| AAEL011196 | | -1.4318 | -0.9786 | 0.2461 | 0.9996 | |
| AAEL007764 | | 0.4260 | 6.3857 | 0.2463 | 0.9996 | dipeptidyl-peptidase [Source:VB Community Annotation] |
| AAEL003373 | | -0.3522 | 5.4734 | 0.2465 | 0.9996 | |
| AAEL009502 | | -1.1278 | 0.5470 | 0.2468 | 0.9996 | |
| AAEL003377 | | 0.3564 | 4.9415 | 0.2469 | 0.9996 | Signal recognition particle subunit SRP72 [Source:UniProtKB/TrEMBL;Acc:Q17FK4] |
| AAEL003873 | | 0.8304 | 6.0634 | 0.2470 | 0.9996 | Glycerol-3-phosphate dehydrogenase [Source:UniProtKB/TrEMBL;Acc:Q17E82] |
| AAEL001616 | | 0.4192 | 7.1661 | 0.2471 | 0.9996 | vesicular-fusion protein nsf [Source:VB Community Annotation] |
| AAEL010066 | | 0.9695 | 4.4351 | 0.2472 | 0.9996 | microfibril-associated protein [Source:VB Community Annotation] |
| AAEL004608 | | 0.5888 | 2.9013 | 0.2475 | 0.9996 | |
| AAEL007563 | DUOX | -1.0209 | 2.8574 | 0.2475 | 0.9996 | Dual Oxidase: Peroxidase and NADPH-Oxidase domains. [Source:VB Community Annotation] |
| AAEL012694 | | -0.7290 | 4.9878 | 0.2476 | 0.9996 | juvenile hormone-inducible protein, putative [Source:VB Community Annotation] |
| AAEL003522 | | 0.5427 | 2.5613 | 0.2477 | 0.9996 | protein arginine n-methyltransferase [Source:VB Community Annotation] |
| AAEL001844 | | -0.5800 | 4.3439 | 0.2479 | 0.9996 | zinc carboxypeptidase [Source:VB Community Annotation] |
| AAEL014139 | | -1.0239 | 1.0093 | 0.2479 | 0.9996 | proacrosin, putative [Source:VB Community Annotation] |
| AAEL016729 | tRNA-Gln | 1.5553 | -0.6827 | 0.2481 | 0.9996 | |
| AAEL026928 | NA | 0.3711 | 3.7888 | 0.2482 | 0.9996 | NA |
| AAEL020542 | NA | -0.6289 | 1.3463 | 0.2483 | 0.9996 | NA |
| AAEL009965 | | 0.6053 | 2.5242 | 0.2484 | 0.9996 | |
| AAEL011500 | | -1.1288 | 0.5637 | 0.2486 | 0.9996 | Cdc42 protein, putative [Source:VB Community Annotation] |
| AAEL010602 | | -0.7057 | 8.1575 | 0.2489 | 0.9996 | starch branching enzyme ii [Source:VB Community Annotation] |

| | | | | | | |
|---|---|---|---|---|---|---|
| AAEL008071 | | -1.1677 | 1.4576 | 0.2491 | 0.9996 | apsB, putative [Source:VB Community Annotation] |
| AAEL001444 | | 1.1214 | -0.3964 | 0.2493 | 0.9996 | |
| AAEL003473 | GPRGRP1 | 1.7402 | -0.4885 | 0.2493 | 0.9996 | GPCRG astrin/Bombesin Family [Source:VB Community Annotation] |
| AAEL000351 | | -0.9357 | 1.3089 | 0.2495 | 0.9996 | |
| AAEL002107 | | 0.4481 | 5.4865 | 0.2496 | 0.9996 | sulfide quinone reductase [Source:VB Community Annotation] |
| AAEL001480 | | 0.3681 | 5.8745 | 0.2497 | 0.9996 | phosphoglucomutase [Source:VB Community Annotation] |
| AAEL019883 | NA | -0.5618 | 6.8713 | 0.2498 | 0.9996 | NA |
| AAEL008706 | | -0.7989 | 2.6537 | 0.2499 | 0.9996 | smooth muscle caldesmon, putative [Source:VB Community Annotation] |
| AAEL000647 | | -0.9249 | 5.7094 | 0.2499 | 0.9996 | |
| AAEL024829 | NA | 0.8574 | 2.2350 | 0.2500 | 0.9996 | NA |
| AAEL003993 | | -0.4297 | 7.0505 | 0.2500 | 0.9996 | cyclohex-1-ene-1-carboxyl-CoA hydratase, putative [Source:VB Community Annotation] |
| AAEL011799 | | 0.4206 | 3.4143 | 0.2502 | 0.9996 | |
| AAEL010435 | | -1.6052 | -1.8009 | 0.2504 | 0.9996 | |
| AAEL007940 | Gr77 | 1.6535 | -0.1031 | 0.2505 | 0.9996 | gustatory receptor Gr77 [Source:VB Community Annotation] |
| AAEL012077 | | 0.3229 | 4.2768 | 0.2506 | 0.9996 | NAD dehydrogenase [Source:VB Community Annotation] |
| AAEL002207 | | -0.3845 | 7.4145 | 0.2506 | 0.9996 | |
| AAEL025486 | NA | 1.3305 | -1.6191 | 0.2507 | 0.9996 | NA |
| AAEL002974 | | -0.7529 | 1.8266 | 0.2508 | 0.9996 | |
| AAEL011870 | | -0.5067 | 5.6745 | 0.2509 | 0.9996 | rap55 [Source:VB Community Annotation] |
| AAEL020071 | NA | -0.8352 | 1.6249 | 0.2510 | 0.9996 | NA |
| AAEL003109 | | 0.2803 | 6.4539 | 0.2515 | 0.9996 | atlastin [Source:VB Community Annotation] |
| AAEL013396 | | 0.4041 | 4.2213 | 0.2516 | 0.9996 | Defective in cullin neddylation protein [Source:UniProtKB/TrEMBL;Acc:A0A1S4FZE3] |
| AAEL021034 | NA | -0.6514 | 1.2379 | 0.2516 | 0.9996 | NA |
| AAEL021396 | NA | 1.4336 | 1.7604 | 0.2519 | 0.9996 | NA |
| AAEL011133 | | 0.6476 | 3.7913 | 0.2519 | 0.9996 | |
| AAEL021464 | NA | 1.1989 | -0.4312 | 0.2520 | 0.9996 | NA |
| AAEL013961 | | -1.3862 | 0.2671 | 0.2520 | 0.9996 | |
| AAEL008109 | | 1.4654 | 1.4002 | 0.2521 | 0.9996 | Protein mab-21-like [Source:UniProtKB/Swiss-Prot;Acc:Q0IES7] |
| AAEL008741 | | -0.3215 | 3.3911 | 0.2523 | 0.9996 | importin (ran-binding protein) [Source:VB Community Annotation] |
| AAEL004983 | | 0.2973 | 5.6131 | 0.2524 | 0.9996 | Inhibitor of growth proteining, ing4 [Source:VB Community Annotation] |
| AAEL025001 | NA | 0.4257 | 4.2825 | 0.2524 | 0.9996 | NA |
| AAEL019712 | NA | -1.4731 | 1.2420 | 0.2524 | 0.9996 | NA |
| AAEL002847 | | 0.5879 | 1.1509 | 0.2525 | 0.9996 | |
| AAEL020116 | NA | 0.3300 | 4.7853 | 0.2525 | 0.9996 | NA |
| AAEL004739 | | -0.6083 | 7.4660 | 0.2525 | 0.9996 | acyl-coa dehydrogenase [Source:VB Community Annotation] |
| AAEL013883 | | -0.4086 | 5.1821 | 0.2526 | 0.9996 | |
| AAEL025883 | NA | 0.8698 | -0.2618 | 0.2526 | 0.9996 | NA |
| AAEL010766 | | 0.4162 | 3.0541 | 0.2528 | 0.9996 | inositol triphosphate 3-kinase c [Source:VB Community Annotation] |
| AAEL008186 | | 1.2011 | -0.3866 | 0.2531 | 0.9996 | |
| AAEL003645 | | 0.3755 | 5.1251 | 0.2533 | 0.9996 | stearoyl-coa desaturase [Source:VB Community Annotation] |

| | | | | | | |
|---|---|---|---|---|---|---|
| AAEL007442 | | -1.1727 | 1.7328 | 0.2535 | 0.9996 | F-box/leucine rich repeat protein [Source:VB Community Annotation] |
| AAEL019673 | NA | -1.2670 | 1.4184 | 0.2536 | 0.9996 | NA |
| AAEL009224 | | 0.3818 | 4.0167 | 0.2539 | 0.9996 | |
| AAEL014662 | | 2.0687 | 0.2569 | 0.2539 | 0.9996 | AMP dependent coa ligase [Source:VB Community Annotation] |
| AAEL004989 | | 1.1000 | 4.1429 | 0.2540 | 0.9996 | RNA-binding protein [Source:VB Community Annotation] |
| AAEL012486 | | 0.5205 | 2.3528 | 0.2540 | 0.9996 | |
| AAEL027606 | NA | 0.4576 | 4.1794 | 0.2541 | 0.9996 | NA |
| AAEL017368 | | -0.6105 | 6.0554 | 0.2543 | 0.9996 | |
| AAEL007221 | | 0.6906 | 1.5526 | 0.2545 | 0.9996 | brain-specific homeobox protein, putative [Source:VB Community Annotation] |
| AAEL026727 | NA | -1.2361 | -0.7852 | 0.2545 | 0.9996 | NA |
| AAEL005491 | | -0.8306 | 4.5320 | 0.2546 | 0.9996 | ABC transporter [Source:VB Community Annotation] |
| AAEL024536 | NA | -0.3829 | 11.3971 | 0.2546 | 0.9996 | NA |
| AAEL005544 | | -0.5002 | 5.6248 | 0.2547 | 0.9996 | YTH domain protein [Source:VB Community Annotation] |
| AAEL002160 | | 0.6257 | 5.8723 | 0.2547 | 0.9996 | GTP-binding protein [Source:VB Community Annotation] |
| AAEL014331 | | 0.5551 | 4.7467 | 0.2552 | 0.9996 | protein phosphatase 2c [Source:VB Community Annotation] |
| AAEL020387 | NA | 0.6031 | 3.6764 | 0.2553 | 0.9996 | NA |
| AAEL012920 | GPRALS3 | -0.7088 | 1.6245 | 0.2554 | 0.9996 | GPCR Galanin/Allatostatin Family [Source:VB Community Annotation] |
| AAEL015533 | | -0.5782 | 4.2652 | 0.2555 | 0.9996 | |
| AAEL000184 | | 1.1485 | 0.7956 | 0.2555 | 0.9996 | |
| AAEL000679 | | -0.2954 | 7.8488 | 0.2557 | 0.9996 | NEDD8, putative [Source:VB Community Annotation] |
| AAEL002357 | | 0.3680 | 4.2904 | 0.2559 | 0.9996 | |
| AAEL008178 | | 0.5377 | 2.3129 | 0.2561 | 0.9996 | DNA replication licensing factor MCM1 [Source:VB Community Annotation] |
| AAEL001759 | RpS9 | -0.7153 | 10.1963 | 0.2564 | 0.9996 | 40S ribosomal protein S9 [Source:VB Community Annotation] |
| AAEL006338 | | -1.0489 | -0.3047 | 0.2564 | 0.9996 | sulfotransferase (sult) [Source:VB Community Annotation] |
| AAEL022268 | NA | 0.3480 | 3.9123 | 0.2564 | 0.9996 | NA |
| AAEL012276 | | -0.3146 | 6.0742 | 0.2566 | 0.9996 | survival motor neuron protein [Source:VB Community Annotation] |
| AAEL010570 | | -1.2425 | 2.7114 | 0.2567 | 0.9996 | acetylcholine receptor, beta-type subunit invertebrate [Source:VB Community Annotation] |
| AAEL015432 | | -1.0172 | 7.5912 | 0.2569 | 0.9996 | trypsin, putative [Source:VB Community Annotation] |
| AAEL002716 | | 0.7308 | 2.8976 | 0.2570 | 0.9996 | |
| AAEL012770 | CYP325N1 | -0.5262 | 4.0788 | 0.2571 | 0.9996 | cytochrome P450 [Source:VB Community Annotation] |
| AAEL008189 | | 0.4392 | 3.8796 | 0.2571 | 0.9996 | |
| AAEL004515 | | -0.5251 | 2.7937 | 0.2572 | 0.9996 | |
| AAEL025393 | NA | 0.5257 | 3.9202 | 0.2572 | 0.9996 | NA |
| AAEL013679 | | 0.4550 | 4.3347 | 0.2572 | 0.9996 | coilin-interacting nulcear ATPase protein, putative [Source:VB Community Annotation] |
| AAEL026758 | NA | -0.3977 | 3.8278 | 0.2575 | 0.9996 | NA |
| AAEL024657 | NA | -0.5485 | 2.1040 | 0.2576 | 0.9996 | NA |
| AAEL025321 | NA | -0.5803 | 8.3108 | 0.2576 | 0.9996 | NA |
| AAEL001591 | | 0.4706 | 3.4341 | 0.2577 | 0.9996 | zinc finger protein [Source:VB Community Annotation] |
| AAEL007383 | | 0.4290 | 10.4449 | 0.2578 | 0.9996 | secreted ferritin G subunit precursor, putative [Source:VB Community Annotation] |
| AAEL025680 | NA | -1.2722 | 3.5431 | 0.2580 | 0.9996 | NA |

| | | | | | | |
|---|---|---|---|---|---|---|
| AAEL028195 | NA | -0.7164 | 2.2439 | 0.2583 | 0.9996 | NA |
| AAEL009658 | | -0.3725 | 6.5680 | 0.2585 | 0.9996 | alpha,alpha-trehalase [Source:VB Community Annotation] |
| AAEL005676 | | -0.9026 | 1.2564 | 0.2586 | 0.9996 | adenosine deaminase [Source:VB Community Annotation] |
| AAEL012994 | | 0.3333 | 6.3814 | 0.2589 | 0.9996 | Glucose-6-phosphate isomerase [Source:UniProtKB/TrEMBL;Acc:Q16KI0] |
| AAEL007650 | | 0.5519 | 5.0558 | 0.2592 | 0.9996 | chaperonin [Source:VB Community Annotation] |
| AAEL021041 | NA | 0.9723 | -0.0739 | 0.2593 | 0.9996 | NA |
| AAEL001871 | | 0.3885 | 3.6684 | 0.2594 | 0.9996 | Protein-lysine N-methyltransferase AAEL001871 [Source:UniProtKB/TrEMBL;Acc:Q17K09] |
| AAEL010388 | | 0.3885 | 3.7412 | 0.2597 | 0.9996 | |
| AAEL005038 | | 0.3502 | 7.0064 | 0.2598 | 0.9996 | ubiquitin-conjugating enzyme E2 [Source:VB Community Annotation] |
| AAEL001594 | | -0.8331 | 3.3848 | 0.2599 | 0.9996 | |
| AAEL026825 | NA | -0.3789 | 5.7980 | 0.2600 | 0.9996 | NA |
| AAEL023575 | NA | -1.7955 | -1.9108 | 0.2601 | 0.9996 | NA |
| AAEL001181 | | -0.3663 | 4.4839 | 0.2601 | 0.9996 | |
| AAEL021054 | NA | -0.6760 | 5.9810 | 0.2602 | 0.9996 | NA |
| AAEL002624 | | -0.7530 | 6.1897 | 0.2603 | 0.9996 | serine protease [Source:VB Community Annotation] |
| AAEL023243 | NA | -1.7774 | -0.3057 | 0.2603 | 0.9996 | NA |
| AAEL020709 | NA | -0.7438 | 2.2988 | 0.2604 | 0.9996 | NA |
| AAEL004705 | | -1.0168 | 1.0401 | 0.2605 | 0.9996 | |
| AAEL008530 | | 0.4283 | 4.4427 | 0.2605 | 0.9996 | |
| AAEL008940 | | -0.5957 | 7.2353 | 0.2605 | 0.9996 | chaoptin [Source:VB Community Annotation] |
| AAEL000510 | | 0.3552 | 4.8835 | 0.2609 | 0.9996 | |
| AAEL006098 | | 0.3452 | 6.2489 | 0.2610 | 0.9996 | |
| AAEL012676 | | 1.1903 | 3.0997 | 0.2611 | 0.9996 | |
| AAEL002279 | | 0.3840 | 4.1328 | 0.2614 | 0.9996 | |
| AAEL008782 | | -1.6395 | -1.1597 | 0.2615 | 0.9996 | serine-type enodpeptidase, [Source:VB Community Annotation] |
| AAEL001304 | | 0.7399 | 4.7515 | 0.2616 | 0.9996 | nuclear hormone receptor ftz-f1 beta [Source:VB Community Annotation] |
| AAEL021629 | NA | -0.5737 | 2.4159 | 0.2617 | 0.9996 | NA |
| AAEL000860 | | -0.4262 | 7.0050 | 0.2619 | 0.9996 | |
| AAEL006830 | | -0.6044 | 2.7671 | 0.2620 | 0.9996 | yellow protein precursor [Source:VB Community Annotation] |
| AAEL013648 | | -0.3802 | 4.6887 | 0.2620 | 0.9996 | Fatty acyl-CoA reductase [Source:UniProtKB/TrEMBL;Acc:Q16II8] |
| AAEL000175 | eIF3-S8 | -0.2695 | 7.0957 | 0.2620 | 0.9996 | eukaryotic translation initiation factor 3 subunit C [Source:VB Community Annotation] |
| AAEL013054 | | 0.5943 | 1.2649 | 0.2623 | 0.9996 | |
| AAEL004697 | | 0.2982 | 5.6223 | 0.2623 | 0.9996 | synoviolin [Source:VB Community Annotation] |
| AAEL020960 | NA | -0.6023 | 5.6186 | 0.2624 | 0.9996 | NA |
| AAEL012237 | | 0.8423 | 3.0766 | 0.2624 | 0.9996 | bhlhzip transcription factor max/bigmax [Source:VB Community Annotation] |
| AAEL017971 | U2 | 0.7258 | 1.0807 | 0.2631 | 0.9996 | U2 spliceosomal RNA [Source:RFAM;Acc:RF00004] |
| AAEL019425 | NA | 0.3323 | 3.8373 | 0.2631 | 0.9996 | NA |
| AAEL011825 | | 0.5110 | 3.9887 | 0.2632 | 0.9996 | aryl hydrocarbon receptor [Source:VB Community Annotation] |
| AAEL010368 | | -0.4405 | 5.2560 | 0.2632 | 0.9996 | |
| AAEL002629 | | -0.8178 | 6.9120 | 0.2632 | 0.9996 | serine protease [Source:VB Community Annotation] |

| | | | | | | |
|---|---|---|---|---|---|---|
| AAEL010381 | | -0.9944 | -0.3131 | 0.2633 | 0.9996 | glucosyl/glucuronosyl transferases [Source:VB Community Annotation] |
| AAEL010898 | | 0.3925 | 5.3086 | 0.2634 | 0.9996 | |
| AAEL024406 | NA | -0.6849 | 6.9874 | 0.2636 | 0.9996 | NA |
| AAEL021359 | NA | -0.4180 | 3.2724 | 0.2636 | 0.9996 | NA |
| AAEL026367 | NA | 1.5421 | 1.4853 | 0.2636 | 0.9996 | NA |
| AAEL011476 | | 0.6365 | 7.3773 | 0.2636 | 0.9996 | |
| AAEL018293 | | 0.3950 | 4.1767 | 0.2637 | 0.9996 | |
| AAEL001717 | | 0.3846 | 3.1250 | 0.2640 | 0.9996 | |
| AAEL004547 | | -1.1214 | 4.0363 | 0.2640 | 0.9996 | |
| AAEL003929 | | -0.4321 | 5.2528 | 0.2641 | 0.9996 | |
| AAEL009899 | | -0.8718 | 5.8231 | 0.2641 | 0.9996 | |
| AAEL013903 | | 0.4412 | 2.6892 | 0.2642 | 0.9996 | gamma-tubulin complex component 2 (gcp-2) [Source:VB Community Annotation] |
| AAEL017513 | | -1.0946 | 9.4530 | 0.2644 | 0.9996 | |
| AAEL008105 | | 0.2486 | 5.6978 | 0.2646 | 0.9996 | glutamate cysteine ligase [Source:VB Community Annotation] |
| AAEL007367 | | 0.4003 | 4.1391 | 0.2648 | 0.9996 | |
| AAEL007151 | | -0.5079 | 6.6539 | 0.2648 | 0.9996 | paramyosin, putative [Source:VB Community Annotation] |
| AAEL021276 | NA | 0.4545 | 5.3450 | 0.2652 | 0.9996 | NA |
| AAEL008496 | | 1.0951 | -0.3801 | 0.2652 | 0.9996 | |
| AAEL002633 | CYP9J31 | -0.3553 | 5.4340 | 0.2653 | 0.9996 | cytochrome P450 [Source:VB Community Annotation] |
| AAEL006206 | | 1.5166 | -0.7995 | 0.2655 | 0.9996 | n-twist [Source:VB Community Annotation] |
| AAEL011867 | | -0.5005 | 5.3483 | 0.2655 | 0.9996 | |
| AAEL002358 | | -0.5603 | 6.2718 | 0.2656 | 0.9996 | |
| AAEL003089 | | 0.5551 | 2.5857 | 0.2656 | 0.9996 | |
| AAEL020396 | NA | -1.0268 | 4.5769 | 0.2657 | 0.9996 | NA |
| AAEL026278 | NA | 0.7035 | 5.9624 | 0.2663 | 0.9996 | NA |
| AAEL006169 | | -0.3480 | 10.6293 | 0.2664 | 0.9996 | Lysosomal aspartic protease Precursor (EC 3.4.23.-) [Source:VB Community Annotation] |
| AAEL020840 | NA | 0.6535 | 1.5485 | 0.2665 | 0.9996 | NA |
| AAEL025032 | NA | 0.6939 | 2.3943 | 0.2666 | 0.9996 | NA |
| AAEL012218 | | -0.3747 | 4.8483 | 0.2667 | 0.9996 | mitochondria associated granulocyte macrophage csf signaling molecule [Source:VB Community Annotation] |
| AAEL009891 | | 0.3288 | 4.6591 | 0.2667 | 0.9996 | |
| AAEL009637 | | -0.3234 | 9.2875 | 0.2667 | 0.9996 | cathepsin b [Source:VB Community Annotation] |
| AAEL022219 | NA | 0.5367 | 3.3690 | 0.2667 | 0.9996 | NA |
| AAEL002047 | RpS10 | 0.3497 | 11.2194 | 0.2669 | 0.9996 | 40S ribosomal protein S10 [Source:VB Community Annotation] |
| AAEL002654 | | 1.9123 | 0.6804 | 0.2670 | 0.9996 | |
| AAEL008783 | | -0.3655 | 5.2286 | 0.2673 | 0.9996 | |
| AAEL027614 | NA | -1.4383 | -2.0739 | 0.2675 | 0.9996 | NA |
| AAEL010802 | | 0.7505 | 2.3777 | 0.2678 | 0.9996 | |
| AAEL004317 | | 0.6487 | 4.5807 | 0.2678 | 0.9996 | |
| AAEL020044 | NA | -0.4688 | 5.7757 | 0.2680 | 0.9996 | NA |
| AAEL021147 | NA | -0.4287 | 4.4186 | 0.2680 | 0.9996 | NA |

| | | | | | | |
|---|---|---|---|---|---|---|
| AAEL021879 | NA | -0.9981 | 1.5635 | 0.2680 | 0.9996 | NA |
| AAEL003674 | | 0.4479 | 4.0125 | 0.2681 | 0.9996 | |
| AAEL007558 | | 0.3483 | 4.5299 | 0.2681 | 0.9996 | helicase [Source:VB Community Annotation] |
| AAEL006362 | | -0.6464 | 5.6947 | 0.2682 | 0.9996 | mitochondrial solute carrier [Source:VB Community Annotation] |
| AAEL012621 | | -0.3273 | 6.3628 | 0.2685 | 0.9996 | arginine/serine-rich splicing factor [Source:VB Community Annotation] |
| AAEL015559 | | -1.6326 | -1.6013 | 0.2686 | 0.9996 | zinc carboxypeptidase [Source:VB Community Annotation] |
| AAEL013458 | | 0.4415 | 9.6481 | 0.2686 | 0.9996 | glutamine synthetase 1, 2 (glutamate-amonia ligase) (gs) [Source:VB Community Annotation] |
| AAEL006487 | | -0.3450 | 5.5539 | 0.2687 | 0.9996 | sodium/solute symporter [Source:VB Community Annotation] |
| AAEL007527 | | 0.8159 | 0.8451 | 0.2688 | 0.9996 | |
| AAEL014177 | | 0.7787 | 3.3193 | 0.2689 | 0.9996 | ADP-ribosylation factor, arf [Source:VB Community Annotation] |
| AAEL014333 | | 0.6519 | 1.8536 | 0.2691 | 0.9996 | |
| AAEL001811 | | 0.2548 | 3.7120 | 0.2693 | 0.9996 | |
| AAEL003527 | | 0.5008 | 2.9403 | 0.2696 | 0.9996 | |
| AAEL007482 | Med27 | 0.5422 | 3.3760 | 0.2697 | 0.9996 | Mediator of RNA polymerase II transcription subunit 27 (Mediator complex subunit 27) [Source:VB Community Annotation] |
| AAEL017381 | | -0.6459 | 2.5939 | 0.2700 | 0.9996 | |
| AAEL017539 | CYP6BY1 | 1.4030 | -0.3832 | 0.2701 | 0.9996 | cytochrome P450 [Source:VB Community Annotation] |
| AAEL020350 | NA | 1.3190 | -1.4927 | 0.2701 | 0.9996 | NA |
| AAEL027189 | NA | 0.4122 | 5.4012 | 0.2701 | 0.9996 | NA |
| AAEL012718 | | -1.4209 | 0.3386 | 0.2704 | 0.9996 | |
| AAEL018130 | | 0.7980 | 3.8701 | 0.2705 | 0.9996 | |
| AAEL010576 | | 0.2964 | 7.6966 | 0.2707 | 0.9996 | modifier of mdg4 [Source:VB Community Annotation] |
| AAEL013434 | | 0.4404 | 4.7667 | 0.2710 | 0.9996 | spaetzle-like cytokine [Source:VB Community Annotation] |
| AAEL017186 | | 0.7901 | 1.0510 | 0.2711 | 0.9996 | ATP-dependent DNA helicase PIF1 [Source:UniProtKB/TrEMBL;Acc:J9HF21] |
| AAEL019527 | NA | -0.9704 | 2.8205 | 0.2714 | 0.9996 | NA |
| AAEL001099 | | 0.3462 | 3.4103 | 0.2714 | 0.9996 | DEAD box polypeptide [Source:VB Community Annotation] |
| AAEL025982 | NA | 0.5248 | 2.5094 | 0.2714 | 0.9996 | NA |
| AAEL006992 | CYP6AG6 | -0.5631 | 3.0746 | 0.2714 | 0.9996 | cytochrome P450 [Source:VB Community Annotation] |
| AAEL017332 | | 0.4360 | 2.7230 | 0.2716 | 0.9996 | |
| AAEL023603 | NA | 0.7871 | 5.7188 | 0.2717 | 0.9996 | NA |
| AAEL026852 | NA | 0.4458 | 3.6679 | 0.2718 | 0.9996 | NA |
| AAEL009489 | | 0.8480 | 0.3618 | 0.2719 | 0.9996 | forkhead box protein (AaegFOXM1) [Source:VB Community Annotation] |
| AAEL021449 | NA | -0.8489 | 0.5577 | 0.2720 | 0.9996 | NA |
| AAEL012380 | PGRPLA | -0.9067 | 1.1544 | 0.2720 | 0.9996 | Peptidoglycan Recognition Protein (Long) [Source:VB Community Annotation] |
| AAEL026253 | NA | -0.2144 | 5.6433 | 0.2721 | 0.9996 | NA |
| AAEL009844 | | -1.9417 | -0.6887 | 0.2721 | 0.9996 | |
| AAEL007392 | | -0.3623 | 6.7416 | 0.2723 | 0.9996 | mitochondrial import receptor subunit tom20 [Source:VB Community Annotation] |
| AAEL009029 | ALDH9029 | -0.3398 | 6.2006 | 0.2724 | 0.9996 | aldehyde dehydrogenase [Source:VB Community Annotation] |
| AAEL012094 | | 0.2953 | 6.9700 | 0.2724 | 0.9996 | casein kinase ii, alpha chain (cmgc group iv) [Source:VB Community Annotation] |
| AAEL011202 | | 0.3086 | 6.3458 | 0.2726 | 0.9996 | bhlhzip transcription factor bigmax [Source:VB Community Annotation] |
| AAEL009385 | | -1.0213 | 3.5934 | 0.2727 | 0.9996 | |

| | | | | | | |
|---|---|---|---|---|---|---|
| AAEL012278 | | 0.3789 | 5.7499 | 0.2727 | 0.9996 | metalloprotease [Source:VB Community Annotation] |
| AAEL000372 | | -0.3929 | 8.0480 | 0.2728 | 0.9996 | myo inositol monophosphatase [Source:VB Community Annotation] |
| AAEL023666 | NA | -1.8761 | -0.7257 | 0.2728 | 0.9996 | NA |
| AAEL022142 | NA | -0.5544 | 1.9895 | 0.2731 | 0.9996 | NA |
| AAEL002581 | | -0.5704 | 2.3087 | 0.2732 | 0.9996 | structural maintenance of chromosomes 6 smc6 [Source:VB Community Annotation] |
| AAEL008017 | CYP4C50 | 0.6509 | 1.5241 | 0.2733 | 0.9996 | cytochrome P450 [Source:VB Community Annotation] |
| AAEL026069 | NA | -0.4184 | 7.4235 | 0.2735 | 0.9996 | NA |
| AAEL025526 | NA | -1.2443 | -2.4310 | 0.2735 | 0.9996 | NA |
| AAEL013626 | | 0.4833 | 3.1368 | 0.2735 | 0.9996 | |
| AAEL023664 | NA | -0.8791 | 1.2207 | 0.2737 | 0.9996 | NA |
| AAEL020729 | NA | -2.6434 | 0.3488 | 0.2737 | 0.9996 | NA |
| AAEL001641 | | 0.4509 | 7.1993 | 0.2739 | 0.9996 | deoxyribonuclease I, putative [Source:VB Community Annotation] |
| AAEL014733 | | 0.4960 | 4.7310 | 0.2740 | 0.9996 | nuclear pore complex protein nup214 [Source:VB Community Annotation] |
| AAEL011810 | | 0.5988 | 5.2177 | 0.2740 | 0.9996 | |
| AAEL012248 | | 0.5193 | 3.8548 | 0.2742 | 0.9996 | histamine-gated chloride channel subunit [Source:VB Community Annotation] |
| AAEL026837 | NA | 0.6917 | 4.0443 | 0.2743 | 0.9996 | NA |
| AAEL013316 | | 0.3878 | 3.2367 | 0.2743 | 0.9996 | smile protein [Source:VB Community Annotation] |
| AAEL002718 | | -1.1755 | 1.3787 | 0.2744 | 0.9996 | chitin synthase [Source:VB Community Annotation] |
| AAEL004410 | | -0.7272 | 4.2418 | 0.2745 | 0.9996 | protein phosphatase [Source:VB Community Annotation] |
| AAEL010843 | | -0.2346 | 7.1856 | 0.2745 | 0.9996 | |
| AAEL006969 | | -0.8735 | 1.1008 | 0.2747 | 0.9996 | |
| AAEL000257 | | 0.3130 | 3.5667 | 0.2749 | 0.9996 | |
| AAEL000595 | | -0.2779 | 5.2577 | 0.2750 | 0.9996 | beat protein [Source:VB Community Annotation] |
| AAEL000004 | | 0.7360 | 2.7280 | 0.2750 | 0.9996 | glycosyltransferase [Source:VB Community Annotation] |
| AAEL019530 | NA | -0.5552 | 3.2905 | 0.2754 | 0.9996 | NA |
| AAEL023389 | NA | -1.4784 | -0.9109 | 0.2755 | 0.9996 | NA |
| AAEL011621 | CTLMA13 | -0.7177 | 2.0691 | 0.2756 | 0.9996 | C-Type Lectin (CTL) - mannose binding. [Source:VB Community Annotation] |
| AAEL009295 | | -0.3940 | 4.7480 | 0.2759 | 0.9996 | lachesin [Source:VB Community Annotation] |
| AAEL008761 | | 0.3849 | 3.7773 | 0.2761 | 0.9996 | |
| AAEL006771 | | -0.4773 | 7.5854 | 0.2763 | 0.9996 | |
| AAEL008232 | | 0.4494 | 6.0263 | 0.2763 | 0.9996 | sugar transporter [Source:VB Community Annotation] |
| AAEL018057 | | 0.3363 | 5.5154 | 0.2765 | 0.9996 | |
| AAEL019945 | NA | -0.4342 | 2.0686 | 0.2765 | 0.9996 | NA |
| AAEL025990 | NA | 1.0422 | 0.0790 | 0.2767 | 0.9996 | NA |
| AAEL006129 | | 0.4851 | 5.7919 | 0.2768 | 0.9996 | Deoxyhypusine hydroxylase [Source:UniProtKB/TrEMBL;Acc:Q177G8] |
| AAEL009465 | | 0.3898 | 3.1419 | 0.2769 | 0.9996 | replication factor c / DNA polymerase iii gamma-tau subunit [Source:VB Community Annotation] |
| AAEL002182 | | 0.3253 | 5.6470 | 0.2769 | 0.9996 | t-diRNAhydrouridine synthase [Source:VB Community Annotation] |
| AAEL000962 | | 0.6516 | 1.1408 | 0.2769 | 0.9996 | Bardet-Biedl syndrome 7 protein homolog [Source:UniProtKB/TrEMBL;Acc:Q17MR4] |
| AAEL020214 | NA | -2.2065 | 2.0606 | 0.2774 | 0.9996 | NA |
| AAEL005665 | SRPN3 | -0.8872 | 3.1359 | 0.2775 | 0.9996 | Serine Protease Inhibitor (serpin)  likely cleavage at T/I. [Source:VB Community Annotation] |

| | | | | | | |
|---|---|---|---|---|---|---|
| AAEL014357 | | -0.9768 | 0.8828 | 0.2776 | 0.9996 | |
| AAEL004716 | | -0.6062 | 6.8080 | 0.2779 | 0.9996 | chromodomain helicase DNA binding protein [Source:VB Community Annotation] |
| AAEL012265 | | 0.4022 | 3.2644 | 0.2783 | 0.9996 | transcription initiation factor RRN3 [Source:VB Community Annotation] |
| AAEL012508 | | -0.4374 | 2.9154 | 0.2783 | 0.9996 | |
| AAEL008798 | | 0.9598 | -0.2503 | 0.2783 | 0.9996 | |
| AAEL026868 | NA | -0.9299 | 0.5081 | 0.2786 | 0.9996 | NA |
| AAEL009877 | | 0.3359 | 3.6293 | 0.2786 | 0.9996 | amyloid binding protein [Source:VB Community Annotation] |
| AAEL012084 | | 0.2657 | 5.3389 | 0.2787 | 0.9996 | |
| AAEL007825 | | 0.3222 | 3.8448 | 0.2787 | 0.9996 | |
| AAEL010171 | PGRPLB | -0.8277 | 6.2192 | 0.2787 | 0.9996 | peptidoglycan recognition protein (Long) [Source:VB Community Annotation] |
| AAEL012547 | | -1.1138 | 1.8153 | 0.2788 | 0.9996 | lkb1 interacting protein [Source:VB Community Annotation] |
| AAEL000529 | RpS21 | -0.3910 | 2.7501 | 0.2788 | 0.9996 | 40S ribosomal protein S21 [Source:UniProtKB/TrEMBL;Acc:Q1HR25] |
| AAEL000385 | | 0.3813 | 3.4541 | 0.2790 | 0.9996 | developmentally regulated RNA-binding protein [Source:VB Community Annotation] |
| AAEL020530 | NA | 0.5804 | 4.3838 | 0.2790 | 0.9996 | NA |
| AAEL013522 | | -0.3992 | 3.9689 | 0.2791 | 0.9996 | pinn [Source:VB Community Annotation] |
| AAEL020151 | NA | -1.6083 | -0.7805 | 0.2792 | 0.9996 | NA |
| AAEL023409 | NA | -1.1209 | 0.4076 | 0.2794 | 0.9996 | NA |
| AAEL007375 | | 0.3586 | 5.1181 | 0.2796 | 0.9996 | pyruvate dehydrogenase [Source:VB Community Annotation] |
| AAEL012125 | | -1.5688 | 0.7969 | 0.2796 | 0.9996 | activin receptor type I, putative [Source:VB Community Annotation] |
| AAEL012931 | | -0.4329 | 5.5039 | 0.2796 | 0.9996 | |
| AAEL008595 | mael | -1.4115 | -1.4223 | 0.2797 | 0.9996 | Protein maelstrom homolog [Source:UniProtKB/Swiss-Prot;Acc:Q16YA8] |
| AAEL021784 | NA | 0.3666 | 5.1577 | 0.2797 | 0.9996 | NA |
| AAEL006776 | | 0.2675 | 6.0982 | 0.2798 | 0.9996 | blastoderm specific protein 25D, putative [Source:VB Community Annotation] |
| AAEL000564 | | 0.3948 | 4.9737 | 0.2798 | 0.9996 | myotubularin [Source:VB Community Annotation] |
| AAEL007007 | | 1.0916 | 0.3131 | 0.2802 | 0.9996 | DNA replication licensing factor MCM2 [Source:VB Community Annotation] |
| AAEL016612 | tRNA-Ser | -0.9886 | -1.0519 | 0.2805 | 0.9996 | |
| AAEL012473 | | -1.1114 | 3.7296 | 0.2805 | 0.9996 | vav1 [Source:VB Community Annotation] |
| AAEL001052 | | 0.3587 | 6.6375 | 0.2806 | 0.9996 | heat shock protein, putative [Source:VB Community Annotation] |
| AAEL006519 | | 0.5525 | 4.8295 | 0.2806 | 0.9996 | |
| AAEL009132 | CYP6Y3 | -0.6062 | 4.6373 | 0.2806 | 0.9996 | cytochrome P450 [Source:VB Community Annotation] |
| AAEL014275 | | 0.3348 | 6.4802 | 0.2807 | 0.9996 | molybdopterin cofactor sulfurase (mosc) [Source:VB Community Annotation] |
| AAEL025232 | NA | -0.8923 | 0.6348 | 0.2809 | 0.9996 | NA |
| AAEL002632 | | -0.6830 | 2.4683 | 0.2812 | 0.9996 | proacrosin, putative [Source:VB Community Annotation] |
| AAEL019975 | NA | -1.6167 | -0.3917 | 0.2813 | 0.9996 | NA |
| AAEL010109 | | 0.3744 | 3.7547 | 0.2814 | 0.9996 | |
| AAEL025518 | NA | -0.4856 | 4.9299 | 0.2815 | 0.9996 | NA |
| AAEL019861 | NA | -0.4899 | 8.0040 | 0.2816 | 0.9996 | NA |
| AAEL014627 | | -0.5505 | 2.8985 | 0.2817 | 0.9996 | short-chain dehydrogenase [Source:VB Community Annotation] |
| AAEL004090 | | 1.1016 | 4.4883 | 0.2818 | 0.9996 | |
| AAEL006921 | | 0.3467 | 4.6995 | 0.2819 | 0.9996 | calmodulin [Source:VB Community Annotation] |

| | | | | | | |
|---|---|---|---|---|---|---|
| AAEL005068 | | 0.3199 | 3.8155 | 0.2819 | 0.9996 | S-phase kinase-associated protein 2 (skp2), putative [Source:VB Community Annotation] |
| AAEL027500 | NA | 0.3482 | 3.9276 | 0.2821 | 0.9996 | NA |
| AAEL004863 | | -0.4022 | 5.3051 | 0.2825 | 0.9996 | |
| AAEL005428 | | 1.9972 | 4.6958 | 0.2825 | 0.9996 | |
| AAEL008878 | | -0.2301 | 3.4846 | 0.2826 | 0.9996 | diacylglycerol o-acyltransferase [Source:VB Community Annotation] |
| AAEL027562 | NA | 1.1142 | 0.7660 | 0.2829 | 0.9996 | NA |
| AAEL006232 | GPRNND3 | -1.8155 | -0.3185 | 0.2829 | 0.9996 | GPCR Orphan/Putative Class D Family [Source:VB Community Annotation] |
| AAEL027172 | NA | 1.2370 | -1.2053 | 0.2830 | 0.9996 | NA |
| AAEL022466 | NA | -1.1930 | 0.1642 | 0.2830 | 0.9996 | NA |
| AAEL003914 | | -0.5413 | 7.2515 | 0.2830 | 0.9996 | calcium/calmodulin-dependent serine protein kinase membrane-associated guanylate kinase (cask) [Source:VB Community Annotation] |
| AAEL002527 | | 0.3730 | 8.4912 | 0.2831 | 0.9996 | |
| AAEL009999 | | 0.4919 | 1.7193 | 0.2832 | 0.9996 | |
| AAEL010220 | | -0.9140 | 0.3315 | 0.2832 | 0.9996 | |
| AAEL019765 | NA | -1.5263 | -0.2312 | 0.2834 | 0.9996 | NA |
| AAEL027037 | NA | -1.0843 | -0.6466 | 0.2834 | 0.9996 | NA |
| AAEL006912 | | -0.3575 | 5.5236 | 0.2835 | 0.9996 | acetyl-coa acetyltransferase 2, [Source:VB Community Annotation] |
| AAEL011201 | | -0.4246 | 2.6737 | 0.2836 | 0.9996 | |
| AAEL025697 | NA | 0.2683 | 8.4858 | 0.2836 | 0.9996 | NA |
| AAEL019630 | NA | 0.3649 | 3.9990 | 0.2837 | 0.9996 | NA |
| AAEL012347 | | 0.5585 | 3.5189 | 0.2838 | 0.9996 | |
| AAEL007591 | | 1.4036 | -1.3542 | 0.2840 | 0.9996 | |
| AAEL011112 | | 1.8128 | -0.6976 | 0.2841 | 0.9996 | alcohol dehydrogenase [Source:VB Community Annotation] |
| AAEL007395 | | 0.3407 | 4.2510 | 0.2841 | 0.9996 | |
| AAEL005567 | | -0.5055 | 8.2036 | 0.2842 | 0.9996 | nucleosome assembly protein [Source:VB Community Annotation] |
| AAEL002551 | | -0.5339 | 6.9622 | 0.2842 | 0.9996 | DNA topoisomerase type I [Source:VB Community Annotation] |
| AAEL012858 | | 1.0742 | 3.9900 | 0.2843 | 0.9996 | |
| AAEL011231 | | -1.0384 | 0.8418 | 0.2844 | 0.9996 | |
| AAEL021475 | NA | 0.5077 | 2.4324 | 0.2844 | 0.9996 | NA |
| AAEL006038 | | 0.3160 | 4.6230 | 0.2847 | 0.9996 | WD-repeat protein [Source:VB Community Annotation] |
| AAEL010783 | | -0.6979 | 10.2123 | 0.2849 | 0.9996 | sodium/potassium-dependent ATPase beta-2 subunit [Source:VB Community Annotation] |
| AAEL019742 | NA | -0.3761 | 4.1051 | 0.2849 | 0.9996 | NA |
| AAEL004068 | | 0.2965 | 4.1772 | 0.2851 | 0.9996 | |
| AAEL005634 | | -0.4422 | 2.9638 | 0.2854 | 0.9996 | harmonin (Usher syndrome 1C protein homolog) (PDZ domain-containing protein) [Source:VB Community Annotation] |
| AAEL003366 | | -0.8101 | 5.9455 | 0.2854 | 0.9996 | DNA-J, putative [Source:VB Community Annotation] |
| AAEL006972 | | 0.4720 | 3.0174 | 0.2855 | 0.9996 | hepatocellular carcinoma-associated antigen [Source:VB Community Annotation] |
| AAEL000707 | | -0.3584 | 3.7281 | 0.2857 | 0.9996 | cell cycle control protein cwf22 [Source:VB Community Annotation] |
| AAEL027342 | NA | 0.2944 | 4.2585 | 0.2859 | 0.9996 | NA |
| AAEL021342 | NA | 0.4123 | 4.5759 | 0.2862 | 0.9996 | NA |
| AAEL003676 | | -0.3398 | 6.2315 | 0.2862 | 0.9996 | myosin I homologue, putative [Source:VB Community Annotation] |
| AAEL003106 | | 0.3200 | 6.3581 | 0.2863 | 0.9996 | clathrin coat associated protein ap-50 [Source:VB Community Annotation] |

| | | | | | | |
|---|---|---|---|---|---|---|
| AAEL014735 | | 0.3695 | 4.4566 | 0.2863 | 0.9996 | |
| AAEL007636 | | 0.2967 | 4.5595 | 0.2863 | 0.9996 | |
| AAEL017467 | | -2.1551 | -0.3339 | 0.2865 | 0.9996 | |
| AAEL000477 | | -1.1748 | -0.3332 | 0.2866 | 0.9996 | NADPH FAD oxidoreductase [Source:VB Community Annotation] |
| AAEL012553 | HOP | -0.6257 | 3.1209 | 0.2866 | 0.9996 | JAKSTAT pathway signalling  Janus Kinase  Hopscotch. [Source:VB Community Annotation] |
| AAEL010338 | | -1.6929 | -1.3630 | 0.2866 | 0.9996 | |
| AAEL008693 | | 0.8632 | 1.2195 | 0.2867 | 0.9996 | cation efflux protein/ zinc transporter [Source:VB Community Annotation] |
| AAEL011134 | | 0.6112 | 1.7913 | 0.2867 | 0.9996 | esophageal cancer associated protein [Source:VB Community Annotation] |
| AAEL009121 | CYP6N9 | -0.7346 | 3.0379 | 0.2868 | 0.9996 | cytochrome P450 [Source:VB Community Annotation] |
| AAEL001014 | | 0.2428 | 4.6589 | 0.2870 | 0.9996 | vacuolar protein sorting-associated [Source:VB Community Annotation] |
| AAEL019652 | NA | 0.2869 | 6.3041 | 0.2870 | 0.9996 | NA |
| AAEL019925 | NA | -0.3461 | 5.8416 | 0.2873 | 0.9996 | NA |
| AAEL002638 | CYP9J6 | -0.3090 | 7.4928 | 0.2873 | 0.9996 | cytochrome P450 [Source:VB Community Annotation] |
| AAEL022354 | NA | -1.2492 | -1.3399 | 0.2874 | 0.9996 | NA |
| AAEL027232 | NA | 0.4024 | 2.9473 | 0.2874 | 0.9996 | NA |
| AAEL027916 | NA | 0.3653 | 6.9435 | 0.2875 | 0.9996 | NA |
| AAEL001730 | | 0.6666 | 3.0348 | 0.2875 | 0.9996 | |
| AAEL003964 | | -0.3232 | 4.2627 | 0.2877 | 0.9996 | |
| AAEL011129 | | 0.6660 | 5.1309 | 0.2878 | 0.9996 | alcohol dehydrogenase [Source:VB Community Annotation] |
| AAEL009631 | | -1.4916 | -1.1350 | 0.2879 | 0.9996 | short-chain dehydrogenase [Source:VB Community Annotation] |
| AAEL007492 | | 0.6808 | 2.0463 | 0.2879 | 0.9996 | |
| AAEL016689 | tRNA-Gly | 1.2679 | -0.1641 | 0.2879 | 0.9996 | |
| AAEL010546 | | -0.3799 | 6.5129 | 0.2880 | 0.9996 | heat shock factor binding protein, putative [Source:VB Community Annotation] |
| AAEL014354 | CLIPB43 | -0.5961 | 4.5687 | 0.2882 | 0.9996 | Clip-Domain Serine Protease family B. [Source:VB Community Annotation] |
| AAEL026490 | NA | -0.3071 | 4.1082 | 0.2884 | 0.9996 | NA |
| AAEL011162 | | 0.4284 | 4.3713 | 0.2885 | 0.9996 | Anaphase Promoting Complex, putative [Source:VB Community Annotation] |
| AAEL014883 | | 0.5723 | 4.2877 | 0.2886 | 0.9996 | dynactin, p27-subunit, putative [Source:VB Community Annotation] |
| AAEL025890 | NA | 1.0407 | -0.2735 | 0.2887 | 0.9996 | NA |
| AAEL003487 | | 0.3064 | 4.9736 | 0.2888 | 0.9996 | development and differentiation-enhancing factor, ddef [Source:VB Community Annotation] |
| AAEL005739 | | 1.6653 | -0.8709 | 0.2889 | 0.9996 | |
| AAEL011630 | | -0.5807 | 3.1262 | 0.2890 | 0.9996 | |
| AAEL004819 | | 0.5260 | 2.1122 | 0.2893 | 0.9996 | |
| AAEL024913 | NA | 0.2748 | 6.7885 | 0.2894 | 0.9996 | NA |
| AAEL006794 | | 0.3409 | 5.1900 | 0.2898 | 0.9996 | dicer-2 [Source:VB Community Annotation] |
| AAEL006309 | | 0.2666 | 4.8151 | 0.2899 | 0.9996 | |
| AAEL006727 | | 0.3249 | 5.7379 | 0.2900 | 0.9996 | multisynthetase complex, auxiliary protein, p38, putative [Source:VB Community Annotation] |
| AAEL006491 | | 0.4382 | 2.1298 | 0.2900 | 0.9996 | peroxisomal targeting signal 2 receptor [Source:VB Community Annotation] |
| AAEL002851 | | -0.5901 | 8.7225 | 0.2901 | 0.9996 | Tubulin beta chain [Source:UniProtKB/TrEMBL;Acc:Q17GX9] |
| AAEL007284 | | 0.4431 | 4.6503 | 0.2902 | 0.9996 | serine/threonine-protein kinase pk61c [Source:VB Community Annotation] |
| AAEL004566 | | -1.0725 | 3.3797 | 0.2902 | 0.9996 | myo inositol monophosphatase [Source:VB Community Annotation] |

| | | | | | | |
|---|---|---|---|---|---|---|
| AAEL011987 | | -1.6180 | -0.5327 | 0.2902 | 0.9996 | |
| AAEL005417 | | 0.8692 | 2.2862 | 0.2902 | 0.9996 | annexin x [Source:VB Community Annotation] |
| AAEL001046 | | -0.6703 | 3.4677 | 0.2902 | 0.9996 | phosphatase fragment [Source:VB Community Annotation] |
| AAEL014067 | | 1.0206 | -0.0662 | 0.2907 | 0.9996 | |
| AAEL027862 | NA | 1.5350 | 1.0272 | 0.2909 | 0.9996 | NA |
| AAEL012040 | | 0.4352 | 2.8660 | 0.2909 | 0.9996 | |
| AAEL008565 | | -0.3738 | 6.7936 | 0.2910 | 0.9996 | metalloprotease m41 ftsh [Source:VB Community Annotation] |
| AAEL002922 | | 0.9203 | 0.1571 | 0.2914 | 0.9996 | glutamate receptor 7 (ampa) [Source:VB Community Annotation] |
| AAEL024830 | NA | -0.8772 | 1.9543 | 0.2916 | 0.9996 | NA |
| AAEL008459 | | 0.6029 | 3.3033 | 0.2918 | 0.9996 | |
| AAEL009117 | CYP6M5 | -0.4905 | 3.1556 | 0.2921 | 0.9996 | cytochrome P450 [Source:VB Community Annotation] |
| AAEL005457 | | -0.3047 | 6.6330 | 0.2921 | 0.9996 | |
| AAEL002661 | | -0.6750 | 1.5127 | 0.2921 | 0.9996 | matrix metalloproteinase [Source:VB Community Annotation] |
| AAEL005720 | | 0.5503 | 3.4154 | 0.2922 | 0.9996 | |
| AAEL027890 | NA | 1.4067 | -0.8311 | 0.2923 | 0.9996 | NA |
| AAEL005704 | | 0.3388 | 4.5627 | 0.2926 | 0.9996 | |
| AAEL021925 | NA | -0.3512 | 4.6007 | 0.2928 | 0.9996 | NA |
| AAEL025053 | NA | 0.6028 | 2.5237 | 0.2931 | 0.9996 | NA |
| AAEL009859 | | 0.4068 | 8.3553 | 0.2932 | 0.9996 | Nucleolar GTP-binding protein 1 [Source:UniProtKB/TrEMBL;Acc:Q16UL5] |
| AAEL020051 | NA | 0.5336 | 5.2577 | 0.2932 | 0.9996 | NA |
| AAEL007715 | RpL21 | 0.3160 | 9.9741 | 0.2933 | 0.9996 | 60S ribosomal protein L21 [Source:UniProtKB/TrEMBL;Acc:Q1HRN4] |
| AAEL002741 | SCRB6 | -0.5587 | 3.4502 | 0.2933 | 0.9996 | Class B Scavenger Receptor (CD36 domain). [Source:VB Community Annotation |
| AAEL018011 | | -0.9268 | 2.2501 | 0.2934 | 0.9996 | |
| AAEL012035 | | -0.3277 | 7.5987 | 0.2935 | 0.9996 | vacuolar ATP synthase subunit e [Source:VB Community Annotation] |
| AAEL014228 | | -1.0894 | 0.0992 | 0.2935 | 0.9996 | pickpocket [Source:VB Community Annotation] |
| AAEL014910 | | -1.2411 | -0.4735 | 0.2935 | 0.9996 | synaptic vesicle protein [Source:VB Community Annotation] |
| AAEL010892 | | 0.3322 | 3.7697 | 0.2936 | 0.9996 | |
| AAEL009946 | | 0.3161 | 5.9266 | 0.2936 | 0.9996 | DnaJ homolog subfamily B member 11 precursor [Source:VB Community Annotation] |
| AAEL010704 | | 0.3266 | 5.6444 | 0.2939 | 0.9996 | clathrin coat assembly protein ap-1 [Source:VB Community Annotation] |
| AAEL024724 | NA | 0.6332 | 1.9676 | 0.2939 | 0.9996 | NA |
| AAEL006134 | | 0.4909 | 1.9207 | 0.2939 | 0.9996 | |
| AAEL000475 | | 0.3834 | 5.0493 | 0.2940 | 0.9996 | |
| AAEL001103 | | 0.3823 | 5.1320 | 0.2940 | 0.9996 | prefoldin, subunit, putative [Source:VB Community Annotation] |
| AAEL019917 | NA | -0.3452 | 4.1510 | 0.2942 | 0.9996 | NA |
| AAEL005207 | | 1.0356 | -0.3765 | 0.2943 | 0.9996 | neprilysin [Source:VB Community Annotation] |
| AAEL001358 | | 0.6225 | 2.6853 | 0.2943 | 0.9996 | |
| AAEL015142 | | -0.4124 | 3.8564 | 0.2944 | 0.9996 | RNA polymerase ii ctd phosphatase [Source:VB Community Annotation] |
| AAEL027229 | NA | -1.4262 | -0.9797 | 0.2947 | 0.9996 | NA |
| AAEL019846 | NA | 0.5109 | 2.7464 | 0.2948 | 0.9996 | NA |
| AAEL008502 | | 0.5867 | 4.9667 | 0.2948 | 0.9996 | |

| | | | | | | |
|---|---|---|---|---|---|---|
| AAEL013005 | | -0.4668 | 6.6144 | 0.2948 | 0.9996 | |
| AAEL005100 | | -0.2756 | 2.8029 | 0.2949 | 0.9996 | |
| AAEL010116 | | 0.2687 | 5.8956 | 0.2950 | 0.9996 | |
| AAEL023712 | NA | 1.7696 | 3.9500 | 0.2952 | 0.9996 | NA |
| AAEL025720 | NA | 0.7630 | 0.5971 | 0.2953 | 0.9996 | NA |
| AAEL028188 | NA | -0.3319 | 10.1328 | 0.2955 | 0.9996 | NA |
| AAEL002831 | | 0.3139 | 4.2602 | 0.2956 | 0.9996 | |
| AAEL006904 | | 0.4271 | 4.3418 | 0.2956 | 0.9996 | |
| AAEL001964 | | -0.3612 | 10.2665 | 0.2957 | 0.9996 | protein serine/threonine kinase, putative [Source:VB Community Annotation] |
| AAEL017030 | | 0.3396 | 7.3721 | 0.2958 | 0.9996 | |
| AAEL007049 | | 0.3115 | 6.9052 | 0.2958 | 0.9996 | proteasome subunit beta type [Source:VB Community Annotation] |
| AAEL014656 | | 1.0226 | -1.0389 | 0.2960 | 0.9996 | |
| AAEL018161 | | -0.5935 | 6.7705 | 0.2961 | 0.9996 | |
| AAEL005214 | | 0.4213 | 4.8795 | 0.2961 | 0.9996 | |
| AAEL008175 | mRpL44 | -0.5846 | 3.0840 | 0.2962 | 0.9996 | mitochondrial ribosomal protein, L44, putative [Source:VB Community Annotation] |
| AAEL000089 | | 0.9149 | 0.0988 | 0.2962 | 0.9996 | |
| AAEL005403 | | 0.4745 | 4.8356 | 0.2963 | 0.9996 | |
| AAEL018344 | | 0.5826 | 2.5507 | 0.2963 | 0.9996 | |
| AAEL006373 | | -0.4266 | 3.1733 | 0.2963 | 0.9996 | serine protease htra2 [Source:VB Community Annotation] |
| AAEL008036 | | -0.7313 | 0.9397 | 0.2964 | 0.9996 | dynein heavy chain [Source:VB Community Annotation] |
| AAEL003682 | | 0.3524 | 4.2104 | 0.2966 | 0.9996 | tropomyosin, putative [Source:VB Community Annotation] |
| AAEL010147 | | -0.8080 | 3.2471 | 0.2967 | 0.9996 | |
| AAEL024812 | NA | 1.6304 | 0.4845 | 0.2969 | 0.9996 | NA |
| AAEL002488 | | 0.4108 | 4.4598 | 0.2969 | 0.9996 | DEAD box ATP-dependent RNA helicase [Source:VB Community Annotation] |
| AAEL011882 | | 0.4186 | 4.3536 | 0.2970 | 0.9996 | trypsin-zeta, putative [Source:VB Community Annotation] |
| AAEL007313 | | 0.3422 | 2.9939 | 0.2971 | 0.9996 | |
| AAEL009268 | | 0.4331 | 2.5343 | 0.2971 | 0.9996 | pla2g4b [Source:VB Community Annotation] |
| AAEL013291 | | 0.3166 | 5.2759 | 0.2975 | 0.9996 | |
| AAEL021952 | NA | 1.1429 | -0.0621 | 0.2976 | 0.9996 | NA |
| AAEL010850 | | 0.5413 | 9.0869 | 0.2976 | 0.9996 | troponin i [Source:VB Community Annotation] |
| AAEL011711 | | 0.3819 | 7.3693 | 0.2977 | 0.9996 | nascent polypeptide associated complex alpha subunit (nac alpha) [Source:VB Community Annotation] |
| AAEL024863 | NA | 0.9251 | 0.1632 | 0.2978 | 0.9996 | NA |
| AAEL007565 | | 0.2422 | 5.6231 | 0.2979 | 0.9996 | leucine rich protein, putative [Source:VB Community Annotation] |
| AAEL024022 | NA | 0.2736 | 5.8232 | 0.2979 | 0.9996 | NA |
| AAEL009208 | | 1.4865 | 3.0057 | 0.2981 | 0.9996 | |
| AAEL005466 | | 0.2643 | 4.1426 | 0.2981 | 0.9996 | |
| AAEL012255 | LRIM13 | -0.9929 | 5.3303 | 0.2984 | 0.9996 | leucine-rich immune protein (Short) [Source:VB Community Annotation] |
| AAEL002678 | | 0.3465 | 5.0846 | 0.2986 | 0.9996 | ras suppressor protein 1, rsu1 [Source:VB Community Annotation] |
| AAEL004603 | | 0.3369 | 6.1613 | 0.2988 | 0.9996 | signal peptidase 12kDa subunit, putative [Source:VB Community Annotation] |
| AAEL000786 | | -0.7396 | 6.5045 | 0.2990 | 0.9996 | |

| | | | | | | |
|---|---|---|---|---|---|---|
| AAEL000750 | | 0.5363 | 5.2306 | 0.2990 | 0.9996 | |
| AAEL001496 | | 0.2336 | 4.9859 | 0.2990 | 0.9996 | transcription elongation factor s-ii [Source:VB Community Annotation] |
| AAEL027755 | NA | 0.3370 | 3.8575 | 0.2990 | 0.9996 | NA |
| AAEL020430 | NA | 1.2043 | 0.1406 | 0.2994 | 0.9996 | NA |
| AAEL001729 | | 0.3814 | 3.5314 | 0.2994 | 0.9996 | |
| AAEL003051 | | -1.5591 | -0.6231 | 0.2995 | 0.9996 | |
| AAEL006424 | D7 | -2.2968 | 5.4193 | 0.2998 | 0.9996 | 37 kDa salivary gland allergen Aed a 2 Precursor (Protein D7)(Allergen Aed a 2) [Source:VB Community Annotation] |
| AAEL012355 | | 0.3774 | 2.9649 | 0.2998 | 0.9996 | helicase [Source:VB Community Annotation] |
| AAEL007066 | | 0.4201 | 3.7215 | 0.3000 | 0.9996 | mitotic checkpoint protein and poly(a)+ RNA export protein [Source:VB Community Annotation] |
| AAEL025736 | NA | -0.2880 | 5.1136 | 0.3000 | 0.9996 | NA |
| AAEL002760 | | 0.4267 | 3.8836 | 0.3000 | 0.9996 | |
| AAEL001656 | | -0.7144 | 4.7350 | 0.3001 | 0.9996 | sodium-dependent phosphate transporter [Source:VB Community Annotation] |
| AAEL003356 | | 0.3419 | 2.9064 | 0.3003 | 0.9996 | |
| AAEL014154 | | 0.3549 | 4.7529 | 0.3003 | 0.9996 | Cytosolic Fe-S cluster assembly factor NUBP2 homolog [Source:UniProtKB/Swiss-Prot;Acc:Q16H50] |
| AAEL022135 | NA | 0.4855 | 2.6219 | 0.3003 | 0.9996 | NA |
| AAEL008377 | | -1.1175 | 0.4185 | 0.3003 | 0.9996 | |
| AAEL022424 | NA | 0.7857 | -0.1364 | 0.3003 | 0.9996 | NA |
| AAEL010135 | | 0.3237 | 3.8600 | 0.3003 | 0.9996 | |
| AAEL009218 | | 0.5568 | 1.4732 | 0.3003 | 0.9996 | mitochondrial carrier protein, putative [Source:VB Community Annotation] |
| AAEL010146 | | -0.4434 | 7.4384 | 0.3004 | 0.9996 | 3-hydroxyacyl-coa dehyrogenase [Source:VB Community Annotation] |
| AAEL005522 | | 0.3268 | 5.3689 | 0.3006 | 0.9996 | |
| AAEL002566 | | -0.6711 | 1.0579 | 0.3006 | 0.9996 | |
| AAEL000038 | CLIPB6 | -0.5118 | 6.3279 | 0.3008 | 0.9996 | Clip-Domain Serine Protease family B. [Source:VB Community Annotation] |
| AAEL018126 | | -0.6785 | 7.7226 | 0.3011 | 0.9996 | |
| AAEL024144 | NA | -0.8808 | 2.2288 | 0.3012 | 0.9996 | NA |
| AAEL027536 | NA | 0.6177 | 4.3498 | 0.3012 | 0.9996 | NA |
| AAEL004907 | | 1.4227 | -0.3177 | 0.3012 | 0.9996 | |
| AAEL000839 | | 0.4431 | 2.9650 | 0.3013 | 0.9996 | O-fucosyltransferase, putative [Source:VB Community Annotation] |
| AAEL019735 | NA | -0.3815 | 4.5488 | 0.3013 | 0.9996 | NA |
| AAEL014698 | | 0.4080 | 3.7923 | 0.3014 | 0.9996 | |
| AAEL001883 | Med17 | -0.4290 | 2.8125 | 0.3015 | 0.9996 | Mediator of RNA polymerase II transcription subunit 17 (Med17) [Source:VB Community Annotation] |
| AAEL019990 | NA | -0.4849 | 8.8870 | 0.3017 | 0.9996 | NA |
| AAEL027885 | NA | 1.9080 | -2.0122 | 0.3017 | 0.9996 | NA |
| AAEL000949 | | 0.3685 | 5.2927 | 0.3018 | 0.9996 | |
| AAEL012471 | DOME | 0.2823 | 4.5089 | 0.3020 | 0.9996 | JAKSTAT pathway signalling Transmembrane Receptor Domeless. [Source:VB Community Annotation] |
| AAEL019904 | NA | 0.5987 | 1.5265 | 0.3020 | 0.9996 | NA |
| AAEL000278 | | -0.4864 | 5.1450 | 0.3020 | 0.9996 | poly(p)/ATP NAD kinase [Source:VB Community Annotation] |
| AAEL013992 | | 0.4041 | 5.8309 | 0.3021 | 0.9996 | |
| AAEL008037 | | 0.3157 | 4.6979 | 0.3022 | 0.9996 | n-acetylgalactosaminyltransferase [Source:VB Community Annotation] |
| AAEL012034 | | 0.3875 | 3.9092 | 0.3022 | 0.9996 | alpha-1,3-mannosyltransferase [Source:VB Community Annotation] |

| | | | | | | |
|---|---|---|---|---|---|---|
| AAEL025401 | NA | 0.3101 | 4.2354 | 0.3023 | 0.9996 | NA |
| AAEL004119 | | 0.2709 | 7.7443 | 0.3023 | 0.9996 | ribonuclease p/mrp subunit [Source:VB Community Annotation] |
| AAEL009744 | | 0.3997 | 2.6552 | 0.3024 | 0.9996 | |
| AAEL000458 | | -0.4409 | 10.1859 | 0.3025 | 0.9996 | |
| AAEL011455 | CTLMA12 | -0.6924 | 4.7704 | 0.3027 | 0.9996 | C-Type Lectin (CTLMA12) - mannose binding [Source:VB Community Annotation] |
| AAEL025065 | NA | 0.3812 | 5.8475 | 0.3029 | 0.9996 | NA |
| AAEL001929 | SPZ5 | -1.3374 | -1.2457 | 0.3029 | 0.9996 | spaetzle-like cytokine [Source:VB Community Annotation] |
| AAEL024367 | NA | 0.6616 | 1.0355 | 0.3030 | 0.9996 | NA |
| AAEL000486 | | 0.2691 | 6.6673 | 0.3032 | 0.9996 | chaperonin [Source:VB Community Annotation] |
| AAEL007710 | | 0.2537 | 5.8009 | 0.3032 | 0.9996 | Ubiquitin thioesterase [Source:UniProtKB/TrEMBL;Acc:Q171B5] |
| AAEL010254 | | 0.4755 | 5.4471 | 0.3032 | 0.9996 | |
| AAEL000875 | | -0.3628 | 5.7695 | 0.3035 | 0.9996 | |
| AAEL025380 | NA | 0.3860 | 4.5734 | 0.3037 | 0.9996 | NA |
| AAEL011242 | | -0.7442 | 1.9647 | 0.3037 | 0.9996 | UDP-glucose 6-dehydrogenase [Source:UniProtKB/TrEMBL;Acc:Q16QM2] |
| AAEL002919 | | 0.4282 | 3.4319 | 0.3038 | 0.9996 | |
| AAEL013730 | | -0.3895 | 4.1809 | 0.3038 | 0.9996 | |
| AAEL017424 | | 0.5261 | 4.7661 | 0.3039 | 0.9996 | |
| AAEL011752 | GSTI1 | -1.1334 | 2.2462 | 0.3039 | 0.9996 | glutathione transferase [Source:VB Community Annotation] |
| AAEL019516 | NA | -1.2337 | 2.2987 | 0.3040 | 0.9996 | NA |
| AAEL015051 | | -0.9295 | 2.5181 | 0.3043 | 0.9996 | glycoside  hydrolases [Source:VB Community Annotation] |
| AAEL010577 | | 0.4362 | 3.8340 | 0.3044 | 0.9996 | |
| AAEL011382 | | 0.3961 | 3.5750 | 0.3044 | 0.9996 | 116 kda U5 small nuclear ribonucleoprotein component [Source:VB Community Annotation] |
| AAEL005224 | | -0.2844 | 4.8318 | 0.3044 | 0.9996 | |
| AAEL004485 | NHE8 | 0.5470 | 3.4146 | 0.3045 | 0.9996 | sodium/hydrogen exchanger 5, 6, 8 (nhe5, nhe6, nhe8) [Source:VB Community Annotation] |
| AAEL012500 | | -0.5413 | 4.4389 | 0.3047 | 0.9996 | ubiquitin-protein ligase [Source:VB Community Annotation] |
| AAEL027164 | NA | 0.3407 | 4.1424 | 0.3047 | 0.9996 | NA |
| AAEL012051 | | -0.3370 | 3.8588 | 0.3048 | 0.9996 | |
| AAEL025610 | NA | 0.6882 | 1.4952 | 0.3049 | 0.9996 | NA |
| AAEL007077 | | -0.7292 | 0.8653 | 0.3049 | 0.9996 | integrin beta 1 binding protein (melusin) [Source:VB Community Annotation] |
| AAEL006128 | | -0.5117 | 2.7952 | 0.3052 | 0.9996 | |
| AAEL021487 | NA | 1.1117 | -1.0329 | 0.3052 | 0.9996 | NA |
| AAEL008141 | PER | 0.8823 | 6.4222 | 0.3053 | 0.9996 | period circadian protein [Source:VB Community Annotation] |
| AAEL006293 | | 1.2487 | -1.0138 | 0.3053 | 0.9996 | |
| AAEL006459 | | 0.4909 | 1.8975 | 0.3054 | 0.9996 | |
| AAEL003342 | | 0.8076 | -0.2442 | 0.3055 | 0.9996 | |
| AAEL026104 | NA | -0.5977 | 1.9484 | 0.3058 | 0.9996 | NA |
| AAEL027905 | NA | -1.0412 | 3.2894 | 0.3058 | 0.9996 | NA |
| AAEL023844 | NA | 0.4745 | 3.7243 | 0.3058 | 0.9996 | NA |
| AAEL023977 | NA | 1.0216 | -0.5417 | 0.3060 | 0.9996 | NA |
| AAEL003484 | | 0.6600 | 1.0137 | 0.3060 | 0.9996 | |

| | | | | | | |
|---|---|---|---|---|---|---|
| AAEL001323 | | -0.4542 | 9.1134 | 0.3061 | 0.9996 | |
| AAEL004675 | | -0.3636 | 5.5300 | 0.3061 | 0.9996 | |
| AAEL027103 | NA | 0.7594 | 0.5785 | 0.3062 | 0.9996 | NA |
| AAEL003903 | | -0.3980 | 5.9250 | 0.3062 | 0.9996 | acid phosphatase-1 [Source:VB Community Annotation] |
| AAEL006521 | | -0.3222 | 5.1434 | 0.3063 | 0.9996 | |
| AAEL008592 | | -0.8296 | -0.2890 | 0.3063 | 0.9996 | ribonuclease iii [Source:VB Community Annotation] |
| AAEL021587 | NA | 0.5209 | 1.2157 | 0.3063 | 0.9996 | NA |
| AAEL009544 | | -0.6359 | 2.9496 | 0.3064 | 0.9996 | |
| AAEL009839 | Cbp80 | -0.2744 | 3.9125 | 0.3065 | 0.9996 | Nuclear cap-binding protein subunit 1 (80 kDa nuclear cap-binding protein)(NCBP 80 kDa subunit)(CBP80) [Source:VB Community Annota |
| AAEL028107 | NA | 0.3520 | 4.8610 | 0.3066 | 0.9996 | NA |
| AAEL022334 | NA | 0.8784 | 7.5430 | 0.3066 | 0.9996 | NA |
| AAEL023751 | NA | 1.3294 | -0.6227 | 0.3067 | 0.9996 | NA |
| AAEL008800 | mRpS26 | -0.3914 | 5.4763 | 0.3069 | 0.9996 | mitochondrial ribosomal protein, S26, putative [Source:VB Community Annotation] |
| AAEL005738 | | -0.4681 | 5.7200 | 0.3070 | 0.9996 | yellow protein precursor [Source:VB Community Annotation] |
| AAEL009415 | | 0.3139 | 4.6279 | 0.3070 | 0.9996 | synembryn [Source:VB Community Annotation] |
| AAEL022595 | NA | 0.5280 | 3.4201 | 0.3071 | 0.9996 | NA |
| AAEL002167 | Gr2 | 0.7262 | 1.2080 | 0.3071 | 0.9996 | gustatory receptor (Gr2) [Source:VB Community Annotation] |
| AAEL009952 | | -0.8736 | -0.0553 | 0.3073 | 0.9996 | |
| AAEL011367 | | 0.5325 | 1.0600 | 0.3077 | 0.9996 | |
| AAEL008655 | GPRVPR2 | -0.9834 | -0.2196 | 0.3078 | 0.9996 | GPCR Vasopressin Family [Source:VB Community Annotation] |
| AAEL005831 | | -0.2636 | 3.3500 | 0.3078 | 0.9996 | |
| AAEL026474 | NA | 0.4852 | 3.3928 | 0.3078 | 0.9996 | NA |
| AAEL000314 | | 0.3483 | 4.6481 | 0.3078 | 0.9996 | malate dehydrogenase [Source:VB Community Annotation] |
| AAEL019878 | NA | 1.7120 | 3.6279 | 0.3079 | 0.9996 | NA |
| AAEL005150 | mRpL23 | -0.2834 | 5.2256 | 0.3079 | 0.9996 | mitochondrial ribosomal protein, L23, putative [Source:VB Community Annotation] |
| AAEL005888 | | 1.0710 | -0.6719 | 0.3080 | 0.9996 | DNA polymerase theta [Source:VB Community Annotation] |
| AAEL028020 | NA | 1.0840 | 0.4688 | 0.3081 | 0.9996 | NA |
| AAEL010568 | | 0.9904 | 0.1805 | 0.3081 | 0.9996 | chromosome-associated kinesin KIF4A (chromokinesin) [Source:VB Community Annotation] |
| AAEL007849 | | 0.4024 | 4.6456 | 0.3083 | 0.9996 | |
| AAEL012654 | | 1.1681 | 0.5049 | 0.3083 | 0.9996 | triacylglycerol lipase, pancreatic [Source:VB Community Annotation] |
| AAEL006214 | | 0.4965 | 2.9527 | 0.3086 | 0.9996 | |
| AAEL005061 | | 0.3448 | 4.4022 | 0.3087 | 0.9996 | sec10 [Source:VB Community Annotation] |
| AAEL001776 | | -1.4992 | 2.9522 | 0.3088 | 0.9996 | |
| AAEL026966 | NA | 0.5987 | 0.8216 | 0.3089 | 0.9996 | NA |
| AAEL014492 | | -0.2496 | 5.9889 | 0.3089 | 0.9996 | |
| AAEL014824 | | 1.1780 | 1.4878 | 0.3091 | 0.9996 | |
| AAEL008755 | | 0.3715 | 4.8108 | 0.3091 | 0.9996 | DNA-directed RNA polymerase II subunit [Source:VB Community Annotation] |
| AAEL012069 | GPXH1 | 0.4709 | 7.4664 | 0.3093 | 0.9996 | Glutathione peroxidase [Source:UniProtKB/TrEMBL;Acc:Q16N54] |
| AAEL006879 | | 0.3114 | 5.7979 | 0.3094 | 0.9996 | folate carrier protein [Source:VB Community Annotation] |
| AAEL009853 | | 1.0553 | -1.0924 | 0.3095 | 0.9996 | trypsin, putative [Source:VB Community Annotation] |

| | | | | | | |
|---|---|---|---|---|---|---|
| AAEL019610 | NA | -0.6760 | 3.5118 | 0.3098 | 0.9996 | NA |
| AAEL006171 | | -0.6474 | 5.3878 | 0.3099 | 0.9996 | n-myc downstream regulated [Source:VB Community Annotation] |
| AAEL007132 | | 0.2706 | 7.2709 | 0.3099 | 0.9996 | dynein heavy chain [Source:VB Community Annotation] |
| AAEL000752 | | 0.2408 | 5.5129 | 0.3100 | 0.9996 | |
| AAEL007311 | | -1.3132 | 1.3271 | 0.3102 | 0.9996 | |
| AAEL014147 | | 0.3378 | 3.6732 | 0.3103 | 0.9996 | |
| AAEL018662 | COX1 | 1.1642 | -1.2447 | 0.3105 | 0.9996 | cytochrome c oxidase subunit I [Source:European Nucleotide Archive;Acc:COX1] |
| AAEL017499 | | -0.2366 | 7.0857 | 0.3105 | 0.9996 | |
| AAEL003632 | CLIPB39 | -0.6634 | 2.4977 | 0.3107 | 0.9996 | Clip-Domain Serine Protease  family B. [Source:VB Community Annotation] |
| AAEL020485 | NA | 0.3679 | 5.2880 | 0.3108 | 0.9996 | NA |
| AAEL006027 | | -0.5486 | 7.5775 | 0.3110 | 0.9996 | lipase [Source:VB Community Annotation] |
| AAEL000195 | | 0.3127 | 3.8267 | 0.3110 | 0.9996 | |
| AAEL004663 | | 0.3508 | 3.4290 | 0.3110 | 0.9996 | pseudouridylate synthase [Source:VB Community Annotation] |
| AAEL012712 | CLIPC13 | 0.4741 | 4.8123 | 0.3112 | 0.9996 | Clip-Domain Serine Protease family C. [Source:VB Community Annotation] |
| AAEL010042 | | -0.3595 | 5.9968 | 0.3117 | 0.9996 | |
| AAEL005791 | | -0.5617 | 2.0568 | 0.3117 | 0.9996 | |
| AAEL018278 | | 0.3254 | 5.3715 | 0.3117 | 0.9996 | |
| AAEL005479 | | -0.3678 | 7.0811 | 0.3117 | 0.9996 | synaptogyrin, [Source:VB Community Annotation] |
| AAEL013213 | | -0.3871 | 3.3273 | 0.3119 | 0.9996 | enhancer of zeste, ezh [Source:VB Community Annotation] |
| AAEL011435 | | 0.4272 | 3.4874 | 0.3120 | 0.9996 | radical sam proteins [Source:VB Community Annotation] |
| AAEL015425 | | 0.4529 | 3.1833 | 0.3120 | 0.9996 | |
| AAEL008324 | | 0.6495 | 2.6622 | 0.3121 | 0.9996 | zinc finger protein [Source:VB Community Annotation] |
| AAEL004004 | | -0.4096 | 2.9105 | 0.3123 | 0.9996 | chromatin regulatory protein sir2 [Source:VB Community Annotation] |
| AAEL013310 | | 0.3864 | 3.8367 | 0.3125 | 0.9996 | |
| AAEL009217 | mRpS35 | -0.3015 | 5.1170 | 0.3127 | 0.9996 | mitochondrial ribosomal protein, S35, putative [Source:VB Community Annotation] |
| AAEL027199 | NA | -0.5788 | 4.9494 | 0.3128 | 0.9996 | NA |
| AAEL009541 | | -0.3324 | 5.0681 | 0.3128 | 0.9996 | |
| AAEL010944 | | -0.3984 | 5.3842 | 0.3128 | 0.9996 | |
| AAEL006564 | | -0.2073 | 6.1750 | 0.3130 | 0.9996 | mitochondrial RNA splicing protein [Source:VB Community Annotation] |
| AAEL026878 | NA | -0.3466 | 5.9082 | 0.3131 | 0.9996 | NA |
| AAEL024950 | NA | -0.3269 | 6.5830 | 0.3131 | 0.9996 | NA |
| AAEL013108 | | 0.3442 | 3.9797 | 0.3132 | 0.9996 | |
| AAEL008320 | | 0.6622 | 0.2152 | 0.3133 | 0.9996 | |
| AAEL013087 | Med16 | 1.1749 | 0.9800 | 0.3133 | 0.9996 | Mediator of RNA polymerase II transcription subunit 16 (Med16) [Source:VB Community Annotation] |
| AAEL001232 | | -1.2744 | 3.8953 | 0.3134 | 0.9996 | tubulointerstitial nephritis antigen [Source:VB Community Annotation] |
| AAEL001007 | | 0.5655 | 3.5928 | 0.3136 | 0.9996 | |
| AAEL013425 | | 0.5077 | 2.1648 | 0.3136 | 0.9996 | |
| AAEL003378 | GPRNNA1 | -0.3673 | 3.8495 | 0.3137 | 0.9996 | GPCR Orphan/Putative Class A Family [Source:VB Community Annotation] |
| AAEL013896 | | 0.4094 | 4.4251 | 0.3138 | 0.9996 | smad4 [Source:VB Community Annotation] |
| AAEL014949 | | -1.3067 | -1.6428 | 0.3139 | 0.9996 | |

| | | | | | | |
|---|---|---|---|---|---|---|
| AAEL007449 | | 0.3651 | 3.7688 | 0.3139 | 0.9996 | |
| AAEL000336 | | -1.1801 | 2.6961 | 0.3142 | 0.9996 | |
| AAEL011448 | | -0.6312 | 3.6303 | 0.3146 | 0.9996 | |
| AAEL005484 | | -1.2741 | -1.3194 | 0.3148 | 0.9996 | sorting nexin [Source:VB Community Annotation] |
| AAEL027013 | NA | 1.3632 | -1.4524 | 0.3148 | 0.9996 | NA |
| AAEL021888 | NA | -0.9553 | 0.9092 | 0.3151 | 0.9996 | NA |
| AAEL006116 | | -1.0065 | 2.6911 | 0.3151 | 0.9996 | Putative e3 ubiquitin-protein ligase herc2 [Source:UniProtKB/TrEMBL;Acc:A0A0P6J635] |
| AAEL006882 | | 1.1183 | -0.0045 | 0.3152 | 0.9996 | |
| AAEL003514 | | -0.4310 | 6.0683 | 0.3152 | 0.9996 | centromere/microtubule binding protein cbf5 [Source:VB Community Annotation] |
| AAEL010377 | | -0.5374 | 0.8339 | 0.3154 | 0.9996 | |
| AAEL025911 | NA | 0.3240 | 5.6078 | 0.3155 | 0.9996 | NA |
| AAEL022822 | NA | -0.4823 | 1.2139 | 0.3157 | 0.9996 | NA |
| AAEL000931 | | -0.4354 | 7.5349 | 0.3158 | 0.9996 | Alkaline phosphatase [Source:UniProtKB/TrEMBL;Acc:Q1HQK7] |
| AAEL014378 | | -0.2954 | 5.9268 | 0.3161 | 0.9996 | |
| AAEL022880 | NA | -0.4989 | 2.6081 | 0.3161 | 0.9996 | NA |
| AAEL008475 | | 0.4347 | 1.7244 | 0.3161 | 0.9996 | |
| AAEL000452 | Pcaf | 0.3263 | 5.3478 | 0.3162 | 0.9996 | histone acetyltransferase [Source:VB Community Annotation] |
| AAEL017198 | | 0.3366 | 10.6277 | 0.3162 | 0.9996 | Ribosomal protein L37 [Source:UniProtKB/TrEMBL;Acc:Q6Q9G1] |
| AAEL021614 | NA | -1.3635 | 0.7935 | 0.3166 | 0.9996 | NA |
| AAEL001246 | | -0.5312 | 2.7944 | 0.3170 | 0.9996 | Thymidylate kinase, putative [Source:VB Community Annotation] |
| AAEL025530 | NA | 1.1821 | 0.3918 | 0.3172 | 0.9996 | NA |
| AAEL014604 | | -0.4473 | 3.8480 | 0.3173 | 0.9996 | cytochrome P450 [Source:VB Community Annotation] |
| AAEL004118 | | 0.6541 | 5.9377 | 0.3173 | 0.9996 | aldo-keto reductase [Source:VB Community Annotation] |
| AAEL023989 | NA | 0.8262 | 2.3455 | 0.3173 | 0.9996 | NA |
| AAEL012456 | | -1.0477 | 1.8912 | 0.3173 | 0.9996 | unkempt protein [Source:VB Community Annotation] |
| AAEL025884 | NA | 0.8099 | 0.7162 | 0.3174 | 0.9996 | NA |
| AAEL027061 | NA | -1.4168 | 0.3176 | 0.3175 | 0.9996 | NA |
| AAEL002694 | | 0.9085 | 1.8819 | 0.3176 | 0.9996 | predicted G-protein coupled receptor (GPCR) [Source:VB Community Annotation] |
| AAEL019441 | NA | -0.3692 | 6.6774 | 0.3176 | 0.9996 | NA |
| AAEL027339 | NA | 0.6883 | 1.0604 | 0.3176 | 0.9996 | NA |
| AAEL022263 | NA | -0.5126 | 3.1481 | 0.3177 | 0.9996 | NA |
| AAEL005438 | | 0.5119 | 2.0267 | 0.3178 | 0.9996 | |
| AAEL003907 | | 0.3703 | 4.0824 | 0.3181 | 0.9996 | ubiquitination factor E4a [Source:VB Community Annotation] |
| AAEL007468 | | 0.4906 | 2.5384 | 0.3181 | 0.9996 | zinc finger protein, putative [Source:VB Community Annotation] |
| AAEL014771 | | 0.3358 | 4.2051 | 0.3182 | 0.9996 | |
| AAEL022456 | NA | -0.4499 | 4.6351 | 0.3183 | 0.9996 | NA |
| AAEL000634 | | -1.4893 | 1.8769 | 0.3184 | 0.9996 | ceramide glucosyltransferase [Source:VB Community Annotation] |
| AAEL011732 | | 0.3181 | 4.5911 | 0.3186 | 0.9996 | prip interacting protein. pimt [Source:VB Community Annotation] |
| AAEL018346 | | -0.4575 | 3.1219 | 0.3188 | 0.9996 | |
| AAEL014386 | | -0.8486 | 4.0591 | 0.3190 | 0.9996 | clip-domain serine protease, putative [Source:VB Community Annotation] |

| | | | | | | |
|---|---|---|---|---|---|---|
| AAEL002139 | | 0.5521 | 4.8990 | 0.3191 | 0.9996 | |
| AAEL024841 | NA | 0.3314 | 6.5615 | 0.3191 | 0.9996 | NA |
| AAEL019632 | NA | -0.2621 | 4.1247 | 0.3192 | 0.9996 | NA |
| AAEL005854 | | -0.8532 | 0.1069 | 0.3193 | 0.9996 | amino acid transporter [Source:VB Community Annotation] |
| AAEL019802 | NA | -0.2428 | 5.9062 | 0.3193 | 0.9996 | NA |
| AAEL001041 | | 0.2680 | 5.8787 | 0.3194 | 0.9996 | guanine nucleotide-binding protein beta 3 (g protein beta3) [Source:VB Community Annotation] |
| AAEL018241 | | -0.8570 | 2.8974 | 0.3194 | 0.9996 | |
| AAEL006271 | CUSOD2 | -0.3915 | 7.9153 | 0.3197 | 0.9996 | copper-zinc (Cu-Zn) superoxide dismutase [Source:VB Community Annotation] |
| AAEL008274 | | -0.4635 | 6.7647 | 0.3199 | 0.9996 | |
| AAEL023078 | NA | -2.9033 | -1.5184 | 0.3199 | 0.9996 | NA |
| AAEL025760 | NA | -0.5751 | 1.7287 | 0.3200 | 0.9996 | NA |
| AAEL007584 | | -0.8297 | -1.0544 | 0.3201 | 0.9996 | |
| AAEL005599 | | -1.1776 | 3.3844 | 0.3201 | 0.9996 | defective proboscis extension response, putative [Source:VB Community Annotation] |
| AAEL002614 | | 0.2558 | 5.0844 | 0.3202 | 0.9996 | equilibrative nucleoside transporter [Source:VB Community Annotation] |
| AAEL013150 | | -0.2735 | 4.2820 | 0.3202 | 0.9996 | Methionine aminopeptidase [Source:UniProtKB/TrEMBL;Acc:Q16K07] |
| AAEL028038 | NA | -0.6893 | 2.8266 | 0.3203 | 0.9996 | NA |
| AAEL023349 | NA | 0.3778 | 4.7435 | 0.3204 | 0.9996 | NA |
| AAEL001708 | | -0.6433 | 4.2729 | 0.3205 | 0.9996 | |
| AAEL020191 | NA | -1.1838 | -1.2432 | 0.3205 | 0.9996 | NA |
| AAEL013026 | | -0.5912 | 3.0238 | 0.3206 | 0.9996 | guanylate cyclase [Source:VB Community Annotation] |
| AAEL007827 | | 0.3472 | 3.4953 | 0.3206 | 0.9996 | |
| AAEL000050 | | 0.7508 | 5.2060 | 0.3209 | 0.9996 | |
| AAEL000024 | | -0.6748 | 5.9009 | 0.3210 | 0.9996 | dopachrome-conversion enzyme (DCE), putative [Source:VB Community Annotation] |
| AAEL019641 | NA | -0.4786 | 5.4740 | 0.3212 | 0.9996 | NA |
| AAEL012242 | | -0.3248 | 3.1144 | 0.3214 | 0.9996 | |
| AAEL008671 | | 0.3029 | 5.9056 | 0.3216 | 0.9996 | Mitochondrial fission 1 protein [Source:UniProtKB/TrEMBL;Acc:Q16Y49] |
| AAEL022793 | NA | 0.4280 | 5.1107 | 0.3216 | 0.9996 | NA |
| AAEL012318 | | -0.3599 | 4.7204 | 0.3219 | 0.9996 | 2-amino-3-ketobutyrate coenzyme a ligase [Source:VB Community Annotation] |
| AAEL001653 | | 0.3358 | 3.5927 | 0.3220 | 0.9996 | fetal globin-inducing factor [Source:VB Community Annotation] |
| AAEL012575 | | -1.3963 | -1.0321 | 0.3221 | 0.9996 | serine protease, putative [Source:VB Community Annotation] |
| AAEL014425 | | -0.8649 | 2.6414 | 0.3221 | 0.9996 | |
| AAEL002887 | arm | 0.3944 | 5.6540 | 0.3222 | 0.9996 | Armadillo segment polarity protein [Source:UniProtKB/Swiss-Prot;Acc:Q17GS9] |
| AAEL010459 | | 0.5196 | 2.1649 | 0.3223 | 0.9996 | |
| AAEL007055 | | -1.7737 | 0.2268 | 0.3224 | 0.9996 | lipase [Source:VB Community Annotation] |
| AAEL013249 | | -0.4539 | 3.5907 | 0.3225 | 0.9996 | |
| AAEL002665 | | -0.7129 | 4.6301 | 0.3225 | 0.9996 | matrix metalloproteinase [Source:VB Community Annotation] |
| AAEL012552 | | -0.2792 | 8.3176 | 0.3230 | 0.9996 | NADH-ubiquinone oxidoreductase [Source:VB Community Annotation] |
| AAEL003417 | | -0.5359 | 7.8453 | 0.3233 | 0.9996 | mitochondrial ATPase inhibitor, putative [Source:VB Community Annotation] |
| AAEL005432 | | 1.3993 | 1.2226 | 0.3236 | 0.9996 | |
| AAEL005203 | | 0.6565 | 1.7557 | 0.3239 | 0.9996 | |

| | | | | | | |
|---|---|---|---|---|---|---|
| AAEL010508 | | -0.4351 | 3.4918 | 0.3239 | 0.9996 | vacuolar protein sorting-associated protein (vps13) [Source:VB Community Annotation] |
| AAEL026323 | NA | 0.8907 | 0.4781 | 0.3241 | 0.9996 | NA |
| AAEL019443 | NA | 0.2368 | 6.2226 | 0.3241 | 0.9996 | NA |
| AAEL008380 | | 0.8099 | 1.5362 | 0.3242 | 0.9996 | |
| AAEL012280 | | 0.2648 | 4.8908 | 0.3243 | 0.9996 | pef protein with a long n-terminal hydrophobic domain (peflin) [Source:VB Community Annotation] |
| AAEL019623 | NA | -0.2702 | 6.0205 | 0.3245 | 0.9996 | NA |
| AAEL023182 | NA | -1.2033 | 7.1585 | 0.3246 | 0.9996 | NA |
| AAEL001363 | | -0.2543 | 4.9276 | 0.3247 | 0.9996 | small nuclear ribonucleoprotein Sm D1, putative [Source:VB Community Annotation] |
| AAEL022520 | NA | -0.9896 | 0.0500 | 0.3250 | 0.9996 | NA |
| AAEL009616 | | 0.4468 | 3.6838 | 0.3255 | 0.9996 | cadherin [Source:VB Community Annotation] |
| AAEL022443 | NA | 0.3023 | 3.6380 | 0.3257 | 0.9996 | NA |
| AAEL005969 | | -1.0619 | 1.6818 | 0.3258 | 0.9996 | phospholipase b, plb1 [Source:VB Community Annotation] |
| AAEL013083 | | 0.5644 | 3.2302 | 0.3259 | 0.9996 | |
| AAEL019828 | NA | 1.7799 | -0.0141 | 0.3260 | 0.9996 | NA |
| AAEL027376 | NA | 0.5270 | 1.3673 | 0.3260 | 0.9996 | NA |
| AAEL011383 | | 0.4561 | 1.7521 | 0.3262 | 0.9996 | MAGE-41 protein, putative [Source:VB Community Annotation] |
| AAEL004242 | | -0.3827 | 7.8916 | 0.3262 | 0.9996 | juvenile hormone-inducible protein, putative [Source:VB Community Annotation] |
| AAEL020011 | NA | -1.1479 | -1.0771 | 0.3262 | 0.9996 | NA |
| AAEL011642 | | -0.4056 | 3.1883 | 0.3263 | 0.9996 | |
| AAEL007696 | REL1A | 0.2930 | 5.7643 | 0.3263 | 0.9996 | TOLL pathway signalling  NF-kappaB Relish-like transcription factor [Source:VB Community Annotation] |
| AAEL009606 | | -0.7018 | 4.1182 | 0.3264 | 0.9996 | |
| AAEL006463 | | -0.3538 | 4.5301 | 0.3264 | 0.9996 | Peroxisome assembly protein 12 [Source:UniProtKB/TrEMBL;Acc:Q176A8] |
| AAEL012947 | | -0.9456 | -0.1065 | 0.3267 | 0.9996 | |
| AAEL009649 | | 0.3149 | 5.3463 | 0.3270 | 0.9996 | brg-1 associated factor [Source:VB Community Annotation] |
| AAEL002713 | | -0.6529 | 2.1452 | 0.3271 | 0.9996 | fibrinogen and fibronectin [Source:VB Community Annotation] |
| AAEL025199 | NA | -0.6111 | 7.4570 | 0.3271 | 0.9996 | NA |
| AAEL019541 | NA | 0.3417 | 6.0092 | 0.3272 | 0.9996 | NA |
| AAEL004147 | | -0.2820 | 4.8521 | 0.3273 | 0.9996 | tetraspanin, putative [Source:VB Community Annotation] |
| AAEL014537 | | -0.4445 | 2.9992 | 0.3274 | 0.9996 | maltose phosphorylase [Source:VB Community Annotation] |
| AAEL003539 | | 0.3321 | 4.1183 | 0.3274 | 0.9996 | WD-repeat protein [Source:VB Community Annotation] |
| AAEL005722 | RpL26 | -0.2604 | 11.2804 | 0.3276 | 0.9996 | 60S ribosomal protein L7a [Source:VB Community Annotation] |
| AAEL002310 | | 0.9915 | 0.8049 | 0.3278 | 0.9996 | |
| AAEL009580 | | -1.4704 | -0.4119 | 0.3279 | 0.9996 | |
| AAEL010891 | | 0.7637 | 2.8491 | 0.3279 | 0.9996 | |
| AAEL011168 | | 0.3792 | 4.2191 | 0.3282 | 0.9996 | GTP-binding protein (i) alpha subunit, gnai [Source:VB Community Annotation] |
| AAEL003147 | | 0.5193 | 2.5463 | 0.3283 | 0.9996 | |
| AAEL006259 | GPROP2 | 0.5542 | 15.9825 | 0.3283 | 0.9996 | long wavelength sensitive opsin [Source:VB Community Annotation] |
| AAEL007243 | | 0.2250 | 6.6353 | 0.3283 | 0.9996 | valacyclovir hydrolase [Source:VB Community Annotation] |
| AAEL003113 | | 0.4004 | 3.1489 | 0.3284 | 0.9996 | |
| AAEL005355 | | -0.2916 | 6.6750 | 0.3285 | 0.9996 | small nuclear ribonucleoprotein sm [Source:VB Community Annotation] |

| | | | | | | |
|---|---|---|---|---|---|---|
| AAEL000245 | | -0.2751 | 7.4438 | 0.3285 | 0.9996 | |
| AAEL019683 | NA | -0.3216 | 4.4102 | 0.3286 | 0.9996 | NA |
| AAEL017164 | | -0.5109 | 1.8122 | 0.3286 | 0.9996 | |
| AAEL010094 | | -0.8002 | 0.5340 | 0.3287 | 0.9996 | cyclin b [Source:VB Community Annotation] |
| AAEL020473 | NA | 0.4710 | 2.0408 | 0.3287 | 0.9996 | NA |
| AAEL003535 | | 0.4149 | 2.7572 | 0.3288 | 0.9996 | |
| AAEL025498 | NA | 0.3358 | 4.4151 | 0.3288 | 0.9996 | NA |
| AAEL005846 | | 0.3058 | 4.4328 | 0.3289 | 0.9996 | |
| AAEL011854 | | -0.4539 | 2.8978 | 0.3296 | 0.9996 | |
| AAEL012180 | | 0.3068 | 3.9588 | 0.3296 | 0.9996 | |
| AAEL001086 | | -0.4793 | 2.2171 | 0.3302 | 0.9996 | |
| AAEL006389 | | -0.3388 | 8.8173 | 0.3303 | 0.9996 | cathepsin l [Source:VB Community Annotation] |
| AAEL001020 | | -0.6659 | 6.5102 | 0.3303 | 0.9996 | anterior fat body protein [Source:VB Community Annotation] |
| AAEL006862 | | 0.3280 | 5.3420 | 0.3303 | 0.9996 | |
| AAEL012089 | | -0.7816 | 6.7889 | 0.3304 | 0.9996 | |
| AAEL002639 | Rpl36-2 | -0.5998 | 3.9931 | 0.3304 | 0.9996 | 60S ribosomal protein L36 [Source:UniProtKB/TrEMBL;Acc:Q17HK5] |
| AAEL027722 | NA | 0.4155 | 3.6376 | 0.3305 | 0.9996 | NA |
| AAEL019503 | NA | -0.7235 | 4.1645 | 0.3307 | 0.9996 | NA |
| AAEL026380 | NA | 0.5618 | 4.1881 | 0.3308 | 0.9996 | NA |
| AAEL000936 | | 0.3502 | 3.8512 | 0.3308 | 0.9996 | |
| AAEL002209 | | 0.4318 | 4.3047 | 0.3309 | 0.9996 | |
| AAEL003014 | | -0.8408 | 6.9736 | 0.3309 | 0.9996 | |
| AAEL008463 | | -0.4577 | 1.5692 | 0.3310 | 0.9996 | DEAD box ATP-dependent RNA helicase [Source:VB Community Annotation] |
| AAEL000471 | | 0.7622 | 2.7403 | 0.3311 | 0.9996 | monocarboxylate transporter [Source:VB Community Annotation] |
| AAEL002298 | | 0.3281 | 3.5302 | 0.3311 | 0.9996 | |
| AAEL006167 | | 0.7347 | 0.2980 | 0.3315 | 0.9996 | runt [Source:VB Community Annotation] |
| AAEL002370 | | 0.3518 | 3.2079 | 0.3317 | 0.9996 | |
| AAEL017363 | | 0.5238 | 5.2811 | 0.3318 | 0.9996 | DNA-directed RNA polymerase subunit [Source:UniProtKB/TrEMBL;Acc:J9HHP4 |
| AAEL006938 | | -0.4621 | 2.3489 | 0.3321 | 0.9996 | |
| AAEL008478 | | -0.2967 | 5.9240 | 0.3323 | 0.9996 | |
| AAEL004277 | | 1.2373 | -0.8626 | 0.3323 | 0.9996 | |
| AAEL001249 | | 0.5132 | 3.0880 | 0.3323 | 0.9996 | |
| AAEL003504 | | -0.6536 | 0.7426 | 0.3324 | 0.9996 | emx homeobox protein [Source:VB Community Annotation] |
| AAEL014138 | SRPN16 | -0.3155 | 5.6529 | 0.3326 | 0.9996 | Serine Protease Inhibitor (serpin) likely cleavage at S/L. [Source:VB Community Annotation] |
| AAEL001215 | | 0.2765 | 4.8263 | 0.3327 | 0.9996 | |
| AAEL008539 | | 0.4275 | 2.3024 | 0.3328 | 0.9996 | |
| AAEL005604 | | -0.5934 | 2.9166 | 0.3328 | 0.9996 | trypsin-epsilon, putative [Source:VB Community Annotation] |
| AAEL007233 | | 0.2570 | 3.4202 | 0.3328 | 0.9996 | zinc finger protein [Source:VB Community Annotation] |
| AAEL020113 | NA | -0.1982 | 5.8497 | 0.3329 | 0.9996 | NA |
| AAEL013003 | | 0.4635 | 3.3052 | 0.3329 | 0.9996 | |

| | | | | | | |
|---|---|---|---|---|---|---|
| AAEL006356 | | 0.4625 | 3.2433 | 0.3330 | 0.9996 | TFIIH basal transcription factor complex p52 subunit [Source:VB Community Annotation] |
| AAEL008582 | RpS2 | 0.2909 | 4.6270 | 0.3331 | 0.9996 | 40S ribosomal protein S2 [Source:VB Community Annotation] |
| AAEL020855 | NA | -0.3306 | 4.0690 | 0.3331 | 0.9996 | NA |
| AAEL003156 | | -1.3028 | -1.6203 | 0.3332 | 0.9996 | fibrinogen and fibronectin [Source:VB Community Annotation] |
| AAEL014946 | | -0.4586 | 3.2723 | 0.3333 | 0.9996 | protease U48 caax prenyl protease rce1 [Source:VB Community Annotation] |
| AAEL008375 | | -0.3914 | 4.8514 | 0.3335 | 0.9996 | |
| AAEL012863 | | -0.3676 | 6.8197 | 0.3336 | 0.9996 | |
| AAEL004297 | | -0.5513 | 8.7854 | 0.3336 | 0.9996 | ATP-citrate synthase [Source:UniProtKB/TrEMBL;Acc:Q17D87] |
| AAEL009414 | | 0.8808 | 7.0216 | 0.3337 | 0.9996 | NADH-ubiquinone oxidoreductase 39 kda subunit [Source:VB Community Annotation] |
| AAEL004059 | | -0.4450 | 7.9233 | 0.3337 | 0.9996 | cystathionine beta-lyase [Source:VB Community Annotation] |
| AAEL021584 | NA | -0.4104 | 6.5742 | 0.3338 | 0.9996 | NA |
| AAEL007344 | | 0.6843 | 2.1954 | 0.3339 | 0.9996 | |
| AAEL005730 | | -0.2399 | 4.2154 | 0.3339 | 0.9996 | cop9 signalosome complex subunit [Source:VB Community Annotation] |
| AAEL007672 | | -0.4193 | 2.4869 | 0.3339 | 0.9996 | myst histone acetyltransferase [Source:VB Community Annotation] |
| AAEL008624 | | -0.2337 | 7.0820 | 0.3340 | 0.9996 | ABC transporter [Source:VB Community Annotation] |
| AAEL000714 | | 0.3499 | 8.7037 | 0.3341 | 0.9996 | sodium-dependent excitatory amino acid transporter [Source:VB Community Annotation] |
| AAEL008625 | | -0.3176 | 5.3926 | 0.3341 | 0.9996 | ABC transporter [Source:VB Community Annotation] |
| AAEL011212 | | 0.3585 | 2.4538 | 0.3342 | 0.9996 | nuclear transcription factor, x-box binding 1 (nfx1) [Source:VB Community Annotation] |
| AAEL011087 | | 0.3148 | 4.9125 | 0.3344 | 0.9996 | DNA-directed RNA polymerase II [Source:VB Community Annotation] |
| AAEL010083 | IMD | -0.6786 | 1.1037 | 0.3345 | 0.9996 | IMD pathway signalling  Immune Deficiency (Imd). [Source:VB Community Annotation] |
| AAEL003568 | | -1.3529 | 0.1719 | 0.3345 | 0.9996 | threonine dehydratase/deaminase [Source:VB Community Annotation] |
| AAEL014137 | CLIPB25 | -0.5677 | 6.3576 | 0.3345 | 0.9996 | Clip-Domain Serine Protease  family B. [Source:VB Community Annotation] |
| AAEL014528 | | -0.3019 | 5.8722 | 0.3346 | 0.9996 | nuclear protein skip [Source:VB Community Annotation] |
| AAEL011687 | | 0.3091 | 4.4475 | 0.3350 | 0.9996 | alternative splicing type 3 and, putative [Source:VB Community Annotation] |
| AAEL001671 | | -1.2748 | 0.2144 | 0.3350 | 0.9996 | |
| AAEL007604 | | -0.8555 | 1.4253 | 0.3351 | 0.9996 | odorant-binding protein 56a, putative [Source:VB Community Annotation] |
| AAEL012011 | | 0.4385 | 3.5139 | 0.3351 | 0.9996 | nedd8-conjugating enzyme nce2 [Source:VB Community Annotation] |
| AAEL000252 | | -0.9189 | 0.7235 | 0.3351 | 0.9996 | |
| AAEL004471 | | 0.3103 | 4.7066 | 0.3351 | 0.9996 | |
| AAEL013413 | | -0.6586 | 2.1788 | 0.3352 | 0.9996 | |
| AAEL012206 | | 0.4011 | 3.3678 | 0.3352 | 0.9996 | microtubule-associated protein tau [Source:VB Community Annotation] |
| AAEL012763 | LRIM24 | -0.5771 | 5.3353 | 0.3352 | 0.9996 | leucine-rich immune protein (Coil-less) [Source:VB Community Annotation] |
| AAEL024250 | NA | -1.4671 | 0.3170 | 0.3353 | 0.9996 | NA |
| AAEL006581 | | -0.7614 | 1.9686 | 0.3353 | 0.9996 | juvenile hormone-inducible protein, putative [Source:VB Community Annotation] |
| AAEL018032 | | -0.2383 | 6.3181 | 0.3355 | 0.9996 | |
| AAEL021611 | NA | -1.2494 | -0.4875 | 0.3356 | 0.9996 | NA |
| AAEL004431 | | -0.3555 | 3.3523 | 0.3356 | 0.9996 | phospholipase c gamma [Source:VB Community Annotation] |
| AAEL025363 | NA | 0.2935 | 7.7160 | 0.3358 | 0.9996 | NA |
| AAEL013284 | LT1 | 1.6471 | -1.9913 | 0.3358 | 0.9996 | late trypsin 1, serine-type enodpeptidase [Source:VB Community Annotation] |
| AAEL005330 | | 0.9910 | -0.5048 | 0.3359 | 0.9996 | atrial natriuretic peptide receptor [Source:VB Community Annotation] |

| | | | | | | |
|---|---|---|---|---|---|---|
| AAEL016041 | tRNA-Gly | 1.1353 | -1.8863 | 0.3360 | 0.9996 | |
| AAEL001131 | | -0.5036 | 4.2111 | 0.3360 | 0.9996 | splicing factor, putative [Source:VB Community Annotation] |
| AAEL005662 | | 0.2379 | 6.2090 | 0.3362 | 0.9996 | adenosine diphosphatase [Source:VB Community Annotation] |
| AAEL020619 | NA | 1.5017 | -1.8852 | 0.3363 | 0.9996 | NA |
| AAEL010432 | | 0.2737 | 4.6836 | 0.3363 | 0.9996 | exocyst complex-subunit protein, 84kD-subunit, putative [Source:VB Community Annotation] |
| AAEL008650 | | 0.7263 | 3.5408 | 0.3364 | 0.9996 | |
| AAEL014084 | | 0.6698 | 0.0322 | 0.3364 | 0.9996 | kinesin eg-5 [Source:VB Community Annotation] |
| AAEL007131 | | 1.3900 | -0.7204 | 0.3365 | 0.9996 | sugar transporter [Source:VB Community Annotation] |
| AAEL024732 | NA | -1.3853 | -1.2939 | 0.3365 | 0.9996 | NA |
| AAEL004730 | | -0.9069 | 1.2107 | 0.3365 | 0.9996 | |
| AAEL021070 | NA | 0.3219 | 5.1448 | 0.3365 | 0.9996 | NA |
| AAEL010262 | | -1.0703 | 5.6316 | 0.3366 | 0.9996 | |
| AAEL004740 | | -0.4691 | 2.3292 | 0.3367 | 0.9996 | 35 kDa GTP-binding protein, putative [Source:VB Community Annotation] |
| AAEL007738 | | 0.4667 | 5.5974 | 0.3367 | 0.9996 | app binding protein [Source:VB Community Annotation] |
| AAEL001974 | | 0.3545 | 5.9262 | 0.3370 | 0.9996 | |
| AAEL022092 | NA | -1.5149 | -1.3916 | 0.3371 | 0.9996 | NA |
| AAEL020695 | NA | 0.3732 | 4.8638 | 0.3371 | 0.9996 | NA |
| AAEL002337 | | -0.2620 | 6.9972 | 0.3372 | 0.9996 | prefoldin, subunit, putative [Source:VB Community Annotation] |
| AAEL010523 | | -0.5117 | 7.9889 | 0.3372 | 0.9996 | |
| AAEL000902 | | -0.3682 | 7.8082 | 0.3373 | 0.9996 | sugar transporter [Source:VB Community Annotation] |
| AAEL003646 | | 0.4832 | 5.6809 | 0.3373 | 0.9996 | |
| AAEL007913 | | 0.8418 | 0.0907 | 0.3375 | 0.9996 | sulfotransferase (sult) [Source:VB Community Annotation] |
| AAEL014271 | | -0.2887 | 5.7321 | 0.3378 | 0.9996 | |
| AAEL010593 | | 0.5101 | 2.3657 | 0.3379 | 0.9996 | |
| AAEL026609 | NA | 0.8298 | 2.4363 | 0.3379 | 0.9996 | NA |
| AAEL007810 | | -0.5193 | 1.6575 | 0.3380 | 0.9996 | |
| AAEL008610 | | 1.4303 | -0.5349 | 0.3380 | 0.9996 | myosin vii [Source:VB Community Annotation] |
| AAEL006970 | | 0.8756 | 0.1887 | 0.3382 | 0.9996 | lipase [Source:VB Community Annotation] |
| AAEL003398 | | -0.4598 | 4.7495 | 0.3383 | 0.9996 | |
| AAEL011819 | | 0.2167 | 4.7611 | 0.3385 | 0.9996 | Cohesin loading complex subunit SCC4 homolog [Source:VB Community Annotation] |
| AAEL001587 | | 0.5382 | 4.0385 | 0.3385 | 0.9996 | |
| AAEL010127 | | 0.9307 | -0.3922 | 0.3385 | 0.9996 | pupal cuticle protein 78E, putative [Source:VB Community Annotation] |
| AAEL014257 | | 0.4355 | 3.9630 | 0.3385 | 0.9996 | |
| AAEL004673 | | 0.6885 | 2.3700 | 0.3389 | 0.9996 | |
| AAEL014316 | | -1.0585 | 3.1522 | 0.3389 | 0.9996 | |
| AAEL008542 | | -0.2343 | 8.5899 | 0.3390 | 0.9996 | kinesin heavy chain subunit [Source:VB Community Annotation] |
| AAEL001328 | | -0.4460 | 5.7710 | 0.3391 | 0.9996 | |
| AAEL004588 | | 1.1287 | 1.9471 | 0.3391 | 0.9996 | |
| AAEL023988 | NA | -1.0924 | -1.3888 | 0.3392 | 0.9996 | NA |
| AAEL010089 | | 0.3459 | 3.6171 | 0.3393 | 0.9996 | |

| | | | | | | |
|---|---|---|---|---|---|---|
| AAEL008585 | | 1.3198 | 1.6877 | 0.3393 | 0.9996 | kinase suppressor of ras (ksr) [Source:VB Community Annotation] |
| AAEL003001 | | 0.2650 | 4.7181 | 0.3395 | 0.9996 | WD-repeat protein [Source:VB Community Annotation] |
| AAEL002158 | | -1.2602 | 3.2136 | 0.3395 | 0.9996 | secreted ferritin G subunit precursor, putative [Source:VB Community Annotation] |
| AAEL023219 | NA | 0.3826 | 4.8196 | 0.3396 | 0.9996 | NA |
| AAEL000341 | | 0.3905 | 3.1760 | 0.3397 | 0.9996 | basic helix-loop-helix protein [Source:VB Community Annotation] |
| AAEL005200 | | 0.7576 | 4.5245 | 0.3397 | 0.9996 | Carboxylic ester hydrolase [Source:UniProtKB/TrEMBL;Acc:A0A1S4F9Y2] |
| AAEL027504 | NA | -0.4839 | 5.5911 | 0.3398 | 0.9996 | NA |
| AAEL024823 | NA | 1.1521 | -0.3343 | 0.3399 | 0.9996 | NA |
| AAEL010633 | | 0.4262 | 2.8609 | 0.3404 | 0.9996 | |
| AAEL012409 | | -0.2731 | 6.3331 | 0.3406 | 0.9996 | pantothenate kinase [Source:VB Community Annotation] |
| AAEL003569 | | 0.4544 | 3.7217 | 0.3407 | 0.9996 | acyl-coa thioesterase [Source:VB Community Annotation] |
| AAEL008752 | | -1.2268 | -1.5767 | 0.3407 | 0.9996 | |
| AAEL010827 | | -0.3384 | 4.2867 | 0.3407 | 0.9996 | programmed cell death protein 11 (pre-rRNA processing protein rrp5) [Source:VB Community Annotation] |
| AAEL010551 | | -0.6927 | 0.9771 | 0.3409 | 0.9996 | |
| AAEL023793 | NA | -1.6930 | -0.1642 | 0.3409 | 0.9996 | NA |
| AAEL008865 | | 0.5735 | 1.9033 | 0.3413 | 0.9996 | oligoribonuclease, mitochondrial [Source:VB Community Annotation] |
| AAEL019582 | NA | 0.7828 | 4.1604 | 0.3414 | 0.9996 | NA |
| AAEL013479 | | 0.3042 | 4.9556 | 0.3415 | 0.9996 | |
| AAEL016986 | | 0.3887 | 3.4077 | 0.3415 | 0.9996 | |
| AAEL004662 | | -0.3399 | 4.8579 | 0.3415 | 0.9996 | |
| AAEL016841 | tRNA-Leu | 1.2305 | -1.8678 | 0.3416 | 0.9996 | |
| AAEL017081 | | -0.2201 | 8.3386 | 0.3419 | 0.9996 | |
| AAEL020963 | NA | -0.4782 | 12.4152 | 0.3420 | 0.9996 | NA |
| AAEL006413 | | -0.2867 | 3.7755 | 0.3421 | 0.9996 | ubiquitin-conjugating enzyme morgue [Source:VB Community Annotation] |
| AAEL001247 | | 0.2399 | 5.2875 | 0.3421 | 0.9996 | |
| AAEL007373 | | 0.9244 | 1.2188 | 0.3422 | 0.9996 | ammonium transporter [Source:VB Community Annotation] |
| AAEL008045 | | 1.5241 | 0.6784 | 0.3423 | 0.9996 | hexamerin 2 beta [Source:VB Community Annotation] |
| AAEL010620 | | -0.4019 | 4.6714 | 0.3424 | 0.9996 | |
| AAEL028662 | NA | -0.8746 | 0.5167 | 0.3425 | 0.9996 | NA |
| AAEL008628 | | -0.4046 | 4.2619 | 0.3425 | 0.9996 | ABC transporter [Source:VB Community Annotation] |
| AAEL000942 | | -0.2467 | 7.0460 | 0.3426 | 0.9996 | ER membrane protein complex subunit 4 [Source:UniProtKB/TrEMBL;Acc:Q17MQ7] |
| AAEL015006 | RpL23 | -0.3053 | 11.5540 | 0.3426 | 0.9996 | 60S ribosomal protein L23 (L17A)(AeRpL17A) [Source:VB Community Annotation] |
| AAEL024853 | NA | 0.2822 | 6.8111 | 0.3426 | 0.9996 | NA |
| AAEL012323 | | 0.3093 | 5.8425 | 0.3429 | 0.9996 | MSF1 protein, putative [Source:VB Community Annotation] |
| AAEL005929 | | -0.3174 | 3.3010 | 0.3430 | 0.9996 | ATP-binding cassette transporter [Source:VB Community Annotation] |
| AAEL001666 | | -1.0413 | 0.0684 | 0.3431 | 0.9996 | |
| AAEL010430 | | 0.2582 | 6.2286 | 0.3434 | 0.9996 | ras-related protein, putative [Source:VB Community Annotation] |
| AAEL012637 | | -0.3150 | 3.8662 | 0.3434 | 0.9996 | |
| AAEL024421 | NA | -1.4746 | 1.0454 | 0.3434 | 0.9996 | NA |
| AAEL012059 | | 0.4469 | 3.5965 | 0.3435 | 0.9996 | |

| | | | | | | |
|---|---|---|---|---|---|---|
| AAEL003681 | | -0.6399 | 1.0909 | 0.3435 | 0.9996 | |
| AAEL013760 | | -1.0912 | 0.1737 | 0.3435 | 0.9996 | homeobox protein nk-2 [Source:VB Community Annotation] |
| AAEL012375 | | 0.3177 | 4.8578 | 0.3436 | 0.9996 | |
| AAEL004686 | pont | 0.3773 | 3.6220 | 0.3438 | 0.9996 | RuvB-like helicase 1 (EC 3.6.4.12) (Pontin) [Source:VB Community Annotation] |
| AAEL007881 | | -0.4777 | 7.3778 | 0.3438 | 0.9996 | |
| AAEL007150 | | -0.3857 | 2.8675 | 0.3439 | 0.9996 | ATP-dependent RNA helicase [Source:VB Community Annotation] |
| AAEL018341 | | -1.3894 | -0.5486 | 0.3440 | 0.9996 | |
| AAEL011199 | | -0.3482 | 4.5123 | 0.3440 | 0.9996 | |
| AAEL003573 | | 0.4169 | 4.5973 | 0.3441 | 0.9996 | transportin [Source:VB Community Annotation] |
| AAEL023586 | NA | 0.4040 | 1.4609 | 0.3441 | 0.9996 | NA |
| AAEL004978 | | -0.3679 | 3.8595 | 0.3442 | 0.9996 | DEAD box ATP-dependent RNA helicase [Source:VB Community Annotation] |
| AAEL007947 | GSTE3 | -0.4001 | 4.1369 | 0.3442 | 0.9996 | glutathione transferase [Source:VB Community Annotation] |
| AAEL014702 | | 0.3970 | 6.6782 | 0.3444 | 0.9996 | lysyl-tRNA synthetase [Source:VB Community Annotation] |
| AAEL006908 | | 0.2907 | 3.4772 | 0.3444 | 0.9996 | |
| AAEL018163 | | -1.4767 | 0.8567 | 0.3445 | 0.9996 | |
| AAEL018117 | | 0.4847 | 5.6933 | 0.3446 | 0.9996 | |
| AAEL022995 | NA | -2.0875 | 1.8809 | 0.3446 | 0.9996 | NA |
| AAEL009242 | | 0.2956 | 3.0031 | 0.3447 | 0.9996 | |
| AAEL013616 | | -0.3213 | 3.6997 | 0.3448 | 0.9996 | |
| AAEL024423 | NA | -0.8825 | 0.4303 | 0.3448 | 0.9996 | NA |
| AAEL010261 | | 0.6066 | 2.0062 | 0.3450 | 0.9996 | |
| AAEL022405 | NA | 0.5356 | 1.7182 | 0.3451 | 0.9996 | NA |
| AAEL003924 | | 0.2355 | 6.5129 | 0.3451 | 0.9996 | |
| AAEL021145 | NA | -0.3178 | 4.3049 | 0.3452 | 0.9996 | NA |
| AAEL012086 | LRIM1 | -0.5576 | 7.8047 | 0.3452 | 0.9996 | leucine-rich immune protein (Long) [Source:VB Community Annotation] |
| AAEL013979 | | 0.2821 | 6.5866 | 0.3452 | 0.9996 | |
| AAEL017064 | | 0.3411 | 5.3001 | 0.3453 | 0.9996 | |
| AAEL011663 | | 0.2627 | 3.8381 | 0.3453 | 0.9996 | |
| AAEL011988 | | -0.2534 | 4.5922 | 0.3453 | 0.9996 | tRNA selenocysteine associated protein (secp43) [Source:VB Community Annotation] |
| AAEL009662 | | -0.3025 | 3.6800 | 0.3454 | 0.9996 | |
| AAEL014044 | | -0.5600 | 2.8441 | 0.3455 | 0.9996 | |
| AAEL007883 | | 0.3051 | 7.3319 | 0.3455 | 0.9996 | fk506-binding protein [Source:VB Community Annotation] |
| AAEL011637 | | 0.4007 | 2.8155 | 0.3456 | 0.9996 | fms interacting protein [Source:VB Community Annotation] |
| AAEL000856 | | 0.2835 | 3.7884 | 0.3457 | 0.9996 | germ cell-less protein [Source:VB Community Annotation] |
| AAEL002580 | | -0.7724 | 6.9356 | 0.3457 | 0.9996 | |
| AAEL008102 | | 0.5047 | 2.8671 | 0.3458 | 0.9996 | actin binding protein, putative [Source:VB Community Annotation] |
| AAEL002601 | CLIPA1 | -0.5726 | 4.4874 | 0.3458 | 0.9996 | Clip-Domain Serine Protease family A. Protease homologue. [Source:VB Community Annotation] |
| AAEL005721 | | 0.3242 | 3.7673 | 0.3461 | 0.9996 | |
| AAEL021755 | NA | 1.0422 | 0.0388 | 0.3461 | 0.9996 | NA |
| AAEL008942 | | -0.5491 | 5.9567 | 0.3464 | 0.9996 | |

| | | | | | | |
|---|---|---|---|---|---|---|
| AAEL015609 | | 0.8298 | 1.3490 | 0.3466 | 0.9996 | small calcium-binding mitochondrial carrier, putative [Source:VB Community Annotation] |
| AAEL006779 | | -0.2352 | 3.6618 | 0.3468 | 0.9996 | cytochrome c oxidase assembly protein cox11 [Source:VB Community Annotation] |
| AAEL023706 | NA | 0.4799 | 3.5968 | 0.3469 | 0.9996 | NA |
| AAEL009681 | | 0.6920 | 2.4275 | 0.3470 | 0.9996 | Rhomboid-like protein [Source:UniProtKB/TrEMBL;Acc:Q16V56] |
| AAEL007651 | | 0.3489 | 4.7279 | 0.3471 | 0.9996 | phosphorylase b kinase [Source:VB Community Annotation] |
| AAEL012525 | | 0.4831 | 2.7898 | 0.3472 | 0.9996 | YEATS domain containing protein 4 [Source:VB Community Annotation] |
| AAEL003034 | | 0.3768 | 3.0148 | 0.3474 | 0.9996 | |
| AAEL019463 | NA | -0.3109 | 5.2385 | 0.3475 | 0.9996 | NA |
| AAEL008931 | Kir2B | 1.1258 | 1.6540 | 0.3477 | 0.9996 | inward-rectifying potassium channel [Source:VB Community Annotation] |
| AAEL024045 | NA | 1.0657 | -0.9584 | 0.3477 | 0.9996 | NA |
| AAEL002861 | | 0.3375 | 5.1342 | 0.3478 | 0.9996 | |
| AAEL004913 | Arf3 | -0.5034 | 5.9080 | 0.3478 | 0.9996 | ADP-ribosylation factor 3 [Source:VB Community Annotation] |
| AAEL007790 | | -0.3300 | 4.4695 | 0.3478 | 0.9996 | maternal g10 transcript [Source:VB Community Annotation] |
| AAEL009686 | | 0.8791 | 2.2161 | 0.3479 | 0.9996 | red protein (ik factor) (cytokine ik) [Source:VB Community Annotation] |
| AAEL022496 | NA | -0.8712 | 0.4923 | 0.3482 | 0.9996 | NA |
| AAEL026823 | NA | 0.6059 | 1.0086 | 0.3483 | 0.9996 | NA |
| AAEL026412 | NA | 0.3395 | 4.5767 | 0.3483 | 0.9996 | NA |
| AAEL009995 | | 0.4863 | 2.5731 | 0.3485 | 0.9996 | transmembrane protein, putative [Source:VB Community Annotation] |
| AAEL006784 | CYP9J17 | 0.5757 | 1.4146 | 0.3486 | 0.9996 | cytochrome P450 [Source:VB Community Annotation] |
| AAEL010559 | | 0.3415 | 4.9241 | 0.3486 | 0.9996 | vacuolar protein sorting vps16 [Source:VB Community Annotation] |
| AAEL015651 | | 0.3888 | 5.9977 | 0.3486 | 0.9996 | Poly [ADP-ribose] polymerase [Source:UniProtKB/TrEMBL;Acc:Q1DGF7] |
| AAEL000182 | | -0.4106 | 11.2872 | 0.3486 | 0.9996 | |
| AAEL000832 | | 0.2904 | 4.6077 | 0.3487 | 0.9996 | cleavage and polyadenylation specificity factor cpsf [Source:VB Community Annotation] |
| AAEL001332 | | 0.3465 | 3.9725 | 0.3488 | 0.9996 | |
| AAEL011173 | | 1.0541 | -0.7996 | 0.3488 | 0.9996 | |
| AAEL017553 | CCEAE2B | 0.8111 | -0.6690 | 0.3489 | 0.9996 | Carboxy/choline esterase Alpha Esterase [Source:VB Community Annotation] |
| AAEL001410 | | 0.4171 | 3.1714 | 0.3489 | 0.9996 | snf2 histone linker phd ring helicase [Source:VB Community Annotation] |
| AAEL020753 | NA | -1.3693 | 6.1179 | 0.3490 | 0.9996 | NA |
| AAEL001378 | | 0.2835 | 4.9216 | 0.3491 | 0.9996 | spermine synthase [Source:VB Community Annotation] |
| AAEL005504 | | 0.3000 | 3.9355 | 0.3492 | 0.9996 | |
| AAEL011649 | | -0.8536 | 0.7387 | 0.3494 | 0.9996 | DNA polymerase theta [Source:VB Community Annotation] |
| AAEL014315 | | 0.5183 | 9.5955 | 0.3494 | 0.9996 | |
| AAEL012611 | | 0.3663 | 3.0530 | 0.3496 | 0.9996 | Putative s-m checkpoint control protein cid1 [Source:UniProtKB/TrEMBL;Acc:A0A0N8ERZ5] |
| AAEL019838 | NA | -0.8119 | 2.8852 | 0.3496 | 0.9996 | NA |
| AAEL000179 | | 0.2566 | 5.8687 | 0.3501 | 0.9996 | ubiquitin-conjugating enzyme E2 l [Source:VB Community Annotation] |
| AAEL026507 | NA | -1.0972 | -0.3539 | 0.3501 | 0.9996 | NA |
| AAEL000185 | | 0.2356 | 8.2509 | 0.3502 | 0.9996 | eukaryotic translation initiation factor [Source:VB Community Annotation] |
| AAEL012981 | | -1.3099 | 0.1037 | 0.3502 | 0.9996 | sugar transporter [Source:VB Community Annotation] |
| AAEL004523 | | 0.3331 | 7.2495 | 0.3504 | 0.9996 | preprotein translocase secy subunit (sec61) [Source:VB Community Annotation |
| AAEL024363 | NA | -1.0555 | -1.5045 | 0.3504 | 0.9996 | NA |

| | | | | | | |
|---|---|---|---|---|---|---|
| AAEL012393 | | -1.3445 | -0.3276 | 0.3505 | 0.9996 | |
| AAEL013314 | | 0.3480 | 4.0810 | 0.3507 | 0.9996 | calicylin binding protein [Source:VB Community Annotation] |
| AAEL006364 | | 0.3009 | 5.3658 | 0.3509 | 0.9996 | |
| AAEL002964 | | -0.3873 | 5.4762 | 0.3510 | 0.9996 | brain chitinase and chia [Source:VB Community Annotation] |
| AAEL003737 | | 0.3488 | 3.4708 | 0.3512 | 0.9996 | |
| AAEL012169 | | 0.8862 | 3.1234 | 0.3512 | 0.9996 | |
| AAEL020502 | NA | 0.4474 | 4.9327 | 0.3514 | 0.9996 | NA |
| AAEL010689 | | 0.5214 | 2.6802 | 0.3515 | 0.9996 | |
| AAEL003888 | | 0.3450 | 8.8194 | 0.3516 | 0.9996 | ubiquitin [Source:VB Community Annotation] |
| AAEL020864 | NA | -1.1742 | -1.8468 | 0.3516 | 0.9996 | NA |
| AAEL013879 | | 0.5494 | 3.1836 | 0.3517 | 0.9996 | |
| AAEL004764 | | 0.8265 | -0.4056 | 0.3518 | 0.9996 | pupal cuticle protein, putative [Source:VB Community Annotation] |
| AAEL006711 | | 0.5592 | 5.3006 | 0.3519 | 0.9996 | |
| AAEL014282 | | -0.2422 | 5.9255 | 0.3520 | 0.9996 | signalosome subunit [Source:VB Community Annotation] |
| AAEL008247 | | 0.7908 | -0.7162 | 0.3521 | 0.9996 | DNA polymerase alpha catalytic subunit [Source:VB Community Annotation] |
| AAEL002895 | | 0.2234 | 4.3202 | 0.3523 | 0.9996 | |
| AAEL026863 | NA | 1.2776 | -2.0254 | 0.3523 | 0.9996 | NA |
| AAEL010589 | | -0.9708 | 2.3551 | 0.3528 | 0.9996 | gamma-soluble nsf attachment protein (snap) [Source:VB Community Annotation] |
| AAEL004283 | | -0.9739 | 1.8853 | 0.3530 | 0.9996 | |
| AAEL027644 | NA | -0.4786 | 2.7744 | 0.3533 | 0.9996 | NA |
| AAEL000424 | | -0.2446 | 5.8570 | 0.3534 | 0.9996 | leucine aminopeptidase [Source:VB Community Annotation] |
| AAEL014864 | | 0.3918 | 1.6868 | 0.3535 | 0.9996 | |
| AAEL002673 | | 0.4239 | 5.1651 | 0.3535 | 0.9996 | elongase, putative [Source:VB Community Annotation] |
| AAEL001405 | | -0.2855 | 4.0889 | 0.3536 | 0.9996 | clathrin coat assembly protein [Source:VB Community Annotation] |
| AAEL003963 | | 1.2674 | 0.3392 | 0.3537 | 0.9996 | calpain 4, 6, 7, invertebrate [Source:VB Community Annotation] |
| AAEL013347 | | 0.5911 | 3.9812 | 0.3537 | 0.9996 | lethal(2)essential for life protein, l2efl [Source:VB Community Annotation] |
| AAEL008724 | | 0.8315 | -0.4246 | 0.3538 | 0.9996 | |
| AAEL026700 | NA | -0.2219 | 6.6887 | 0.3539 | 0.9996 | NA |
| AAEL001395 | | -0.4029 | 3.6015 | 0.3542 | 0.9996 | suppression of tumorigenicity [Source:VB Community Annotation] |
| AAEL019435 | NA | 0.7784 | 2.0761 | 0.3543 | 0.9996 | NA |
| AAEL012122 | | 0.3856 | 7.0088 | 0.3543 | 0.9996 | 26S proteasome regulatory subunit S3 [Source:VB Community Annotation] |
| AAEL013890 | | -0.3608 | 6.6184 | 0.3544 | 0.9996 | 26S proteasome non-ATPase regulatory subunit [Source:VB Community Annotation] |
| AAEL025704 | NA | -0.3660 | 3.7819 | 0.3546 | 0.9996 | NA |
| AAEL004075 | | -0.2753 | 3.3592 | 0.3546 | 0.9996 | RNA binding motif protein [Source:VB Community Annotation] |
| AAEL000146 | | -1.1864 | -2.2848 | 0.3547 | 0.9996 | nitrilase, putative [Source:VB Community Annotation] |
| AAEL002329 | | -0.8815 | 2.0060 | 0.3548 | 0.9996 | alpha-1,3-mannosyl-glycoprotein beta-1, 2-n-acetylglucosaminyltransferase [Source:VB Community Annotation] |
| AAEL026672 | NA | 0.3812 | 2.9887 | 0.3549 | 0.9996 | NA |
| AAEL011787 | | 0.3028 | 3.4543 | 0.3551 | 0.9996 | |
| AAEL003926 | | -0.3778 | 3.6484 | 0.3553 | 0.9996 | GTP-binding protein lepa [Source:VB Community Annotation] |
| AAEL025664 | NA | 0.5811 | 1.7825 | 0.3554 | 0.9996 | NA |

| | | | | | | |
|---|---|---|---|---|---|---|
| AAEL018150 | | -0.2967 | 4.6479 | 0.3555 | 0.9996 | Putative sodium/solute symporter [Source:UniProtKB/TrEMBL;Acc:A0A0P6IYV1] |
| AAEL024391 | NA | -1.2099 | -2.5121 | 0.3555 | 0.9996 | NA |
| AAEL013142 | | 0.5258 | 2.0686 | 0.3556 | 0.9996 | |
| AAEL010246 | | 0.2542 | 4.4981 | 0.3557 | 0.9996 | |
| AAEL019570 | NA | 0.4375 | 1.9159 | 0.3558 | 0.9996 | NA |
| AAEL022671 | NA | 0.6676 | 0.7345 | 0.3558 | 0.9996 | NA |
| AAEL013547 | | 0.4157 | 2.6031 | 0.3558 | 0.9996 | |
| AAEL026775 | NA | -1.0050 | -0.6892 | 0.3560 | 0.9996 | NA |
| AAEL007895 | | -0.2672 | 3.9701 | 0.3561 | 0.9996 | beta-1,4-galactosyltransferase [Source:VB Community Annotation] |
| AAEL001476 | | -0.3450 | 3.2727 | 0.3561 | 0.9996 | |
| AAEL012134 | | -0.7780 | 8.1870 | 0.3562 | 0.9996 | pur-alpha [Source:VB Community Annotation] |
| AAEL014823 | | 1.2176 | 0.4763 | 0.3562 | 0.9996 | |
| AAEL021857 | NA | 0.5659 | 1.7334 | 0.3565 | 0.9996 | NA |
| AAEL020112 | NA | 0.4880 | 4.7811 | 0.3566 | 0.9996 | NA |
| AAEL012861 | | 0.3002 | 3.9028 | 0.3568 | 0.9996 | |
| AAEL002194 | | 0.7508 | 4.7009 | 0.3568 | 0.9996 | Uricase [Source:UniProtKB/TrEMBL;Acc:Q17J02] |
| AAEL010773 | CLIPE10 | -0.7154 | 5.5181 | 0.3568 | 0.9996 | Clip-Domain Serine Protease  family E. Protease homologue. [Source:VB Community Annotation] |
| AAEL021093 | NA | 0.5233 | 2.2557 | 0.3569 | 0.9996 | NA |
| AAEL016602 | tRNA-Arg | -0.6736 | 0.9252 | 0.3570 | 0.9996 | |
| AAEL012154 | | -0.7495 | 2.8034 | 0.3571 | 0.9996 | 2,3-cyclic-nucleotide 2-phosphodiesterase [Source:VB Community Annotation] |
| AAEL014613 | CYP9J24 | -1.1839 | 0.0560 | 0.3574 | 0.9996 | cytochrome P450 [Source:VB Community Annotation] |
| AAEL000173 | | -0.3634 | 3.7850 | 0.3574 | 0.9996 | chromatin assembly factor-I p150 subunit, putative [Source:VB Community Annotation] |
| AAEL021793 | NA | -0.6356 | 2.9402 | 0.3575 | 0.9996 | NA |
| AAEL009284 | | -0.5402 | 0.5942 | 0.3576 | 0.9996 | centromere protein-A, putative [Source:VB Community Annotation] |
| AAEL012955 | | -0.6419 | 4.7206 | 0.3577 | 0.9996 | phosphatidylethanolamine-binding protein [Source:VB Community Annotation] |
| AAEL024636 | NA | 0.6217 | 2.2757 | 0.3577 | 0.9996 | NA |
| AAEL011874 | | 0.5055 | 2.6494 | 0.3577 | 0.9996 | |
| AAEL012227 | | -0.4308 | 4.5366 | 0.3578 | 0.9996 | |
| AAEL013596 | | -0.7264 | 5.9701 | 0.3578 | 0.9996 | phosphatidylinositol 3-kinase regulatory subunit [Source:VB Community Annotation] |
| AAEL024757 | NA | 0.6443 | 0.0064 | 0.3578 | 0.9996 | NA |
| AAEL008884 | | 1.3964 | -1.3358 | 0.3580 | 0.9996 | |
| AAEL022976 | NA | -1.1035 | -1.8104 | 0.3582 | 0.9996 | NA |
| AAEL013808 | | 0.4079 | 6.1300 | 0.3584 | 0.9996 | Fascin [Source:UniProtKB/TrEMBL;Acc:Q16I34] |
| AAEL000824 | | -0.2615 | 6.3230 | 0.3584 | 0.9996 | |
| AAEL008961 | | -0.6878 | 3.5910 | 0.3585 | 0.9996 | |
| AAEL003950 | | 0.8780 | -1.0869 | 0.3585 | 0.9996 | helicase [Source:VB Community Annotation] |
| AAEL004512 | | 0.7676 | 0.9241 | 0.3587 | 0.9996 | zinc finger protein [Source:VB Community Annotation] |
| AAEL012382 | | -0.3675 | 5.3002 | 0.3587 | 0.9996 | |
| AAEL003361 | | 0.7315 | 0.9269 | 0.3590 | 0.9996 | |
| AAEL011790 | | 0.3740 | 4.7784 | 0.3592 | 0.9996 | |

| | | | | | | |
|---|---|---|---|---|---|---|
| AAEL004947 | | 1.0129 | -0.0739 | 0.3593 | 0.9996 | elongase, putative [Source:VB Community Annotation] |
| AAEL026826 | NA | 0.7877 | 0.0259 | 0.3594 | 0.9996 | NA |
| AAEL019870 | NA | -0.5556 | 3.7328 | 0.3597 | 0.9996 | NA |
| AAEL021222 | NA | -0.7770 | 1.2187 | 0.3601 | 0.9996 | NA |
| AAEL021871 | NA | 0.4130 | 2.6194 | 0.3601 | 0.9996 | NA |
| AAEL000658 | | -0.8271 | 0.9524 | 0.3601 | 0.9996 | |
| AAEL006091 | | 0.3062 | 4.9518 | 0.3602 | 0.9996 | rab6 [Source:VB Community Annotation] |
| AAEL023941 | NA | 0.3558 | 4.9991 | 0.3606 | 0.9996 | NA |
| AAEL024655 | NA | 0.2768 | 6.6419 | 0.3607 | 0.9996 | NA |
| AAEL002775 | | 0.3310 | 3.0886 | 0.3609 | 0.9996 | |
| AAEL008557 | | 0.4126 | 3.6118 | 0.3613 | 0.9996 | |
| AAEL002083 | | 0.2716 | 9.3369 | 0.3614 | 0.9996 | DEAD box ATP-dependent RNA helicase [Source:VB Community Annotation] |
| AAEL001820 | | 0.8197 | 0.0687 | 0.3619 | 0.9996 | |
| AAEL011088 | Dat | 0.2207 | 6.4897 | 0.3620 | 0.9996 | dopamine N-acetyltransferase [Source:VB Community Annotation] |
| AAEL013421 | | -1.7002 | -0.3123 | 0.3620 | 0.9996 | Alpha-amylase [Source:UniProtKB/TrEMBL;Acc:Q16J70] |
| AAEL003425 | | -0.5681 | 6.6237 | 0.3621 | 0.9996 | |
| AAEL001037 | | -0.3864 | 5.6110 | 0.3622 | 0.9996 | ribosomal RNA methyltransferase [Source:VB Community Annotation] |
| AAEL002486 | | 0.3356 | 5.2552 | 0.3622 | 0.9996 | mitochondrial inner membrane protein translocase, 9kD-subunit, putative [Source:VB Community Annotation] |
| AAEL025927 | NA | -0.2768 | 4.0379 | 0.3622 | 0.9996 | NA |
| AAEL014161 | | -0.3126 | 4.3819 | 0.3623 | 0.9996 | amino acids transporter [Source:VB Community Annotation] |
| AAEL004738 | | -0.2917 | 6.5973 | 0.3624 | 0.9996 | Methionine aminopeptidase 2 [Source:UniProtKB/TrEMBL;Acc:Q17BZ1] |
| AAEL013684 | | -0.4810 | 7.3740 | 0.3625 | 0.9996 | |
| AAEL004043 | | -0.3805 | 3.9851 | 0.3625 | 0.9996 | |
| AAEL017023 | | -0.7251 | 6.5859 | 0.3625 | 0.9996 | |
| AAEL003959 | | -0.5092 | 3.1620 | 0.3626 | 0.9996 | short-chain dehydrogenase [Source:VB Community Annotation] |
| AAEL021566 | NA | 0.8235 | 0.6030 | 0.3626 | 0.9996 | NA |
| AAEL006367 | | 0.4769 | 2.6378 | 0.3627 | 0.9996 | |
| AAEL012110 | | -0.2886 | 8.9590 | 0.3627 | 0.9996 | protease m1 zinc metalloprotease [Source:VB Community Annotation] |
| AAEL000544 | | -0.3644 | 9.4830 | 0.3629 | 0.9996 | |
| AAEL011071 | | 0.4283 | 3.8710 | 0.3631 | 0.9996 | U2 snrnp auxiliary factor, small subunit [Source:VB Community Annotation] |
| AAEL004809 | | -0.5954 | 7.2283 | 0.3632 | 0.9996 | |
| AAEL006606 | | -0.7522 | 0.4162 | 0.3634 | 0.9996 | |
| AAEL005358 | | 0.3196 | 4.0161 | 0.3634 | 0.9996 | |
| AAEL000109 | | 1.3994 | 0.5963 | 0.3634 | 0.9996 | Enolase-phosphatase E1 (EC 3.1.3.77)(2,3-diketo-5-methylthio-1-phosphopentane phosphatase) [Source:VB Community Annotation] |
| AAEL006724 | | 0.2073 | 3.9328 | 0.3636 | 0.9996 | |
| AAEL001061 | GSTD1 | 0.3016 | 7.9242 | 0.3637 | 0.9996 | glutathione S-transferase (GSTD1) [Source:VB Community Annotation] |
| AAEL014371 | | -0.7684 | 2.4512 | 0.3638 | 0.9996 | glucosyl/glucuronosyl transferases [Source:VB Community Annotation] |
| AAEL002725 | | -1.0054 | -1.2109 | 0.3638 | 0.9996 | |
| AAEL015593 | | 0.3542 | 5.6732 | 0.3639 | 0.9996 | |
| AAEL020654 | NA | 0.3935 | 5.1973 | 0.3640 | 0.9996 | NA |

| | | | | | | |
|---|---|---|---|---|---|---|
| AAEL026039 | NA | 0.4159 | 2.5444 | 0.3640 | 0.9996 | NA |
| AAEL010853 | | 0.9982 | 0.9241 | 0.3641 | 0.9996 | AMP dependent ligase [Source:VB Community Annotation] |
| AAEL010743 | | -0.2865 | 6.9475 | 0.3641 | 0.9996 | cysteine desulfurylase [Source:VB Community Annotation] |
| AAEL022162 | NA | -0.6913 | 2.8550 | 0.3641 | 0.9996 | NA |
| AAEL005914 | | 0.2233 | 6.0649 | 0.3645 | 0.9996 | |
| AAEL010378 | | 0.4425 | 1.1320 | 0.3647 | 0.9996 | |
| AAEL005163 | | 0.3982 | 4.6286 | 0.3647 | 0.9996 | 60S ribosome subunit biogenesis protein NIP7 homolog [Source:UniProtKB/TrEMBL;Acc:Q17AX1] |
| AAEL011388 | | -0.3497 | 3.7432 | 0.3648 | 0.9996 | Vacuolar-sorting protein SNF8 [Source:UniProtKB/TrEMBL;Acc:Q16Q65] |
| AAEL013814 | | 0.4226 | 3.6411 | 0.3649 | 0.9996 | phosphomevalonate kinase, putative [Source:VB Community Annotation] |
| AAEL019624 | NA | 0.2666 | 5.0601 | 0.3649 | 0.9996 | NA |
| AAEL009823 | | 0.3523 | 5.0200 | 0.3649 | 0.9996 | |
| AAEL013110 | | 0.8803 | -0.4841 | 0.3651 | 0.9996 | |
| AAEL027675 | NA | 1.4618 | -1.2218 | 0.3652 | 0.9996 | NA |
| AAEL024413 | NA | 0.5219 | 2.8756 | 0.3652 | 0.9996 | NA |
| AAEL012138 | | -0.3197 | 5.1213 | 0.3653 | 0.9996 | 24-dehydrocholesterol reductase [Source:VB Community Annotation] |
| AAEL000941 | | 0.2867 | 7.4025 | 0.3655 | 0.9996 | mitochondrial inner membrane protein translocase, 8kD-subunit, putative [Source:VB Community Annotation] |
| AAEL009177 | | 0.6590 | 1.4296 | 0.3655 | 0.9996 | |
| AAEL007914 | | -1.6368 | 1.5621 | 0.3655 | 0.9996 | discs large protein [Source:VB Community Annotation] |
| AAEL012886 | | -0.3581 | 5.1012 | 0.3655 | 0.9996 | Carboxylic ester hydrolase (Fragment) [Source:UniProtKB/TrEMBL;Acc:Q16KS8] |
| AAEL002384 | | -0.2864 | 5.1309 | 0.3656 | 0.9996 | |
| AAEL020915 | NA | 0.2271 | 4.9740 | 0.3658 | 0.9996 | NA |
| AAEL011667 | | 0.3127 | 4.6820 | 0.3658 | 0.9996 | cir [Source:VB Community Annotation] |
| AAEL002828 | | 0.2907 | 4.0151 | 0.3658 | 0.9996 | |
| AAEL008370 | SCRB17 | 0.2460 | 5.6973 | 0.3658 | 0.9996 | Class B Scavenger Receptor (CD36 domain). [Source:VB Community Annotation |
| AAEL007364 | | 0.4659 | 3.5204 | 0.3659 | 0.9996 | 7,8-dihydro-8-oxoguanine-triphosphatase, putative [Source:VB Community Annotation] |
| AAEL010326 | | 0.4801 | 2.5825 | 0.3659 | 0.9996 | Phosphotriesterase-related protein (EC 3.1.-.-)(Parathion hydrolase-related protein) [Source:VB Community Annotation] |
| AAEL020769 | NA | 0.4486 | 3.2460 | 0.3660 | 0.9996 | NA |
| AAEL008174 | | -1.1045 | -0.6612 | 0.3662 | 0.9996 | |
| AAEL019448 | NA | -1.0006 | 0.6770 | 0.3662 | 0.9996 | NA |
| AAEL003481 | | 0.5884 | 2.5392 | 0.3668 | 0.9996 | Glycosyltransferase 25 family member Precursor (EC 2.-.-.-) [Source:VB Community Annotation] |
| AAEL001268 | | 1.1312 | -0.7951 | 0.3669 | 0.9996 | |
| AAEL010769 | SRPN6 | 0.2900 | 5.3539 | 0.3670 | 0.9996 | Serine Protease Inhibitor (serpin) likely cleavage at S/A. [Source:VB Community Annotation] |
| AAEL019483 | NA | -1.6837 | -0.4157 | 0.3670 | 0.9996 | NA |
| AAEL009148 | | -1.1035 | -1.5664 | 0.3671 | 0.9996 | |
| AAEL004217 | | 0.4636 | 3.6032 | 0.3673 | 0.9996 | |
| AAEL026165 | NA | -1.2858 | 7.3535 | 0.3674 | 0.9996 | NA |
| AAEL010781 | | -0.4608 | 2.8512 | 0.3675 | 0.9996 | ap endonuclease [Source:VB Community Annotation] |
| AAEL002190 | | 0.2211 | 4.7999 | 0.3676 | 0.9996 | |
| AAEL004562 | | 0.4889 | 0.9640 | 0.3676 | 0.9996 | DNA polymerase eta [Source:VB Community Annotation] |
| AAEL004500 | | 0.3046 | 10.5567 | 0.3676 | 0.9996 | eukaryotic translation elongation factor [Source:VB Community Annotation] |

| | | | | | | |
|---|---|---|---|---|---|---|
| AAEL004779 | | 0.3551 | 3.4329 | 0.3681 | 0.9996 | cyclophilin-10 [Source:VB Community Annotation] |
| AAEL019427 | NA | 0.6804 | 2.0864 | 0.3685 | 0.9996 | NA |
| AAEL006601 | | 0.3696 | 3.7501 | 0.3688 | 0.9996 | 50-kda dystrophin-associated glycoprotein, putative [Source:VB Community Annotation] |
| AAEL024991 | NA | 0.5415 | 0.9959 | 0.3690 | 0.9996 | NA |
| AAEL017811 | RNase_MR | -0.4817 | 5.4333 | 0.3692 | 0.9996 | RNase MRP [Source:RFAM;Acc:RF00030] |
| AAEL008412 | | -0.4890 | 4.0357 | 0.3692 | 0.9996 | Mitochondrial import inner membrane translocase subunit TIM44 [Source:UniProtKB/TrEMBL;Acc:Q16YW1] |
| AAEL004552 | | 0.3726 | 2.3920 | 0.3694 | 0.9996 | |
| AAEL007935 | Gr20 | -0.8362 | 0.7394 | 0.3695 | 0.9996 | gustatory receptor Gr20 [Source:VB Community Annotation] |
| AAEL003589 | | -2.7164 | 1.2027 | 0.3696 | 0.9996 | transcription factor, putative [Source:VB Community Annotation] |
| AAEL005737 | | 0.7948 | -0.3304 | 0.3697 | 0.9996 | |
| AAEL026406 | NA | 0.3167 | 5.0992 | 0.3702 | 0.9996 | NA |
| AAEL024358 | NA | -0.6717 | 1.7490 | 0.3704 | 0.9996 | NA |
| AAEL002134 | | 0.6104 | 2.2490 | 0.3704 | 0.9996 | |
| AAEL024884 | NA | -1.0300 | 0.1708 | 0.3704 | 0.9996 | NA |
| AAEL014893 | CYP6BB2 | 0.6011 | 7.5255 | 0.3704 | 0.9996 | cytochrome P450 [Source:VB Community Annotation] |
| AAEL005827 | | -1.5235 | 0.4782 | 0.3704 | 0.9996 | katanin P80 subunit [Source:VB Community Annotation] |
| AAEL022068 | NA | 0.2741 | 4.2568 | 0.3706 | 0.9996 | NA |
| AAEL013509 | | 0.2738 | 4.9133 | 0.3709 | 0.9996 | membralin [Source:VB Community Annotation] |
| AAEL021795 | NA | 0.9040 | 6.7089 | 0.3710 | 0.9996 | NA |
| AAEL012326 | | -0.5531 | 11.6649 | 0.3710 | 0.9996 | calmodulin [Source:VB Community Annotation] |
| AAEL009969 | | 0.6846 | 1.0484 | 0.3712 | 0.9996 | axonemal dynein light chain [Source:VB Community Annotation] |
| AAEL014253 | | 0.4405 | 3.4970 | 0.3713 | 0.9996 | |
| AAEL022712 | NA | -0.3519 | 5.9812 | 0.3713 | 0.9996 | NA |
| AAEL007853 | | 0.3354 | 3.0645 | 0.3714 | 0.9996 | lupus la protein (Sjogren syndrome type B antigen) (La/SS-B), putative [Source:VB Community Annotation] |
| AAEL002832 | RpS26 | 0.3065 | 11.5407 | 0.3715 | 0.9996 | 40S ribosomal protein S26 [Source:UniProtKB/TrEMBL;Acc:Q1HQE4] |
| AAEL015283 | | -0.9893 | -1.2874 | 0.3715 | 0.9996 | synaptic vesicle protein [Source:VB Community Annotation] |
| AAEL004196 | GALE3 | -0.2727 | 4.4192 | 0.3717 | 0.9996 | galectin [Source:VB Community Annotation] |
| AAEL004765 | | -0.5846 | 1.5189 | 0.3718 | 0.9996 | pupal cuticle protein, putative [Source:VB Community Annotation] |
| AAEL013964 | RpL20 | -0.2776 | 6.6662 | 0.3719 | 0.9996 | 60S ribosomal protein L20, putative [Source:VB Community Annotation] |
| AAEL022140 | NA | -0.3400 | 13.0320 | 0.3719 | 0.9996 | NA |
| AAEL004958 | | 0.7337 | 3.3048 | 0.3719 | 0.9996 | |
| AAEL007856 | | -0.4805 | 1.0947 | 0.3719 | 0.9996 | crumbs [Source:VB Community Annotation] |
| AAEL017317 | | 0.3347 | 5.1446 | 0.3720 | 0.9996 | |
| AAEL017061 | | -0.2999 | 5.7003 | 0.3720 | 0.9996 | |
| AAEL006922 | | -1.3455 | 3.9347 | 0.3720 | 0.9996 | calponin/transgelin [Source:VB Community Annotation] |
| AAEL011397 | | 0.6974 | 0.1777 | 0.3723 | 0.9996 | |
| AAEL006227 | | -0.3738 | 4.9553 | 0.3723 | 0.9996 | |
| AAEL025728 | NA | -0.9929 | 0.3742 | 0.3724 | 0.9996 | NA |
| AAEL008641 | | -0.5880 | 6.8690 | 0.3724 | 0.9996 | GTP-binding  protein (o) alpha subunit, gnao [Source:VB Community Annotation] |
| AAEL010834 | | 0.2282 | 4.1726 | 0.3724 | 0.9996 | arf GTPase-activating protein [Source:VB Community Annotation] |

| | | | | | | |
|---|---|---|---|---|---|---|
| AAEL000820 | | 1.0838 | -1.0068 | 0.3728 | 0.9996 | dimethylaniline monooxygenase [Source:VB Community Annotation] |
| AAEL023386 | NA | 0.3559 | 3.0558 | 0.3729 | 0.9996 | NA |
| AAEL001716 | | 0.2290 | 4.5827 | 0.3730 | 0.9996 | THO complex subunit 2 (Tho2) [Source:VB Community Annotation] |
| AAEL002636 | | -0.4459 | 2.8335 | 0.3732 | 0.9996 | |
| AAEL005313 | | 0.3154 | 5.6249 | 0.3734 | 0.9996 | |
| AAEL002592 | | 0.4606 | 4.6056 | 0.3735 | 0.9996 | |
| AAEL006032 | | 1.7476 | 2.1588 | 0.3736 | 0.9996 | |
| AAEL001382 | | -0.3396 | 5.5180 | 0.3737 | 0.9996 | small nuclear ribonucleoprotein sm d1 [Source:VB Community Annotation] |
| AAEL002809 | | -0.3955 | 4.3095 | 0.3737 | 0.9996 | down syndrome critical region protein [Source:VB Community Annotation] |
| AAEL019633 | NA | -0.5996 | 2.8300 | 0.3739 | 0.9996 | NA |
| AAEL014096 | | 0.3899 | 0.9678 | 0.3739 | 0.9996 | |
| AAEL008135 | Cog3 | 0.3759 | 5.2691 | 0.3743 | 0.9996 | Conserved oligomeric Golgi complex subunit 3 (COG complex subunit 3)(Component of oligomeric Golgi complex 3) [Source:VB Commun |
| AAEL009547 | | 0.7823 | 2.7739 | 0.3743 | 0.9996 | NAD dehydrogenase [Source:VB Community Annotation] |
| AAEL008596 | SPZ3A | -0.3078 | 4.5239 | 0.3745 | 0.9996 | spaetzle-like cytokine [Source:VB Community Annotation] |
| AAEL022645 | NA | 0.4356 | 2.4533 | 0.3746 | 0.9996 | NA |
| AAEL010883 | | 0.2094 | 4.7979 | 0.3746 | 0.9996 | sodium/chloride dependent transporter [Source:VB Community Annotation] |
| AAEL000431 | | 0.3722 | 7.5449 | 0.3747 | 0.9996 | |
| AAEL000327 | E78 | 0.3110 | 3.5937 | 0.3747 | 0.9996 | Ecdysone-induced protein 78C Nuclear receptor [Source:VB Community Annotation] |
| AAEL008184 | | 0.3393 | 3.8546 | 0.3748 | 0.9996 | |
| AAEL014291 | | 0.5959 | 0.5048 | 0.3750 | 0.9996 | |
| AAEL011738 | | 0.3902 | 3.1297 | 0.3751 | 0.9996 | |
| AAEL004955 | | 0.7612 | 0.9106 | 0.3754 | 0.9996 | |
| AAEL001756 | | 0.3111 | 4.2277 | 0.3754 | 0.9996 | anaphase-promoting complex subunit [Source:VB Community Annotation] |
| AAEL013496 | PPO8 | 1.3609 | 2.1465 | 0.3754 | 0.9996 | prophenoloxidase [Source:VB Community Annotation] |
| AAEL003886 | | 1.0349 | -0.1863 | 0.3756 | 0.9996 | |
| AAEL005803 | | -0.3838 | 2.0270 | 0.3757 | 0.9996 | |
| AAEL010018 | | -0.4606 | 8.2505 | 0.3757 | 0.9996 | |
| AAEL022445 | NA | -1.4422 | 1.1976 | 0.3760 | 0.9996 | NA |
| AAEL014326 | | 0.3188 | 4.7632 | 0.3760 | 0.9996 | out at first protein [Source:VB Community Annotation] |
| AAEL021441 | NA | -1.2099 | -1.9336 | 0.3760 | 0.9996 | NA |
| AAEL004212 | | 0.4411 | 5.2722 | 0.3760 | 0.9996 | |
| AAEL012762 | CYP325N2 | -0.4190 | 3.3561 | 0.3761 | 0.9996 | cytochrome P450 [Source:VB Community Annotation] |
| AAEL012352 | | -0.4859 | 5.9802 | 0.3761 | 0.9996 | |
| AAEL006445 | | -0.3365 | 2.8205 | 0.3762 | 0.9996 | Phosphatidylinositol-glycan biosynthesis class W protein [Source:UniProtKB/TrEMBL;Acc:Q176D1] |
| AAEL005999 | Or2 | 1.1016 | -0.8194 | 0.3763 | 0.9996 | odorant receptor [Source:VB Community Annotation] |
| AAEL000546 | | 0.5374 | 2.9661 | 0.3763 | 0.9996 | Carboxylic ester hydrolase (Fragment) [Source:UniProtKB/TrEMBL;Acc:Q17NX5] |
| AAEL021602 | NA | 0.4718 | 3.7395 | 0.3764 | 0.9996 | NA |
| AAEL021931 | NA | -2.6718 | 2.7734 | 0.3765 | 0.9996 | NA |
| AAEL011812 | | 0.4819 | 2.0378 | 0.3765 | 0.9996 | gonadotropin inducible transcription factor [Source:VB Community Annotation |
| AAEL020929 | NA | 0.4170 | 4.1937 | 0.3766 | 0.9996 | NA |

| | | | | | | |
|---|---|---|---|---|---|---|
| AAEL019831 | NA | 0.4005 | 1.6842 | 0.3766 | 0.9996 | NA |
| AAEL019857 | NA | -0.6145 | 3.6457 | 0.3766 | 0.9996 | NA |
| AAEL009704 | | 0.3442 | 2.7188 | 0.3766 | 0.9996 | |
| AAEL008007 | | -0.4844 | 4.9298 | 0.3770 | 0.9996 | |
| AAEL027145 | NA | 0.8434 | -0.5750 | 0.3770 | 0.9996 | NA |
| AAEL004490 | | -0.2983 | 5.1535 | 0.3770 | 0.9996 | |
| AAEL007173 | | 0.2792 | 6.2696 | 0.3771 | 0.9996 | |
| AAEL012993 | | 0.3625 | 5.5943 | 0.3771 | 0.9996 | |
| AAEL025711 | NA | 0.5432 | 0.2746 | 0.3771 | 0.9996 | NA |
| AAEL003879 | | -0.2894 | 3.1873 | 0.3773 | 0.9996 | |
| AAEL020145 | NA | 1.5828 | -1.1122 | 0.3774 | 0.9996 | NA |
| AAEL023223 | NA | -0.8911 | 8.1516 | 0.3776 | 0.9996 | NA |
| AAEL007253 | | 1.0584 | -0.1659 | 0.3776 | 0.9996 | |
| AAEL009632 | | 0.7019 | 1.2032 | 0.3776 | 0.9996 | short-chain dehydrogenase [Source:VB Community Annotation] |
| AAEL026028 | NA | -0.5587 | 3.1312 | 0.3779 | 0.9996 | NA |
| AAEL012528 | | 0.7419 | 2.0813 | 0.3780 | 0.9996 | tetraspanin, putative [Source:VB Community Annotation] |
| AAEL011634 | | -1.3433 | 1.5246 | 0.3780 | 0.9996 | fibrinogen and fibronectin [Source:VB Community Annotation] |
| AAEL000815 | | -0.7271 | 1.7206 | 0.3781 | 0.9996 | |
| AAEL011425 | | -0.3200 | 6.0782 | 0.3782 | 0.9996 | structure-specific recognition protein [Source:VB Community Annotation] |
| AAEL001794 | | -0.6124 | 7.3907 | 0.3784 | 0.9996 | macroglobulin/complement [Source:VB Community Annotation] |
| AAEL011680 | GPRNNA9 | 0.9961 | -0.0267 | 0.3784 | 0.9996 | GPCR Orphan/Putative Class A Family [Source:VB Community Annotation] |
| AAEL002255 | | -0.7885 | 4.3682 | 0.3786 | 0.9996 | |
| AAEL027751 | NA | 1.4998 | 0.8510 | 0.3789 | 0.9996 | NA |
| AAEL009287 | | 0.2911 | 7.2539 | 0.3790 | 0.9996 | ran [Source:VB Community Annotation] |
| AAEL023497 | NA | 0.4692 | 6.1050 | 0.3790 | 0.9996 | NA |
| AAEL025827 | NA | -0.6153 | 0.2295 | 0.3791 | 0.9996 | NA |
| AAEL020741 | NA | -1.4419 | 0.9509 | 0.3793 | 0.9996 | NA |
| AAEL001561 | | -0.4507 | 4.9774 | 0.3794 | 0.9996 | |
| AAEL005271 | | 0.2319 | 5.5479 | 0.3795 | 0.9996 | |
| AAEL027910 | NA | 0.2427 | 4.6266 | 0.3795 | 0.9996 | NA |
| AAEL008365 | | -0.4816 | 7.4249 | 0.3795 | 0.9996 | |
| AAEL004193 | | -0.3038 | 4.2114 | 0.3796 | 0.9996 | rhophilin [Source:VB Community Annotation] |
| AAEL024535 | NA | -1.4699 | 1.6936 | 0.3796 | 0.9996 | NA |
| AAEL019594 | NA | -0.7688 | 4.1315 | 0.3796 | 0.9996 | NA |
| AAEL009259 | | 0.2067 | 4.6859 | 0.3797 | 0.9996 | |
| AAEL010037 | | 0.3762 | 6.3719 | 0.3798 | 0.9996 | phosphoglucomutase [Source:VB Community Annotation] |
| AAEL026867 | NA | 0.2564 | 8.1452 | 0.3798 | 0.9996 | NA |
| AAEL012830 | | 0.2732 | 3.3321 | 0.3798 | 0.9996 | anti-silencing protein [Source:VB Community Annotation] |
| AAEL028043 | NA | -0.2749 | 9.7984 | 0.3800 | 0.9996 | NA |
| AAEL011765 | | 0.5233 | 1.7778 | 0.3800 | 0.9996 | |

| | | | | | | |
|---|---|---|---|---|---|---|
| AAEL001604 | | 0.3058 | 5.3662 | 0.3800 | 0.9996 | guanine nucleotide exchange factor [Source:VB Community Annotation] |
| AAEL002391 | | -0.4850 | 1.4590 | 0.3802 | 0.9996 | Carboxylic ester hydrolase (Fragment) [Source:UniProtKB/TrEMBL;Acc:Q17IF9] |
| AAEL009814 | | 0.3198 | 4.2228 | 0.3802 | 0.9996 | autophagy protein [Source:VB Community Annotation] |
| AAEL003235 | | -1.2282 | 4.5396 | 0.3802 | 0.9996 | |
| AAEL011468 | | -0.3316 | 7.8942 | 0.3803 | 0.9996 | protein kinase C inhibitor, putative [Source:VB Community Annotation] |
| AAEL006525 | | 0.6615 | 2.2696 | 0.3804 | 0.9996 | kelch repeat protein [Source:VB Community Annotation] |
| AAEL022540 | NA | 0.3623 | 3.9770 | 0.3805 | 0.9996 | NA |
| AAEL014148 | Dredd | 0.3748 | 2.4429 | 0.3805 | 0.9996 | caspase (long) [Source:VB Community Annotation] |
| AAEL009127 | CYP6M11 | 0.4811 | 3.3518 | 0.3805 | 0.9996 | cytochrome P450 [Source:VB Community Annotation] |
| AAEL007788 | | -0.3361 | 3.5203 | 0.3807 | 0.9996 | |
| AAEL011099 | | 0.2366 | 7.5813 | 0.3807 | 0.9996 | molybdopterin-binding [Source:VB Community Annotation] |
| AAEL023342 | NA | -0.5110 | 2.1604 | 0.3809 | 0.9996 | NA |
| AAEL006494 | Gr45 | 1.1395 | -0.7505 | 0.3809 | 0.9996 | gustatory receptor (Gr45) [Source:VB Community Annotation] |
| AAEL008664 | | 0.6066 | 5.2797 | 0.3809 | 0.9996 | |
| AAEL001931 | | -0.3091 | 4.5945 | 0.3810 | 0.9996 | o-sialoglycoprotein endopeptidase [Source:VB Community Annotation] |
| AAEL009276 | | -0.5649 | 0.8223 | 0.3810 | 0.9996 | |
| AAEL006965 | | -0.5026 | 2.1348 | 0.3811 | 0.9996 | NBP2b protein, putative [Source:VB Community Annotation] |
| AAEL005261 | | 0.2976 | 5.9288 | 0.3811 | 0.9996 | |
| AAEL023795 | NA | -0.5805 | 0.5118 | 0.3811 | 0.9996 | NA |
| AAEL014744 | | -0.8602 | 0.3985 | 0.3812 | 0.9996 | |
| AAEL003415 | | -0.2753 | 4.0708 | 0.3813 | 0.9996 | lamin [Source:VB Community Annotation] |
| AAEL013305 | | -0.2928 | 4.3600 | 0.3813 | 0.9996 | bifunctional dihydrofolate reductase-thymidylate synthase [Source:VB Community Annotation] |
| AAEL004404 | | -0.3004 | 8.7501 | 0.3813 | 0.9996 | HIG1 domain family member 2A, putative [Source:VB Community Annotation] |
| AAEL012845 | | -0.2468 | 6.8471 | 0.3814 | 0.9996 | Mitochondrial import inner membrane translocase subunit TIM44 [Source:UniProtKB/TrEMBL;Acc:Q16KX3] |
| AAEL002089 | | -0.3109 | 5.4131 | 0.3815 | 0.9996 | |
| AAEL013545 | | 0.5042 | 2.2142 | 0.3817 | 0.9996 | |
| AAEL001986 | | 0.3463 | 2.6795 | 0.3817 | 0.9996 | kinesin-like protein KIF1B [Source:VB Community Annotation] |
| AAEL022656 | NA | 0.5164 | 1.6186 | 0.3818 | 0.9996 | NA |
| AAEL003264 | | -0.2031 | 4.9535 | 0.3818 | 0.9996 | |
| AAEL021548 | NA | 0.3997 | 3.7420 | 0.3822 | 0.9996 | NA |
| AAEL001615 | mRpS18C | 0.2599 | 5.4690 | 0.3825 | 0.9996 | mitochondrial ribosomal protein, S18C, putative [Source:VB Community Annotation] |
| AAEL004592 | | 0.7746 | 2.7827 | 0.3825 | 0.9996 | tyrosine-protein kinase src64b [Source:VB Community Annotation] |
| AAEL023437 | NA | -0.6478 | 3.2759 | 0.3827 | 0.9996 | NA |
| AAEL006239 | | 0.3656 | 3.2971 | 0.3827 | 0.9996 | glycerol kinase [Source:VB Community Annotation] |
| AAEL013218 | | -1.2268 | -1.3639 | 0.3828 | 0.9996 | thiamine transporter [Source:VB Community Annotation] |
| AAEL007941 | | -1.6795 | 1.4078 | 0.3828 | 0.9996 | triacylglycerol lipase, putative [Source:VB Community Annotation] |
| AAEL012929 | retm | 0.4393 | 4.2324 | 0.3830 | 0.9996 | Protein real-time [Source:UniProtKB/Swiss-Prot;Acc:Q16KN5] |
| AAEL014583 | RpLP2 | -0.2503 | 12.0501 | 0.3832 | 0.9996 | 60S acidic ribosomal protein P2 [Source:UniProtKB/TrEMBL;Acc:Q1HRM9] |
| AAEL017329 | | -0.9386 | 6.3824 | 0.3833 | 0.9996 | |
| AAEL012942 | | -0.4485 | 3.4067 | 0.3837 | 0.9996 | |

| | | | | | | |
|---|---|---|---|---|---|---|
| AAEL023525 | NA | -1.8306 | -0.3460 | 0.3839 | 0.9996 | NA |
| AAEL025976 | NA | 0.4986 | 2.0929 | 0.3840 | 0.9996 | NA |
| AAEL001719 | | 0.5187 | 1.7395 | 0.3841 | 0.9996 | ATP-dependent RNA helicase [Source:VB Community Annotation] |
| AAEL005759 | | -0.7422 | -0.7286 | 0.3842 | 0.9996 | |
| AAEL001447 | | 0.2425 | 5.6812 | 0.3844 | 0.9996 | |
| AAEL023932 | NA | 1.0630 | -1.9613 | 0.3846 | 0.9996 | NA |
| AAEL008099 | | 0.2080 | 7.0047 | 0.3846 | 0.9996 | procollagen-lysine,2-oxoglutarate 5-dioxygenase [Source:VB Community Annotation] |
| AAEL024746 | NA | 0.3928 | 3.4788 | 0.3847 | 0.9996 | NA |
| AAEL003064 | | -0.1856 | 6.8256 | 0.3847 | 0.9996 | |
| AAEL006241 | | 0.5321 | 1.8006 | 0.3847 | 0.9996 | sugar transporter [Source:VB Community Annotation] |
| AAEL017664 | U2 | 0.5711 | -0.0074 | 0.3848 | 0.9996 | U2 spliceosomal RNA [Source:RFAM;Acc:RF00004] |
| AAEL020160 | NA | 0.6046 | 1.3750 | 0.3850 | 0.9996 | NA |
| AAEL018054 | | 0.2783 | 3.8564 | 0.3851 | 0.9996 | |
| AAEL003291 | | -0.3702 | 5.3873 | 0.3852 | 0.9996 | |
| AAEL003157 | | 0.3588 | 3.6775 | 0.3852 | 0.9996 | Protein SMG8 (Protein smg-8 homolog) [Source:VB Community Annotation] |
| AAEL014032 | | -1.1383 | -1.1954 | 0.3854 | 0.9996 | |
| AAEL007882 | | 0.3796 | 3.4545 | 0.3854 | 0.9996 | histone H1, putative [Source:VB Community Annotation] |
| AAEL019833 | NA | -0.7206 | 8.7985 | 0.3855 | 0.9996 | NA |
| AAEL024058 | NA | -1.0146 | -1.3507 | 0.3856 | 0.9996 | NA |
| AAEL013585 | | -0.9971 | -1.5161 | 0.3857 | 0.9996 | |
| AAEL010779 | | 0.7502 | 0.6882 | 0.3857 | 0.9996 | pickpocket [Source:VB Community Annotation] |
| AAEL021861 | NA | 0.4382 | 4.2937 | 0.3858 | 0.9996 | NA |
| AAEL011892 | | 0.5790 | 2.8318 | 0.3858 | 0.9996 | receptor for activated C kinase, putative [Source:VB Community Annotation] |
| AAEL012743 | | 0.3264 | 4.5185 | 0.3859 | 0.9996 | glycerol-3-phosphate acyltransferase [Source:VB Community Annotation] |
| AAEL017462 | | -0.2424 | 7.7507 | 0.3860 | 0.9996 | |
| AAEL017290 | | -0.2082 | 6.8293 | 0.3861 | 0.9996 | Coiled-coil domain-containing protein 72 homolog [Source:VB Community Annotation] |
| AAEL005453 | | -0.3163 | 4.0764 | 0.3861 | 0.9996 | |
| AAEL006354 | | 0.9989 | -0.7972 | 0.3862 | 0.9996 | epoxide hydrolase [Source:VB Community Annotation] |
| AAEL026924 | NA | 0.3330 | 5.0394 | 0.3867 | 0.9996 | NA |
| AAEL028100 | NA | 0.7395 | 1.9682 | 0.3869 | 0.9996 | NA |
| AAEL005031 | | -0.5726 | 0.8413 | 0.3869 | 0.9996 | |
| AAEL012944 | RpL11 | -0.2272 | 9.9701 | 0.3873 | 0.9996 | 60S ribosomal protein L11 [Source:VB Community Annotation] |
| AAEL009813 | | 0.4445 | 3.2804 | 0.3873 | 0.9996 | glutamate receptor 7 (ampa) [Source:VB Community Annotation] |
| AAEL025118 | NA | -0.9950 | -1.1605 | 0.3874 | 0.9996 | NA |
| AAEL014272 | | 0.2696 | 4.5023 | 0.3877 | 0.9996 | molybdopterin cofactor sulfurase (mosc) [Source:VB Community Annotation] |
| AAEL001267 | | -1.0357 | 0.4867 | 0.3879 | 0.9996 | |
| AAEL026302 | NA | -0.4108 | 5.5796 | 0.3880 | 0.9996 | NA |
| AAEL014366 | | -0.5648 | 2.9899 | 0.3881 | 0.9996 | |
| AAEL009784 | | -1.0487 | -0.3541 | 0.3881 | 0.9996 | cuticle protein, putative [Source:VB Community Annotation] |
| AAEL002401 | | -0.2322 | 6.3830 | 0.3882 | 0.9996 | Proteasome subunit beta type [Source:UniProtKB/TrEMBL;Acc:Q17IE3] |

| | | | | | | |
|---|---|---|---|---|---|---|
| AAEL012741 | | -1.2219 | 0.1894 | 0.3883 | 0.9996 | |
| AAEL012096 | | 0.7790 | 0.0609 | 0.3883 | 0.9996 | |
| AAEL001479 | | 0.2445 | 7.1959 | 0.3883 | 0.9996 | protoheme ix farnesyltransferase [Source:VB Community Annotation] |
| AAEL013897 | | 0.2169 | 4.4186 | 0.3884 | 0.9996 | n-acetylglucosaminyltransferase vi [Source:VB Community Annotation] |
| AAEL005618 | | -1.0507 | 0.9425 | 0.3884 | 0.9996 | chitin synthase [Source:VB Community Annotation] |
| AAEL010043 | GPRCAL1 | -0.3401 | 5.2495 | 0.3884 | 0.9996 | GPCR Calcitonin/Diuretic Hormone Family [Source:VB Community Annotation] |
| AAEL004243 | | 0.3401 | 3.3960 | 0.3886 | 0.9996 | S1 RNA binding domain protein, putative [Source:VB Community Annotation] |
| AAEL008658 | LRIM16 | -0.5398 | 6.6216 | 0.3887 | 0.9996 | leucine-rich immune protein (TM) [Source:VB Community Annotation] |
| AAEL009416 | | 0.2932 | 4.1864 | 0.3887 | 0.9996 | PHD finger domain [Source:VB Community Annotation] |
| AAEL025110 | NA | 0.3912 | 9.0748 | 0.3888 | 0.9996 | NA |
| AAEL005044 | | -1.4552 | 1.2551 | 0.3889 | 0.9996 | |
| AAEL014134 | | 0.3557 | 2.5051 | 0.3889 | 0.9996 | kinesin heavy chain [Source:VB Community Annotation] |
| AAEL008386 | | -0.8765 | -0.1866 | 0.3890 | 0.9996 | ATP-binding cassette sub-family A member 3, putative [Source:VB Community Annotation] |
| AAEL013006 | | -0.6795 | 0.7471 | 0.3891 | 0.9996 | |
| AAEL020307 | NA | 0.3415 | 5.0541 | 0.3891 | 0.9996 | NA |
| AAEL007603 | OBP10 | -0.6960 | 5.9620 | 0.3892 | 0.9996 | odorant binding protein (OBP10) [Source:VB Community Annotation] |
| AAEL023384 | NA | 0.4482 | 3.2559 | 0.3893 | 0.9996 | NA |
| AAEL025098 | NA | -0.7846 | 0.3906 | 0.3894 | 0.9996 | NA |
| AAEL008025 | | 0.2644 | 6.7420 | 0.3894 | 0.9996 | |
| AAEL028771 | NA | -1.0651 | -2.2143 | 0.3894 | 0.9996 | NA |
| AAEL015238 | | -0.2854 | 8.4860 | 0.3897 | 0.9996 | UPF0729 protein AAEL015238 [Source:UniProtKB/Swiss-Prot;Acc:Q16EE5] |
| AAEL021017 | NA | 0.7907 | -0.7010 | 0.3899 | 0.9996 | NA |
| AAEL026895 | NA | -0.2600 | 6.8256 | 0.3899 | 0.9996 | NA |
| AAEL019670 | NA | 0.7240 | 1.8927 | 0.3899 | 0.9996 | NA |
| AAEL012472 | | -0.9854 | 2.3735 | 0.3900 | 0.9996 | kinesin light chain 1 and [Source:VB Community Annotation] |
| AAEL017685 | RNaseP_nu | -1.2173 | 1.3510 | 0.3901 | 0.9996 | Nuclear RNase P [Source:RFAM;Acc:RF00009 |
| AAEL003133 | | -0.2475 | 5.9155 | 0.3901 | 0.9996 | |
| AAEL007981 | | 0.6255 | 0.6399 | 0.3902 | 0.9996 | |
| AAEL007703 | | 1.0493 | 3.1360 | 0.3902 | 0.9996 | |
| AAEL019812 | NA | -0.4328 | 4.1389 | 0.3903 | 0.9996 | NA |
| AAEL004551 | | 0.6937 | 2.0618 | 0.3903 | 0.9996 | |
| AAEL008786 | | -0.2179 | 6.9690 | 0.3903 | 0.9996 | caax prenyl protease ste24 [Source:VB Community Annotation] |
| AAEL001935 | | 0.2852 | 4.6099 | 0.3903 | 0.9996 | CTL-like protein 1 [Source:UniProtKB/Swiss-Prot;Acc:Q17JQ7] |
| AAEL024750 | NA | 1.2747 | 0.8395 | 0.3905 | 0.9996 | NA |
| AAEL020403 | NA | 0.3725 | 4.8402 | 0.3905 | 0.9996 | NA |
| AAEL013384 | | -0.8919 | 3.4740 | 0.3908 | 0.9996 | |
| AAEL002759 | | 0.7222 | 7.7416 | 0.3909 | 0.9996 | tropomyosin invertebrate [Source:VB Community Annotation] |
| AAEL010582 | GSTD11 | 0.8206 | 2.2199 | 0.3909 | 0.9996 | glutathione transferase [Source:VB Community Annotation] |
| AAEL009981 | | -0.2454 | 5.3526 | 0.3910 | 0.9996 | chaperone atp11p [Source:VB Community Annotation] |
| AAEL021090 | NA | 0.3087 | 2.4129 | 0.3911 | 0.9996 | NA |

| | | | | | | |
|---|---|---|---|---|---|---|
| AAEL008936 | | -0.8638 | 6.4750 | 0.3911 | 0.9996 | |
| AAEL014264 | | -0.2376 | 3.2677 | 0.3912 | 0.9996 | |
| AAEL012220 | | -0.3432 | 2.9407 | 0.3912 | 0.9996 | |
| AAEL010048 | | 1.2354 | 0.8903 | 0.3914 | 0.9996 | 26S proteasome non-ATPase regulatory subunit [Source:VB Community Annotation] |
| AAEL002249 | | -0.3345 | 2.9273 | 0.3915 | 0.9996 | |
| AAEL005202 | | 0.3437 | 2.9626 | 0.3915 | 0.9996 | |
| AAEL002386 | | -0.2543 | 5.7844 | 0.3916 | 0.9996 | |
| AAEL015147 | Or4 | -0.8421 | -0.4358 | 0.3917 | 0.9996 | odorant receptor (Or4) [Source:VB Community Annotation] |
| AAEL007654 | | 0.4418 | 1.9001 | 0.3919 | 0.9996 | |
| AAEL001497 | | 0.4617 | 2.6171 | 0.3919 | 0.9996 | vacuolar protein sorting [Source:VB Community Annotation] |
| AAEL013422 | | -0.7904 | -0.5813 | 0.3920 | 0.9996 | odorant receptor 42a, putative [Source:VB Community Annotation] |
| AAEL013132 | | 0.2902 | 6.2889 | 0.3922 | 0.9996 | 6-phosphofructo-2-kinase/fructose-2,6-bisphosphatase short form [Source:VB Community Annotation] |
| AAEL005539 | | 0.4976 | 2.4503 | 0.3922 | 0.9996 | |
| AAEL009252 | | 0.4045 | 1.9203 | 0.3922 | 0.9996 | |
| AAEL022924 | NA | -0.8862 | -0.4234 | 0.3925 | 0.9996 | NA |
| AAEL017418 | | 0.3968 | 3.9916 | 0.3926 | 0.9996 | |
| AAEL006909 | | 0.5773 | 5.9910 | 0.3926 | 0.9996 | |
| AAEL022505 | NA | 0.1941 | 4.8391 | 0.3928 | 0.9996 | NA |
| AAEL006139 | | 0.1834 | 6.7405 | 0.3928 | 0.9996 | sodium/solute symporter [Source:VB Community Annotation] |
| AAEL006744 | | -0.2416 | 5.2018 | 0.3930 | 0.9996 | exonuclease nef-sp [Source:VB Community Annotation] |
| AAEL004567 | | -0.5787 | 5.2131 | 0.3930 | 0.9996 | fermitin, putative [Source:VB Community Annotation] |
| AAEL003844 | GALE5 | 0.3632 | 6.4267 | 0.3931 | 0.9996 | Galectin [Source:UniProtKB/TrEMBL;Acc:Q17EC8] |
| AAEL012211 | | 0.2521 | 4.5076 | 0.3932 | 0.9996 | microfibrillar-associated protein, putative [Source:VB Community Annotation] |
| AAEL025611 | NA | -0.7328 | 5.7053 | 0.3933 | 0.9996 | NA |
| AAEL010971 | | -0.6814 | 4.5878 | 0.3935 | 0.9996 | |
| AAEL010975 | | 0.7193 | 6.1248 | 0.3935 | 0.9996 | paramyosin, long form [Source:VB Community Annotation] |
| AAEL012691 | | -0.3704 | 6.1710 | 0.3937 | 0.9996 | sodium/solute symporter [Source:VB Community Annotation] |
| AAEL014187 | | -0.2166 | 5.6792 | 0.3937 | 0.9996 | chilling-inducible protein, putative [Source:VB Community Annotation] |
| AAEL014280 | | 0.2786 | 4.3367 | 0.3937 | 0.9996 | SNAPIN protein homolog [Source:UniProtKB/TrEMBL;Acc:Q16GS5] |
| AAEL025423 | NA | -0.3386 | 2.2956 | 0.3938 | 0.9996 | NA |
| AAEL006010 | | 0.3319 | 4.0983 | 0.3939 | 0.9996 | Vesicle transport protein [Source:UniProtKB/TrEMBL;Acc:Q177W9] |
| AAEL012503 | Pym | 0.2459 | 5.3948 | 0.3941 | 0.9996 | Partner of Y14 and mago (Protein wibg homolog) [Source:VB Community Annotation] |
| AAEL007390 | | -0.3848 | 5.5698 | 0.3941 | 0.9996 | UDP-glucose 4-epimerase [Source:VB Community Annotation] |
| AAEL020452 | NA | -0.5434 | 8.7753 | 0.3941 | 0.9996 | NA |
| AAEL015566 | OBP62 | 0.4757 | 6.4852 | 0.3944 | 0.9996 | odorant binding protein OBP62 [Source:VB Community Annotation] |
| AAEL010382 | | 0.8724 | 3.0905 | 0.3944 | 0.9996 | aldehyde oxidase [Source:VB Community Annotation] |
| AAEL022296 | NA | -0.8901 | -0.1682 | 0.3944 | 0.9996 | NA |
| AAEL025527 | NA | -0.7463 | -0.4961 | 0.3944 | 0.9996 | NA |
| AAEL004456 | | 0.5726 | 2.8367 | 0.3944 | 0.9996 | DEAD box ATP-dependent RNA helicase [Source:VB Community Annotation] |
| AAEL004390 | HPX8B | -1.3665 | -0.7320 | 0.3945 | 0.9996 | heme peroxidase [Source:VB Community Annotation] |

| | | | | | | |
|---|---|---|---|---|---|---|
| AAEL012632 | | -0.2943 | 5.8121 | 0.3946 | 0.9996 | |
| AAEL027512 | NA | -0.4543 | 2.5491 | 0.3947 | 0.9996 | NA |
| AAEL025725 | NA | -0.5039 | 1.1310 | 0.3948 | 0.9996 | NA |
| AAEL013188 | | 0.2618 | 4.8896 | 0.3948 | 0.9996 | cle7 [Source:VB Community Annotation] |
| AAEL010656 | LRIM12 | 0.6979 | 4.9282 | 0.3951 | 0.9996 | leucine-rich immune protein (Short) [Source:VB Community Annotation] |
| AAEL006899 | | 0.2725 | 4.7292 | 0.3951 | 0.9996 | DNA-J/hsp40 [Source:VB Community Annotation] |
| AAEL002374 | | 0.3142 | 3.0179 | 0.3951 | 0.9996 | taxilin [Source:VB Community Annotation] |
| AAEL020950 | NA | -1.6486 | 2.8583 | 0.3954 | 0.9996 | NA |
| AAEL008289 | | 0.3834 | 10.4425 | 0.3954 | 0.9996 | pupal cuticle protein 78E, putative [Source:VB Community Annotation] |
| AAEL019689 | NA | 0.5516 | 1.0578 | 0.3955 | 0.9996 | NA |
| AAEL004676 | | -0.3825 | 4.8634 | 0.3958 | 0.9996 | (s)-2-hydroxy-acid oxidase [Source:VB Community Annotation] |
| AAEL011023 | | 0.2721 | 4.4577 | 0.3958 | 0.9996 | |
| AAEL000495 | GPXH3 | 0.9590 | -2.0852 | 0.3962 | 0.9996 | glutathione peroxidase [Source:VB Community Annotation] |
| AAEL019486 | NA | 0.3197 | 5.3697 | 0.3964 | 0.9996 | NA |
| AAEL016969 | | -0.3623 | 2.2305 | 0.3965 | 0.9996 | |
| AAEL009412 | | 0.2741 | 4.5269 | 0.3966 | 0.9996 | ligatin [Source:VB Community Annotation] |
| AAEL007697 | | -0.3550 | 3.4954 | 0.3967 | 0.9996 | |
| AAEL007519 | | -0.3707 | 1.7042 | 0.3968 | 0.9996 | oviductin [Source:VB Community Annotation] |
| AAEL023462 | NA | -0.8573 | 0.0923 | 0.3968 | 0.9996 | NA |
| AAEL009262 | | -0.2591 | 4.0237 | 0.3969 | 0.9996 | |
| AAEL025459 | NA | 0.4007 | 1.9160 | 0.3969 | 0.9996 | NA |
| AAEL011130 | | 0.6121 | 3.0515 | 0.3969 | 0.9996 | alcohol dehydrogenase [Source:VB Community Annotation] |
| AAEL019404 | NA | 0.3115 | 3.8034 | 0.3970 | 0.9996 | NA |
| AAEL009950 | | 0.6573 | 0.6319 | 0.3973 | 0.9996 | homeotic deformed protein, putative [Source:VB Community Annotation] |
| AAEL002626 | | 0.8603 | 0.7542 | 0.3973 | 0.9996 | odorant-binding protein 56e, putative [Source:VB Community Annotation] |
| AAEL022605 | NA | 0.5682 | 3.3492 | 0.3975 | 0.9996 | NA |
| AAEL009880 | | 0.7449 | -0.4521 | 0.3975 | 0.9996 | serine/threonine protein kinase [Source:VB Community Annotation] |
| AAEL004048 | | 0.5152 | 4.5283 | 0.3975 | 0.9996 | UNC93A protein, putative [Source:VB Community Annotation] |
| AAEL006550 | | 0.2820 | 5.8782 | 0.3979 | 0.9996 | rnf5 [Source:VB Community Annotation] |
| AAEL010302 | | 0.2491 | 3.6628 | 0.3979 | 0.9996 | |
| AAEL008676 | | 0.2953 | 4.1041 | 0.3980 | 0.9996 | |
| AAEL000983 | | 0.1980 | 6.1675 | 0.3980 | 0.9996 | clathrin coat assembly protein ap19 [Source:VB Community Annotation] |
| AAEL005770 | OPB21 | 0.4052 | 4.4423 | 0.3981 | 0.9996 | odorant binding protein OBP21 [Source:VB Community Annotation] |
| AAEL011979 | | -0.2756 | 4.2655 | 0.3983 | 0.9996 | calmodulin [Source:VB Community Annotation] |
| AAEL027798 | NA | -1.1101 | -1.2237 | 0.3985 | 0.9996 | NA |
| AAEL003938 | | 0.2853 | 5.0780 | 0.3991 | 0.9996 | mRNA cleavage stimulating factor, 50kD-subunit, putative [Source:VB Community Annotation] |
| AAEL005902 | | -0.2385 | 6.2050 | 0.3992 | 0.9996 | |
| AAEL014038 | | 0.5643 | 0.5890 | 0.3993 | 0.9996 | NIF3-like protein 1, putative [Source:VB Community Annotation] |
| AAEL012897 | | 0.2978 | 8.4886 | 0.3996 | 0.9996 | aconitase, mitochondrial [Source:VB Community Annotation] |
| AAEL026731 | NA | 0.4566 | 3.8801 | 0.3998 | 0.9996 | NA |

| | | | | | | |
|---|---|---|---|---|---|---|
| AAEL000566 | | 1.2661 | -0.4043 | 0.3999 | 0.9996 | |
| AAEL002893 | | -0.4026 | 4.4482 | 0.3999 | 0.9996 | short-chain dehydrogenase [Source:VB Community Annotation] |
| AAEL024687 | NA | -0.9267 | -2.7706 | 0.4001 | 0.9996 | NA |
| AAEL001325 | | -0.4074 | 6.6393 | 0.4004 | 0.9996 | |
| AAEL009734 | | -0.3951 | 7.0025 | 0.4006 | 0.9996 | |
| AAEL019569 | NA | 0.5548 | 0.1317 | 0.4007 | 0.9996 | NA |
| AAEL019747 | NA | 0.2696 | 3.7007 | 0.4007 | 0.9996 | NA |
| AAEL019727 | NA | -0.3588 | 7.6118 | 0.4009 | 0.9996 | NA |
| AAEL019912 | NA | -1.0888 | -2.1032 | 0.4010 | 0.9996 | NA |
| AAEL010477 | | 0.5485 | 1.2143 | 0.4010 | 0.9996 | |
| AAEL012904 | | 0.3156 | 6.6634 | 0.4011 | 0.9996 | rab gdp-dissociation inhibitor [Source:VB Community Annotation] |
| AAEL000248 | | 1.0453 | 3.5133 | 0.4012 | 0.9996 | GTP binding protein [Source:VB Community Annotation] |
| AAEL004406 | | -0.2200 | 4.7503 | 0.4014 | 0.9996 | |
| AAEL004209 | | -0.2861 | 6.4536 | 0.4014 | 0.9996 | opioid-binding protein/cell adhesion molecule, putative [Source:VB Community Annotation] |
| AAEL022165 | NA | -1.5173 | 0.1427 | 0.4015 | 0.9996 | NA |
| AAEL019552 | NA | 0.2033 | 4.8619 | 0.4017 | 0.9996 | NA |
| AAEL010607 | | 0.2677 | 3.9032 | 0.4019 | 0.9996 | |
| AAEL005290 | | 0.2324 | 4.5052 | 0.4019 | 0.9996 | |
| AAEL021887 | NA | -1.4447 | -0.4751 | 0.4020 | 0.9996 | NA |
| AAEL024602 | NA | -0.5550 | 3.9005 | 0.4020 | 0.9996 | NA |
| AAEL012015 | | -0.9807 | -1.4113 | 0.4021 | 0.9996 | DEAD box ATP-dependent RNA helicase [Source:VB Community Annotation] |
| AAEL019446 | NA | -0.4722 | 2.4030 | 0.4021 | 0.9996 | NA |
| AAEL006102 | | 0.4482 | 9.1575 | 0.4022 | 0.9996 | gelsolin precursor [Source:VB Community Annotation] |
| AAEL008276 | | 0.2781 | 3.5685 | 0.4022 | 0.9996 | |
| AAEL000623 | | -0.9761 | -1.1770 | 0.4025 | 0.9996 | |
| AAEL014915 | | 0.2913 | 5.6239 | 0.4025 | 0.9996 | 26S proteasome subunit [Source:VB Community Annotation] |
| AAEL012368 | | -0.3660 | 3.2639 | 0.4026 | 0.9996 | |
| AAEL025218 | NA | -0.8604 | -0.6335 | 0.4027 | 0.9996 | NA |
| AAEL004579 | | -0.2825 | 3.4838 | 0.4028 | 0.9996 | short-chain dehydrogenase [Source:VB Community Annotation] |
| AAEL012918 | | 0.2520 | 5.0662 | 0.4029 | 0.9996 | puromycin-sensitive aminopeptidase [Source:VB Community Annotation] |
| AAEL024776 | NA | -1.1102 | 2.4879 | 0.4030 | 0.9996 | NA |
| AAEL003505 | | 0.5320 | 4.9288 | 0.4032 | 0.9996 | jun [Source:VB Community Annotation] |
| AAEL024260 | NA | 0.4511 | 2.5668 | 0.4034 | 0.9996 | NA |
| AAEL014843 | | 0.4270 | 9.0552 | 0.4035 | 0.9996 | heat shock protein [Source:VB Community Annotation] |
| AAEL002481 | | -0.4995 | 3.6159 | 0.4040 | 0.9996 | |
| AAEL019886 | NA | 0.3018 | 3.4643 | 0.4044 | 0.9996 | NA |
| AAEL019783 | NA | 0.3393 | 4.5218 | 0.4046 | 0.9996 | NA |
| AAEL011193 | | -0.3053 | 5.6018 | 0.4046 | 0.9996 | steroid dehydrogenase [Source:VB Community Annotation] |
| AAEL000583 | | -0.4189 | 3.4920 | 0.4047 | 0.9996 | peroxisome assembly factor-2 (peroxisomal-type ATPase 1) [Source:VB Community Annotation] |
| AAEL002833 | | -0.2819 | 9.9580 | 0.4047 | 0.9996 | cathepsin l [Source:VB Community Annotation] |

| | | | | | | |
|---|---|---|---|---|---|---|
| AAEL013741 | | 0.3954 | 1.5695 | 0.4048 | 0.9996 | |
| AAEL004117 | | 0.3570 | 4.4677 | 0.4049 | 0.9996 | ATP-dependent RNA helicase [Source:VB Community Annotation] |
| AAEL004734 | | 0.3049 | 3.6929 | 0.4049 | 0.9996 | UBX domain-containing protein 7 [Source:UniProtKB/TrEMBL;Acc:A0A1S4F8G8] |
| AAEL014135 | | 0.3445 | 3.9765 | 0.4049 | 0.9996 | |
| AAEL005027 | RpLP1 | -0.3019 | 5.4304 | 0.4050 | 0.9996 | acidic ribosomal protein P1, putative [Source:VB Community Annotation] |
| AAEL012229 | | -0.3389 | 3.4861 | 0.4050 | 0.9996 | |
| AAEL008601 | mRpL28 | -0.3002 | 6.8863 | 0.4051 | 0.9996 | 39S mitochondrial ribosomal protein L28 [Source:VB Community Annotation] |
| AAEL000308 | | 0.4286 | 3.0613 | 0.4052 | 0.9996 | |
| AAEL006666 | | 0.2794 | 3.2437 | 0.4052 | 0.9996 | cmp-sialic acid transporter [Source:VB Community Annotation] |
| AAEL008902 | | -0.1893 | 5.7885 | 0.4054 | 0.9996 | cation-transporting ATPase fly [Source:VB Community Annotation] |
| AAEL000457 | | -0.2591 | 10.3020 | 0.4058 | 0.9996 | |
| AAEL022278 | NA | 0.9481 | -2.6651 | 0.4059 | 0.9996 | NA |
| AAEL011268 | | 0.4835 | 2.7144 | 0.4061 | 0.9996 | phosphatidylethanolamine-binding protein [Source:VB Community Annotation] |
| AAEL008959 | | -1.5327 | 1.7404 | 0.4061 | 0.9996 | ras-related protein Rab-39B, putative [Source:VB Community Annotation] |
| AAEL003601 | | -1.5221 | 7.2538 | 0.4062 | 0.9996 | |
| AAEL008683 | | 0.3315 | 2.7725 | 0.4063 | 0.9996 | |
| AAEL006749 | | -0.7510 | 5.4927 | 0.4063 | 0.9996 | |
| AAEL008867 | | 0.3138 | 2.1605 | 0.4064 | 0.9996 | |
| AAEL001244 | | 0.4550 | 3.7437 | 0.4066 | 0.9996 | |
| AAEL007824 | RpS29 | -0.2178 | 12.6785 | 0.4067 | 0.9996 | 40S ribosomal protein S29 [Source:UniProtKB/TrEMBL;Acc:Q1HRA1] |
| AAEL003875 | | -0.2271 | 4.3542 | 0.4067 | 0.9996 | transcription factor SP4, putative [Source:VB Community Annotation] |
| AAEL025224 | NA | 0.9239 | 0.7105 | 0.4067 | 0.9996 | NA |
| AAEL010978 | | 0.2571 | 4.0422 | 0.4068 | 0.9996 | |
| AAEL004923 | | 0.8064 | 1.9590 | 0.4069 | 0.9996 | nicotinic acetylcholine receptor, putative [Source:VB Community Annotation] |
| AAEL018237 | | -0.4114 | 4.4473 | 0.4069 | 0.9996 | |
| AAEL000126 | | 0.7836 | 2.9108 | 0.4070 | 0.9996 | molybdopterin cofactor synthesis protein a [Source:VB Community Annotation] |
| AAEL001597 | | -0.4023 | 1.4485 | 0.4070 | 0.9996 | |
| AAEL010041 | | 0.2068 | 4.2693 | 0.4071 | 0.9996 | |
| AAEL014486 | | 0.8541 | 2.3708 | 0.4072 | 0.9996 | |
| AAEL010170 | | 0.4819 | 4.3424 | 0.4073 | 0.9996 | ras-related protein Rab-8A, putative [Source:VB Community Annotation] |
| AAEL006614 | | 0.2182 | 3.5316 | 0.4073 | 0.9996 | zinc finger protein [Source:VB Community Annotation] |
| AAEL001694 | | 0.9746 | -0.8575 | 0.4073 | 0.9996 | |
| AAEL001166 | | -0.2940 | 4.6206 | 0.4074 | 0.9996 | protein phosphatases pp1 regulatory subunit [Source:VB Community Annotation] |
| AAEL011690 | | -1.0486 | -0.9391 | 0.4074 | 0.9996 | |
| AAEL018148 | | 0.1924 | 3.9642 | 0.4075 | 0.9996 | Nicotinamide-nucleotide adenylyltransferase [Source:UniProtKB/TrEMBL;Acc:A0A1S4G764] |
| AAEL026231 | NA | 0.3908 | 2.6736 | 0.4075 | 0.9996 | NA |
| AAEL022123 | NA | -0.3208 | 2.3540 | 0.4076 | 0.9996 | NA |
| AAEL001991 | | 0.7276 | 1.3303 | 0.4076 | 0.9996 | protein serine/threonine kinase, putative [Source:VB Community Annotation] |
| AAEL001557 | | -0.2395 | 4.5100 | 0.4078 | 0.9996 | mucolipin [Source:VB Community Annotation] |
| AAEL026946 | NA | -0.2469 | 6.4346 | 0.4078 | 0.9996 | NA |

| | | | | | | |
|---|---|---|---|---|---|---|
| AAEL003275 | | -0.4866 | 7.1486 | 0.4078 | 0.9996 | |
| AAEL011429 | | 0.3104 | 6.2401 | 0.4079 | 0.9996 | clathrin coat adaptor ap3 medium chain [Source:VB Community Annotation] |
| AAEL024820 | NA | 0.5590 | 1.6568 | 0.4080 | 0.9996 | NA |
| AAEL005964 | | -0.9856 | 0.6204 | 0.4081 | 0.9996 | actin [Source:VB Community Annotation] |
| AAEL005444 | | 0.3313 | 4.1075 | 0.4083 | 0.9996 | pyrokinin, putative [Source:VB Community Annotation] |
| AAEL021929 | NA | -0.7624 | 6.2351 | 0.4083 | 0.9996 | NA |
| AAEL018298 | | -1.1166 | 3.8260 | 0.4084 | 0.9996 | |
| AAEL009874 | | -0.3182 | 6.3085 | 0.4084 | 0.9996 | |
| AAEL004571 | | 0.4729 | 1.2600 | 0.4085 | 0.9996 | o-sialoglycoprotein endopeptidase [Source:VB Community Annotation] |
| AAEL008111 | | -0.2733 | 8.5594 | 0.4087 | 0.9996 | |
| AAEL020073 | NA | -0.2049 | 5.9457 | 0.4088 | 0.9996 | NA |
| AAEL002346 | plexA | -0.5950 | 4.8465 | 0.4088 | 0.9996 | plexin a [Source:VB Community Annotation] |
| AAEL017263 | | 0.2260 | 5.6137 | 0.4089 | 0.9996 | |
| AAEL001508 | | -0.5077 | 3.8861 | 0.4090 | 0.9996 | |
| AAEL002388 | | 0.3184 | 2.6242 | 0.4090 | 0.9996 | zinc finger protein [Source:VB Community Annotation] |
| AAEL012912 | | 0.2869 | 3.7795 | 0.4091 | 0.9996 | |
| AAEL027735 | NA | 0.6297 | 1.4754 | 0.4092 | 0.9996 | NA |
| AAEL006635 | | -0.4730 | 1.9299 | 0.4094 | 0.9996 | Fatty acyl-CoA reductase [Source:UniProtKB/TrEMBL;Acc:A0A1S4FE71] |
| AAEL014058 | | 0.2748 | 3.5440 | 0.4094 | 0.9996 | copper homeostasis protein [Source:VB Community Annotation] |
| AAEL009533 | | 1.1130 | -0.8760 | 0.4095 | 0.9996 | |
| AAEL027332 | NA | -0.5038 | 1.0818 | 0.4096 | 0.9996 | NA |
| AAEL019674 | NA | 0.3042 | 7.9406 | 0.4097 | 0.9996 | NA |
| AAEL002451 | | -0.8812 | 2.7234 | 0.4097 | 0.9996 | zinc finger protein [Source:VB Community Annotation] |
| AAEL017249 | SRPN24 | -0.8130 | 2.5230 | 0.4097 | 0.9996 | Serine Protease Inhibitor (serpin) homologue - unlikely to be inhibitory. [Source:VB Community Annotation] |
| AAEL009668 | | -0.2094 | 3.4284 | 0.4098 | 0.9996 | |
| AAEL014151 | | -0.3929 | 2.9648 | 0.4098 | 0.9996 | six/sine homebox transcription factors [Source:VB Community Annotation] |
| AAEL019500 | NA | -0.7783 | 5.0111 | 0.4099 | 0.9996 | NA |
| AAEL006414 | | -1.2805 | -2.4969 | 0.4100 | 0.9996 | trypsin [Source:VB Community Annotation] |
| AAEL028052 | NA | -0.2579 | 4.3910 | 0.4101 | 0.9996 | NA |
| AAEL023677 | NA | -0.9498 | 0.1290 | 0.4101 | 0.9996 | NA |
| AAEL024288 | NA | -0.6590 | 1.0556 | 0.4101 | 0.9996 | NA |
| AAEL026037 | NA | -0.5739 | 1.2248 | 0.4102 | 0.9996 | NA |
| AAEL013621 | | 0.6567 | 0.6150 | 0.4102 | 0.9996 | |
| AAEL025803 | NA | -0.7992 | 0.9037 | 0.4102 | 0.9996 | NA |
| AAEL008754 | | 0.8728 | 0.7578 | 0.4102 | 0.9996 | |
| AAEL012884 | | 0.2918 | 3.6869 | 0.4103 | 0.9996 | |
| AAEL008132 | | -0.8237 | -0.4004 | 0.4106 | 0.9996 | CRAL/TRIO domain-containing protein [Source:VB Community Annotation] |
| AAEL005374 | SCRB1 | 0.4968 | 6.3735 | 0.4108 | 0.9996 | Class B Scavenger Receptor (CD36 domain) [Source:VB Community Annotation] |
| AAEL000690 | | 0.6532 | 2.1313 | 0.4111 | 0.9996 | steroid dehydrogenase [Source:VB Community Annotation] |
| AAEL027277 | NA | 0.4806 | 1.0010 | 0.4111 | 0.9996 | NA |

| | | | | | | |
|---|---|---|---|---|---|---|
| AAEL003253 | CLIPB13B | -0.6867 | 3.1428 | 0.4111 | 0.9996 | Clip-Domain Serine Protease  family B. [Source:VB Community Annotation] |
| AAEL027115 | NA | -2.7735 | 2.8960 | 0.4111 | 0.9996 | NA |
| AAEL001154 | | -0.2742 | 6.1692 | 0.4111 | 0.9996 | |
| AAEL002983 | | 0.2688 | 5.1764 | 0.4113 | 0.9996 | |
| AAEL023888 | NA | 1.0779 | -1.8826 | 0.4114 | 0.9996 | NA |
| AAEL021231 | NA | 0.2631 | 3.2646 | 0.4116 | 0.9996 | NA |
| AAEL001808 | | -0.3437 | 4.5655 | 0.4117 | 0.9996 | organic anion transporter [Source:VB Community Annotation] |
| AAEL006973 | | -0.3427 | 5.4240 | 0.4117 | 0.9996 | ubiquitin conjugating enzyme 7 interacting protein [Source:VB Community Annotation] |
| AAEL011989 | | -0.2466 | 5.8114 | 0.4117 | 0.9996 | signal peptide peptidase [Source:VB Community Annotation] |
| AAEL027238 | NA | -0.5890 | 0.6660 | 0.4118 | 0.9996 | NA |
| AAEL006571 | PELLE | 0.3340 | 2.3955 | 0.4120 | 0.9996 | TOLL pathway signalling  Ser/Thr Kinase. [Source:VB Community Annotation] |
| AAEL004964 | | -0.2842 | 3.6983 | 0.4120 | 0.9996 | |
| AAEL022189 | NA | 0.5618 | 1.8047 | 0.4121 | 0.9996 | NA |
| AAEL005106 | | 0.4400 | 5.5239 | 0.4123 | 0.9996 | |
| AAEL014900 | | 0.3206 | 3.0835 | 0.4124 | 0.9996 | ataxia telangiectasia mutated (atm) [Source:VB Community Annotation] |
| AAEL021313 | NA | -0.5415 | 0.0462 | 0.4126 | 0.9996 | NA |
| AAEL000937 | | -0.3456 | 7.1107 | 0.4126 | 0.9996 | |
| AAEL026175 | NA | -1.4736 | -1.4005 | 0.4127 | 0.9996 | NA |
| AAEL027175 | NA | -0.5213 | 6.2117 | 0.4128 | 0.9996 | NA |
| AAEL002587 | OBP11 | -0.3660 | 8.4352 | 0.4128 | 0.9996 | odorant binding protein OBP11 [Source:VB Community Annotation] |
| AAEL001562 | | -1.0117 | 1.8227 | 0.4129 | 0.9996 | |
| AAEL002686 | | -0.8442 | 5.2268 | 0.4129 | 0.9996 | testisin precursor, putative [Source:VB Community Annotation] |
| AAEL010777 | | 0.1971 | 9.2191 | 0.4130 | 0.9996 | thioredoxin (TRX), putative [Source:VB Community Annotation] |
| AAEL005008 | | 0.6314 | 6.3486 | 0.4130 | 0.9996 | aquaporin [Source:VB Community Annotation] |
| AAEL009888 | CCC2 | -0.6916 | 2.9867 | 0.4131 | 0.9996 | sodium-coupled cation-chloride cotransporter [Source:VB Community Annotation] |
| AAEL000242 | | -0.9597 | 3.4556 | 0.4131 | 0.9996 | voltage-gated potassium channel [Source:VB Community Annotation] |
| AAEL008660 | | -0.2635 | 3.5105 | 0.4131 | 0.9996 | fig-alpha [Source:VB Community Annotation] |
| AAEL007101 | | -0.7518 | -0.7366 | 0.4132 | 0.9996 | adult cuticle protein, putative [Source:VB Community Annotation] |
| AAEL010396 | | -0.8986 | -0.2958 | 0.4132 | 0.9996 | secreted ferritin G subunit precursor, putative [Source:VB Community Annotation] |
| AAEL015060 | | 0.4951 | 0.9838 | 0.4133 | 0.9996 | Rad51A protein, putative [Source:VB Community Annotation] |
| AAEL008737 | | 0.3375 | 2.6791 | 0.4133 | 0.9996 | |
| AAEL013530 | | -0.2684 | 5.1097 | 0.4134 | 0.9996 | cullin [Source:VB Community Annotation] |
| AAEL003347 | | -0.7320 | 5.4790 | 0.4135 | 0.9996 | CRAL/TRIO domain-containing protein [Source:VB Community Annotation] |
| AAEL005575 | | -0.2733 | 12.1172 | 0.4136 | 0.9996 | transient receptor potential channel 4, [Source:VB Community Annotation] |
| AAEL000328 | | -0.2001 | 7.5978 | 0.4139 | 0.9996 | |
| AAEL008924 | | 0.2452 | 5.7160 | 0.4140 | 0.9996 | cysteine-rich hydrophobic domain 2 (chic1) [Source:VB Community Annotation] |
| AAEL001735 | | 1.1362 | -2.2931 | 0.4140 | 0.9996 | pupal cuticle protein 78E, putative [Source:VB Community Annotation] |
| AAEL004238 | | 0.4417 | 3.0307 | 0.4142 | 0.9996 | |
| AAEL000304 | | -0.4693 | 7.1447 | 0.4142 | 0.9996 | |
| AAEL004979 | CLIPD2 | -0.6149 | 2.7881 | 0.4143 | 0.9996 | Clip-Domain Serine Protease  family D. [Source:VB Community Annotation] |

| | | | | | | |
|---|---|---|---|---|---|---|
| AAEL015236 | | 0.2909 | 5.4762 | 0.4144 | 0.9996 | signal recognition particle, 9kD-subunit, putative [Source:VB Community Annotation] |
| AAEL002756 | | -1.1517 | 4.5451 | 0.4144 | 0.9996 | synaptotagmin-4, [Source:VB Community Annotation] |
| AAEL027533 | NA | -0.3903 | 1.4189 | 0.4145 | 0.9996 | NA |
| AAEL017079 | Or47 | 0.5332 | 2.0090 | 0.4146 | 0.9996 | Odorant receptor [Source:UniProtKB/TrEMBL;Acc:J9HTA3] |
| AAEL003160 | | -0.2562 | 9.2904 | 0.4146 | 0.9996 | |
| AAEL002719 | | 0.4534 | 3.2693 | 0.4147 | 0.9996 | |
| AAEL018982 | tRNA-Lys | -0.5107 | 1.5760 | 0.4147 | 0.9996 | |
| AAEL022944 | NA | -1.0934 | -1.4611 | 0.4147 | 0.9996 | NA |
| AAEL005033 | | 0.3209 | 2.1016 | 0.4148 | 0.9996 | |
| AAEL011624 | | -0.5976 | 5.9363 | 0.4150 | 0.9996 | granzyme A precursor, putative [Source:VB Community Annotation] |
| AAEL004724 | | 0.3530 | 4.6660 | 0.4150 | 0.9996 | Carboxylic ester hydrolase (Fragment) [Source:UniProtKB/TrEMBL;Acc:Q17C44] |
| AAEL020163 | NA | -1.1664 | -1.7998 | 0.4151 | 0.9996 | NA |
| AAEL005270 | | -0.9955 | 5.9595 | 0.4152 | 0.9996 | |
| AAEL026575 | NA | 0.7021 | 0.1115 | 0.4152 | 0.9996 | NA |
| AAEL014484 | | 0.3642 | 7.1656 | 0.4152 | 0.9996 | |
| AAEL028143 | NA | -0.8341 | -0.1752 | 0.4153 | 0.9996 | NA |
| AAEL015606 | | 0.7802 | 1.1426 | 0.4154 | 0.9996 | |
| AAEL023845 | NA | -1.1423 | 3.2206 | 0.4155 | 0.9996 | NA |
| AAEL006995 | | -1.0039 | 1.7502 | 0.4156 | 0.9996 | sodium/solute symporter [Source:VB Community Annotation] |
| AAEL022680 | NA | 0.8435 | 2.0654 | 0.4156 | 0.9996 | NA |
| AAEL011666 | mRpL41 | -0.2843 | 5.2171 | 0.4157 | 0.9996 | mitochondrial ribosomal protein, L41, putative [Source:VB Community Annotation] |
| AAEL005019 | | 1.0022 | -1.6245 | 0.4157 | 0.9996 | lactosylceramide 4-alpha-galactosyltransferase (alpha- 1,4-galactosyltransferase) [Source:VB Community Annotation] |
| AAEL007773 | | 0.4436 | 4.1271 | 0.4158 | 0.9996 | |
| AAEL007466 | | -0.3664 | 5.1866 | 0.4158 | 0.9996 | histone-fold protein CHRAC subunit, putative [Source:VB Community Annotation] |
| AAEL012098 | | -0.9527 | 1.5711 | 0.4158 | 0.9996 | synaptic vesicle protein [Source:VB Community Annotation] |
| AAEL027772 | NA | 0.8875 | 1.5228 | 0.4159 | 0.9996 | NA |
| AAEL003967 | | -0.5360 | 0.1186 | 0.4160 | 0.9996 | calpain 4, 6, 7, invertebrate [Source:VB Community Annotation] |
| AAEL003616 | | 0.8365 | -1.0004 | 0.4160 | 0.9996 | |
| AAEL002714 | | 0.9447 | -0.8074 | 0.4163 | 0.9996 | kinesin-like protein KIF23 (mitotic kinesin-like protein 1) [Source:VB Community Annotation] |
| AAEL019874 | NA | 0.9871 | -0.7219 | 0.4164 | 0.9996 | NA |
| AAEL006839 | | 0.2140 | 3.5693 | 0.4164 | 0.9996 | ring finger protein [Source:VB Community Annotation] |
| AAEL023898 | NA | -1.2901 | -1.0204 | 0.4164 | 0.9996 | NA |
| AAEL004042 | | 0.1609 | 6.5001 | 0.4165 | 0.9996 | |
| AAEL023241 | NA | -1.3766 | 0.0930 | 0.4165 | 0.9996 | NA |
| AAEL003510 | | 0.4444 | 1.5134 | 0.4166 | 0.9996 | ribonuclease hi large subunit [Source:VB Community Annotation] |
| AAEL005774 | | -1.0671 | -0.8655 | 0.4166 | 0.9996 | hect type E3 ubiquitin ligase [Source:VB Community Annotation] |
| AAEL007803 | | -0.7313 | 1.7115 | 0.4166 | 0.9996 | lamin b receptor [Source:VB Community Annotation] |
| AAEL001627 | | 0.5893 | 2.8199 | 0.4167 | 0.9996 | udp-n-acteylglucosamine pyrophosphorylase [Source:VB Community Annotation] |
| AAEL009101 | eIF3-S5 | -0.2138 | 8.7360 | 0.4167 | 0.9996 | Eukaryotic translation initiation factor 3 subunit F (eIF3f)(Eukaryotic translation initiation factor 3 subunit 5) [Source:VB Community Ann |
| AAEL005708 | | 0.2982 | 3.0694 | 0.4171 | 0.9996 | WD-repeat protein [Source:VB Community Annotation] |

| | | | | | | |
|---|---|---|---|---|---|---|
| AAEL005668 | | -0.7563 | -1.1261 | 0.4172 | 0.9996 | |
| AAEL013649 | | 0.5839 | 1.7142 | 0.4174 | 0.9996 | elongase, putative [Source:VB Community Annotation] |
| AAEL012724 | | -0.5308 | 0.7556 | 0.4175 | 0.9996 | |
| AAEL001738 | | -0.5081 | 3.2846 | 0.4177 | 0.9996 | MRAS2, putative [Source:VB Community Annotation] |
| AAEL020998 | NA | 0.3078 | 6.0353 | 0.4177 | 0.9996 | NA |
| AAEL001356 | | 0.1836 | 8.1619 | 0.4178 | 0.9996 | RNA-binding protein [Source:VB Community Annotation] |
| AAEL004155 | | 0.6143 | 3.9567 | 0.4182 | 0.9996 | |
| AAEL010422 | | 0.3515 | 2.6183 | 0.4183 | 0.9996 | replication-associated histone mRNA stem loop-binding protein, putative [Source:VB Community Annotation] |
| AAEL018109 | | -0.5283 | 4.8068 | 0.4184 | 0.9996 | |
| AAEL014311 | | -1.5965 | 0.4979 | 0.4185 | 0.9996 | tetraspanin, putative [Source:VB Community Annotation] |
| AAEL023294 | NA | 0.5874 | 0.7897 | 0.4185 | 0.9996 | NA |
| AAEL010816 | | 0.3477 | 3.1660 | 0.4185 | 0.9996 | prolyl-tRNA synthetase [Source:VB Community Annotation] |
| AAEL003367 | | -0.2483 | 4.5824 | 0.4187 | 0.9996 | nucleolar complex protein [Source:VB Community Annotation] |
| AAEL005315 | | -0.2279 | 4.7355 | 0.4189 | 0.9996 | |
| AAEL025089 | NA | -0.5201 | 3.9824 | 0.4190 | 0.9996 | NA |
| AAEL003374 | | 0.7851 | 1.1906 | 0.4191 | 0.9996 | Suppressor of fused homolog [Source:UniProtKB/TrEMBL;Acc:Q17FL2] |
| AAEL011126 | | -0.3575 | 7.5801 | 0.4192 | 0.9996 | alcohol dehydrogenase [Source:VB Community Annotation] |
| AAEL008843 | | 0.8535 | 0.5211 | 0.4192 | 0.9996 | |
| AAEL013528 | TPX1 | -0.2112 | 6.3954 | 0.4193 | 0.9996 | thioredoxin peroxidase [Source:VB Community Annotation] |
| AAEL002560 | | 0.5189 | 1.6793 | 0.4193 | 0.9996 | |
| AAEL007114 | | 1.3006 | 2.1875 | 0.4194 | 0.9996 | |
| AAEL019524 | NA | -0.4750 | 1.6908 | 0.4194 | 0.9996 | NA |
| AAEL014344 | | 0.3561 | 3.0556 | 0.4195 | 0.9996 | adam (a disintegrin and metalloprotease) [Source:VB Community Annotation] |
| AAEL015300 | | -0.3534 | 2.2026 | 0.4196 | 0.9996 | |
| AAEL027307 | NA | -0.8227 | 5.0625 | 0.4199 | 0.9996 | NA |
| AAEL005469 | | 0.8909 | -1.2605 | 0.4199 | 0.9996 | |
| AAEL011782 | | 0.3110 | 3.5352 | 0.4200 | 0.9996 | |
| AAEL010200 | | 0.3100 | 4.1605 | 0.4200 | 0.9996 | |
| AAEL022727 | NA | -0.8713 | 0.5349 | 0.4201 | 0.9996 | NA |
| AAEL005016 | | 0.3008 | 3.8908 | 0.4202 | 0.9996 | |
| AAEL001411 | | -0.6099 | 6.0871 | 0.4203 | 0.9996 | myosin heavy chain, nonmuscle or smooth muscle [Source:VB Community Annotation] |
| AAEL019753 | NA | -0.4139 | 5.3381 | 0.4204 | 0.9996 | NA |
| AAEL022698 | NA | -0.2769 | 3.2120 | 0.4205 | 0.9996 | NA |
| AAEL008831 | | 0.5605 | 0.5969 | 0.4207 | 0.9996 | |
| AAEL008299 | CTL11 | -0.4194 | 3.2031 | 0.4209 | 0.9996 | C-Type Lectin (CTL). [Source:VB Community Annotation] |
| AAEL028180 | NA | 0.3628 | 3.2077 | 0.4212 | 0.9996 | NA |
| AAEL022938 | NA | -0.2694 | 4.7198 | 0.4212 | 0.9996 | NA |
| AAEL006429 | | -0.9101 | -2.9279 | 0.4215 | 0.9996 | trypsin [Source:VB Community Annotation] |
| AAEL024128 | NA | -0.7243 | -0.0416 | 0.4216 | 0.9996 | NA |
| AAEL016966 | Or104 | -0.7202 | 0.2826 | 0.4216 | 0.9996 | Odorant receptor [Source:UniProtKB/TrEMBL;Acc:J9HTK1] |

| | | | | | | |
|---|---|---|---|---|---|---|
| AAEL015119 | | -0.4459 | 5.6156 | 0.4216 | 0.9996 | cuticle protein, putative [Source:VB Community Annotation] |
| AAEL015288 | | -1.2805 | -1.4865 | 0.4219 | 0.9996 | |
| AAEL007444 | | 0.3935 | 9.0330 | 0.4220 | 0.9996 | |
| AAEL019501 | NA | -0.9109 | -1.1019 | 0.4220 | 0.9996 | NA |
| AAEL027594 | NA | -0.2265 | 5.1429 | 0.4221 | 0.9996 | NA |
| AAEL019511 | NA | 0.2941 | 5.7178 | 0.4222 | 0.9996 | NA |
| AAEL008064 | | 0.2657 | 5.0555 | 0.4223 | 0.9996 | |
| AAEL013853 | CTLGA2 | 1.0180 | 3.9496 | 0.4223 | 0.9996 | C-Type Lectin (CTL) - galactose binding. [Source:VB Community Annotation] |
| AAEL010222 | | -0.4653 | 2.6787 | 0.4223 | 0.9996 | transcription factor GATA-4 (GATA binding factor-4) [Source:VB Community Annotation] |
| AAEL000032 | RpS6 | -0.2583 | 10.8796 | 0.4224 | 0.9996 | 40S ribosomal protein S6 [Source:UniProtKB/Swiss-Prot;Acc:Q9U761] |
| AAEL014263 | | 0.3344 | 3.4718 | 0.4225 | 0.9996 | |
| AAEL019480 | NA | 0.3011 | 4.9287 | 0.4225 | 0.9996 | NA |
| AAEL005913 | | 0.3589 | 3.0834 | 0.4227 | 0.9996 | |
| AAEL000922 | | 0.3772 | 2.6928 | 0.4228 | 0.9996 | polyribonucleotide nucleotidyltransferase [Source:VB Community Annotation] |
| AAEL011954 | | -0.8499 | 1.4729 | 0.4229 | 0.9996 | elongase, putative [Source:VB Community Annotation] |
| AAEL018303 | | 0.7273 | 1.6226 | 0.4231 | 0.9996 | |
| AAEL020608 | NA | 1.0913 | -1.6459 | 0.4231 | 0.9996 | NA |
| AAEL026427 | NA | -0.8995 | 0.1477 | 0.4232 | 0.9996 | NA |
| AAEL009628 | | -0.6292 | 0.6288 | 0.4233 | 0.9996 | short-chain dehydrogenase [Source:VB Community Annotation] |
| AAEL014231 | | 0.3847 | 3.6247 | 0.4235 | 0.9996 | |
| AAEL021859 | NA | -0.3882 | 2.6719 | 0.4235 | 0.9996 | NA |
| AAEL013739 | | -0.2261 | 6.8908 | 0.4235 | 0.9996 | electron transport oxidoreductase [Source:VB Community Annotation] |
| AAEL003210 | | 0.2815 | 7.9968 | 0.4236 | 0.9996 | tetraspanin 29fa [Source:VB Community Annotation] |
| AAEL004755 | | 0.6322 | 5.0330 | 0.4237 | 0.9996 | 3-2-trans-enoyl-CoA isomerase, putative [Source:VB Community Annotation] |
| AAEL007797 | | -0.4219 | 1.9607 | 0.4239 | 0.9996 | |
| AAEL000164 | | -0.5638 | 2.0182 | 0.4239 | 0.9996 | d-amino acid oxidase [Source:VB Community Annotation] |
| AAEL025607 | NA | 1.0453 | -0.9178 | 0.4240 | 0.9996 | NA |
| AAEL013399 | | -0.5448 | 0.3110 | 0.4241 | 0.9996 | |
| AAEL004480 | | -1.3048 | -0.3811 | 0.4243 | 0.9996 | cell division cycle 20 (cdc20) (fizzy) [Source:VB Community Annotation] |
| AAEL002301 | | -0.6997 | 7.1380 | 0.4244 | 0.9996 | serine protease [Source:VB Community Annotation] |
| AAEL006188 | | 0.9253 | 0.4020 | 0.4246 | 0.9996 | |
| AAEL003083 | | -1.0663 | -0.3468 | 0.4247 | 0.9996 | myotubularin [Source:VB Community Annotation] |
| AAEL013713 | | -0.5771 | 6.5218 | 0.4247 | 0.9996 | trypsin [Source:VB Community Annotation] |
| AAEL013884 | | -0.5990 | 0.8193 | 0.4247 | 0.9996 | synaptic vesicle protein [Source:VB Community Annotation] |
| AAEL002282 | | -0.3473 | 3.3346 | 0.4247 | 0.9996 | zinc finger protein [Source:VB Community Annotation] |
| AAEL004438 | | 0.3099 | 6.8449 | 0.4247 | 0.9996 | GrpE protein homolog [Source:UniProtKB/TrEMBL;Acc:Q17CT4] |
| AAEL013688 | | 0.6171 | 1.1742 | 0.4247 | 0.9996 | |
| AAEL003174 | | -0.3265 | 4.9262 | 0.4249 | 0.9996 | translation initiation factor 1A, putative [Source:VB Community Annotation] |
| AAEL003056 | | 1.2635 | -0.2269 | 0.4249 | 0.9996 | brain chitinase and chia [Source:VB Community Annotation] |
| AAEL021091 | NA | -1.0865 | -2.0561 | 0.4252 | 0.9996 | NA |

| | | | | | | |
|---|---|---|---|---|---|---|
| AAEL027914 | NA | 0.2213 | 5.9108 | 0.4252 | 0.9996 | NA |
| AAEL001511 | | 0.6189 | 5.7714 | 0.4252 | 0.9996 | |
| AAEL014037 | | -0.2764 | 4.3053 | 0.4253 | 0.9996 | PAF acetylhydrolase 45 kDa subunit, putative [Source:VB Community Annotation] |
| AAEL007874 | | -0.1966 | 6.3906 | 0.4253 | 0.9996 | |
| AAEL023822 | NA | 0.4157 | 3.4451 | 0.4253 | 0.9996 | NA |
| AAEL014252 | | 0.3777 | 4.3737 | 0.4255 | 0.9996 | sulfite reductase [Source:VB Community Annotation] |
| AAEL001346 | | -0.2515 | 6.6127 | 0.4256 | 0.9996 | |
| AAEL021218 | NA | -0.9457 | 0.2455 | 0.4256 | 0.9996 | NA |
| AAEL002097 | | 0.3091 | 5.4927 | 0.4256 | 0.9996 | |
| AAEL006950 | | 0.3217 | 4.1555 | 0.4258 | 0.9996 | |
| AAEL007048 | | -0.3712 | 3.4167 | 0.4258 | 0.9996 | |
| AAEL001031 | | -0.2521 | 6.8720 | 0.4258 | 0.9996 | |
| AAEL020706 | NA | -0.5176 | 4.8258 | 0.4260 | 0.9996 | NA |
| AAEL009092 | | 0.2240 | 5.5230 | 0.4263 | 0.9996 | longevity assurance factor 1 (lag1) [Source:VB Community Annotation] |
| AAEL019855 | NA | -0.5314 | 5.2758 | 0.4263 | 0.9996 | NA |
| AAEL024902 | NA | -0.4719 | 1.3895 | 0.4264 | 0.9996 | NA |
| AAEL027264 | NA | -0.3436 | 1.3401 | 0.4265 | 0.9996 | NA |
| AAEL005470 | | -0.3509 | 3.2407 | 0.4266 | 0.9996 | peroxisomal membrane protein, putative [Source:VB Community Annotation] |
| AAEL026331 | NA | 0.5845 | 2.7265 | 0.4266 | 0.9996 | NA |
| AAEL010395 | | -0.2159 | 5.5024 | 0.4272 | 0.9996 | beat protein [Source:VB Community Annotation] |
| AAEL002538 | | -0.6182 | 3.6144 | 0.4273 | 0.9996 | glutamate receptor, ionotropic kainate 1, 2, 3 (glur5, glur6, glur7) [Source:VB Community Annotation] |
| AAEL017380 | | -0.8415 | 5.4681 | 0.4274 | 0.9996 | |
| AAEL027927 | NA | -0.3752 | 6.5313 | 0.4276 | 0.9996 | NA |
| AAEL009971 | | 0.4266 | 4.6236 | 0.4276 | 0.9996 | |
| AAEL012392 | | 0.3507 | 1.6760 | 0.4278 | 0.9996 | |
| AAEL005641 | CTLGA5 | -0.8944 | 3.6942 | 0.4278 | 0.9996 | C-Type Lectin (CTL) - galactose binding. [Source:VB Community Annotation] |
| AAEL002454 | | 0.4919 | 1.1472 | 0.4279 | 0.9996 | |
| AAEL008090 | | -1.0867 | 0.3689 | 0.4280 | 0.9996 | glutamate receptor, ionotropic, n-methyl d-aspartate epsilon (nmda epsilon) [Source:VB Community Annotation] |
| AAEL002910 | | 0.9429 | 1.0967 | 0.4280 | 0.9996 | |
| AAEL019489 | NA | -1.1663 | -0.4188 | 0.4281 | 0.9996 | NA |
| AAEL025164 | NA | -0.8670 | -0.6687 | 0.4284 | 0.9996 | NA |
| AAEL025635 | NA | -0.2985 | 3.5228 | 0.4285 | 0.9996 | NA |
| AAEL005832 | | -0.2922 | 7.0225 | 0.4289 | 0.9996 | programmed cell death [Source:VB Community Annotation] |
| AAEL027142 | NA | -0.5625 | 3.1765 | 0.4289 | 0.9996 | NA |
| AAEL001833 | | 0.6432 | 2.2879 | 0.4290 | 0.9996 | juvenile hormone-inducible protein, putative [Source:VB Community Annotation] |
| AAEL029010 | NA | -0.2173 | 6.7173 | 0.4290 | 0.9996 | NA |
| AAEL013776 | | 0.2362 | 6.7716 | 0.4292 | 0.9996 | |
| AAEL007042 | | 0.2571 | 7.9152 | 0.4293 | 0.9996 | far upstream (fuse) binding protein [Source:VB Community Annotation] |
| AAEL027226 | NA | -0.7025 | 1.5098 | 0.4294 | 0.9996 | NA |
| AAEL007681 | | 0.2319 | 8.7282 | 0.4297 | 0.9996 | NADH-ubiquinone oxidoreductase flavoprotein 1 (ndufv1) [Source:VB Community Annotation] |

| | | | | | | |
|---|---|---|---|---|---|---|
| AAEL022787 | NA | 0.2957 | 5.1340 | 0.4297 | 0.9996 | NA |
| AAEL026619 | NA | 0.6938 | 3.9056 | 0.4297 | 0.9996 | NA |
| AAEL014149 | | 0.2890 | 3.3167 | 0.4298 | 0.9996 | |
| AAEL024781 | NA | -0.6225 | -0.1646 | 0.4298 | 0.9996 | NA |
| AAEL010128 | LRIM4 | -0.4670 | 5.8584 | 0.4298 | 0.9996 | leucine-rich immune protein (Long) [Source:VB Community Annotation] |
| AAEL002137 | | 0.2720 | 4.9237 | 0.4298 | 0.9996 | transcriptional adaptor 2 (ada2) [Source:VB Community Annotation] |
| AAEL001515 | DEBCL | 0.3573 | 3.2902 | 0.4301 | 0.9996 | autophagy related gene DEBCL [Source:VB Community Annotation] |
| AAEL001420 | LRIM8 | -0.5737 | 8.4074 | 0.4302 | 0.9996 | leucine-rich immune protein (Short) [Source:VB Community Annotation] |
| AAEL011055 | | -0.2569 | 6.3892 | 0.4302 | 0.9996 | chaperone protein DNAj [Source:VB Community Annotation] |
| AAEL002077 | | -0.4801 | 5.3260 | 0.4303 | 0.9996 | UV excision repair protein rad23 [Source:VB Community Annotation] |
| AAEL005216 | | 0.3444 | 3.7285 | 0.4304 | 0.9996 | |
| AAEL006637 | | 0.2696 | 3.9735 | 0.4305 | 0.9996 | |
| AAEL001101 | | -0.2661 | 5.0680 | 0.4307 | 0.9996 | ATP-dependent transporter [Source:VB Community Annotation] |
| AAEL007593 | CLIPC2 | -0.2482 | 7.2322 | 0.4307 | 0.9996 | Clip-Domain Serine Protease family C. [Source:VB Community Annotation] |
| AAEL003758 | | 0.6852 | 2.4159 | 0.4309 | 0.9996 | Sorting nexin [Source:UniProtKB/TrEMBL;Acc:Q0IG11] |
| AAEL001142 | | -0.6461 | 2.0276 | 0.4309 | 0.9996 | rab gdp/GTP exchange factor [Source:VB Community Annotation] |
| AAEL004701 | | 0.9428 | 1.7886 | 0.4309 | 0.9996 | Argininosuccinate synthase (EC 6.3.4.5)(Citrulline--aspartate ligase) [Source:VB Community Annotation] |
| AAEL002435 | | 0.1840 | 5.9596 | 0.4310 | 0.9996 | |
| AAEL009745 | | -1.3552 | 0.0977 | 0.4310 | 0.9996 | Nitric oxide synthase [Source:UniProtKB/TrEMBL;Acc:Q16UY1] |
| AAEL013548 | | -0.6342 | 2.1078 | 0.4311 | 0.9996 | |
| AAEL011118 | | 0.3384 | 1.7220 | 0.4312 | 0.9996 | dual specificity protein kinase [Source:VB Community Annotation] |
| AAEL027261 | NA | -0.6563 | 0.8299 | 0.4316 | 0.9996 | NA |
| AAEL021703 | NA | 0.7233 | 3.5947 | 0.4316 | 0.9996 | NA |
| AAEL005843 | | -0.7378 | 5.4252 | 0.4317 | 0.9996 | |
| AAEL018181 | | 0.4394 | 3.4923 | 0.4320 | 0.9996 | |
| AAEL025150 | NA | 0.6338 | 0.5034 | 0.4321 | 0.9996 | NA |
| AAEL026582 | NA | -0.6184 | 3.9544 | 0.4321 | 0.9996 | NA |
| AAEL009023 | | 0.5368 | 2.6075 | 0.4323 | 0.9996 | |
| AAEL011233 | | -0.3122 | 4.8224 | 0.4323 | 0.9996 | SM protein G, putative [Source:VB Community Annotation] |
| AAEL003063 | | -0.6196 | 3.9105 | 0.4324 | 0.9996 | semaphorin [Source:VB Community Annotation] |
| AAEL000442 | osk | -1.2715 | -0.6078 | 0.4325 | 0.9996 | protein oskar [Source:VB Community Annotation] |
| AAEL012093 | | -1.1130 | -1.2199 | 0.4325 | 0.9996 | leucine-rich transmembrane protein [Source:VB Community Annotation] |
| AAEL006350 | | 0.2988 | 2.1210 | 0.4326 | 0.9996 | |
| AAEL017536 | GRRP | -0.5868 | 12.6714 | 0.4328 | 0.9996 | holotricin glycine rich repeat protein (GRRP) anti-microbial peptide [Source:VB Community Annotation] |
| AAEL004063 | | -0.3554 | 4.1665 | 0.4328 | 0.9996 | WD-repeat protein [Source:VB Community Annotation] |
| AAEL009374 | | -0.2700 | 2.9211 | 0.4330 | 0.9996 | |
| AAEL007029 | | 0.2904 | 5.8732 | 0.4330 | 0.9996 | tropomodulin [Source:VB Community Annotation] |
| AAEL026357 | NA | 1.3458 | 2.8203 | 0.4331 | 0.9996 | NA |
| AAEL010893 | | -1.1894 | 1.5399 | 0.4332 | 0.9996 | carbonic anhydrase [Source:VB Community Annotation] |
| AAEL005364 | | 0.8539 | 0.2451 | 0.4332 | 0.9996 | adaptin, alpha/gamma/epsilon [Source:VB Community Annotation] |

| | | | | | | |
|---|---|---|---|---|---|---|
| AAEL003976 | | -0.2608 | 7.0957 | 0.4334 | 0.9996 | |
| AAEL017460 | | -0.7430 | 8.0937 | 0.4338 | 0.9996 | |
| AAEL005717 | | 0.2624 | 3.5828 | 0.4340 | 0.9996 | |
| AAEL010842 | | 0.2939 | 3.9729 | 0.4341 | 0.9996 | |
| AAEL023581 | NA | -1.1630 | -0.7664 | 0.4341 | 0.9996 | NA |
| AAEL005796 | | -0.1767 | 4.8454 | 0.4342 | 0.9996 | eukaryotic translation initiation factor 4e type [Source:VB Community Annotation] |
| AAEL020625 | NA | -0.1847 | 5.4908 | 0.4343 | 0.9996 | NA |
| AAEL000926 | | 0.8926 | -0.6201 | 0.4343 | 0.9996 | pickpocket [Source:VB Community Annotation] |
| AAEL008000 | | 0.2687 | 5.6919 | 0.4344 | 0.9996 | golgi reassembly stacking protein 2 (grasp2) [Source:VB Community Annotation] |
| AAEL025126 | NA | -0.7913 | 5.8530 | 0.4345 | 0.9996 | NA |
| AAEL009913 | | -0.5818 | 3.2414 | 0.4345 | 0.9996 | DEAD box ATP-dependent RNA helicase [Source:VB Community Annotation] |
| AAEL027572 | NA | 0.4748 | 0.5309 | 0.4345 | 0.9996 | NA |
| AAEL026746 | NA | -0.3905 | 1.6265 | 0.4345 | 0.9996 | NA |
| AAEL019767 | NA | -0.4108 | 7.2138 | 0.4347 | 0.9996 | NA |
| AAEL002113 | | 0.2959 | 4.6540 | 0.4348 | 0.9996 | 3-oxoacyl-[acyl-carrier-protein] synthase [Source:UniProtKB/TrEMBL;Acc:Q17J59] |
| AAEL019559 | NA | 0.3844 | 5.8362 | 0.4349 | 0.9996 | NA |
| AAEL003690 | | 0.4197 | 1.5864 | 0.4349 | 0.9996 | |
| AAEL011106 | | -0.2435 | 5.6881 | 0.4351 | 0.9996 | |
| AAEL003243 | CLIPB13A | -0.4137 | 5.6952 | 0.4351 | 0.9996 | Clip-Domain Serine Protease  family B. [Source:VB Community Annotation] |
| AAEL001583 | | 0.3901 | 3.8513 | 0.4352 | 0.9996 | |
| AAEL000254 | | 0.3442 | 3.4635 | 0.4352 | 0.9996 | nuclear factor i [Source:VB Community Annotation] |
| AAEL008492 | | -0.5211 | 4.5229 | 0.4353 | 0.9996 | |
| AAEL012926 | | 0.9707 | 0.6796 | 0.4355 | 0.9996 | |
| AAEL012837 | | -1.0615 | -0.9706 | 0.4355 | 0.9996 | |
| AAEL002805 | | 0.3270 | 3.3480 | 0.4357 | 0.9996 | |
| AAEL013840 | | -0.2200 | 5.9246 | 0.4358 | 0.9996 | |
| AAEL012509 | | -0.4579 | 6.1728 | 0.4361 | 0.9996 | Carboxylic ester hydrolase (Fragment) [Source:UniProtKB/TrEMBL;Acc:Q16LV6] |
| AAEL008108 | | -0.2579 | 9.8387 | 0.4363 | 0.9996 | guanine nucleotide-binding protein beta 2 (g protein beta2) [Source:VB Community Annotation] |
| AAEL001903 | | 0.5919 | 0.7277 | 0.4364 | 0.9996 | |
| AAEL019513 | NA | -0.3124 | 5.3938 | 0.4365 | 0.9996 | NA |
| AAEL011340 | | 0.2341 | 6.6132 | 0.4365 | 0.9996 | U1 small nuclear ribonucleoprotein 70 kd [Source:VB Community Annotation] |
| AAEL010172 | | 0.3925 | 3.4190 | 0.4366 | 0.9996 | Leucokinins Precursor [Contains Leucokinin-1;Leucokinin-3;Leucokinin-2] [Source:VB Community Annotation] |
| AAEL009661 | | 0.2038 | 6.6776 | 0.4367 | 0.9996 | |
| AAEL008169 | RpS12 | 0.3242 | 5.3292 | 0.4367 | 0.9996 | 40S ribosomal protein S12 [Source:VB Community Annotation] |
| AAEL006022 | | 0.2272 | 3.9912 | 0.4368 | 0.9996 | myotubularin [Source:VB Community Annotation] |
| AAEL009328 | | 0.9908 | -0.9360 | 0.4369 | 0.9996 | hydroxyacyl dehydrogenase [Source:VB Community Annotation] |
| AAEL009991 | | -0.6336 | 6.2231 | 0.4369 | 0.9996 | myosin iii [Source:VB Community Annotation] |
| AAEL022898 | NA | -1.1864 | -2.7751 | 0.4370 | 0.9996 | NA |
| AAEL005946 | | -0.2757 | 7.4668 | 0.4371 | 0.9996 | NADH-ubiquinone oxidoreductase subunit B14.5b [Source:VB Community Annotation] |
| AAEL023495 | NA | -1.2645 | -0.8776 | 0.4371 | 0.9996 | NA |

| | | | | | | |
|---|---|---|---|---|---|---|
| AAEL012866 | | 1.0063 | 0.1668 | 0.4372 | 0.9996 | |
| AAEL013936 | SRPN4 | -0.2537 | 6.9467 | 0.4376 | 0.9996 | Serine Protease Inhibitor (serpin)  likely cleavage at I/S. Transcript A. [Source:VB Community Annotation] |
| AAEL007903 | | -0.4540 | 1.5385 | 0.4376 | 0.9996 | |
| AAEL001737 | | 1.4282 | -0.9148 | 0.4377 | 0.9996 | Fatty acyl-CoA reductase (Fragment) [Source:UniProtKB/TrEMBL;Acc:Q17KB2] |
| AAEL003536 | | 0.3158 | 4.1146 | 0.4377 | 0.9996 | E3 UFM1-protein ligase 1 homolog (EC 6.3.2.-) [Source:VB Community Annotation] |
| AAEL008501 | | -0.3341 | 3.6308 | 0.4380 | 0.9996 | |
| AAEL007030 | | -0.2167 | 6.1957 | 0.4381 | 0.9996 | ceramidase [Source:VB Community Annotation] |
| AAEL000999 | | -0.3024 | 3.1609 | 0.4381 | 0.9996 | DNA replication licensing factor MCM7 [Source:VB Community Annotation] |
| AAEL015079 | | -0.9929 | -1.0529 | 0.4382 | 0.9996 | |
| AAEL012081 | | 0.2417 | 4.8098 | 0.4383 | 0.9996 | |
| AAEL000193 | | 0.3010 | 4.1616 | 0.4383 | 0.9996 | Histone-lysine N-methyltransferase [Source:UniProtKB/TrEMBL;Acc:Q17PZ6] |
| AAEL012739 | | 0.3364 | 4.2528 | 0.4383 | 0.9996 | |
| AAEL020528 | NA | -0.1599 | 4.4774 | 0.4384 | 0.9996 | NA |
| AAEL005845 | | -0.5944 | 5.9998 | 0.4386 | 0.9996 | beta chain spectrin [Source:VB Community Annotation] |
| AAEL008046 | | -0.2271 | 5.3030 | 0.4387 | 0.9996 | rh antigen [Source:VB Community Annotation] |
| AAEL011042 | | -0.9085 | 1.4890 | 0.4387 | 0.9996 | |
| AAEL007595 | | 0.2651 | 4.3945 | 0.4389 | 0.9996 | |
| AAEL026933 | NA | -1.2306 | -0.3549 | 0.4389 | 0.9996 | NA |
| AAEL002948 | | -0.2256 | 4.6178 | 0.4389 | 0.9996 | frataxin, putative [Source:VB Community Annotation] |
| AAEL019862 | NA | 0.3406 | 4.8261 | 0.4390 | 0.9996 | NA |
| AAEL023509 | NA | -1.0817 | -1.0622 | 0.4390 | 0.9996 | NA |
| AAEL005258 | | 0.2039 | 5.3142 | 0.4392 | 0.9996 | syntaxin, putative [Source:VB Community Annotation] |
| AAEL001850 | | 0.4861 | 1.0616 | 0.4392 | 0.9996 | |
| AAEL007424 | | 0.9904 | -0.9524 | 0.4396 | 0.9996 | phospholipid scramblase, putative [Source:VB Community Annotation] |
| AAEL014099 | | -0.4070 | 2.3987 | 0.4396 | 0.9996 | valyl-tRNA synthetase [Source:VB Community Annotation] |
| AAEL010653 | | -0.2292 | 5.0057 | 0.4396 | 0.9996 | alpha-l-fucosidase [Source:VB Community Annotation] |
| AAEL009513 | | -1.2691 | 0.2311 | 0.4397 | 0.9996 | adenylate cyclase [Source:VB Community Annotation] |
| AAEL005390 | | 0.2136 | 4.0538 | 0.4401 | 0.9996 | importin [Source:VB Community Annotation] |
| AAEL011597 | | 0.3245 | 4.2239 | 0.4401 | 0.9996 | |
| AAEL008901 | | -0.3581 | 3.3921 | 0.4401 | 0.9996 | |
| AAEL010336 | | 0.2723 | 3.6775 | 0.4402 | 0.9996 | zinc phosphodiesterase [Source:VB Community Annotation] |
| AAEL012390 | | 0.3470 | 4.4246 | 0.4403 | 0.9996 | |
| AAEL013812 | | -1.0888 | -1.9946 | 0.4403 | 0.9996 | |
| AAEL002804 | | 0.2092 | 4.5262 | 0.4404 | 0.9996 | oligosaccharyl transferase [Source:VB Community Annotation] |
| AAEL002534 | RpL10 | -0.3567 | 9.6175 | 0.4407 | 0.9996 | 60S ribosomal protein L10 [Source:VB Community Annotation] |
| AAEL024259 | NA | -1.0683 | -2.2099 | 0.4407 | 0.9996 | NA |
| AAEL006678 | | 0.2880 | 4.9336 | 0.4410 | 0.9996 | |
| AAEL000147 | | -0.4702 | 2.7342 | 0.4411 | 0.9996 | single-stranded DNA binding protein, putative [Source:VB Community Annotation] |
| AAEL011160 | | -0.9107 | -0.6227 | 0.4411 | 0.9996 | |
| AAEL022917 | NA | -0.6800 | 1.6556 | 0.4412 | 0.9996 | NA |

| | | | | | | |
|---|---|---|---|---|---|---|
| AAEL017005 | GPRNPY6 | 0.7991 | -0.0239 | 0.4417 | 0.9996 | GPCR Neuropeptide Y Family [Source:VB Community Annotation] |
| AAEL021126 | NA | -0.3195 | 3.4746 | 0.4418 | 0.9996 | NA |
| AAEL009919 | | 0.2538 | 3.5347 | 0.4419 | 0.9996 | zinc finger protein [Source:VB Community Annotation] |
| AAEL004306 | mRpL19 | -0.2735 | 5.1649 | 0.4419 | 0.9996 | 39S ribosomal protein L19, mitochondrial precursor [Source:VB Community Annotation] |
| AAEL003897 | | -0.7056 | 0.3777 | 0.4421 | 0.9996 | DNA repair protein xp-c / rad4 [Source:VB Community Annotation] |
| AAEL016998 | | -0.8276 | 0.4065 | 0.4421 | 0.9996 | |
| AAEL008543 | Cdc42 | -0.9360 | 5.0832 | 0.4422 | 0.9996 | Cdc42 homolog Precursor [Source:VB Community Annotation] |
| AAEL014350 | | -0.5099 | 4.8828 | 0.4424 | 0.9996 | |
| AAEL019845 | NA | 0.3294 | 5.4072 | 0.4426 | 0.9996 | NA |
| AAEL000027 | | -0.6654 | -0.2989 | 0.4426 | 0.9996 | |
| AAEL009698 | GPRNNA7 | -1.1587 | 1.8670 | 0.4427 | 0.9996 | GPCR Orphan/Putative Class A Family [Source:VB Community Annotation] |
| AAEL019764 | NA | 0.2312 | 3.3511 | 0.4427 | 0.9996 | NA |
| AAEL008124 | | 0.4665 | 2.0826 | 0.4428 | 0.9996 | possible RNA methyltransferase, putative [Source:VB Community Annotation] |
| AAEL025134 | NA | -0.8467 | 3.3518 | 0.4433 | 0.9996 | NA |
| AAEL002818 | | 0.5457 | 2.0985 | 0.4434 | 0.9996 | splicing factor u2af large subunit [Source:VB Community Annotation] |
| AAEL014457 | | -0.3434 | 6.1639 | 0.4434 | 0.9996 | neuronal cell adhesion molecule [Source:VB Community Annotation] |
| AAEL024339 | NA | -1.0683 | -2.0135 | 0.4435 | 0.9996 | NA |
| AAEL023634 | NA | 0.5342 | 7.9922 | 0.4436 | 0.9996 | NA |
| AAEL018124 | | 0.3600 | 2.4296 | 0.4436 | 0.9996 | |
| AAEL013982 | | 0.3570 | 3.8832 | 0.4437 | 0.9996 | RNA binding motif protein 4,lark [Source:VB Community Annotation] |
| AAEL011794 | | -1.2023 | -1.2961 | 0.4438 | 0.9996 | |
| AAEL007915 | | -0.3916 | 7.7283 | 0.4438 | 0.9996 | Moesin/ezrin/radixin homolog 1 [Source:VB Community Annotation] |
| AAEL003151 | | 0.3360 | 4.0323 | 0.4439 | 0.9996 | |
| AAEL001169 | | 0.3018 | 4.0473 | 0.4440 | 0.9996 | Ribosome biogenesis protein BOP1 homolog [Source:UniProtKB/Swiss-Prot;Acc:Q17LZ2] |
| AAEL003214 | | 0.5139 | 0.3672 | 0.4441 | 0.9996 | salivary gland growth factor [Source:VB Community Annotation] |
| AAEL000984 | | -1.4462 | 1.2762 | 0.4442 | 0.9996 | |
| AAEL001143 | | 0.4327 | 3.3509 | 0.4443 | 0.9996 | |
| AAEL001368 | | -0.3528 | 5.7934 | 0.4443 | 0.9996 | kek1 [Source:VB Community Annotation] |
| AAEL019820 | NA | -1.5663 | 1.2171 | 0.4443 | 0.9996 | NA |
| AAEL003322 | | -1.3484 | -0.5688 | 0.4443 | 0.9996 | protein phosphatase-7 [Source:VB Community Annotation] |
| AAEL005175 | | 0.2254 | 6.3696 | 0.4444 | 0.9996 | lipin [Source:VB Community Annotation] |
| AAEL027674 | NA | -0.9432 | 5.0782 | 0.4445 | 0.9996 | NA |
| AAEL013167 | | 0.2342 | 3.6240 | 0.4446 | 0.9996 | |
| AAEL019793 | NA | -0.7037 | 7.5806 | 0.4447 | 0.9996 | NA |
| AAEL025433 | NA | -0.2932 | 4.8087 | 0.4447 | 0.9996 | NA |
| AAEL005536 | | 0.7074 | 3.9591 | 0.4450 | 0.9996 | tetraspanin 29fb [Source:VB Community Annotation] |
| AAEL001299 | | -0.3098 | 4.0090 | 0.4452 | 0.9996 | |
| AAEL001448 | | 0.2054 | 5.5629 | 0.4453 | 0.9996 | |
| AAEL026452 | NA | 0.2670 | 3.5640 | 0.4454 | 0.9996 | NA |
| AAEL006951 | | 0.2123 | 6.7916 | 0.4456 | 0.9996 | protein phosphatase 2c [Source:VB Community Annotation] |

| | | | | | | |
|---|---|---|---|---|---|---|
| AAEL010054 | | 0.2925 | 3.5681 | 0.4456 | 0.9996 | |
| AAEL023759 | NA | 0.4011 | 3.1792 | 0.4456 | 0.9996 | NA |
| AAEL007487 | | -1.2490 | 0.3272 | 0.4457 | 0.9996 | |
| AAEL008257 | | -0.4997 | 7.4741 | 0.4457 | 0.9996 | heterogeneous nuclear ribonucleoprotein 27c [Source:VB Community Annotation] |
| AAEL017113 | | -0.5338 | 6.4726 | 0.4458 | 0.9996 | H/ACA ribonucleoprotein complex subunit [Source:UniProtKB/TrEMBL;Acc:J9EAK1] |
| AAEL009708 | | 0.3624 | 7.4709 | 0.4461 | 0.9996 | thioredoxin M, putative [Source:VB Community Annotation] |
| AAEL005213 | | -0.3606 | 5.4101 | 0.4461 | 0.9996 | |
| AAEL023500 | NA | 0.2204 | 6.1416 | 0.4463 | 0.9996 | NA |
| AAEL013515 | | -0.5233 | 9.5376 | 0.4463 | 0.9996 | pupal cuticle protein, putative [Source:VB Community Annotation] |
| AAEL001626 | | -0.8691 | 3.2542 | 0.4464 | 0.9996 | zinc/iron transporter [Source:VB Community Annotation] |
| AAEL019532 | NA | -1.4142 | 0.6469 | 0.4465 | 0.9996 | NA |
| AAEL020440 | NA | 0.3390 | 3.7023 | 0.4465 | 0.9996 | NA |
| AAEL006014 | HPX1 | 0.8379 | 1.4091 | 0.4466 | 0.9996 | heme peroxidase [Source:VB Community Annotation] |
| AAEL001936 | | 0.6401 | 1.3763 | 0.4466 | 0.9996 | |
| AAEL005645 | | -0.3542 | 4.3556 | 0.4467 | 0.9996 | |
| AAEL019568 | NA | -0.3740 | 9.7252 | 0.4467 | 0.9996 | NA |
| AAEL000704 | | -0.9635 | 7.4518 | 0.4468 | 0.9996 | synaptotagmin, [Source:VB Community Annotation] |
| AAEL011102 | | 0.1892 | 6.5027 | 0.4470 | 0.9996 | lysosomal-associated transmembrane protein [Source:VB Community Annotation] |
| AAEL006117 | mRpL12 | -0.3378 | 6.7326 | 0.4471 | 0.9996 | 39S ribosomal protein L12, mitochondrial [Source:VB Community Annotation] |
| AAEL001241 | | -0.2870 | 5.1155 | 0.4472 | 0.9996 | |
| AAEL018308 | | 0.2709 | 4.6252 | 0.4475 | 0.9996 | |
| AAEL008562 | | -0.5776 | 4.1940 | 0.4475 | 0.9996 | |
| AAEL014414 | | 0.2437 | 5.7304 | 0.4475 | 0.9996 | DEAD box ATP-dependent RNA helicase [Source:VB Community Annotation] |
| AAEL010596 | | -0.2662 | 6.3373 | 0.4477 | 0.9996 | n-acetylgalactosaminyltransferase [Source:VB Community Annotation] |
| AAEL020078 | NA | 0.7674 | 1.7147 | 0.4478 | 0.9996 | NA |
| AAEL012151 | | -0.3822 | 5.5597 | 0.4478 | 0.9996 | ral guanine nucleotide exchange factor  with ph domain and sh3 binding motif, ralgps [Source:VB Community Annotation] |
| AAEL002208 | | 0.7608 | -0.6631 | 0.4479 | 0.9996 | |
| AAEL003887 | | -0.7373 | 1.7780 | 0.4481 | 0.9996 | vacuolar membrane protein pep11 [Source:VB Community Annotation] |
| AAEL023923 | NA | -0.5612 | 1.1099 | 0.4483 | 0.9996 | NA |
| AAEL004581 | | 0.3735 | 3.6838 | 0.4483 | 0.9996 | |
| AAEL002019 | | -0.2202 | 4.2705 | 0.4484 | 0.9996 | |
| AAEL000804 | | 0.2688 | 3.9892 | 0.4484 | 0.9996 | |
| AAEL002438 | | -0.7345 | 1.1088 | 0.4486 | 0.9996 | Protein quiver [Source:UniProtKB/TrEMBL;Acc:A0A1S4F1U2] |
| AAEL010063 | | 0.2424 | 5.3710 | 0.4487 | 0.9996 | chaperonin [Source:VB Community Annotation] |
| AAEL002502 | | 0.1990 | 5.1908 | 0.4487 | 0.9996 | |
| AAEL021261 | NA | -0.3830 | 3.8075 | 0.4489 | 0.9996 | NA |
| AAEL005305 | | -0.4773 | 5.2057 | 0.4490 | 0.9996 | |
| AAEL003770 | | -0.3212 | 4.4025 | 0.4491 | 0.9996 | |
| AAEL012279 | | -0.2978 | 6.7000 | 0.4492 | 0.9996 | Eukaryotic translation initiation factor 3 subunit J (eIF3j) [Source:VB Community Annotation] |
| AAEL001899 | | -0.2691 | 5.9880 | 0.4492 | 0.9996 | juvenile hormone-inducible protein, putative [Source:VB Community Annotation] |

| | | | | | | |
|---|---|---|---|---|---|---|
| AAEL015115 | | 0.2857 | 2.4911 | 0.4493 | 0.9996 | |
| AAEL003419 | | -1.4069 | 2.6511 | 0.4494 | 0.9996 | |
| AAEL014283 | | -0.3445 | 3.6311 | 0.4494 | 0.9996 | |
| AAEL023374 | NA | -1.6845 | 1.1701 | 0.4496 | 0.9996 | NA |
| AAEL025822 | NA | 0.2938 | 5.6967 | 0.4499 | 0.9996 | NA |
| AAEL000562 | | 0.1577 | 4.5110 | 0.4500 | 0.9996 | |
| AAEL013276 | | -0.5132 | 2.3080 | 0.4500 | 0.9996 | acid phosphatase [Source:VB Community Annotation] |
| AAEL019894 | NA | 0.5458 | 0.3111 | 0.4500 | 0.9996 | NA |
| AAEL020233 | NA | -1.0093 | 1.3460 | 0.4502 | 0.9996 | NA |
| AAEL000005 | | 0.2766 | 2.7286 | 0.4502 | 0.9996 | |
| AAEL010778 | | -0.3145 | 6.0252 | 0.4503 | 0.9996 | |
| AAEL012660 | | 0.3816 | 3.1783 | 0.4504 | 0.9996 | |
| AAEL013279 | | -0.2239 | 9.8501 | 0.4507 | 0.9996 | peptidyl-prolyl cis-trans isomerase (cyclophilin) [Source:VB Community Annotation] |
| AAEL011200 | | 0.3994 | 4.2253 | 0.4507 | 0.9996 | |
| AAEL000343 | | -0.2357 | 4.6058 | 0.4507 | 0.9996 | serine/threonine-protein kinase vrk [Source:VB Community Annotation] |
| AAEL014981 | | 0.3142 | 2.8886 | 0.4507 | 0.9996 | |
| AAEL003935 | | 0.2179 | 3.8986 | 0.4509 | 0.9996 | |
| AAEL001275 | | 0.1908 | 6.8586 | 0.4509 | 0.9996 | ef-hand protein nucb1 [Source:VB Community Annotation] |
| AAEL003743 | | 0.1724 | 6.2763 | 0.4509 | 0.9996 | vacuolar proton ATPases [Source:VB Community Annotation] |
| AAEL005816 | | -0.3518 | 3.4922 | 0.4510 | 0.9996 | chromatin regulatory protein sir2 [Source:VB Community Annotation] |
| AAEL008028 | | 0.4542 | 4.4993 | 0.4510 | 0.9996 | monocarboxylate transporter [Source:VB Community Annotation] |
| AAEL003954 | | -0.3302 | 5.0886 | 0.4511 | 0.9996 | juvenile hormone-inducible protein, putative [Source:VB Community Annotation] |
| AAEL018296 | | 0.3502 | 2.7434 | 0.4514 | 0.9996 | |
| AAEL007971 | | 0.2777 | 5.3845 | 0.4517 | 0.9996 | tyrosine transporter [Source:VB Community Annotation] |
| AAEL014880 | | 0.3222 | 2.7668 | 0.4518 | 0.9996 | |
| AAEL006280 | JAHMT | 0.8656 | 2.8987 | 0.4518 | 0.9996 | juvenile hormone acid methyltransferase, putative [Source:VB Community Annotation] |
| AAEL011974 | | -0.2378 | 5.6931 | 0.4519 | 0.9996 | |
| AAEL008639 | | -0.5332 | 6.4692 | 0.4520 | 0.9996 | |
| AAEL007726 | | 0.5113 | 0.2309 | 0.4520 | 0.9996 | |
| AAEL005185 | | 0.3837 | 3.3400 | 0.4521 | 0.9996 | |
| AAEL000116 | | 0.2915 | 2.5533 | 0.4523 | 0.9996 | |
| AAEL016967 | | -0.3379 | 5.4379 | 0.4524 | 0.9996 | |
| AAEL013410 | | -0.2023 | 4.6566 | 0.4527 | 0.9996 | |
| AAEL014646 | | 0.2793 | 2.9738 | 0.4527 | 0.9996 | Ubiquinone biosynthesis protein COQ4 homolog, mitochondrial Precursor (Coenzyme Q biosynthesis protein 4 homolog) [Source:VB Con |
| AAEL001852 | | 0.1935 | 6.9375 | 0.4527 | 0.9996 | |
| AAEL018248 | | 0.8853 | 0.5941 | 0.4528 | 0.9996 | |
| AAEL011784 | | -0.1707 | 4.2601 | 0.4529 | 0.9996 | alpha(1,3)fucosyltransferase [Source:VB Community Annotation] |
| AAEL006193 | Med4 | 0.2515 | 5.2895 | 0.4530 | 0.9996 | Mediator of RNA polymerase II transcription subunit 4 (Med4) [Source:VB Community Annotation] |
| AAEL003581 | | 0.5934 | 5.6041 | 0.4533 | 0.9996 | Amidophosphoribosyltransferase [Source:UniProtKB/TrEMBL;Acc:Q17F21] |
| AAEL008161 | | -0.3411 | 1.8389 | 0.4533 | 0.9996 | abnormal oocyte, putative [Source:VB Community Annotation] |

| | | | | | | |
|---|---|---|---|---|---|---|
| AAEL009062 | | -1.4881 | 2.3486 | 0.4533 | 0.9996 | |
| AAEL019821 | NA | -0.2832 | 3.5978 | 0.4533 | 0.9996 | NA |
| AAEL017975 | HSP70Ba | -0.2733 | 3.0559 | 0.4533 | 0.9996 | heat shock protein HSP70 [Source:VB Community Annotation] |
| AAEL006787 | | 0.3876 | 2.5693 | 0.4534 | 0.9996 | |
| AAEL013216 | | -1.2171 | 0.3397 | 0.4536 | 0.9996 | |
| AAEL011157 | | -0.1982 | 7.4219 | 0.4537 | 0.9996 | NADH dehydrogenase, putative [Source:VB Community Annotation] |
| AAEL004950 | | -0.6312 | 0.8858 | 0.4539 | 0.9996 | zinc finger protein [Source:VB Community Annotation] |
| AAEL006501 | | 0.2676 | 3.2362 | 0.4539 | 0.9996 | DNA replication complex GINS protein SLD5 [Source:UniProtKB/TrEMBL;Acc:Q175W9] |
| AAEL013795 | | -0.2158 | 4.2040 | 0.4539 | 0.9996 | spliceosome associated protein [Source:VB Community Annotation] |
| AAEL006121 | | 0.6019 | 1.9040 | 0.4540 | 0.9996 | trypsin, putative [Source:VB Community Annotation] |
| AAEL000098 | | -0.9795 | -1.1539 | 0.4541 | 0.9996 | |
| AAEL008342 | | 0.3566 | 3.7513 | 0.4541 | 0.9996 | monocarboxylate transporter [Source:VB Community Annotation] |
| AAEL013335 | | -0.7134 | 0.6125 | 0.4542 | 0.9996 | |
| AAEL013793 | | -0.6329 | 1.4684 | 0.4543 | 0.9996 | |
| AAEL001316 | | 0.2686 | 6.0900 | 0.4544 | 0.9996 | |
| AAEL008556 | | -0.4828 | 3.0625 | 0.4544 | 0.9996 | prp4 [Source:VB Community Annotation] |
| AAEL013049 | | -0.3275 | 5.0065 | 0.4545 | 0.9996 | nitrilase, putative [Source:VB Community Annotation] |
| AAEL001216 | | 0.2211 | 5.3107 | 0.4545 | 0.9996 | DEAD box ATP-dependent RNA helicase [Source:VB Community Annotation] |
| AAEL028915 | NA | 1.3681 | -0.3708 | 0.4545 | 0.9996 | NA |
| AAEL003206 | | 0.2855 | 6.3811 | 0.4546 | 0.9996 | glutaredoxin, grx [Source:VB Community Annotation] |
| AAEL005060 | CLIPB44 | 0.7411 | -0.6303 | 0.4546 | 0.9996 | Clip-Domain Serine Protease family B. [Source:VB Community Annotation] |
| AAEL026522 | NA | -0.3625 | 2.9253 | 0.4547 | 0.9996 | NA |
| AAEL014978 | | -0.5162 | 2.6229 | 0.4548 | 0.9996 | adult cuticle protein, putative [Source:VB Community Annotation] |
| AAEL007823 | | 0.6982 | 2.6949 | 0.4548 | 0.9996 | PIWI [Source:VB Community Annotation] |
| AAEL004420 | | 0.3677 | 2.4725 | 0.4550 | 0.9996 | |
| AAEL008849 | | 0.4394 | 3.0745 | 0.4550 | 0.9996 | selenophosphate synthase [Source:VB Community Annotation] |
| AAEL001399 | | 0.5018 | 2.0494 | 0.4551 | 0.9996 | |
| AAEL007293 | | 0.3071 | 8.8434 | 0.4551 | 0.9996 | cAMP-dependent protein kinase catalytic subunit [Source:VB Community Annotation] |
| AAEL010502 | | 0.3786 | 3.8499 | 0.4551 | 0.9996 | transcriptional regulator ATRX (X-linked helicase II) [Source:VB Community Annotation] |
| AAEL012442 | | 0.3296 | 3.5027 | 0.4553 | 0.9996 | |
| AAEL002355 | | 0.7633 | 0.2669 | 0.4553 | 0.9996 | |
| AAEL011707 | | 0.2200 | 2.3195 | 0.4555 | 0.9996 | |
| AAEL008899 | | 0.2992 | 4.5291 | 0.4555 | 0.9996 | |
| AAEL004305 | | -0.1798 | 5.1757 | 0.4555 | 0.9996 | |
| AAEL022658 | NA | -0.6712 | 4.4948 | 0.4555 | 0.9996 | NA |
| AAEL022970 | NA | 0.1947 | 3.9237 | 0.4556 | 0.9996 | NA |
| AAEL001879 | | -0.2660 | 2.9430 | 0.4556 | 0.9996 | |
| AAEL024369 | NA | 0.8412 | -2.4221 | 0.4559 | 0.9996 | NA |
| AAEL015557 | | -0.2755 | 3.0815 | 0.4560 | 0.9996 | alpha methylacyl-coa racemase [Source:VB Community Annotation] |
| AAEL005132 | | 0.3210 | 1.8412 | 0.4560 | 0.9996 | |

| | | | | | | |
|---|---|---|---|---|---|---|
| AAEL027052 | NA | -0.6980 | 10.4102 | 0.4561 | 0.9996 | NA |
| AAEL006820 | | 0.6761 | 7.0360 | 0.4562 | 0.9996 | lipid storage droplets surface binding protein 2 (lsd2) [Source:VB Community Annotation] |
| AAEL001341 | | 0.2339 | 5.0629 | 0.4563 | 0.9996 | |
| AAEL000246 | GPRSTN | -1.1192 | -0.6087 | 0.4565 | 0.9996 | GPCR Frizzled/Smoothened Family [Source:VB Community Annotation] |
| AAEL004507 | | -0.2673 | 3.9067 | 0.4565 | 0.9996 | valacyclovir hydrolase [Source:VB Community Annotation] |
| AAEL000855 | | -0.3460 | 2.9391 | 0.4565 | 0.9996 | mannosyltransferase 1, [Source:VB Community Annotation] |
| AAEL015603 | | -0.1798 | 5.6098 | 0.4565 | 0.9996 | ceramide kinase [Source:VB Community Annotation] |
| AAEL002069 | | 0.2778 | 3.8177 | 0.4565 | 0.9996 | malonyl coa-acyl carrier protein transacylase [Source:VB Community Annotation] |
| AAEL015304 | | -0.3420 | 6.5690 | 0.4566 | 0.9996 | |
| AAEL021301 | NA | -0.5671 | 4.5799 | 0.4567 | 0.9996 | NA |
| AAEL009091 | | 0.8703 | -2.2028 | 0.4568 | 0.9996 | |
| AAEL020450 | NA | 0.2621 | 4.8941 | 0.4568 | 0.9996 | NA |
| AAEL009156 | | 0.2646 | 4.3164 | 0.4568 | 0.9996 | tRNA (guanine-N(7)-)-methyltransferase (EC 2.1.1.33)(tRNA(m7G46)-methyltransferase) [Source:VB Community Annotation] |
| AAEL008784 | | -1.0300 | 2.4462 | 0.4569 | 0.9996 | serine-type enodpeptidase, [Source:VB Community Annotation] |
| AAEL000668 | | -0.5955 | 6.5132 | 0.4569 | 0.9996 | |
| AAEL005149 | | 0.2684 | 5.5493 | 0.4569 | 0.9996 | liprin-beta1, putative [Source:VB Community Annotation] |
| AAEL012756 | | -0.7360 | 5.4591 | 0.4571 | 0.9996 | |
| AAEL020057 | NA | -0.1899 | 4.2049 | 0.4571 | 0.9996 | NA |
| AAEL004682 | | -0.1717 | 6.5308 | 0.4571 | 0.9996 | |
| AAEL027985 | NA | -0.7202 | 3.6521 | 0.4573 | 0.9996 | NA |
| AAEL002385 | CCEAE3B | 0.4637 | 3.3977 | 0.4573 | 0.9996 | Carboxy/choline esterase Alpha Esterase [Source:VB Community Annotation] |
| AAEL005976 | | -0.2306 | 6.0573 | 0.4574 | 0.9996 | adenine phosphoribosyltransferase, putative [Source:VB Community Annotation] |
| AAEL013904 | | 0.4060 | 6.0089 | 0.4574 | 0.9996 | 3-hydroxyisobutyrate dehydrogenase [Source:UniProtKB/TrEMBL;Acc:Q16HU0] |
| AAEL020446 | NA | 0.2895 | 3.4398 | 0.4575 | 0.9996 | NA |
| AAEL023068 | NA | -0.5057 | 2.6938 | 0.4575 | 0.9996 | NA |
| AAEL003893 | | 0.7083 | 0.3045 | 0.4576 | 0.9996 | DNA repair protein xp-c / rad4 [Source:VB Community Annotation] |
| AAEL004690 | | -0.2888 | 3.5440 | 0.4576 | 0.9996 | |
| AAEL001289 | | -0.9651 | 2.4085 | 0.4577 | 0.9996 | permease, putative [Source:VB Community Annotation] |
| AAEL004493 | | -0.3055 | 6.1432 | 0.4578 | 0.9996 | ribosome biogenesis protein tsr1 (20S rRNA accumulation protein 1) [Source:VB Community Annotation] |
| AAEL011325 | AKHR | -0.3973 | 5.4483 | 0.4579 | 0.9996 | gonadotropin-releasing hormone receptor [Source:VB Community Annotation] |
| AAEL020344 | NA | -0.4925 | 5.0305 | 0.4579 | 0.9996 | NA |
| AAEL024495 | NA | -0.8998 | -2.7842 | 0.4580 | 0.9996 | NA |
| AAEL021461 | NA | -0.7964 | -0.3059 | 0.4580 | 0.9996 | NA |
| AAEL009240 | | 0.2824 | 3.9441 | 0.4581 | 0.9996 | neurotactin [Source:VB Community Annotation] |
| AAEL000244 | | 0.2986 | 3.1768 | 0.4582 | 0.9996 | alpha(1,3)fucosyltransferase [Source:VB Community Annotation] |
| AAEL000292 | | 0.3854 | 1.9520 | 0.4582 | 0.9996 | |
| AAEL001605 | | 0.3105 | 5.3659 | 0.4582 | 0.9996 | microtubule binding protein, putative [Source:VB Community Annotation] |
| AAEL020676 | NA | -0.6884 | 0.2018 | 0.4582 | 0.9996 | NA |
| AAEL009400 | | -0.4785 | 3.4988 | 0.4582 | 0.9996 | ccr4-associated factor [Source:VB Community Annotation] |
| AAEL017331 | | -0.2281 | 4.1647 | 0.4583 | 0.9996 | |

| | | | | | | |
|---|---|---|---|---|---|---|
| AAEL000215 | | 0.2801 | 3.7954 | 0.4583 | 0.9996 | ribonuclease P, 29kD-subunit, putative [Source:VB Community Annotation] |
| AAEL000667 | | -0.5079 | 5.1400 | 0.4584 | 0.9996 | alpha-amylase [Source:VB Community Annotation] |
| AAEL007046 | | 0.7119 | 3.0438 | 0.4584 | 0.9996 | mitochondrial brown fat uncoupling protein [Source:VB Community Annotation] |
| AAEL000834 | | -0.5575 | 5.8791 | 0.4584 | 0.9996 | dimethylaniline monooxygenase [Source:VB Community Annotation] |
| AAEL022562 | NA | 0.6271 | 0.6255 | 0.4585 | 0.9996 | NA |
| AAEL006216 | | -0.7479 | 1.8914 | 0.4586 | 0.9996 | |
| AAEL022849 | NA | -0.9895 | 1.0819 | 0.4586 | 0.9996 | NA |
| AAEL025049 | NA | -0.6343 | 8.5019 | 0.4586 | 0.9996 | NA |
| AAEL020407 | NA | 1.1611 | 0.1788 | 0.4586 | 0.9996 | NA |
| AAEL013823 | | -0.8682 | 7.3553 | 0.4589 | 0.9996 | calcium/calmodulin dependent protein kinase ii [Source:VB Community Annotation] |
| AAEL000297 | mRpS25 | -0.2610 | 5.5408 | 0.4590 | 0.9996 | mitochondrial ribosomal protein S25 [Source:VB Community Annotation] |
| AAEL000893 | | 0.2729 | 3.5608 | 0.4590 | 0.9996 | sex-determining region y protein, sry [Source:VB Community Annotation] |
| AAEL011527 | | -0.2897 | 6.6556 | 0.4591 | 0.9996 | eukaryotic translation initiation factor [Source:VB Community Annotation] |
| AAEL006264 | | -0.2146 | 5.7253 | 0.4592 | 0.9996 | glucose transporter (sugar transporter [Source:VB Community Annotation] |
| AAEL000199 | | 0.2047 | 4.1431 | 0.4592 | 0.9996 | arginyl-tRNA synthetase [Source:VB Community Annotation] |
| AAEL006386 | mRpL39 | 0.2003 | 4.8705 | 0.4593 | 0.9996 | mitochondrial 39S ribosomal protein L39 [Source:VB Community Annotation] |
| AAEL003606 | | 0.6072 | 6.9660 | 0.4593 | 0.9996 | purine biosynthesis protein 6, pur6 [Source:VB Community Annotation] |
| AAEL022046 | NA | -0.4654 | 4.4822 | 0.4594 | 0.9996 | NA |
| AAEL019986 | NA | -0.2875 | 5.7333 | 0.4595 | 0.9996 | NA |
| AAEL010760 | | 0.2932 | 4.0442 | 0.4595 | 0.9996 | |
| AAEL019850 | NA | 0.9985 | 1.7720 | 0.4596 | 0.9996 | NA |
| AAEL022892 | NA | -0.7899 | 2.0627 | 0.4596 | 0.9996 | NA |
| AAEL008408 | | 0.1885 | 5.1552 | 0.4596 | 0.9996 | |
| AAEL004897 | | 1.3462 | 1.0722 | 0.4599 | 0.9996 | brain chitinase and chia [Source:VB Community Annotation] |
| AAEL013119 | | -0.2496 | 5.4933 | 0.4601 | 0.9996 | charged multivesicular body protein [Source:VB Community Annotation] |
| AAEL017123 | Or113 | 0.4253 | 0.8622 | 0.4602 | 0.9996 | Odorant receptor [Source:UniProtKB/TrEMBL;Acc:J9E9D8] |
| AAEL009858 | SIFa | 0.4787 | 7.4538 | 0.4602 | 0.9996 | SIFamide [Source:VB Community Annotation] |
| AAEL008970 | | -1.0297 | -0.3610 | 0.4603 | 0.9996 | |
| AAEL021641 | NA | -0.2081 | 5.4531 | 0.4603 | 0.9996 | NA |
| AAEL019832 | NA | 0.3404 | 4.4291 | 0.4604 | 0.9996 | NA |
| AAEL021256 | NA | 0.2727 | 4.1670 | 0.4605 | 0.9996 | NA |
| AAEL002390 | | 0.4102 | 3.5995 | 0.4607 | 0.9996 | zinc finger protein [Source:VB Community Annotation] |
| AAEL007310 | | 0.2220 | 3.4701 | 0.4607 | 0.9996 | |
| AAEL017702 | U6 | 0.6307 | 0.3148 | 0.4607 | 0.9996 | U6 spliceosomal RNA [Source:RFAM;Acc:RF00026] |
| AAEL013872 | | 0.3494 | 3.5774 | 0.4608 | 0.9996 | |
| AAEL003432 | | 0.3492 | 3.9542 | 0.4609 | 0.9996 | |
| AAEL019432 | NA | -0.3941 | 2.0899 | 0.4609 | 0.9996 | NA |
| AAEL010349 | | 0.4284 | 2.2299 | 0.4610 | 0.9996 | tRNA (guanine-N2-)-methyltransferase, putative [Source:VB Community Annotation] |
| AAEL003896 | | -0.9447 | 2.1873 | 0.4610 | 0.9996 | serine/threonine protein kinase [Source:VB Community Annotation] |
| AAEL019553 | NA | -0.8423 | 4.5147 | 0.4612 | 0.9996 | NA |

| | | | | | | |
|---|---|---|---|---|---|---|
| AAEL002599 | | 0.3807 | 3.3773 | 0.4615 | 0.9996 | |
| AAEL026355 | NA | -0.2619 | 5.0297 | 0.4615 | 0.9996 | NA |
| AAEL003839 | | -0.9784 | 1.3835 | 0.4616 | 0.9996 | |
| AAEL027340 | NA | 0.3910 | 2.8814 | 0.4616 | 0.9996 | NA |
| AAEL019578 | NA | -1.0337 | 1.5495 | 0.4616 | 0.9996 | NA |
| AAEL002462 | | 0.2530 | 2.5900 | 0.4618 | 0.9996 | |
| AAEL012457 | | 0.5320 | 2.8094 | 0.4620 | 0.9996 | alcohol dehydrogenase [Source:VB Community Annotation] |
| AAEL002503 | | 0.8115 | 3.8050 | 0.4620 | 0.9996 | yippee protein [Source:VB Community Annotation] |
| AAEL001306 | | -0.3799 | 10.8720 | 0.4620 | 0.9996 | |
| AAEL020314 | NA | 0.2524 | 5.4606 | 0.4621 | 0.9996 | NA |
| AAEL004065 | | -0.2618 | 6.9365 | 0.4623 | 0.9996 | |
| AAEL004386 | pxt | -1.3301 | -1.1134 | 0.4624 | 0.9996 | Chorion peroxidase [Source:UniProtKB/Swiss-Prot;Acc:P82600] |
| AAEL026287 | NA | -1.2854 | -0.9877 | 0.4624 | 0.9996 | NA |
| AAEL010590 | | -0.3195 | 4.4190 | 0.4625 | 0.9996 | aldose-1-epimerase [Source:VB Community Annotation] |
| AAEL003477 | | -0.2524 | 4.4088 | 0.4625 | 0.9996 | peptidyl-prolyl cis-trans isomerase h, ppih [Source:VB Community Annotation] |
| AAEL001261 | | -0.4385 | 6.1060 | 0.4625 | 0.9996 | |
| AAEL005075 | | 0.2593 | 3.9759 | 0.4628 | 0.9996 | ecsit (evolutionarily conserved signaling intermediate in toll pathways) [Source:VB Community Annotation] |
| AAEL006688 | | -0.2141 | 5.7247 | 0.4628 | 0.9996 | |
| AAEL023902 | NA | -0.7752 | 6.1516 | 0.4628 | 0.9996 | NA |
| AAEL013349 | | -0.6601 | -0.7194 | 0.4630 | 0.9996 | lethal(2)essential for life protein, l2efl [Source:VB Community Annotation] |
| AAEL000436 | | 0.3852 | 4.5074 | 0.4631 | 0.9996 | |
| AAEL011180 | | 0.7371 | 3.3959 | 0.4632 | 0.9996 | |
| AAEL003990 | | 0.4184 | 3.5095 | 0.4632 | 0.9996 | myeloid leukemia factor, [Source:VB Community Annotation] |
| AAEL001010 | | -1.3784 | 0.6245 | 0.4632 | 0.9996 | defective proboscis extension response, putative [Source:VB Community Annotation] |
| AAEL023798 | NA | -0.4690 | 4.3069 | 0.4633 | 0.9996 | NA |
| AAEL018307 | | -0.5578 | 0.6226 | 0.4634 | 0.9996 | |
| AAEL006674 | CLIPB29 | -0.7148 | 7.6059 | 0.4634 | 0.9996 | Clip-Domain Serine Protease family B. [Source:VB Community Annotation] |
| AAEL000080 | | 0.7514 | 4.6617 | 0.4634 | 0.9996 | phosphoenolpyruvate carboxykinase [Source:VB Community Annotation] |
| AAEL020203 | NA | 0.2534 | 5.6648 | 0.4637 | 0.9996 | NA |
| AAEL017048 | GPRGHP4 | -0.7722 | 0.4249 | 0.4637 | 0.9996 | GPCR Growth Hormone Releasing Hormone Family [Source:VB Community Annotation] |
| AAEL002050 | | 0.4902 | 2.0978 | 0.4637 | 0.9996 | Nitric oxide synthase-interacting protein homolog [Source:UniProtKB/TrEMBL;Acc:Q17JB3] |
| AAEL007787 | | 0.2715 | 6.2487 | 0.4640 | 0.9996 | 5-aminolevulinic acid synthase [Source:VB Community Annotation] |
| AAEL011958 | | -0.2335 | 4.0776 | 0.4641 | 0.9996 | |
| AAEL018352 | | -0.8480 | 4.8207 | 0.4641 | 0.9996 | |
| AAEL011209 | | 0.1473 | 6.2368 | 0.4642 | 0.9996 | bat5 hla-b-associated transcript [Source:VB Community Annotation] |
| AAEL001100 | | 0.3871 | 7.2858 | 0.4642 | 0.9996 | phosphoserine phosphatase [Source:VB Community Annotation] |
| AAEL002394 | | -0.7271 | -0.6094 | 0.4643 | 0.9996 | |
| AAEL001318 | | -0.5771 | 0.8663 | 0.4644 | 0.9996 | CRAL/TRIO domain-containing protein [Source:VB Community Annotation] |
| AAEL009505 | | -0.2632 | 2.9865 | 0.4645 | 0.9996 | |
| AAEL000028 | CLIPB34 | -0.3647 | 10.0864 | 0.4645 | 0.9996 | Clip-Domain Serine Protease family B. [Source:VB Community Annotation] |

| | | | | | | |
|---|---|---|---|---|---|---|
| AAEL021307 | NA | -0.2206 | 5.7512 | 0.4645 | 0.9996 | NA |
| AAEL011822 | | -0.6090 | 1.1053 | 0.4647 | 0.9996 | gamma glutamyl transpeptidases [Source:VB Community Annotation] |
| AAEL014039 | | -0.2583 | 10.5052 | 0.4648 | 0.9996 | |
| AAEL026159 | NA | 0.8505 | -0.6141 | 0.4649 | 0.9996 | NA |
| AAEL001159 | | 0.2391 | 5.9900 | 0.4649 | 0.9996 | ribonuclease t2 [Source:VB Community Annotation] |
| AAEL011113 | | -0.2197 | 5.6198 | 0.4650 | 0.9996 | |
| AAEL012513 | | 0.3317 | 6.9299 | 0.4651 | 0.9996 | calcium-binding protein E63-1 [Source:VB Community Annotation] |
| AAEL019737 | NA | 0.3537 | 6.2787 | 0.4651 | 0.9996 | NA |
| AAEL002159 | | 0.2750 | 2.3464 | 0.4653 | 0.9996 | |
| AAEL006920 | LRIM20 | -0.4575 | 3.4322 | 0.4653 | 0.9996 | leucine-rich immune protein (Coil-less) [Source:VB Community Annotation] |
| AAEL012466 | | 0.2920 | 5.5825 | 0.4655 | 0.9996 | integrin beta subunit [Source:VB Community Annotation] |
| AAEL021583 | NA | 0.3214 | 4.6179 | 0.4655 | 0.9996 | NA |
| AAEL001797 | | -0.6965 | 1.1735 | 0.4655 | 0.9996 | endopeptidase clp, putative [Source:VB Community Annotation] |
| AAEL013965 | | 0.5387 | 0.3084 | 0.4656 | 0.9996 | |
| AAEL018914 | tRNA-Glu | 0.7625 | -1.4589 | 0.4656 | 0.9996 | |
| AAEL019908 | NA | -1.2066 | -1.2199 | 0.4657 | 0.9996 | NA |
| AAEL019638 | NA | 1.0978 | 0.1371 | 0.4658 | 0.9996 | NA |
| AAEL009760 | | -1.0125 | 3.1412 | 0.4658 | 0.9996 | Niemann-Pick Type C-2, putative [Source:VB Community Annotation] |
| AAEL007772 | | -1.1015 | -0.4575 | 0.4659 | 0.9996 | |
| AAEL025140 | NA | 0.9008 | 1.1395 | 0.4661 | 0.9996 | NA |
| AAEL025122 | NA | -0.1661 | 5.0422 | 0.4662 | 0.9996 | NA |
| AAEL000010 | Rpl36-1 | -0.2105 | 11.0117 | 0.4664 | 0.9996 | 60S ribosomal protein L36 [Source:UniProtKB/TrEMBL;Acc:Q1HR17] |
| AAEL007702 | | 0.2626 | 6.2134 | 0.4664 | 0.9996 | chaperonin [Source:VB Community Annotation] |
| AAEL004074 | | -0.2665 | 4.1799 | 0.4665 | 0.9996 | |
| AAEL018217 | | 0.4262 | 3.1774 | 0.4665 | 0.9996 | |
| AAEL017012 | | -0.2087 | 8.9817 | 0.4666 | 0.9996 | |
| AAEL001985 | | 0.2997 | 8.2290 | 0.4667 | 0.9996 | protein serine/threonine kinase, putative [Source:VB Community Annotation] |
| AAEL002808 | | -0.2016 | 4.7007 | 0.4667 | 0.9996 | diphthine synthase [Source:VB Community Annotation] |
| AAEL012079 | | 0.5145 | 3.2550 | 0.4667 | 0.9996 | |
| AAEL027462 | NA | 0.7324 | -0.1482 | 0.4668 | 0.9996 | NA |
| AAEL019889 | NA | 0.2565 | 2.3570 | 0.4668 | 0.9996 | NA |
| AAEL004244 | | -0.3832 | 1.3755 | 0.4670 | 0.9996 | |
| AAEL021556 | NA | -0.4919 | 3.9628 | 0.4672 | 0.9996 | NA |
| AAEL003912 | | 0.2181 | 4.9857 | 0.4675 | 0.9996 | trafficking protein particle complex subunit 6b [Source:VB Community Annotation] |
| AAEL003433 | | -0.2227 | 3.5751 | 0.4677 | 0.9996 | copper-transporting ATPase 1, 2 (copper pump 1, 2) [Source:VB Community Annotation] |
| AAEL004124 | | 0.2227 | 3.5910 | 0.4677 | 0.9996 | mitochondrial glutamate carrier, putative [Source:VB Community Annotation] |
| AAEL011659 | | -0.2799 | 3.7850 | 0.4680 | 0.9996 | |
| AAEL014070 | | 0.2830 | 3.1541 | 0.4681 | 0.9996 | vacuolar protein sorting [Source:VB Community Annotation] |
| AAEL009589 | | 0.2808 | 2.7971 | 0.4681 | 0.9996 | |
| AAEL012507 | | 0.1692 | 5.0471 | 0.4682 | 0.9996 | prefoldin, subunit, putative [Source:VB Community Annotation] |

| | | | | | | |
|---|---|---|---|---|---|---|
| AAEL000578 | | -0.5345 | 0.3223 | 0.4683 | 0.9996 | |
| AAEL019517 | NA | 0.3946 | 2.1803 | 0.4684 | 0.9996 | NA |
| AAEL001092 | | -0.2474 | 7.0765 | 0.4685 | 0.9996 | udp-glucose pyrophosphatase [Source:VB Community Annotation] |
| AAEL010543 | | -0.3014 | 4.1723 | 0.4688 | 0.9996 | |
| AAEL005580 | | 0.5281 | 2.9726 | 0.4691 | 0.9996 | |
| AAEL008773 | | -0.3541 | 4.4649 | 0.4691 | 0.9996 | laminin A chain, putative [Source:VB Community Annotation] |
| AAEL002036 | | -0.6254 | 0.7615 | 0.4692 | 0.9996 | |
| AAEL002450 | | 0.2202 | 4.1444 | 0.4694 | 0.9996 | |
| AAEL013520 | | 1.5103 | 0.6452 | 0.4694 | 0.9996 | pupal cuticle protein, putative [Source:VB Community Annotation] |
| AAEL003165 | | 0.3665 | 4.5318 | 0.4694 | 0.9996 | low molecular weight protein-tyrosine-phosphatase [Source:VB Community Annotation] |
| AAEL014413 | CYP304C1 | 0.2487 | 5.5362 | 0.4695 | 0.9996 | cytochrome P450 [Source:VB Community Annotation] |
| AAEL026769 | NA | -0.3029 | 5.4901 | 0.4696 | 0.9996 | NA |
| AAEL005123 | | 0.3503 | 1.9786 | 0.4696 | 0.9996 | Carboxylic ester hydrolase [Source:UniProtKB/TrEMBL;Acc:A0A1S4F9L9] |
| AAEL024659 | NA | -0.7768 | 0.5726 | 0.4697 | 0.9996 | NA |
| AAEL006236 | | -0.3491 | 2.6957 | 0.4697 | 0.9996 | dtdp-glucose 4-6-dehydratase [Source:VB Community Annotation] |
| AAEL026347 | NA | -0.4352 | 2.4524 | 0.4698 | 0.9996 | NA |
| AAEL006055 | | -1.2004 | -0.3948 | 0.4700 | 0.9996 | potassium channel interacting protein [Source:VB Community Annotation] |
| AAEL003013 | | 0.2339 | 5.0662 | 0.4700 | 0.9996 | dual specificity mitogen-activated protein kinase kinase 4 MAPKK4 [Source:VB Community Annotation] |
| AAEL001132 | | -0.5190 | 3.1877 | 0.4700 | 0.9996 | |
| AAEL027273 | NA | -0.7067 | 5.0102 | 0.4705 | 0.9996 | NA |
| AAEL023265 | NA | -0.2957 | 3.5929 | 0.4705 | 0.9996 | NA |
| AAEL023151 | NA | -0.2524 | 5.4855 | 0.4705 | 0.9996 | NA |
| AAEL014207 | | -0.3473 | 1.2680 | 0.4705 | 0.9996 | sugar transporter [Source:VB Community Annotation] |
| AAEL004064 | | -0.2790 | 3.0007 | 0.4705 | 0.9996 | meiotic checkpoint regulator cut4 [Source:VB Community Annotation] |
| AAEL013432 | | -0.3710 | 3.7120 | 0.4705 | 0.9996 | serine protease, putative [Source:VB Community Annotation] |
| AAEL017374 | GPRMTH3 | -0.6726 | 5.2247 | 0.4707 | 0.9996 | GPCR Methuselah Family [Source:VB Community Annotation] |
| AAEL009653 | RpS30 | 0.2459 | 11.7680 | 0.4707 | 0.9996 | 40S ribosomal protein S30 [Source:UniProtKB/TrEMBL;Acc:Q0IEK2] |
| AAEL004657 | | 0.2683 | 7.2917 | 0.4707 | 0.9996 | |
| AAEL024086 | NA | -0.4081 | 3.7974 | 0.4709 | 0.9996 | NA |
| AAEL013652 | | 0.2870 | 4.9319 | 0.4709 | 0.9996 | oxidoreductase [Source:VB Community Annotation] |
| AAEL009593 | | 0.3720 | 4.9738 | 0.4709 | 0.9996 | |
| AAEL017251 | AGO2 | 0.2991 | 4.1617 | 0.4710 | 0.9996 | |
| AAEL014526 | | -0.2951 | 7.0436 | 0.4710 | 0.9996 | sideroflexin 1,2,3 [Source:VB Community Annotation] |
| AAEL024330 | NA | 0.8553 | -0.5019 | 0.4710 | 0.9996 | NA |
| AAEL001984 | | -0.2387 | 3.0696 | 0.4710 | 0.9996 | |
| AAEL008461 | | 0.2504 | 5.8336 | 0.4711 | 0.9996 | surfeit locus protein [Source:VB Community Annotation] |
| AAEL005959 | | -0.3336 | 3.8376 | 0.4711 | 0.9996 | phospholipase b, plb1 [Source:VB Community Annotation] |
| AAEL022376 | NA | -0.5444 | -0.1951 | 0.4713 | 0.9996 | NA |
| AAEL028148 | NA | 0.3656 | 2.7370 | 0.4714 | 0.9996 | NA |
| AAEL012372 | | 0.3784 | 2.7493 | 0.4717 | 0.9996 | |

| | | | | | | |
|---|---|---|---|---|---|---|
| AAEL001886 | | -0.2927 | 3.1752 | 0.4718 | 0.9996 | zinc finger protein, putative [Source:VB Community Annotation] |
| AAEL012629 | | -0.3150 | 2.8132 | 0.4718 | 0.9996 | deoxyuridine 5'-triphosphate nucleotidohydrolase [Source:VB Community Annotation] |
| AAEL026787 | NA | 0.2816 | 6.2893 | 0.4718 | 0.9996 | NA |
| AAEL006249 | | -0.4093 | 4.1236 | 0.4719 | 0.9996 | poly(a) polymerase cid (pap) (caffein-induced death protein) [Source:VB Community Annotation] |
| AAEL000550 | | 0.2301 | 3.4994 | 0.4719 | 0.9996 | werner helicase interacting protein [Source:VB Community Annotation] |
| AAEL003058 | | -0.4906 | 1.2365 | 0.4719 | 0.9996 | glucosyl/glucuronosyl transferases [Source:VB Community Annotation] |
| AAEL003354 | | 0.2220 | 3.8675 | 0.4720 | 0.9996 | |
| AAEL010453 | | 0.2329 | 5.8220 | 0.4720 | 0.9996 | mago nashi, putative [Source:VB Community Annotation] |
| AAEL002557 | | 0.2140 | 5.5805 | 0.4721 | 0.9996 | cationic amino acid transporter [Source:VB Community Annotation] |
| AAEL013325 | | -0.4037 | 1.2207 | 0.4721 | 0.9996 | |
| AAEL007992 | | -0.6155 | 1.4379 | 0.4722 | 0.9996 | trypsin, putative [Source:VB Community Annotation] |
| AAEL012996 | | 0.2158 | 5.9054 | 0.4722 | 0.9996 | rho guanine dissociation factor [Source:VB Community Annotation] |
| AAEL002291 | | 0.8626 | -1.0635 | 0.4724 | 0.9996 | |
| AAEL013617 | | 0.4664 | 2.1422 | 0.4724 | 0.9996 | chimerin (rho- GTPase-activating protein) [Source:VB Community Annotation] |
| AAEL027444 | NA | -0.1534 | 5.9463 | 0.4724 | 0.9996 | NA |
| AAEL000747 | | -1.0198 | -0.1277 | 0.4726 | 0.9996 | |
| AAEL007700 | | -0.4954 | 0.8211 | 0.4726 | 0.9996 | ERm [Source:VB Community Annotation] |
| AAEL004900 | | 0.2010 | 5.2040 | 0.4726 | 0.9996 | geranylgeranyl pyrophosphate synthase [Source:VB Community Annotation] |
| AAEL007792 | | 1.0982 | -0.0184 | 0.4727 | 0.9996 | XK-related protein [Source:UniProtKB/TrEMBL;Acc:A0A1S4FHP7] |
| AAEL010087 | Prosalpha3 | -0.2054 | 6.6645 | 0.4727 | 0.9996 | 26S proteasome alpha 3 subunit [Source:VB Community Annotation] |
| AAEL008960 | | -0.2980 | 2.8325 | 0.4729 | 0.9996 | Regulator of telomere elongation helicase 1 homolog (EC 3.6.4.12) [Source:VB Community Annotation] |
| AAEL013011 | | 0.2208 | 4.4126 | 0.4730 | 0.9996 | |
| AAEL005931 | | -0.3928 | 7.5696 | 0.4732 | 0.9996 | 6-phosphogluconate dehydrogenase, decarboxylating [Source:UniProtKB/TrEMBL;Acc:Q178E4] |
| AAEL008228 | | -0.2007 | 5.4981 | 0.4733 | 0.9996 | small nuclear ribonucleoprotein, core, putative [Source:VB Community Annotation] |
| AAEL021572 | NA | -1.4505 | -0.5783 | 0.4733 | 0.9996 | NA |
| AAEL007410 | | -0.1981 | 5.9694 | 0.4734 | 0.9996 | Inhibitor-2, putative [Source:VB Community Annotation] |
| AAEL000776 | | -0.8666 | -1.1625 | 0.4735 | 0.9996 | |
| AAEL000703 | | 0.5682 | 5.8251 | 0.4736 | 0.9996 | glycogen phosphorylase [Source:VB Community Annotation] |
| AAEL000438 | | 0.4030 | 6.6083 | 0.4737 | 0.9996 | epoxide hydrolase [Source:VB Community Annotation] |
| AAEL027938 | NA | 0.2413 | 8.7521 | 0.4738 | 0.9996 | NA |
| AAEL013151 | | -0.5093 | 2.6499 | 0.4739 | 0.9996 | orf protein [Source:VB Community Annotation] |
| AAEL002285 | | 0.4533 | 4.5960 | 0.4740 | 0.9996 | zinc finger protein [Source:VB Community Annotation] |
| AAEL004910 | | 0.2309 | 3.7129 | 0.4740 | 0.9996 | |
| AAEL001271 | | -0.3671 | 7.3778 | 0.4741 | 0.9996 | |
| AAEL005014 | | 0.4079 | 3.8259 | 0.4741 | 0.9996 | transient receptor potential channel [Source:VB Community Annotation] |
| AAEL009288 | mRpL48 | 0.2577 | 5.9750 | 0.4742 | 0.9996 | mitochondrial ribosomal protein, L48, putative [Source:VB Community Annotation] |
| AAEL004931 | | -0.3400 | 5.1098 | 0.4742 | 0.9996 | beta-hexosaminidase b [Source:VB Community Annotation] |
| AAEL006750 | | -0.5937 | 1.2283 | 0.4744 | 0.9996 | |
| AAEL024741 | NA | -0.6441 | 0.0890 | 0.4745 | 0.9996 | NA |
| AAEL007171 | | 0.7413 | -1.0969 | 0.4746 | 0.9996 | protein phosphatase 2c [Source:VB Community Annotation] |

| | | | | | | |
|---|---|---|---|---|---|---|
| AAEL027131 | NA | 1.2758 | 2.4908 | 0.4747 | 0.9996 | NA |
| AAEL020016 | NA | -0.8696 | 2.6145 | 0.4748 | 0.9996 | NA |
| AAEL009539 | | 0.2771 | 7.0322 | 0.4748 | 0.9996 | acyl-protein thioesterase 1,2 (lysophospholipase i,ii) [Source:VB Community Annotation] |
| AAEL013048 | | -0.3029 | 1.7214 | 0.4749 | 0.9996 | |
| AAEL006990 | | 0.4057 | 3.9543 | 0.4749 | 0.9996 | |
| AAEL006971 | | 0.6398 | 3.1456 | 0.4751 | 0.9996 | |
| AAEL013558 | | -0.9497 | -1.3373 | 0.4751 | 0.9996 | |
| AAEL025808 | NA | -1.2341 | 1.7455 | 0.4751 | 0.9996 | NA |
| AAEL012423 | | 1.2837 | 2.0437 | 0.4752 | 0.9996 | |
| AAEL005326 | | 0.2892 | 3.7981 | 0.4752 | 0.9996 | |
| AAEL008842 | | 0.4011 | 0.3783 | 0.4752 | 0.9996 | |
| AAEL023644 | NA | 0.2030 | 4.6807 | 0.4752 | 0.9996 | NA |
| AAEL000421 | | 0.2921 | 2.4176 | 0.4755 | 0.9996 | protein farnesyltransferase alpha subunit/rab geranylgeranyl transferase alpha subunit [Source:VB Community Annotation] |
| AAEL024471 | NA | 0.5471 | 3.4657 | 0.4756 | 0.9996 | NA |
| AAEL020535 | NA | -0.1731 | 5.5306 | 0.4757 | 0.9996 | NA |
| AAEL006346 | | 0.1953 | 4.6873 | 0.4758 | 0.9996 | |
| AAEL027478 | NA | -0.3106 | 3.1457 | 0.4760 | 0.9996 | NA |
| AAEL011639 | | -0.3129 | 10.0733 | 0.4762 | 0.9996 | WAP four-disulfide core domain protein 2 precursor, putative [Source:VB Community Annotation] |
| AAEL003664 | | -0.3003 | 3.3234 | 0.4762 | 0.9996 | lupus la ribonucleoprotein [Source:VB Community Annotation] |
| AAEL012019 | | 0.1831 | 5.1246 | 0.4763 | 0.9996 | |
| AAEL011309 | | 0.2730 | 5.1030 | 0.4763 | 0.9996 | orotidine-5'-phosphate decarboxylase, putative [Source:VB Community Annotation] |
| AAEL002232 | | 0.2706 | 4.0119 | 0.4763 | 0.9996 | |
| AAEL002688 | | 0.2967 | 5.5091 | 0.4764 | 0.9996 | glucosyl/glucuronosyl transferases [Source:VB Community Annotation] |
| AAEL018152 | | -0.3063 | 6.2768 | 0.4765 | 0.9996 | |
| AAEL009303 | | -0.2238 | 4.0862 | 0.4766 | 0.9996 | regulator of chromosome condensation [Source:VB Community Annotation] |
| AAEL025882 | NA | -0.2941 | 2.4612 | 0.4767 | 0.9996 | NA |
| AAEL024595 | NA | 0.4100 | 0.6746 | 0.4770 | 0.9996 | NA |
| AAEL007280 | | -0.2492 | 3.2639 | 0.4771 | 0.9996 | |
| AAEL023348 | NA | -0.9787 | 1.6889 | 0.4771 | 0.9996 | NA |
| AAEL020038 | NA | 0.9481 | -1.6388 | 0.4772 | 0.9996 | NA |
| AAEL006247 | | -0.4155 | 3.8672 | 0.4775 | 0.9996 | |
| AAEL022872 | NA | -0.6772 | -0.3628 | 0.4777 | 0.9996 | NA |
| AAEL007414 | | 0.2399 | 3.4914 | 0.4778 | 0.9996 | |
| AAEL002529 | | 0.2635 | 4.6091 | 0.4778 | 0.9996 | |
| AAEL003406 | | 0.9133 | -1.5275 | 0.4778 | 0.9996 | |
| AAEL018319 | | 0.3591 | 2.2830 | 0.4782 | 0.9996 | |
| AAEL017022 | | 0.9691 | 5.0752 | 0.4783 | 0.9996 | |
| AAEL007929 | | -1.0562 | 1.4846 | 0.4783 | 0.9996 | |
| AAEL001501 | | 0.2693 | 5.2781 | 0.4783 | 0.9996 | anamorsin homolog (Fe-S cluster assembly protein DRE2 homolog) [Source:VB Community Annotation] |
| AAEL013624 | | 0.2720 | 2.5819 | 0.4784 | 0.9996 | |

| | | | | | | |
|---|---|---|---|---|---|---|
| AAEL013257 | | 0.4270 | 2.0377 | 0.4786 | 0.9996 | |
| AAEL020033 | NA | -0.6705 | 3.5272 | 0.4788 | 0.9996 | NA |
| AAEL019860 | NA | -0.6139 | 4.9599 | 0.4788 | 0.9996 | NA |
| AAEL019639 | NA | 0.8076 | 3.1478 | 0.4788 | 0.9996 | NA |
| AAEL011301 | | 0.2793 | 3.0293 | 0.4788 | 0.9996 | |
| AAEL024398 | NA | -1.3246 | 0.5082 | 0.4789 | 0.9996 | NA |
| AAEL004440 | | 0.2891 | 6.4976 | 0.4790 | 0.9996 | tubulin-specific chaperone e [Source:VB Community Annotation] |
| AAEL008421 | | -0.3713 | 2.7739 | 0.4792 | 0.9996 | cadherin [Source:VB Community Annotation] |
| AAEL020646 | NA | -0.8171 | 5.2462 | 0.4792 | 0.9996 | NA |
| AAEL005329 | | -0.2644 | 6.8053 | 0.4795 | 0.9996 | |
| AAEL007431 | | 0.2361 | 6.2148 | 0.4798 | 0.9996 | |
| AAEL015450 | | -0.6089 | 7.7510 | 0.4798 | 0.9996 | ribonuclease UK114, putative [Source:VB Community Annotation] |
| AAEL014422 | | 0.3274 | 4.0236 | 0.4799 | 0.9996 | syntrophin [Source:VB Community Annotation] |
| AAEL003416 | | -0.2512 | 4.7919 | 0.4799 | 0.9996 | Sorting nexin [Source:UniProtKB/TrEMBL;Acc:Q17FH9] |
| AAEL026274 | NA | -0.2640 | 4.6584 | 0.4800 | 0.9996 | NA |
| AAEL009481 | | -0.2531 | 4.4032 | 0.4802 | 0.9996 | |
| AAEL000263 | | -0.9465 | -0.3725 | 0.4802 | 0.9996 | Zinc finger protein jing homolog [Source:UniProtKB/Swiss-Prot;Acc:Q17PR1] |
| AAEL002797 | | -0.2790 | 7.7774 | 0.4802 | 0.9996 | |
| AAEL007103 | LRIM15 | -0.6334 | 5.7943 | 0.4803 | 0.9996 | leucine-rich immune protein (TM) [Source:VB Community Annotation] |
| AAEL002646 | | 0.2648 | 3.3762 | 0.4805 | 0.9996 | cytohesin 1, 2, 3, 4 (guanine nucleotide-exchange protein) [Source:VB Community Annotation] |
| AAEL022914 | NA | -0.5628 | 0.1153 | 0.4806 | 0.9996 | NA |
| AAEL014561 | 15a-3 | 1.1913 | -2.5538 | 0.4807 | 0.9996 | Vitelline membrane protein 15a-3 [Source:UniProtKB/Swiss-Prot;Acc:Q16G12] |
| AAEL023022 | NA | -0.2594 | 5.9035 | 0.4807 | 0.9996 | NA |
| AAEL012363 | | 0.8548 | 1.9454 | 0.4808 | 0.9996 | |
| AAEL014057 | | -0.2107 | 5.9335 | 0.4808 | 0.9996 | |
| AAEL011951 | | -0.5956 | -0.5458 | 0.4809 | 0.9996 | elongase, putative [Source:VB Community Annotation] |
| AAEL011004 | | -0.3645 | 5.0543 | 0.4809 | 0.9996 | |
| AAEL000656 | | -0.3835 | 3.3618 | 0.4810 | 0.9996 | pangolin [Source:VB Community Annotation] |
| AAEL017437 | | -0.3173 | 6.3653 | 0.4810 | 0.9996 | |
| AAEL018219 | | -0.4285 | 6.1721 | 0.4810 | 0.9996 | |
| AAEL001690 | | 1.3398 | 0.8824 | 0.4812 | 0.9996 | serine-type enodpeptidase [Source:VB Community Annotation] |
| AAEL010138 | | 0.3832 | 1.6708 | 0.4812 | 0.9996 | |
| AAEL007088 | | -0.4180 | 3.3333 | 0.4813 | 0.9996 | ribitol kinase [Source:VB Community Annotation] |
| AAEL005635 | | 0.3419 | 4.9593 | 0.4814 | 0.9996 | nucleoporin [Source:VB Community Annotation] |
| AAEL025889 | NA | 0.2425 | 5.6303 | 0.4816 | 0.9996 | NA |
| AAEL024866 | NA | -0.8517 | -1.6691 | 0.4816 | 0.9996 | NA |
| AAEL012074 | | 0.2737 | 5.7753 | 0.4818 | 0.9996 | |
| AAEL007451 | | 0.2125 | 5.2181 | 0.4819 | 0.9996 | WD-repeat protein [Source:VB Community Annotation] |
| AAEL007619 | TOLL5A | -0.4041 | 3.7821 | 0.4822 | 0.9996 | Toll-like receptor [Source:VB Community Annotation] |
| AAEL011768 | | -0.6379 | 3.1943 | 0.4825 | 0.9996 | |

| | | | | | | |
|---|---|---|---|---|---|---|
| AAEL012039 | | -0.5028 | 0.9181 | 0.4826 | 0.9996 | zinc finger protein [Source:VB Community Annotation] |
| AAEL006726 | inx4 | 1.0677 | -1.6886 | 0.4826 | 0.9996 | Innexin [Source:UniProtKB/TrEMBL;Acc:Q174Z8] |
| AAEL007692 | | -0.4658 | 1.2195 | 0.4827 | 0.9996 | DNA polymerase alpha subunit B [Source:UniProtKB/TrEMBL;Acc:Q171D3] |
| AAEL027399 | NA | -0.1927 | 5.5779 | 0.4828 | 0.9996 | NA |
| AAEL000079 | | -0.6776 | 6.4796 | 0.4828 | 0.9996 | |
| AAEL000978 | | 0.2440 | 4.9381 | 0.4829 | 0.9996 | |
| AAEL014842 | | -0.2274 | 5.3261 | 0.4830 | 0.9996 | multiple inositol polyphosphate phosphatase [Source:VB Community Annotation] |
| AAEL010855 | | 0.3507 | 0.0784 | 0.4832 | 0.9996 | cdc6 [Source:VB Community Annotation] |
| AAEL019430 | NA | 0.3049 | 5.3267 | 0.4832 | 0.9996 | NA |
| AAEL026487 | NA | 0.3663 | 1.3896 | 0.4833 | 0.9996 | NA |
| AAEL028087 | NA | 0.3385 | 2.7457 | 0.4833 | 0.9996 | NA |
| AAEL008728 | | -0.2810 | 2.8229 | 0.4834 | 0.9996 | DEAD box ATP-dependent RNA helicase [Source:VB Community Annotation] |
| AAEL003163 | | -1.3570 | -0.2647 | 0.4835 | 0.9996 | forkhead protein/ forkhead protein domain [Source:VB Community Annotation] |
| AAEL013441 | TOLL9A | 0.4672 | 2.1324 | 0.4835 | 0.9996 | Toll-like receptor [Source:VB Community Annotation] |
| AAEL022418 | NA | -0.9352 | -1.3971 | 0.4837 | 0.9996 | NA |
| AAEL002416 | | 0.4058 | 5.4751 | 0.4837 | 0.9996 | short-chain dehydrogenase [Source:VB Community Annotation] |
| AAEL004320 | | 1.0475 | 1.2411 | 0.4838 | 0.9996 | WOC protein, putative [Source:VB Community Annotation] |
| AAEL012567 | | 0.3564 | 3.2754 | 0.4839 | 0.9996 | synaptic vesicle protein [Source:VB Community Annotation] |
| AAEL003548 | | 0.3654 | 2.7370 | 0.4842 | 0.9996 | sulphate transporter [Source:VB Community Annotation] |
| AAEL014198 | | -0.2454 | 4.5735 | 0.4842 | 0.9996 | cdp-diacylglycerol--glycerol-3-phosphate 3-phosphatidyltransferase [Source:VB Community Annotation] |
| AAEL004287 | | 0.3226 | 3.7078 | 0.4842 | 0.9996 | fas-associated protein [Source:VB Community Annotation] |
| AAEL004026 | | 0.2285 | 4.1564 | 0.4844 | 0.9996 | |
| AAEL013391 | | -0.3761 | 2.5730 | 0.4845 | 0.9996 | |
| AAEL006285 | | 0.2469 | 3.4801 | 0.4845 | 0.9996 | |
| AAEL010799 | | -1.0836 | 0.0680 | 0.4846 | 0.9996 | |
| AAEL020019 | NA | -0.5388 | 2.2987 | 0.4848 | 0.9996 | NA |
| AAEL002834 | | 0.2558 | 7.6704 | 0.4849 | 0.9996 | myo-inositol-1 phosphate synthase [Source:VB Community Annotation] |
| AAEL000276 | | -0.2802 | 2.8170 | 0.4849 | 0.9996 | |
| AAEL001385 | | 0.1825 | 5.6035 | 0.4850 | 0.9996 | |
| AAEL019905 | NA | 0.2548 | 5.4285 | 0.4851 | 0.9996 | NA |
| AAEL012126 | | 0.4047 | 3.2499 | 0.4852 | 0.9996 | F-box/lrr protein, drome [Source:VB Community Annotation] |
| AAEL001146 | | 0.8703 | -1.7364 | 0.4852 | 0.9996 | n-acetylgalactosaminyltransferase [Source:VB Community Annotation] |
| AAEL009876 | | -0.2307 | 4.9611 | 0.4853 | 0.9996 | |
| AAEL011757 | | 1.0235 | 0.1225 | 0.4854 | 0.9996 | |
| AAEL004000 | TOLL10 | 0.2753 | 2.8899 | 0.4854 | 0.9996 | Toll-like receptor [Source:VB Community Annotation] |
| AAEL010909 | | -0.2478 | 4.2173 | 0.4854 | 0.9996 | |
| AAEL001698 | | 0.1532 | 6.9207 | 0.4855 | 0.9996 | charged multivesicular body protein 4b [Source:VB Community Annotation] |
| AAEL011918 | | 0.5206 | 5.6197 | 0.4857 | 0.9996 | |
| AAEL012851 | | 0.4031 | 4.9593 | 0.4857 | 0.9996 | WD-repeat protein [Source:VB Community Annotation] |
| AAEL003070 | | 0.3416 | 2.5591 | 0.4857 | 0.9996 | |

| | | | | | | |
|---|---|---|---|---|---|---|
| AAEL004175 | RpS17 | 0.2393 | 10.7369 | 0.4861 | 0.9996 | 40S ribosomal protein S17 [Source:UniProtKB/TrEMBL;Acc:Q52UT2] |
| AAEL017226 | | -0.8552 | -0.2459 | 0.4861 | 0.9996 | |
| AAEL012254 | Or8 | -0.5635 | 0.1754 | 0.4862 | 0.9996 | Odorant receptor [Source:UniProtKB/TrEMBL;Acc:Q16MN0] |
| AAEL012421 | | -1.3520 | 1.4470 | 0.4863 | 0.9996 | cadherin [Source:VB Community Annotation] |
| AAEL013723 | | -0.4050 | 9.5138 | 0.4863 | 0.9996 | polypyrimidine tract binding protein [Source:VB Community Annotation] |
| AAEL029014 | NA | 0.1963 | 4.2341 | 0.4864 | 0.9996 | NA |
| AAEL010567 | | -0.7188 | 7.8967 | 0.4866 | 0.9996 | |
| AAEL007441 | | -0.2109 | 6.3541 | 0.4866 | 0.9996 | translocon-associated protein, gamma subunit [Source:VB Community Annotation] |
| AAEL014642 | | -0.5777 | 5.7629 | 0.4867 | 0.9996 | ubiquitin conjugating enzyme, putative [Source:VB Community Annotation] |
| AAEL009932 | | 0.1573 | 5.0158 | 0.4867 | 0.9996 | lethal giant larva homologue [Source:VB Community Annotation] |
| AAEL011279 | | 0.2491 | 3.2740 | 0.4869 | 0.9996 | requim, req/dpf2 [Source:VB Community Annotation] |
| AAEL007850 | | 0.4127 | 2.3970 | 0.4869 | 0.9996 | |
| AAEL025157 | NA | 0.7628 | -0.9687 | 0.4869 | 0.9996 | NA |
| AAEL011465 | | -0.3403 | 4.2950 | 0.4869 | 0.9996 | |
| AAEL010067 | | -1.5374 | -0.1622 | 0.4871 | 0.9996 | |
| AAEL006446 | | -0.4026 | 6.1077 | 0.4871 | 0.9996 | trehalose-6-phosphate synthase [Source:VB Community Annotation] |
| AAEL009137 | CYP6N13 | 0.6619 | -1.0189 | 0.4872 | 0.9996 | cytochrome P450 [Source:VB Community Annotation] |
| AAEL005373 | GPROP12 | 0.4078 | 1.9290 | 0.4872 | 0.9996 | pteropsin [Source:VB Community Annotation] |
| AAEL013561 | | 0.2707 | 3.7289 | 0.4873 | 0.9996 | |
| AAEL003526 | | -0.1770 | 4.2272 | 0.4873 | 0.9996 | |
| AAEL003091 | | -0.3882 | 6.1202 | 0.4874 | 0.9996 | glucosyl/glucuronosyl transferases [Source:VB Community Annotation] |
| AAEL006002 | | -0.1955 | 4.7324 | 0.4876 | 0.9996 | |
| AAEL004460 | | -0.2074 | 7.9123 | 0.4877 | 0.9996 | sulfatase [Source:VB Community Annotation] |
| AAEL027949 | NA | 0.1817 | 5.1442 | 0.4877 | 0.9996 | NA |
| AAEL005577 | | 0.2575 | 1.8793 | 0.4878 | 0.9996 | |
| AAEL006605 | | 0.4741 | 1.8958 | 0.4879 | 0.9996 | juvenile hormone-inducible protein, putative [Source:VB Community Annotation] |
| AAEL002683 | | 0.4447 | 5.2784 | 0.4880 | 0.9996 | aldehyde oxidase [Source:VB Community Annotation] |
| AAEL028222 | NA | 0.7643 | 0.0677 | 0.4882 | 0.9996 | NA |
| AAEL003619 | | 0.3611 | 5.5316 | 0.4883 | 0.9996 | sodium/chloride dependent amino acid transporter [Source:VB Community Annotation] |
| AAEL010844 | | 0.1984 | 4.5272 | 0.4883 | 0.9996 | ubiquitin-conjugating enzyme E2 j2 [Source:VB Community Annotation] |
| AAEL000746 | | -0.2093 | 7.4324 | 0.4885 | 0.9996 | NADP-specific isocitrate dehydrogenase [Source:VB Community Annotation] |
| AAEL008947 | | 0.2455 | 3.4955 | 0.4888 | 0.9996 | |
| AAEL006125 | | 0.2484 | 4.1937 | 0.4890 | 0.9996 | |
| AAEL001317 | | 0.2466 | 5.4216 | 0.4890 | 0.9996 | DEAD box ATP-dependent RNA helicase [Source:VB Community Annotation] |
| AAEL001683 | | -0.6930 | 5.7369 | 0.4892 | 0.9996 | |
| AAEL013211 | | -0.6536 | 4.8540 | 0.4893 | 0.9996 | |
| AAEL003035 | GPROP9 | 0.2597 | 11.0588 | 0.4894 | 0.9996 | short wavelength sensitive opsin [Source:VB Community Annotation] |
| AAEL014527 | | -0.1970 | 5.4955 | 0.4895 | 0.9996 | potassium-dependent sodium-calcium exchanger, putative [Source:VB Community Annotation] |
| AAEL004977 | | 0.2364 | 5.2188 | 0.4896 | 0.9996 | Sec24B protein, putative [Source:VB Community Annotation] |
| AAEL011731 | | 0.2202 | 5.0669 | 0.4896 | 0.9996 | metalloprotease [Source:VB Community Annotation] |

| | | | | | | |
|---|---|---|---|---|---|---|
| AAEL012378 | | 0.2424 | 3.7570 | 0.4900 | 0.9996 | serine-type protease inhibitor [Source:VB Community Annotation] |
| AAEL002796 | | 0.2999 | 3.5532 | 0.4900 | 0.9996 | l-asparaginase i [Source:VB Community Annotation] |
| AAEL000502 | | 0.6041 | 0.1288 | 0.4900 | 0.9996 | NADPH FAD oxidoreductase [Source:VB Community Annotation] |
| AAEL014760 | | -0.2315 | 4.7977 | 0.4901 | 0.9996 | |
| AAEL023321 | NA | -0.9226 | -1.4023 | 0.4902 | 0.9996 | NA |
| AAEL014274 | | -0.5024 | 4.8290 | 0.4902 | 0.9996 | |
| AAEL000260 | | 0.3202 | 2.5472 | 0.4903 | 0.9996 | |
| AAEL020614 | NA | -0.2209 | 5.4954 | 0.4903 | 0.9996 | NA |
| AAEL020849 | NA | -1.2184 | -2.0879 | 0.4905 | 0.9996 | NA |
| AAEL005909 | | 0.3552 | 5.5257 | 0.4906 | 0.9996 | splicing factor SC35, putative [Source:VB Community Annotation] |
| AAEL007295 | | -0.6986 | -0.1452 | 0.4911 | 0.9996 | |
| AAEL003454 | | 0.1553 | 5.4050 | 0.4911 | 0.9996 | phocein protein, putative [Source:VB Community Annotation] |
| AAEL021318 | NA | 0.2468 | 4.3135 | 0.4912 | 0.9996 | NA |
| AAEL005017 | | -0.3025 | 7.7059 | 0.4913 | 0.9996 | |
| AAEL010655 | SCRASP2 | -1.0783 | -0.3634 | 0.4913 | 0.9996 | Class A Scavenger Receptor (SRCR domain) with Serine Protease domain. [Source:VB Community Annotation] |
| AAEL024566 | NA | -0.2165 | 5.6870 | 0.4914 | 0.9996 | NA |
| AAEL000955 | APG3 | 0.2124 | 5.2828 | 0.4914 | 0.9996 | autophagy related gene [Source:VB Community Annotation] |
| AAEL005266 | RpS14 | 0.1613 | 10.8360 | 0.4914 | 0.9996 | 40S ribosomal protein S14 [Source:UniProtKB/Swiss-Prot;Acc:Q1HR24] |
| AAEL010681 | | 0.2666 | 5.4162 | 0.4915 | 0.9996 | sodium/chloride dependent neurotransmitter transporter [Source:VB Community Annotation] |
| AAEL010491 | FKBP12 | 0.2004 | 3.8472 | 0.4916 | 0.9996 | fk506-binding protein [Source:VB Community Annotation] |
| AAEL013144 | | -0.1941 | 8.8481 | 0.4917 | 0.9996 | Eukaryotic translation initiation factor 3 subunit I (eIF3i) [Source:VB Community Annotation] |
| AAEL004402 | | 0.5638 | 0.4948 | 0.4919 | 0.9996 | alpha-l-iduronidase [Source:VB Community Annotation] |
| AAEL006053 | | 0.6231 | 1.2883 | 0.4920 | 0.9996 | |
| AAEL004465 | | 0.4427 | 1.9373 | 0.4921 | 0.9996 | |
| AAEL006437 | | -0.3072 | 2.1242 | 0.4921 | 0.9996 | |
| AAEL005789 | | -0.3224 | 4.5446 | 0.4923 | 0.9996 | |
| AAEL027968 | NA | -0.7996 | 4.3169 | 0.4923 | 0.9996 | NA |
| AAEL005709 | | 0.2230 | 7.4798 | 0.4924 | 0.9996 | n-acetyltransferase separation anxiety [Source:VB Community Annotation] |
| AAEL021099 | NA | 0.3285 | 3.5730 | 0.4924 | 0.9996 | NA |
| AAEL003192 | | 0.8461 | 0.5929 | 0.4924 | 0.9996 | mfs transporter [Source:VB Community Annotation] |
| AAEL013070 | | 0.2949 | 3.0916 | 0.4924 | 0.9996 | |
| AAEL024720 | NA | 0.2608 | 4.0421 | 0.4925 | 0.9996 | NA |
| AAEL019562 | NA | -1.1944 | 2.3165 | 0.4931 | 0.9996 | NA |
| AAEL003597 | | 0.2457 | 3.5638 | 0.4932 | 0.9996 | leucine-rich transmembrane protein [Source:VB Community Annotation] |
| AAEL024921 | NA | -0.1788 | 9.8160 | 0.4932 | 0.9996 | NA |
| AAEL023069 | NA | 0.2704 | 7.3889 | 0.4933 | 0.9996 | NA |
| AAEL022508 | NA | -1.1215 | -1.6510 | 0.4933 | 0.9996 | NA |
| AAEL001050 | | 0.9062 | -0.6162 | 0.4934 | 0.9996 | |
| AAEL019538 | NA | -0.5686 | 6.5076 | 0.4934 | 0.9996 | NA |
| AAEL028092 | NA | -0.6380 | 4.3125 | 0.4934 | 0.9996 | NA |

| | | | | | | |
|---|---|---|---|---|---|---|
| AAEL000873 | | 0.9360 | -2.5570 | 0.4935 | 0.9996 | pickpocket [Source:VB Community Annotation] |
| AAEL007322 | | -1.1184 | 0.8223 | 0.4935 | 0.9996 | phosphatidate phosphatase [Source:VB Community Annotation] |
| AAEL027610 | NA | 0.7257 | 1.1097 | 0.4937 | 0.9996 | NA |
| AAEL019592 | NA | -0.3427 | 2.9867 | 0.4937 | 0.9996 | NA |
| AAEL011551 | | 1.1511 | 1.6389 | 0.4937 | 0.9996 | |
| AAEL000699 | | -0.2932 | 2.1758 | 0.4939 | 0.9996 | |
| AAEL011897 | | 0.3066 | 3.7275 | 0.4940 | 0.9996 | |
| AAEL014932 | | -0.2882 | 9.2763 | 0.4941 | 0.9996 | Transcription factor BTF3 [Source:UniProtKB/TrEMBL;Acc:Q1HRK3] |
| AAEL011486 | | -0.8335 | 1.0703 | 0.4943 | 0.9996 | |
| AAEL026777 | NA | 0.3596 | 1.1713 | 0.4943 | 0.9996 | NA |
| AAEL007892 | | -0.2648 | 4.4710 | 0.4943 | 0.9996 | xaa-pro aminopeptidase [Source:VB Community Annotation] |
| AAEL006515 | | -0.2164 | 2.9909 | 0.4943 | 0.9996 | |
| AAEL027088 | NA | 0.2059 | 5.1731 | 0.4943 | 0.9996 | NA |
| AAEL007885 | | 0.2361 | 4.6234 | 0.4944 | 0.9996 | translation initiation factor-3 (IF3), putative [Source:VB Community Annotation] |
| AAEL002547 | | 0.1970 | 5.2499 | 0.4946 | 0.9996 | Dipeptidase [Source:UniProtKB/TrEMBL;Acc:Q17HT5] |
| AAEL001342 | Or59 | -0.6306 | -0.5149 | 0.4946 | 0.9996 | Odorant receptor [Source:UniProtKB/TrEMBL;Acc:Q17LK1] |
| AAEL017194 | | -0.5514 | 3.9822 | 0.4946 | 0.9996 | |
| AAEL022542 | NA | -0.9666 | -0.7071 | 0.4947 | 0.9996 | NA |
| AAEL025740 | NA | 0.6460 | 2.6857 | 0.4948 | 0.9996 | NA |
| AAEL026707 | NA | 0.6943 | -0.9000 | 0.4950 | 0.9996 | NA |
| AAEL000143 | | -0.2471 | 5.3990 | 0.4952 | 0.9996 | |
| AAEL019884 | NA | -0.4846 | 3.1624 | 0.4953 | 0.9996 | NA |
| AAEL017213 | | 0.1877 | 5.7130 | 0.4953 | 0.9996 | DNA-directed RNA polymerase subunit beta [Source:UniProtKB/TrEMBL;Acc:J9E9C4] |
| AAEL022005 | NA | -0.6324 | 3.7011 | 0.4954 | 0.9996 | NA |
| AAEL028211 | NA | -0.2256 | 3.9053 | 0.4954 | 0.9996 | NA |
| AAEL019817 | NA | 0.3168 | 1.0888 | 0.4955 | 0.9996 | NA |
| AAEL002542 | Tpi | -0.2535 | 7.8210 | 0.4956 | 0.9996 | Triosephosphate isomerase [Source:UniProtKB/TrEMBL;Acc:Q17HW3] |
| AAEL000861 | | 0.8043 | 1.1069 | 0.4957 | 0.9996 | |
| AAEL007796 | CLIPD1 | -0.3496 | 5.0206 | 0.4958 | 0.9996 | Clip-Domain Serine Protease  family D [Source:VB Community Annotation] |
| AAEL002782 | | 0.2473 | 3.0238 | 0.4959 | 0.9996 | |
| AAEL000383 | | 0.3667 | 3.9499 | 0.4960 | 0.9996 | beta-1,3-galactosyltransferase brn [Source:VB Community Annotation] |
| AAEL008952 | | 0.1886 | 4.9647 | 0.4961 | 0.9996 | sentrin/sumo-specific protease [Source:VB Community Annotation] |
| AAEL008827 | | 0.9695 | 2.2169 | 0.4962 | 0.9996 | cell adhesion molecule [Source:VB Community Annotation] |
| AAEL001987 | | 0.7633 | -0.4045 | 0.4966 | 0.9996 | protein serine/threonine kinase, putative [Source:VB Community Annotation] |
| AAEL006538 | | 0.2334 | 6.5510 | 0.4966 | 0.9996 | peroxisomal membrane protein 2, pxmp2 [Source:VB Community Annotation] |
| AAEL007244 | | 0.5665 | 0.7672 | 0.4967 | 0.9996 | zinc finger protein [Source:VB Community Annotation] |
| AAEL023825 | NA | 0.3282 | 2.2313 | 0.4968 | 0.9996 | NA |
| AAEL015347 | | 0.4263 | 1.2280 | 0.4969 | 0.9996 | |
| AAEL018114 | | -0.9166 | 0.1735 | 0.4969 | 0.9996 | |
| AAEL003039 | | -0.2633 | 6.8369 | 0.4971 | 0.9996 | nonsense-mediated mRNA decay protein [Source:VB Community Annotation] |

| | | | | | | |
|---|---|---|---|---|---|---|
| AAEL008574 | | -0.3019 | 5.7195 | 0.4972 | 0.9996 | acyl-CoA oxidase [Source:VB Community Annotation] |
| AAEL000465 | | -0.4715 | 2.2982 | 0.4974 | 0.9996 | |
| AAEL006690 | | 0.5835 | -0.1954 | 0.4975 | 0.9996 | ribbon [Source:VB Community Annotation] |
| AAEL007668 | mRpS28 | -0.2560 | 6.1128 | 0.4976 | 0.9996 | mitochondrial ribosomal protein, S29, putative [Source:VB Community Annotation] |
| AAEL014732 | | 0.8218 | -0.1309 | 0.4976 | 0.9996 | |
| AAEL005029 | | 0.5027 | 2.2170 | 0.4977 | 0.9996 | |
| AAEL002979 | | -0.5845 | 0.1293 | 0.4977 | 0.9996 | |
| AAEL013966 | | -0.2831 | 7.1519 | 0.4978 | 0.9996 | ATP-dependent clp protease ATP-binding subunit clpx [Source:VB Community Annotation] |
| AAEL017305 | Or81 | 0.7684 | -0.3047 | 0.4979 | 0.9996 | Odorant receptor [Source:UniProtKB/TrEMBL;Acc:J9E9I7] |
| AAEL002418 | mRpL27 | -0.1964 | 4.9279 | 0.4980 | 0.9996 | mitochondrial ribosomal protein, L27, putative [Source:VB Community Annotation] |
| AAEL010895 | | 0.3669 | 2.8940 | 0.4981 | 0.9996 | |
| AAEL019680 | NA | 0.2318 | 5.8306 | 0.4981 | 0.9996 | NA |
| AAEL007397 | E75 | -0.4118 | 5.8372 | 0.4981 | 0.9996 | Ecdysone-induced protein 75B isoform A Nuclear receptor [Source:VB Community Annotation] |
| AAEL002116 | | 0.2168 | 4.5523 | 0.4982 | 0.9996 | supercoiling factor, putative [Source:VB Community Annotation] |
| AAEL012381 | | -0.5716 | 0.3995 | 0.4982 | 0.9996 | |
| AAEL013134 | | -0.2897 | 3.6002 | 0.4982 | 0.9996 | DNA-directed RNA polymerase I largest subunit [Source:VB Community Annotation] |
| AAEL009373 | | 0.2525 | 6.2867 | 0.4985 | 0.9996 | n-acetyltransferase [Source:VB Community Annotation] |
| AAEL000782 | | 0.1981 | 4.1421 | 0.4986 | 0.9996 | |
| AAEL005111 | | -0.9317 | 3.3062 | 0.4986 | 0.9996 | |
| AAEL012628 | | 0.7584 | 0.0183 | 0.4986 | 0.9996 | |
| AAEL017357 | | -0.5655 | 2.1511 | 0.4987 | 0.9996 | |
| AAEL011407 | CTL20 | -0.7849 | -1.1146 | 0.4987 | 0.9996 | C-Type Lectin (CTL20) [Source:VB Community Annotation] |
| AAEL012082 | | -0.6067 | 1.4524 | 0.4987 | 0.9996 | NF-180, putative [Source:VB Community Annotation] |
| AAEL006699 | | -1.1772 | 2.5664 | 0.4989 | 0.9996 | fibrinogen and fibronectin [Source:VB Community Annotation] |
| AAEL014078 | | -0.3579 | 8.3534 | 0.4990 | 0.9996 | serine protease inhibitor, serpin [Source:VB Community Annotation] |
| AAEL014734 | | 0.6001 | 2.8488 | 0.4990 | 0.9996 | |
| AAEL018263 | | -0.7602 | -0.2687 | 0.4992 | 0.9996 | |
| AAEL027019 | NA | -0.3919 | 4.2856 | 0.4992 | 0.9996 | NA |
| AAEL007677 | | 0.2960 | 2.0570 | 0.4993 | 0.9996 | phospholysine phosphohistidine inorganic pyrophosphate phosphatase [Source:VB Community Annotation] |
| AAEL025598 | NA | -1.1131 | -2.2538 | 0.4993 | 0.9996 | NA |
| AAEL001009 | | -0.8249 | 2.7725 | 0.4994 | 0.9996 | dab2-interacting protein [Source:VB Community Annotation] |
| AAEL005228 | | -0.4041 | 2.4879 | 0.4996 | 0.9996 | |
| AAEL002781 | | -0.4737 | 7.4426 | 0.5000 | 0.9996 | galactokinase [Source:VB Community Annotation] |
| AAEL021839 | NA | 0.2178 | 4.5022 | 0.5003 | 0.9996 | NA |
| AAEL006185 | | 0.4186 | 2.3529 | 0.5003 | 0.9996 | exocyst componenet sec8 [Source:VB Community Annotation] |
| AAEL013369 | | 0.2315 | 5.5467 | 0.5003 | 0.9996 | |
| AAEL007054 | | -0.2990 | 8.8160 | 0.5003 | 0.9996 | NADH dehydrogenase, putative [Source:VB Community Annotation] |
| AAEL009542 | | 0.2322 | 2.4984 | 0.5005 | 0.9996 | |
| AAEL001596 | | -0.1675 | 6.3486 | 0.5005 | 0.9996 | Protein YIPF [Source:UniProtKB/TrEMBL;Acc:Q17KU7] |
| AAEL011136 | ASNA1-1 | -0.1770 | 5.8701 | 0.5007 | 0.9996 | arsenical pump-driving ATPase [Source:VB Community Annotation] |

| | | | | | | |
|---|---|---|---|---|---|---|
| AAEL017167 | GPRHE6 | -0.7304 | -0.8114 | 0.5008 | 0.9996 | GPCR HE6-like Family [Source:VB Community Annotation] |
| AAEL000322 | | 0.3424 | 2.8937 | 0.5011 | 0.9996 | |
| AAEL005579 | | 0.2804 | 4.9177 | 0.5012 | 0.9996 | developmentally regulated GTP-binding protein 1 (drg 1) [Source:VB Community Annotation] |
| AAEL000622 | | -0.2092 | 7.6315 | 0.5013 | 0.9996 | |
| AAEL006412 | | -0.1650 | 5.7304 | 0.5014 | 0.9996 | sodium- and chloride-dependent neurotransmitter transporter [Source:VB Community Annotation] |
| AAEL002324 | | 0.2282 | 5.0465 | 0.5014 | 0.9996 | |
| AAEL003270 | | 0.2574 | 3.9796 | 0.5016 | 0.9996 | |
| AAEL001059 | GSTD3 | -1.0484 | -0.1783 | 0.5017 | 0.9996 | glutathione S-transferase (GSTD3) [Source:VB Community Annotation] |
| AAEL025399 | NA | -0.4920 | 3.6351 | 0.5019 | 0.9996 | NA |
| AAEL011832 | | -0.3361 | 4.2641 | 0.5019 | 0.9996 | |
| AAEL019938 | NA | -0.9659 | -2.0490 | 0.5020 | 0.9996 | NA |
| AAEL019746 | NA | -0.6913 | 8.3698 | 0.5020 | 0.9996 | NA |
| AAEL006603 | | 0.2018 | 3.4851 | 0.5021 | 0.9996 | |
| AAEL011427 | | 0.3508 | 1.1471 | 0.5022 | 0.9996 | |
| AAEL023261 | NA | -0.2634 | 2.5750 | 0.5023 | 0.9996 | NA |
| AAEL024451 | NA | 1.1927 | 0.0477 | 0.5023 | 0.9996 | NA |
| AAEL008663 | | 0.7240 | -0.7086 | 0.5024 | 0.9996 | aldo-keto reductase [Source:VB Community Annotation] |
| AAEL024154 | NA | 0.5452 | 0.0374 | 0.5024 | 0.9996 | NA |
| AAEL010318 | | -0.5868 | 10.9503 | 0.5025 | 0.9996 | Polyadenylate-binding protein [Source:UniProtKB/TrEMBL;Acc:Q1HR66] |
| AAEL005870 | Fen1 | 0.2370 | 4.3151 | 0.5026 | 0.9996 | flap endonuclease-1 [Source:VB Community Annotation] |
| AAEL000488 | | -0.3308 | 4.1491 | 0.5026 | 0.9996 | |
| AAEL004183 | | 0.1943 | 3.7751 | 0.5027 | 0.9996 | |
| AAEL002825 | | 0.2731 | 8.3908 | 0.5028 | 0.9996 | NADH:ubiquinone dehydrogenase, putative [Source:VB Community Annotation] |
| AAEL007979 | | -0.2634 | 6.4318 | 0.5029 | 0.9996 | tyrosine transporter [Source:VB Community Annotation] |
| AAEL008632 | | -0.3622 | 4.0455 | 0.5029 | 0.9996 | ABC transporter [Source:VB Community Annotation] |
| AAEL021429 | NA | 0.5808 | 2.9082 | 0.5030 | 0.9996 | NA |
| AAEL000958 | | 0.2572 | 6.9199 | 0.5031 | 0.9996 | |
| AAEL019721 | NA | -0.3892 | 5.0292 | 0.5032 | 0.9996 | NA |
| AAEL009442 | | -1.0596 | -0.0447 | 0.5032 | 0.9996 | |
| AAEL017994 | | -0.4520 | 2.4764 | 0.5032 | 0.9996 | |
| AAEL012262 | | -0.2973 | 7.8258 | 0.5033 | 0.9996 | Putative neural cell adhesion molecule l1 [Source:UniProtKB/TrEMBL;Acc:A0A0N8ES29] |
| AAEL011081 | | -0.2000 | 6.7580 | 0.5034 | 0.9996 | |
| AAEL000469 | | -0.5088 | 4.3183 | 0.5034 | 0.9996 | |
| AAEL010168 | RpS2 | 0.2274 | 10.3445 | 0.5036 | 0.9996 | 40S ribosomal protein S2 [Source:VB Community Annotation] |
| AAEL019482 | NA | -0.1672 | 8.2393 | 0.5036 | 0.9996 | NA |
| AAEL027904 | NA | -0.8139 | -0.6591 | 0.5036 | 0.9996 | NA |
| AAEL021471 | NA | -0.1994 | 3.9314 | 0.5037 | 0.9996 | NA |
| AAEL004756 | | 0.2252 | 4.0301 | 0.5037 | 0.9996 | vesicle docking protein P115 [Source:VB Community Annotation] |
| AAEL024857 | NA | -0.5585 | 2.5186 | 0.5038 | 0.9996 | NA |
| AAEL008255 | | -0.1690 | 4.6405 | 0.5038 | 0.9996 | mbp-1 interacting protein-2a [Source:VB Community Annotation] |

| | | | | | | |
|---|---|---|---|---|---|---|
| AAEL004228 | | 0.1857 | 3.9734 | 0.5039 | 0.9996 | oligosaccharyl transferase [Source:VB Community Annotation] |
| AAEL006052 | | 0.3446 | 1.2617 | 0.5041 | 0.9996 | |
| AAEL022637 | NA | -1.0645 | 1.3962 | 0.5043 | 0.9996 | NA |
| AAEL010469 | | -0.9951 | 3.7520 | 0.5043 | 0.9996 | |
| AAEL008369 | | 0.2714 | 5.0497 | 0.5043 | 0.9996 | acyl phosphatase, putative [Source:VB Community Annotation] |
| AAEL005639 | | -0.2243 | 4.3629 | 0.5047 | 0.9996 | alpha-1,2-Mannosidase [Source:UniProtKB/TrEMBL;Acc:Q0IFF5] |
| AAEL014825 | | -1.1652 | 0.7615 | 0.5050 | 0.9996 | |
| AAEL008373 | | 0.2226 | 4.2472 | 0.5051 | 0.9996 | dipeptidyl-peptidase [Source:VB Community Annotation] |
| AAEL010178 | | 0.2093 | 4.2819 | 0.5052 | 0.9996 | |
| AAEL001235 | | 0.1963 | 5.8529 | 0.5052 | 0.9996 | palmitoyl-protein thioesterase [Source:VB Community Annotation] |
| AAEL022051 | NA | 0.2479 | 8.9818 | 0.5053 | 0.9996 | NA |
| AAEL025572 | NA | -1.1497 | 0.1691 | 0.5053 | 0.9996 | NA |
| AAEL019961 | NA | 0.8479 | -1.7137 | 0.5053 | 0.9996 | NA |
| AAEL006186 | | 0.1876 | 6.7206 | 0.5056 | 0.9996 | |
| AAEL004672 | | -0.2928 | 2.9907 | 0.5056 | 0.9996 | |
| AAEL003994 | | -0.4213 | 1.9147 | 0.5058 | 0.9996 | phenylalanyl-tRNA synthetase mitochondrial [Source:VB Community Annotation] |
| AAEL012187 | | 0.4213 | 3.5142 | 0.5059 | 0.9996 | lethal(3)malignant brain tumor [Source:VB Community Annotation] |
| AAEL027455 | NA | -0.9005 | 1.0088 | 0.5060 | 0.9996 | NA |
| AAEL022460 | NA | 0.5080 | 1.1036 | 0.5060 | 0.9996 | NA |
| AAEL004102 | | 0.4703 | 4.9550 | 0.5061 | 0.9996 | aldo-keto reductase [Source:VB Community Annotation] |
| AAEL021957 | NA | 0.6129 | -0.7388 | 0.5061 | 0.9996 | NA |
| AAEL003599 | | -0.2779 | 4.3544 | 0.5062 | 0.9996 | |
| AAEL005080 | | 0.3334 | 2.3576 | 0.5062 | 0.9996 | protein phosphatase-5 [Source:VB Community Annotation] |
| AAEL003257 | | -0.3075 | 2.3565 | 0.5065 | 0.9996 | |
| AAEL005866 | | 1.1971 | -1.2927 | 0.5065 | 0.9996 | succinate dehydrogenase assembly factor 2, mitochondrial precursor (SDH assembly factor 2) [Source:VB Community Annotation] |
| AAEL010685 | | -0.9053 | -1.8813 | 0.5066 | 0.9996 | |
| AAEL004041 | | 0.3371 | 5.0232 | 0.5067 | 0.9996 | flotillin-2 [Source:VB Community Annotation] |
| AAEL003471 | | -0.3271 | 2.3335 | 0.5067 | 0.9996 | |
| AAEL003978 | | 0.7740 | 0.2875 | 0.5067 | 0.9996 | |
| AAEL006404 | | -0.3879 | 1.0256 | 0.5068 | 0.9996 | |
| AAEL004976 | | 0.4042 | 1.9103 | 0.5069 | 0.9996 | |
| AAEL024325 | NA | -0.7672 | -1.1560 | 0.5070 | 0.9996 | NA |
| AAEL002078 | | 0.3862 | 3.9136 | 0.5071 | 0.9996 | |
| AAEL013467 | | 0.1544 | 5.5504 | 0.5071 | 0.9996 | |
| AAEL019826 | NA | 0.2579 | 5.9458 | 0.5072 | 0.9996 | NA |
| AAEL019780 | NA | -0.2013 | 7.1598 | 0.5072 | 0.9996 | NA |
| AAEL007162 | APG8 | 0.3025 | 6.6527 | 0.5073 | 0.9996 | autophagy related gene [Source:VB Community Annotation] |
| AAEL008279 | | -0.8007 | -1.2121 | 0.5074 | 0.9996 | |
| AAEL021570 | NA | -0.2664 | 3.8964 | 0.5074 | 0.9996 | NA |
| AAEL009130 | CYP6Z7 | -0.4719 | 4.6341 | 0.5075 | 0.9996 | cytochrome P450 [Source:VB Community Annotation] |

| | | | | | | |
|---|---|---|---|---|---|---|
| AAEL008248 | | -0.2735 | 5.4991 | 0.5076 | 0.9996 | |
| AAEL025750 | NA | -0.9560 | 1.3782 | 0.5077 | 0.9996 | NA |
| AAEL017516 | | 0.2584 | 11.1807 | 0.5078 | 0.9996 | |
| AAEL019935 | NA | -0.3439 | 8.1509 | 0.5080 | 0.9996 | NA |
| AAEL019888 | NA | 0.1827 | 3.9939 | 0.5081 | 0.9996 | NA |
| AAEL005255 | PDP1 | -0.4549 | 7.1615 | 0.5081 | 0.9996 | PAR-domain protein 1 [Source:VB Community Annotation] |
| AAEL000231 | | 0.2509 | 5.8804 | 0.5082 | 0.9996 | oligopeptidase [Source:VB Community Annotation] |
| AAEL024110 | NA | 0.2475 | 2.5092 | 0.5083 | 0.9996 | NA |
| AAEL007196 | | -0.2184 | 5.9277 | 0.5083 | 0.9996 | |
| AAEL000401 | | 0.5205 | 1.7700 | 0.5084 | 0.9996 | |
| AAEL027472 | NA | 0.2560 | 2.3985 | 0.5086 | 0.9996 | NA |
| AAEL009815 | | 0.8803 | 0.2152 | 0.5086 | 0.9996 | myotubularin [Source:VB Community Annotation] |
| AAEL009916 | | -0.2557 | 3.6942 | 0.5087 | 0.9996 | |
| AAEL018240 | | 0.1672 | 4.9250 | 0.5088 | 0.9996 | |
| AAEL007996 | | -1.3340 | 1.7661 | 0.5089 | 0.9996 | centaurin alpha [Source:VB Community Annotation] |
| AAEL006870 | | 0.2873 | 2.2723 | 0.5090 | 0.9996 | sorting nexin [Source:VB Community Annotation] |
| AAEL022590 | NA | 0.5532 | 0.1805 | 0.5091 | 0.9996 | NA |
| AAEL010244 | | 0.1922 | 5.8497 | 0.5091 | 0.9996 | abrupt protein [Source:VB Community Annotation] |
| AAEL009323 | | -1.2066 | 1.7560 | 0.5092 | 0.9996 | carbonic anhydrase precursor [Source:VB Community Annotation] |
| AAEL005347 | | 0.1785 | 4.8492 | 0.5092 | 0.9996 | |
| AAEL006474 | | 0.2558 | 4.9116 | 0.5093 | 0.9996 | ATP-dependent Lon protease, putative [Source:VB Community Annotation] |
| AAEL023585 | NA | -0.2271 | 3.3903 | 0.5094 | 0.9996 | NA |
| AAEL000281 | | -0.9241 | -1.7881 | 0.5094 | 0.9996 | |
| AAEL003977 | | -0.5266 | 3.1187 | 0.5094 | 0.9996 | elongase, putative [Source:VB Community Annotation] |
| AAEL004067 | | 0.2164 | 5.7322 | 0.5095 | 0.9996 | mitochondrial benzodiazepine receptor, putative [Source:VB Community Annotation] |
| AAEL003002 | | -0.2766 | 3.4490 | 0.5097 | 0.9996 | |
| AAEL024728 | NA | -0.6213 | 0.8385 | 0.5097 | 0.9996 | NA |
| AAEL007456 | | 0.4209 | 0.9874 | 0.5097 | 0.9996 | zinc finger protein, putative [Source:VB Community Annotation] |
| AAEL010005 | | -0.2301 | 4.8336 | 0.5098 | 0.9996 | |
| AAEL010408 | GPRGBB2 | -0.9432 | 2.9941 | 0.5099 | 0.9996 | GPCR GABA B Family [Source:VB Community Annotation] |
| AAEL001769 | | 0.2452 | 5.6066 | 0.5099 | 0.9996 | DEAD box ATP-dependent RNA helicase [Source:VB Community Annotation] |
| AAEL020550 | NA | -1.1567 | -1.3713 | 0.5100 | 0.9996 | NA |
| AAEL006168 | CLIPB42 | 0.5603 | 2.9337 | 0.5100 | 0.9996 | Clip-Domain Serine Protease family B. [Source:VB Community Annotation] |
| AAEL017188 | | -0.4006 | 3.4305 | 0.5101 | 0.9996 | |
| AAEL015107 | | 0.2637 | 5.6005 | 0.5101 | 0.9996 | |
| AAEL000551 | | -0.3076 | 6.9309 | 0.5102 | 0.9996 | |
| AAEL006747 | | -0.2776 | 2.7861 | 0.5102 | 0.9996 | |
| AAEL010757 | | -0.8983 | 3.8832 | 0.5104 | 0.9996 | |
| AAEL013230 | | 0.3978 | 4.7333 | 0.5106 | 0.9996 | coatomer delta subunit [Source:VB Community Annotation] |
| AAEL001581 | | -0.4630 | 1.3747 | 0.5106 | 0.9996 | |

| | | | | | | |
|---|---|---|---|---|---|---|
| AAEL022845 | NA | -1.0169 | 0.2429 | 0.5107 | 0.9996 | NA |
| AAEL007904 | | -0.2212 | 3.8531 | 0.5108 | 0.9996 | cleavage and polyadenylation specificity factor [Source:VB Community Annotation] |
| AAEL003331 | | -0.3056 | 3.2198 | 0.5109 | 0.9996 | |
| AAEL006140 | | -0.2888 | 2.1533 | 0.5109 | 0.9996 | mitosis inhibitor protein kinase [Source:VB Community Annotation] |
| AAEL021724 | NA | 0.9468 | -1.0993 | 0.5109 | 0.9996 | NA |
| AAEL024336 | NA | 0.3831 | 2.0381 | 0.5109 | 0.9996 | NA |
| AAEL007085 | | -0.3960 | 3.5871 | 0.5110 | 0.9996 | adult cuticle protein, putative [Source:VB Community Annotation] |
| AAEL006669 | GPRSMO | 0.2025 | 2.8845 | 0.5110 | 0.9996 | GPCR Frizzled/Smoothened Family [Source:VB Community Annotation] |
| AAEL011727 | mal2 | -0.7650 | -0.2396 | 0.5110 | 0.9996 | Molybdenum cofactor sulfurase 2 (MoCo sulfurase 2)(MOS 2)(EC 4.4.-.-)(Protein maroon-like 2)(Ma-l 2) [Source:VB Community Annotati |
| AAEL006073 | | -0.4270 | 0.2214 | 0.5110 | 0.9996 | ubiquitin-conjugating enzyme E2-230k [Source:VB Community Annotation] |
| AAEL001464 | | 0.1946 | 3.1022 | 0.5112 | 0.9996 | |
| AAEL006286 | | -0.2852 | 3.4656 | 0.5113 | 0.9996 | |
| AAEL005965 | | 0.1469 | 5.5024 | 0.5114 | 0.9996 | presenilin enhancer, putative [Source:VB Community Annotation] |
| AAEL010029 | | -0.2713 | 3.6403 | 0.5115 | 0.9996 | |
| AAEL007750 | | -0.2654 | 5.7679 | 0.5115 | 0.9996 | |
| AAEL013971 | | 0.5470 | 0.9195 | 0.5115 | 0.9996 | |
| AAEL013517 | | -0.5356 | 7.4362 | 0.5116 | 0.9996 | pupal cuticle protein 78E, putative [Source:VB Community Annotation] |
| AAEL001485 | | 0.4814 | 4.9667 | 0.5116 | 0.9996 | |
| AAEL020120 | NA | -0.2842 | 2.5858 | 0.5116 | 0.9996 | NA |
| AAEL000240 | | -0.1650 | 5.5321 | 0.5117 | 0.9996 | |
| AAEL020097 | NA | 0.4305 | 8.4015 | 0.5118 | 0.9996 | NA |
| AAEL006572 | | 0.4558 | 8.3284 | 0.5118 | 0.9996 | troponin C [Source:VB Community Annotation] |
| AAEL009851 | | 0.2616 | 2.9396 | 0.5121 | 0.9996 | DNA polymerase zeta catalytic subunit [Source:VB Community Annotation] |
| AAEL005204 | | 1.2499 | 1.4871 | 0.5121 | 0.9996 | |
| AAEL005197 | | 0.1841 | 5.1995 | 0.5122 | 0.9996 | |
| AAEL011121 | | 0.1976 | 3.2530 | 0.5123 | 0.9996 | CAP10 family protein AAEL011121 Precursor [Source:VB Community Annotation] |
| AAEL025431 | NA | 0.1585 | 5.2390 | 0.5124 | 0.9996 | NA |
| AAEL002651 | | 0.2085 | 3.9445 | 0.5125 | 0.9996 | |
| AAEL013404 | | -0.8652 | 4.7872 | 0.5125 | 0.9996 | tetraspanin, putative [Source:VB Community Annotation] |
| AAEL004455 | | 0.4302 | 5.1973 | 0.5127 | 0.9996 | |
| AAEL019491 | NA | 0.2388 | 2.8189 | 0.5129 | 0.9996 | NA |
| AAEL011957 | | -0.2736 | 4.5630 | 0.5129 | 0.9996 | elongase, putative [Source:VB Community Annotation] |
| AAEL001243 | | -0.3138 | 1.3448 | 0.5130 | 0.9996 | growth factor independence [Source:VB Community Annotation] |
| AAEL006676 | | 0.2535 | 5.7281 | 0.5132 | 0.9996 | |
| AAEL023729 | NA | 1.3145 | 1.2137 | 0.5132 | 0.9996 | NA |
| AAEL008002 | | -0.2426 | 8.5175 | 0.5133 | 0.9996 | mitochondrial NADH:ubiquinone oxidoreductase ESSS subunit, putative [Source:VB Community Annotation] |
| AAEL010427 | APG6 | 0.1670 | 4.5903 | 0.5133 | 0.9996 | autophagy related gene [Source:VB Community Annotation] |
| AAEL022431 | NA | -0.7835 | 2.0111 | 0.5133 | 0.9996 | NA |
| AAEL010694 | | 0.4385 | 4.8277 | 0.5134 | 0.9996 | 5-formyltetrahydrofolate cyclo-ligase [Source:UniProtKB/TrEMBL;Acc:Q16S54] |
| AAEL001568 | | 0.5381 | 0.3504 | 0.5134 | 0.9996 | glycine receptor beta precursor, putative [Source:VB Community Annotation] |

| | | | | | | |
|---|---|---|---|---|---|---|
| AAEL005429 | | 0.5475 | 2.3650 | 0.5134 | 0.9996 | 2-oxoglutarate dehydrogenase [Source:VB Community Annotation] |
| AAEL024184 | NA | 0.2341 | 6.1640 | 0.5135 | 0.9996 | NA |
| AAEL003910 | | -0.2182 | 4.9505 | 0.5135 | 0.9996 | adam [Source:VB Community Annotation] |
| AAEL025314 | NA | -0.9392 | -1.3987 | 0.5136 | 0.9996 | NA |
| AAEL021842 | NA | -0.2629 | 3.8292 | 0.5136 | 0.9996 | NA |
| AAEL027597 | NA | -0.8858 | 3.0752 | 0.5137 | 0.9996 | NA |
| AAEL000315 | | 0.1749 | 5.1313 | 0.5137 | 0.9996 | pigeon protein (linotte protein) [Source:VB Community Annotation] |
| AAEL008374 | | 0.2375 | 5.5334 | 0.5138 | 0.9996 | E3 ubiquitin-protein ligase nedd-4 [Source:VB Community Annotation] |
| AAEL020155 | NA | -0.6696 | -2.8702 | 0.5139 | 0.9996 | NA |
| AAEL019909 | NA | 0.2517 | 4.0654 | 0.5140 | 0.9996 | NA |
| AAEL000455 | | 0.2865 | 4.9474 | 0.5141 | 0.9996 | |
| AAEL001556 | | -0.3776 | 1.3262 | 0.5142 | 0.9996 | |
| AAEL015048 | | -0.2726 | 2.2012 | 0.5142 | 0.9996 | |
| AAEL008509 | Aats-ala-m | 0.2527 | 2.7567 | 0.5143 | 0.9996 | mitochondrial alanyl-tRNA synthetase [Source:VB Community Annotation] |
| AAEL021367 | NA | 0.2769 | 6.1835 | 0.5143 | 0.9996 | NA |
| AAEL001863 | | -0.4913 | 9.5210 | 0.5144 | 0.9996 | zinc carboxypeptidase [Source:VB Community Annotation] |
| AAEL010642 | | -0.2251 | 3.6320 | 0.5147 | 0.9996 | poly(A)-binding protein, putative [Source:VB Community Annotation] |
| AAEL009326 | | -0.5878 | 4.7525 | 0.5147 | 0.9996 | fragile X mental retardation syndrome-related protein 1, putative [Source:VB Community Annotation] |
| AAEL009464 | | 0.3812 | 1.6039 | 0.5148 | 0.9996 | knolle, putative [Source:VB Community Annotation] |
| AAEL007318 | | 0.2265 | 3.5675 | 0.5149 | 0.9996 | |
| AAEL009313 | | 0.1947 | 7.2487 | 0.5150 | 0.9996 | elongation factor -1 beta,delta [Source:VB Community Annotation] |
| AAEL011441 | | 0.2381 | 4.6460 | 0.5150 | 0.9996 | calcium/calmodulin-dependent protein kinase type 1 (camki) [Source:VB Community Annotation] |
| AAEL000092 | GSTX1 | 0.9034 | -0.1767 | 0.5151 | 0.9996 | glutathione transferase [Source:VB Community Annotation] |
| AAEL023098 | NA | -0.3363 | 6.9324 | 0.5153 | 0.9996 | NA |
| AAEL025391 | NA | -0.3752 | 8.0705 | 0.5154 | 0.9996 | NA |
| AAEL004761 | | -0.2806 | 2.9297 | 0.5154 | 0.9996 | serine/threonine-protein kinase MAK [Source:VB Community Annotation] |
| AAEL021848 | NA | 0.2667 | 3.4280 | 0.5155 | 0.9996 | NA |
| AAEL003551 | | 0.1716 | 6.9626 | 0.5155 | 0.9996 | |
| AAEL000650 | | -0.5229 | 6.5603 | 0.5156 | 0.9996 | |
| AAEL028178 | NA | 0.5896 | 2.0922 | 0.5156 | 0.9996 | NA |
| AAEL001016 | | -0.4575 | 0.9455 | 0.5157 | 0.9996 | zinc finger protein [Source:VB Community Annotation] |
| AAEL017419 | | -0.2550 | 9.3016 | 0.5160 | 0.9996 | |
| AAEL005861 | | 0.2142 | 6.1641 | 0.5161 | 0.9996 | vacuolar sorting protein (vps) [Source:VB Community Annotation] |
| AAEL009537 | | -1.0136 | -1.2910 | 0.5164 | 0.9996 | |
| AAEL011085 | | -0.7931 | -0.7714 | 0.5164 | 0.9996 | |
| AAEL000785 | | 0.1813 | 4.1889 | 0.5164 | 0.9996 | |
| AAEL013478 | | 0.2549 | 3.4196 | 0.5165 | 0.9996 | |
| AAEL005600 | | 0.2420 | 3.1942 | 0.5165 | 0.9996 | |
| AAEL011059 | | -0.2136 | 5.1382 | 0.5166 | 0.9996 | |
| AAEL012499 | | 0.3431 | 1.8525 | 0.5166 | 0.9996 | Histone H2A [Source:UniProtKB/TrEMBL;Acc:Q16LW9] |

| | | | | | | |
|---|---|---|---|---|---|---|
| AAEL026560 | NA | -1.2380 | 1.6063 | 0.5166 | 0.9996 | NA |
| AAEL014289 | | 0.2960 | 2.8048 | 0.5167 | 0.9996 | |
| AAEL010468 | | 0.2137 | 3.4580 | 0.5167 | 0.9996 | DNA-directed RNA polymerase I, 12kD-subunit, putative [Source:VB Community Annotation] |
| AAEL002369 | | 0.2295 | 4.1473 | 0.5168 | 0.9996 | |
| AAEL009893 | | 0.2286 | 4.6713 | 0.5168 | 0.9996 | |
| AAEL007062 | | -0.1659 | 7.1566 | 0.5169 | 0.9996 | |
| AAEL015336 | | 0.3427 | 3.1957 | 0.5169 | 0.9996 | |
| AAEL013221 | RpL10a | 0.1813 | 10.6930 | 0.5170 | 0.9996 | 60S ribosomal protein L10a [Source:VB Community Annotation] |
| AAEL018113 | | 0.2810 | 3.1186 | 0.5171 | 0.9996 | |
| AAEL000558 | | -0.2447 | 5.5301 | 0.5172 | 0.9996 | neural stem cell-derived dendrite regulator [Source:VB Community Annotation] |
| AAEL009285 | | -0.2815 | 3.9448 | 0.5172 | 0.9996 | DEAD box ATP-dependent RNA helicase [Source:VB Community Annotation] |
| AAEL004898 | | 0.4543 | 2.9626 | 0.5173 | 0.9996 | |
| AAEL020794 | NA | -0.5321 | 1.3040 | 0.5174 | 0.9996 | NA |
| AAEL011281 | | -0.6213 | 0.7246 | 0.5176 | 0.9996 | cytochrome B561 [Source:VB Community Annotation] |
| AAEL005289 | | 0.3699 | 5.4546 | 0.5177 | 0.9996 | ornithine aminotransferase [Source:VB Community Annotation] |
| AAEL008062 | | -0.4660 | 3.2684 | 0.5179 | 0.9996 | thrombospondin [Source:VB Community Annotation] |
| AAEL006556 | | -0.3452 | 1.5761 | 0.5180 | 0.9996 | |
| AAEL002191 | | -1.0693 | -0.7500 | 0.5180 | 0.9996 | cuticle protein, putative [Source:VB Community Annotation] |
| AAEL005171 | | -0.3282 | 3.0788 | 0.5180 | 0.9996 | |
| AAEL009407 | | 0.1716 | 5.0243 | 0.5181 | 0.9996 | |
| AAEL018221 | | 0.9744 | 0.2892 | 0.5181 | 0.9996 | |
| AAEL009360 | | -0.5943 | 1.1065 | 0.5182 | 0.9996 | serine/threonine-protein kinase PLK4 (EC 2.7.11.21)(Polo-like kinase 4)(PLK-4)(Serine/threonine-protein kinase SAK) [Source:VB Commur |
| AAEL023229 | NA | -0.3138 | 3.9446 | 0.5182 | 0.9996 | NA |
| AAEL011277 | | -0.2151 | 6.2283 | 0.5183 | 0.9996 | apoptosis stimulating of P53 [Source:VB Community Annotation] |
| AAEL005048 | | 0.4524 | 4.7016 | 0.5183 | 0.9996 | |
| AAEL007607 | | -0.5488 | 0.2622 | 0.5186 | 0.9996 | |
| AAEL021791 | NA | -0.7864 | 1.1589 | 0.5187 | 0.9996 | NA |
| AAEL020123 | NA | -0.5112 | 4.6197 | 0.5187 | 0.9996 | NA |
| AAEL026571 | NA | -0.2263 | 4.7477 | 0.5188 | 0.9996 | NA |
| AAEL012725 | | -0.2717 | 2.3899 | 0.5188 | 0.9996 | |
| AAEL017334 | | 0.9396 | 2.9040 | 0.5189 | 0.9996 | |
| AAEL019697 | NA | -0.3974 | 4.8403 | 0.5190 | 0.9996 | NA |
| AAEL019558 | NA | 0.1817 | 5.3992 | 0.5190 | 0.9996 | NA |
| AAEL004397 | | 0.8321 | -0.3629 | 0.5190 | 0.9996 | |
| AAEL003298 | | 0.8824 | 0.2337 | 0.5191 | 0.9996 | Alkaline phosphatase [Source:UniProtKB/TrEMBL;Acc:Q17FS9] |
| AAEL000572 | | -0.1599 | 5.2887 | 0.5191 | 0.9996 | AMP dependent ligase [Source:VB Community Annotation] |
| AAEL013550 | | 0.2892 | 2.6520 | 0.5194 | 0.9996 | |
| AAEL009843 | | 1.1319 | -1.3335 | 0.5196 | 0.9996 | serine-type enodpeptidase, [Source:VB Community Annotation] |
| AAEL000289 | | 0.1941 | 3.4681 | 0.5197 | 0.9996 | |
| AAEL001636 | | 0.2870 | 4.8937 | 0.5198 | 0.9996 | nuclear transcription factor, x-box binding 1 (nfx1) [Source:VB Community Annotation] |

| | | | | | | |
|---|---|---|---|---|---|---|
| AAEL002506 | | 0.2079 | 4.9263 | 0.5198 | 0.9996 | glutamate receptor, ionotropic kainate 1, 2, 3 (glur5, glur6, glur7) [Source:VB Community Annotation] |
| AAEL005950 | | -0.3470 | 4.0063 | 0.5199 | 0.9996 | chloride channel protein 2 [Source:VB Community Annotation] |
| AAEL027520 | NA | 0.3359 | 1.9440 | 0.5199 | 0.9996 | NA |
| AAEL003961 | | 0.4867 | 2.7786 | 0.5199 | 0.9996 | short-chain dehydrogenase [Source:VB Community Annotation] |
| AAEL023716 | NA | 0.1847 | 5.0526 | 0.5199 | 0.9996 | NA |
| AAEL006696 | | -1.0027 | 0.1873 | 0.5200 | 0.9996 | lumbrokinase-3T2, putative [Source:VB Community Annotation] |
| AAEL003861 | | -0.4220 | 6.2080 | 0.5201 | 0.9996 | bmp-induced factor [Source:VB Community Annotation] |
| AAEL022782 | NA | -1.0519 | -0.0080 | 0.5202 | 0.9996 | NA |
| AAEL009339 | | -0.6662 | 2.6018 | 0.5203 | 0.9996 | activin receptor type I, putative [Source:VB Community Annotation] |
| AAEL016984 | | 0.3355 | 11.3648 | 0.5203 | 0.9996 | Glyceraldehyde-3-phosphate dehydrogenase [Source:UniProtKB/TrEMBL;Acc:J9HYM2] |
| AAEL024497 | NA | 0.2361 | 6.9071 | 0.5204 | 0.9996 | NA |
| AAEL010386 | | -0.5093 | 1.5054 | 0.5204 | 0.9996 | glucosyl/glucuronosyl transferases [Source:VB Community Annotation] |
| AAEL012063 | | -0.3996 | 2.0054 | 0.5204 | 0.9996 | |
| AAEL022155 | NA | 0.6800 | -0.8822 | 0.5205 | 0.9996 | NA |
| AAEL010716 | | 0.2114 | 5.0682 | 0.5206 | 0.9996 | preprotein translocase secy subunit (sec61) [Source:VB Community Annotation |
| AAEL008128 | | 0.2460 | 5.2400 | 0.5206 | 0.9996 | mitochondrial inner membrane protein translocase, 13kD-subunit, putative [Source:VB Community Annotation] |
| AAEL000106 | | 0.2033 | 3.1593 | 0.5206 | 0.9996 | |
| AAEL024649 | NA | 0.8198 | -1.8878 | 0.5207 | 0.9996 | NA |
| AAEL012013 | | -0.4259 | 1.8138 | 0.5208 | 0.9996 | |
| AAEL022204 | NA | -0.7473 | -1.1869 | 0.5208 | 0.9996 | NA |
| AAEL010052 | | -0.1617 | 4.6455 | 0.5209 | 0.9996 | |
| AAEL007501 | | 0.2719 | 5.9045 | 0.5209 | 0.9996 | nikI, nikkomycin biosynthesis protein P6, putative [Source:VB Community Annotation] |
| AAEL024871 | NA | -0.9265 | -1.2352 | 0.5210 | 0.9996 | NA |
| AAEL003365 | | 0.2864 | 6.4782 | 0.5211 | 0.9996 | fumarylacetoacetate hydrolase [Source:VB Community Annotation] |
| AAEL011653 | | -0.4043 | 0.4210 | 0.5211 | 0.9996 | thyroid hormone receptor interactor [Source:VB Community Annotation] |
| AAEL023322 | NA | 1.1083 | -0.3016 | 0.5212 | 0.9996 | NA |
| AAEL026845 | NA | 0.7239 | -0.2448 | 0.5212 | 0.9996 | NA |
| AAEL009084 | | -0.2643 | 4.5622 | 0.5213 | 0.9996 | slender lobes, putative [Source:VB Community Annotation] |
| AAEL025650 | NA | -0.6955 | 1.7223 | 0.5214 | 0.9996 | NA |
| AAEL011862 | | -0.9866 | -1.1816 | 0.5214 | 0.9996 | |
| AAEL022485 | NA | 0.2075 | 4.7114 | 0.5217 | 0.9996 | NA |
| AAEL016960 | | 0.2630 | 4.5681 | 0.5219 | 0.9996 | |
| AAEL003594 | | 0.4826 | 2.0298 | 0.5220 | 0.9996 | kinectin, putative [Source:VB Community Annotation] |
| AAEL003661 | | -0.2259 | 5.3532 | 0.5220 | 0.9996 | translation initiation factor [Source:VB Community Annotation] |
| AAEL012860 | | 0.2611 | 6.6485 | 0.5221 | 0.9996 | |
| AAEL004467 | | 0.1770 | 6.0638 | 0.5223 | 0.9996 | |
| AAEL009038 | | -0.2802 | 5.0971 | 0.5223 | 0.9996 | prolylcarboxypeptidase, putative [Source:VB Community Annotation] |
| AAEL002711 | | 0.8219 | 1.1814 | 0.5224 | 0.9996 | |
| AAEL017283 | | 0.2851 | 2.0240 | 0.5225 | 0.9996 | |
| AAEL002311 | | 0.2299 | 4.9202 | 0.5226 | 0.9996 | ADP-ribosylation factor, putative [Source:VB Community Annotation] |

| | | | | | | |
|---|---|---|---|---|---|---|
| AAEL009509 | | -0.3097 | 4.7621 | 0.5226 | 0.9996 | zinc finger protein [Source:VB Community Annotation] |
| AAEL003355 | | -0.8830 | 2.1159 | 0.5227 | 0.9996 | |
| AAEL005884 | | 0.3017 | 6.3146 | 0.5228 | 0.9996 | |
| AAEL006838 | | -0.2408 | 3.6316 | 0.5228 | 0.9996 | |
| AAEL003100 | | -1.1929 | 5.1469 | 0.5229 | 0.9996 | |
| AAEL001128 | | -0.5793 | 6.0146 | 0.5229 | 0.9996 | AMP dependent coa ligase [Source:VB Community Annotation] |
| AAEL008692 | | 0.2998 | 6.0272 | 0.5229 | 0.9996 | barrier-to-autointegration factor, putative [Source:VB Community Annotation] |
| AAEL010854 | | 0.2898 | 6.0768 | 0.5231 | 0.9996 | ubiquitin specific protease 2, [Source:VB Community Annotation] |
| AAEL022478 | NA | -0.9605 | 3.2023 | 0.5231 | 0.9996 | NA |
| AAEL001932 | FADD | 0.2767 | 3.0064 | 0.5231 | 0.9996 | IMD pathway signalling  Fas-Associated Death Domain  (FADD). [Source:VB Community Annotation] |
| AAEL000899 | | 0.3003 | 3.6215 | 0.5233 | 0.9996 | glycosyltransferase [Source:VB Community Annotation] |
| AAEL001422 | | 0.2815 | 5.6408 | 0.5234 | 0.9996 | proteasome inhibitor [Source:VB Community Annotation] |
| AAEL015402 | | 0.2995 | 3.2468 | 0.5234 | 0.9996 | |
| AAEL012417 | | 0.2201 | 3.8734 | 0.5234 | 0.9996 | |
| AAEL005956 | CASPS16 | 0.4133 | 0.4652 | 0.5235 | 0.9996 | caspase (short) [Source:VB Community Annotation] |
| AAEL010340 | | -0.2434 | 5.2880 | 0.5236 | 0.9996 | serine/arginine rich splicing factor [Source:VB Community Annotation] |
| AAEL001992 | | -0.2813 | 5.6715 | 0.5237 | 0.9996 | |
| AAEL024175 | NA | 0.3540 | 3.2297 | 0.5237 | 0.9996 | NA |
| AAEL018715 | aae-mir-93 | 0.8421 | -1.2992 | 0.5238 | 0.9996 | |
| AAEL024608 | NA | 0.3781 | 1.8034 | 0.5239 | 0.9996 | NA |
| AAEL005169 | | -0.2614 | 4.0429 | 0.5239 | 0.9996 | carnitine o-acyltransferase [Source:VB Community Annotation] |
| AAEL019824 | NA | -0.2941 | 5.6114 | 0.5239 | 0.9996 | NA |
| AAEL003915 | | 0.2252 | 3.5710 | 0.5240 | 0.9996 | acid phosphatase-1 [Source:VB Community Annotation] |
| AAEL021625 | NA | 0.2914 | 3.2801 | 0.5241 | 0.9996 | NA |
| AAEL010472 | | 0.5544 | 0.6998 | 0.5243 | 0.9996 | helix-loop-helix protein hen [Source:VB Community Annotation] |
| AAEL009926 | Exo2 | -0.2329 | 3.5553 | 0.5244 | 0.9996 | exocyst complex component 2 [Source:VB Community Annotation] |
| AAEL015022 | | -0.2811 | 5.8835 | 0.5244 | 0.9996 | glycoside  hydrolases [Source:VB Community Annotation] |
| AAEL023126 | NA | -0.5671 | 0.2028 | 0.5245 | 0.9996 | NA |
| AAEL019890 | NA | -0.2335 | 3.5879 | 0.5245 | 0.9996 | NA |
| AAEL009977 | | 0.5441 | -0.0351 | 0.5246 | 0.9996 | |
| AAEL024527 | NA | -0.2040 | 4.7126 | 0.5247 | 0.9996 | NA |
| AAEL004364 | | 0.2607 | 4.3956 | 0.5249 | 0.9996 | |
| AAEL000265 | | 0.2002 | 6.6735 | 0.5250 | 0.9996 | Protein crossbronx homolog [Source:UniProtKB/Swiss-Prot;Acc:Q17PP1] |
| AAEL006205 | | -0.2807 | 4.3414 | 0.5250 | 0.9996 | |
| AAEL005036 | | 0.1816 | 3.9594 | 0.5252 | 0.9996 | |
| AAEL000107 | | 0.2382 | 1.9548 | 0.5252 | 0.9996 | |
| AAEL006593 | | 0.2237 | 3.2149 | 0.5253 | 0.9996 | |
| AAEL010863 | | -0.5813 | -0.5509 | 0.5256 | 0.9996 | |
| AAEL013556 | CYP4J15 | -0.4840 | 1.2917 | 0.5256 | 0.9996 | cytochrome P450 [Source:VB Community Annotation] |
| AAEL024482 | NA | 0.8323 | 5.0473 | 0.5257 | 0.9996 | NA |

| | | | | | | |
|---|---|---|---|---|---|---|
| AAEL023646 | NA | 0.2653 | 2.6577 | 0.5257 | 0.9996 | NA |
| AAEL024934 | NA | -0.6696 | -2.5963 | 0.5257 | 0.9996 | NA |
| AAEL008823 | | -1.2729 | 3.1158 | 0.5257 | 0.9996 | rac serine/threonine kinase [Source:VB Community Annotation] |
| AAEL025700 | NA | -0.7276 | -2.2869 | 0.5258 | 0.9996 | NA |
| AAEL005281 | | 0.6808 | -0.3319 | 0.5259 | 0.9996 | |
| AAEL007955 | GSTE8 | -0.3042 | 5.9478 | 0.5259 | 0.9996 | glutathione transferase [Source:VB Community Annotation] |
| AAEL009607 | | 0.1644 | 6.3850 | 0.5261 | 0.9996 | Protein lingerer [Source:VB Community Annotation] |
| AAEL010447 | | 0.4170 | 1.8511 | 0.5261 | 0.9996 | |
| AAEL003698 | | 0.1867 | 4.4463 | 0.5262 | 0.9996 | |
| AAEL022715 | NA | 0.7004 | -0.6950 | 0.5263 | 0.9996 | NA |
| AAEL024519 | NA | -0.5293 | 2.5301 | 0.5266 | 0.9996 | NA |
| AAEL011966 | | -0.8547 | -2.4054 | 0.5267 | 0.9996 | |
| AAEL021733 | NA | 0.5948 | 2.3603 | 0.5267 | 0.9996 | NA |
| AAEL020488 | NA | -0.6595 | 8.2556 | 0.5269 | 0.9996 | NA |
| AAEL023715 | NA | -0.8353 | -0.7992 | 0.5269 | 0.9996 | NA |
| AAEL007785 | | 0.2211 | 5.8808 | 0.5270 | 0.9996 | leucine-rich transmembrane protein [Source:VB Community Annotation] |
| AAEL005970 | | 0.1865 | 4.6478 | 0.5271 | 0.9996 | |
| AAEL008599 | | 0.7641 | -1.1051 | 0.5272 | 0.9996 | zinc carboxypeptidase [Source:VB Community Annotation] |
| AAEL006949 | | 0.3396 | 3.6108 | 0.5273 | 0.9996 | |
| AAEL024562 | NA | -0.3587 | 2.2551 | 0.5273 | 0.9996 | NA |
| AAEL022346 | NA | 0.2773 | 5.1927 | 0.5273 | 0.9996 | NA |
| AAEL004575 | | -0.2917 | 5.7014 | 0.5273 | 0.9996 | Beta-galactosidase [Source:UniProtKB/TrEMBL;Acc:Q17CH5] |
| AAEL004037 | | -1.1511 | -1.1717 | 0.5274 | 0.9996 | p15-2b protein, putative [Source:VB Community Annotation] |
| AAEL025265 | NA | -0.3382 | 2.2037 | 0.5274 | 0.9996 | NA |
| AAEL025143 | NA | -0.2394 | 6.4874 | 0.5275 | 0.9996 | NA |
| AAEL010145 | | -0.2723 | 8.3166 | 0.5276 | 0.9996 | sodium/potassium-dependent ATPase beta-2 subunit [Source:VB Community Annotation] |
| AAEL000219 | | 0.2732 | 4.8797 | 0.5276 | 0.9996 | lactoylglutathione lyase [Source:VB Community Annotation] |
| AAEL000755 | | 0.3027 | 2.7094 | 0.5277 | 0.9996 | |
| AAEL002593 | | -0.3419 | 7.4788 | 0.5277 | 0.9996 | serine protease [Source:VB Community Annotation] |
| AAEL010478 | | -1.0683 | -1.7218 | 0.5280 | 0.9996 | sugar transporter [Source:VB Community Annotation] |
| AAEL000659 | | 0.3709 | 2.5540 | 0.5280 | 0.9996 | exosome complex exonuclease rrp43 (ribosomal RNA processing protein 43) [Source:VB Community Annotation] |
| AAEL012446 | IAP6 | 0.1737 | 5.5116 | 0.5281 | 0.9996 | Inhibitor of Apoptosis (IAP) containing Baculoviral IAP Repeat(s) (BIR domains). [Source:VB Community Annotation] |
| AAEL006987 | | 0.9893 | 0.3767 | 0.5281 | 0.9996 | |
| AAEL007283 | | 0.7724 | 3.5806 | 0.5282 | 0.9996 | acetyl-coa synthetase [Source:VB Community Annotation] |
| AAEL011074 | | 0.2958 | 2.8830 | 0.5282 | 0.9996 | dedicator of cytokinesis protein 2 [Source:VB Community Annotation] |
| AAEL026633 | NA | -0.1426 | 6.6173 | 0.5283 | 0.9996 | NA |
| AAEL025309 | NA | -0.5642 | -0.4254 | 0.5284 | 0.9996 | NA |
| AAEL001887 | | 0.3536 | 7.4956 | 0.5285 | 0.9996 | glutamine synthetase 1, 2 (glutamate-amonia ligase) (gs) [Source:VB Community Annotation] |
| AAEL003046 | | -0.9879 | 4.3209 | 0.5285 | 0.9996 | saposin [Source:VB Community Annotation] |
| AAEL020028 | NA | -0.7703 | 7.0827 | 0.5287 | 0.9996 | NA |

| | | | | | | |
|---|---|---|---|---|---|---|
| AAEL010315 | | 0.4188 | 0.8237 | 0.5288 | 0.9996 | |
| AAEL000485 | | -0.3824 | 5.8837 | 0.5292 | 0.9996 | paramyosin, putative [Source:VB Community Annotation] |
| AAEL018174 | | 0.2584 | 4.3659 | 0.5292 | 0.9996 | |
| AAEL028742 | NA | -0.6168 | 1.8344 | 0.5296 | 0.9996 | NA |
| AAEL001506 | | -0.2468 | 4.1303 | 0.5298 | 0.9996 | U3 small nucleolar ribonucleoprotein protein mpp10 [Source:VB Community Annotation] |
| AAEL014749 | | -1.3575 | 1.1773 | 0.5300 | 0.9996 | ral [Source:VB Community Annotation] |
| AAEL002876 | | -0.5837 | 6.3472 | 0.5300 | 0.9996 | |
| AAEL001651 | | -0.4836 | 1.8183 | 0.5304 | 0.9996 | Putative regulator of nuclear mrna [Source:UniProtKB/TrEMBL;Acc:A0A0P6ITS4] |
| AAEL002048 | | 0.2171 | 4.2544 | 0.5305 | 0.9996 | histidyl-tRNA synthetase [Source:VB Community Annotation] |
| AAEL007858 | | 0.2980 | 2.5828 | 0.5308 | 0.9996 | ran GTPase-activating protein [Source:VB Community Annotation] |
| AAEL022522 | NA | 0.6320 | -0.3180 | 0.5310 | 0.9996 | NA |
| AAEL026941 | NA | 0.6762 | -1.2603 | 0.5311 | 0.9996 | NA |
| AAEL026714 | NA | 0.3730 | -0.0330 | 0.5311 | 0.9996 | NA |
| AAEL024425 | NA | -0.2732 | 6.0719 | 0.5312 | 0.9996 | NA |
| AAEL025868 | NA | 0.3188 | 2.1464 | 0.5313 | 0.9996 | NA |
| AAEL023718 | NA | 0.2547 | 2.9874 | 0.5315 | 0.9996 | NA |
| AAEL019615 | NA | -0.6679 | -0.8547 | 0.5316 | 0.9996 | NA |
| AAEL028229 | NA | -0.5855 | 0.5467 | 0.5317 | 0.9996 | NA |
| AAEL003242 | | 0.6139 | -0.0681 | 0.5319 | 0.9996 | pupal cuticle protein, putative [Source:VB Community Annotation] |
| AAEL005535 | | -0.3433 | 4.8555 | 0.5319 | 0.9996 | |
| AAEL014768 | | -0.3756 | 7.2992 | 0.5319 | 0.9996 | glutamate synthase [Source:VB Community Annotation] |
| AAEL013262 | | 0.3840 | 4.2017 | 0.5321 | 0.9996 | |
| AAEL008742 | | -0.1589 | 4.1680 | 0.5321 | 0.9996 | |
| AAEL002119 | | 0.4108 | 3.0975 | 0.5323 | 0.9996 | ras [Source:VB Community Annotation] |
| AAEL016231 | tRNA-Asn | 0.3880 | 1.8820 | 0.5325 | 0.9996 | |
| AAEL005726 | | -0.2275 | 4.0950 | 0.5326 | 0.9996 | breakpoint cluster region protein (bcr) [Source:VB Community Annotation] |
| AAEL026256 | NA | -0.2409 | 2.9158 | 0.5327 | 0.9996 | NA |
| AAEL007535 | | 0.3398 | 2.8972 | 0.5327 | 0.9996 | |
| AAEL027436 | NA | 0.2092 | 5.4708 | 0.5328 | 0.9996 | NA |
| AAEL027632 | NA | 0.9177 | -1.6089 | 0.5328 | 0.9996 | NA |
| AAEL005391 | | 0.1797 | 4.6564 | 0.5328 | 0.9996 | syntenin [Source:VB Community Annotation] |
| AAEL011376 | | -0.8464 | -1.5720 | 0.5329 | 0.9996 | mitosis inhibitor protein kinase [Source:VB Community Annotation] |
| AAEL002567 | | -0.8733 | -1.0405 | 0.5331 | 0.9996 | |
| AAEL017204 | | -0.2664 | 4.4032 | 0.5331 | 0.9996 | Transmembrane 9 superfamily member [Source:UniProtKB/TrEMBL;Acc:J9HZW5] |
| AAEL009002 | | 0.5894 | 1.5657 | 0.5333 | 0.9996 | |
| AAEL002579 | | -0.7256 | -2.1080 | 0.5334 | 0.9996 | |
| AAEL004529 | | 0.2181 | 4.4062 | 0.5334 | 0.9996 | glyoxylate/hydroxypyruvate reductase [Source:VB Community Annotation] |
| AAEL002546 | | 0.2652 | 2.6926 | 0.5335 | 0.9996 | |
| AAEL008382 | | -0.8718 | 1.7911 | 0.5335 | 0.9996 | |
| AAEL005152 | | 0.2306 | 3.0578 | 0.5336 | 0.9996 | |

| | | | | | | |
|---|---|---|---|---|---|---|
| AAEL014314 | | 0.4660 | 0.0567 | 0.5337 | 0.9996 | DNA primase [Source:UniProtKB/TrEMBL;Acc:Q16GN9] |
| AAEL026218 | NA | 0.3115 | 4.5968 | 0.5337 | 0.9996 | NA |
| AAEL009810 | | -0.3154 | 6.9131 | 0.5337 | 0.9996 | sideroflexin 1,2,3 [Source:VB Community Annotation] |
| AAEL009212 | | 0.2308 | 4.7103 | 0.5337 | 0.9996 | lola [Source:VB Community Annotation] |
| AAEL020230 | NA | -1.0091 | -0.1058 | 0.5338 | 0.9996 | NA |
| AAEL008275 | | -0.8777 | -1.2067 | 0.5339 | 0.9996 | |
| AAEL001360 | | 0.4528 | 3.7116 | 0.5342 | 0.9996 | Coronin [Source:UniProtKB/TrEMBL;Acc:Q17LF4] |
| AAEL005651 | | -0.2261 | 7.2476 | 0.5342 | 0.9996 | ethanolamine-phosphate cytidylyltransferase [Source:VB Community Annotation] |
| AAEL021986 | NA | -0.2642 | 3.5136 | 0.5343 | 0.9996 | NA |
| AAEL012502 | | -0.2842 | 3.4894 | 0.5348 | 0.9996 | |
| AAEL003952 | | -0.1618 | 5.9804 | 0.5348 | 0.9996 | calpain 4, 6, 7, invertebrate [Source:VB Community Annotation] |
| AAEL022008 | NA | 0.5529 | 0.2920 | 0.5348 | 0.9996 | NA |
| AAEL009538 | | 0.2064 | 6.0454 | 0.5349 | 0.9996 | |
| AAEL019454 | NA | -0.4985 | 4.6639 | 0.5349 | 0.9996 | NA |
| AAEL005375 | | -0.9869 | -1.3386 | 0.5350 | 0.9996 | glucosyl/glucuronosyl transferases [Source:VB Community Annotation] |
| AAEL003103 | | -0.1736 | 8.3042 | 0.5353 | 0.9996 | ubiquitin-conjugating enzyme E2 [Source:VB Community Annotation] |
| AAEL012184 | | 0.2195 | 3.9620 | 0.5354 | 0.9996 | glutaminyl-peptide cyclotransferase [Source:VB Community Annotation] |
| AAEL027694 | NA | -0.4138 | 4.6389 | 0.5354 | 0.9996 | NA |
| AAEL010235 | | -1.1566 | 10.0732 | 0.5355 | 0.9996 | 30 kDa salivary gland allergen Aed a 3 Precursor (Allergen Aed a 3) [Source:VB Community Annotation] |
| AAEL009779 | | -0.2130 | 5.0243 | 0.5356 | 0.9996 | NBP2b protein, putative [Source:VB Community Annotation] |
| AAEL014136 | | 0.5139 | -0.4858 | 0.5357 | 0.9996 | |
| AAEL002108 | | -0.2132 | 3.5791 | 0.5358 | 0.9996 | nucleolar essential protein 1 (nep1) [Source:VB Community Annotation] |
| AAEL025971 | NA | -0.3561 | 3.3146 | 0.5358 | 0.9996 | NA |
| AAEL007896 | | -0.3762 | 0.7487 | 0.5358 | 0.9996 | |
| AAEL018060 | | 0.5258 | -0.5203 | 0.5359 | 0.9996 | |
| AAEL000298 | | -0.7257 | -1.6179 | 0.5359 | 0.9996 | sodium/chloride dependent amino acid transporter [Source:VB Community Annotation] |
| AAEL009818 | | -0.1912 | 7.1830 | 0.5360 | 0.9996 | |
| AAEL007541 | | 0.1691 | 4.4120 | 0.5360 | 0.9996 | DNA polymerase delta small subunit [Source:VB Community Annotation] |
| AAEL001645 | | -0.9282 | -0.6671 | 0.5361 | 0.9996 | alkaline ceramidase [Source:VB Community Annotation] |
| AAEL011983 | | 0.1979 | 3.3708 | 0.5361 | 0.9996 | scaffold protein salvador (shar-pei) [Source:VB Community Annotation] |
| AAEL020162 | NA | -0.3764 | 2.6682 | 0.5361 | 0.9996 | NA |
| AAEL003552 | | 0.1940 | 5.6017 | 0.5362 | 0.9996 | DNA-directed RNA polymerase subunit rpb6 [Source:VB Community Annotation] |
| AAEL006523 | | -0.5766 | 3.8847 | 0.5362 | 0.9996 | crk [Source:VB Community Annotation] |
| AAEL014454 | | -0.2191 | 4.5987 | 0.5363 | 0.9996 | |
| AAEL006136 | | -0.2596 | 6.9302 | 0.5365 | 0.9996 | serine protease, putative [Source:VB Community Annotation] |
| AAEL012099 | | 0.2785 | 3.7279 | 0.5366 | 0.9996 | protease m1 zinc metalloprotease [Source:VB Community Annotation] |
| AAEL000735 | | -0.5429 | 1.3440 | 0.5366 | 0.9996 | acyl-CoA oxidase [Source:VB Community Annotation] |
| AAEL006584 | | -0.6327 | -1.3204 | 0.5366 | 0.9996 | |
| AAEL027866 | NA | -0.1957 | 4.7158 | 0.5367 | 0.9996 | NA |
| AAEL004161 | | -0.2584 | 5.8870 | 0.5368 | 0.9996 | |

| | | | | | | |
|---|---|---|---|---|---|---|
| AAEL007542 | | -0.2674 | 7.5981 | 0.5372 | 0.9996 | glutamate decarboxylase [Source:VB Community Annotation] |
| AAEL005731 | | -0.1907 | 4.9582 | 0.5373 | 0.9996 | forkhead box protein (AaegFOXL) [Source:VB Community Annotation] |
| AAEL025906 | NA | 0.2350 | 5.7766 | 0.5374 | 0.9996 | NA |
| AAEL006131 | | -0.3540 | 5.1966 | 0.5375 | 0.9996 | |
| AAEL004757 | | -0.1316 | 4.9861 | 0.5377 | 0.9996 | cleavage and polyadenylation specificity factor [Source:VB Community Annotation] |
| AAEL027567 | NA | -0.2772 | 2.7685 | 0.5377 | 0.9996 | NA |
| AAEL012827 | | -0.2494 | 7.4061 | 0.5377 | 0.9996 | endoplasmin [Source:VB Community Annotation] |
| AAEL019734 | NA | -0.7768 | -1.9939 | 0.5378 | 0.9996 | NA |
| AAEL027616 | NA | -0.3321 | 4.9555 | 0.5378 | 0.9996 | NA |
| AAEL010809 | | 0.2447 | 4.3056 | 0.5379 | 0.9996 | set [Source:VB Community Annotation] |
| AAEL013355 | | 0.3967 | 3.1325 | 0.5381 | 0.9996 | |
| AAEL012914 | | -0.3414 | 4.1970 | 0.5382 | 0.9996 | |
| AAEL025880 | NA | -1.1747 | -0.1383 | 0.5382 | 0.9996 | NA |
| AAEL003677 | | -0.2466 | 2.0923 | 0.5383 | 0.9996 | |
| AAEL003982 | | -1.0877 | 4.0227 | 0.5384 | 0.9996 | |
| AAEL008431 | | 0.2502 | 5.5323 | 0.5384 | 0.9996 | kinase anchor protein [Source:VB Community Annotation] |
| AAEL022725 | NA | -0.1545 | 4.8281 | 0.5384 | 0.9996 | NA |
| AAEL013635 | | 0.8521 | -1.3888 | 0.5384 | 0.9996 | |
| AAEL004553 | | 0.2590 | 3.6687 | 0.5385 | 0.9996 | |
| AAEL007664 | | -0.8617 | 2.9326 | 0.5385 | 0.9996 | nicotinic acetylcholine receptor subunit [Source:VB Community Annotation] |
| AAEL006956 | | -0.3262 | 5.7873 | 0.5386 | 0.9996 | suppressor of ty [Source:VB Community Annotation] |
| AAEL006881 | | -0.2970 | 3.5316 | 0.5388 | 0.9996 | multiple C2 domain and transmembrane region protein, putative [Source:VB Community Annotation] |
| AAEL022663 | NA | 0.8025 | -2.0195 | 0.5389 | 0.9996 | NA |
| AAEL000258 | | 0.1783 | 5.7627 | 0.5389 | 0.9996 | 5'-nucleotidase [Source:UniProtKB/TrEMBL;Acc:Q17PQ5] |
| AAEL012793 | | 0.2504 | 3.1228 | 0.5389 | 0.9996 | |
| AAEL007900 | | -0.2195 | 5.5277 | 0.5389 | 0.9996 | |
| AAEL006035 | | -0.7242 | 6.5002 | 0.5390 | 0.9996 | |
| AAEL010504 | | -0.1844 | 6.0838 | 0.5390 | 0.9996 | |
| AAEL013225 | | 0.2829 | 8.2812 | 0.5392 | 0.9996 | NADH-cytochrome b5 reductase [Source:UniProtKB/TrEMBL;Acc:Q16JT1] |
| AAEL026995 | NA | 0.2539 | 4.0654 | 0.5392 | 0.9996 | NA |
| AAEL006644 | | 0.2141 | 3.5842 | 0.5392 | 0.9996 | |
| AAEL019561 | NA | 0.2885 | 4.5870 | 0.5394 | 0.9996 | NA |
| AAEL012463 | | -0.1690 | 4.7770 | 0.5396 | 0.9996 | |
| AAEL003003 | | -0.9176 | 6.0835 | 0.5397 | 0.9996 | glutamate-gated chloride channel [Source:VB Community Annotation] |
| AAEL013243 | | -0.1679 | 3.8801 | 0.5397 | 0.9996 | |
| AAEL001632 | | -0.8964 | -0.1495 | 0.5399 | 0.9996 | multicopper oxidase [Source:VB Community Annotation] |
| AAEL009603 | | -0.2237 | 4.8426 | 0.5399 | 0.9996 | tRNA (guanine(37)-N1)-methyltransferase [Source:UniProtKB/TrEMBL;Acc:A0A1S4FMY3] |
| AAEL019982 | NA | -0.4931 | 4.1652 | 0.5401 | 0.9996 | NA |
| AAEL010045 | | -0.8260 | -1.6330 | 0.5403 | 0.9996 | Protein FAM50 homolog [Source:UniProtKB/Swiss-Prot;Acc:Q16U25] |
| AAEL006432 | | 0.8650 | -0.7489 | 0.5405 | 0.9996 | sugar transporter [Source:VB Community Annotation] |

| | | | | | | |
|---|---|---|---|---|---|---|
| AAEL001898 | | -0.2708 | 5.3365 | 0.5405 | 0.9996 | |
| AAEL006611 | | -0.1310 | 6.6995 | 0.5407 | 0.9996 | ER membrane protein complex subunit 3 [Source:UniProtKB/TrEMBL;Acc:Q175K6] |
| AAEL014372 | | -0.3361 | 4.3271 | 0.5407 | 0.9996 | juvenile hormone-inducible protein, putative [Source:VB Community Annotation] |
| AAEL010728 | | 0.2188 | 2.6084 | 0.5407 | 0.9996 | sphingosine-1-phosphate phosphohydrolase [Source:VB Community Annotation] |
| AAEL009398 | | 0.3227 | 3.5984 | 0.5408 | 0.9996 | Pep12p, putative [Source:VB Community Annotation] |
| AAEL004725 | | 0.1973 | 5.8337 | 0.5408 | 0.9996 | |
| AAEL010411 | | 0.3028 | 3.9244 | 0.5409 | 0.9996 | dual specificity protein phosphatase [Source:VB Community Annotation] |
| AAEL000749 | | -1.1049 | 7.8541 | 0.5409 | 0.9996 | |
| AAEL002769 | | -0.6390 | 8.7816 | 0.5410 | 0.9996 | homeobox protein prospero/prox-1 [Source:VB Community Annotation] |
| AAEL012684 | | 0.2227 | 7.0413 | 0.5413 | 0.9996 | |
| AAEL013761 | | 0.3340 | 5.0592 | 0.5416 | 0.9996 | ADP-ribosylation factor, arf [Source:VB Community Annotation] |
| AAEL006107 | | 0.3578 | 1.4043 | 0.5416 | 0.9996 | mitotic and DNA damage checkpoint protein hus1 [Source:VB Community Annotation] |
| AAEL024100 | NA | 0.2827 | 2.2401 | 0.5418 | 0.9996 | NA |
| AAEL000204 | | 0.6678 | 1.7233 | 0.5418 | 0.9996 | |
| AAEL007067 | | -0.1680 | 5.3852 | 0.5418 | 0.9996 | WD-repeat protein [Source:VB Community Annotation] |
| AAEL002011 | | -0.7109 | 1.5971 | 0.5420 | 0.9996 | |
| AAEL000744 | | 0.4729 | 5.1617 | 0.5420 | 0.9996 | troponin C [Source:VB Community Annotation] |
| AAEL004620 | | 0.3003 | 6.4174 | 0.5421 | 0.9996 | sorting nexin [Source:VB Community Annotation] |
| AAEL005473 | | -1.3218 | 0.2655 | 0.5421 | 0.9996 | |
| AAEL008393 | | 0.1915 | 5.4070 | 0.5421 | 0.9996 | phosphatidylserine synthase [Source:VB Community Annotation] |
| AAEL010640 | | 0.8755 | 4.2426 | 0.5421 | 0.9996 | phosphoribosylamine-glycine ligase [Source:VB Community Annotation] |
| AAEL011329 | | -0.2773 | 2.4729 | 0.5422 | 0.9996 | |
| AAEL021376 | NA | -0.2504 | 3.3505 | 0.5423 | 0.9996 | NA |
| AAEL010162 | | 0.1823 | 3.8100 | 0.5424 | 0.9996 | |
| AAEL025477 | NA | 0.1975 | 5.8951 | 0.5425 | 0.9996 | NA |
| AAEL022146 | NA | -0.5091 | 4.7187 | 0.5425 | 0.9996 | NA |
| AAEL011273 | ucdk1 | 0.1639 | 4.6366 | 0.5425 | 0.9996 | uridine cytidine kinase i [Source:VB Community Annotation] |
| AAEL018234 | | -1.0578 | -0.6164 | 0.5425 | 0.9996 | |
| AAEL003511 | | -0.7242 | -0.3632 | 0.5426 | 0.9996 | |
| AAEL022895 | NA | 0.5833 | -0.0927 | 0.5426 | 0.9996 | NA |
| AAEL003407 | | -0.4123 | 5.4854 | 0.5426 | 0.9996 | |
| AAEL023647 | NA | -0.6706 | 1.2668 | 0.5428 | 0.9996 | NA |
| AAEL002157 | | 0.2144 | 4.1165 | 0.5429 | 0.9996 | |
| AAEL010440 | | 0.2278 | 3.5776 | 0.5430 | 0.9996 | |
| AAEL012460 | | 0.2290 | 2.7824 | 0.5431 | 0.9996 | |
| AAEL002098 | | -0.2541 | 2.2304 | 0.5432 | 0.9996 | DNA repair endonuclease xp-f / mei-9 / rad1 [Source:VB Community Annotation] |
| AAEL010829 | | 0.4788 | -0.1041 | 0.5432 | 0.9996 | protein arginine n-methyltransferase [Source:VB Community Annotation] |
| AAEL016982 | | 0.2535 | 7.7318 | 0.5433 | 0.9996 | |
| AAEL013170 | | 0.3832 | 4.4305 | 0.5433 | 0.9996 | phosphatidylcholine-sterol acyltransferase (lecithin-cholesterol acyltransferase) [Source:VB Community Annotation] |
| AAEL002184 | | 0.5999 | 3.9750 | 0.5435 | 0.9996 | F-actin capping protein beta subunit [Source:VB Community Annotation] |

| | | | | | | |
|---|---|---|---|---|---|---|
| AAEL024475 | NA | 0.2774 | 3.4150 | 0.5436 | 0.9996 | NA |
| AAEL026095 | NA | 0.4029 | 2.3059 | 0.5436 | 0.9996 | NA |
| AAEL000878 | | -1.1771 | -0.6326 | 0.5437 | 0.9996 | cuticle protein, putative [Source:VB Community Annotation] |
| AAEL002917 | | 0.2028 | 6.8066 | 0.5438 | 0.9996 | |
| AAEL000561 | | 0.2433 | 4.5511 | 0.5438 | 0.9996 | |
| AAEL010322 | | 0.3633 | 1.4623 | 0.5438 | 0.9996 | hemoglobin (heterodimeric), putative [Source:VB Community Annotation] |
| AAEL013770 | | 1.3884 | 1.7643 | 0.5440 | 0.9996 | zinc finger protein [Source:VB Community Annotation] |
| AAEL019989 | NA | 1.0647 | 0.7691 | 0.5440 | 0.9996 | NA |
| AAEL010343 | | -0.6586 | 4.3845 | 0.5443 | 0.9996 | aryl hydrocarbon receptor nuclear translocator (arnt protein) (hypoxia-inducible factor 1 beta) [Source:VB Community Annotation] |
| AAEL003474 | | 0.4887 | 1.7240 | 0.5444 | 0.9996 | |
| AAEL008607 | | -0.2957 | 9.0204 | 0.5445 | 0.9996 | tep3 [Source:VB Community Annotation] |
| AAEL013615 | | 0.2403 | 4.5855 | 0.5447 | 0.9996 | |
| AAEL027808 | NA | 0.4129 | 2.5362 | 0.5449 | 0.9996 | NA |
| AAEL006279 | | -0.3154 | 1.9519 | 0.5450 | 0.9996 | |
| AAEL027364 | NA | -0.7041 | -1.5115 | 0.5450 | 0.9996 | NA |
| AAEL018249 | | -0.6516 | 0.7643 | 0.5451 | 0.9996 | Aminopeptidase [Source:UniProtKB/TrEMBL;Acc:A0A1S4G775] |
| AAEL020018 | NA | -0.5059 | 5.2748 | 0.5451 | 0.9996 | NA |
| AAEL004867 | | -0.3807 | 1.8152 | 0.5451 | 0.9996 | NBP2b protein, putative [Source:VB Community Annotation] |
| AAEL020765 | NA | 0.7088 | -0.4733 | 0.5452 | 0.9996 | NA |
| AAEL019701 | NA | -0.9700 | 1.3422 | 0.5452 | 0.9996 | NA |
| AAEL029007 | NA | -0.4119 | 7.6695 | 0.5453 | 0.9996 | NA |
| AAEL008406 | | 0.2523 | 2.9315 | 0.5454 | 0.9996 | cationic amino acid transporter [Source:VB Community Annotation] |
| AAEL022743 | NA | -0.4203 | 2.4195 | 0.5455 | 0.9996 | NA |
| AAEL007234 | | -0.7588 | 3.0685 | 0.5456 | 0.9996 | |
| AAEL021440 | NA | -0.4635 | 4.4752 | 0.5456 | 0.9996 | NA |
| AAEL000275 | | 0.3805 | 5.9028 | 0.5457 | 0.9996 | |
| AAEL003492 | | -0.1474 | 7.2644 | 0.5458 | 0.9996 | ribonuclease, putative [Source:VB Community Annotation] |
| AAEL002474 | | -0.5003 | 3.9321 | 0.5460 | 0.9996 | |
| AAEL010712 | | -0.2916 | 8.2212 | 0.5462 | 0.9996 | low-density lipoprotein receptor (ldl) [Source:VB Community Annotation] |
| AAEL009199 | | -0.5698 | 0.4390 | 0.5462 | 0.9996 | core 1 UDP-galactose:n-acetylgalactosamine-alpha-r beta 1,3- galactosyltransferase [Source:VB Community Annotation] |
| AAEL000886 | | -0.4843 | 8.6263 | 0.5463 | 0.9996 | |
| AAEL025665 | NA | -1.0255 | 3.4667 | 0.5465 | 0.9996 | NA |
| AAEL019756 | NA | -0.2120 | 3.0866 | 0.5465 | 0.9996 | NA |
| AAEL022339 | NA | -0.2460 | 3.6361 | 0.5465 | 0.9996 | NA |
| AAEL012377 | OBP55 | -0.3483 | 9.9424 | 0.5465 | 0.9996 | odorant binding protein OBP55 [Source:VB Community Annotation] |
| AAEL020035 | NA | -1.2982 | 0.7280 | 0.5466 | 0.9996 | NA |
| AAEL012062 | | -0.3047 | 10.6609 | 0.5466 | 0.9996 | Na+/K+ ATPase alpha subunit [Source:VB Community Annotation] |
| AAEL008471 | | -0.2739 | 5.6367 | 0.5466 | 0.9996 | |
| AAEL009773 | | 0.5883 | 0.3938 | 0.5467 | 0.9996 | geminin, putative [Source:VB Community Annotation] |
| AAEL000335 | | -0.8188 | -0.3869 | 0.5468 | 0.9996 | lamin [Source:VB Community Annotation] |

| | | | | | | |
|---|---|---|---|---|---|---|
| AAEL007559 | | 0.2259 | 2.7992 | 0.5470 | 0.9996 | guanyl-nucleotide exchange factor, putative [Source:VB Community Annotation] |
| AAEL000139 | OBP5 | -0.3591 | 2.5919 | 0.5470 | 0.9996 | odorant binding protein OBP5 [Source:VB Community Annotation] |
| AAEL007983 | | 0.2213 | 2.3868 | 0.5470 | 0.9996 | |
| AAEL006963 | | 0.2211 | 3.4160 | 0.5471 | 0.9996 | |
| AAEL001838 | | 0.1719 | 6.3167 | 0.5471 | 0.9996 | |
| AAEL014847 | inx2 | -0.6241 | 4.5966 | 0.5475 | 0.9996 | Innexin [Source:UniProtKB/TrEMBL;Acc:Q16FA2] |
| AAEL022001 | NA | -0.6303 | 0.0643 | 0.5476 | 0.9996 | NA |
| AAEL007292 | | -0.2417 | 5.5924 | 0.5476 | 0.9996 | |
| AAEL019682 | NA | -0.6408 | 7.2626 | 0.5476 | 0.9996 | NA |
| AAEL002562 | | 0.1585 | 4.9364 | 0.5480 | 0.9996 | |
| AAEL010473 | | 0.2990 | 4.8890 | 0.5481 | 0.9996 | NAD dependent epimerase/dehydratase [Source:VB Community Annotation] |
| AAEL009171 | | 0.2828 | 5.0568 | 0.5482 | 0.9996 | |
| AAEL005074 | | -0.2164 | 1.9058 | 0.5483 | 0.9996 | |
| AAEL018348 | | -0.1977 | 5.8727 | 0.5483 | 0.9996 | |
| AAEL026829 | NA | 0.6388 | 0.0492 | 0.5483 | 0.9996 | NA |
| AAEL008262 | | -0.2504 | 4.1434 | 0.5483 | 0.9996 | ppa (fragment) [Source:VB Community Annotation] |
| AAEL009088 | | -0.6361 | 6.4147 | 0.5483 | 0.9996 | liquid facets [Source:VB Community Annotation] |
| AAEL012864 | | -0.5278 | 1.0831 | 0.5484 | 0.9996 | |
| AAEL004837 | | 0.2105 | 3.3970 | 0.5484 | 0.9996 | |
| AAEL005840 | | -0.2436 | 2.7403 | 0.5485 | 0.9996 | |
| AAEL010142 | | 0.2203 | 4.7716 | 0.5485 | 0.9996 | |
| AAEL029012 | NA | -0.2156 | 4.6666 | 0.5485 | 0.9996 | NA |
| AAEL027069 | NA | 0.2703 | 2.6148 | 0.5486 | 0.9996 | NA |
| AAEL026220 | NA | 0.2185 | 4.8429 | 0.5486 | 0.9996 | NA |
| AAEL005409 | | -0.9241 | -0.9834 | 0.5487 | 0.9996 | regulator of g protein signaling [Source:VB Community Annotation] |
| AAEL019830 | NA | -1.0225 | 2.5587 | 0.5488 | 0.9996 | NA |
| AAEL014063 | | -0.5675 | 1.0996 | 0.5488 | 0.9996 | activating signal cointegrator 1 complex subunit 3, helc1 [Source:VB Community Annotation] |
| AAEL008428 | | 1.5570 | 1.5896 | 0.5489 | 0.9996 | |
| AAEL000091 | | 0.2283 | 3.7739 | 0.5491 | 0.9996 | sumo-1-activating enzyme E1a [Source:VB Community Annotation] |
| AAEL004967 | | 0.2552 | 4.1808 | 0.5491 | 0.9996 | myo inositol monophosphatase [Source:VB Community Annotation] |
| AAEL009892 | | 0.1639 | 4.6264 | 0.5491 | 0.9996 | |
| AAEL011700 | | 0.4256 | 2.3716 | 0.5491 | 0.9996 | phosphatidylinositol glycan, class c [Source:VB Community Annotation] |
| AAEL007931 | | 0.2564 | 3.1103 | 0.5491 | 0.9996 | P21-activated kinase, pak [Source:VB Community Annotation] |
| AAEL013069 | Rack1 | 0.1731 | 11.0797 | 0.5494 | 0.9996 | guanine nucleotide-binding protein subunit beta-like protein [Source:VB Community Annotation] |
| AAEL004002 | | 0.4578 | 2.8050 | 0.5495 | 0.9996 | glucose dehydrogenase [Source:VB Community Annotation] |
| AAEL025533 | NA | 0.2469 | 4.0049 | 0.5496 | 0.9996 | NA |
| AAEL010416 | | 0.9583 | 0.9754 | 0.5496 | 0.9996 | |
| AAEL014426 | | 0.5011 | 3.1774 | 0.5497 | 0.9996 | glycine dehydrogenase [Source:VB Community Annotation] |
| AAEL023874 | NA | -0.5541 | 0.3031 | 0.5497 | 0.9996 | NA |
| AAEL003345 | | -0.4302 | 8.3171 | 0.5498 | 0.9996 | argininosuccinate lyase [Source:VB Community Annotation] |

| | | | | | | |
|---|---|---|---|---|---|---|
| AAEL012349 | | 0.3850 | 4.6349 | 0.5499 | 0.9996 | lipase 1 precursor [Source:VB Community Annotation] |
| AAEL026058 | NA | -0.5686 | 0.1171 | 0.5500 | 0.9996 | NA |
| AAEL007999 | | -0.6342 | 0.6098 | 0.5500 | 0.9996 | |
| AAEL024411 | NA | 0.2176 | 3.1023 | 0.5500 | 0.9996 | NA |
| AAEL010538 | | -0.4269 | 5.9319 | 0.5501 | 0.9996 | |
| AAEL008826 | | -0.2465 | 6.4943 | 0.5501 | 0.9996 | |
| AAEL017505 | Or103 | 0.4555 | 0.4354 | 0.5502 | 0.9996 | Odorant receptor [Source:UniProtKB/TrEMBL;Acc:J9HGV1] |
| AAEL001123 | | 0.2702 | 2.5712 | 0.5503 | 0.9996 | |
| AAEL003727 | | 0.5004 | 0.3653 | 0.5504 | 0.9996 | ninjurin a [Source:VB Community Annotation] |
| AAEL021718 | NA | -0.9880 | -1.5717 | 0.5505 | 0.9996 | NA |
| AAEL010932 | | 0.4845 | 2.6826 | 0.5507 | 0.9996 | RNAse h [Source:VB Community Annotation] |
| AAEL021562 | NA | -0.5198 | -0.3299 | 0.5508 | 0.9996 | NA |
| AAEL022609 | NA | 0.2185 | 7.0651 | 0.5508 | 0.9996 | NA |
| AAEL002449 | | -0.4955 | 5.5487 | 0.5510 | 0.9996 | |
| AAEL012298 | | -0.1808 | 5.2966 | 0.5512 | 0.9996 | sterol desaturase [Source:VB Community Annotation] |
| AAEL008521 | | -0.1978 | 6.6758 | 0.5512 | 0.9996 | |
| AAEL012636 | | 0.6344 | -0.5586 | 0.5512 | 0.9996 | cytochrome b5, putative [Source:VB Community Annotation] |
| AAEL007385 | | 0.1850 | 10.8246 | 0.5512 | 0.9996 | Ferritin subunit Precursor (EC 1.16.3.1)(Ferritin heavy chain-like protein)(AeFer(H)) [Source:VB Community Annotation] |
| AAEL010939 | | 0.3856 | 3.3552 | 0.5512 | 0.9996 | group ii plp decarboxylase [Source:VB Community Annotation] |
| AAEL007854 | | 0.2078 | 4.5907 | 0.5512 | 0.9996 | pelota [Source:VB Community Annotation] |
| AAEL004702 | | -0.1610 | 5.7209 | 0.5513 | 0.9996 | |
| AAEL001071 | GSTD5 | -0.4151 | 1.0444 | 0.5513 | 0.9996 | glutathione S-transferase (GSTD5) [Source:VB Community Annotation] |
| AAEL008322 | GPRFZ4 | -0.8011 | 0.3710 | 0.5515 | 0.9996 | GPCR Frizzled/Smoothened Family [Source:VB Community Annotation] |
| AAEL014533 | | 0.2566 | 2.7198 | 0.5515 | 0.9996 | |
| AAEL026967 | NA | 0.9019 | 6.0487 | 0.5517 | 0.9996 | NA |
| AAEL000571 | | 0.2398 | 4.2971 | 0.5517 | 0.9996 | |
| AAEL009808 | | -0.2639 | 6.9370 | 0.5518 | 0.9996 | ATP synthase subunit d [Source:VB Community Annotation] |
| AAEL002367 | CCEAE1B | 0.2427 | 3.8652 | 0.5519 | 0.9996 | Carboxy/choline esterase Alpha Esterase [Source:VB Community Annotation] |
| AAEL019547 | NA | -0.4079 | 0.9199 | 0.5520 | 0.9996 | NA |
| AAEL013484 | | 0.3894 | 8.0300 | 0.5520 | 0.9996 | |
| AAEL002571 | | -0.2558 | 2.8692 | 0.5520 | 0.9996 | band 4.1-like protein 4A (NBL4 protein), putative [Source:VB Community Annotation] |
| AAEL027453 | NA | 0.2403 | 8.2447 | 0.5522 | 0.9996 | NA |
| AAEL008055 | | -0.9522 | 0.3629 | 0.5522 | 0.9996 | nicotinic acetylcholine receptor subunit [Source:VB Community Annotation] |
| AAEL000324 | | -0.9137 | -0.4268 | 0.5523 | 0.9996 | tyrosine-protein kinase drl [Source:VB Community Annotation] |
| AAEL001336 | | 0.2701 | 5.5027 | 0.5525 | 0.9996 | charged multivesicular body protein 2a [Source:VB Community Annotation] |
| AAEL020471 | NA | -0.6936 | 1.5303 | 0.5525 | 0.9996 | NA |
| AAEL022313 | NA | 0.2730 | 5.0250 | 0.5526 | 0.9996 | NA |
| AAEL001894 | | -1.3316 | 1.2349 | 0.5527 | 0.9996 | kek1 [Source:VB Community Annotation] |
| AAEL000733 | | -1.1213 | 2.3464 | 0.5528 | 0.9996 | hydroxysteroid dehydrogenase [Source:VB Community Annotation] |
| AAEL006751 | | -0.2007 | 8.0584 | 0.5529 | 0.9996 | |

| | | | | | | |
|---|---|---|---|---|---|---|
| AAEL006479 | | 0.3275 | 3.7040 | 0.5529 | 0.9996 | |
| AAEL010522 | | 0.1821 | 4.0117 | 0.5529 | 0.9996 | |
| AAEL011014 | | 0.6608 | -0.8360 | 0.5529 | 0.9996 | |
| AAEL006976 | | -0.3386 | 6.6314 | 0.5529 | 0.9996 | Repressor of RNA polymerase III transcription MAF1 [Source:UniProtKB/TrEMBL;Acc:Q174A4] |
| AAEL007197 | | -0.1812 | 6.8010 | 0.5529 | 0.9996 | amino acid transporter [Source:VB Community Annotation] |
| AAEL000932 | | -0.5308 | 4.3248 | 0.5529 | 0.9996 | |
| AAEL004607 | | 0.1842 | 4.7067 | 0.5530 | 0.9996 | Adenylyltransferase and sulfurtransferase MOCS3 (Molybdenum cofactor synthesis protein 3) [Includes Adenylyltransferase MOCS3(EC 2 |
| AAEL014550 | | -1.2261 | 0.3163 | 0.5533 | 0.9996 | homeobox protein pknox1 [Source:VB Community Annotation] |
| AAEL004698 | | -0.3350 | 0.9274 | 0.5533 | 0.9996 | DNA primase large subunit [Source:UniProtKB/TrEMBL;Acc:Q0IFM2] |
| AAEL008227 | | -0.2392 | 5.9862 | 0.5533 | 0.9996 | short-chain dehydrogenase [Source:VB Community Annotation] |
| AAEL003651 | | 0.2337 | 3.1232 | 0.5534 | 0.9996 | |
| AAEL019754 | NA | -0.6513 | -0.4438 | 0.5535 | 0.9996 | NA |
| AAEL004235 | | -0.2080 | 7.0704 | 0.5535 | 0.9996 | kinesin-like protein Klp10A [Source:VB Community Annotation] |
| AAEL005316 | | -0.2233 | 3.5466 | 0.5535 | 0.9996 | |
| AAEL004773 | | -0.1492 | 4.2275 | 0.5537 | 0.9996 | leucine carboxyl methyltransferase [Source:VB Community Annotation] |
| AAEL023488 | NA | -0.4993 | 4.1772 | 0.5537 | 0.9996 | NA |
| AAEL026338 | NA | -0.9189 | 1.8441 | 0.5538 | 0.9996 | NA |
| AAEL000673 | | 0.2016 | 5.2793 | 0.5539 | 0.9996 | ATP-dependent (S)-NAD(P)H-hydrate dehydratase (EC 4.2.1.93) [Source:VB Community Annotation] |
| AAEL012853 | | -0.2539 | 6.2761 | 0.5540 | 0.9996 | |
| AAEL004544 | | -1.0962 | 0.2367 | 0.5541 | 0.9996 | proteophosphoglycan, putative [Source:VB Community Annotation] |
| AAEL017435 | | 0.2033 | 2.9648 | 0.5542 | 0.9996 | |
| AAEL008286 | | -0.5073 | 1.6985 | 0.5543 | 0.9996 | |
| AAEL006883 | | 0.2867 | 3.8499 | 0.5545 | 0.9996 | |
| AAEL012792 | | -0.6157 | 1.4564 | 0.5546 | 0.9996 | |
| AAEL006977 | | -0.1995 | 5.9770 | 0.5547 | 0.9996 | serine/threonine protein phosphatase 2a regulatory subunit a [Source:VB Community Annotation] |
| AAEL022104 | NA | -0.5001 | 3.2499 | 0.5547 | 0.9996 | NA |
| AAEL006201 | | -0.1811 | 3.8932 | 0.5548 | 0.9996 | |
| AAEL010206 | | -0.5035 | 5.8744 | 0.5548 | 0.9996 | xylulose kinase [Source:VB Community Annotation] |
| AAEL000172 | | -0.2677 | 5.3774 | 0.5549 | 0.9996 | |
| AAEL010659 | | 0.2650 | 7.8162 | 0.5551 | 0.9996 | lethal(2)essential for life protein, l2efl [Source:VB Community Annotation] |
| AAEL002091 | | 0.1876 | 3.9700 | 0.5551 | 0.9996 | |
| AAEL004437 | | 0.1830 | 4.5270 | 0.5551 | 0.9996 | dual-specificity protein phosphatase, putative [Source:VB Community Annotation] |
| AAEL022977 | NA | 0.9691 | -0.5432 | 0.5552 | 0.9996 | NA |
| AAEL021805 | NA | -0.7449 | 4.9607 | 0.5552 | 0.9996 | NA |
| AAEL020747 | NA | 0.3626 | 3.1186 | 0.5552 | 0.9996 | NA |
| AAEL001391 | | 0.4452 | 0.1676 | 0.5553 | 0.9996 | |
| AAEL028168 | NA | -0.2069 | 3.9134 | 0.5553 | 0.9996 | NA |
| AAEL027353 | NA | 0.3124 | 3.5888 | 0.5553 | 0.9996 | NA |
| AAEL001905 | | -0.2117 | 4.3930 | 0.5554 | 0.9996 | |
| AAEL004892 | | 0.1745 | 4.3001 | 0.5555 | 0.9996 | |

| | | | | | | |
|---|---|---|---|---|---|---|
| AAEL021386 | NA | -0.1996 | 5.9196 | 0.5556 | 0.9996 | NA |
| AAEL008764 | | 0.4086 | 6.2701 | 0.5557 | 0.9996 | cuticle protein, putative [Source:VB Community Annotation] |
| AAEL013324 | | 0.6012 | 3.5745 | 0.5557 | 0.9996 | high affinity nuclear juvenile hormone binding protein, putative [Source:VB Community Annotation] |
| AAEL015493 | | -0.4512 | 5.0809 | 0.5557 | 0.9996 | |
| AAEL007760 | | -0.1785 | 4.7697 | 0.5557 | 0.9996 | mct-1 protein [Source:VB Community Annotation] |
| AAEL009766 | | 0.2857 | 4.5455 | 0.5559 | 0.9996 | lipoamide acyltransferase component of branched-chain alpha-keto acid dehydrogenase [Source:VB Community Annotation] |
| AAEL001855 | | -0.3412 | 1.6728 | 0.5560 | 0.9996 | zinc carboxypeptidase [Source:VB Community Annotation] |
| AAEL014244 | | -0.5511 | -0.0073 | 0.5563 | 0.9996 | glucosyl/glucuronosyl transferases [Source:VB Community Annotation] |
| AAEL019696 | NA | 0.1912 | 4.4508 | 0.5563 | 0.9996 | NA |
| AAEL018160 | | 0.1835 | 4.1760 | 0.5563 | 0.9996 | |
| AAEL020983 | NA | 0.2746 | 1.5115 | 0.5564 | 0.9996 | NA |
| AAEL019718 | NA | 0.3322 | 7.6493 | 0.5565 | 0.9996 | NA |
| AAEL011873 | | -0.1284 | 6.9153 | 0.5566 | 0.9996 | ubiquitin-conjugating enzyme [Source:VB Community Annotation] |
| AAEL004680 | | 0.4152 | 3.7226 | 0.5566 | 0.9996 | nuclear lamin L1 alpha, putative [Source:VB Community Annotation] |
| AAEL001141 | | -0.2406 | 4.3664 | 0.5567 | 0.9996 | transcriptional regulator ATRX (X-linked helicase II) [Source:VB Community Annotation] |
| AAEL013471 | | 0.5868 | 0.0364 | 0.5567 | 0.9996 | |
| AAEL007423 | | 0.2328 | 3.1476 | 0.5568 | 0.9996 | |
| AAEL009936 | | -0.2297 | 4.7403 | 0.5569 | 0.9996 | |
| AAEL017976 | HSP70Bb | 0.6632 | 0.1584 | 0.5572 | 0.9996 | heat shock protein HSP70 [Source:VB Community Annotation] |
| AAEL004799 | | -0.9125 | 0.8764 | 0.5572 | 0.9996 | |
| AAEL023441 | NA | 0.5257 | 2.9474 | 0.5574 | 0.9996 | NA |
| AAEL004016 | | -0.5791 | -0.4135 | 0.5574 | 0.9996 | |
| AAEL010754 | | -0.2314 | 11.2902 | 0.5575 | 0.9996 | |
| AAEL020872 | NA | 0.3452 | 2.3324 | 0.5577 | 0.9996 | NA |
| AAEL005904 | | 0.2787 | 4.5732 | 0.5578 | 0.9996 | |
| AAEL009682 | | 0.6891 | 0.5033 | 0.5578 | 0.9996 | serine collagenase 1 precursor, putative [Source:VB Community Annotation] |
| AAEL019898 | NA | -0.1459 | 6.5794 | 0.5579 | 0.9996 | NA |
| AAEL007626 | GNBPA1 | -0.3527 | 5.6984 | 0.5580 | 0.9996 | Gram-Negative Binding Protein (GNBP) or Beta-1 3-Glucan Binding Protein (BGBP). [Source:VB Community Annotation] |
| AAEL001184 | | 0.3195 | 2.0156 | 0.5580 | 0.9996 | |
| AAEL019706 | NA | -1.0907 | 2.4199 | 0.5582 | 0.9996 | NA |
| AAEL010375 | | -0.3341 | 1.6979 | 0.5583 | 0.9996 | |
| AAEL000268 | | 0.5079 | 0.2639 | 0.5585 | 0.9996 | lipase [Source:VB Community Annotation] |
| AAEL023769 | NA | -0.1776 | 7.0529 | 0.5585 | 0.9996 | NA |
| AAEL001036 | | 0.2477 | 3.3462 | 0.5587 | 0.9996 | ikappab kinase complex-associated protein [Source:VB Community Annotation] |
| AAEL010784 | | 0.1906 | 4.3707 | 0.5587 | 0.9996 | |
| AAEL010770 | | -0.2781 | 2.0520 | 0.5588 | 0.9996 | |
| AAEL007907 | | -0.8180 | -1.3556 | 0.5588 | 0.9996 | serine/threonine protein kinase [Source:VB Community Annotation] |
| AAEL020796 | NA | -0.3128 | 1.2709 | 0.5589 | 0.9996 | NA |
| AAEL012425 | | 0.3586 | 5.4451 | 0.5590 | 0.9996 | sterol desaturase [Source:VB Community Annotation] |
| AAEL024021 | NA | -0.3243 | 2.4383 | 0.5590 | 0.9996 | NA |

| | | | | | | |
|---|---|---|---|---|---|---|
| AAEL010059 | | 0.1712 | 7.6875 | 0.5591 | 0.9996 | bacterial-type ABC transport ATP-binding subunit? or RNAse l inhibitor [Source:VB Community Annotation] |
| AAEL012522 | | 0.2477 | 3.1461 | 0.5592 | 0.9996 | sodium-dependent phosphate transporter [Source:VB Community Annotation] |
| AAEL022611 | NA | -0.9702 | 4.1570 | 0.5593 | 0.9996 | NA |
| AAEL011652 | | -0.3825 | 3.1980 | 0.5594 | 0.9996 | |
| AAEL024700 | NA | -0.1863 | 3.6895 | 0.5594 | 0.9996 | NA |
| AAEL001361 | | -0.2098 | 3.4721 | 0.5596 | 0.9996 | brahma associated protein 170kD, putative [Source:VB Community Annotation] |
| AAEL003971 | | -0.3851 | 1.9815 | 0.5598 | 0.9996 | |
| AAEL000119 | | 0.3147 | 3.3197 | 0.5599 | 0.9996 | AMP dependent coa ligase [Source:VB Community Annotation] |
| AAEL007798 | CYP4K3 | -0.2648 | 2.2895 | 0.5602 | 0.9996 | cytochrome P450 [Source:VB Community Annotation] |
| AAEL004590 | | -0.7740 | -1.9184 | 0.5603 | 0.9996 | |
| AAEL000721 | | -0.6118 | 0.0973 | 0.5603 | 0.9996 | |
| AAEL024800 | NA | -0.5313 | 0.7909 | 0.5604 | 0.9996 | NA |
| AAEL009109 | | 0.2102 | 4.9405 | 0.5605 | 0.9996 | |
| AAEL009847 | | -0.7058 | 7.2743 | 0.5606 | 0.9996 | microtubule-associated protein [Source:VB Community Annotation] |
| AAEL012822 | | 0.3226 | 3.3274 | 0.5607 | 0.9996 | Misexpression suppressor of KSR, putative [Source:VB Community Annotation] |
| AAEL020316 | NA | 0.5691 | -0.6496 | 0.5610 | 0.9996 | NA |
| AAEL014238 | | -0.3664 | 3.8947 | 0.5610 | 0.9996 | aromatic amino acid decarboxylase [Source:VB Community Annotation] |
| AAEL013662 | | -0.3297 | 3.0310 | 0.5610 | 0.9996 | anterior fat body protein [Source:VB Community Annotation] |
| AAEL018281 | | 0.2062 | 4.4153 | 0.5610 | 0.9996 | |
| AAEL008540 | | -0.1719 | 2.7894 | 0.5610 | 0.9996 | |
| AAEL011375 | | -0.9627 | -0.4007 | 0.5611 | 0.9996 | trypsin, putative [Source:VB Community Annotation] |
| AAEL005857 | | -0.1643 | 4.3371 | 0.5613 | 0.9996 | |
| AAEL001008 | | 0.2151 | 2.6689 | 0.5614 | 0.9996 | |
| AAEL022402 | NA | -1.0794 | -0.1998 | 0.5614 | 0.9996 | NA |
| AAEL006845 | | -0.2460 | 4.0696 | 0.5615 | 0.9996 | |
| AAEL022009 | NA | -0.4693 | 0.6554 | 0.5616 | 0.9996 | NA |
| AAEL025921 | NA | 0.6161 | 3.1909 | 0.5616 | 0.9996 | NA |
| AAEL004227 | | 0.1779 | 6.1904 | 0.5617 | 0.9996 | myosin VI [Source:VB Community Annotation] |
| AAEL003627 | | -0.2145 | 3.9594 | 0.5619 | 0.9996 | sodium/chloride dependent amino acid transporter [Source:VB Community Annotation] |
| AAEL010765 | | -0.3043 | 7.9468 | 0.5619 | 0.9996 | |
| AAEL024632 | NA | -0.3852 | 7.6791 | 0.5619 | 0.9996 | NA |
| AAEL014451 | | -0.5439 | 0.9206 | 0.5619 | 0.9996 | |
| AAEL003789 | | -0.2327 | 4.7140 | 0.5620 | 0.9996 | exportin, putative [Source:VB Community Annotation] |
| AAEL014203 | | 0.1962 | 3.1068 | 0.5621 | 0.9996 | nervy [Source:VB Community Annotation] |
| AAEL003060 | | 0.8969 | 9.0663 | 0.5621 | 0.9996 | female-specific chymotrypsin [Source:VB Community Annotation] |
| AAEL021292 | NA | 0.5567 | 2.7991 | 0.5624 | 0.9996 | NA |
| AAEL000549 | | 0.3371 | 3.2980 | 0.5624 | 0.9996 | fibulin 1 and [Source:VB Community Annotation] |
| AAEL006339 | | -0.6671 | 0.6457 | 0.5625 | 0.9996 | peptidylglycine alpha-amidating monooxygenase COOH-terminal interactor protein-1 [Source:VB Community Annotation] |
| AAEL000996 | | 0.1965 | 3.8590 | 0.5626 | 0.9996 | |
| AAEL005303 | | -0.2287 | 4.9616 | 0.5628 | 0.9996 | beta-tubulin cofactor d [Source:VB Community Annotation] |

| | | | | | | |
|---|---|---|---|---|---|---|
| AAEL026154 | NA | -0.4499 | 2.8114 | 0.5628 | 0.9996 | NA |
| AAEL006178 | | 0.8295 | 0.2713 | 0.5629 | 0.9996 | |
| AAEL004591 | | 0.4608 | 1.0670 | 0.5630 | 0.9996 | |
| AAEL008739 | | 0.3016 | 4.3708 | 0.5631 | 0.9996 | shc transforming protein [Source:VB Community Annotation] |
| AAEL012066 | | -0.2864 | 2.0665 | 0.5633 | 0.9996 | |
| AAEL023694 | NA | -0.7501 | 3.8057 | 0.5633 | 0.9996 | NA |
| AAEL001595 | | 0.4211 | 0.8551 | 0.5634 | 0.9996 | |
| AAEL019907 | NA | -0.3797 | 3.3585 | 0.5635 | 0.9996 | NA |
| AAEL004984 | | -0.2485 | 4.4711 | 0.5637 | 0.9996 | cullin-associated NEDD8-dissociated protein 1 [Source:VB Community Annotation] |
| AAEL012041 | | -0.2625 | 3.0449 | 0.5637 | 0.9996 | sulphate transporter [Source:VB Community Annotation] |
| AAEL000985 | | -0.6741 | 1.9759 | 0.5637 | 0.9996 | |
| AAEL006406 | | -0.9172 | 8.1724 | 0.5637 | 0.9996 | |
| AAEL001446 | | 0.1844 | 4.6290 | 0.5641 | 0.9996 | testis-specific protein pbs13 (t-complex 11 ) [Source:VB Community Annotation] |
| AAEL012952 | | 0.2431 | 2.3273 | 0.5641 | 0.9996 | |
| AAEL006707 | | -0.1881 | 4.0314 | 0.5643 | 0.9996 | |
| AAEL001065 | | 0.3129 | 3.9679 | 0.5643 | 0.9996 | |
| AAEL008584 | | -0.2216 | 5.5280 | 0.5644 | 0.9996 | |
| AAEL014595 | | -0.3175 | 2.1413 | 0.5645 | 0.9996 | |
| AAEL026581 | NA | -1.1682 | 0.0297 | 0.5647 | 0.9996 | NA |
| AAEL012101 | | -0.5032 | 2.1725 | 0.5648 | 0.9996 | Tubulin alpha chain [Source:UniProtKB/TrEMBL;Acc:Q16N35] |
| AAEL006585 | | -0.4823 | 5.6516 | 0.5649 | 0.9996 | |
| AAEL008390 | | -0.7632 | 0.4164 | 0.5649 | 0.9996 | Guanylate cyclase [Source:UniProtKB/TrEMBL;Acc:Q16YZ6] |
| AAEL006582 | | 0.2822 | 10.3349 | 0.5651 | 0.9996 | calcium-transporting ATPase sarcoplasmic/endoplasmic reticulum type [Source:VB Community Annotation] |
| AAEL011869 | | -0.2271 | 3.2326 | 0.5651 | 0.9996 | |
| AAEL012726 | | 0.3304 | 4.7841 | 0.5651 | 0.9996 | |
| AAEL026668 | NA | -0.4134 | 3.6774 | 0.5652 | 0.9996 | NA |
| AAEL015355 | | -0.4705 | 1.2125 | 0.5654 | 0.9996 | |
| AAEL002792 | | 0.4198 | 0.7345 | 0.5654 | 0.9996 | dynein light chain [Source:VB Community Annotation] |
| AAEL010323 | | -0.2270 | 2.4166 | 0.5654 | 0.9996 | |
| AAEL010759 | | 0.2731 | 1.8324 | 0.5655 | 0.9996 | DNA repair protein rad9 [Source:VB Community Annotation] |
| AAEL011232 | | 0.2359 | 2.7756 | 0.5656 | 0.9996 | |
| AAEL022646 | NA | -0.7297 | 1.4912 | 0.5656 | 0.9996 | NA |
| AAEL012658 | | 0.2223 | 6.5940 | 0.5657 | 0.9996 | rgs-gaip interacting protein gipc [Source:VB Community Annotation] |
| AAEL012404 | | -0.7532 | 2.0710 | 0.5658 | 0.9996 | transcription factor sp8,sp9 [Source:VB Community Annotation] |
| AAEL014931 | | 0.6066 | 2.3382 | 0.5658 | 0.9996 | sarm1 [Source:VB Community Annotation] |
| AAEL005392 | | 0.2227 | 4.5107 | 0.5659 | 0.9996 | dihydropyridine-sensitive l-type calcium channel [Source:VB Community Annotation] |
| AAEL001725 | | 0.1225 | 4.9691 | 0.5660 | 0.9996 | |
| AAEL005666 | | -0.7457 | 4.9562 | 0.5661 | 0.9996 | matrix metalloproteinase [Source:VB Community Annotation] |
| AAEL025812 | NA | -0.6566 | -0.8528 | 0.5663 | 0.9996 | NA |
| AAEL007598 | | 0.4437 | 0.6725 | 0.5664 | 0.9996 | |

| | | | | | | |
|---|---|---|---|---|---|---|
| AAEL013715 | | 1.1258 | -0.2558 | 0.5664 | 0.9996 | trypsin [Source:VB Community Annotation] |
| AAEL003748 | CYP9AE1 | -0.6080 | -0.6708 | 0.5665 | 0.9996 | cytochrome P450 [Source:VB Community Annotation] |
| AAEL017391 | | -0.4301 | 7.3321 | 0.5666 | 0.9996 | |
| AAEL012202 | | 0.3020 | 2.0919 | 0.5666 | 0.9996 | transcription initiation factor IIE, alpha subunit [Source:VB Community Annotation] |
| AAEL012026 | | -0.3371 | 7.1287 | 0.5666 | 0.9996 | translation initiation factor 5C [Source:VB Community Annotation] |
| AAEL000948 | | -0.6232 | 2.1229 | 0.5666 | 0.9996 | |
| AAEL005220 | RpL30 | -0.1364 | 10.6122 | 0.5667 | 0.9996 | 60S ribosomal protein L30 [Source:UniProtKB/TrEMBL;Acc:Q1HR35] |
| AAEL023600 | NA | 0.8585 | -1.2932 | 0.5667 | 0.9996 | NA |
| AAEL009024 | GPRCAL3 | -0.2615 | 3.7578 | 0.5668 | 0.9996 | GPCR Calcitonin/Diuretic Hormone Family [Source:VB Community Annotation] |
| AAEL026410 | NA | -0.1859 | 4.2383 | 0.5670 | 0.9996 | NA |
| AAEL002234 | | -1.2972 | 1.7454 | 0.5673 | 0.9996 | |
| AAEL027111 | NA | 0.1932 | 5.3375 | 0.5673 | 0.9996 | NA |
| AAEL007547 | | 0.1437 | 4.9980 | 0.5673 | 0.9996 | Chloride channel protein [Source:UniProtKB/TrEMBL;Acc:Q171T6] |
| AAEL010249 | | -0.2441 | 2.0380 | 0.5674 | 0.9996 | |
| AAEL000066 | | -0.4462 | 0.3789 | 0.5674 | 0.9996 | |
| AAEL006880 | | -0.2203 | 5.6811 | 0.5675 | 0.9996 | rab32 [Source:VB Community Annotation] |
| AAEL023473 | NA | 0.7313 | 1.6063 | 0.5676 | 0.9996 | NA |
| AAEL023162 | NA | -0.3194 | 2.2055 | 0.5679 | 0.9996 | NA |
| AAEL000939 | | -0.7440 | -0.2838 | 0.5680 | 0.9996 | |
| AAEL014739 | | 0.1918 | 4.2925 | 0.5680 | 0.9996 | |
| AAEL002175 | | -0.9002 | 2.5789 | 0.5681 | 0.9996 | |
| AAEL001575 | | -0.7562 | -1.8587 | 0.5681 | 0.9996 | monocarboxylate transporter [Source:VB Community Annotation] |
| AAEL001109 | | -0.1933 | 6.4932 | 0.5681 | 0.9996 | glutaredoxin [Source:VB Community Annotation] |
| AAEL002658 | | 0.3741 | 5.6496 | 0.5683 | 0.9996 | AMP dependent ligase [Source:VB Community Annotation] |
| AAEL028066 | NA | 1.9474 | 0.5093 | 0.5683 | 0.9996 | NA |
| AAEL011960 | | -0.2288 | 4.3353 | 0.5686 | 0.9996 | |
| AAEL013433 | SPZ1C | 0.3794 | 1.9749 | 0.5686 | 0.9996 | spaetzle-like cytokine [Source:VB Community Annotation] |
| AAEL009777 | | -0.4560 | 2.9161 | 0.5686 | 0.9996 | heparan sulphate sulfotransferase [Source:VB Community Annotation] |
| AAEL005908 | | -0.2130 | 2.8156 | 0.5687 | 0.9996 | rolling pebbles [Source:VB Community Annotation] |
| AAEL003776 | | 0.2307 | 5.6587 | 0.5687 | 0.9996 | |
| AAEL023246 | NA | 0.6331 | -0.4270 | 0.5688 | 0.9996 | NA |
| AAEL019403 | NA | -0.2403 | 9.8345 | 0.5688 | 0.9996 | NA |
| AAEL001635 | | 0.4038 | 0.2754 | 0.5689 | 0.9996 | |
| AAEL010733 | | 0.2434 | 2.4247 | 0.5689 | 0.9996 | |
| AAEL011766 | | -0.4304 | 2.4852 | 0.5690 | 0.9996 | |
| AAEL008958 | | -0.2644 | 6.6292 | 0.5690 | 0.9996 | forkhead box protein (AaegFOXK1) [Source:VB Community Annotation] |
| AAEL004174 | | 0.3575 | 1.6992 | 0.5690 | 0.9996 | T-box transcription factor tbx6 [Source:VB Community Annotation] |
| AAEL000251 | | -0.6962 | 7.6366 | 0.5691 | 0.9996 | |
| AAEL004988 | Pgk | -0.2323 | 9.2081 | 0.5692 | 0.9996 | Phosphoglycerate kinase [Source:UniProtKB/TrEMBL;Acc:Q8WQL0] |
| AAEL006196 | | -0.1549 | 6.3841 | 0.5692 | 0.9996 | hemomucin [Source:VB Community Annotation] |

| | | | | | | |
|---|---|---|---|---|---|---|
| AAEL001245 | | -0.7356 | 5.3263 | 0.5693 | 0.9996 | heterogeneous nuclear ribonucleoprotein [Source:VB Community Annotation] |
| AAEL019603 | NA | 0.3456 | 4.3128 | 0.5693 | 0.9996 | NA |
| AAEL011109 | | -0.3471 | 4.5328 | 0.5695 | 0.9996 | |
| AAEL002520 | | 0.4600 | 3.5093 | 0.5696 | 0.9996 | apolipoprotein D, putative [Source:VB Community Annotation] |
| AAEL009430 | | -0.4693 | 7.6729 | 0.5697 | 0.9996 | |
| AAEL023511 | NA | -0.3115 | 3.8124 | 0.5698 | 0.9996 | NA |
| AAEL018153 | | -0.5600 | 4.7080 | 0.5701 | 0.9996 | |
| AAEL001972 | | -0.2109 | 6.2221 | 0.5702 | 0.9996 | TATA box binding protein (TBP)-associated factor,, putative [Source:VB Community Annotation] |
| AAEL008847 | | -0.1769 | 2.0370 | 0.5703 | 0.9996 | wingless [Source:VB Community Annotation] |
| AAEL000337 | | -0.2446 | 1.9496 | 0.5703 | 0.9996 | |
| AAEL023438 | NA | 0.8571 | -1.1346 | 0.5706 | 0.9996 | NA |
| AAEL000795 | | 0.1338 | 9.0009 | 0.5707 | 0.9996 | ubiquitin [Source:VB Community Annotation] |
| AAEL002198 | | 0.1129 | 5.7982 | 0.5707 | 0.9996 | |
| AAEL000478 | | 0.1387 | 4.5433 | 0.5709 | 0.9996 | Sm protein G, putative [Source:VB Community Annotation] |
| AAEL008763 | | -0.2126 | 5.1161 | 0.5709 | 0.9996 | tata-binding protein-associated phosphoprotein (dr1) [Source:VB Community Annotation] |
| AAEL026229 | NA | 0.2588 | 2.3610 | 0.5710 | 0.9996 | NA |
| AAEL011661 | | 0.6397 | 0.2350 | 0.5710 | 0.9996 | |
| AAEL019596 | NA | -0.4377 | 5.6126 | 0.5712 | 0.9996 | NA |
| AAEL012175 | | -0.2316 | 10.8813 | 0.5712 | 0.9996 | ATP synthase alpha subunit mitochondrial [Source:VB Community Annotation] |
| AAEL008106 | | -0.4116 | 9.2381 | 0.5713 | 0.9996 | |
| AAEL001394 | | -0.1384 | 5.7412 | 0.5713 | 0.9996 | |
| AAEL024713 | NA | -0.5716 | 3.2562 | 0.5715 | 0.9996 | NA |
| AAEL001960 | | -0.2004 | 5.6180 | 0.5716 | 0.9996 | cytochrome P450 [Source:VB Community Annotation] |
| AAEL023517 | NA | -0.4643 | 2.1171 | 0.5717 | 0.9996 | NA |
| AAEL006736 | | 0.3155 | 2.5810 | 0.5719 | 0.9996 | |
| AAEL027527 | NA | -0.7309 | -0.0043 | 0.5720 | 0.9996 | NA |
| AAEL006069 | | -0.6779 | 0.9901 | 0.5721 | 0.9996 | |
| AAEL006754 | | -0.2211 | 3.3479 | 0.5723 | 0.9996 | |
| AAEL009530 | | 0.2534 | 3.6065 | 0.5723 | 0.9996 | tmc6 protein (evin) [Source:VB Community Annotation] |
| AAEL008329 | RpL24 | 0.2412 | 11.4710 | 0.5723 | 0.9996 | 60S ribosomal protein L24 [Source:VB Community Annotation] |
| AAEL006434 | | -0.3693 | 6.2347 | 0.5724 | 0.9996 | serine protease, putative [Source:VB Community Annotation] |
| AAEL019447 | NA | -0.2351 | 4.2720 | 0.5724 | 0.9996 | NA |
| AAEL007095 | | 0.4813 | 0.3079 | 0.5725 | 0.9996 | adult cuticle protein, putative [Source:VB Community Annotation] |
| AAEL018318 | | 0.1543 | 4.4075 | 0.5727 | 0.9996 | E3 ubiquitin-protein ligase parkin [Source:UniProtKB/TrEMBL;Acc:A0A1S4G862] |
| AAEL012711 | CLIPC12 | -0.4803 | 1.0256 | 0.5728 | 0.9996 | Clip-Domain Serine Protease family C. [Source:VB Community Annotation] |
| AAEL019914 | NA | 0.1996 | 6.3580 | 0.5728 | 0.9996 | NA |
| AAEL009736 | | -0.5347 | 4.4980 | 0.5728 | 0.9996 | |
| AAEL006126 | | -1.2272 | -0.4997 | 0.5729 | 0.9996 | |
| AAEL003179 | | 0.1887 | 6.0948 | 0.5732 | 0.9996 | protein arginine n-methyltransferase 1, [Source:VB Community Annotation] |
| AAEL008133 | | 0.1890 | 4.6076 | 0.5732 | 0.9996 | GTP-binding protein hflx [Source:VB Community Annotation] |

| | | | | | | |
|---|---|---|---|---|---|---|
| AAEL023983 | NA | -0.4165 | 10.6290 | 0.5733 | 0.9996 | NA |
| AAEL020954 | NA | -1.0875 | 2.3446 | 0.5733 | 0.9996 | NA |
| AAEL003231 | | 0.5657 | -0.7756 | 0.5734 | 0.9996 | |
| AAEL014029 | | -0.1666 | 3.7935 | 0.5735 | 0.9996 | t1/st2 receptor binding protein [Source:VB Community Annotation] |
| AAEL012016 | | 0.1288 | 5.7567 | 0.5736 | 0.9996 | endomembrane protein emp70 [Source:VB Community Annotation] |
| AAEL008815 | | -0.2992 | 4.3423 | 0.5737 | 0.9996 | Fatty acyl-CoA reductase [Source:UniProtKB/TrEMBL;Acc:Q16XN0] |
| AAEL003427 | RpS16 | 0.2171 | 11.6089 | 0.5738 | 0.9996 | 40S ribosomal protein S16 [Source:UniProtKB/Swiss-Prot;Acc:P62251] |
| AAEL003128 | | -0.2951 | 5.8002 | 0.5740 | 0.9996 | |
| AAEL023695 | NA | -0.1516 | 4.6492 | 0.5740 | 0.9996 | NA |
| AAEL010369 | | 0.5055 | 0.3854 | 0.5741 | 0.9996 | phospholipase b, plb1 [Source:VB Community Annotation] |
| AAEL020846 | NA | 0.1882 | 5.3003 | 0.5741 | 0.9996 | NA |
| AAEL008376 | | -0.2560 | 2.1617 | 0.5741 | 0.9996 | |
| AAEL011556 | | 0.1784 | 4.5966 | 0.5742 | 0.9996 | |
| AAEL014334 | | -0.5783 | -0.0186 | 0.5743 | 0.9996 | lachesin, putative [Source:VB Community Annotation] |
| AAEL028224 | NA | 0.2606 | 2.3625 | 0.5743 | 0.9996 | NA |
| AAEL010867 | | -0.2928 | 6.8317 | 0.5745 | 0.9996 | serine protease [Source:VB Community Annotation] |
| AAEL007691 | | -0.3032 | 6.2396 | 0.5746 | 0.9996 | organic anion transporter [Source:VB Community Annotation] |
| AAEL027731 | NA | 0.1545 | 4.7484 | 0.5746 | 0.9996 | NA |
| AAEL004154 | | 0.2121 | 2.9627 | 0.5747 | 0.9996 | |
| AAEL011548 | | 0.2092 | 3.4034 | 0.5747 | 0.9996 | |
| AAEL012100 | | -0.3142 | 2.9346 | 0.5748 | 0.9996 | |
| AAEL007562 | | -0.2631 | 4.1995 | 0.5750 | 0.9996 | |
| AAEL001126 | | -0.1401 | 4.7894 | 0.5750 | 0.9996 | rest corepressor (corest) protein [Source:VB Community Annotation] |
| AAEL007586 | | -0.1505 | 5.6139 | 0.5751 | 0.9996 | nuclear pore complex protein nup98 [Source:VB Community Annotation] |
| AAEL011306 | | -0.2492 | 2.8876 | 0.5751 | 0.9996 | |
| AAEL013359 | | 0.1536 | 9.8634 | 0.5751 | 0.9996 | DEAD box ATP-dependent RNA helicase [Source:VB Community Annotation] |
| AAEL012439 | | -0.2929 | 5.5590 | 0.5753 | 0.9996 | enoyl-CoA hydratase (enoyl hydrase) (unsaturated acyl-CoA hydratase), putative [Source:VB Community Annotation] |
| AAEL017646 | U1 | 0.5952 | 0.9737 | 0.5757 | 0.9996 | U1 spliceosomal RNA [Source:RFAM;Acc:RF00003] |
| AAEL013139 | | -0.2048 | 8.8384 | 0.5757 | 0.9996 | GTPase_rho [Source:VB Community Annotation] |
| AAEL027990 | NA | 0.6747 | -0.7567 | 0.5758 | 0.9996 | NA |
| AAEL001607 | | -0.4701 | 7.1249 | 0.5758 | 0.9996 | Galactose-1-phosphate uridylyltransferase [Source:UniProtKB/TrEMBL;Acc:Q17KP5] |
| AAEL019694 | NA | 0.1671 | 5.5175 | 0.5759 | 0.9996 | NA |
| AAEL027007 | NA | 0.1623 | 4.6504 | 0.5760 | 0.9996 | NA |
| AAEL004137 | | -0.3365 | 2.7178 | 0.5761 | 0.9996 | acyl-coa dehydrogenase [Source:VB Community Annotation] |
| AAEL013727 | | 0.5738 | 2.1660 | 0.5762 | 0.9996 | |
| AAEL011786 | | 1.0464 | 0.5500 | 0.5762 | 0.9996 | |
| AAEL003670 | | -0.1617 | 6.6688 | 0.5762 | 0.9996 | myelinprotein expression factor [Source:VB Community Annotation] |
| AAEL013321 | | -0.1764 | 6.4815 | 0.5763 | 0.9996 | |
| AAEL003283 | | -0.5009 | 6.7787 | 0.5763 | 0.9996 | bitesize isoform [Source:VB Community Annotation] |
| AAEL019667 | NA | -1.0393 | -0.3553 | 0.5764 | 0.9996 | NA |

| | | | | | | |
|---|---|---|---|---|---|---|
| AAEL002934 | | -0.1408 | 3.2352 | 0.5764 | 0.9996 | |
| AAEL020093 | NA | 0.6519 | -0.6597 | 0.5765 | 0.9996 | NA |
| AAEL007658 | | -0.2101 | 4.1141 | 0.5765 | 0.9996 | partitioning defective 3, par-3 [Source:VB Community Annotation] |
| AAEL000249 | | 0.2100 | 5.1764 | 0.5766 | 0.9996 | mitochondrial carrier protein [Source:VB Community Annotation] |
| AAEL013783 | | -0.1684 | 6.5048 | 0.5766 | 0.9996 | protein farnesyltransferase alpha subunit [Source:VB Community Annotation] |
| AAEL007127 | | 0.2869 | 3.3047 | 0.5767 | 0.9996 | |
| AAEL001766 | | 0.2845 | 2.8503 | 0.5767 | 0.9996 | leucine-rich transmembrane proteins [Source:VB Community Annotation] |
| AAEL001649 | | 0.2359 | 5.4951 | 0.5768 | 0.9996 | leucine aminopeptidase [Source:VB Community Annotation] |
| AAEL009432 | SCRBQ3 | -0.8504 | 0.4804 | 0.5769 | 0.9996 | Class B Scavenger Receptor (CD36 domain). [Source:VB Community Annotation |
| AAEL003279 | | -0.3092 | 3.7958 | 0.5770 | 0.9996 | clip-domain serine protease, putative [Source:VB Community Annotation] |
| AAEL013969 | | -0.8803 | 4.3502 | 0.5771 | 0.9996 | Syndecan [Source:UniProtKB/TrEMBL;Acc:Q16HN0] |
| AAEL004711 | | 0.2825 | 2.2400 | 0.5772 | 0.9996 | testis specific leucine rich repeat protein [Source:VB Community Annotation] |
| AAEL023895 | NA | -0.4960 | 6.9083 | 0.5775 | 0.9996 | NA |
| AAEL011826 | | 0.2031 | 3.5813 | 0.5779 | 0.9996 | |
| AAEL007147 | | -0.3636 | 0.1847 | 0.5782 | 0.9996 | |
| AAEL004727 | | -0.2992 | 2.6746 | 0.5782 | 0.9996 | |
| AAEL010217 | ix | -0.2639 | 3.7795 | 0.5782 | 0.9996 | Mediator of RNA polymerase II transcription subunit 29 (Mediator complex subunit 29)(Protein intersex) [Source:VB Community Annotat |
| AAEL010785 | | 0.1949 | 2.4863 | 0.5783 | 0.9996 | |
| AAEL010180 | | -1.2274 | 3.5553 | 0.5784 | 0.9996 | |
| AAEL005308 | | -0.1670 | 6.2923 | 0.5785 | 0.9996 | Pyruvate dehydrogenase E1 component subunit alpha [Source:UniProtKB/TrEMBL;Acc:Q17AH1] |
| AAEL003412 | | -1.0896 | 2.7847 | 0.5786 | 0.9996 | nuclear lim interactor-interacting factor (nli-interacting factor) (nli-if) [Source:VB Community Annotation] |
| AAEL004046 | | -0.3938 | 0.6026 | 0.5787 | 0.9996 | |
| AAEL006156 | | 0.2687 | 2.3891 | 0.5789 | 0.9996 | |
| AAEL027459 | NA | 0.4923 | 1.4300 | 0.5790 | 0.9996 | NA |
| AAEL013565 | | -1.0682 | 1.7318 | 0.5790 | 0.9996 | |
| AAEL022195 | NA | 0.2151 | 2.8288 | 0.5791 | 0.9996 | NA |
| AAEL025656 | NA | 0.1906 | 4.5138 | 0.5791 | 0.9996 | NA |
| AAEL005400 | | -0.2303 | 7.6745 | 0.5792 | 0.9996 | 2-hydroxyacid dehydrogenase [Source:VB Community Annotation] |
| AAEL004361 | | 0.2497 | 3.0035 | 0.5792 | 0.9996 | alpha-glucosidase [Source:VB Community Annotation] |
| AAEL005238 | | -0.7416 | 3.9328 | 0.5792 | 0.9996 | mck1 [Source:VB Community Annotation] |
| AAEL012139 | | -0.1979 | 5.6891 | 0.5794 | 0.9996 | allatostatin, B-type, putative [Source:VB Community Annotation] |
| AAEL024342 | NA | -0.1727 | 3.5062 | 0.5795 | 0.9996 | NA |
| AAEL001503 | | -0.9551 | 1.6782 | 0.5795 | 0.9996 | sodium/hydrogen exchanger 3 (nhe3) [Source:VB Community Annotation] |
| AAEL024908 | NA | -0.2212 | 3.5999 | 0.5795 | 0.9996 | NA |
| AAEL004860 | | 0.2207 | 6.5256 | 0.5796 | 0.9996 | acireductone dioxygenase [Source:VB Community Annotation] |
| AAEL025744 | NA | -0.3833 | 4.2815 | 0.5796 | 0.9996 | NA |
| AAEL004497 | | 0.1648 | 6.4245 | 0.5799 | 0.9996 | |
| AAEL007988 | | -0.1361 | 6.2699 | 0.5799 | 0.9996 | |
| AAEL004631 | | 0.2795 | 7.8563 | 0.5799 | 0.9996 | actin [Source:VB Community Annotation] |
| AAEL026089 | NA | -0.2999 | 3.2940 | 0.5801 | 0.9996 | NA |

| | | | | | | |
|---|---|---|---|---|---|---|
| AAEL002752 | | 0.2422 | 2.4828 | 0.5801 | 0.9996 | anaphase-promoting complex, subunit-5, putative [Source:VB Community Annotation] |
| AAEL014816 | | -0.3606 | 4.8681 | 0.5805 | 0.9996 | |
| AAEL002879 | | -0.2496 | 9.3316 | 0.5806 | 0.9996 | heterogeneous nuclear ribonucleoprotein r [Source:VB Community Annotation] |
| AAEL004970 | | 0.6650 | 1.3768 | 0.5807 | 0.9996 | |
| AAEL009250 | | -0.2737 | 2.8117 | 0.5808 | 0.9996 | |
| AAEL025784 | NA | -0.7424 | 1.8764 | 0.5809 | 0.9996 | NA |
| AAEL008323 | | -0.1658 | 7.0705 | 0.5809 | 0.9996 | |
| AAEL006077 | | 0.8198 | -1.6666 | 0.5810 | 0.9996 | allatostatin receptor [Source:VB Community Annotation] |
| AAEL014649 | | -0.2314 | 3.9104 | 0.5810 | 0.9996 | |
| AAEL007105 | | -0.3135 | 3.3238 | 0.5811 | 0.9996 | |
| AAEL009904 | | -1.0962 | 0.7880 | 0.5813 | 0.9996 | |
| AAEL009017 | GSTT1 | -0.2225 | 4.6990 | 0.5813 | 0.9996 | glutathione transferase [Source:VB Community Annotation] |
| AAEL004439 | | 0.3205 | 2.7926 | 0.5813 | 0.9996 | |
| AAEL023629 | NA | -0.1822 | 7.1315 | 0.5814 | 0.9996 | NA |
| AAEL024028 | NA | -1.7927 | 1.8030 | 0.5815 | 0.9996 | NA |
| AAEL011093 | | 0.2270 | 3.3987 | 0.5815 | 0.9996 | homer [Source:VB Community Annotation] |
| AAEL001969 | | -0.2739 | 9.1107 | 0.5815 | 0.9996 | protein serine/threonine kinase, putative [Source:VB Community Annotation] |
| AAEL007452 | | -0.1543 | 7.4849 | 0.5816 | 0.9996 | |
| AAEL011928 | | -0.2576 | 7.5650 | 0.5816 | 0.9996 | Lipase maturation factor [Source:UniProtKB/TrEMBL;Acc:A0A1S4FUY8] |
| AAEL007597 | CLIPC3 | -0.1855 | 7.0993 | 0.5817 | 0.9996 | Clip-Domain Serine Protease family C [Source:VB Community Annotation] |
| AAEL012548 | | 0.2083 | 3.3083 | 0.5819 | 0.9996 | glycosylphosphatidylinositol-specific phospholipase C, putative [Source:VB Community Annotation] |
| AAEL020256 | NA | -1.0255 | 0.1923 | 0.5820 | 0.9996 | NA |
| AAEL000213 | | -0.7265 | 1.1752 | 0.5820 | 0.9996 | d-amino acid oxidase [Source:VB Community Annotation] |
| AAEL001321 | | 0.3143 | 2.0308 | 0.5822 | 0.9996 | transcription factor dp [Source:VB Community Annotation] |
| AAEL001815 | | -0.2483 | 4.2222 | 0.5823 | 0.9996 | |
| AAEL026057 | NA | -0.3828 | 4.8709 | 0.5825 | 0.9996 | NA |
| AAEL008874 | | -0.2621 | 3.9966 | 0.5826 | 0.9996 | |
| AAEL010242 | | -0.9505 | 7.6260 | 0.5827 | 0.9996 | |
| AAEL009837 | | -1.1940 | 0.4171 | 0.5828 | 0.9996 | |
| AAEL002951 | | 0.4059 | -0.1961 | 0.5829 | 0.9996 | forkhead protein/ forkhead protein domain [Source:VB Community Annotation] |
| AAEL012143 | CASPS7 | 0.6695 | -1.3434 | 0.5829 | 0.9996 | caspase (short) [Source:VB Community Annotation] |
| AAEL000288 | | -0.5891 | 5.1195 | 0.5831 | 0.9996 | |
| AAEL015202 | | 0.4070 | 6.1464 | 0.5831 | 0.9996 | |
| AAEL005234 | | -0.3174 | 0.9186 | 0.5831 | 0.9996 | |
| AAEL001751 | | -0.1690 | 4.8205 | 0.5831 | 0.9996 | cyclin [Source:VB Community Annotation] |
| AAEL009906 | | 0.2317 | 5.7788 | 0.5832 | 0.9996 | |
| AAEL006486 | | -0.3672 | 3.8770 | 0.5832 | 0.9996 | |
| AAEL021878 | NA | -0.5678 | -0.2158 | 0.5833 | 0.9996 | NA |
| AAEL008125 | | -0.3110 | 5.1442 | 0.5833 | 0.9996 | Fatty acyl-CoA reductase [Source:UniProtKB/TrEMBL;Acc:Q16ZP3] |
| AAEL027002 | NA | 0.2111 | 3.8492 | 0.5834 | 0.9996 | NA |

| | | | | | | |
|---|---|---|---|---|---|---|
| AAEL002004 | | -0.7153 | -0.4461 | 0.5834 | 0.9996 | protein serine/threonine kinase, putative [Source:VB Community Annotation] |
| AAEL020822 | NA | -0.2514 | 4.9699 | 0.5836 | 0.9996 | NA |
| AAEL005985 | | -0.2944 | 6.7508 | 0.5836 | 0.9996 | Autophagy-related protein 13 [Source:UniProtKB/TrEMBL;Acc:Q177Z0] |
| AAEL020104 | NA | 0.1710 | 3.3741 | 0.5837 | 0.9996 | NA |
| AAEL013749 | | -0.1471 | 5.6145 | 0.5838 | 0.9996 | |
| AAEL008609 | | 1.0096 | -0.4014 | 0.5839 | 0.9996 | zinc carboxypeptidase [Source:VB Community Annotation] |
| AAEL003234 | | -0.2346 | 11.5032 | 0.5840 | 0.9996 | cytochrome c oxidase, subunit VIA, putative [Source:VB Community Annotation] |
| AAEL002510 | | 0.4258 | 7.8536 | 0.5840 | 0.9996 | Serine hydroxymethyltransferase [Source:UniProtKB/TrEMBL;Acc:Q17I00] |
| AAEL008164 | | -0.6358 | 5.5189 | 0.5841 | 0.9996 | |
| AAEL020955 | NA | -0.2140 | 4.5541 | 0.5841 | 0.9996 | NA |
| AAEL020290 | NA | -0.1210 | 11.9044 | 0.5841 | 0.9996 | NA |
| AAEL007804 | | 0.8249 | 2.5985 | 0.5841 | 0.9996 | semaphorin [Source:VB Community Annotation] |
| AAEL012047 | | 0.1799 | 3.0279 | 0.5842 | 0.9996 | |
| AAEL016214 | tRNA-Lys | 0.3561 | 0.3441 | 0.5844 | 0.9996 | |
| AAEL001554 | | 0.1737 | 3.5084 | 0.5844 | 0.9996 | PHD finger protein [Source:VB Community Annotation] |
| AAEL008608 | | -0.2680 | 3.4809 | 0.5844 | 0.9996 | |
| AAEL028121 | NA | -0.9472 | -0.2166 | 0.5845 | 0.9996 | NA |
| AAEL004009 | | -0.3107 | 2.1427 | 0.5845 | 0.9996 | glucose dehydrogenase [Source:VB Community Annotation] |
| AAEL026000 | NA | -0.4247 | 0.4683 | 0.5845 | 0.9996 | NA |
| AAEL006957 | GPRNNB4 | -0.8464 | -1.3972 | 0.5846 | 0.9996 | GPCR Orphan/Putative Class B Family [Source:VB Community Annotation] |
| AAEL003768 | | 0.7178 | 0.4950 | 0.5846 | 0.9996 | |
| AAEL019507 | NA | -0.4660 | 5.7142 | 0.5846 | 0.9996 | NA |
| AAEL006731 | | 0.1561 | 4.5639 | 0.5846 | 0.9996 | |
| AAEL002427 | | 0.6159 | -0.9301 | 0.5846 | 0.9996 | transcription factor IIIB 90 kDa subunit (TFIIIB90) [Source:VB Community Annotation] |
| AAEL002748 | | 0.1995 | 5.7564 | 0.5849 | 0.9996 | aspartyl-tRNA synthetase [Source:VB Community Annotation] |
| AAEL001219 | | -0.6971 | -1.3523 | 0.5849 | 0.9996 | |
| AAEL005071 | | 0.1727 | 6.1382 | 0.5850 | 0.9996 | GTP binding protein [Source:VB Community Annotation] |
| AAEL010561 | | -0.2147 | 5.1917 | 0.5851 | 0.9996 | |
| AAEL024269 | NA | -1.2052 | -0.0323 | 0.5852 | 0.9996 | NA |
| AAEL012023 | | -0.1333 | 5.6590 | 0.5853 | 0.9996 | |
| AAEL026994 | NA | -0.8804 | 3.1018 | 0.5853 | 0.9996 | NA |
| AAEL002647 | | 0.4522 | 2.5581 | 0.5853 | 0.9996 | steroid receptor-interacting snf2 domain protein [Source:VB Community Annotation] |
| AAEL025559 | NA | -0.5311 | -2.1422 | 0.5855 | 0.9996 | NA |
| AAEL011412 | | 0.3324 | 2.5781 | 0.5855 | 0.9996 | |
| AAEL009324 | | 0.1713 | 5.8710 | 0.5856 | 0.9996 | hydroxyacyl dehydrogenase [Source:VB Community Annotation] |
| AAEL008354 | | -0.6129 | 6.8446 | 0.5856 | 0.9996 | gaba receptor invertebrate [Source:VB Community Annotation] |
| AAEL011718 | | 0.1959 | 4.9248 | 0.5856 | 0.9996 | |
| AAEL006103 | OBP25 | 0.1943 | 7.7574 | 0.5856 | 0.9996 | odorant binding protein OBP25 [Source:VB Community Annotation] |
| AAEL005777 | | 0.2072 | 2.1508 | 0.5856 | 0.9996 | |
| AAEL001421 | | -0.5570 | 3.5561 | 0.5857 | 0.9996 | high density lipoprotien binding protein / vigilin [Source:VB Community Annotation] |

| | | | | | | |
|---|---|---|---|---|---|---|
| AAEL020253 | NA | -0.6983 | 4.1862 | 0.5857 | 0.9996 | NA |
| AAEL002572 | | 0.3658 | 9.7369 | 0.5858 | 0.9996 | myosin regulatory light chain 2 (mlc-2) [Source:VB Community Annotation] |
| AAEL013846 | | -0.8839 | -0.5863 | 0.5861 | 0.9996 | |
| AAEL021083 | NA | 0.1507 | 11.3791 | 0.5861 | 0.9996 | NA |
| AAEL009835 | | -0.2001 | 5.0281 | 0.5861 | 0.9996 | |
| AAEL006900 | | 0.3128 | 3.2114 | 0.5861 | 0.9996 | |
| AAEL022168 | NA | 0.1708 | 4.9091 | 0.5862 | 0.9996 | NA |
| AAEL011070 | CTLGA3 | -0.3537 | 7.5588 | 0.5862 | 0.9996 | C-Type Lectin (CTL) - galactose binding. [Source:VB Community Annotation] |
| AAEL000386 | | -0.1987 | 4.6847 | 0.5864 | 0.9996 | phopsphatidylinositol 3-kinase, drosophila [Source:VB Community Annotation] |
| AAEL003246 | | 0.3561 | 2.5295 | 0.5866 | 0.9996 | deoxyribose-phosphate aldolase [Source:VB Community Annotation] |
| AAEL017447 | | -0.2817 | 6.6653 | 0.5866 | 0.9996 | Casein kinase II subunit beta [Source:UniProtKB/TrEMBL;Acc:J9I020] |
| AAEL021666 | NA | -0.4222 | 8.7015 | 0.5866 | 0.9996 | NA |
| AAEL003230 | | 0.1324 | 5.5483 | 0.5867 | 0.9996 | |
| AAEL019859 | NA | 0.5406 | 0.4937 | 0.5867 | 0.9996 | NA |
| AAEL012195 | | -0.1325 | 4.0832 | 0.5869 | 0.9996 | small GTPase, putative [Source:VB Community Annotation] |
| AAEL024256 | NA | 0.4372 | 2.4103 | 0.5870 | 0.9996 | NA |
| AAEL024541 | NA | 0.4545 | 2.0003 | 0.5870 | 0.9996 | NA |
| AAEL002539 | | -0.1332 | 7.5276 | 0.5871 | 0.9996 | fimbrin/plastin [Source:VB Community Annotation] |
| AAEL027790 | NA | -0.5880 | 5.7372 | 0.5871 | 0.9996 | NA |
| AAEL012073 | | 0.2621 | 3.1123 | 0.5871 | 0.9996 | |
| AAEL013700 | | -0.1590 | 5.4250 | 0.5872 | 0.9996 | DNA-directed RNA polymerase II 13.3 kDa polypeptide [Source:VB Community Annotation] |
| AAEL009423 | SCRBQ2 | -0.1957 | 5.5192 | 0.5872 | 0.9996 | Class B Scavenger Receptor (CD36 domain). [Source:VB Community Annotation |
| AAEL008305 | | -1.0029 | 9.3143 | 0.5873 | 0.9996 | |
| AAEL006509 | | 0.2211 | 10.2018 | 0.5874 | 0.9996 | |
| AAEL011881 | | -0.1378 | 6.8137 | 0.5875 | 0.9996 | |
| AAEL000405 | | -0.6180 | 6.5291 | 0.5876 | 0.9996 | odd Oz protein [Source:VB Community Annotation] |
| AAEL010823 | | 0.2115 | 9.8550 | 0.5877 | 0.9996 | ATP synthase delta chain [Source:VB Community Annotation] |
| AAEL020196 | NA | 0.2458 | 3.3186 | 0.5877 | 0.9996 | NA |
| AAEL009388 | | -0.1419 | 3.9818 | 0.5878 | 0.9996 | Coiled-coil domain-containing protein 22 homolog [Source:UniProtKB/Swiss-Prot;Acc:Q16VW9] |
| AAEL019607 | NA | 0.4957 | 0.4413 | 0.5879 | 0.9996 | NA |
| AAEL009031 | | -0.9062 | -1.5304 | 0.5880 | 0.9996 | forkhead box protein (AaegFOXF) [Source:VB Community Annotation] |
| AAEL020583 | NA | -0.1531 | 4.0787 | 0.5881 | 0.9996 | NA |
| AAEL011227 | | 0.4825 | -0.1805 | 0.5883 | 0.9996 | |
| AAEL009077 | ALP1 | -0.3505 | 4.1857 | 0.5884 | 0.9996 | alkaline phosphatase [Source:VB Community Annotation] |
| AAEL003022 | | -0.1432 | 5.4384 | 0.5885 | 0.9996 | serine/threonine-protein kinase [Source:VB Community Annotation] |
| AAEL001707 | | 0.3514 | 1.3813 | 0.5885 | 0.9996 | epsilon-trimethyllysine 2-oxoglutarate dioxygenase [Source:VB Community Annotation] |
| AAEL027357 | NA | -0.1932 | 7.3269 | 0.5886 | 0.9996 | NA |
| AAEL005425 | | 0.7043 | -0.7898 | 0.5887 | 0.9996 | |
| AAEL013111 | | -0.2106 | 5.9250 | 0.5887 | 0.9996 | glutamate transporter [Source:VB Community Annotation] |
| AAEL000651 | | -0.9818 | -0.7174 | 0.5887 | 0.9996 | alpha-amylase [Source:VB Community Annotation] |

| | | | | | | |
|---|---|---|---|---|---|---|
| AAEL014292 | RpS24 | -0.1471 | 11.2308 | 0.5888 | 0.9996 | 40S ribosomal protein S24 [Source:UniProtKB/TrEMBL;Acc:Q8WQK7] |
| AAEL013194 | | 0.3381 | 1.2216 | 0.5889 | 0.9996 | |
| AAEL006419 | | -0.9716 | 2.1449 | 0.5892 | 0.9996 | recombining binding protein suppressor of hairless [Source:VB Community Annotation] |
| AAEL024756 | NA | -0.3649 | 3.6545 | 0.5893 | 0.9996 | NA |
| AAEL022218 | NA | 0.4322 | 0.9151 | 0.5893 | 0.9996 | NA |
| AAEL006144 | IPPI | -0.1466 | 5.6702 | 0.5893 | 0.9996 | isopentenyl-diphosphate delta isomerase [Source:VB Community Annotation] |
| AAEL025567 | NA | -0.1705 | 6.2865 | 0.5894 | 0.9996 | NA |
| AAEL021353 | NA | -0.1241 | 4.5764 | 0.5894 | 0.9996 | NA |
| AAEL011478 | | -0.1745 | 6.7725 | 0.5895 | 0.9996 | cytoplasmic dynein light chain [Source:VB Community Annotation] |
| AAEL009467 | | 0.1806 | 3.9882 | 0.5895 | 0.9996 | |
| AAEL008480 | | 0.2368 | 2.9392 | 0.5896 | 0.9996 | |
| AAEL000847 | | -0.1470 | 7.1097 | 0.5898 | 0.9996 | |
| AAEL014804 | | -0.1659 | 5.8682 | 0.5899 | 0.9996 | |
| AAEL014820 | | -0.5653 | 0.6166 | 0.5900 | 0.9996 | |
| AAEL020885 | NA | -0.1955 | 2.6980 | 0.5901 | 0.9996 | NA |
| AAEL029004 | NA | 0.1188 | 6.9425 | 0.5901 | 0.9996 | NA |
| AAEL011316 | | -0.1378 | 3.9375 | 0.5902 | 0.9996 | zinc finger protein [Source:VB Community Annotation] |
| AAEL010945 | | 0.1513 | 4.1369 | 0.5902 | 0.9996 | |
| AAEL024796 | NA | 0.1532 | 4.5084 | 0.5904 | 0.9996 | NA |
| AAEL028132 | NA | -0.8939 | 1.1588 | 0.5905 | 0.9996 | NA |
| AAEL007447 | | -0.6585 | 3.3647 | 0.5906 | 0.9996 | |
| AAEL011995 | | -0.1766 | 5.4837 | 0.5906 | 0.9996 | |
| AAEL003405 | | -0.3517 | 4.2944 | 0.5906 | 0.9996 | |
| AAEL011568 | srp54 | 0.1269 | 5.4782 | 0.5907 | 0.9996 | |
| AAEL002541 | | 0.6221 | 1.0268 | 0.5907 | 0.9996 | cystinosin [Source:VB Community Annotation] |
| AAEL005478 | | 0.1788 | 3.2920 | 0.5907 | 0.9996 | flavohemoprotein B5/b5r [Source:VB Community Annotation] |
| AAEL004874 | | 0.3702 | 2.5815 | 0.5907 | 0.9996 | limd1 [Source:VB Community Annotation] |
| AAEL008718 | | 0.1327 | 4.8251 | 0.5907 | 0.9996 | |
| AAEL006334 | | -0.6882 | 0.0685 | 0.5908 | 0.9996 | sulfotransferase (sult) [Source:VB Community Annotation] |
| AAEL014284 | | 0.2677 | 2.7500 | 0.5908 | 0.9996 | |
| AAEL024106 | NA | 0.1851 | 2.8202 | 0.5909 | 0.9996 | NA |
| AAEL027557 | NA | 0.1616 | 4.2551 | 0.5910 | 0.9996 | NA |
| AAEL007511 | | -0.7085 | -0.5168 | 0.5910 | 0.9996 | serine protease [Source:VB Community Annotation] |
| AAEL008598 | | -0.4558 | -0.2487 | 0.5912 | 0.9996 | |
| AAEL025841 | NA | -0.1837 | 5.1856 | 0.5912 | 0.9996 | NA |
| AAEL002404 | | 0.3560 | 1.5652 | 0.5913 | 0.9996 | receptor protein tyrosine kinase [Source:VB Community Annotation] |
| AAEL000159 | | -0.1804 | 6.1208 | 0.5914 | 0.9996 | nipsnap [Source:VB Community Annotation] |
| AAEL025376 | NA | -0.2431 | 2.3921 | 0.5914 | 0.9996 | NA |
| AAEL020454 | NA | -0.4638 | 0.9786 | 0.5915 | 0.9996 | NA |
| AAEL005407 | | 0.1427 | 6.0330 | 0.5920 | 0.9996 | annexin x [Source:VB Community Annotation] |

| | | | | | | |
|---|---|---|---|---|---|---|
| AAEL020631 | NA | -0.9762 | 1.8021 | 0.5920 | 0.9996 | NA |
| AAEL006694 | | 0.2790 | 1.2571 | 0.5921 | 0.9996 | |
| AAEL005830 | Prosalpha4 | -0.2418 | 7.2482 | 0.5922 | 0.9996 | 26S proteasome alpha 4 subunit [Source:VB Community Annotation] |
| AAEL024792 | NA | -0.8343 | -1.6008 | 0.5922 | 0.9996 | NA |
| AAEL007549 | | 0.1667 | 8.0412 | 0.5922 | 0.9996 | phosphatidylethanolamine-binding protein [Source:VB Community Annotation] |
| AAEL013876 | | -0.2631 | 9.8510 | 0.5923 | 0.9996 | mitochondrial NADH:ubiquinone oxidoreductase B14.7 subunit, putative [Source:VB Community Annotation] |
| AAEL015116 | | 0.8158 | 5.7412 | 0.5925 | 0.9996 | prophenoloxidase [Source:VB Community Annotation] |
| AAEL020390 | NA | -0.7831 | 1.8719 | 0.5926 | 0.9996 | NA |
| AAEL000022 | | 0.1752 | 5.4100 | 0.5926 | 0.9996 | |
| AAEL009225 | | 0.1997 | 4.2327 | 0.5927 | 0.9996 | mitochondrial ribosome recycling factor [Source:VB Community Annotation] |
| AAEL009959 | | 0.1574 | 6.0226 | 0.5928 | 0.9996 | pre-mRNA splicing factor prp8 [Source:VB Community Annotation] |
| AAEL010974 | | -0.1536 | 4.4102 | 0.5929 | 0.9996 | Putative phd finger protein af10 [Source:UniProtKB/TrEMBL;Acc:A0A0P6IWC6] |
| AAEL007699 | RpL9 | 0.2496 | 11.5170 | 0.5931 | 0.9996 | 60S ribosomal protein L9 [Source:VB Community Annotation] |
| AAEL009055 | | -0.2288 | 3.1608 | 0.5931 | 0.9996 | gliotactin [Source:VB Community Annotation] |
| AAEL002216 | | 0.2338 | 6.4312 | 0.5932 | 0.9996 | 5-AMP-activated protein kinase, beta subunit [Source:VB Community Annotation] |
| AAEL005262 | | 0.2130 | 6.2279 | 0.5933 | 0.9996 | |
| AAEL009402 | | 0.6138 | -1.2275 | 0.5935 | 0.9996 | map kinase-activated protein kinase (mapkapk) [Source:VB Community Annotation] |
| AAEL001519 | | -0.4117 | 0.9700 | 0.5935 | 0.9996 | |
| AAEL024251 | NA | -0.3201 | 4.8067 | 0.5935 | 0.9996 | NA |
| AAEL017503 | | 0.2048 | 3.3950 | 0.5936 | 0.9996 | Presenilin [Source:UniProtKB/TrEMBL;Acc:J9I024] |
| AAEL002610 | | -0.5047 | 8.2525 | 0.5937 | 0.9996 | serine protease [Source:VB Community Annotation] |
| AAEL024733 | NA | -0.6421 | -1.4821 | 0.5938 | 0.9996 | NA |
| AAEL022465 | NA | -0.6497 | 4.1241 | 0.5939 | 0.9996 | NA |
| AAEL020484 | NA | -0.9021 | -1.2165 | 0.5940 | 0.9996 | NA |
| AAEL006876 | | 0.6742 | -1.0811 | 0.5941 | 0.9996 | igf2 mRNA binding protein, putative [Source:VB Community Annotation] |
| AAEL008889 | CYP6AL1 | -0.3703 | 5.6197 | 0.5941 | 0.9996 | cytochrome P450 [Source:VB Community Annotation] |
| AAEL009762 | CYP307A1 | 0.8198 | -2.0875 | 0.5944 | 0.9996 | cytochrome P450 [Source:VB Community Annotation] |
| AAEL022901 | NA | -0.1722 | 5.1989 | 0.5944 | 0.9996 | NA |
| AAEL005728 | | 0.1412 | 6.0537 | 0.5945 | 0.9996 | |
| AAEL012064 | | -0.2488 | 8.5632 | 0.5947 | 0.9996 | Niemann-Pick Type C-2, putative [Source:VB Community Annotation] |
| AAEL014286 | | 0.2858 | 2.2377 | 0.5949 | 0.9996 | |
| AAEL011865 | | 0.2037 | 4.8711 | 0.5949 | 0.9996 | |
| AAEL027384 | NA | -0.2911 | 7.1179 | 0.5950 | 0.9996 | NA |
| AAEL000340 | | -0.3175 | 3.5266 | 0.5950 | 0.9996 | cytochrome P450 [Source:VB Community Annotation] |
| AAEL012887 | | -0.1722 | 6.0044 | 0.5950 | 0.9996 | |
| AAEL020547 | NA | -0.6563 | 3.2665 | 0.5951 | 0.9996 | NA |
| AAEL024226 | NA | 0.8636 | -1.3464 | 0.5952 | 0.9996 | NA |
| AAEL014827 | | 0.2653 | 2.4722 | 0.5952 | 0.9996 | |
| AAEL020559 | NA | -0.1414 | 7.4166 | 0.5952 | 0.9996 | NA |
| AAEL022773 | NA | 0.2058 | 4.2403 | 0.5952 | 0.9996 | NA |

| | | | | | | |
|---|---|---|---|---|---|---|
| AAEL028155 | NA | 0.2512 | 2.3351 | 0.5953 | 0.9996 | NA |
| AAEL006634 | | 0.3926 | 5.7267 | 0.5954 | 0.9996 | acetyl-coa acetyltransferase, mitochondrial (acetoacetyl-coa thiolase) [Source:VB Community Annotation] |
| AAEL017345 | | -0.4055 | 8.6068 | 0.5954 | 0.9996 | |
| AAEL007653 | | 0.5038 | 6.9863 | 0.5954 | 0.9996 | allantoinase [Source:VB Community Annotation] |
| AAEL001761 | | -0.1949 | 3.6794 | 0.5957 | 0.9996 | exonuclease [Source:VB Community Annotation] |
| AAEL001959 | | -0.1729 | 5.3808 | 0.5957 | 0.9996 | |
| AAEL013338 | | -0.9165 | -0.8268 | 0.5959 | 0.9996 | lethal(2)essential for life protein, l2efl [Source:VB Community Annotation] |
| AAEL000021 | | 0.5427 | 0.2609 | 0.5959 | 0.9996 | |
| AAEL026027 | NA | -0.3054 | 4.5360 | 0.5959 | 0.9996 | NA |
| AAEL005692 | | -0.3510 | 9.3694 | 0.5960 | 0.9996 | |
| AAEL000134 | | -0.1650 | 4.0372 | 0.5960 | 0.9996 | |
| AAEL013869 | | 0.1418 | 4.3471 | 0.5961 | 0.9996 | RNA-binding protein [Source:VB Community Annotation] |
| AAEL021798 | NA | 0.6946 | -1.2390 | 0.5963 | 0.9996 | NA |
| AAEL009511 | | 0.2582 | 2.0550 | 0.5965 | 0.9996 | |
| AAEL005284 | | -0.6729 | 3.9473 | 0.5965 | 0.9996 | receptor tyrosine phosphatase type r2a [Source:VB Community Annotation] |
| AAEL006109 | OBP23 | -0.4559 | 4.6959 | 0.5965 | 0.9996 | odorant binding protein OBP23 [Source:VB Community Annotation] |
| AAEL009232 | | 0.1180 | 6.3767 | 0.5965 | 0.9996 | long-chain-fatty-acid coa ligase [Source:VB Community Annotation] |
| AAEL020878 | NA | 0.1826 | 6.4673 | 0.5966 | 0.9996 | NA |
| AAEL000904 | | 0.6026 | -0.8989 | 0.5968 | 0.9996 | Carboxylic ester hydrolase (Fragment) [Source:UniProtKB/TrEMBL;Acc:Q17MV6] |
| AAEL009561 | | 0.7116 | 0.2851 | 0.5970 | 0.9996 | apolipoprotein D, putative [Source:VB Community Annotation] |
| AAEL011414 | | -0.4289 | 8.2232 | 0.5970 | 0.9996 | high mobility group non-histone protein, putative [Source:VB Community Annotation] |
| AAEL019877 | NA | -0.2128 | 5.6551 | 0.5970 | 0.9996 | NA |
| AAEL010623 | | 0.3009 | 1.6700 | 0.5973 | 0.9996 | |
| AAEL007236 | | -0.2287 | 8.3174 | 0.5973 | 0.9996 | |
| AAEL020242 | NA | 0.2015 | 4.0487 | 0.5973 | 0.9996 | NA |
| AAEL001509 | | -0.2261 | 4.7762 | 0.5975 | 0.9996 | |
| AAEL018050 | | -0.1949 | 2.7357 | 0.5976 | 0.9996 | |
| AAEL012586 | | -0.4239 | 3.7571 | 0.5977 | 0.9996 | |
| AAEL001119 | | 0.1598 | 4.8279 | 0.5977 | 0.9996 | DNA-binding protein Ewg, putative [Source:VB Community Annotation] |
| AAEL009371 | | -0.7239 | 0.9184 | 0.5978 | 0.9996 | |
| AAEL006460 | | -0.9417 | 1.9840 | 0.5978 | 0.9996 | par-6 gamma [Source:VB Community Annotation] |
| AAEL012867 | | 0.6309 | -0.0837 | 0.5979 | 0.9996 | |
| AAEL002009 | | 0.5919 | 2.2017 | 0.5979 | 0.9996 | neutral Sphingomyelinase, putative [Source:VB Community Annotation] |
| AAEL004445 | | 0.1708 | 5.2654 | 0.5979 | 0.9996 | DEAD box ATP-dependent RNA helicase [Source:VB Community Annotation] |
| AAEL000415 | | -0.3182 | 6.0037 | 0.5981 | 0.9996 | AMP dependent coa ligase [Source:VB Community Annotation] |
| AAEL005481 | | -0.1660 | 5.9499 | 0.5981 | 0.9996 | alpha-glucosidase [Source:VB Community Annotation] |
| AAEL001352 | | 0.1381 | 6.5469 | 0.5982 | 0.9996 | scaffold attachment factor b [Source:VB Community Annotation] |
| AAEL017526 | | -0.4536 | 4.5502 | 0.5983 | 0.9996 | |
| AAEL011764 | PPO10 | 0.6395 | 3.8561 | 0.5983 | 0.9996 | prophenoloxidase [Source:VB Community Annotation] |
| AAEL012260 | | -0.2740 | 3.7755 | 0.5983 | 0.9996 | WD-repeat protein [Source:VB Community Annotation] |

| | | | | | | |
|---|---|---|---|---|---|---|
| AAEL005386 | | 0.3946 | 2.5919 | 0.5983 | 0.9996 | collagen alpha chain, anopheles [Source:VB Community Annotation] |
| AAEL019813 | NA | -0.2253 | 6.6074 | 0.5985 | 0.9996 | NA |
| AAEL004653 | | -0.1501 | 5.8438 | 0.5985 | 0.9996 | elongation factor SIII p15 subunit, putative [Source:VB Community Annotation] |
| AAEL006733 | | -0.2264 | 4.6385 | 0.5986 | 0.9996 | FAD NADPH dehydrogenase [Source:VB Community Annotation] |
| AAEL010073 | | -0.1460 | 5.3253 | 0.5987 | 0.9996 | metalloendopeptidase [Source:VB Community Annotation] |
| AAEL010004 | | -0.1772 | 6.3844 | 0.5988 | 0.9996 | MICOS complex subunit MIC60 [Source:UniProtKB/TrEMBL;Acc:A0A1S4FP00] |
| AAEL012924 | mRpS24 | 0.1707 | 4.7635 | 0.5990 | 0.9996 | mitochondrial ribosomal protein, S24, putative [Source:VB Community Annotation] |
| AAEL002508 | | -0.1636 | 6.6250 | 0.5991 | 0.9996 | 26S protease regulatory subunit 6a [Source:VB Community Annotation] |
| AAEL019805 | NA | 0.2258 | 3.1337 | 0.5992 | 0.9996 | NA |
| AAEL023183 | NA | 0.3106 | 1.2090 | 0.5993 | 0.9996 | NA |
| AAEL003132 | | -0.2278 | 2.2788 | 0.5994 | 0.9996 | intraflagellar transport 52 homolog (protein NGD5) [Source:VB Community Annotation] |
| AAEL007472 | | -0.2578 | 3.5549 | 0.5996 | 0.9996 | |
| AAEL014889 | | 0.1851 | 8.1377 | 0.5997 | 0.9996 | NADH:ubiquinone dehydrogenase, putative [Source:VB Community Annotation] |
| AAEL027774 | NA | 0.8045 | -0.0282 | 0.5997 | 0.9996 | NA |
| AAEL000687 | | 0.3621 | 2.8869 | 0.5998 | 0.9996 | glucosyl/glucuronosyl transferases [Source:VB Community Annotation] |
| AAEL019731 | NA | -0.2320 | 5.5418 | 0.5998 | 0.9996 | NA |
| AAEL011656 | RpS15 | 0.1970 | 11.4797 | 0.5998 | 0.9996 | 40S ribosomal protein S15 [Source:VB Community Annotation] |
| AAEL011288 | | 0.2356 | 8.8460 | 0.5998 | 0.9996 | elongation factor 1 gamma [Source:VB Community Annotation] |
| AAEL024441 | NA | -0.1487 | 5.4135 | 0.5999 | 0.9996 | NA |
| AAEL023329 | NA | 0.3371 | 1.9146 | 0.5999 | 0.9996 | NA |
| AAEL010665 | | 0.2162 | 3.6072 | 0.6001 | 0.9996 | developmentally regulated RNA-binding protein [Source:VB Community Annotation] |
| AAEL019649 | NA | 0.3727 | 0.6799 | 0.6003 | 0.9996 | NA |
| AAEL022530 | NA | -0.2821 | 3.0564 | 0.6004 | 0.9996 | NA |
| AAEL025755 | NA | 0.3302 | 2.3094 | 0.6004 | 0.9996 | NA |
| AAEL004685 | | 0.1250 | 5.5712 | 0.6004 | 0.9996 | succinate semialdehyde dehydrogenase [Source:VB Community Annotation] |
| AAEL009317 | | -0.1345 | 7.1399 | 0.6004 | 0.9996 | rab11 [Source:VB Community Annotation] |
| AAEL023676 | NA | -0.7051 | 3.7285 | 0.6004 | 0.9996 | NA |
| AAEL004715 | | -0.6898 | 6.1954 | 0.6004 | 0.9996 | b-cell translocation protein [Source:VB Community Annotation] |
| AAEL021895 | NA | 0.4661 | 1.1920 | 0.6005 | 0.9996 | NA |
| AAEL027120 | NA | 0.1289 | 5.3193 | 0.6006 | 0.9996 | NA |
| AAEL021349 | NA | 0.6058 | -0.1408 | 0.6007 | 0.9996 | NA |
| AAEL002764 | | 0.2164 | 7.9599 | 0.6008 | 0.9996 | dihydrolipoamide succinyltransferase component of 2-oxoglutarate dehydrogenase [Source:VB Community Annotation] |
| AAEL001315 | | 0.2075 | 3.0941 | 0.6008 | 0.9996 | |
| AAEL019565 | NA | -0.4819 | 4.4465 | 0.6009 | 0.9996 | NA |
| AAEL011877 | Art7 | -0.2101 | 2.3316 | 0.6012 | 0.9996 | Protein arginine N-methyltransferase 7 (EC 2.1.1.-) [Source:VB Community Annotation] |
| AAEL017301 | | -0.2021 | 9.2013 | 0.6014 | 0.9996 | Elongation factor 1-alpha [Source:UniProtKB/TrEMBL;Acc:J9HYQ9] |
| AAEL007351 | | 0.1638 | 4.8800 | 0.6014 | 0.9996 | |
| AAEL006548 | | -0.2091 | 2.2674 | 0.6014 | 0.9996 | |
| AAEL008334 | | -0.3203 | 4.5798 | 0.6015 | 0.9996 | |
| AAEL003193 | | 0.1460 | 7.7531 | 0.6016 | 0.9996 | inorganic pyrophosphatase [Source:VB Community Annotation] |

| | | | | | | |
|---|---|---|---|---|---|---|
| AAEL027634 | NA | -0.3427 | 0.3065 | 0.6016 | 0.9996 | NA |
| AAEL001238 | | 0.2037 | 3.2113 | 0.6016 | 0.9996 | bap28 [Source:VB Community Annotation] |
| AAEL004096 | | 0.2186 | 3.8039 | 0.6017 | 0.9996 | aldo-keto reductase [Source:VB Community Annotation] |
| AAEL007084 | | -0.4257 | 3.6311 | 0.6017 | 0.9996 | |
| AAEL027186 | NA | 0.1992 | 2.5755 | 0.6020 | 0.9996 | NA |
| AAEL009294 | | 0.2282 | 5.3476 | 0.6022 | 0.9996 | phosphatidylinositol 4-kinase [Source:VB Community Annotation] |
| AAEL007818 | | 0.7260 | 9.4485 | 0.6022 | 0.9996 | Trypsin 3A1 Precursor (EC 3.4.21.4) [Source:VB Community Annotation] |
| AAEL002196 | | 0.2659 | 2.6499 | 0.6023 | 0.9996 | procathepsin L3, putative [Source:VB Community Annotation] |
| AAEL022503 | NA | 0.2397 | 2.4127 | 0.6024 | 0.9996 | NA |
| AAEL001270 | | 0.1979 | 2.0959 | 0.6026 | 0.9996 | |
| AAEL012233 | | -0.6515 | 4.0995 | 0.6026 | 0.9996 | |
| AAEL009389 | | -0.2170 | 7.7158 | 0.6027 | 0.9996 | Transaldolase [Source:UniProtKB/TrEMBL;Acc:Q16VW8] |
| AAEL000420 | | 0.2886 | 3.4045 | 0.6029 | 0.9996 | cathepsin o [Source:VB Community Annotation] |
| AAEL024943 | NA | -0.2281 | 2.3834 | 0.6029 | 0.9996 | NA |
| AAEL027479 | NA | -0.3375 | 3.4667 | 0.6029 | 0.9996 | NA |
| AAEL026443 | NA | -0.1683 | 3.6111 | 0.6031 | 0.9996 | NA |
| AAEL001540 | | 0.2339 | 4.7526 | 0.6034 | 0.9996 | ubiquitin specific protease [Source:VB Community Annotation] |
| AAEL006619 | | -0.1761 | 6.6455 | 0.6034 | 0.9996 | |
| AAEL005367 | | -0.2158 | 4.6139 | 0.6035 | 0.9996 | myotubularin-related protein 2 [Source:VB Community Annotation] |
| AAEL002523 | | -0.1400 | 6.0462 | 0.6036 | 0.9996 | mitochondrial inner membrane protein translocase, 9kD-subunit, putative [Source:VB Community Annotation] |
| AAEL014964 | | -1.2592 | 3.2012 | 0.6036 | 0.9996 | |
| AAEL006774 | | 0.2990 | 2.1260 | 0.6036 | 0.9996 | Fatty acyl-CoA reductase [Source:UniProtKB/TrEMBL;Acc:Q174U8] |
| AAEL011650 | | 0.1146 | 7.1451 | 0.6037 | 0.9996 | coatomer, gamma-subunit, putative [Source:VB Community Annotation] |
| AAEL020749 | NA | -0.1613 | 11.2842 | 0.6038 | 0.9996 | NA |
| AAEL005142 | | -0.2427 | 2.4318 | 0.6038 | 0.9996 | platelet endothelial tetraspan antigen 3 (cd151 antigen) [Source:VB Community Annotation] |
| AAEL011377 | | -0.2097 | 4.3081 | 0.6040 | 0.9996 | |
| AAEL012489 | | -0.2068 | 3.1679 | 0.6040 | 0.9996 | |
| AAEL020582 | NA | 0.6776 | 1.2223 | 0.6040 | 0.9996 | NA |
| AAEL013233 | PIWI5 | 0.1844 | 4.6739 | 0.6041 | 0.9996 | PIWI [Source:VB Community Annotation] |
| AAEL007235 | | 0.1562 | 6.3565 | 0.6041 | 0.9996 | mitochondrial uncoupling protein [Source:VB Community Annotation] |
| AAEL011142 | | 0.1397 | 5.4792 | 0.6042 | 0.9996 | |
| AAEL015313 | OBP59 | 0.2860 | 6.7957 | 0.6042 | 0.9996 | odorant binding protein OBP59 [Source:VB Community Annotation] |
| AAEL006013 | | -0.1868 | 4.1857 | 0.6043 | 0.9996 | huntingtin interacting protein [Source:VB Community Annotation] |
| AAEL014452 | | -0.3813 | 8.6116 | 0.6047 | 0.9996 | acyl-coa dehydrogenase [Source:VB Community Annotation] |
| AAEL011178 | | 0.3263 | 2.7463 | 0.6050 | 0.9996 | posterior sex combs protein [Source:VB Community Annotation] |
| AAEL008234 | | -0.1308 | 4.9105 | 0.6051 | 0.9996 | dishevelled [Source:VB Community Annotation] |
| AAEL005926 | | 0.3023 | 4.2862 | 0.6052 | 0.9996 | ER lumen protein retaining receptor [Source:VB Community Annotation] |
| AAEL007256 | | 0.1265 | 4.5632 | 0.6053 | 0.9996 | DNA helicase recq1 [Source:VB Community Annotation] |
| AAEL007356 | | -0.1891 | 3.6482 | 0.6053 | 0.9996 | |
| AAEL022608 | NA | 0.1429 | 4.6414 | 0.6054 | 0.9996 | NA |

| | | | | | | |
|---|---|---|---|---|---|---|
| AAEL023096 | NA | 0.3102 | 1.1550 | 0.6055 | 0.9996 | NA |
| AAEL009110 | | -0.9127 | 2.0506 | 0.6055 | 0.9996 | smad anchor for receptor activation [Source:VB Community Annotation] |
| AAEL003657 | | 0.2013 | 2.0774 | 0.6056 | 0.9996 | zinc finger protein [Source:VB Community Annotation] |
| AAEL007757 | | -0.4129 | -0.1069 | 0.6058 | 0.9996 | |
| AAEL023164 | NA | -0.4905 | 0.0205 | 0.6058 | 0.9996 | NA |
| AAEL006721 | | 0.1391 | 8.6525 | 0.6059 | 0.9996 | 2-oxoglutarate dehydrogenase [Source:VB Community Annotation] |
| AAEL014003 | | -0.3181 | 3.2922 | 0.6059 | 0.9996 | |
| AAEL026110 | NA | -0.6696 | -2.2825 | 0.6059 | 0.9996 | NA |
| AAEL009282 | | 0.1474 | 3.8409 | 0.6060 | 0.9996 | peptidyl-prolyl cis-trans isomerase (cyclophilin) [Source:VB Community Annotation] |
| AAEL007469 | | -0.2256 | 5.1672 | 0.6060 | 0.9996 | mitotic checkpoint protein bub3 [Source:VB Community Annotation] |
| AAEL006607 | | -1.0226 | -0.4809 | 0.6061 | 0.9996 | juvenile hormone-inducible protein, putative [Source:VB Community Annotation] |
| AAEL003779 | | -0.4171 | 3.2466 | 0.6062 | 0.9996 | |
| AAEL021103 | NA | 0.2437 | 4.9350 | 0.6063 | 0.9996 | NA |
| AAEL003202 | | 0.2790 | 3.7205 | 0.6064 | 0.9996 | cyclohex-1-ene-1-carboxyl-CoA hydratase, putative [Source:VB Community Annotation] |
| AAEL003115 | | 0.1941 | 3.2910 | 0.6064 | 0.9996 | tartan [Source:VB Community Annotation] |
| AAEL018230 | | -0.6691 | -1.5338 | 0.6065 | 0.9996 | |
| AAEL014536 | | 0.1662 | 2.8177 | 0.6066 | 0.9996 | embryonic ectoderm development protein [Source:VB Community Annotation] |
| AAEL027296 | NA | -0.6805 | -1.7103 | 0.6066 | 0.9996 | NA |
| AAEL019608 | NA | 0.2474 | 2.1358 | 0.6071 | 0.9996 | NA |
| AAEL009377 | | -0.3343 | 5.8565 | 0.6071 | 0.9996 | rap1 and [Source:VB Community Annotation] |
| AAEL001116 | | 0.2165 | 3.0046 | 0.6071 | 0.9996 | |
| AAEL013174 | | 0.6972 | 1.2404 | 0.6071 | 0.9996 | |
| AAEL008379 | | 0.1527 | 5.0455 | 0.6071 | 0.9996 | P38 mapk [Source:VB Community Annotation] |
| AAEL003380 | CYP4H28 | -0.5076 | 2.1652 | 0.6072 | 0.9996 | cytochrome P450 [Source:VB Community Annotation] |
| AAEL002447 | | -0.3453 | 3.8459 | 0.6073 | 0.9996 | |
| AAEL000149 | | -0.1829 | 3.6989 | 0.6074 | 0.9996 | Histone deacetylase complex subunit SAP30 homolog [Source:UniProtKB/Swiss-Prot;Acc:Q17Q39] |
| AAEL027636 | NA | 0.4171 | 0.8837 | 0.6074 | 0.9996 | NA |
| AAEL009220 | | 0.1945 | 3.6069 | 0.6075 | 0.9996 | |
| AAEL022453 | NA | 0.7794 | 1.4587 | 0.6075 | 0.9996 | NA |
| AAEL006905 | | -0.5353 | 4.0466 | 0.6075 | 0.9996 | |
| AAEL007267 | | 0.2027 | 3.3314 | 0.6076 | 0.9996 | suppressor of ty3 [Source:VB Community Annotation] |
| AAEL020380 | NA | -0.7055 | 4.2760 | 0.6078 | 0.9996 | NA |
| AAEL023491 | NA | 0.2727 | 1.4968 | 0.6079 | 0.9996 | NA |
| AAEL009202 | | 0.2274 | 4.0211 | 0.6081 | 0.9996 | |
| AAEL018301 | | 0.6504 | -0.9949 | 0.6081 | 0.9996 | |
| AAEL019617 | NA | -0.3769 | 5.4336 | 0.6082 | 0.9996 | NA |
| AAEL013728 | | -0.2362 | 3.9841 | 0.6083 | 0.9996 | |
| AAEL028101 | NA | -0.7199 | 6.7670 | 0.6084 | 0.9996 | NA |
| AAEL012427 | | 0.2017 | 4.3388 | 0.6084 | 0.9996 | |
| AAEL023391 | NA | -0.2057 | 3.3873 | 0.6084 | 0.9996 | NA |

| | | | | | | |
|---|---|---|---|---|---|---|
| AAEL005825 | | 0.1191 | 4.3584 | 0.6085 | 0.9996 | translation initiation factor eif-2b beta subunit [Source:VB Community Annotation] |
| AAEL023412 | NA | 0.2678 | 2.4896 | 0.6085 | 0.9996 | NA |
| AAEL023365 | NA | -0.1725 | 4.3726 | 0.6086 | 0.9996 | NA |
| AAEL009757 | | 0.7143 | -0.7872 | 0.6086 | 0.9996 | |
| AAEL024466 | NA | -0.7921 | -0.4280 | 0.6089 | 0.9996 | NA |
| AAEL003420 | | -0.1716 | 3.4378 | 0.6089 | 0.9996 | |
| AAEL003965 | | -0.2224 | 2.8272 | 0.6090 | 0.9996 | calpain 4, 6, 7, invertebrate [Source:VB Community Annotation] |
| AAEL009836 | | -0.6520 | 0.8508 | 0.6093 | 0.9996 | |
| AAEL015118 | | -0.6559 | 3.9366 | 0.6093 | 0.9996 | |
| AAEL005138 | | 0.1680 | 3.1066 | 0.6093 | 0.9996 | glucosyl/glucuronosyl transferases [Source:VB Community Annotation] |
| AAEL019957 | NA | -0.4457 | 2.1513 | 0.6094 | 0.9996 | NA |
| AAEL023935 | NA | 0.6037 | -0.3722 | 0.6094 | 0.9996 | NA |
| AAEL001200 | | 0.2350 | 2.9776 | 0.6095 | 0.9996 | aspartyl-tRNA synthetase [Source:VB Community Annotation] |
| AAEL008250 | | 0.2162 | 3.1025 | 0.6095 | 0.9996 | |
| AAEL002745 | | -1.3774 | 1.3943 | 0.6096 | 0.9996 | nucleoprotein, putative [Source:VB Community Annotation] |
| AAEL003352 | RpL7ae | -0.2872 | 5.6801 | 0.6097 | 0.9996 | 60S ribosomal protein l7ae [Source:VB Community Annotation] |
| AAEL013600 | | -0.1871 | 3.7382 | 0.6098 | 0.9996 | integrin alpha-ps [Source:VB Community Annotation] |
| AAEL006363 | | 0.3156 | 3.9967 | 0.6098 | 0.9996 | ribose-phosphate pyrophosphokinase 1,2 [Source:VB Community Annotation] |
| AAEL015216 | | 0.1859 | 3.8598 | 0.6098 | 0.9996 | serine/threonine-protein kinase vrk [Source:VB Community Annotation] |
| AAEL019618 | NA | -0.3624 | 6.7429 | 0.6099 | 0.9996 | NA |
| AAEL027988 | NA | -0.4243 | 0.2441 | 0.6099 | 0.9996 | NA |
| AAEL014438 | | -0.5007 | 1.3312 | 0.6102 | 0.9996 | juvenile hormone-inducible protein, putative [Source:VB Community Annotation] |
| AAEL011978 | | -0.1201 | 6.2995 | 0.6102 | 0.9996 | mannosidase alpha class 2a [Source:VB Community Annotation] |
| AAEL011941 | | -0.1994 | 8.1924 | 0.6102 | 0.9996 | oxidase/peroxidase [Source:VB Community Annotation] |
| AAEL013537 | | 0.1635 | 4.3158 | 0.6104 | 0.9996 | |
| AAEL013128 | | -0.2187 | 5.8969 | 0.6104 | 0.9996 | elongase, putative [Source:VB Community Annotation] |
| AAEL028105 | NA | 0.3248 | 5.6329 | 0.6104 | 0.9996 | NA |
| AAEL017451 | | -0.4570 | 6.3803 | 0.6105 | 0.9996 | |
| AAEL004017 | | 0.2053 | 3.8607 | 0.6105 | 0.9996 | DNA polymerase v [Source:VB Community Annotation] |
| AAEL000967 | | 0.4685 | 1.0787 | 0.6106 | 0.9996 | tmc7 protein [Source:VB Community Annotation] |
| AAEL010177 | | 0.1554 | 3.6196 | 0.6106 | 0.9996 | |
| AAEL007705 | | -0.4531 | 5.9514 | 0.6108 | 0.9996 | hect E3 ubiquitin ligase [Source:VB Community Annotation] |
| AAEL008822 | | 0.2438 | 3.1047 | 0.6108 | 0.9996 | |
| AAEL006966 | | 0.1761 | 6.9354 | 0.6108 | 0.9996 | vitellogenin, putative [Source:VB Community Annotation] |
| AAEL008553 | | 0.2043 | 6.9499 | 0.6111 | 0.9996 | |
| AAEL018254 | | -0.1381 | 4.2165 | 0.6111 | 0.9996 | |
| AAEL005734 | | -1.1663 | 2.9039 | 0.6114 | 0.9996 | leucine-rich transmembrane protein [Source:VB Community Annotation] |
| AAEL004347 | | 0.1357 | 7.3047 | 0.6115 | 0.9996 | Eukaryotic translation initiation factor 3 subunit M (eIF3m) [Source:VB Community Annotation] |
| AAEL005681 | GPRHIS | 1.0509 | -0.3364 | 0.6115 | 0.9996 | GPCR Histamine Family [Source:VB Community Annotation] |
| AAEL026965 | NA | -0.6602 | -0.2696 | 0.6115 | 0.9996 | NA |

| | | | | | | |
|---|---|---|---|---|---|---|
| AAEL006441 | Prosbeta1 | -0.1376 | 6.5004 | 0.6115 | 0.9996 | 26S proteasome beta 1 subunit [Source:VB Community Annotation] |
| AAEL009685 | | 0.1547 | 4.2938 | 0.6116 | 0.9996 | oxidoreductase [Source:VB Community Annotation] |
| AAEL008630 | | -0.4103 | 0.1469 | 0.6117 | 0.9996 | GTP-binding protein alpha subunit, gna [Source:VB Community Annotation] |
| AAEL001982 | | -0.1892 | 3.8818 | 0.6117 | 0.9996 | endoplasmic reticulum-resident kdel protein [Source:VB Community Annotation] |
| AAEL009183 | | 0.1765 | 5.4538 | 0.6117 | 0.9996 | cytochrome oxidase biogenesis protein (oxa1 mitochondrial) [Source:VB Community Annotation] |
| AAEL023076 | NA | 0.1540 | 2.3063 | 0.6118 | 0.9996 | NA |
| AAEL019922 | NA | -0.3647 | 3.6952 | 0.6118 | 0.9996 | NA |
| AAEL000905 | | 0.8198 | -1.2282 | 0.6118 | 0.9996 | Carboxylic ester hydrolase (Fragment) [Source:UniProtKB/TrEMBL;Acc:Q17MV5] |
| AAEL010085 | | -0.3506 | 1.2516 | 0.6118 | 0.9996 | DNA polymerase epsilon subunit, putative [Source:VB Community Annotation] |
| AAEL001518 | | 0.2310 | 3.3270 | 0.6118 | 0.9996 | zinc finger protein [Source:VB Community Annotation] |
| AAEL008103 | RpS8 | 0.1671 | 10.9788 | 0.6119 | 0.9996 | 40S ribosomal protein S8 [Source:UniProtKB/TrEMBL;Acc:Q1HRQ9] |
| AAEL005979 | SCRB3 | -0.6476 | 1.6971 | 0.6120 | 0.9996 | Class B Scavenger Receptor (CD36 domain). [Source:VB Community Annotation |
| AAEL004234 | | 0.1058 | 8.1763 | 0.6121 | 0.9996 | |
| AAEL003267 | | -0.3494 | 6.9179 | 0.6121 | 0.9996 | |
| AAEL013687 | | -0.3868 | 2.2858 | 0.6122 | 0.9996 | |
| AAEL004868 | | -0.2623 | 4.7899 | 0.6122 | 0.9996 | hemomucin [Source:VB Community Annotation] |
| AAEL021024 | NA | -0.2394 | 1.7886 | 0.6123 | 0.9996 | NA |
| AAEL010868 | | 0.7241 | -1.4390 | 0.6123 | 0.9996 | sugar transporter [Source:VB Community Annotation] |
| AAEL027972 | NA | 0.6694 | -0.7359 | 0.6125 | 0.9996 | NA |
| AAEL005752 | | 0.6474 | -0.4658 | 0.6125 | 0.9996 | lysosomal alpha-mannosidase (mannosidase alpha class 2b member 1) [Source:VB Community Annotation] |
| AAEL022129 | NA | -0.1951 | 5.0722 | 0.6125 | 0.9996 | NA |
| AAEL001882 | | -0.2654 | 3.5284 | 0.6127 | 0.9996 | |
| AAEL022767 | NA | -0.6945 | 2.2136 | 0.6127 | 0.9996 | NA |
| AAEL021581 | NA | -0.2435 | 3.7347 | 0.6128 | 0.9996 | NA |
| AAEL011632 | | -0.1913 | 5.3433 | 0.6129 | 0.9996 | fk506 binding protein [Source:VB Community Annotation] |
| AAEL000803 | | -0.3766 | 3.7871 | 0.6129 | 0.9996 | |
| AAEL012078 | | 0.1791 | 5.8710 | 0.6129 | 0.9996 | Gamma-glutamylcyclotransferase [Source:UniProtKB/TrEMBL;Acc:Q16N66] |
| AAEL002273 | | 0.7308 | -0.1656 | 0.6131 | 0.9996 | trypsin, putative [Source:VB Community Annotation] |
| AAEL020740 | NA | -0.3287 | 2.9794 | 0.6131 | 0.9996 | NA |
| AAEL012219 | | -0.3065 | 6.6807 | 0.6132 | 0.9996 | ubiquitin specific protease [Source:VB Community Annotation] |
| AAEL007259 | | -0.3953 | 7.3760 | 0.6132 | 0.9996 | |
| AAEL010498 | | -0.2662 | 1.7672 | 0.6132 | 0.9996 | zinc finger protein [Source:VB Community Annotation] |
| AAEL001639 | | -0.3817 | 1.3895 | 0.6132 | 0.9996 | |
| AAEL011520 | | 0.6302 | -0.1311 | 0.6133 | 0.9996 | sucrose transport protein [Source:VB Community Annotation] |
| AAEL004273 | | -0.5808 | -1.2283 | 0.6133 | 0.9996 | short-chain dehydrogenase [Source:VB Community Annotation] |
| AAEL007464 | | 0.2709 | 0.9769 | 0.6134 | 0.9996 | |
| AAEL011135 | | 0.2225 | 3.7377 | 0.6134 | 0.9996 | |
| AAEL014904 | | -0.2418 | 4.3650 | 0.6135 | 0.9996 | DEAD box ATP-dependent RNA helicase [Source:VB Community Annotation] |
| AAEL007534 | | 0.1605 | 5.5178 | 0.6136 | 0.9996 | |
| AAEL020382 | NA | -0.1364 | 6.7930 | 0.6137 | 0.9996 | NA |

| | | | | | | |
|---|---|---|---|---|---|---|
| AAEL027566 | NA | 0.5779 | -0.8706 | 0.6138 | 0.9996 | NA |
| AAEL026354 | NA | 1.0068 | 0.9505 | 0.6138 | 0.9996 | NA |
| AAEL001577 | | -0.9967 | 2.6954 | 0.6139 | 0.9996 | |
| AAEL019332 | LSU_rRNA_ | -0.8575 | -1.5993 | 0.6140 | 0.9996 | Eukaryotic large subunit ribosomal RNA [Source:RFAM;Acc:RF02543] |
| AAEL009782 | | 0.1656 | 5.9202 | 0.6141 | 0.9996 | brain chitinase and chia [Source:VB Community Annotation] |
| AAEL003032 | | -0.1674 | 3.4359 | 0.6143 | 0.9996 | PHD finger protein [Source:VB Community Annotation] |
| AAEL013203 | | 0.2258 | 6.1042 | 0.6143 | 0.9996 | |
| AAEL010626 | GPRNPY2 | -0.9158 | -0.8199 | 0.6144 | 0.9996 | GPCR Neuropeptide Y Family [Source:VB Community Annotation] |
| AAEL003951 | | 0.2828 | 3.0655 | 0.6147 | 0.9996 | |
| AAEL023611 | NA | -0.3891 | 3.8534 | 0.6148 | 0.9996 | NA |
| AAEL008144 | | -0.2715 | 5.9342 | 0.6149 | 0.9996 | AMP dependent ligase [Source:VB Community Annotation] |
| AAEL005474 | | -0.2421 | 7.9965 | 0.6149 | 0.9996 | |
| AAEL008160 | FAS2 | -0.8999 | -0.4109 | 0.6150 | 0.9996 | fatty acid synthase [Source:VB Community Annotation] |
| AAEL011598 | | 0.3774 | 1.0136 | 0.6151 | 0.9996 | |
| AAEL007872 | | 0.8640 | 6.6021 | 0.6151 | 0.9996 | |
| AAEL001901 | | -0.6186 | 3.6191 | 0.6151 | 0.9996 | MRAS2, putative [Source:VB Community Annotation] |
| AAEL006576 | | 0.4081 | 7.7567 | 0.6152 | 0.9996 | clip-domain serine protease, putative [Source:VB Community Annotation] |
| AAEL005863 | | 0.1552 | 3.6895 | 0.6152 | 0.9996 | |
| AAEL003692 | | 0.2923 | 0.8424 | 0.6152 | 0.9996 | |
| AAEL013993 | | -0.3710 | 0.4574 | 0.6153 | 0.9996 | |
| AAEL005568 | | -0.1639 | 4.1506 | 0.6153 | 0.9996 | |
| AAEL011708 | | -0.1711 | 7.3665 | 0.6154 | 0.9996 | heat shock protein [Source:VB Community Annotation] |
| AAEL022184 | NA | 0.2377 | 3.9462 | 0.6154 | 0.9996 | NA |
| AAEL002373 | | 0.3513 | 1.9864 | 0.6154 | 0.9996 | juvenile hormone-inducible protein, putative [Source:VB Community Annotation] |
| AAEL006044 | CYP325Q1 | 0.2953 | 2.1076 | 0.6154 | 0.9996 | cytochrome P450 [Source:VB Community Annotation] |
| AAEL012577 | | -0.3080 | 6.5558 | 0.6156 | 0.9996 | |
| AAEL007285 | | 0.2985 | 1.2283 | 0.6158 | 0.9996 | |
| AAEL012618 | | 0.2162 | 5.3328 | 0.6159 | 0.9996 | |
| AAEL023691 | NA | -0.4416 | 7.1217 | 0.6159 | 0.9996 | NA |
| AAEL008126 | GPRCIR | -0.7403 | 0.2523 | 0.6159 | 0.9996 | GPCR Latrophilin Family [Source:VB Community Annotation] |
| AAEL018186 | | 0.1803 | 3.4825 | 0.6161 | 0.9996 | Phosphatidylinositol 3-kinase [Source:UniProtKB/TrEMBL;Acc:A0A1S4G7T1] |
| AAEL014943 | | -0.1720 | 7.0777 | 0.6164 | 0.9996 | |
| AAEL000384 | | -0.3368 | 1.2607 | 0.6167 | 0.9996 | vesicular acetylcholine transporter [Source:VB Community Annotation] |
| AAEL002524 | CTL24 | -0.3615 | 6.6096 | 0.6167 | 0.9996 | C-Type Lectin (CTL). [Source:VB Community Annotation] |
| AAEL005879 | | -0.1763 | 5.0748 | 0.6169 | 0.9996 | vinculin [Source:VB Community Annotation] |
| AAEL008740 | | 0.1527 | 7.5743 | 0.6170 | 0.9996 | synaptic glycoprotein sc2 [Source:VB Community Annotation] |
| AAEL013729 | | -0.4452 | 6.4972 | 0.6171 | 0.9996 | myotonin-protein kinase [Source:VB Community Annotation] |
| AAEL012226 | | -0.2447 | 4.7476 | 0.6172 | 0.9996 | |
| AAEL019645 | NA | 0.2260 | 2.4096 | 0.6172 | 0.9996 | NA |
| AAEL024540 | NA | -0.2599 | 3.6476 | 0.6173 | 0.9996 | NA |

| | | | | | | |
|---|---|---|---|---|---|---|
| AAEL008424 | | 0.6215 | 1.5024 | 0.6173 | 0.9996 | sodium/chloride dependent amino acid transporter [Source:VB Community Annotation] |
| AAEL003423 | | -0.2141 | 9.1968 | 0.6173 | 0.9996 | NADH dehydrogenase, putative [Source:VB Community Annotation] |
| AAEL024258 | NA | -0.2151 | 3.3708 | 0.6173 | 0.9996 | NA |
| AAEL019657 | NA | 0.1247 | 3.5413 | 0.6174 | 0.9996 | NA |
| AAEL000753 | | 0.2203 | 2.1741 | 0.6174 | 0.9996 | |
| AAEL012147 | | 0.1496 | 2.9994 | 0.6178 | 0.9996 | |
| AAEL009722 | | 0.4281 | 6.9047 | 0.6178 | 0.9996 | clip-domain serine protease, putative [Source:VB Community Annotation] |
| AAEL028205 | NA | 0.3687 | 1.4188 | 0.6178 | 0.9996 | NA |
| AAEL006224 | | -0.1850 | 6.3802 | 0.6179 | 0.9996 | short-chain dehydrogenase [Source:VB Community Annotation] |
| AAEL021426 | NA | 0.4526 | -0.7033 | 0.6179 | 0.9996 | NA |
| AAEL012969 | | 0.1668 | 4.1632 | 0.6179 | 0.9996 | |
| AAEL007483 | | 1.1281 | 0.3939 | 0.6179 | 0.9996 | |
| AAEL007993 | CLIPB27 | -0.7703 | 0.1735 | 0.6179 | 0.9996 | Clip-Domain Serine Protease family B. [Source:VB Community Annotation] |
| AAEL020013 | NA | 0.1216 | 3.6690 | 0.6183 | 0.9996 | NA |
| AAEL023523 | NA | 0.2136 | 3.9308 | 0.6186 | 0.9996 | NA |
| AAEL024849 | NA | -0.3508 | 6.9075 | 0.6188 | 0.9996 | NA |
| AAEL020169 | NA | 0.4770 | 0.2048 | 0.6188 | 0.9996 | NA |
| AAEL014226 | | -0.3103 | 7.4885 | 0.6189 | 0.9996 | |
| AAEL002202 | | 0.5090 | -0.1160 | 0.6190 | 0.9996 | |
| AAEL009052 | | -0.3222 | 2.9539 | 0.6192 | 0.9996 | |
| AAEL005792 | CLIPE8 | -0.5301 | 2.2310 | 0.6193 | 0.9996 | Clip-Domain Serine Protease family E. Protease homologue. [Source:VB Community Annotation] |
| AAEL022497 | NA | -0.1219 | 6.2656 | 0.6194 | 0.9996 | NA |
| AAEL021616 | NA | -0.3625 | 0.6460 | 0.6194 | 0.9996 | NA |
| AAEL019844 | NA | -0.8942 | 3.0760 | 0.6195 | 0.9996 | NA |
| AAEL000427 | | -0.2021 | 4.8688 | 0.6197 | 0.9996 | |
| AAEL025436 | NA | -0.2184 | 2.9747 | 0.6197 | 0.9996 | NA |
| AAEL009342 | | 0.1725 | 4.0897 | 0.6197 | 0.9996 | |
| AAEL004783 | Oda | -0.2038 | 9.6904 | 0.6197 | 0.9996 | Ornithine decarboxylase antizyme (ODC-Az) [Source:VB Community Annotation] |
| AAEL018746 | aae-mir-27 | -0.3560 | 1.6839 | 0.6197 | 0.9996 | |
| AAEL010636 | | 0.5390 | -2.8128 | 0.6198 | 0.9996 | |
| AAEL008146 | | -0.2163 | 4.7958 | 0.6198 | 0.9996 | zinc transporter [Source:VB Community Annotation] |
| AAEL000476 | | 0.3730 | 0.5912 | 0.6199 | 0.9996 | |
| AAEL011116 | | 0.3503 | 10.0938 | 0.6199 | 0.9996 | 14-3-3 protein sigma, gamma, zeta, beta/alpha [Source:VB Community Annotation] |
| AAEL011011 | | 0.3228 | 1.2177 | 0.6200 | 0.9996 | lipase [Source:VB Community Annotation] |
| AAEL019930 | NA | -0.1678 | 4.6537 | 0.6201 | 0.9996 | NA |
| AAEL017987 | | 1.1429 | 0.2461 | 0.6201 | 0.9996 | |
| AAEL013985 | | -0.3571 | 3.7646 | 0.6204 | 0.9996 | DEAD box ATP-dependent RNA helicase [Source:VB Community Annotation] |
| AAEL014578 | | 0.1469 | 6.0658 | 0.6204 | 0.9996 | ssm4 protein [Source:VB Community Annotation] |
| AAEL002959 | | 0.4940 | 1.8238 | 0.6207 | 0.9996 | brain chitinase and chia [Source:VB Community Annotation] |
| AAEL004710 | | -0.2801 | 3.6017 | 0.6208 | 0.9996 | spingomyelin synthetase [Source:VB Community Annotation] |

| | | | | | | |
|---|---|---|---|---|---|---|
| AAEL006319 | | -0.5272 | -0.8786 | 0.6212 | 0.9996 | |
| AAEL007409 | | 0.1655 | 2.5148 | 0.6212 | 0.9996 | xylosyltransferase [Source:VB Community Annotation] |
| AAEL007494 | | 0.1779 | 7.6655 | 0.6212 | 0.9996 | calcineurin b subunit [Source:VB Community Annotation] |
| AAEL013968 | | -0.2520 | 5.0625 | 0.6213 | 0.9996 | |
| AAEL000760 | CLIPB30 | -0.2845 | 2.4932 | 0.6214 | 0.9996 | Clip-Domain Serine Protease  family B. [Source:VB Community Annotation] |
| AAEL021323 | NA | -0.2428 | 4.9598 | 0.6214 | 0.9996 | NA |
| AAEL011197 | | 0.1796 | 8.8937 | 0.6215 | 0.9996 | actin [Source:VB Community Annotation] |
| AAEL002345 | | 0.1567 | 4.3371 | 0.6217 | 0.9996 | |
| AAEL026290 | NA | -0.7589 | 2.4180 | 0.6218 | 0.9996 | NA |
| AAEL014945 | | -0.4693 | 0.9989 | 0.6219 | 0.9996 | |
| AAEL020110 | NA | -0.4054 | 1.9880 | 0.6219 | 0.9996 | NA |
| AAEL001250 | | -0.1226 | 3.8957 | 0.6219 | 0.9996 | |
| AAEL013535 | | 0.3811 | 11.4908 | 0.6219 | 0.9996 | phosrestin ii (arrestin a) (arrestin 1) [Source:VB Community Annotation] |
| AAEL002766 | gatA | 0.1734 | 2.6309 | 0.6219 | 0.9996 | Glutamyl-tRNA(Gln) amidotransferase subunit A, mitochondrial [Source:UniProtKB/Swiss-Prot;Acc:Q17H91] |
| AAEL002819 | | -0.1429 | 4.5445 | 0.6221 | 0.9996 | |
| AAEL006150 | | 0.1281 | 5.2602 | 0.6221 | 0.9996 | brca1-associated protein (brap2) [Source:VB Community Annotation] |
| AAEL013045 | | 0.2280 | 1.8522 | 0.6222 | 0.9996 | exosome complex exonuclease RRP41, putative [Source:VB Community Annotation] |
| AAEL008242 | | -0.1680 | 3.6844 | 0.6225 | 0.9996 | hook protein [Source:VB Community Annotation] |
| AAEL026093 | NA | 0.7443 | 3.5465 | 0.6225 | 0.9996 | NA |
| AAEL019825 | NA | -0.3037 | 5.9031 | 0.6226 | 0.9996 | NA |
| AAEL015416 | | -0.7282 | 1.4277 | 0.6228 | 0.9996 | |
| AAEL011179 | | -0.9534 | 1.5515 | 0.6228 | 0.9996 | ring finger protein [Source:VB Community Annotation] |
| AAEL018201 | | 0.2246 | 3.5713 | 0.6229 | 0.9996 | |
| AAEL014579 | | 0.3521 | 1.7604 | 0.6229 | 0.9996 | trypsin, putative [Source:VB Community Annotation] |
| AAEL012412 | | -0.8864 | 3.8461 | 0.6229 | 0.9996 | slit protein [Source:VB Community Annotation] |
| AAEL019892 | NA | 0.2154 | 3.0888 | 0.6230 | 0.9996 | NA |
| AAEL003864 | | -0.2822 | 3.9551 | 0.6231 | 0.9996 | |
| AAEL013143 | | 0.4045 | 3.8324 | 0.6232 | 0.9996 | |
| AAEL024784 | NA | -0.5169 | -0.2938 | 0.6232 | 0.9996 | NA |
| AAEL004378 | | -0.1042 | 8.3078 | 0.6233 | 0.9996 | eukaryotic translation initiation factor 1A (eIF-1A) [Source:VB Community Annotation] |
| AAEL020853 | NA | -0.7754 | -0.2731 | 0.6235 | 0.9996 | NA |
| AAEL012873 | | -0.1611 | 3.4392 | 0.6237 | 0.9996 | angiotensin-converting enzyme (dipeptidyl carboxypeptidase [Source:VB Community Annotation] |
| AAEL011421 | | 0.1352 | 5.3293 | 0.6238 | 0.9996 | multiple inositol polyphosphate phosphatase [Source:VB Community Annotation] |
| AAEL009765 | | 0.1939 | 4.7363 | 0.6239 | 0.9996 | choline/ethanolamine kinase [Source:VB Community Annotation] |
| AAEL007587 | | -0.3199 | 6.4599 | 0.6240 | 0.9996 | |
| AAEL011221 | | -0.1187 | 7.4045 | 0.6243 | 0.9996 | nmda receptor glutamate-binding chain [Source:VB Community Annotation] |
| AAEL008857 | | 0.7315 | -0.4685 | 0.6245 | 0.9996 | deoxyribonuclease I, putative [Source:VB Community Annotation] |
| AAEL008679 | | -0.3615 | 5.2865 | 0.6245 | 0.9996 | Alpha-tubulin N-acetyltransferase [Source:UniProtKB/Swiss-Prot;Acc:Q16Y34] |
| AAEL002469 | | -0.6976 | 4.0793 | 0.6246 | 0.9996 | endophilin a, [Source:VB Community Annotation] |
| AAEL008115 | | -0.2322 | 2.0297 | 0.6246 | 0.9996 | |

| | | | | | | |
|---|---|---|---|---|---|---|
| AAEL012117 | | -0.4580 | 5.4050 | 0.6246 | 0.9996 | mitochondrial carrier protein [Source:VB Community Annotation] |
| AAEL004169 | | -0.8464 | -1.4404 | 0.6247 | 0.9996 | |
| AAEL011696 | | -0.2296 | 4.2728 | 0.6248 | 0.9996 | |
| AAEL006642 | | -0.1802 | 10.6922 | 0.6248 | 0.9996 | Tubulin alpha chain [Source:UniProtKB/TrEMBL;Acc:Q1HR53] |
| AAEL024618 | NA | 0.3040 | 3.1649 | 0.6248 | 0.9996 | NA |
| AAEL010339 | | -0.1744 | 3.9757 | 0.6248 | 0.9996 | |
| AAEL023547 | NA | 0.1664 | 5.4888 | 0.6249 | 0.9996 | NA |
| AAEL019660 | NA | 0.2486 | 4.8260 | 0.6251 | 0.9996 | NA |
| AAEL001130 | | 0.5030 | -1.0555 | 0.6251 | 0.9996 | Alpha-amylase [Source:UniProtKB/TrEMBL;Acc:Q17M63] |
| AAEL008498 | | -0.7678 | -1.5422 | 0.6251 | 0.9996 | cohesin-subunit, putative [Source:VB Community Annotation] |
| AAEL027465 | NA | -0.1681 | 5.8089 | 0.6254 | 0.9996 | NA |
| AAEL002317 | InR | -1.0183 | 2.7133 | 0.6255 | 0.9996 | Insulin-like receptor Precursor (MIR)(EC 2.7.10.1) [Source:VB Community Annotation] |
| AAEL007902 | | -0.6328 | 2.1380 | 0.6255 | 0.9996 | |
| AAEL003093 | | 0.5098 | 1.7281 | 0.6256 | 0.9996 | elongase, putative [Source:VB Community Annotation] |
| AAEL025718 | NA | -0.1258 | 3.7426 | 0.6256 | 0.9996 | NA |
| AAEL013605 | | 0.4594 | -0.3096 | 0.6258 | 0.9996 | |
| AAEL014041 | | -0.1377 | 3.9317 | 0.6258 | 0.9996 | |
| AAEL010913 | | 0.1824 | 2.3339 | 0.6259 | 0.9996 | structural maintenance of chromosomes 5 smc5 [Source:VB Community Annotation] |
| AAEL022884 | NA | -0.3709 | 2.5340 | 0.6259 | 0.9996 | NA |
| AAEL006177 | | -0.1246 | 5.0673 | 0.6259 | 0.9996 | histone acetyltransferase type b catalytic subunit [Source:VB Community Annotation] |
| AAEL010641 | APG7A | -0.1411 | 5.1694 | 0.6260 | 0.9996 | autophagy related gene [Source:VB Community Annotation] |
| AAEL019794 | NA | -0.6453 | -0.3379 | 0.6261 | 0.9996 | NA |
| AAEL017115 | | -0.7041 | -0.4229 | 0.6261 | 0.9996 | |
| AAEL001703 | JHA15 | -0.8366 | 6.9085 | 0.6261 | 0.9996 | juvenile hormone-regulated chymotrypsin-like serine protease [Source:VB Community Annotation] |
| AAEL013980 | | -0.2018 | 7.9928 | 0.6262 | 0.9996 | glutaredoxin, putative [Source:VB Community Annotation] |
| AAEL010727 | | -0.1095 | 5.2518 | 0.6262 | 0.9996 | glutaminyl-peptide cyclotransferase [Source:VB Community Annotation] |
| AAEL014045 | | 0.4068 | 5.5735 | 0.6262 | 0.9996 | allantoicase [Source:VB Community Annotation] |
| AAEL002287 | | -0.1754 | 4.7118 | 0.6262 | 0.9996 | trans-prenyltransferase [Source:VB Community Annotation] |
| AAEL027256 | NA | -0.3789 | 0.8507 | 0.6264 | 0.9996 | NA |
| AAEL025404 | NA | -0.7352 | 1.7815 | 0.6265 | 0.9996 | NA |
| AAEL019807 | NA | 0.1557 | 2.7566 | 0.6265 | 0.9996 | NA |
| AAEL006780 | | 0.1839 | 3.5015 | 0.6265 | 0.9996 | Palmitoyltransferase [Source:UniProtKB/TrEMBL;Acc:A0A1S4FEL9] |
| AAEL018216 | | 0.4575 | -0.1603 | 0.6266 | 0.9996 | |
| AAEL006056 | | -0.4564 | 0.8966 | 0.6267 | 0.9996 | nuclear protein localization [Source:VB Community Annotation] |
| AAEL014853 | | 0.6844 | -1.8821 | 0.6267 | 0.9996 | otoferlin [Source:VB Community Annotation] |
| AAEL019459 | NA | -0.1311 | 5.1753 | 0.6267 | 0.9996 | NA |
| AAEL007090 | | 0.2497 | 3.4682 | 0.6268 | 0.9996 | 4-nitrophenylphosphatase [Source:VB Community Annotation] |
| AAEL001433 | | 0.1607 | 5.2915 | 0.6269 | 0.9996 | fgf receptor activating protein [Source:VB Community Annotation] |
| AAEL003781 | | 0.2558 | 2.9924 | 0.6269 | 0.9996 | zinc carboxypeptidase [Source:VB Community Annotation] |
| AAEL011779 | | 0.4834 | 1.0525 | 0.6271 | 0.9996 | |

| | | | | | | |
|---|---|---|---|---|---|---|
| AAEL016064 | tRNA-Pro | -0.4774 | 0.1748 | 0.6271 | 0.9996 | |
| AAEL024663 | NA | 0.3411 | 0.6999 | 0.6271 | 0.9996 | NA |
| AAEL000811 | GPRMTH7 | 0.3168 | 1.1000 | 0.6271 | 0.9996 | GPCR Methuselah Family [Source:VB Community Annotation] |
| AAEL023286 | NA | 0.1865 | 4.1935 | 0.6273 | 0.9996 | NA |
| AAEL015073 | | 0.3903 | 0.7390 | 0.6274 | 0.9996 | |
| AAEL000574 | | 0.1392 | 6.0657 | 0.6275 | 0.9996 | |
| AAEL027346 | NA | -0.8207 | 1.0665 | 0.6276 | 0.9996 | NA |
| AAEL002891 | | 0.2356 | 3.5881 | 0.6276 | 0.9996 | |
| AAEL019477 | NA | 0.2579 | 4.9778 | 0.6277 | 0.9996 | NA |
| AAEL001344 | | -0.2140 | 1.9103 | 0.6278 | 0.9996 | pair-rule protein odd-paired [Source:VB Community Annotation] |
| AAEL003409 | mRpL15 | -0.2007 | 5.6144 | 0.6278 | 0.9996 | 39S ribosomal protein L15, mitochondrial [Source:VB Community Annotation] |
| AAEL014709 | | 0.1248 | 5.7788 | 0.6279 | 0.9996 | methionine-tRNA synthetase [Source:VB Community Annotation] |
| AAEL008591 | | 0.2513 | 4.5207 | 0.6280 | 0.9996 | zinc finger protein, putative [Source:VB Community Annotation] |
| AAEL014361 | | 0.2766 | 6.7670 | 0.6284 | 0.9996 | amidase [Source:VB Community Annotation] |
| AAEL000678 | | -0.5635 | -2.6133 | 0.6285 | 0.9996 | alpha-amylase [Source:VB Community Annotation] |
| AAEL020482 | NA | -0.1954 | 5.9913 | 0.6285 | 0.9996 | NA |
| AAEL006502 | | -0.1743 | 4.7629 | 0.6286 | 0.9996 | |
| AAEL010139 | | -0.4876 | 5.0816 | 0.6287 | 0.9996 | serine protease, putative [Source:VB Community Annotation] |
| AAEL002173 | | 0.2843 | 1.8470 | 0.6288 | 0.9996 | |
| AAEL010831 | | -0.1245 | 3.9670 | 0.6288 | 0.9996 | |
| AAEL023524 | NA | -0.6159 | 0.4013 | 0.6288 | 0.9996 | NA |
| AAEL008468 | | 0.3534 | 2.2656 | 0.6288 | 0.9996 | cysteine synthase [Source:VB Community Annotation] |
| AAEL027213 | NA | 0.1593 | 4.6549 | 0.6290 | 0.9996 | NA |
| AAEL013470 | | 0.1396 | 4.9987 | 0.6291 | 0.9996 | |
| AAEL004366 | | 0.1722 | 3.0270 | 0.6291 | 0.9996 | |
| AAEL025398 | NA | -0.3415 | 2.0083 | 0.6293 | 0.9996 | NA |
| AAEL020014 | NA | 0.2377 | 2.2147 | 0.6295 | 0.9996 | NA |
| AAEL010954 | | -0.1886 | 5.2135 | 0.6295 | 0.9996 | |
| AAEL000953 | | 0.2972 | 3.1372 | 0.6296 | 0.9996 | |
| AAEL014846 | inx1 | -0.5312 | 3.4986 | 0.6298 | 0.9996 | Innexin [Source:UniProtKB/TrEMBL;Acc:Q16FA3] |
| AAEL010903 | | 0.1564 | 4.2005 | 0.6299 | 0.9996 | |
| AAEL020442 | NA | -0.4646 | -0.5811 | 0.6299 | 0.9996 | NA |
| AAEL004052 | | 0.1583 | 4.9945 | 0.6299 | 0.9996 | |
| AAEL022080 | NA | -0.3137 | 4.2655 | 0.6299 | 0.9996 | NA |
| AAEL026142 | NA | -0.1746 | 4.3884 | 0.6301 | 0.9996 | NA |
| AAEL011350 | | -0.1559 | 6.6371 | 0.6301 | 0.9996 | |
| AAEL014273 | | 0.1377 | 5.1287 | 0.6301 | 0.9996 | |
| AAEL027903 | NA | -0.3803 | 2.0059 | 0.6302 | 0.9996 | NA |
| AAEL006511 | | -0.1640 | 10.2250 | 0.6302 | 0.9996 | ubiquitin [Source:VB Community Annotation] |
| AAEL023485 | NA | 0.4751 | -2.7480 | 0.6302 | 0.9996 | NA |

| | | | | | | |
|---|---|---|---|---|---|---|
| AAEL022573 | NA | -0.2486 | 4.5846 | 0.6303 | 0.9996 | NA |
| AAEL024486 | NA | 0.9109 | -0.0441 | 0.6304 | 0.9996 | NA |
| AAEL001140 | | -0.1460 | 6.0986 | 0.6304 | 0.9996 | MutS protein homolog 4 [Source:VB Community Annotation] |
| AAEL021812 | NA | 0.1882 | 4.2574 | 0.6305 | 0.9996 | NA |
| AAEL003467 | | 0.2355 | 6.3570 | 0.6306 | 0.9996 | |
| AAEL024326 | NA | 0.3856 | 1.9025 | 0.6306 | 0.9996 | NA |
| AAEL024204 | NA | 0.1101 | 4.7386 | 0.6307 | 0.9996 | NA |
| AAEL005802 | | -0.1425 | 5.1605 | 0.6307 | 0.9996 | structural maintenance of chromosomes 1 smc1 [Source:VB Community Annotation] |
| AAEL013900 | | -0.6645 | -1.3425 | 0.6307 | 0.9996 | cAMP and cAMP-inhibited cgmp 3,5-cyclic phosphodiesterase [Source:VB Community Annotation] |
| AAEL020095 | NA | 0.1564 | 3.9066 | 0.6309 | 0.9996 | NA |
| AAEL004328 | | 0.3110 | 0.7921 | 0.6309 | 0.9996 | origin recognition complex subunit [Source:VB Community Annotation] |
| AAEL001039 | | 0.1681 | 4.0356 | 0.6310 | 0.9996 | metaxin [Source:VB Community Annotation] |
| AAEL022392 | NA | 0.2722 | 0.8015 | 0.6312 | 0.9996 | NA |
| AAEL019563 | NA | -0.6783 | 2.1210 | 0.6315 | 0.9996 | NA |
| AAEL019512 | NA | 0.1354 | 5.7307 | 0.6316 | 0.9996 | NA |
| AAEL011737 | | 0.3380 | 2.8851 | 0.6316 | 0.9996 | |
| AAEL008837 | | 0.7784 | 2.6224 | 0.6317 | 0.9996 | ubiquitin-conjugating enzyme E2r [Source:VB Community Annotation] |
| AAEL028022 | NA | -0.3848 | 0.0813 | 0.6320 | 0.9996 | NA |
| AAEL009767 | | 0.1568 | 5.4787 | 0.6320 | 0.9996 | |
| AAEL019526 | NA | -0.4331 | 7.5237 | 0.6321 | 0.9996 | NA |
| AAEL019939 | NA | -0.1949 | 4.9150 | 0.6322 | 0.9996 | NA |
| AAEL018324 | | -0.3768 | 6.5124 | 0.6322 | 0.9996 | |
| AAEL015151 | | -0.2160 | 5.2239 | 0.6322 | 0.9996 | acid phosphatase [Source:VB Community Annotation] |
| AAEL024613 | NA | -0.3247 | 1.7266 | 0.6323 | 0.9996 | NA |
| AAEL011067 | | 0.2969 | 3.6122 | 0.6323 | 0.9996 | tubulin-specific chaperone, putative [Source:VB Community Annotation] |
| AAEL004385 | | 0.1885 | 2.9872 | 0.6324 | 0.9996 | UGA suppressor tRNA-associated antigenic protein, putative [Source:VB Community Annotation] |
| AAEL008725 | | 0.1370 | 4.9155 | 0.6326 | 0.9996 | |
| AAEL010061 | | -0.5669 | 1.9567 | 0.6329 | 0.9996 | |
| AAEL027794 | NA | -0.4734 | -0.5134 | 0.6329 | 0.9996 | NA |
| AAEL004736 | | 0.3022 | 0.8799 | 0.6330 | 0.9996 | |
| AAEL006728 | | 0.1317 | 5.1985 | 0.6330 | 0.9996 | ubiquitin-conjugating enzyme E2 c [Source:VB Community Annotation] |
| AAEL022457 | NA | 0.2748 | 2.5624 | 0.6331 | 0.9996 | NA |
| AAEL006276 | | 0.5129 | -2.4688 | 0.6334 | 0.9996 | maltose phosphorylase [Source:VB Community Annotation] |
| AAEL026955 | NA | -0.5567 | 7.7947 | 0.6335 | 0.9996 | NA |
| AAEL007868 | | -0.2067 | 10.9861 | 0.6337 | 0.9996 | ubiquinol-cytochrome c reductase complex 14 kd protein [Source:VB Community Annotation] |
| AAEL014891 | CYP6P12 | 0.1746 | 5.2412 | 0.6338 | 0.9996 | cytochrome P450 [Source:VB Community Annotation] |
| AAEL018700 | | -0.5519 | 3.9407 | 0.6338 | 0.9996 | |
| AAEL019474 | NA | -0.2416 | 6.3656 | 0.6338 | 0.9996 | NA |
| AAEL000025 | | -0.6005 | -1.9222 | 0.6339 | 0.9996 | phosphoenolpyruvate carboxykinase [Source:VB Community Annotation] |
| AAEL013554 | CYP4J14 | 0.2967 | 2.4669 | 0.6341 | 0.9996 | cytochrome P450 [Source:VB Community Annotation] |

| | | | | | | |
|---|---|---|---|---|---|---|
| AAEL014040 | | -0.2111 | 2.4897 | 0.6341 | 0.9996 | |
| AAEL015019 | | -0.4575 | 1.6647 | 0.6341 | 0.9996 | |
| AAEL001526 | | -0.6511 | 3.8751 | 0.6342 | 0.9996 | zinc finger protein [Source:VB Community Annotation] |
| AAEL007990 | | -0.5975 | 3.5533 | 0.6342 | 0.9996 | SH3 domain-binding glutamic acid-rich-like protein [Source:UniProtKB/TrEMBL;Acc:A0A1S4FIE8] |
| AAEL027361 | NA | -0.1608 | 4.1785 | 0.6343 | 0.9996 | NA |
| AAEL004195 | | 0.1459 | 7.5380 | 0.6345 | 0.9996 | membrane associated progesterone receptor [Source:VB Community Annotation] |
| AAEL008031 | | -0.2644 | 3.9499 | 0.6345 | 0.9996 | carbonic anhydrase [Source:VB Community Annotation] |
| AAEL011352 | | -0.4198 | 0.1953 | 0.6346 | 0.9996 | |
| AAEL002810 | | 0.2886 | 1.7397 | 0.6346 | 0.9996 | DNA replication licensing factor MCM5 [Source:VB Community Annotation] |
| AAEL023159 | NA | -0.7223 | 2.2960 | 0.6346 | 0.9996 | NA |
| AAEL003628 | CLIPB38 | -0.3563 | 3.2224 | 0.6347 | 0.9996 | Clip-Domain Serine Protease  family B. [Source:VB Community Annotation] |
| AAEL004435 | | 0.1594 | 5.8795 | 0.6349 | 0.9996 | kynurenine aminotransferase [Source:VB Community Annotation] |
| AAEL000239 | | -0.1721 | 5.9250 | 0.6349 | 0.9996 | clathrin coat assembly protein [Source:VB Community Annotation] |
| AAEL000708 | | -0.2183 | 3.5248 | 0.6353 | 0.9996 | hiv-1 rev binding protein [Source:VB Community Annotation] |
| AAEL001765 | | -0.3899 | 2.8684 | 0.6353 | 0.9996 | |
| AAEL004648 | | 0.3965 | -0.1657 | 0.6353 | 0.9996 | |
| AAEL026217 | NA | -0.4379 | 7.6309 | 0.6354 | 0.9996 | NA |
| AAEL019852 | NA | -0.3408 | 3.7688 | 0.6354 | 0.9996 | NA |
| AAEL006753 | | 0.2215 | 3.5576 | 0.6355 | 0.9996 | |
| AAEL014762 | | 0.1608 | 6.7183 | 0.6355 | 0.9996 | zinc transporter [Source:VB Community Annotation] |
| AAEL021462 | NA | 0.5361 | -0.3655 | 0.6356 | 0.9996 | NA |
| AAEL001177 | | 0.1051 | 5.6680 | 0.6356 | 0.9996 | signal recognition particle, 14kD, putative [Source:VB Community Annotation] |
| AAEL022006 | NA | 0.4697 | -1.2451 | 0.6359 | 0.9996 | NA |
| AAEL006552 | | -0.1599 | 6.8908 | 0.6362 | 0.9996 | WD-repeat protein [Source:VB Community Annotation] |
| AAEL021027 | NA | 0.3590 | 3.9624 | 0.6363 | 0.9996 | NA |
| AAEL011105 | | -0.1694 | 6.9556 | 0.6368 | 0.9996 | adducin [Source:VB Community Annotation] |
| AAEL007063 | | 0.6014 | -1.9487 | 0.6369 | 0.9996 | lipase [Source:VB Community Annotation] |
| AAEL002701 | | -0.1500 | 6.0368 | 0.6370 | 0.9996 | mannosyltransferase [Source:VB Community Annotation] |
| AAEL004729 | | 0.2543 | 4.5741 | 0.6371 | 0.9996 | |
| AAEL011222 | SCRB5 | -0.1290 | 6.9141 | 0.6372 | 0.9996 | Class B Scavenger Receptor (CD36 domain). [Source:VB Community Annotation |
| AAEL012418 | | 0.5539 | 0.7036 | 0.6372 | 0.9996 | deoxyribonuclease ii [Source:VB Community Annotation] |
| AAEL009764 | | 0.1562 | 4.9962 | 0.6372 | 0.9996 | xaa-pro aminopeptidase [Source:VB Community Annotation] |
| AAEL013689 | | -0.7875 | 0.9488 | 0.6374 | 0.9996 | neuronal calcium sensor, putative [Source:VB Community Annotation] |
| AAEL029008 | NA | -0.7727 | -0.6083 | 0.6375 | 0.9996 | NA |
| AAEL017338 | | -0.3203 | 4.5902 | 0.6375 | 0.9996 | Mitogen-activated protein kinase kinase kinase kinase [Source:UniProtKB/TrEMBL;Acc:A0A1S4G5Q8] |
| AAEL027034 | NA | 0.1966 | 5.0354 | 0.6376 | 0.9996 | NA |
| AAEL004088 | | 0.1852 | 6.9632 | 0.6376 | 0.9996 | aldo-keto reductase [Source:VB Community Annotation] |
| AAEL013199 | | 0.3283 | 2.9837 | 0.6377 | 0.9996 | DNA-J, putative [Source:VB Community Annotation] |
| AAEL021836 | NA | 0.2039 | 5.2282 | 0.6379 | 0.9996 | NA |
| AAEL009198 | | -0.2199 | 5.9054 | 0.6379 | 0.9996 | |

| | | | | | | |
|---|---|---|---|---|---|---|
| AAEL007505 | | -0.7732 | -0.6951 | 0.6379 | 0.9996 | iroquois-class homeodomain protein irx [Source:VB Community Annotation] |
| AAEL025048 | NA | -0.1863 | 3.4403 | 0.6380 | 0.9996 | NA |
| AAEL003190 | | 0.7990 | -0.5143 | 0.6381 | 0.9996 | |
| AAEL007500 | | -0.2630 | 4.9133 | 0.6381 | 0.9996 | |
| AAEL002068 | | 0.1549 | 6.3186 | 0.6383 | 0.9996 | mitochondrial processing peptidase alpha subunit [Source:VB Community Annotation] |
| AAEL003922 | | 0.2043 | 2.6556 | 0.6384 | 0.9996 | |
| AAEL013942 | | 0.1400 | 4.6081 | 0.6384 | 0.9996 | aminoadipate-semialdehyde dehydrogenase [Source:VB Community Annotation] |
| AAEL008163 | | 0.7186 | -1.1372 | 0.6384 | 0.9996 | protease m1 zinc metalloprotease [Source:VB Community Annotation] |
| AAEL004359 | | 0.1517 | 6.4981 | 0.6384 | 0.9996 | |
| AAEL000311 | | -0.3345 | 6.7466 | 0.6384 | 0.9996 | |
| AAEL022867 | NA | -0.1432 | 6.8723 | 0.6385 | 0.9996 | NA |
| AAEL005773 | | 0.4221 | 0.1151 | 0.6388 | 0.9996 | |
| AAEL000770 | | -0.3601 | 5.8847 | 0.6388 | 0.9996 | platelet-activating factor acetylhydrolase isoform 1b alpha subunit [Source:VB Community Annotation] |
| AAEL001005 | | -0.1496 | 8.7167 | 0.6388 | 0.9996 | calreticulin [Source:VB Community Annotation] |
| AAEL006748 | | -0.1303 | 5.7064 | 0.6390 | 0.9996 | ss-DNA binding protein 12RNP2 precursor, putative [Source:VB Community Annotation] |
| AAEL005763 | | -0.2119 | 7.4488 | 0.6391 | 0.9996 | lysosomal alpha-mannosidase (mannosidase alpha class 2b member 1) [Source:VB Community Annotation] |
| AAEL028001 | NA | 0.7522 | -1.3417 | 0.6391 | 0.9996 | NA |
| AAEL000037 | CLIPB35 | -0.3806 | 6.1191 | 0.6392 | 0.9996 | Clip-Domain Serine Protease family B. [Source:VB Community Annotation] |
| AAEL006066 | | 0.1847 | 3.5846 | 0.6394 | 0.9996 | peptidyl-tRNA hydrolase [Source:VB Community Annotation] |
| AAEL003561 | | 0.5203 | 1.4885 | 0.6394 | 0.9996 | phospholipase A2, putative [Source:VB Community Annotation] |
| AAEL002869 | | 0.1567 | 4.0832 | 0.6395 | 0.9996 | |
| AAEL011361 | | -0.1264 | 5.8287 | 0.6395 | 0.9996 | |
| AAEL017004 | | 0.1310 | 4.4018 | 0.6396 | 0.9996 | |
| AAEL001688 | | 0.2281 | 1.8291 | 0.6396 | 0.9996 | |
| AAEL006191 | | -0.8152 | 1.9590 | 0.6399 | 0.9996 | |
| AAEL021308 | NA | -0.2108 | 2.2931 | 0.6401 | 0.9996 | NA |
| AAEL002378 | | -0.4021 | 4.4520 | 0.6401 | 0.9996 | Carboxylic ester hydrolase [Source:UniProtKB/TrEMBL;Acc:A0A1S4F1J5] |
| AAEL022720 | NA | -0.2718 | 2.7429 | 0.6402 | 0.9996 | NA |
| AAEL001032 | | 0.1935 | 3.3786 | 0.6402 | 0.9996 | |
| AAEL010879 | | 0.1804 | 5.0989 | 0.6403 | 0.9996 | |
| AAEL024225 | NA | -0.3128 | 1.1003 | 0.6403 | 0.9996 | NA |
| AAEL024359 | NA | -0.3849 | 3.7614 | 0.6403 | 0.9996 | NA |
| AAEL004249 | | 0.3207 | 5.8729 | 0.6405 | 0.9996 | |
| AAEL014450 | | 0.1134 | 8.0447 | 0.6407 | 0.9996 | peptidyl-glycine alpha-amidating monooxygenase [Source:VB Community Annotation] |
| AAEL028144 | NA | -0.3540 | 4.2355 | 0.6408 | 0.9996 | NA |
| AAEL001106 | | 0.2211 | 4.1491 | 0.6408 | 0.9996 | |
| AAEL019408 | NA | 0.1311 | 7.8287 | 0.6408 | 0.9996 | NA |
| AAEL002675 | | -0.3707 | 6.0215 | 0.6409 | 0.9996 | Arginase [Source:UniProtKB/TrEMBL;Acc:Q17HI3] |
| AAEL006827 | CYP12F8 | 0.5721 | -0.9896 | 0.6409 | 0.9996 | cytochrome P450 [Source:VB Community Annotation] |
| AAEL019580 | NA | 0.3730 | 2.6341 | 0.6409 | 0.9996 | NA |

| | | | | | | |
|---|---|---|---|---|---|---|
| AAEL010856 | | 0.1527 | 5.4827 | 0.6409 | 0.9996 | dopamine beta hydroxylase [Source:VB Community Annotation] |
| AAEL002578 | | 0.7065 | 1.6568 | 0.6409 | 0.9996 | |
| AAEL010832 | | 0.1852 | 3.6275 | 0.6410 | 0.9996 | Pop5 protein, putative [Source:VB Community Annotation] |
| AAEL008022 | | -0.3367 | 1.3646 | 0.6410 | 0.9996 | homeobox protein [Source:VB Community Annotation] |
| AAEL023104 | NA | -0.7760 | 2.7610 | 0.6413 | 0.9996 | NA |
| AAEL020525 | NA | -0.5254 | 4.0572 | 0.6416 | 0.9996 | NA |
| AAEL000283 | CTLMA16 | 0.6230 | 0.6746 | 0.6416 | 0.9996 | C-Type Lectin (CTL) - mannose binding. [Source:VB Community Annotation] |
| AAEL005850 | HR4 | -0.8055 | -0.9576 | 0.6416 | 0.9996 | Hormone receptor-like in 4 (nuclear receptor) [Source:VB Community Annotation] |
| AAEL010657 | | 0.2292 | 2.5913 | 0.6417 | 0.9996 | |
| AAEL009751 | | -0.2829 | 1.7163 | 0.6417 | 0.9996 | |
| AAEL008058 | | 0.1866 | 4.8297 | 0.6418 | 0.9996 | liprin alpha (lar-interacting protein alpha) (synapse defective protein 2) [Source:VB Community Annotation] |
| AAEL011742 | | -0.1250 | 6.5415 | 0.6420 | 0.9996 | eukaryotic peptide chain release factor subunit [Source:VB Community Annotation] |
| AAEL001412 | | 0.1772 | 4.8173 | 0.6421 | 0.9996 | |
| AAEL019439 | NA | -0.9127 | 1.4296 | 0.6421 | 0.9996 | NA |
| AAEL025934 | NA | -0.6696 | -2.7863 | 0.6422 | 0.9996 | NA |
| AAEL008068 | | 0.2380 | 2.4623 | 0.6423 | 0.9996 | |
| AAEL012325 | | 0.1023 | 4.9739 | 0.6424 | 0.9996 | |
| AAEL013327 | | 0.2130 | 6.2714 | 0.6425 | 0.9996 | |
| AAEL027948 | NA | -0.9909 | 0.4363 | 0.6426 | 0.9996 | NA |
| AAEL006685 | | -0.1316 | 8.9708 | 0.6427 | 0.9996 | G-protein, gamma-subunit, putative [Source:VB Community Annotation] |
| AAEL013107 | | -0.1243 | 4.2999 | 0.6428 | 0.9996 | |
| AAEL010296 | | -0.1425 | 5.1731 | 0.6431 | 0.9996 | carboxylase:pyruvate/acetyl-coa/propionyl-coa [Source:VB Community Annotation] |
| AAEL010619 | | -0.1838 | 3.2554 | 0.6432 | 0.9996 | |
| AAEL026497 | NA | 0.1294 | 5.3163 | 0.6432 | 0.9996 | NA |
| AAEL019573 | NA | 0.7196 | 4.3133 | 0.6435 | 0.9996 | NA |
| AAEL021196 | NA | 0.7753 | -0.7037 | 0.6436 | 0.9996 | NA |
| AAEL024814 | NA | 0.1407 | 3.6779 | 0.6437 | 0.9996 | NA |
| AAEL025091 | NA | 0.1376 | 6.1277 | 0.6438 | 0.9996 | NA |
| AAEL003107 | | -0.8050 | 4.2831 | 0.6439 | 0.9996 | |
| AAEL003125 | | 0.2345 | 4.7772 | 0.6440 | 0.9996 | acyl-coa dehydrogenase [Source:VB Community Annotation] |
| AAEL008758 | Fsn | -0.2329 | 3.9520 | 0.6442 | 0.9996 | F-box/SPRY domain-containing protein 1 [Source:UniProtKB/Swiss-Prot;Acc:Q16XV7] |
| AAEL024905 | NA | -0.5409 | -0.6021 | 0.6443 | 0.9996 | NA |
| AAEL000362 | | -0.4710 | 1.8843 | 0.6443 | 0.9996 | |
| AAEL013237 | | 0.4272 | -0.3739 | 0.6445 | 0.9996 | pom1 [Source:VB Community Annotation] |
| AAEL027443 | NA | 0.5662 | -0.8553 | 0.6445 | 0.9996 | NA |
| AAEL005273 | | -0.3818 | -0.4393 | 0.6446 | 0.9996 | |
| AAEL025577 | NA | 1.0200 | -0.8511 | 0.6447 | 0.9996 | NA |
| AAEL011716 | | 0.8235 | 0.9710 | 0.6449 | 0.9996 | tartan [Source:VB Community Annotation] |
| AAEL006535 | | -0.1254 | 7.3419 | 0.6450 | 0.9996 | |
| AAEL008359 | | 0.6138 | -0.6424 | 0.6451 | 0.9996 | |

| | | | | | | |
|---|---|---|---|---|---|---|
| AAEL019537 | NA | 0.6037 | -1.7954 | 0.6453 | 0.9996 | NA |
| AAEL008154 | | -0.6495 | 2.5162 | 0.6453 | 0.9996 | |
| AAEL012685 | | 0.1866 | 5.6856 | 0.6453 | 0.9996 | |
| AAEL002696 | | 0.3496 | 4.6007 | 0.6453 | 0.9996 | |
| AAEL003043 | | 0.1993 | 3.5046 | 0.6453 | 0.9996 | |
| AAEL008495 | | 0.1924 | 3.1938 | 0.6453 | 0.9996 | |
| AAEL010247 | | 0.3615 | 1.7030 | 0.6456 | 0.9996 | |
| AAEL002322 | | 0.1950 | 3.3760 | 0.6456 | 0.9996 | |
| AAEL010684 | | -0.2807 | 6.5487 | 0.6456 | 0.9996 | Trehalose 6-phosphate phosphatase [Source:UniProtKB/TrEMBL;Acc:A0A1S4FR40] |
| AAEL005680 | | -0.5358 | -0.9265 | 0.6456 | 0.9996 | Odorant receptor [Source:UniProtKB/TrEMBL;Acc:Q179B4] |
| AAEL026437 | NA | -0.3275 | 7.9825 | 0.6457 | 0.9996 | NA |
| AAEL007652 | | 0.1790 | 3.1920 | 0.6460 | 0.9996 | |
| AAEL001233 | CLIPE9 | -0.1874 | 3.0991 | 0.6460 | 0.9996 | Clip-Domain Serine Protease family E. Protease homologue. [Source:VB Community Annotation] |
| AAEL007693 | | -0.4686 | -0.3537 | 0.6460 | 0.9996 | organic anion transporter [Source:VB Community Annotation] |
| AAEL003968 | | 0.1434 | 4.6953 | 0.6461 | 0.9996 | helicase [Source:VB Community Annotation] |
| AAEL009496 | RpS7 | 0.1750 | 11.4563 | 0.6463 | 0.9996 | 40S ribosomal protein S7 [Source:VB Community Annotation] |
| AAEL001439 | mRpL22 | -0.2285 | 6.9472 | 0.6463 | 0.9996 | mitochondrial ribosomal protein, L22, putative [Source:VB Community Annotation] |
| AAEL004865 | | -0.3246 | 9.7071 | 0.6464 | 0.9996 | cyclin g [Source:VB Community Annotation] |
| AAEL014720 | | 0.1317 | 4.0009 | 0.6464 | 0.9996 | |
| AAEL001162 | | 0.2725 | 1.2593 | 0.6465 | 0.9996 | |
| AAEL022200 | NA | 0.3718 | 3.3568 | 0.6465 | 0.9996 | NA |
| AAEL002724 | | -0.6795 | 3.7941 | 0.6469 | 0.9996 | munc13-4 [Source:VB Community Annotation] |
| AAEL012203 | | -0.5242 | 4.9887 | 0.6469 | 0.9996 | |
| AAEL024592 | NA | -0.3987 | 3.1856 | 0.6470 | 0.9996 | NA |
| AAEL018127 | | -0.1311 | 3.6050 | 0.6470 | 0.9996 | |
| AAEL017500 | | -0.6213 | 6.0572 | 0.6471 | 0.9996 | |
| AAEL023993 | NA | -0.5576 | -1.1520 | 0.6471 | 0.9996 | NA |
| AAEL027227 | NA | 0.1994 | 9.0568 | 0.6472 | 0.9996 | NA |
| AAEL010317 | | -0.2859 | 2.9341 | 0.6472 | 0.9996 | DEAD box ATP-dependent RNA helicase [Source:VB Community Annotation] |
| AAEL021557 | NA | 0.2719 | 2.1520 | 0.6472 | 0.9996 | NA |
| AAEL009086 | | -0.2213 | 3.5974 | 0.6473 | 0.9996 | |
| AAEL024906 | NA | -0.7376 | -1.3060 | 0.6473 | 0.9996 | NA |
| AAEL005947 | | 0.1816 | 5.0754 | 0.6473 | 0.9996 | G-rich sequence factor-1, putative [Source:VB Community Annotation] |
| AAEL001917 | | 0.2333 | 4.6636 | 0.6473 | 0.9996 | ribosome biogenesis protein brix [Source:VB Community Annotation] |
| AAEL001813 | | 0.3681 | 3.6635 | 0.6474 | 0.9996 | sodium/solute symporter [Source:VB Community Annotation] |
| AAEL010196 | | 0.9522 | 1.2797 | 0.6477 | 0.9996 | trypsin [Source:VB Community Annotation] |
| AAEL017545 | | 0.1751 | 6.5295 | 0.6477 | 0.9996 | |
| AAEL005581 | | -0.1687 | 4.7821 | 0.6478 | 0.9996 | norepinephrine/norepinephrine transporter [Source:VB Community Annotation] |
| AAEL012247 | | 0.1703 | 3.0816 | 0.6478 | 0.9996 | |
| AAEL009678 | | 0.2545 | 3.3811 | 0.6479 | 0.9996 | golgi-specific brefeldin a-resistance factor [Source:VB Community Annotation] |

| | | | | | | |
|---|---|---|---|---|---|---|
| AAEL024963 | NA | 0.2638 | 0.1105 | 0.6481 | 0.9996 | NA |
| AAEL029013 | NA | -0.2042 | 9.8779 | 0.6481 | 0.9996 | NA |
| AAEL013847 | | -0.4594 | 7.0366 | 0.6483 | 0.9996 | diacylglycerol kinase, zeta, iota [Source:VB Community Annotation] |
| AAEL010817 | | 0.1679 | 2.7573 | 0.6483 | 0.9996 | |
| AAEL002007 | | 0.5821 | 0.0507 | 0.6484 | 0.9996 | protein serine/threonine kinase, putative [Source:VB Community Annotation] |
| AAEL006308 | | -0.1024 | 5.4563 | 0.6485 | 0.9996 | px serine/threonine kinase (pxk) [Source:VB Community Annotation] |
| AAEL014493 | | 0.3730 | 0.5786 | 0.6486 | 0.9996 | aldehyde oxidase [Source:VB Community Annotation] |
| AAEL019488 | NA | 0.3072 | 3.0856 | 0.6486 | 0.9996 | NA |
| AAEL026676 | NA | -0.2169 | 3.3320 | 0.6487 | 0.9996 | NA |
| AAEL025379 | NA | -0.1978 | 6.7848 | 0.6488 | 0.9996 | NA |
| AAEL009608 | mRpS16 | -0.1314 | 5.7542 | 0.6492 | 0.9996 | 28S ribosomal protein S16, mitochondrial [Source:VB Community Annotation] |
| AAEL006915 | | -0.1831 | 5.1524 | 0.6495 | 0.9996 | |
| AAEL009380 | | -0.6122 | 6.7146 | 0.6495 | 0.9996 | phospholipase c beta [Source:VB Community Annotation] |
| AAEL006478 | | 0.1598 | 3.3638 | 0.6496 | 0.9996 | lachesin, putative [Source:VB Community Annotation] |
| AAEL010581 | | -0.1925 | 3.1403 | 0.6497 | 0.9996 | zinc finger protein, putative [Source:VB Community Annotation] |
| AAEL022718 | NA | -0.6083 | 3.6943 | 0.6499 | 0.9996 | NA |
| AAEL012964 | | -0.4342 | 2.2627 | 0.6499 | 0.9996 | |
| AAEL008069 | | -0.2601 | 3.5592 | 0.6502 | 0.9996 | notch [Source:VB Community Annotation] |
| AAEL021676 | NA | 0.1885 | 2.6268 | 0.6502 | 0.9996 | NA |
| AAEL020197 | NA | -0.2365 | 2.8495 | 0.6502 | 0.9996 | NA |
| AAEL010731 | | 0.1267 | 4.1246 | 0.6503 | 0.9996 | |
| AAEL000787 | calypso | 0.2626 | 1.6396 | 0.6506 | 0.9996 | Ubiquitin carboxyl-terminal hydrolase calypso (EC 3.4.19.12) [Source:VB Community Annotation] |
| AAEL008893 | | 0.1908 | 4.8387 | 0.6506 | 0.9996 | xaa-pro dipeptidase pepd/pepq(e.coli) [Source:VB Community Annotation] |
| AAEL000216 | | 0.3449 | 0.3562 | 0.6507 | 0.9996 | elongase, putative [Source:VB Community Annotation] |
| AAEL011086 | | -0.2983 | 2.7337 | 0.6508 | 0.9996 | |
| AAEL003650 | | -0.2727 | 1.6794 | 0.6508 | 0.9996 | inhibitor of growth protein, ing1 [Source:VB Community Annotation] |
| AAEL009112 | | -0.2191 | 4.5390 | 0.6508 | 0.9996 | |
| AAEL010362 | | 0.1525 | 2.9752 | 0.6508 | 0.9996 | |
| AAEL010955 | | 0.1523 | 5.6153 | 0.6510 | 0.9996 | |
| AAEL000186 | | -0.3327 | 4.4079 | 0.6511 | 0.9996 | |
| AAEL021179 | NA | -0.3753 | 3.1151 | 0.6511 | 0.9996 | NA |
| AAEL028635 | NA | -0.2751 | 3.6138 | 0.6512 | 0.9996 | NA |
| AAEL008723 | | -0.1264 | 7.6321 | 0.6513 | 0.9996 | |
| AAEL003295 | | 0.2563 | 3.4432 | 0.6513 | 0.9996 | |
| AAEL009196 | | -0.2509 | 1.7523 | 0.6514 | 0.9996 | calpain [Source:VB Community Annotation] |
| AAEL006009 | | 0.1690 | 3.0599 | 0.6514 | 0.9996 | |
| AAEL008253 | | -0.2672 | 3.3898 | 0.6515 | 0.9996 | |
| AAEL004151 | | -0.1198 | 11.2415 | 0.6515 | 0.9996 | 60S ribosomal protein L29 [Source:UniProtKB/TrEMBL;Acc:Q0IFR8] |
| AAEL013495 | | -0.2231 | 6.2894 | 0.6516 | 0.9996 | |
| AAEL020757 | NA | 0.1314 | 6.0338 | 0.6517 | 0.9996 | NA |

| | | | | | | |
|---|---|---|---|---|---|---|
| AAEL015298 | | 0.1465 | 4.8397 | 0.6518 | 0.9996 | |
| AAEL019539 | NA | -0.8483 | 2.0436 | 0.6520 | 0.9996 | NA |
| AAEL000934 | | -0.1087 | 6.2148 | 0.6521 | 0.9996 | clathrin light chain [Source:VB Community Annotation] |
| AAEL019481 | NA | -0.4531 | 8.7134 | 0.6521 | 0.9996 | NA |
| AAEL008192 | RpS3 | 0.1075 | 10.7644 | 0.6523 | 0.9996 | 40S ribosomal protein S3 [Source:VB Community Annotation] |
| AAEL010433 | gro | -0.5776 | 3.1681 | 0.6524 | 0.9996 | groucho protein [Source:VB Community Annotation] |
| AAEL001359 | | 0.1877 | 2.6323 | 0.6526 | 0.9996 | |
| AAEL009042 | | 0.1567 | 6.4927 | 0.6526 | 0.9996 | ubiquitin fusion degradaton protein [Source:VB Community Annotation] |
| AAEL009165 | | 0.5390 | -2.5234 | 0.6527 | 0.9996 | |
| AAEL012925 | | 0.1452 | 5.1426 | 0.6528 | 0.9996 | carbon catabolite repressor protein [Source:VB Community Annotation] |
| AAEL008486 | | 0.1268 | 4.2058 | 0.6528 | 0.9996 | protein kinase C inhibitor, putative [Source:VB Community Annotation] |
| AAEL007546 | | 0.1482 | 7.6412 | 0.6530 | 0.9996 | actin-related protein 2/3 complex subunit 1A [Source:VB Community Annotation] |
| AAEL011814 | mRpS30 | 0.1242 | 5.3124 | 0.6530 | 0.9996 | mitochondrial ribosomal protein, S30, putative [Source:VB Community Annotation] |
| AAEL013163 | | 0.1832 | 3.9625 | 0.6531 | 0.9996 | |
| AAEL005139 | | 0.1754 | 4.3561 | 0.6532 | 0.9996 | |
| AAEL009105 | APG9 | 0.1790 | 4.8592 | 0.6532 | 0.9996 | autophagy related gene [Source:VB Community Annotation] |
| AAEL021750 | NA | -0.8590 | 1.5342 | 0.6533 | 0.9996 | NA |
| AAEL019690 | NA | -0.4790 | 5.1469 | 0.6534 | 0.9996 | NA |
| AAEL006006 | | 0.1496 | 7.4277 | 0.6534 | 0.9996 | swiprosin [Source:VB Community Annotation] |
| AAEL009383 | | 0.2076 | 2.7215 | 0.6534 | 0.9996 | crooked neck protein [Source:VB Community Annotation] |
| AAEL024480 | NA | -0.1763 | 2.0746 | 0.6536 | 0.9996 | NA |
| AAEL006189 | | 0.0940 | 5.5091 | 0.6537 | 0.9996 | |
| AAEL008179 | Rheb | -0.1306 | 4.8349 | 0.6539 | 0.9996 | GTP-binding protein Rheb [Source:VB Community Annotation] |
| AAEL008066 | | -0.3958 | 3.3320 | 0.6540 | 0.9996 | trkB protein, putative [Source:VB Community Annotation] |
| AAEL001104 | | 0.1089 | 4.5778 | 0.6541 | 0.9996 | |
| AAEL004036 | | 0.5833 | -0.8341 | 0.6541 | 0.9996 | glucose dehydrogenase [Source:VB Community Annotation] |
| AAEL013004 | | 0.2230 | 4.6785 | 0.6542 | 0.9996 | |
| AAEL013633 | | 0.2456 | 2.9206 | 0.6543 | 0.9996 | ubiquitin-conjugating enzyme h [Source:VB Community Annotation] |
| AAEL008136 | | 0.2497 | 2.6673 | 0.6544 | 0.9996 | |
| AAEL019891 | NA | -0.2153 | 1.7437 | 0.6545 | 0.9996 | NA |
| AAEL013408 | | 0.8284 | -0.9255 | 0.6545 | 0.9996 | suppressin [Source:VB Community Annotation] |
| AAEL005787 | | -0.1983 | 4.0253 | 0.6546 | 0.9996 | serine protease, putative [Source:VB Community Annotation] |
| AAEL021333 | NA | -0.4945 | 3.2460 | 0.6547 | 0.9996 | NA |
| AAEL026038 | NA | -0.5839 | -1.1397 | 0.6548 | 0.9996 | NA |
| AAEL017367 | | 0.5846 | 0.1373 | 0.6548 | 0.9996 | |
| AAEL000053 | | -0.5378 | 1.1468 | 0.6548 | 0.9996 | myotubularin [Source:VB Community Annotation] |
| AAEL013271 | | 0.1631 | 6.4526 | 0.6548 | 0.9996 | organic cation transporter [Source:VB Community Annotation] |
| AAEL007371 | | -0.3277 | 6.5593 | 0.6549 | 0.9996 | |
| AAEL003956 | | 0.4393 | 0.3197 | 0.6550 | 0.9996 | amino acid transporter [Source:VB Community Annotation] |
| AAEL014871 | | 0.3812 | 5.5780 | 0.6550 | 0.9996 | methylenetetrahydrofolate dehydrogenase [Source:VB Community Annotation] |

| | | | | | | |
|---|---|---|---|---|---|---|
| AAEL012911 | LRIM18 | 0.1455 | 6.7916 | 0.6550 | 0.9996 | leucine-rich immune protein (Coil-less) [Source:VB Community Annotation] |
| AAEL009922 | | -0.1247 | 4.1707 | 0.6550 | 0.9996 | |
| AAEL008620 | | -0.2444 | 6.7206 | 0.6552 | 0.9996 | D7 protein, putative [Source:VB Community Annotation] |
| AAEL004744 | | -0.1408 | 5.1314 | 0.6553 | 0.9996 | ccaat-binding transcription factor subunit a [Source:VB Community Annotation] |
| AAEL004371 | | 0.1653 | 4.6090 | 0.6554 | 0.9996 | SWI/SNF related matrix associated actin dependent regulator of chromatin subfamily B member 1 [Source:VB Community Annotation] |
| AAEL002595 | | -0.2874 | 8.2110 | 0.6554 | 0.9996 | serine protease [Source:VB Community Annotation] |
| AAEL013712 | | 0.8791 | -0.7809 | 0.6555 | 0.9996 | trypsin 5G1 Precursor (EC 3.4.21.4) [Source:VB Community Annotation] |
| AAEL025097 | NA | -0.6931 | 3.1374 | 0.6555 | 0.9996 | NA |
| AAEL003146 | | 0.1942 | 3.8804 | 0.6555 | 0.9996 | |
| AAEL002051 | | 0.1993 | 1.7712 | 0.6556 | 0.9996 | sodium/hydrogen exchanger 7, 9 (nhe7, nhe9) [Source:VB Community Annotation] |
| AAEL018678 | ND5 | 0.6373 | -0.0356 | 0.6558 | 0.9996 | NADH dehydrogenase subunit 5 [Source:European Nucleotide Archive;Acc:ND5] |
| AAEL026430 | NA | -0.3321 | 3.1929 | 0.6559 | 0.9996 | NA |
| AAEL004875 | | 0.4644 | 3.4300 | 0.6560 | 0.9996 | F-box only protein [Source:VB Community Annotation] |
| AAEL010966 | | -0.1499 | 6.0437 | 0.6561 | 0.9996 | ubiquitin carboxyl-terminal hydrolase isozyme L5 [Source:VB Community Annotation] |
| AAEL025591 | NA | 0.1299 | 5.4994 | 0.6563 | 0.9996 | NA |
| AAEL020031 | NA | 0.3475 | 0.4716 | 0.6563 | 0.9996 | NA |
| AAEL007061 | | 0.1295 | 5.6375 | 0.6563 | 0.9996 | cyclophilin [Source:VB Community Annotation] |
| AAEL024268 | NA | 0.3509 | 3.0509 | 0.6565 | 0.9996 | NA |
| AAEL004329 | | 0.1313 | 5.3734 | 0.6566 | 0.9996 | rab gdp-dissociation inhibitor [Source:VB Community Annotation] |
| AAEL011739 | | -0.1435 | 7.0375 | 0.6567 | 0.9996 | MAFF protein, putative [Source:VB Community Annotation] |
| AAEL008716 | | -0.2291 | 2.7946 | 0.6568 | 0.9996 | |
| AAEL023028 | NA | 0.1301 | 4.8233 | 0.6568 | 0.9996 | NA |
| AAEL009882 | | 0.1239 | 4.5096 | 0.6568 | 0.9996 | retinoblastoma-binding protein 4 (rbbp4) [Source:VB Community Annotation] |
| AAEL008190 | | -0.2497 | 2.8638 | 0.6569 | 0.9996 | bhlh factor math6 (atoh8 protein) (bhlh transcription factor) [Source:VB Community Annotation] |
| AAEL011570 | | 0.1682 | 3.4670 | 0.6570 | 0.9996 | acidic fibroblast growth factor intracellular binding protein [Source:VB Community Annotation] |
| AAEL019451 | NA | -0.2348 | 5.6770 | 0.6571 | 0.9996 | NA |
| AAEL025647 | NA | -0.2302 | 4.6314 | 0.6571 | 0.9996 | NA |
| AAEL011805 | | 0.2972 | 1.4987 | 0.6571 | 0.9996 | zinc finger protein [Source:VB Community Annotation] |
| AAEL006948 | | -0.2699 | 6.5034 | 0.6571 | 0.9996 | tomosyn [Source:VB Community Annotation] |
| AAEL015653 | | 0.1658 | 6.6254 | 0.6572 | 0.9996 | 1-hydroxy-2-glutathionyl-2-methyl-3-butene dehydrogenase, putative [Source:VB Community Annotation] |
| AAEL000138 | | -0.1657 | 8.8510 | 0.6572 | 0.9996 | NADH dehydrogenase, putative [Source:VB Community Annotation] |
| AAEL001918 | | -0.3455 | 5.4487 | 0.6573 | 0.9996 | |
| AAEL006347 | APY | -0.7783 | 8.5636 | 0.6573 | 0.9996 | Apyrase Precursor (EC 3.6.1.5)(Adenosine diphosphatase)(ATP-diphosphohydrolase)(ATP-diphosphatase)(ADPase)(Allergen Aed a 1) [Sou |
| AAEL003918 | | 0.6263 | 1.9924 | 0.6575 | 0.9996 | calcium-dependent protein kinase [Source:VB Community Annotation] |
| AAEL003675 | | 0.1346 | 10.0533 | 0.6575 | 0.9996 | ubiquinol-cytochrome c reductase iron-sulfur subunit [Source:VB Community Annotation] |
| AAEL014428 | | -0.1422 | 3.0513 | 0.6576 | 0.9996 | ABC transporter [Source:VB Community Annotation] |
| AAEL021667 | NA | 0.5708 | -0.4230 | 0.6576 | 0.9996 | NA |
| AAEL006823 | | -0.3154 | 3.1668 | 0.6577 | 0.9996 | AMP dependent ligase [Source:VB Community Annotation] |
| AAEL002932 | | -0.6773 | -1.5320 | 0.6577 | 0.9996 | |
| AAEL000099 | CLIPB33 | -0.3738 | 6.6319 | 0.6577 | 0.9996 | Clip-Domain Serine Protease family B. [Source:VB Community Annotation] |

| | | | | | | |
|---|---|---|---|---|---|---|
| AAEL021138 | NA | 0.1319 | 3.0812 | 0.6578 | 0.9996 | NA |
| AAEL011643 | | -0.6803 | 5.6163 | 0.6579 | 0.9996 | homothorax homeobox protein [Source:VB Community Annotation] |
| AAEL013303 | | -0.3090 | 4.3116 | 0.6579 | 0.9996 | |
| AAEL012234 | | -0.1701 | 5.4597 | 0.6580 | 0.9996 | cytoplasmic dynein intermediate chain, (dhic) [Source:VB Community Annotation] |
| AAEL009476 | | -0.3958 | 4.1321 | 0.6580 | 0.9996 | |
| AAEL026555 | NA | -0.9138 | 2.6636 | 0.6582 | 0.9996 | NA |
| AAEL004999 | | 0.2755 | 4.8395 | 0.6582 | 0.9996 | |
| AAEL012330 | | -0.4455 | 2.2399 | 0.6584 | 0.9996 | tata-box binding protein [Source:VB Community Annotation] |
| AAEL027319 | NA | -0.2724 | 0.2815 | 0.6585 | 0.9996 | NA |
| AAEL007185 | | 0.1530 | 5.8226 | 0.6586 | 0.9996 | |
| AAEL005732 | | 0.3508 | 4.6467 | 0.6587 | 0.9996 | acyl-coa dehydrogenase [Source:VB Community Annotation] |
| AAEL007744 | | -0.1918 | 2.6450 | 0.6587 | 0.9996 | |
| AAEL003018 | | 0.1844 | 4.4207 | 0.6588 | 0.9996 | |
| AAEL001952 | | -0.7609 | 0.1571 | 0.6588 | 0.9996 | 28 kDa heat- and acid-stable phosphoprotein (PDGF-associated protein), putative [Source:VB Community Annotation] |
| AAEL002652 | | -0.6658 | -1.8929 | 0.6589 | 0.9996 | |
| AAEL004833 | DPT1 | -0.2644 | 4.1207 | 0.6590 | 0.9996 | diptericin  anti-microbial peptide [Source:VB Community Annotation] |
| AAEL018347 | CLIPE12 | 0.1332 | 4.3563 | 0.6591 | 0.9996 | Clip-domain serine protease, family E [Source:VB Community Annotation] |
| AAEL005072 | | -0.1675 | 6.9845 | 0.6591 | 0.9996 | MRAS2, putative [Source:VB Community Annotation] |
| AAEL014908 | | 0.2303 | 4.2076 | 0.6592 | 0.9996 | |
| AAEL009887 | | -0.1224 | 4.9575 | 0.6594 | 0.9996 | WD-repeat protein [Source:VB Community Annotation] |
| AAEL005829 | | -0.3937 | 4.1886 | 0.6596 | 0.9996 | |
| AAEL004884 | | 0.2356 | 4.3550 | 0.6597 | 0.9996 | hemomucin [Source:VB Community Annotation] |
| AAEL019588 | NA | -0.3136 | 5.2489 | 0.6597 | 0.9996 | NA |
| AAEL007682 | | -0.4588 | 5.4936 | 0.6600 | 0.9996 | |
| AAEL000568 | | 0.1068 | 5.7410 | 0.6600 | 0.9996 | 26S proteasome non-ATPase regulatory subunit [Source:VB Community Annotation] |
| AAEL011741 | GSTS1 | -0.1461 | 6.6696 | 0.6601 | 0.9996 | glutathione transferase [Source:VB Community Annotation] |
| AAEL005174 | | -0.1865 | 6.6931 | 0.6601 | 0.9996 | |
| AAEL014896 | | -0.5653 | 0.8987 | 0.6601 | 0.9996 | |
| AAEL027509 | NA | -0.2624 | 0.7406 | 0.6604 | 0.9996 | NA |
| AAEL003782 | | 0.1758 | 3.8063 | 0.6604 | 0.9996 | |
| AAEL007228 | APG4B | 0.6857 | -0.6231 | 0.6605 | 0.9996 | autophagy related gene [Source:VB Community Annotation] |
| AAEL006372 | | 0.2549 | 5.7537 | 0.6605 | 0.9996 | sulphate transporter [Source:VB Community Annotation] |
| AAEL007097 | | -0.6994 | 0.7142 | 0.6605 | 0.9996 | 4-nitrophenylphosphatase [Source:VB Community Annotation] |
| AAEL013169 | | 0.3192 | -0.0933 | 0.6606 | 0.9996 | |
| AAEL002092 | | 0.6893 | 3.3312 | 0.6607 | 0.9996 | cuticle protein, putative [Source:VB Community Annotation] |
| AAEL014408 | | -0.2761 | 2.3340 | 0.6608 | 0.9996 | m-phase inducer phosphatase(cdc25) [Source:VB Community Annotation] |
| AAEL001438 | | 0.2219 | 3.0295 | 0.6608 | 0.9996 | |
| AAEL005712 | | -0.4863 | 1.6838 | 0.6608 | 0.9996 | microtubule associated protein xmap215 [Source:VB Community Annotation] |
| AAEL013744 | | 0.1943 | 9.2711 | 0.6608 | 0.9996 | NADH:ubiquinone dehydrogenase, putative [Source:VB Community Annotation] |
| AAEL021092 | NA | -0.5755 | 3.0977 | 0.6608 | 0.9996 | NA |

| | | | | | | |
|---|---|---|---|---|---|---|
| AAEL021006 | NA | -0.3913 | 5.7388 | 0.6609 | 0.9996 | NA |
| AAEL023680 | NA | 0.1582 | 6.2666 | 0.6610 | 0.9996 | NA |
| AAEL019664 | NA | 0.1483 | 2.9861 | 0.6612 | 0.9996 | NA |
| AAEL026652 | NA | -0.1394 | 6.0120 | 0.6615 | 0.9996 | NA |
| AAEL025908 | NA | -0.8903 | 0.7561 | 0.6615 | 0.9996 | NA |
| AAEL012008 | | 0.1527 | 3.3920 | 0.6616 | 0.9996 | cyclophilin [Source:VB Community Annotation] |
| AAEL012639 | | 0.9600 | 0.7907 | 0.6617 | 0.9996 | |
| AAEL011444 | | 0.8941 | -1.0668 | 0.6618 | 0.9996 | pupal cuticle protein, putative [Source:VB Community Annotation] |
| AAEL010981 | | 0.4381 | 2.5524 | 0.6618 | 0.9996 | |
| AAEL020092 | NA | -0.2767 | 10.1174 | 0.6620 | 0.9996 | NA |
| AAEL000829 | | -0.2161 | 4.3674 | 0.6621 | 0.9996 | dimethylaniline monooxygenase [Source:VB Community Annotation] |
| AAEL012284 | | 0.1931 | 4.5000 | 0.6621 | 0.9996 | DNA-directed RNA polymerase I 16 kDa polypeptide [Source:VB Community Annotation] |
| AAEL010181 | mRpL51 | -0.1514 | 5.6421 | 0.6623 | 0.9996 | mitochondrial ribosomal protein, L51, putative [Source:VB Community Annotation] |
| AAEL014717 | | -0.1118 | 6.9241 | 0.6623 | 0.9996 | adiponectin receptor [Source:VB Community Annotation] |
| AAEL012161 | | -0.1115 | 6.3637 | 0.6623 | 0.9996 | |
| AAEL005799 | | 0.1792 | 5.8366 | 0.6627 | 0.9996 | peptidyl-prolyl cis-trans isomerase g, ppig [Source:VB Community Annotation] |
| AAEL004568 | | 0.1909 | 1.7574 | 0.6628 | 0.9996 | ADP-ribosylation factor, arf [Source:VB Community Annotation] |
| AAEL013002 | | -0.1524 | 5.2120 | 0.6629 | 0.9996 | cdk9 [Source:VB Community Annotation] |
| AAEL001252 | | 0.2046 | 4.2015 | 0.6629 | 0.9996 | rap1 GTPase-gdp dissociation stimulator [Source:VB Community Annotation] |
| AAEL025686 | NA | 0.2720 | 6.4762 | 0.6630 | 0.9996 | NA |
| AAEL009501 | | -0.1338 | 4.3579 | 0.6630 | 0.9996 | methylglutaconyl-CoA hydratase, putative [Source:VB Community Annotation] |
| AAEL012704 | SCP-2L3 | -0.1301 | 7.2490 | 0.6633 | 0.9996 | |
| AAEL012566 | | 0.2388 | 1.3315 | 0.6635 | 0.9996 | |
| AAEL024549 | NA | -0.2557 | 10.4835 | 0.6635 | 0.9996 | NA |
| AAEL011012 | | -0.1724 | 6.2917 | 0.6636 | 0.9996 | septin [Source:VB Community Annotation] |
| AAEL018314 | | -0.2517 | 6.0531 | 0.6636 | 0.9996 | |
| AAEL013703 | | -0.3303 | 5.5429 | 0.6637 | 0.9996 | trypsin [Source:VB Community Annotation] |
| AAEL026574 | NA | -0.3126 | 0.7539 | 0.6638 | 0.9996 | NA |
| AAEL006801 | | 0.6542 | -1.1949 | 0.6638 | 0.9996 | Vang-like protein [Source:UniProtKB/TrEMBL;Acc:Q174T9] |
| AAEL007121 | | -0.5711 | 0.1115 | 0.6639 | 0.9996 | tartan [Source:VB Community Annotation] |
| AAEL019997 | NA | -0.5806 | 5.1590 | 0.6640 | 0.9996 | NA |
| AAEL019895 | NA | 0.1379 | 7.3369 | 0.6640 | 0.9996 | NA |
| AAEL019882 | NA | 0.1466 | 5.6268 | 0.6641 | 0.9996 | NA |
| AAEL021502 | NA | -0.5058 | -0.3810 | 0.6642 | 0.9996 | NA |
| AAEL009735 | | -0.2694 | 0.7955 | 0.6643 | 0.9996 | iodotyrosine dehalogenase [Source:VB Community Annotation] |
| AAEL013040 | | -0.6696 | -1.8137 | 0.6643 | 0.9996 | |
| AAEL008787 | VhaA | -0.1030 | 7.3508 | 0.6644 | 0.9996 | V-type proton ATPase catalytic subunit A (V-ATPase subunit A)(EC 3.6.3.14)(Vacuolar proton pump subunit alpha)(V-ATPase 69 kDa subu |
| AAEL003326 | | -0.2421 | 5.3772 | 0.6645 | 0.9996 | |
| AAEL006343 | | -0.1701 | 5.0698 | 0.6645 | 0.9996 | |
| AAEL004146 | CRY1 | -0.2440 | 7.4226 | 0.6645 | 0.9996 | cryptochrome 1 [Source:VB Community Annotation] |

| | | | | | | |
|---|---|---|---|---|---|---|
| AAEL012675 | | -0.1533 | 3.1901 | 0.6646 | 0.9996 | |
| AAEL009731 | | 0.2754 | 4.1077 | 0.6647 | 0.9996 | epsilon-trimethyllysine 2-oxoglutarate dioxygenase [Source:VB Community Annotation] |
| AAEL012865 | | -0.1650 | 5.8009 | 0.6648 | 0.9996 | |
| AAEL012526 | | 0.2226 | 2.7852 | 0.6648 | 0.9996 | |
| AAEL012690 | | -0.1740 | 4.0173 | 0.6648 | 0.9996 | 3-5 exonuclease [Source:VB Community Annotation] |
| AAEL005971 | | -0.6300 | 1.4585 | 0.6649 | 0.9996 | |
| AAEL023407 | NA | 0.2110 | 3.3378 | 0.6650 | 0.9996 | NA |
| AAEL010398 | | -0.2663 | 3.5934 | 0.6651 | 0.9996 | |
| AAEL005199 | | -0.2857 | 3.2820 | 0.6651 | 0.9996 | |
| AAEL012324 | | 0.1441 | 4.3551 | 0.6652 | 0.9996 | protease m50 membrane-bound transcription factor site 2 protease [Source:VB Community Annotation] |
| AAEL027150 | NA | -0.3270 | 5.5560 | 0.6653 | 0.9996 | NA |
| AAEL006452 | | 0.1259 | 4.1974 | 0.6654 | 0.9996 | n-acetylgalactosaminyltransferase [Source:VB Community Annotation] |
| AAEL002433 | | -0.1477 | 3.0857 | 0.6655 | 0.9996 | |
| AAEL022459 | NA | -0.8180 | -1.0731 | 0.6655 | 0.9996 | NA |
| AAEL025995 | NA | 0.4135 | 6.1602 | 0.6656 | 0.9996 | NA |
| AAEL010070 | | 0.1089 | 5.8096 | 0.6656 | 0.9996 | guanine nucleotide-binding protein beta 5 (g protein beta5) [Source:VB Community Annotation] |
| AAEL019955 | NA | 0.6155 | -0.8864 | 0.6656 | 0.9996 | NA |
| AAEL018168 | | 0.1890 | 2.9308 | 0.6656 | 0.9996 | |
| AAEL022229 | NA | -0.1550 | 4.1646 | 0.6658 | 0.9996 | NA |
| AAEL001242 | | -0.9467 | 2.2496 | 0.6658 | 0.9996 | ribosomal pseudouridine synthase [Source:VB Community Annotation] |
| AAEL026516 | NA | 0.1799 | 4.3781 | 0.6662 | 0.9996 | NA |
| AAEL001022 | | -0.3595 | 7.6821 | 0.6662 | 0.9996 | anterior fat body protein [Source:VB Community Annotation] |
| AAEL025353 | NA | 0.6812 | -1.3611 | 0.6663 | 0.9996 | NA |
| AAEL013466 | | -0.2094 | 7.0149 | 0.6663 | 0.9996 | ankyrin 2,3/unc44 [Source:VB Community Annotation] |
| AAEL014615 | CYP9J23 | -0.2251 | 8.4619 | 0.6664 | 0.9996 | cytochrome P450 [Source:VB Community Annotation] |
| AAEL005556 | | -0.2612 | 1.8293 | 0.6666 | 0.9996 | |
| AAEL012640 | | -0.7700 | 3.3965 | 0.6667 | 0.9996 | |
| AAEL023561 | NA | 0.1238 | 3.8770 | 0.6667 | 0.9996 | NA |
| AAEL009359 | | -0.2680 | 3.9261 | 0.6668 | 0.9996 | |
| AAEL003728 | | -0.4587 | 1.4343 | 0.6668 | 0.9996 | |
| AAEL013608 | | -0.1364 | 6.5411 | 0.6669 | 0.9996 | sugar transporter [Source:VB Community Annotation] |
| AAEL009611 | | 0.3135 | 1.1507 | 0.6669 | 0.9996 | |
| AAEL008157 | | 0.4479 | 0.2138 | 0.6669 | 0.9996 | |
| AAEL019952 | NA | -0.3407 | 4.1317 | 0.6669 | 0.9996 | NA |
| AAEL010943 | | -0.1886 | 7.6975 | 0.6669 | 0.9996 | |
| AAEL019791 | NA | 0.0970 | 5.3737 | 0.6670 | 0.9996 | NA |
| AAEL023046 | NA | -0.5644 | 2.5846 | 0.6671 | 0.9996 | NA |
| AAEL003972 | | -0.1871 | 3.7843 | 0.6672 | 0.9996 | |
| AAEL025418 | NA | 0.3230 | 4.1067 | 0.6672 | 0.9996 | NA |
| AAEL000408 | | 0.1813 | 4.4923 | 0.6673 | 0.9996 | |

| | | | | | | |
|---|---|---|---|---|---|---|
| AAEL000810 | | -0.7920 | -0.1809 | 0.6673 | 0.9996 | Protein kinase C [Source:UniProtKB/TrEMBL;Acc:Q17N56] |
| AAEL004039 | | 0.1815 | 4.1667 | 0.6675 | 0.9996 | blooms syndrome DNA helicase [Source:VB Community Annotation] |
| AAEL014715 | | 0.2180 | 5.4449 | 0.6677 | 0.9996 | Eukaryotic translation initiation factor 3 subunit L (eIF3l) [Source:VB Community Annotation] |
| AAEL014549 | | -0.1077 | 5.4570 | 0.6677 | 0.9996 | |
| AAEL013385 | | 0.4839 | -0.2965 | 0.6677 | 0.9996 | brain chitinase and chia [Source:VB Community Annotation] |
| AAEL014617 | CYP9J28 | -0.3799 | 2.3863 | 0.6678 | 0.9996 | cytochrome P450 [Source:VB Community Annotation] |
| AAEL010197 | | -0.3403 | 0.2164 | 0.6679 | 0.9996 | axonemal dynein intermediate chain [Source:VB Community Annotation] |
| AAEL009720 | | 0.1578 | 3.1860 | 0.6679 | 0.9996 | |
| AAEL004165 | | 0.1213 | 5.4031 | 0.6681 | 0.9996 | bone morphogenetic protein receptor type II, putative [Source:VB Community Annotation] |
| AAEL010423 | | 0.1529 | 5.3846 | 0.6681 | 0.9996 | ARL3, putative [Source:VB Community Annotation] |
| AAEL011763 | PPO3 | 0.5563 | 5.4141 | 0.6681 | 0.9996 | prophenoloxidase [Source:VB Community Annotation] |
| AAEL002214 | | 0.1909 | 4.7548 | 0.6682 | 0.9996 | amino acid transporter [Source:VB Community Annotation] |
| AAEL021394 | NA | -0.2706 | 4.6527 | 0.6682 | 0.9996 | NA |
| AAEL013058 | | 0.1301 | 4.0275 | 0.6683 | 0.9996 | mitochondrial peptide chain release factor [Source:VB Community Annotation] |
| AAEL019974 | NA | -0.3077 | 5.8941 | 0.6684 | 0.9996 | NA |
| AAEL019692 | NA | -0.6309 | 4.5288 | 0.6684 | 0.9996 | NA |
| AAEL014695 | | -0.3318 | 3.4199 | 0.6685 | 0.9996 | zinc finger protein [Source:VB Community Annotation] |
| AAEL021262 | NA | 0.1845 | 4.4006 | 0.6685 | 0.9996 | NA |
| AAEL001623 | REG | 0.1129 | 6.6252 | 0.6686 | 0.9996 | proteasome activator subunit REG [Source:VB Community Annotation] |
| AAEL002791 | | -0.4525 | -1.1265 | 0.6686 | 0.9996 | |
| AAEL004389 | | -0.3469 | 4.8879 | 0.6687 | 0.9996 | mannosidase alpha class 2a [Source:VB Community Annotation] |
| AAEL020347 | NA | -0.2247 | 2.8883 | 0.6687 | 0.9996 | NA |
| AAEL003213 | | -0.1657 | 3.4878 | 0.6688 | 0.9996 | |
| AAEL020001 | NA | -0.2164 | 3.9618 | 0.6688 | 0.9996 | NA |
| AAEL014439 | | 0.1695 | 2.7154 | 0.6689 | 0.9996 | juvenile hormone-inducible protein, putative [Source:VB Community Annotation] |
| AAEL002288 | | -0.3018 | 5.9847 | 0.6689 | 0.9996 | serine protease [Source:VB Community Annotation] |
| AAEL012650 | | -0.6917 | -1.2503 | 0.6689 | 0.9996 | |
| AAEL008832 | | -0.1318 | 5.3815 | 0.6689 | 0.9996 | forkhead box protein (AaegFOXN1) [Source:VB Community Annotation] |
| AAEL000807 | | 0.1626 | 3.9243 | 0.6690 | 0.9996 | tetratricopeptide repeat protein, putative [Source:VB Community Annotation] |
| AAEL019766 | NA | -0.8517 | 0.7126 | 0.6690 | 0.9996 | NA |
| AAEL003890 | | 0.2577 | 3.9036 | 0.6690 | 0.9996 | cytochrome P450 [Source:VB Community Annotation] |
| AAEL005798 | | -0.1815 | 4.3344 | 0.6690 | 0.9996 | ATP synthase subunit beta vacuolar [Source:VB Community Annotation] |
| AAEL018118 | | -0.1752 | 2.7575 | 0.6692 | 0.9996 | |
| AAEL013375 | | 0.4596 | 0.2648 | 0.6693 | 0.9996 | |
| AAEL008829 | | -0.6844 | -1.6231 | 0.6694 | 0.9996 | |
| AAEL006011 | | 0.3643 | 5.8164 | 0.6695 | 0.9996 | |
| AAEL005558 | | 0.2623 | 6.0429 | 0.6697 | 0.9996 | |
| AAEL021473 | NA | -0.1218 | 4.8193 | 0.6698 | 0.9996 | NA |
| AAEL002464 | | 0.1196 | 7.4585 | 0.6699 | 0.9996 | vacuolar ATP synthase subunit f [Source:VB Community Annotation] |
| AAEL011541 | | 0.1244 | 3.3831 | 0.6700 | 0.9996 | ubiquitin protein ligase [Source:VB Community Annotation] |

| | | | | | | |
|---|---|---|---|---|---|---|
| AAEL022666 | NA | 0.1622 | 3.2330 | 0.6702 | 0.9996 | NA |
| AAEL026486 | NA | -0.4062 | -0.3468 | 0.6702 | 0.9996 | NA |
| AAEL008634 | | -0.1778 | 7.4561 | 0.6705 | 0.9996 | jnk [Source:VB Community Annotation] |
| AAEL006457 | | -0.1934 | 3.0494 | 0.6705 | 0.9996 | asparagine synthetase [Source:VB Community Annotation] |
| AAEL028782 | NA | -1.0904 | 0.2057 | 0.6707 | 0.9996 | NA |
| AAEL014566 | | -0.2688 | 4.0425 | 0.6707 | 0.9996 | wingless [Source:VB Community Annotation] |
| AAEL011750 | | -0.7820 | 0.5933 | 0.6707 | 0.9996 | actin [Source:VB Community Annotation] |
| AAEL001789 | | 0.1534 | 3.4833 | 0.6710 | 0.9996 | |
| AAEL006428 | | -0.1275 | 3.9271 | 0.6710 | 0.9996 | ferredoxin, putative [Source:VB Community Annotation] |
| AAEL011163 | | 0.1416 | 2.9530 | 0.6711 | 0.9996 | |
| AAEL007422 | | -0.1787 | 3.1673 | 0.6713 | 0.9996 | cp27, putative [Source:VB Community Annotation] |
| AAEL000618 | | -0.3403 | -0.6225 | 0.6713 | 0.9996 | wingless protein, putative [Source:VB Community Annotation] |
| AAEL009772 | | 0.1058 | 5.7335 | 0.6717 | 0.9996 | |
| AAEL022328 | NA | -0.6407 | 4.9117 | 0.6717 | 0.9996 | NA |
| AAEL018159 | | 0.2889 | 6.6881 | 0.6718 | 0.9996 | |
| AAEL011703 | | -0.1687 | 3.1377 | 0.6719 | 0.9996 | NAD kinase 2, mitochondrial [Source:UniProtKB/TrEMBL;Acc:Q16PA6] |
| AAEL003958 | | -0.5717 | 0.7056 | 0.6721 | 0.9996 | neurotransmitter gated ion channel [Source:VB Community Annotation] |
| AAEL000332 | | 0.1547 | 3.8394 | 0.6723 | 0.9996 | |
| AAEL012774 | | 0.3998 | -2.0355 | 0.6723 | 0.9996 | protease m1 zinc metalloprotease [Source:VB Community Annotation] |
| AAEL005463 | | -0.7801 | -0.4574 | 0.6723 | 0.9996 | calbindin-32 [Source:VB Community Annotation] |
| AAEL018111 | | 0.1964 | 3.0345 | 0.6726 | 0.9996 | |
| AAEL015307 | | -0.1771 | 3.9022 | 0.6726 | 0.9996 | |
| AAEL027771 | NA | 0.2119 | 9.1352 | 0.6729 | 0.9996 | NA |
| AAEL008048 | | -0.1512 | 5.0984 | 0.6729 | 0.9996 | |
| AAEL023227 | NA | 0.2487 | 1.0271 | 0.6729 | 0.9996 | NA |
| AAEL008472 | | -0.1691 | 5.0987 | 0.6730 | 0.9996 | glycerol kinase [Source:VB Community Annotation] |
| AAEL007143 | | -0.5233 | 4.4700 | 0.6731 | 0.9996 | voltage-gated potassium channel [Source:VB Community Annotation] |
| AAEL001030 | | -0.1169 | 4.7710 | 0.6733 | 0.9996 | S-formylglutathione hydrolase, putative [Source:VB Community Annotation] |
| AAEL011719 | | 0.1825 | 4.1758 | 0.6733 | 0.9996 | |
| AAEL008617 | | -0.5477 | 4.3823 | 0.6733 | 0.9996 | |
| AAEL006602 | | -0.2411 | 7.0050 | 0.6734 | 0.9996 | |
| AAEL019629 | NA | 0.4098 | 0.2490 | 0.6734 | 0.9996 | NA |
| AAEL024191 | NA | -0.1501 | 6.0759 | 0.6736 | 0.9996 | NA |
| AAEL022406 | NA | -0.1482 | 4.8624 | 0.6736 | 0.9996 | NA |
| AAEL004846 | | -0.4247 | -0.8048 | 0.6736 | 0.9996 | |
| AAEL000416 | | -0.3526 | 0.1962 | 0.6736 | 0.9996 | |
| AAEL003049 | | -0.6314 | 3.9634 | 0.6737 | 0.9996 | pupal cuticle protein 78E, putative [Source:VB Community Annotation] |
| AAEL011781 | | 0.1680 | 5.9742 | 0.6739 | 0.9996 | perixosomal biogenesis factor [Source:VB Community Annotation] |
| AAEL022945 | NA | 0.1731 | 2.3881 | 0.6741 | 0.9996 | NA |
| AAEL028202 | NA | 0.4988 | 0.0695 | 0.6742 | 0.9996 | NA |

| | | | | | | |
|---|---|---|---|---|---|---|
| AAEL026468 | NA | -0.1800 | 2.6979 | 0.6742 | 0.9996 | NA |
| AAEL008419 | | 0.1692 | 1.9672 | 0.6746 | 0.9996 | |
| AAEL017986 | | -0.7286 | 0.4134 | 0.6747 | 0.9996 | |
| AAEL004451 | | 0.1774 | 4.0235 | 0.6747 | 0.9996 | organic cation transporter [Source:VB Community Annotation] |
| AAEL001919 | | -0.1261 | 4.9676 | 0.6750 | 0.9996 | protein tyrosine phosphatase, non-receptor type nt1 [Source:VB Community Annotation] |
| AAEL008356 | | -0.3426 | 6.5841 | 0.6752 | 0.9996 | |
| AAEL008932 | Kir1 | -0.1514 | 3.1573 | 0.6752 | 0.9996 | inward-rectifying potassium channel [Source:VB Community Annotation] |
| AAEL006135 | Cbp20 | 0.1715 | 4.4092 | 0.6754 | 0.9996 | Nuclear cap-binding protein subunit 2 (20 kDa nuclear cap-binding protein)(NCBP 20 kDa subunit)(CBP20) [Source:VB Community Annota |
| AAEL006541 | | 0.3381 | 1.5793 | 0.6755 | 0.9996 | axonemal dynein intermediate chain inner arm i1 [Source:VB Community Annotation] |
| AAEL018125 | | 0.6522 | 0.4447 | 0.6755 | 0.9996 | |
| AAEL018858 | tRNA-Arg | 0.3112 | 0.3981 | 0.6757 | 0.9996 | |
| AAEL013990 | | 0.3322 | 5.1701 | 0.6757 | 0.9996 | hexamerin 2 beta [Source:VB Community Annotation] |
| AAEL018141 | | 0.2471 | 1.8006 | 0.6758 | 0.9996 | |
| AAEL025111 | NA | 0.6796 | -1.6007 | 0.6760 | 0.9996 | NA |
| AAEL006609 | | -0.2521 | 5.4197 | 0.6760 | 0.9996 | zinc finger protein [Source:VB Community Annotation] |
| AAEL000722 | | -0.1844 | 4.6933 | 0.6760 | 0.9996 | |
| AAEL027422 | NA | 0.4085 | -1.0976 | 0.6761 | 0.9996 | NA |
| AAEL010749 | | -0.5325 | 3.1548 | 0.6762 | 0.9996 | |
| AAEL012162 | | 0.3915 | 3.6784 | 0.6762 | 0.9996 | Aldehyde dehydrogenase [Source:UniProtKB/TrEMBL;Acc:Q16MV5] |
| AAEL023379 | NA | -0.5419 | -0.7271 | 0.6763 | 0.9996 | NA |
| AAEL000412 | | 0.1326 | 5.3834 | 0.6763 | 0.9996 | |
| AAEL010359 | | 0.1851 | 6.9017 | 0.6763 | 0.9996 | ATP-dependent transporter [Source:VB Community Annotation] |
| AAEL007015 | | 0.2368 | 4.1387 | 0.6764 | 0.9996 | |
| AAEL020903 | NA | 0.1261 | 5.5373 | 0.6765 | 0.9996 | NA |
| AAEL001091 | | -0.1733 | 7.6339 | 0.6767 | 0.9996 | malic enzyme [Source:VB Community Annotation] |
| AAEL005690 | | -0.1111 | 4.4852 | 0.6767 | 0.9996 | cyclophilin-6 [Source:VB Community Annotation] |
| AAEL004025 | | -0.2218 | 5.8376 | 0.6768 | 0.9996 | glucose dehydrogenase [Source:VB Community Annotation] |
| AAEL010906 | | -0.2368 | 2.9630 | 0.6768 | 0.9996 | |
| AAEL023490 | NA | -0.7733 | -0.1093 | 0.6769 | 0.9996 | NA |
| AAEL008722 | | 0.2971 | 2.7549 | 0.6770 | 0.9996 | |
| AAEL022022 | NA | -0.1705 | 2.5282 | 0.6770 | 0.9996 | NA |
| AAEL024714 | NA | 0.1468 | 2.7256 | 0.6771 | 0.9996 | NA |
| AAEL021375 | NA | -0.2409 | 3.0649 | 0.6771 | 0.9996 | NA |
| AAEL010509 | | 0.2124 | 5.1222 | 0.6772 | 0.9996 | bridging integrator [Source:VB Community Annotation] |
| AAEL008138 | | 0.0902 | 6.0243 | 0.6772 | 0.9996 | ABC transporter [Source:VB Community Annotation] |
| AAEL012114 | | 0.2814 | 6.9146 | 0.6775 | 0.9996 | troponin C [Source:VB Community Annotation] |
| AAEL025179 | NA | -0.2038 | 3.0150 | 0.6776 | 0.9996 | NA |
| AAEL004633 | | -0.1167 | 4.5337 | 0.6776 | 0.9996 | nicastrin, putative [Source:VB Community Annotation] |
| AAEL013902 | | -0.2514 | 0.8853 | 0.6777 | 0.9996 | pcdc2/rp-8 (programmed cell death protein 2) [Source:VB Community Annotation] |
| AAEL019549 | NA | 0.2373 | 5.3361 | 0.6778 | 0.9996 | NA |

| | | | | | | |
|---|---|---|---|---|---|---|
| AAEL024887 | NA | -0.1511 | 6.9815 | 0.6778 | 0.9996 | NA |
| AAEL025502 | NA | -0.2145 | 4.9554 | 0.6779 | 0.9996 | NA |
| AAEL004623 | | -0.6460 | 2.2622 | 0.6779 | 0.9996 | band 4.1-like protein 5, putative [Source:VB Community Annotation] |
| AAEL009588 | | -0.1754 | 4.8449 | 0.6780 | 0.9996 | expressed protein (HR3) [Source:VB Community Annotation] |
| AAEL002490 | | -0.4086 | -0.2455 | 0.6780 | 0.9996 | |
| AAEL013789 | | 0.5272 | -0.9531 | 0.6781 | 0.9996 | |
| AAEL009512 | | -0.1198 | 4.7118 | 0.6782 | 0.9996 | glycosylphosphatidylinositol anchor attachment protein, putative [Source:VB Community Annotation] |
| AAEL011641 | | 0.4427 | 1.5645 | 0.6783 | 0.9996 | transferrin [Source:VB Community Annotation] |
| AAEL017567 | | -0.4464 | -2.5738 | 0.6784 | 0.9996 | |
| AAEL012644 | | 0.9311 | 2.4031 | 0.6784 | 0.9996 | |
| AAEL002754 | | -1.0424 | 1.4916 | 0.6785 | 0.9996 | nephrin [Source:VB Community Annotation] |
| AAEL001744 | | -0.1336 | 4.4393 | 0.6787 | 0.9996 | jnk interacting protein (jip) [Source:VB Community Annotation] |
| AAEL011007 | | -0.3322 | 4.9140 | 0.6787 | 0.9996 | fibrinogen and fibronectin [Source:VB Community Annotation] |
| AAEL027611 | NA | -0.1017 | 5.6423 | 0.6788 | 0.9996 | NA |
| AAEL007986 | | 0.6952 | 12.1570 | 0.6789 | 0.9996 | |
| AAEL000267 | | -0.3653 | 0.0161 | 0.6789 | 0.9996 | |
| AAEL010086 | | 0.3183 | 1.1528 | 0.6790 | 0.9996 | DNA replication licensing factor MCM4 [Source:VB Community Annotation] |
| AAEL021366 | NA | -0.4875 | -1.4958 | 0.6790 | 0.9996 | NA |
| AAEL004383 | | -0.1445 | 4.5710 | 0.6791 | 0.9996 | |
| AAEL013939 | | -0.4702 | 5.6014 | 0.6791 | 0.9996 | erk1/2 [Source:VB Community Annotation] |
| AAEL020178 | NA | 0.4858 | 0.0199 | 0.6791 | 0.9996 | NA |
| AAEL009838 | | -0.1038 | 5.8907 | 0.6791 | 0.9996 | glycogen debranching enzyme [Source:VB Community Annotation] |
| AAEL007945 | | 0.1116 | 8.7870 | 0.6791 | 0.9996 | Eukaryotic translation initiation factor 3 subunit H (eIF3h) [Source:VB Community Annotation] |
| AAEL008860 | | -0.2361 | 2.2257 | 0.6792 | 0.9996 | |
| AAEL019421 | NA | -0.1392 | 3.5082 | 0.6793 | 0.9996 | NA |
| AAEL019924 | NA | 0.6514 | 1.6199 | 0.6793 | 0.9996 | NA |
| AAEL010492 | | 0.4755 | -1.0979 | 0.6794 | 0.9996 | |
| AAEL007296 | | -0.1636 | 5.5106 | 0.6794 | 0.9996 | Fatty acyl-CoA reductase [Source:UniProtKB/TrEMBL;Acc:Q172R4] |
| AAEL003194 | | 0.1563 | 5.3709 | 0.6794 | 0.9996 | xaa-pro aminopeptidase [Source:VB Community Annotation] |
| AAEL009173 | | -0.3148 | 4.2297 | 0.6795 | 0.9996 | fasciclin ii (fas ii) [Source:VB Community Annotation] |
| AAEL001144 | | -0.1188 | 4.8030 | 0.6795 | 0.9996 | |
| AAEL018297 | | -0.1452 | 5.0070 | 0.6795 | 0.9996 | |
| AAEL028652 | NA | -0.2982 | 3.6597 | 0.6795 | 0.9996 | NA |
| AAEL026013 | NA | 0.2919 | 2.2529 | 0.6797 | 0.9996 | NA |
| AAEL012076 | | 0.1617 | 4.5511 | 0.6797 | 0.9996 | |
| AAEL005437 | | -0.3891 | 0.8888 | 0.6798 | 0.9996 | transient receptor potential channel [Source:VB Community Annotation] |
| AAEL010730 | | 0.1725 | 3.4626 | 0.6798 | 0.9996 | |
| AAEL010150 | | -0.1913 | 2.6173 | 0.6799 | 0.9996 | |
| AAEL010500 | GSTX2 | 0.1486 | 7.1982 | 0.6799 | 0.9996 | glutathione transferase [Source:VB Community Annotation] |
| AAEL001474 | | 0.1791 | 2.8771 | 0.6799 | 0.9996 | |

| | | | | | | |
|---|---|---|---|---|---|---|
| AAEL001203 | | -0.1300 | 4.8122 | 0.6800 | 0.9996 | Palmitoyltransferase [Source:UniProtKB/TrEMBL;Acc:A0A1S4EY31] |
| AAEL015293 | | 0.2910 | 1.0664 | 0.6801 | 0.9996 | zinc finger protein [Source:VB Community Annotation] |
| AAEL019485 | NA | -0.3795 | 8.2388 | 0.6801 | 0.9996 | NA |
| AAEL011684 | | -0.1609 | 3.4046 | 0.6803 | 0.9996 | |
| AAEL011049 | | 0.4665 | 0.5022 | 0.6803 | 0.9996 | condensin, XCAP-G'-subunit, putative [Source:VB Community Annotation] |
| AAEL007302 | | -0.2595 | 1.2474 | 0.6803 | 0.9996 | |
| AAEL018136 | | -0.3208 | 2.1352 | 0.6805 | 0.9996 | |
| AAEL005895 | | 0.1525 | 8.7145 | 0.6806 | 0.9996 | |
| AAEL014518 | | -0.4912 | -1.2952 | 0.6807 | 0.9996 | |
| AAEL003801 | mRpS5 | -0.1122 | 5.4750 | 0.6807 | 0.9996 | 28S ribosomal protein S5 [Source:VB Community Annotation] |
| AAEL019773 | NA | 0.7658 | 2.3245 | 0.6807 | 0.9996 | NA |
| AAEL007633 | | 0.1471 | 5.9628 | 0.6810 | 0.9996 | dihydropyrimidinase [Source:VB Community Annotation] |
| AAEL011114 | | -0.1662 | 4.1117 | 0.6810 | 0.9996 | serine/threonine-protein kinase rio2 (rio kinase 2) [Source:VB Community Annotation] |
| AAEL024553 | NA | 0.4075 | 0.4389 | 0.6811 | 0.9996 | NA |
| AAEL007034 | | -0.3503 | 6.6678 | 0.6811 | 0.9996 | |
| AAEL020260 | NA | 0.2462 | 2.7394 | 0.6811 | 0.9996 | NA |
| AAEL006682 | | -0.1761 | 6.2446 | 0.6812 | 0.9996 | |
| AAEL021073 | NA | -0.1409 | 4.6995 | 0.6813 | 0.9996 | NA |
| AAEL019875 | NA | -0.5741 | 3.9668 | 0.6815 | 0.9996 | NA |
| AAEL004388 | HPX8A | 0.5700 | -1.2428 | 0.6816 | 0.9996 | heme peroxidase [Source:VB Community Annotation] |
| AAEL023703 | NA | -0.4344 | -1.1998 | 0.6816 | 0.9996 | NA |
| AAEL002397 | | 0.1509 | 4.9285 | 0.6817 | 0.9996 | |
| AAEL011532 | | 0.4480 | -0.5286 | 0.6817 | 0.9996 | |
| AAEL019944 | NA | 0.1488 | 3.7581 | 0.6817 | 0.9996 | NA |
| AAEL024004 | NA | -0.4322 | -0.7412 | 0.6818 | 0.9996 | NA |
| AAEL019709 | NA | -0.7276 | 0.2329 | 0.6819 | 0.9996 | NA |
| AAEL027879 | NA | 0.0987 | 5.4276 | 0.6819 | 0.9996 | NA |
| AAEL002888 | | 0.1576 | 3.9579 | 0.6819 | 0.9996 | williams-beuren syndrome critical region protein [Source:VB Community Annotation] |
| AAEL023010 | NA | -0.2428 | 2.2947 | 0.6819 | 0.9996 | NA |
| AAEL019866 | NA | -0.6873 | 1.6296 | 0.6820 | 0.9996 | NA |
| AAEL024235 | NA | 0.1089 | 5.1421 | 0.6820 | 0.9996 | NA |
| AAEL002260 | | -0.1211 | 5.1674 | 0.6820 | 0.9996 | |
| AAEL015163 | | -0.4931 | -0.0198 | 0.6821 | 0.9996 | cuticle protein, putative [Source:VB Community Annotation] |
| AAEL005584 | | 0.1587 | 1.6459 | 0.6821 | 0.9996 | |
| AAEL019598 | NA | -0.1845 | 3.0431 | 0.6823 | 0.9996 | NA |
| AAEL019740 | NA | -0.2978 | 5.7101 | 0.6824 | 0.9996 | NA |
| AAEL017136 | CYP325V1 | 0.2504 | 5.9211 | 0.6827 | 0.9996 | cytochrome P450 [Source:VB Community Annotation] |
| AAEL010099 | | 0.2388 | 4.5934 | 0.6827 | 0.9996 | 4-nitrophenylphosphatase [Source:VB Community Annotation] |
| AAEL007306 | | 0.1672 | 7.9092 | 0.6828 | 0.9996 | alpha-actinin [Source:VB Community Annotation] |
| AAEL014489 | | 0.1218 | 4.1309 | 0.6829 | 0.9996 | D-tyrosyl-tRNA(Tyr) deacylase [Source:UniProtKB/TrEMBL;Acc:Q16G85] |

| | | | | | | |
|---|---|---|---|---|---|---|
| AAEL020748 | NA | 0.6008 | -1.7717 | 0.6830 | 0.9996 | NA |
| AAEL013818 | | -0.1316 | 4.3841 | 0.6831 | 0.9996 | spliceosome associated protein [Source:VB Community Annotation] |
| AAEL000845 | | -0.3160 | 1.0707 | 0.6832 | 0.9996 | protein-tyrosine phosphatase [Source:VB Community Annotation] |
| AAEL005379 | | -0.3202 | 3.8135 | 0.6832 | 0.9996 | |
| AAEL012876 | | -0.1953 | 2.5394 | 0.6833 | 0.9996 | |
| AAEL010884 | | -0.2361 | 8.8217 | 0.6833 | 0.9996 | ADP,ATP carrier protein [Source:VB Community Annotation] |
| AAEL019902 | NA | 0.3513 | 3.3750 | 0.6833 | 0.9996 | NA |
| AAEL010120 | | 0.1502 | 5.3186 | 0.6833 | 0.9996 | chondroitin 4-sulfotransferase [Source:VB Community Annotation] |
| AAEL009669 | | -0.4994 | 2.4577 | 0.6833 | 0.9996 | |
| AAEL001047 | | -0.2188 | 6.3639 | 0.6834 | 0.9996 | adenylate cyclase [Source:VB Community Annotation] |
| AAEL025597 | NA | 0.1278 | 8.9172 | 0.6837 | 0.9996 | NA |
| AAEL011282 | | 0.1789 | 3.4546 | 0.6837 | 0.9996 | ribosomal RNA small subunit methyltransferase b (sun) [Source:VB Community Annotation] |
| AAEL007770 | | -0.1285 | 3.9878 | 0.6837 | 0.9996 | voltage and ligand gated potassium channel [Source:VB Community Annotation] |
| AAEL007137 | | -0.4593 | 2.5858 | 0.6838 | 0.9996 | |
| AAEL007135 | | 0.1250 | 7.5563 | 0.6840 | 0.9996 | peroxiredoxin 5, prdx5 [Source:VB Community Annotation] |
| AAEL013970 | | -0.1117 | 4.5502 | 0.6840 | 0.9996 | Defective in cullin neddylation protein [Source:UniProtKB/TrEMBL;Acc:Q16HN1] |
| AAEL021450 | NA | -0.2430 | 1.8571 | 0.6840 | 0.9996 | NA |
| AAEL027549 | NA | -0.5586 | -2.3765 | 0.6841 | 0.9996 | NA |
| AAEL008833 | | -0.1626 | 4.6063 | 0.6841 | 0.9996 | |
| AAEL003550 | | -0.5519 | -0.7474 | 0.6842 | 0.9996 | aquaporin [Source:VB Community Annotation] |
| AAEL021016 | NA | 0.2292 | 2.5012 | 0.6842 | 0.9996 | NA |
| AAEL004148 | | -0.1528 | 6.0177 | 0.6842 | 0.9996 | heat shock protein 70 (hsp70)-interacting protein [Source:VB Community Annotation] |
| AAEL028013 | NA | 0.3568 | 3.7058 | 0.6842 | 0.9996 | NA |
| AAEL023549 | NA | 0.2863 | 2.9843 | 0.6843 | 0.9996 | NA |
| AAEL002186 | | 0.1494 | 2.9931 | 0.6843 | 0.9996 | |
| AAEL003773 | | 0.6372 | 0.2844 | 0.6844 | 0.9996 | |
| AAEL001910 | | -0.1510 | 3.3809 | 0.6844 | 0.9996 | zinc finger protein [Source:VB Community Annotation] |
| AAEL017080 | | 0.5585 | -0.1591 | 0.6844 | 0.9996 | |
| AAEL007826 | for | -0.1344 | 4.0503 | 0.6845 | 0.9996 | cGMP-dependent protein kinase [Source:UniProtKB/TrEMBL;Acc:Q170R4] |
| AAEL013118 | | -0.7186 | -1.3553 | 0.6846 | 0.9996 | |
| AAEL016115 | tRNA-Lys | 0.3725 | 0.7166 | 0.6846 | 0.9996 | |
| AAEL011321 | | 0.2928 | 0.7542 | 0.6847 | 0.9996 | |
| AAEL004919 | | -0.1394 | 6.1092 | 0.6847 | 0.9996 | |
| AAEL008120 | | 0.1605 | 5.2007 | 0.6847 | 0.9996 | ribosomal RNA methyltransferase [Source:VB Community Annotation] |
| AAEL014335 | | -0.1626 | 2.8650 | 0.6847 | 0.9996 | |
| AAEL013103 | | 0.1275 | 4.5708 | 0.6848 | 0.9996 | |
| AAEL007106 | | -0.3048 | 3.1128 | 0.6848 | 0.9996 | serine protease, putative [Source:VB Community Annotation] |
| AAEL002235 | | -0.1846 | 6.1912 | 0.6849 | 0.9996 | |
| AAEL014212 | | 0.0953 | 4.8705 | 0.6850 | 0.9996 | androgen induced inhibitor of proliferation (as3) / pds5 [Source:VB Community Annotation] |
| AAEL006451 | | 0.2485 | 11.3649 | 0.6850 | 0.9996 | |

| | | | | | | |
|---|---|---|---|---|---|---|
| AAEL010389 | | 0.2346 | 2.1527 | 0.6851 | 0.9996 | Carboxylic ester hydrolase (Fragment) [Source:UniProtKB/TrEMBL;Acc:Q16T49] |
| AAEL015110 | | 0.5137 | 1.2648 | 0.6851 | 0.9996 | dipeptidyl-peptidase [Source:VB Community Annotation] |
| AAEL007582 | | -0.2152 | 3.4228 | 0.6851 | 0.9996 | granzyme A precursor, putative [Source:VB Community Annotation] |
| AAEL003294 | | -0.2242 | 3.1605 | 0.6852 | 0.9996 | fibrinogen and fibronectin [Source:VB Community Annotation] |
| AAEL026440 | NA | 0.3655 | 4.9868 | 0.6852 | 0.9996 | NA |
| AAEL003989 | | -0.3552 | 6.5554 | 0.6853 | 0.9996 | GTP-binding  protein alpha subunit, gna [Source:VB Community Annotation] |
| AAEL022413 | NA | -0.5295 | 3.1161 | 0.6853 | 0.9996 | NA |
| AAEL009214 | | -0.3246 | 8.6586 | 0.6854 | 0.9996 | diazepam binding inhibitor, putative [Source:VB Community Annotation] |
| AAEL003271 | | 0.3583 | 0.4216 | 0.6857 | 0.9996 | |
| AAEL000805 | | 0.4018 | 3.2820 | 0.6858 | 0.9996 | |
| AAEL004520 | | 0.1114 | 5.1652 | 0.6858 | 0.9996 | cAMP/cgmp cyclic nucleotide phosphodiesterase [Source:VB Community Annotation] |
| AAEL005232 | | 0.1686 | 2.6833 | 0.6858 | 0.9996 | cyclin-dependent kinases regulatory subunit. putative [Source:VB Community Annotation] |
| AAEL001257 | | -0.4334 | 0.9366 | 0.6859 | 0.9996 | sugar transporter [Source:VB Community Annotation] |
| AAEL009050 | | -0.3796 | -0.3513 | 0.6860 | 0.9996 | amine oxidase [Source:VB Community Annotation] |
| AAEL001752 | | -0.4380 | 2.4361 | 0.6860 | 0.9996 | chloride channel protein 3 [Source:VB Community Annotation] |
| AAEL012744 | | 0.1281 | 4.9121 | 0.6860 | 0.9996 | |
| AAEL011677 | | -0.2253 | 3.4501 | 0.6862 | 0.9996 | AMP dependent coa ligase [Source:VB Community Annotation] |
| AAEL013733 | | -0.2039 | 3.3046 | 0.6863 | 0.9996 | Protein distal antenna [Source:VB Community Annotation] |
| AAEL008898 | | -0.2035 | 4.8252 | 0.6864 | 0.9996 | sulfotransferase (sult) [Source:VB Community Annotation] |
| AAEL004980 | | 0.2621 | 2.3662 | 0.6864 | 0.9996 | Ubiquitinyl hydrolase 1 [Source:UniProtKB/TrEMBL;Acc:Q17BE1] |
| AAEL018669 | COX3 | 0.5831 | -0.5800 | 0.6869 | 0.9996 | cytochrome c oxidase subunit III [Source:European Nucleotide Archive;Acc:COX3] |
| AAEL014325 | | 0.1379 | 5.5136 | 0.6870 | 0.9996 | proteasome regulatory subunits [Source:VB Community Annotation] |
| AAEL002499 | | -0.1841 | 2.9738 | 0.6870 | 0.9996 | |
| AAEL011633 | | 0.4707 | 2.5502 | 0.6870 | 0.9996 | fibrinogen and fibronectin [Source:VB Community Annotation] |
| AAEL003508 | | 0.2682 | 2.7655 | 0.6871 | 0.9996 | serine-pyruvate aminotransferase [Source:VB Community Annotation] |
| AAEL020185 | NA | 0.4072 | 0.4453 | 0.6872 | 0.9996 | NA |
| AAEL011050 | | 0.1657 | 4.1785 | 0.6872 | 0.9996 | |
| AAEL008635 | | -0.1489 | 5.3826 | 0.6872 | 0.9996 | ABC transporter [Source:VB Community Annotation] |
| AAEL011793 | | -0.1124 | 5.4548 | 0.6874 | 0.9996 | aspartyl beta-hydroxylase, putative [Source:VB Community Annotation] |
| AAEL001190 | | 0.1529 | 7.0926 | 0.6874 | 0.9996 | |
| AAEL002811 | | -0.4522 | 4.4585 | 0.6877 | 0.9996 | |
| AAEL022493 | NA | 0.1420 | 4.2572 | 0.6877 | 0.9996 | NA |
| AAEL002350 | | -0.1233 | 7.5802 | 0.6878 | 0.9996 | |
| AAEL017071 | | 0.4360 | -0.8775 | 0.6878 | 0.9996 | Carboxylic ester hydrolase (Fragment) [Source:UniProtKB/TrEMBL;Acc:J9HJ23] |
| AAEL008079 | | 0.7821 | 2.1442 | 0.6878 | 0.9996 | trypsin-alpha, putative [Source:VB Community Annotation] |
| AAEL009978 | | 0.5094 | 0.3999 | 0.6880 | 0.9996 | |
| AAEL010376 | | 0.2821 | 0.9000 | 0.6881 | 0.9996 | |
| AAEL012723 | | 0.1493 | 4.5467 | 0.6881 | 0.9996 | mitogen activated protein kinase kinase 2, mapkk2, mek2 [Source:VB Community Annotation] |
| AAEL019466 | NA | -0.5438 | 1.5634 | 0.6882 | 0.9996 | NA |
| AAEL000724 | | 0.1609 | 5.0697 | 0.6884 | 0.9996 | |

| | | | | | | |
|---|---|---|---|---|---|---|
| AAEL006836 | | 0.1854 | 6.5798 | 0.6884 | 0.9996 | dihydropteridine reductase [Source:VB Community Annotation] |
| AAEL002183 | | -0.0884 | 6.7337 | 0.6887 | 0.9996 | oligosaccharyl transferase, subunit, putative [Source:VB Community Annotation] |
| AAEL000636 | | 0.3134 | 1.9164 | 0.6887 | 0.9996 | |
| AAEL001998 | | -0.2637 | 6.2050 | 0.6887 | 0.9996 | |
| AAEL028814 | NA | -0.5348 | -1.3271 | 0.6888 | 0.9996 | NA |
| AAEL023353 | NA | -0.4023 | 5.3428 | 0.6891 | 0.9996 | NA |
| AAEL008579 | | -0.4900 | 5.7425 | 0.6891 | 0.9996 | zinc finger protein [Source:VB Community Annotation] |
| AAEL001916 | | -0.0885 | 6.9576 | 0.6891 | 0.9996 | eukaryotic translation initiation factor 4e [Source:VB Community Annotation] |
| AAEL008044 | | -0.1191 | 4.9942 | 0.6892 | 0.9996 | |
| AAEL002174 | | 0.1291 | 7.3291 | 0.6893 | 0.9996 | dolichyl-diphosphooligosaccharide protein glycosyltransferase [Source:VB Community Annotation] |
| AAEL001673 | | 0.4100 | 8.4828 | 0.6893 | 0.9996 | actin [Source:VB Community Annotation] |
| AAEL000370 | | 0.0930 | 4.7188 | 0.6895 | 0.9996 | acyl-CoA oxidase [Source:VB Community Annotation] |
| AAEL021418 | NA | -0.0941 | 6.1886 | 0.6897 | 0.9996 | NA |
| AAEL000312 | | -0.2291 | 2.8028 | 0.6898 | 0.9996 | |
| AAEL026751 | NA | 0.4603 | 2.9574 | 0.6899 | 0.9996 | NA |
| AAEL009486 | | -0.0841 | 5.6080 | 0.6899 | 0.9996 | ectonucleotide pyrophosphatase/phosphodiesterase [Source:VB Community Annotation] |
| AAEL003998 | | -0.1475 | 2.3884 | 0.6900 | 0.9996 | |
| AAEL025410 | NA | -0.6170 | 5.7158 | 0.6900 | 0.9996 | NA |
| AAEL019590 | NA | -0.1818 | 6.0006 | 0.6900 | 0.9996 | NA |
| AAEL015255 | | -0.2011 | 5.5712 | 0.6900 | 0.9996 | phosphatidylinositol-4-phosphate 5-kinase type i [Source:VB Community Annotation] |
| AAEL012482 | | -0.4882 | 2.2427 | 0.6903 | 0.9996 | |
| AAEL019407 | NA | 0.7160 | 0.5118 | 0.6905 | 0.9996 | NA |
| AAEL007670 | | 0.5841 | 1.3224 | 0.6905 | 0.9996 | tiptop [Source:VB Community Annotation] |
| AAEL005766 | | -0.1333 | 9.5852 | 0.6906 | 0.9996 | Fructose-bisphosphate aldolase [Source:UniProtKB/TrEMBL;Acc:Q178U8] |
| AAEL003817 | | 0.0945 | 4.8767 | 0.6908 | 0.9996 | kappa b-ras [Source:VB Community Annotation] |
| AAEL002001 | | 0.5223 | -2.1933 | 0.6909 | 0.9996 | protein serine/threonine kinase, putative [Source:VB Community Annotation] |
| AAEL019916 | NA | -0.2614 | 2.4834 | 0.6910 | 0.9996 | NA |
| AAEL026915 | NA | -0.6248 | -0.4724 | 0.6911 | 0.9996 | NA |
| AAEL019968 | NA | 0.1745 | 1.6713 | 0.6911 | 0.9996 | NA |
| AAEL004179 | | -0.3632 | -0.7341 | 0.6912 | 0.9996 | |
| AAEL021542 | NA | 0.1393 | 6.4262 | 0.6912 | 0.9996 | NA |
| AAEL001548 | | 0.1451 | 5.7022 | 0.6915 | 0.9996 | glucosyl/glucuronosyl transferases [Source:VB Community Annotation] |
| AAEL004517 | | 0.1205 | 4.7733 | 0.6915 | 0.9996 | |
| AAEL022180 | NA | 0.1378 | 3.7971 | 0.6916 | 0.9996 | NA |
| AAEL009756 | | -0.2306 | 4.5957 | 0.6917 | 0.9996 | |
| AAEL011780 | | -0.1319 | 3.4976 | 0.6917 | 0.9996 | DNA mismatch repair protein muts [Source:VB Community Annotation] |
| AAEL002169 | | -0.4303 | 5.6443 | 0.6918 | 0.9996 | |
| AAEL005165 | | -0.1439 | 9.2532 | 0.6918 | 0.9996 | chaperone protein DNAj [Source:VB Community Annotation] |
| AAEL004376 | | 0.1180 | 3.6658 | 0.6918 | 0.9996 | |
| AAEL002935 | | 0.1576 | 4.1075 | 0.6919 | 0.9996 | |

| | | | | | | |
|---|---|---|---|---|---|---|
| AAEL010434 | VGA1 | 0.5182 | -1.5608 | 0.6920 | 0.9996 | vitellogenin-A1 precursor [Source:VB Community Annotation] |
| AAEL017799 | U1 | -0.6357 | 1.1972 | 0.6920 | 0.9996 | U1 spliceosomal RNA [Source:RFAM;Acc:RF00003] |
| AAEL020949 | NA | -1.0502 | 2.9757 | 0.6921 | 0.9996 | NA |
| AAEL021211 | NA | -0.5986 | 2.0847 | 0.6921 | 0.9996 | NA |
| AAEL019837 | NA | -0.5382 | 4.5056 | 0.6921 | 0.9996 | NA |
| AAEL013756 | | -0.1795 | 5.7743 | 0.6922 | 0.9996 | zinc/iron transporter [Source:VB Community Annotation] |
| AAEL012443 | | -0.3630 | 3.6689 | 0.6922 | 0.9996 | sugar transporter [Source:VB Community Annotation] |
| AAEL019716 | NA | -0.3091 | 4.9423 | 0.6924 | 0.9996 | NA |
| AAEL000074 | CLIPB1 | -0.2703 | 7.5335 | 0.6928 | 0.9996 | Clip-Domain Serine Protease family B. [Source:VB Community Annotation] |
| AAEL007276 | | -0.2604 | 0.6548 | 0.6933 | 0.9996 | |
| AAEL002761 | | 0.2751 | 8.0745 | 0.6934 | 0.9996 | tropomyosin invertebrate [Source:VB Community Annotation] |
| AAEL020285 | NA | -0.2275 | 5.9190 | 0.6934 | 0.9996 | NA |
| AAEL006648 | | 0.1035 | 4.9738 | 0.6935 | 0.9996 | pantothenate kinase [Source:VB Community Annotation] |
| AAEL019787 | NA | -0.5927 | 1.5444 | 0.6937 | 0.9996 | NA |
| AAEL011993 | | 0.1329 | 4.2070 | 0.6937 | 0.9996 | |
| AAEL013261 | | -0.4218 | 0.4602 | 0.6937 | 0.9996 | |
| AAEL024710 | NA | 0.1351 | 4.5750 | 0.6939 | 0.9996 | NA |
| AAEL021519 | NA | 0.3966 | 0.4766 | 0.6940 | 0.9996 | NA |
| AAEL006124 | | -0.1105 | 4.9216 | 0.6940 | 0.9996 | |
| AAEL006492 | | -0.2910 | 0.5887 | 0.6940 | 0.9996 | centrin, putative [Source:VB Community Annotation] |
| AAEL008450 | | -0.1768 | 3.1217 | 0.6941 | 0.9996 | |
| AAEL008577 | | 0.1233 | 4.5792 | 0.6941 | 0.9996 | |
| AAEL004527 | mRpS7 | -0.1519 | 6.6243 | 0.6941 | 0.9996 | mitochondrial ribosomal protein S7 [Source:VB Community Annotation] |
| AAEL027350 | NA | -0.1319 | 2.7616 | 0.6942 | 0.9996 | NA |
| AAEL017455 | | -0.2915 | 6.0751 | 0.6943 | 0.9996 | |
| AAEL016992 | | -0.6685 | -0.7680 | 0.6943 | 0.9996 | |
| AAEL000429 | | 0.1745 | 2.8232 | 0.6944 | 0.9996 | |
| AAEL013191 | | 0.2408 | 4.1345 | 0.6946 | 0.9996 | |
| AAEL013882 | | 0.6981 | 2.5003 | 0.6946 | 0.9996 | tkr [Source:VB Community Annotation] |
| AAEL010917 | | -0.5699 | -0.1662 | 0.6948 | 0.9996 | organic anion transporter [Source:VB Community Annotation] |
| AAEL021495 | NA | 0.1762 | 3.3534 | 0.6948 | 0.9996 | NA |
| AAEL005958 | | 0.1476 | 4.2838 | 0.6951 | 0.9996 | oxidoreductase [Source:VB Community Annotation] |
| AAEL012167 | | -0.1240 | 4.7027 | 0.6951 | 0.9996 | elongation factor tu (ef-tu) [Source:VB Community Annotation] |
| AAEL012317 | | 0.1405 | 7.8957 | 0.6952 | 0.9996 | MICOS complex subunit MIC13 [Source:UniProtKB/TrEMBL;Acc:Q1HRM0] |
| AAEL027688 | NA | 0.1994 | 2.1016 | 0.6954 | 0.9996 | NA |
| AAEL007455 | | -0.4376 | 2.2238 | 0.6955 | 0.9996 | thrombospondin [Source:VB Community Annotation] |
| AAEL003301 | | -0.2110 | 5.1036 | 0.6956 | 0.9996 | MRAS2, putative [Source:VB Community Annotation] |
| AAEL023057 | NA | -0.2211 | 1.7166 | 0.6957 | 0.9996 | NA |
| AAEL011317 | | -0.1139 | 5.1301 | 0.6957 | 0.9996 | actin [Source:VB Community Annotation] |
| AAEL003077 | | -0.1454 | 4.0065 | 0.6958 | 0.9996 | |

| | | | | | | |
|---|---|---|---|---|---|---|
| AAEL001432 | | -0.0877 | 7.5869 | 0.6958 | 0.9996 | protein disulfide isomerase [Source:VB Community Annotation] |
| AAEL022251 | NA | -0.3466 | 3.0182 | 0.6958 | 0.9996 | NA |
| AAEL012311 | | 0.2496 | 8.0160 | 0.6958 | 0.9996 | vitellogenin, putative [Source:VB Community Annotation] |
| AAEL006888 | | -0.2482 | 3.6323 | 0.6958 | 0.9996 | |
| AAEL023617 | NA | 0.1404 | 5.2987 | 0.6960 | 0.9996 | NA |
| AAEL026800 | NA | 0.1653 | 1.6191 | 0.6960 | 0.9996 | NA |
| AAEL008366 | | -0.1579 | 4.9527 | 0.6960 | 0.9996 | pyruvate dehydrogenase [Source:VB Community Annotation] |
| AAEL014546 | | 0.1420 | 3.4777 | 0.6960 | 0.9996 | |
| AAEL009431 | mRpL10 | 0.1499 | 5.8313 | 0.6963 | 0.9996 | mitochondrial ribosomal protein, L10, putative [Source:VB Community Annotation] |
| AAEL005384 | | 0.2674 | 6.1733 | 0.6964 | 0.9996 | phosphoribosylformylglycinamidine synthase, putative [Source:VB Community Annotation] |
| AAEL004007 | | 0.1488 | 2.1555 | 0.6965 | 0.9996 | GTP binding protein (mitochondrial), putative [Source:VB Community Annotation] |
| AAEL004661 | | 0.1529 | 5.2182 | 0.6966 | 0.9996 | beta-hexosaminidase [Source:VB Community Annotation] |
| AAEL009093 | | -0.3640 | -0.4473 | 0.6966 | 0.9996 | crumbs [Source:VB Community Annotation] |
| AAEL021868 | NA | -0.1496 | 4.1940 | 0.6966 | 0.9996 | NA |
| AAEL000597 | | -0.1425 | 6.7367 | 0.6968 | 0.9996 | cadherin [Source:VB Community Annotation] |
| AAEL001695 | | 0.2016 | 2.5875 | 0.6969 | 0.9996 | orthopedia homeobox protein [Source:VB Community Annotation] |
| AAEL023679 | NA | 0.1628 | 3.1243 | 0.6969 | 0.9996 | NA |
| AAEL004399 | GPRFSH | -0.1705 | 4.7339 | 0.6969 | 0.9996 | GPCR Glycoprotein Hormone Family [Source:VB Community Annotation] |
| AAEL014828 | | 0.1158 | 5.2979 | 0.6970 | 0.9996 | |
| AAEL003590 | | -1.1140 | 3.3338 | 0.6971 | 0.9996 | |
| AAEL025230 | NA | -0.2778 | 0.5644 | 0.6972 | 0.9996 | NA |
| AAEL024914 | NA | -0.3389 | 4.0147 | 0.6972 | 0.9996 | NA |
| AAEL002544 | | -0.1643 | 4.8114 | 0.6972 | 0.9996 | |
| AAEL022132 | NA | -0.2426 | 3.4752 | 0.6972 | 0.9996 | NA |
| AAEL021846 | NA | -0.7502 | 2.3262 | 0.6973 | 0.9996 | NA |
| AAEL002600 | | -0.2461 | 8.5803 | 0.6973 | 0.9996 | serine protease [Source:VB Community Annotation] |
| AAEL011629 | | 0.1932 | 3.5464 | 0.6973 | 0.9996 | tyrosyl-DNA phosphodiesterase [Source:VB Community Annotation] |
| AAEL014437 | | -0.1247 | 5.7111 | 0.6975 | 0.9996 | |
| AAEL011323 | Hnf4 | -0.1524 | 5.3961 | 0.6975 | 0.9996 | Hepatocyte nuclear factor 4 isoform A nuclear receptor [Source:VB Community Annotation] |
| AAEL025437 | NA | 0.0994 | 4.9154 | 0.6976 | 0.9996 | NA |
| AAEL004211 | | 0.7335 | 0.1271 | 0.6977 | 0.9996 | |
| AAEL007102 | | -0.2208 | 5.4246 | 0.6977 | 0.9996 | trypsin, putative [Source:VB Community Annotation] |
| AAEL009016 | GSTT2 | 0.2095 | 2.1188 | 0.6978 | 0.9996 | glutathione transferase [Source:VB Community Annotation] |
| AAEL020635 | NA | 0.1987 | 2.0886 | 0.6979 | 0.9996 | NA |
| AAEL002989 | | -0.1566 | 4.5165 | 0.6979 | 0.9996 | |
| AAEL011345 | | -0.3648 | 5.3688 | 0.6980 | 0.9996 | |
| AAEL008821 | | 0.2669 | 1.4498 | 0.6981 | 0.9996 | |
| AAEL020615 | NA | -0.1534 | 2.4688 | 0.6986 | 0.9996 | NA |
| AAEL002221 | | -0.0984 | 4.4745 | 0.6986 | 0.9996 | calcium and integrin-binding protein 1 [Source:VB Community Annotation] |
| AAEL018331 | | -0.1385 | 5.6152 | 0.6988 | 0.9996 | |

| | | | | | | |
|---|---|---|---|---|---|---|
| AAEL011927 | | -0.1807 | 2.1943 | 0.6989 | 0.9996 | mind bomb [Source:VB Community Annotation] |
| AAEL016039 | tRNA-Asp | 0.3700 | 0.7695 | 0.6989 | 0.9996 | |
| AAEL012661 | eIF3-S4-1 | 0.1337 | 8.2460 | 0.6989 | 0.9996 | Eukaryotic translation initiation factor 3 subunit G (eIF3g)(Eukaryotic translation initiation factor 3 subunit 4)(Eukaryotic translation initia |
| AAEL015041 | | -0.1884 | 4.7917 | 0.6990 | 0.9996 | mitochondrial carrier [Source:VB Community Annotation] |
| AAEL005528 | | -0.2644 | 3.7680 | 0.6991 | 0.9996 | |
| AAEL013345 | | 0.5617 | -1.3315 | 0.6991 | 0.9996 | alphaA-crystallin, putative [Source:VB Community Annotation] |
| AAEL010480 | | -0.4436 | 1.9437 | 0.6991 | 0.9996 | serine-pyruvate aminotransferase [Source:VB Community Annotation] |
| AAEL021444 | NA | 0.5564 | -0.5671 | 0.6991 | 0.9996 | NA |
| AAEL007043 | | -0.2479 | 5.9461 | 0.6991 | 0.9996 | |
| AAEL002784 | | 0.1484 | 4.6489 | 0.6991 | 0.9996 | zinc finger protein [Source:VB Community Annotation] |
| AAEL006885 | 14-3-3zeta | -0.1079 | 10.8484 | 0.6992 | 0.9996 | 14-3-3 protein zeta [Source:UniProtKB/Swiss-Prot;Acc:Q1HR36] |
| AAEL002944 | | 0.1134 | 4.1726 | 0.6993 | 0.9996 | |
| AAEL006209 | | -0.1329 | 3.1858 | 0.6995 | 0.9996 | exonuclease [Source:VB Community Annotation] |
| AAEL009308 | | -0.1829 | 3.2527 | 0.6996 | 0.9996 | WD-repeat protein [Source:VB Community Annotation] |
| AAEL011302 | | 0.0889 | 8.1158 | 0.6998 | 0.9996 | Annexin [Source:UniProtKB/TrEMBL;Acc:Q16QE7] |
| AAEL004309 | | -0.1162 | 4.3151 | 0.6999 | 0.9996 | |
| AAEL002822 | Arp5 | 0.2490 | 2.7925 | 0.7000 | 0.9996 | Actin-related protein 5 [Source:UniProtKB/Swiss-Prot;Acc:Q17GZ9] |
| AAEL025396 | NA | -0.4287 | -1.1737 | 0.7001 | 0.9996 | NA |
| AAEL001417 | LRIM7 | 0.2059 | 2.7950 | 0.7001 | 0.9996 | leucine-rich immune protein (Short) [Source:VB Community Annotation] |
| AAEL001127 | | -0.6369 | 1.2214 | 0.7002 | 0.9996 | defective proboscis extension response, putative [Source:VB Community Annotation] |
| AAEL006231 | | -0.1689 | 4.2072 | 0.7003 | 0.9996 | paraflagellar rod protein, putative [Source:VB Community Annotation] |
| AAEL019849 | NA | -0.4709 | 4.4286 | 0.7003 | 0.9996 | NA |
| AAEL008696 | | -0.2225 | 4.7305 | 0.7005 | 0.9996 | smad [Source:VB Community Annotation] |
| AAEL003723 | LYSC11 | -0.4040 | 5.8433 | 0.7005 | 0.9996 | C-Type Lysozyme (Lys-A). [Source:VB Community Annotation] |
| AAEL012710 | | -0.2930 | 5.1839 | 0.7005 | 0.9996 | |
| AAEL025839 | NA | -0.4264 | -1.2182 | 0.7006 | 0.9996 | NA |
| AAEL021370 | NA | 0.1855 | 2.6193 | 0.7008 | 0.9996 | NA |
| AAEL006485 | | -0.6568 | 8.4933 | 0.7008 | 0.9996 | inosine-uridine preferring nucleoside hydrolase [Source:VB Community Annotation] |
| AAEL018294 | | -0.2706 | 4.5134 | 0.7009 | 0.9996 | |
| AAEL012410 | AGO1b | -0.2880 | 6.3603 | 0.7009 | 0.9996 | eukaryotic translation initiation factor 2C [Source:VB Community Annotation] |
| AAEL027240 | NA | 0.1167 | 6.1373 | 0.7010 | 0.9996 | NA |
| AAEL011905 | | -0.2737 | 3.0168 | 0.7011 | 0.9996 | myosin i [Source:VB Community Annotation] |
| AAEL006575 | | -0.3217 | 5.2194 | 0.7014 | 0.9996 | troponin C [Source:VB Community Annotation] |
| AAEL006993 | | -0.6517 | 2.4679 | 0.7015 | 0.9996 | synapse-associated protein [Source:VB Community Annotation] |
| AAEL000388 | | 0.1370 | 5.3307 | 0.7015 | 0.9996 | isoleucyl tRNA synthetase [Source:VB Community Annotation] |
| AAEL010791 | | 0.1555 | 4.6877 | 0.7015 | 0.9996 | Autophagy-specific protein, putative [Source:VB Community Annotation] |
| AAEL010058 | Gr3 | 0.1703 | 3.2525 | 0.7016 | 0.9996 | gustatory receptor Gr3 [Source:VB Community Annotation] |
| AAEL027672 | NA | -0.1832 | 2.6860 | 0.7016 | 0.9996 | NA |
| AAEL009128 | CYP6M6 | 0.1400 | 3.9795 | 0.7017 | 0.9996 | cytochrome P450 [Source:VB Community Annotation] |
| AAEL026906 | NA | -0.3833 | 3.0688 | 0.7018 | 0.9996 | NA |

| | | | | | | |
|---|---|---|---|---|---|---|
| AAEL007820 | | -0.1239 | 4.8164 | 0.7018 | 0.9996 | |
| AAEL011069 | | -0.2903 | 4.7553 | 0.7018 | 0.9996 | |
| AAEL004602 | | -0.2429 | 1.5874 | 0.7019 | 0.9996 | neurogenic differentiation factor, putative [Source:VB Community Annotation] |
| AAEL019781 | NA | -0.2012 | 4.8768 | 0.7020 | 0.9996 | NA |
| AAEL008425 | | -0.1062 | 6.5701 | 0.7021 | 0.9996 | Derlin (Fragment) [Source:UniProtKB/TrEMBL;Acc:Q16YT7] |
| AAEL020042 | NA | -0.1687 | 4.6078 | 0.7023 | 0.9996 | NA |
| AAEL002419 | | -0.2552 | 2.4893 | 0.7024 | 0.9996 | sarcolemmal associated protein-2, putative [Source:VB Community Annotation] |
| AAEL013877 | Gnpda1 | -0.1547 | 3.3831 | 0.7026 | 0.9996 | Glucosamine-6-phosphate isomerase (EC 3.5.99.6)(Glucosamine-6-phosphate deaminase)(GlcN6P deaminase)(GNPDA) [Source:VB Comn |
| AAEL008805 | | 0.3663 | 0.4438 | 0.7028 | 0.9996 | |
| AAEL008622 | | 0.4450 | -0.5639 | 0.7029 | 0.9996 | jnk [Source:VB Community Annotation] |
| AAEL026901 | NA | 0.5479 | 0.8040 | 0.7031 | 0.9996 | NA |
| AAEL028129 | NA | -0.2632 | 1.4255 | 0.7032 | 0.9996 | NA |
| AAEL006337 | | 0.1214 | 5.9555 | 0.7032 | 0.9996 | |
| AAEL001082 | | -0.3176 | 7.5407 | 0.7033 | 0.9996 | |
| AAEL010881 | | 0.5191 | -0.9418 | 0.7033 | 0.9996 | netrin receptor (unc5) [Source:VB Community Annotation] |
| AAEL005729 | | 0.2527 | -0.4350 | 0.7033 | 0.9996 | |
| AAEL010053 | | -0.1451 | 4.2252 | 0.7034 | 0.9996 | |
| AAEL024953 | NA | 0.1454 | 4.2789 | 0.7034 | 0.9996 | NA |
| AAEL013245 | CLIPB28 | -0.2514 | 2.8138 | 0.7035 | 0.9996 | Clip-Domain Serine Protease  family B. [Source:VB Community Annotation] |
| AAEL011332 | | -0.3020 | 3.5573 | 0.7035 | 0.9996 | dopamine beta hydroxylase [Source:VB Community Annotation] |
| AAEL003626 | | -0.3254 | 2.9555 | 0.7035 | 0.9996 | sodium/chloride dependent amino acid transporter [Source:VB Community Annotation] |
| AAEL005643 | | 0.8776 | 1.4146 | 0.7035 | 0.9996 | guanine nucleotide exchange factor [Source:VB Community Annotation] |
| AAEL022025 | NA | -0.1359 | 4.2934 | 0.7037 | 0.9996 | NA |
| AAEL013228 | | -0.3902 | 2.9745 | 0.7039 | 0.9996 | |
| AAEL010558 | | -0.5470 | -0.0679 | 0.7042 | 0.9996 | |
| AAEL017464 | | -0.3778 | 2.8097 | 0.7042 | 0.9996 | |
| AAEL013628 | | -0.5271 | 2.6450 | 0.7043 | 0.9996 | trypsin-eta, putative [Source:VB Community Annotation] |
| AAEL013538 | | 0.1270 | 4.2132 | 0.7043 | 0.9996 | hepatoma-derived GF, putative [Source:VB Community Annotation] |
| AAEL021233 | NA | -0.1685 | 2.6235 | 0.7048 | 0.9996 | NA |
| AAEL017293 | | -0.3072 | 6.7317 | 0.7049 | 0.9996 | |
| AAEL007001 | | 0.1211 | 5.7336 | 0.7050 | 0.9996 | mitochondrial import receptor subunit tom40 [Source:VB Community Annotation] |
| AAEL006741 | | -0.1164 | 8.7094 | 0.7052 | 0.9996 | |
| AAEL007271 | | -0.3316 | 2.5846 | 0.7052 | 0.9996 | basic helix-loop-helix zip transcription factor [Source:VB Community Annotation] |
| AAEL019932 | NA | -0.2686 | 4.7426 | 0.7053 | 0.9996 | NA |
| AAEL027483 | NA | 0.3349 | 2.1592 | 0.7053 | 0.9996 | NA |
| AAEL010483 | ORP8 | -0.2437 | 4.8480 | 0.7054 | 0.9996 | oxysterol-binding protein related protein (ORP8) [Source:VB Community Annotation] |
| AAEL018225 | | 0.2610 | 3.3874 | 0.7054 | 0.9996 | |
| AAEL002721 | | -0.4509 | 1.1220 | 0.7054 | 0.9996 | |
| AAEL009475 | | -0.2332 | 3.0723 | 0.7055 | 0.9996 | carbamoyl-phosphate synthase large chain [Source:VB Community Annotation] |
| AAEL002446 | | 0.1672 | 3.3368 | 0.7055 | 0.9996 | |

| | | | | | | |
|---|---|---|---|---|---|---|
| AAEL001809 | | 0.2437 | 1.8749 | 0.7056 | 0.9996 | |
| AAEL001366 | | -0.1473 | 3.7662 | 0.7057 | 0.9996 | |
| AAEL004824 | | -0.2287 | 4.8376 | 0.7062 | 0.9996 | |
| AAEL018305 | | -0.1873 | 2.1021 | 0.7062 | 0.9996 | |
| AAEL009748 | | 0.5086 | -0.8208 | 0.7063 | 0.9996 | 2-hydroxyphytanoyl-coa lyase [Source:VB Community Annotation] |
| AAEL010406 | | 0.4046 | 0.7584 | 0.7064 | 0.9996 | |
| AAEL000609 | | -0.1646 | 3.0009 | 0.7064 | 0.9996 | |
| AAEL024717 | NA | 0.1879 | 3.8241 | 0.7065 | 0.9996 | NA |
| AAEL012337 | | -0.4267 | 5.9198 | 0.7065 | 0.9996 | goliath E3 ubiquitin ligase [Source:VB Community Annotation] |
| AAEL004775 | | 0.3483 | 0.4422 | 0.7065 | 0.9996 | |
| AAEL011261 | | -0.3455 | 2.5428 | 0.7067 | 0.9996 | yemanuclein [Source:VB Community Annotation] |
| AAEL027055 | NA | -0.1707 | 5.0276 | 0.7067 | 0.9996 | NA |
| AAEL015338 | | -0.1421 | 3.9274 | 0.7067 | 0.9996 | |
| AAEL022350 | NA | -0.4473 | -0.2335 | 0.7067 | 0.9996 | NA |
| AAEL003435 | | 0.1309 | 3.2122 | 0.7068 | 0.9996 | |
| AAEL011161 | | 0.1928 | 5.0763 | 0.7068 | 0.9996 | |
| AAEL020132 | NA | 0.5252 | 3.0366 | 0.7068 | 0.9996 | NA |
| AAEL025608 | NA | 0.9523 | 5.7538 | 0.7069 | 0.9996 | NA |
| AAEL001402 | LRIM10B | -0.2398 | 7.0406 | 0.7069 | 0.9996 | leucine-rich immune protein (Short) [Source:VB Community Annotation] |
| AAEL005997 | | -0.2809 | 3.1494 | 0.7070 | 0.9996 | allergen, putative [Source:VB Community Annotation] |
| AAEL003908 | | -0.1179 | 3.8358 | 0.7072 | 0.9996 | nuclear pore complex protein nup107 [Source:VB Community Annotation] |
| AAEL019711 | NA | -0.5371 | 3.3633 | 0.7072 | 0.9996 | NA |
| AAEL025613 | NA | 0.1994 | 2.1313 | 0.7072 | 0.9996 | NA |
| AAEL020006 | NA | -0.5050 | -0.8752 | 0.7073 | 0.9996 | NA |
| AAEL015077 | | -0.0941 | 5.6118 | 0.7074 | 0.9996 | WD and tetratricopeptide repeat protein [Source:VB Community Annotation] |
| AAEL008464 | | -0.6183 | -1.6864 | 0.7075 | 0.9996 | |
| AAEL000730 | | -0.2856 | 3.2454 | 0.7078 | 0.9996 | |
| AAEL022735 | NA | 0.1380 | 4.8406 | 0.7078 | 0.9996 | NA |
| AAEL000980 | | 0.4898 | -0.3885 | 0.7079 | 0.9996 | |
| AAEL005617 | | 0.1985 | 6.5366 | 0.7079 | 0.9996 | UTP-glucose-1-phosphate uridylyltransferase 2 [Source:VB Community Annotation] |
| AAEL025974 | NA | -0.6465 | -1.1682 | 0.7080 | 0.9996 | NA |
| AAEL011210 | | 0.3004 | 1.4077 | 0.7082 | 0.9996 | |
| AAEL009949 | | 0.2009 | 3.0714 | 0.7082 | 0.9996 | homeotic antennapedia protein, putative [Source:VB Community Annotation] |
| AAEL023144 | NA | 0.1271 | 2.8343 | 0.7083 | 0.9996 | NA |
| AAEL011171 | | -0.3188 | 3.7453 | 0.7084 | 0.9996 | low-Mr GTP-binding protein Rab31, putative [Source:VB Community Annotation] |
| AAEL006691 | | -0.6952 | 1.8537 | 0.7084 | 0.9996 | fibrinogen and fibronectin [Source:VB Community Annotation] |
| AAEL024504 | NA | -0.1234 | 4.1480 | 0.7085 | 0.9996 | NA |
| AAEL006833 | | -0.1104 | 8.4751 | 0.7086 | 0.9996 | succinyl-CoA synthetase small subunit, putative [Source:VB Community Annotation] |
| AAEL024337 | NA | 0.1958 | 2.6791 | 0.7088 | 0.9996 | NA |
| AAEL017262 | | -0.2481 | 11.9799 | 0.7088 | 0.9996 | |

| | | | | | | |
|---|---|---|---|---|---|---|
| AAEL011967 | cry2 | -0.1862 | 5.9580 | 0.7088 | 0.9996 | DNA photolyase [Source:VB Community Annotation] |
| AAEL019956 | NA | -0.3795 | 6.5386 | 0.7089 | 0.9996 | NA |
| AAEL004935 | | 0.3858 | 2.2173 | 0.7090 | 0.9996 | nicotinic acetylcholine receptor, beta-2 subunit, putative [Source:VB Community Annotation] |
| AAEL004528 | | 0.1673 | 3.0432 | 0.7091 | 0.9996 | |
| AAEL013374 | | -0.4638 | 4.5356 | 0.7091 | 0.9996 | disconnected protein [Source:VB Community Annotation] |
| AAEL019602 | NA | 0.1378 | 3.7510 | 0.7092 | 0.9996 | NA |
| AAEL005297 | | -0.3447 | -0.2582 | 0.7093 | 0.9996 | guanine nucleotide exchange factor [Source:VB Community Annotation] |
| AAEL013113 | | 0.3567 | 0.6802 | 0.7094 | 0.9996 | metallo-beta-lactamase, putative [Source:VB Community Annotation] |
| AAEL003150 | | -0.1871 | 2.9025 | 0.7094 | 0.9996 | alpha-n-acetylglucosaminidase [Source:VB Community Annotation] |
| AAEL026834 | NA | -0.4093 | 4.5310 | 0.7095 | 0.9996 | NA |
| AAEL023510 | NA | -0.7184 | 1.8135 | 0.7095 | 0.9996 | NA |
| AAEL005252 | Crz | 0.4006 | 1.9932 | 0.7095 | 0.9996 | corazonin [Source:VB Community Annotation] |
| AAEL004580 | | -0.2446 | 0.9346 | 0.7095 | 0.9996 | Beta-galactosidase [Source:UniProtKB/TrEMBL;Acc:Q17CH4] |
| AAEL005490 | | -0.6214 | 0.7978 | 0.7096 | 0.9996 | microsomal dipeptidase [Source:VB Community Annotation] |
| AAEL011303 | | -0.1653 | 2.5530 | 0.7096 | 0.9996 | cell division protein ftsj [Source:VB Community Annotation] |
| AAEL008220 | | 0.2585 | 4.0631 | 0.7096 | 0.9996 | |
| AAEL011587 | RpL27 | -0.0962 | 10.5524 | 0.7096 | 0.9996 | 60S ribosomal protein L27, putative [Source:VB Community Annotation] |
| AAEL006274 | | -0.6282 | 0.1665 | 0.7098 | 0.9996 | glucose transporter (sugar transporter [Source:VB Community Annotation] |
| AAEL019604 | NA | -0.4393 | 3.9895 | 0.7098 | 0.9996 | NA |
| AAEL019926 | NA | -0.5226 | 1.5247 | 0.7099 | 0.9996 | NA |
| AAEL010926 | | 0.2616 | 1.2263 | 0.7099 | 0.9996 | |
| AAEL013272 | RpL27a | -0.1180 | 10.8562 | 0.7100 | 0.9996 | 60S ribosomal protein L37a [Source:VB Community Annotation] |
| AAEL004294 | | 0.1497 | 7.3677 | 0.7102 | 0.9996 | dihydrolipoamide acetyltransferase component of pyruvate dehydrogenase [Source:VB Community Annotation] |
| AAEL014281 | | -0.0828 | 5.5355 | 0.7102 | 0.9996 | |
| AAEL002881 | | 0.1350 | 11.5118 | 0.7103 | 0.9996 | NADH:ubiquinone dehydrogenase, putative [Source:VB Community Annotation] |
| AAEL008844 | | -0.2170 | 7.2238 | 0.7104 | 0.9996 | calcium-binding protein, putative [Source:VB Community Annotation] |
| AAEL003170 | | 0.1656 | 3.3641 | 0.7104 | 0.9996 | mitochondrial translational initiation factor [Source:VB Community Annotation] |
| AAEL019940 | NA | -0.4204 | 3.4403 | 0.7106 | 0.9996 | NA |
| AAEL007240 | | -0.1651 | 4.8746 | 0.7106 | 0.9996 | cdc42 GTPase-activating protein [Source:VB Community Annotation] |
| AAEL021600 | NA | -0.1590 | 7.6437 | 0.7107 | 0.9996 | NA |
| AAEL002022 | | -0.2349 | 4.5336 | 0.7108 | 0.9996 | protein serine/threonine kinase, putative [Source:VB Community Annotation] |
| AAEL018276 | | 0.1709 | 3.5961 | 0.7111 | 0.9996 | |
| AAEL014938 | | 0.3546 | 1.7172 | 0.7112 | 0.9996 | |
| AAEL010374 | | 0.1492 | 2.8622 | 0.7113 | 0.9996 | tyrosine-protein kinase transmembrane receptor [Source:VB Community Annotation] |
| AAEL004876 | | 0.2133 | 1.5944 | 0.7113 | 0.9996 | guanylate cyclase [Source:VB Community Annotation] |
| AAEL002563 | | 0.1519 | 4.2753 | 0.7114 | 0.9996 | nuclear matrix protein [Source:VB Community Annotation] |
| AAEL002371 | | 0.1301 | 4.2818 | 0.7114 | 0.9996 | |
| AAEL010003 | | 0.1650 | 3.7816 | 0.7116 | 0.9996 | splicesome protein [Source:VB Community Annotation] |
| AAEL001326 | | 0.1439 | 2.5849 | 0.7116 | 0.9996 | |
| AAEL002852 | | 0.1210 | 2.3001 | 0.7117 | 0.9996 | |

| | | | | | | |
|---|---|---|---|---|---|---|
| AAEL006895 | | 0.1312 | 5.6779 | 0.7117 | 0.9996 | phosphofructokinase [Source:VB Community Annotation] |
| AAEL022467 | NA | 0.1080 | 3.9677 | 0.7118 | 0.9996 | NA |
| AAEL010801 | | -0.1308 | 9.7437 | 0.7119 | 0.9996 | |
| AAEL009960 | | -0.4464 | -1.0646 | 0.7120 | 0.9996 | |
| AAEL003736 | | -0.1109 | 4.5250 | 0.7122 | 0.9996 | sec15 [Source:VB Community Annotation] |
| AAEL003708 | | -0.1006 | 4.7800 | 0.7123 | 0.9996 | ninjurin a [Source:VB Community Annotation] |
| AAEL020782 | NA | 0.2404 | 0.9525 | 0.7123 | 0.9996 | NA |
| AAEL027217 | NA | -0.1168 | 4.6229 | 0.7127 | 0.9996 | NA |
| AAEL009574 | | -0.1984 | 4.8627 | 0.7129 | 0.9996 | elongase, putative [Source:VB Community Annotation] |
| AAEL005882 | | -0.1885 | 0.9401 | 0.7129 | 0.9996 | |
| AAEL013841 | | 0.1105 | 4.4202 | 0.7130 | 0.9996 | paraplegin [Source:VB Community Annotation] |
| AAEL008652 | CCAPR | 0.2511 | 0.9973 | 0.7131 | 0.9996 | GPCR vasopressin family receptor 1 [Source:VB Community Annotation] |
| AAEL021291 | NA | 0.1380 | 3.1678 | 0.7132 | 0.9996 | NA |
| AAEL005646 | | 0.1129 | 4.1189 | 0.7133 | 0.9996 | vitellogenin, putative [Source:VB Community Annotation] |
| AAEL010346 | | 0.1873 | 2.2498 | 0.7134 | 0.9996 | |
| AAEL000410 | | -0.1130 | 6.3717 | 0.7134 | 0.9996 | glycoprotein 25l [Source:VB Community Annotation] |
| AAEL022276 | NA | 0.3887 | -2.5428 | 0.7135 | 0.9996 | NA |
| AAEL012902 | | -0.2113 | 3.9532 | 0.7136 | 0.9996 | heterogeneous nuclear ribonucleoprotein, putative [Source:VB Community Annotation] |
| AAEL013546 | | -0.6018 | 1.1197 | 0.7136 | 0.9996 | estrogen-related receptor (ERR) [Source:VB Community Annotation |
| AAEL004798 | | 0.3533 | 6.6226 | 0.7137 | 0.9996 | |
| AAEL013431 | | 0.1773 | 8.7831 | 0.7137 | 0.9996 | proline oxidase [Source:VB Community Annotation] |
| AAEL014169 | | -0.1343 | 5.0048 | 0.7138 | 0.9996 | small nuclear ribonucleoprotein, core, putative [Source:VB Community Annotation] |
| AAEL009747 | RpS18 | 0.1182 | 12.0376 | 0.7138 | 0.9996 | 40S ribosomal protein S18 [Source:VB Community Annotation] |
| AAEL002222 | | 0.1022 | 3.5740 | 0.7138 | 0.9996 | |
| AAEL016997 | GPRGRP2_ | -0.4468 | -0.7618 | 0.7139 | 0.9996 | GPCR Gastrin/Bombesin Family [Source:VB Community Annotation] |
| AAEL006553 | | 0.1152 | 7.0457 | 0.7140 | 0.9996 | protein disulfide isomerase, putative [Source:VB Community Annotation] |
| AAEL005625 | GPROP5 | -0.3320 | 4.2847 | 0.7142 | 0.9996 | long wavelength sensitive opsin [Source:VB Community Annotation] |
| AAEL008751 | | 0.3623 | -0.1672 | 0.7143 | 0.9996 | glucosyl/glucuronosyl transferases [Source:VB Community Annotation] |
| AAEL003517 | | 0.1356 | 3.4134 | 0.7143 | 0.9996 | Ubiquitinyl hydrolase 1 [Source:UniProtKB/TrEMBL;Acc:A0A1S4F4V1] |
| AAEL014959 | | -0.1122 | 6.3716 | 0.7146 | 0.9996 | heterogeneous nuclear ribonucleoprotein k [Source:VB Community Annotation] |
| AAEL006735 | | -0.1121 | 3.6484 | 0.7146 | 0.9996 | |
| AAEL003917 | | 0.1253 | 4.7708 | 0.7147 | 0.9996 | Mitochondrial inner membrane protease subunit 1 [Source:UniProtKB/TrEMBL;Acc:Q17E53] |
| AAEL003272 | | -0.6232 | -1.3954 | 0.7149 | 0.9996 | pupal cuticle protein 78E, putative [Source:VB Community Annotation] |
| AAEL005496 | | -0.1993 | 2.4587 | 0.7150 | 0.9996 | zinc/iron transporter [Source:VB Community Annotation] |
| AAEL005127 | | 0.1783 | 5.1019 | 0.7150 | 0.9996 | ribonuclease UK114, putative [Source:VB Community Annotation] |
| AAEL013612 | | -0.3239 | 7.8550 | 0.7150 | 0.9996 | |
| AAEL012353 | CTL15 | -0.3389 | 5.4243 | 0.7151 | 0.9996 | C-Type Lectin (CTL). [Source:VB Community Annotation] |
| AAEL008645 | | -0.1889 | 6.0644 | 0.7152 | 0.9996 | |
| AAEL020258 | NA | -0.5206 | -1.3107 | 0.7153 | 0.9996 | NA |
| AAEL005719 | | 0.1081 | 3.7229 | 0.7154 | 0.9996 | cleavage stimulation factor [Source:VB Community Annotation] |

| | | | | | | |
|---|---|---|---|---|---|---|
| AAEL000626 | | -0.3138 | 1.0505 | 0.7154 | 0.9996 | |
| AAEL006472 | | 0.1025 | 5.2390 | 0.7154 | 0.9996 | rabconnectin [Source:VB Community Annotation] |
| AAEL006769 | | -0.1458 | 4.9584 | 0.7155 | 0.9996 | tryptophanyl-tRNA synthetase [Source:VB Community Annotation] |
| AAEL014628 | | 0.2419 | 3.4482 | 0.7155 | 0.9996 | |
| AAEL014092 | | -0.1660 | 3.4344 | 0.7156 | 0.9996 | |
| AAEL017514 | | 1.0665 | 1.2368 | 0.7156 | 0.9996 | |
| AAEL026894 | NA | 0.1860 | 5.0515 | 0.7157 | 0.9996 | NA |
| AAEL012843 | | 0.1407 | 3.3603 | 0.7159 | 0.9996 | Tubulin gamma chain [Source:UniProtKB/TrEMBL;Acc:Q16KA0] |
| AAEL022949 | NA | -0.4349 | -1.3342 | 0.7160 | 0.9996 | NA |
| AAEL001686 | | -0.2515 | 2.1843 | 0.7162 | 0.9996 | |
| AAEL018020 | | 0.1403 | 1.8653 | 0.7163 | 0.9996 | |
| AAEL019811 | NA | -0.5122 | -0.1005 | 0.7164 | 0.9996 | NA |
| AAEL026724 | NA | -0.7179 | 1.5155 | 0.7165 | 0.9996 | NA |
| AAEL023003 | NA | -0.3876 | 8.3600 | 0.7165 | 0.9996 | NA |
| AAEL000759 | | -0.1008 | 6.0846 | 0.7165 | 0.9996 | gamma-glutamylcysteine synthetase, putative [Source:VB Community Annotation] |
| AAEL001458 | | -0.7285 | 0.5092 | 0.7165 | 0.9996 | heparan sulphate sulfotransferase [Source:VB Community Annotation] |
| AAEL005191 | | 0.1589 | 2.0339 | 0.7165 | 0.9996 | cdk10/11 [Source:VB Community Annotation] |
| AAEL019452 | NA | -0.3581 | 6.5149 | 0.7165 | 0.9996 | NA |
| AAEL010805 | | -0.2042 | 1.4868 | 0.7166 | 0.9996 | deformed wings, putative [Source:VB Community Annotation] |
| AAEL011887 | | -0.2345 | 0.4220 | 0.7166 | 0.9996 | zinc finger protein [Source:VB Community Annotation] |
| AAEL008285 | | -0.3089 | 7.1796 | 0.7167 | 0.9996 | pupal cuticle protein, putative [Source:VB Community Annotation] |
| AAEL022364 | NA | 0.4072 | -1.0013 | 0.7167 | 0.9996 | NA |
| AAEL009205 | | -0.6479 | -0.7228 | 0.7171 | 0.9996 | |
| AAEL012424 | | -0.2266 | 4.2267 | 0.7173 | 0.9996 | Tubulin alpha chain [Source:UniProtKB/TrEMBL;Acc:Q16M50] |
| AAEL001877 | | -0.5051 | -1.3985 | 0.7173 | 0.9996 | fucosyltransferase 11 (fut11) [Source:VB Community Annotation] |
| AAEL014960 | | 0.1060 | 6.0369 | 0.7173 | 0.9996 | |
| AAEL008803 | | -0.1049 | 6.0920 | 0.7173 | 0.9996 | nice-3 [Source:VB Community Annotation] |
| AAEL000643 | | 0.1818 | 4.8523 | 0.7174 | 0.9996 | zinc finger protein [Source:VB Community Annotation] |
| AAEL022261 | NA | 0.3270 | 7.0964 | 0.7175 | 0.9996 | NA |
| AAEL026828 | NA | 0.4076 | -2.2360 | 0.7175 | 0.9996 | NA |
| AAEL011580 | | -0.1373 | 3.5805 | 0.7176 | 0.9996 | |
| AAEL025280 | NA | 0.3722 | -0.6044 | 0.7176 | 0.9996 | NA |
| AAEL001876 | | -0.1842 | 4.6133 | 0.7179 | 0.9996 | decapentaplegic, deca [Source:VB Community Annotation] |
| AAEL003560 | | -0.3962 | 3.3018 | 0.7180 | 0.9996 | pou domain/drifter/cf-1a [Source:VB Community Annotation] |
| AAEL020850 | NA | 0.1531 | 3.0214 | 0.7180 | 0.9996 | NA |
| AAEL012036 | | 0.1106 | 5.1946 | 0.7182 | 0.9996 | sulphate transporter [Source:VB Community Annotation] |
| AAEL019936 | NA | 0.3887 | -1.9277 | 0.7183 | 0.9996 | NA |
| AAEL013283 | | 0.4407 | -1.6347 | 0.7183 | 0.9996 | serine-type enodpeptidase [Source:VB Community Annotation] |
| AAEL008956 | | 0.3717 | 1.8290 | 0.7184 | 0.9996 | |
| AAEL023266 | NA | -0.3162 | 8.3086 | 0.7185 | 0.9996 | NA |

| | | | | | | |
|---|---|---|---|---|---|---|
| AAEL000234 | SCRB7 | 0.2191 | 3.8827 | 0.7185 | 0.9996 | Class B Scavenger Receptor (CD36 domain). [Source:VB Community Annotation |
| AAEL010090 | | -0.1226 | 4.2928 | 0.7186 | 0.9996 | |
| AAEL000528 | GPRNNA22 | 0.5291 | -0.6389 | 0.7187 | 0.9996 | GPCR Orphan/Putative Class A Family [Source:VB Community Annotation] |
| AAEL024112 | NA | -0.1678 | 4.6165 | 0.7187 | 0.9996 | NA |
| AAEL008871 | | 0.1736 | 9.5592 | 0.7188 | 0.9996 | succinate dehydrogenase [Source:VB Community Annotation] |
| AAEL026232 | NA | -0.3461 | 6.4702 | 0.7188 | 0.9996 | NA |
| AAEL010141 | | -0.1218 | 4.8846 | 0.7189 | 0.9996 | |
| AAEL004311 | | 0.1115 | 5.0391 | 0.7190 | 0.9996 | |
| AAEL008862 | | 0.0889 | 6.5820 | 0.7190 | 0.9996 | |
| AAEL012091 | | 0.2699 | 2.4343 | 0.7191 | 0.9996 | |
| AAEL009019 | | 0.2002 | 1.0841 | 0.7191 | 0.9996 | |
| AAEL004425 | | 0.1340 | 3.7173 | 0.7191 | 0.9996 | ctg4a [Source:VB Community Annotation] |
| AAEL010230 | | 0.1629 | 8.5947 | 0.7191 | 0.9996 | NADH:ubiquinone dehydrogenase, putative [Source:VB Community Annotation] |
| AAEL006081 | | -0.4062 | -1.4890 | 0.7192 | 0.9996 | NADPH fad oxidoreductase [Source:VB Community Annotation] |
| AAEL002140 | | -0.0771 | 6.7273 | 0.7195 | 0.9996 | transcription initiation factor IIA (TFIIA), gamma chain [Source:VB Community Annotation] |
| AAEL020793 | NA | -0.2301 | 1.2647 | 0.7195 | 0.9996 | NA |
| AAEL006425 | | 0.4759 | 5.0802 | 0.7196 | 0.9996 | trypsin [Source:VB Community Annotation] |
| AAEL012245 | | -0.1487 | 5.5308 | 0.7196 | 0.9996 | |
| AAEL000629 | | 0.2608 | 6.0778 | 0.7196 | 0.9996 | adenylate kinase 3, [Source:VB Community Annotation] |
| AAEL017155 | | -0.2391 | 2.4649 | 0.7198 | 0.9996 | |
| AAEL000682 | | -0.2985 | 4.1964 | 0.7198 | 0.9996 | |
| AAEL010347 | | 0.4737 | 1.3868 | 0.7200 | 0.9996 | |
| AAEL028175 | NA | -0.7893 | 6.2055 | 0.7201 | 0.9996 | NA |
| AAEL004349 | | 0.1452 | 3.9293 | 0.7202 | 0.9996 | tetraspanin, putative [Source:VB Community Annotation] |
| AAEL001906 | | -0.2025 | 6.0690 | 0.7203 | 0.9996 | pnuts protein [Source:VB Community Annotation] |
| AAEL010088 | | 0.1264 | 3.7049 | 0.7205 | 0.9996 | |
| AAEL001564 | | 0.1893 | 6.6719 | 0.7206 | 0.9996 | taz protein (tafazzin) [Source:VB Community Annotation] |
| AAEL019650 | NA | -0.2217 | 8.2786 | 0.7208 | 0.9996 | NA |
| AAEL010732 | | 0.1172 | 4.5431 | 0.7213 | 0.9996 | ATP-dependent RNA helicase [Source:VB Community Annotation] |
| AAEL008745 | | 0.1879 | 2.6192 | 0.7214 | 0.9996 | |
| AAEL017691 | U1 | 0.3979 | 0.4820 | 0.7216 | 0.9996 | U1 spliceosomal RNA [Source:RFAM;Acc:RF00003] |
| AAEL009249 | | -0.1236 | 3.9868 | 0.7219 | 0.9996 | coronin [Source:VB Community Annotation] |
| AAEL025926 | NA | 0.2097 | 1.1542 | 0.7219 | 0.9996 | NA |
| AAEL024868 | NA | 0.3895 | -0.5250 | 0.7222 | 0.9996 | NA |
| AAEL007997 | | 0.1615 | 3.1999 | 0.7222 | 0.9996 | |
| AAEL007946 | GSTE6 | 0.2528 | 4.0245 | 0.7224 | 0.9996 | glutathione transferase [Source:VB Community Annotation] |
| AAEL000250 | | 0.1688 | 5.4159 | 0.7224 | 0.9996 | forkhead box protein (AaegFOXP) [Source:VB Community Annotation] |
| AAEL011370 | | 0.1449 | 2.9169 | 0.7224 | 0.9996 | zinc finger protein [Source:VB Community Annotation] |
| AAEL003949 | | -0.1899 | 4.6541 | 0.7225 | 0.9996 | |
| AAEL001612 | | -0.5330 | 1.0699 | 0.7226 | 0.9996 | dicer-1 [Source:VB Community Annotation] |

| | | | | | | |
|---|---|---|---|---|---|---|
| AAEL016621 | tRNA-Ser | -0.2523 | -0.2146 | 0.7226 | 0.9996 | |
| AAEL000266 | GPRNNA21 | -0.7459 | 2.1222 | 0.7228 | 0.9996 | GPCR Orphan/Putative Class A Family [Source:VB Community Annotation] |
| AAEL022285 | NA | 0.3602 | 11.2945 | 0.7230 | 0.9996 | NA |
| AAEL002693 | | -0.6029 | 7.6395 | 0.7230 | 0.9996 | venom allergen [Source:VB Community Annotation] |
| AAEL000111 | | -0.2811 | 7.2770 | 0.7230 | 0.9996 | nitrilase, putative [Source:VB Community Annotation] |
| AAEL002456 | | -0.2729 | 3.2117 | 0.7231 | 0.9996 | b-cell lymphoma/leukemia [Source:VB Community Annotation] |
| AAEL013940 | | -0.1673 | 1.0951 | 0.7231 | 0.9996 | chromatin assembly factor i P60 subunit [Source:VB Community Annotation] |
| AAEL025969 | NA | -0.1015 | 4.1886 | 0.7232 | 0.9996 | NA |
| AAEL005837 | | -0.0807 | 4.3396 | 0.7232 | 0.9996 | neutral sphingomyelinase (n-smase) activation associated factor fan [Source:VB Community Annotation] |
| AAEL009846 | | -0.1688 | 3.4207 | 0.7233 | 0.9996 | Enn protein, putative [Source:VB Community Annotation] |
| AAEL010303 | | 0.1232 | 3.4685 | 0.7233 | 0.9996 | |
| AAEL006423 | | -0.6933 | 8.9213 | 0.7234 | 0.9996 | |
| AAEL025332 | NA | -0.3568 | 1.0652 | 0.7234 | 0.9996 | NA |
| AAEL011001 | | 0.2861 | 0.8845 | 0.7234 | 0.9996 | |
| AAEL012765 | CYP325M3 | -0.4607 | 0.2718 | 0.7235 | 0.9996 | cytochrome P450 [Source:VB Community Annotation] |
| AAEL004233 | | -0.5229 | 0.4620 | 0.7236 | 0.9996 | |
| AAEL000720 | Med11 | -0.0849 | 3.6455 | 0.7236 | 0.9996 | mediator of RNA polymerase II transcription subunit 11 (Med11) [Source:VB Community Annotation] |
| AAEL003426 | | 0.4942 | -0.4039 | 0.7236 | 0.9996 | sodium-dependent phosphate transporter [Source:VB Community Annotation] |
| AAEL004291 | | -0.2406 | 2.7846 | 0.7237 | 0.9996 | translin [Source:VB Community Annotation] |
| AAEL006458 | | 0.2269 | 5.2189 | 0.7239 | 0.9996 | alcohol dehydrogenase [Source:VB Community Annotation] |
| AAEL013851 | | 0.2976 | 3.0335 | 0.7240 | 0.9996 | |
| AAEL001228 | | -0.4468 | 2.1006 | 0.7241 | 0.9996 | myelin transcription factor 1, myt1 [Source:VB Community Annotation] |
| AAEL024437 | NA | 0.5645 | 5.8625 | 0.7241 | 0.9996 | NA |
| AAEL019951 | NA | -0.5499 | 5.1561 | 0.7241 | 0.9996 | NA |
| AAEL012832 | | 0.2165 | 3.4935 | 0.7242 | 0.9996 | cytochrome B561 [Source:VB Community Annotation] |
| AAEL001437 | | -0.5420 | 2.6496 | 0.7244 | 0.9996 | |
| AAEL009059 | | 0.1053 | 6.1852 | 0.7245 | 0.9996 | arp2/3 complex 16 kd subunit (P16-arc) [Source:VB Community Annotation] |
| AAEL027832 | NA | -0.6247 | 0.9364 | 0.7245 | 0.9996 | NA |
| AAEL000210 | mRpL2 | 0.1380 | 5.2844 | 0.7245 | 0.9996 | mitochondrial ribosomal protein L2 [Source:VB Community Annotation] |
| AAEL024053 | NA | 0.1015 | 5.8837 | 0.7246 | 0.9996 | NA |
| AAEL007690 | | -0.1381 | 3.1994 | 0.7247 | 0.9996 | |
| AAEL003111 | | -0.2713 | 2.4415 | 0.7248 | 0.9996 | tartan [Source:VB Community Annotation] |
| AAEL017366 | | 0.1415 | 4.7726 | 0.7248 | 0.9996 | |
| AAEL001425 | | 0.1133 | 3.6638 | 0.7248 | 0.9996 | |
| AAEL019672 | NA | 0.1330 | 4.0593 | 0.7248 | 0.9996 | NA |
| AAEL019720 | NA | -0.1972 | 7.8611 | 0.7250 | 0.9996 | NA |
| AAEL019797 | NA | -0.2793 | 7.3462 | 0.7251 | 0.9996 | NA |
| AAEL001334 | | 0.0957 | 4.9219 | 0.7252 | 0.9996 | geranylgeranyl transferase type ii beta subunit [Source:VB Community Annotation] |
| AAEL014440 | | -0.3311 | -0.6912 | 0.7252 | 0.9996 | juvenile hormone-inducible protein, putative [Source:VB Community Annotation] |
| AAEL002720 | SRPN20 | -0.2648 | 7.4356 | 0.7254 | 0.9996 | Serine Protease Inhibitor (serpin)  likely cleavage at V/V. [Source:VB Community Annotation] |

| | | | | | | |
|---|---|---|---|---|---|---|
| AAEL028110 | NA | 0.1172 | 3.8634 | 0.7254 | 0.9996 | NA |
| AAEL001434 | | 0.1404 | 4.9347 | 0.7255 | 0.9996 | Coronin (Fragment) [Source:UniProtKB/TrEMBL;Acc:Q17L99] |
| AAEL007878 | | -0.2265 | 0.5555 | 0.7256 | 0.9996 | ornithine decarboxylase [Source:VB Community Annotation] |
| AAEL001798 | | 0.0960 | 8.4906 | 0.7256 | 0.9996 | protein tyrosine phosphatase prl [Source:VB Community Annotation] |
| AAEL010290 | | -0.1726 | 6.3907 | 0.7257 | 0.9996 | short-chain dehydrogenase [Source:VB Community Annotation] |
| AAEL026227 | NA | 0.1160 | 3.3563 | 0.7258 | 0.9996 | NA |
| AAEL007038 | | -0.1882 | 3.9920 | 0.7258 | 0.9996 | prolyl 4-hydroxylase alpha subunit 1 [Source:VB Community Annotation] |
| AAEL019999 | NA | 0.4226 | 0.2230 | 0.7259 | 0.9996 | NA |
| AAEL002644 | | -0.1127 | 5.2327 | 0.7259 | 0.9996 | |
| AAEL005633 | | -0.8092 | 2.6915 | 0.7259 | 0.9996 | |
| AAEL024616 | NA | 0.0673 | 4.2138 | 0.7260 | 0.9996 | NA |
| AAEL003457 | | -0.3481 | 5.9361 | 0.7260 | 0.9996 | |
| AAEL011314 | | 0.1444 | 6.1257 | 0.7261 | 0.9996 | Epoxide hydrolase [Source:UniProtKB/TrEMBL;Acc:Q16QD6] |
| AAEL011395 | | -0.0870 | 5.2995 | 0.7261 | 0.9996 | glutaminyl-tRNA synthetase [Source:VB Community Annotation] |
| AAEL013492 | PPO5 | 0.4961 | 5.1539 | 0.7262 | 0.9996 | prophenoloxidase [Source:VB Community Annotation] |
| AAEL015061 | | -0.0837 | 6.1039 | 0.7263 | 0.9996 | chaperone binding protein [Source:VB Community Annotation] |
| AAEL010814 | | 0.1417 | 7.4693 | 0.7263 | 0.9996 | isocitrate dehydrogenase [Source:VB Community Annotation] |
| AAEL022319 | NA | 0.3780 | 0.7398 | 0.7263 | 0.9996 | NA |
| AAEL000101 | | 0.3158 | 2.0562 | 0.7263 | 0.9996 | AMP dependent coa ligase [Source:VB Community Annotation] |
| AAEL024070 | NA | -0.1049 | 6.2316 | 0.7268 | 0.9996 | NA |
| AAEL002886 | | 0.1095 | 8.1178 | 0.7268 | 0.9996 | thioredoxin reductase [Source:VB Community Annotation] |
| AAEL007149 | | 0.6126 | -0.9658 | 0.7269 | 0.9996 | |
| AAEL011685 | | 0.2192 | 1.3693 | 0.7271 | 0.9996 | |
| AAEL025553 | NA | 0.1102 | 10.6974 | 0.7271 | 0.9996 | NA |
| AAEL022091 | NA | 0.1118 | 3.6781 | 0.7271 | 0.9996 | NA |
| AAEL025144 | NA | -0.6574 | -2.3950 | 0.7273 | 0.9996 | NA |
| AAEL011097 | | 0.0965 | 6.1985 | 0.7274 | 0.9996 | |
| AAEL011584 | | -0.1580 | 4.7388 | 0.7275 | 0.9996 | chaperonin-60kD, ch60 [Source:VB Community Annotation] |
| AAEL005269 | | 0.1511 | 9.5570 | 0.7276 | 0.9996 | ubiquinol-cytochrome c reductase complex core protein [Source:VB Community Annotation] |
| AAEL026790 | NA | 0.4201 | -0.7494 | 0.7276 | 0.9996 | NA |
| AAEL020075 | NA | -0.1387 | 7.2935 | 0.7276 | 0.9996 | NA |
| AAEL005674 | | -0.0901 | 4.9086 | 0.7276 | 0.9996 | |
| AAEL026147 | NA | 0.6687 | 2.6480 | 0.7277 | 0.9996 | NA |
| AAEL017095 | | -0.6220 | -0.2319 | 0.7278 | 0.9996 | |
| AAEL000334 | | 0.3184 | 0.1563 | 0.7279 | 0.9996 | villin [Source:VB Community Annotation] |
| AAEL014411 | CYP304B3 | 0.1986 | 8.0392 | 0.7279 | 0.9996 | cytochrome P450 [Source:VB Community Annotation] |
| AAEL021817 | NA | -0.8322 | 0.3124 | 0.7280 | 0.9996 | NA |
| AAEL002080 | | 0.1837 | 2.3107 | 0.7281 | 0.9996 | septin interacting protein, putative [Source:VB Community Annotation] |
| AAEL007821 | | -0.1081 | 6.3123 | 0.7283 | 0.9996 | signalosome, subunit 2, CSN8, putative [Source:VB Community Annotation] |
| AAEL011651 | | -0.1570 | 3.9342 | 0.7285 | 0.9996 | l-caldesmon, putative [Source:VB Community Annotation] |

| | | | | | | |
|---|---|---|---|---|---|---|
| AAEL006120 | | -0.5626 | 3.4572 | 0.7287 | 0.9996 | |
| AAEL000115 | | 0.1404 | 3.2138 | 0.7287 | 0.9996 | |
| AAEL020106 | NA | -0.1636 | 5.2292 | 0.7288 | 0.9996 | NA |
| AAEL018288 | | -0.2460 | 6.7630 | 0.7288 | 0.9996 | |
| AAEL011599 | | 0.2955 | 1.4003 | 0.7288 | 0.9996 | ADP-ribosylation factor, putative [Source:VB Community Annotation] |
| AAEL007977 | | 0.1884 | 2.6318 | 0.7289 | 0.9996 | |
| AAEL001355 | | -0.1212 | 5.9868 | 0.7290 | 0.9996 | |
| AAEL003992 | | 0.1458 | 3.7034 | 0.7290 | 0.9996 | |
| AAEL013032 | | -0.2966 | 5.5148 | 0.7291 | 0.9996 | |
| AAEL009081 | | 0.5987 | 11.9063 | 0.7291 | 0.9996 | |
| AAEL020638 | NA | -0.1307 | 4.2913 | 0.7292 | 0.9996 | NA |
| AAEL005510 | | -0.3022 | 2.0758 | 0.7296 | 0.9996 | WD-repeat protein [Source:VB Community Annotation] |
| AAEL024228 | NA | 0.4210 | 3.4052 | 0.7296 | 0.9996 | NA |
| AAEL025094 | NA | 0.2254 | 2.3482 | 0.7297 | 0.9996 | NA |
| AAEL003143 | | -0.1001 | 4.2754 | 0.7297 | 0.9996 | inositol polyphosphate 5-phosphatase [Source:VB Community Annotation] |
| AAEL008730 | | -0.2934 | 0.2431 | 0.7299 | 0.9996 | anillin/rhotekin (rtkn) [Source:VB Community Annotation] |
| AAEL013790 | mRpL50 | 0.1419 | 5.0215 | 0.7299 | 0.9996 | mitochondrial ribosomal protein, L50 [Source:VB Community Annotation] |
| AAEL000287 | | 0.1620 | 1.4467 | 0.7299 | 0.9996 | |
| AAEL007227 | | 0.1542 | 2.6186 | 0.7300 | 0.9996 | |
| AAEL005431 | CLIPB37 | -0.3357 | 5.4317 | 0.7301 | 0.9996 | Clip-Domain Serine Protease  family B. [Source:VB Community Annotation] |
| AAEL006937 | | -0.4749 | -0.7013 | 0.7301 | 0.9996 | structural maintenance of chromosomes 3 smc3 [Source:VB Community Annotation] |
| AAEL012152 | | 0.0792 | 6.0302 | 0.7302 | 0.9996 | activin receptor type I, putative [Source:VB Community Annotation] |
| AAEL007286 | Manf | 0.0755 | 5.4633 | 0.7302 | 0.9996 | arginine-rich protein, putative [Source:VB Community Annotation] |
| AAEL004534 | | -0.1276 | 6.0112 | 0.7302 | 0.9996 | |
| AAEL008355 | | 0.2792 | 1.8988 | 0.7303 | 0.9996 | |
| AAEL000461 | | 0.0958 | 5.6432 | 0.7304 | 0.9996 | |
| AAEL010154 | CYP4AR2 | 0.3773 | -0.1043 | 0.7304 | 0.9996 | cytochrome P450 [Source:VB Community Annotation] |
| AAEL014256 | | 0.1338 | 3.4847 | 0.7305 | 0.9996 | ATP-dependent RNA helicase [Source:VB Community Annotation] |
| AAEL008753 | | -0.1230 | 7.9638 | 0.7307 | 0.9996 | |
| AAEL013265 | | -0.1156 | 4.7565 | 0.7308 | 0.9996 | |
| AAEL009140 | | 0.1070 | 3.6160 | 0.7312 | 0.9996 | |
| AAEL014094 | | 0.1378 | 2.9830 | 0.7315 | 0.9996 | solute carrier family 35 member C2, putative [Source:VB Community Annotation] |
| AAEL009169 | | 0.0767 | 6.2085 | 0.7315 | 0.9996 | synaptotagmin, putative [Source:VB Community Annotation] |
| AAEL020494 | NA | 0.4541 | -0.2729 | 0.7315 | 0.9996 | NA |
| AAEL013507 | Or52 | -0.2950 | 0.2979 | 0.7316 | 0.9996 | odorant receptor (Or52) [Source:VB Community Annotation] |
| AAEL023599 | NA | -0.5592 | -2.1729 | 0.7317 | 0.9996 | NA |
| AAEL007198 | | 0.4076 | -2.1335 | 0.7317 | 0.9996 | Osiris, putative [Source:VB Community Annotation] |
| AAEL024011 | NA | 0.2880 | -0.8614 | 0.7321 | 0.9996 | NA |
| AAEL014961 | | 0.2471 | 0.6564 | 0.7321 | 0.9996 | gdp mannose-4,6-dehydratase [Source:VB Community Annotation] |
| AAEL014797 | | 0.1309 | 4.9899 | 0.7321 | 0.9996 | acyl-coa dehydrogenase [Source:VB Community Annotation] |

| | | | | | | |
|---|---|---|---|---|---|---|
| AAEL007685 | | 0.1044 | 6.4305 | 0.7324 | 0.9996 | |
| AAEL007525 | | -0.2426 | 3.4096 | 0.7325 | 0.9996 | rab 19 [Source:VB Community Annotation] |
| AAEL021921 | NA | 0.4431 | -0.7587 | 0.7325 | 0.9996 | NA |
| AAEL024890 | NA | -0.2176 | 0.4225 | 0.7326 | 0.9996 | NA |
| AAEL019416 | NA | -0.3637 | 5.2690 | 0.7326 | 0.9996 | NA |
| AAEL000944 | | -0.1656 | 4.9973 | 0.7326 | 0.9996 | |
| AAEL006447 | | -0.3170 | 3.8740 | 0.7326 | 0.9996 | GATA transcription factor (GATAb) [Source:VB Community Annotation] |
| AAEL025922 | NA | 0.1076 | 5.2127 | 0.7327 | 0.9996 | NA |
| AAEL010294 | | -0.1856 | 4.2063 | 0.7327 | 0.9996 | membrane-associated guanylate kinase (maguk) [Source:VB Community Annotation] |
| AAEL002453 | | 0.1274 | 3.3950 | 0.7327 | 0.9996 | |
| AAEL027482 | NA | 0.1804 | 2.2345 | 0.7327 | 0.9996 | NA |
| AAEL011068 | | 0.5906 | 4.6806 | 0.7328 | 0.9996 | |
| AAEL002130 | | -0.1746 | 5.3436 | 0.7328 | 0.9996 | ecdysone inducible protein L2, putative [Source:VB Community Annotation] |
| AAEL022196 | NA | -0.1832 | 4.6071 | 0.7329 | 0.9996 | NA |
| AAEL017156 | | 0.1288 | 3.4482 | 0.7329 | 0.9996 | DNA-directed RNA polymerase subunit beta [Source:UniProtKB/TrEMBL;Acc:J9E9F1] |
| AAEL017201 | Or94 | -0.2788 | -0.4870 | 0.7329 | 0.9996 | Odorant receptor [Source:UniProtKB/TrEMBL;Acc:J9E9Q3] |
| AAEL011092 | | 0.1675 | 4.3458 | 0.7330 | 0.9996 | |
| AAEL027032 | NA | 0.2854 | -0.2457 | 0.7331 | 0.9996 | NA |
| AAEL012761 | CYP325T2 | -0.1808 | 4.1481 | 0.7331 | 0.9996 | cytochrome P450 [Source:VB Community Annotation] |
| AAEL001209 | | -0.1445 | 5.3370 | 0.7331 | 0.9996 | sodium-dependent phosphate transporter [Source:VB Community Annotation] |
| AAEL021095 | NA | 0.2311 | 3.1889 | 0.7332 | 0.9996 | NA |
| AAEL007194 | | 0.4177 | 3.5979 | 0.7332 | 0.9996 | pupal cuticle protein, putative [Source:VB Community Annotation] |
| AAEL007366 | | -0.0946 | 5.2948 | 0.7333 | 0.9996 | |
| AAEL027367 | NA | 0.1054 | 4.3761 | 0.7333 | 0.9996 | NA |
| AAEL000671 | TOLL6 | -0.7046 | 0.2254 | 0.7334 | 0.9996 | Toll-like receptor (TOLL6) [Source:VB Community Annotation] |
| AAEL013296 | | -0.1651 | 1.2810 | 0.7334 | 0.9996 | |
| AAEL021741 | NA | 0.0994 | 4.1553 | 0.7334 | 0.9996 | NA |
| AAEL024522 | NA | -0.4400 | -1.6140 | 0.7335 | 0.9996 | NA |
| AAEL026382 | NA | 0.0914 | 4.7913 | 0.7336 | 0.9996 | NA |
| AAEL008330 | | 0.2677 | 5.6406 | 0.7336 | 0.9996 | hexaprenyldihydroxybenzoate methyltransferase [Source:VB Community Annotation] |
| AAEL023506 | NA | -0.1168 | 4.4376 | 0.7337 | 0.9996 | NA |
| AAEL006415 | | 0.1075 | 5.3125 | 0.7338 | 0.9996 | leucyl-tRNA synthetase [Source:VB Community Annotation] |
| AAEL004191 | | 0.2028 | 1.6935 | 0.7338 | 0.9996 | selenocysteine-specific elongation factor [Source:VB Community Annotation] |
| AAEL001098 | | -0.2733 | 7.9442 | 0.7339 | 0.9996 | clip-domain serine protease, putative [Source:VB Community Annotation] |
| AAEL024598 | NA | 0.1259 | 7.9257 | 0.7339 | 0.9996 | NA |
| AAEL004502 | | -0.1880 | 5.8815 | 0.7339 | 0.9996 | Rhomboid-like protein [Source:UniProtKB/TrEMBL;Acc:Q0IFM3] |
| AAEL023147 | NA | -0.1577 | 2.1097 | 0.7341 | 0.9996 | NA |
| AAEL013304 | | 0.1605 | 5.4287 | 0.7342 | 0.9996 | |
| AAEL014024 | | 0.0891 | 5.1480 | 0.7342 | 0.9996 | |
| AAEL002837 | | -0.1126 | 3.9161 | 0.7342 | 0.9996 | radical sam proteins [Source:VB Community Annotation] |

| | | | | | | |
|---|---|---|---|---|---|---|
| AAEL006717 | | 0.1189 | 3.9808 | 0.7343 | 0.9996 | ABC transporter [Source:VB Community Annotation] |
| AAEL024210 | NA | -0.1000 | 4.6574 | 0.7343 | 0.9996 | NA |
| AAEL012941 | | 0.2311 | 4.3800 | 0.7344 | 0.9996 | |
| AAEL021621 | NA | 0.3152 | 1.7155 | 0.7344 | 0.9996 | NA |
| AAEL008166 | | -0.1301 | 8.6047 | 0.7345 | 0.9996 | malate dehydrogenase [Source:VB Community Annotation] |
| AAEL027092 | NA | 0.2204 | 1.6680 | 0.7346 | 0.9996 | NA |
| AAEL011206 | | 0.1565 | 6.5572 | 0.7348 | 0.9996 | aminoacylase, putative [Source:VB Community Annotation] |
| AAEL026109 | NA | 0.1114 | 6.0488 | 0.7348 | 0.9996 | NA |
| AAEL004940 | | -0.1453 | 2.5647 | 0.7348 | 0.9996 | zinc finger protein [Source:VB Community Annotation] |
| AAEL023675 | NA | 0.0804 | 5.4516 | 0.7349 | 0.9996 | NA |
| AAEL007307 | | -0.1686 | 3.3307 | 0.7349 | 0.9996 | |
| AAEL013052 | | 0.1288 | 8.5450 | 0.7350 | 0.9996 | ubiquitin carboxyl-terminal hydrolase isozyme L3 [Source:VB Community Annotation] |
| AAEL001693 | | 0.5794 | 0.8748 | 0.7351 | 0.9996 | serine-type enodpeptidase [Source:VB Community Annotation] |
| AAEL011827 | | -0.1131 | 2.7014 | 0.7351 | 0.9996 | |
| AAEL015064 | | 0.0907 | 8.8854 | 0.7351 | 0.9996 | Small ubiquitin-related modifier [Source:UniProtKB/TrEMBL;Acc:Q16EQ3] |
| AAEL022177 | NA | 0.1798 | 4.0784 | 0.7352 | 0.9996 | NA |
| AAEL006863 | | 0.1582 | 4.2276 | 0.7352 | 0.9996 | |
| AAEL019433 | NA | -0.4618 | -1.2501 | 0.7355 | 0.9996 | NA |
| AAEL024653 | NA | -0.3370 | 3.3326 | 0.7355 | 0.9996 | NA |
| AAEL004430 | mRpL30 | -0.1530 | 6.0437 | 0.7356 | 0.9996 | mitochondrial ribosomal protein, L30, putative [Source:VB Community Annotation] |
| AAEL007771 | RpL22 | -0.0734 | 11.1336 | 0.7356 | 0.9996 | 60S ribosomal protein L22 [Source:UniProtKB/TrEMBL;Acc:Q1HRP2] |
| AAEL002118 | | -0.1999 | 5.1635 | 0.7357 | 0.9996 | ubiquitin-conjugating enzyme rad6 [Source:VB Community Annotation] |
| AAEL026128 | NA | 0.2477 | 0.5536 | 0.7357 | 0.9996 | NA |
| AAEL013544 | | -0.5413 | -0.2558 | 0.7358 | 0.9996 | snail protein, putative [Source:VB Community Annotation] |
| AAEL021272 | NA | -0.3862 | -1.0457 | 0.7358 | 0.9996 | NA |
| AAEL009894 | LRIM21 | 0.0897 | 4.2373 | 0.7361 | 0.9996 | leucine-rich immune protein (Coil-less) [Source:VB Community Annotation] |
| AAEL004105 | | -0.6243 | 0.6507 | 0.7361 | 0.9996 | neuroglobin, putative [Source:VB Community Annotation] |
| AAEL011073 | Gr19 | -0.3366 | 0.1407 | 0.7362 | 0.9996 | gustatory receptor Gr19 [Source:VB Community Annotation] |
| AAEL027654 | NA | 0.1543 | 7.1663 | 0.7362 | 0.9996 | NA |
| AAEL020977 | NA | -0.6257 | -2.1086 | 0.7363 | 0.9996 | NA |
| AAEL027050 | NA | 0.1032 | 5.3340 | 0.7363 | 0.9996 | NA |
| AAEL000261 | | 0.1697 | 2.0643 | 0.7363 | 0.9996 | |
| AAEL005285 | | -0.1092 | 5.4409 | 0.7363 | 0.9996 | |
| AAEL009271 | | -0.4387 | -1.1717 | 0.7364 | 0.9996 | testin [Source:VB Community Annotation] |
| AAEL001403 | | -0.1239 | 4.1158 | 0.7365 | 0.9996 | |
| AAEL003112 | | 0.2723 | 3.4743 | 0.7366 | 0.9996 | |
| AAEL023279 | NA | -0.2231 | 2.3350 | 0.7366 | 0.9996 | NA |
| AAEL004022 | | -0.2680 | 4.5787 | 0.7367 | 0.9996 | |
| AAEL010160 | | -0.6258 | 0.6718 | 0.7368 | 0.9996 | |
| AAEL004031 | | -0.3920 | -1.0520 | 0.7368 | 0.9996 | |

| | | | | | | |
|---|---|---|---|---|---|---|
| AAEL019967 | NA | 0.1198 | 3.1374 | 0.7369 | 0.9996 | NA |
| AAEL006829 | | 0.1561 | 9.2985 | 0.7370 | 0.9996 | microsomal glutathione s-transferase [Source:VB Community Annotation] |
| AAEL006355 | SCRC1 | -0.1999 | 3.0797 | 0.7370 | 0.9996 | Class C Scavenger Receptor (Sushi/SCR/CCP MAM and Somatomedin B domains). [Source:VB Community Annotation] |
| AAEL005677 | | 0.3749 | 1.0629 | 0.7371 | 0.9996 | |
| AAEL022900 | NA | 0.3445 | -1.4595 | 0.7372 | 0.9996 | NA |
| AAEL002195 | | 0.2338 | 0.3743 | 0.7372 | 0.9996 | |
| AAEL028228 | NA | -0.2148 | 3.1439 | 0.7373 | 0.9996 | NA |
| AAEL019970 | NA | -0.4197 | 5.3092 | 0.7373 | 0.9996 | NA |
| AAEL021788 | NA | -0.4801 | -2.5019 | 0.7373 | 0.9996 | NA |
| AAEL013452 | | 0.2436 | 3.6453 | 0.7374 | 0.9996 | |
| AAEL003464 | | 0.1085 | 4.2494 | 0.7374 | 0.9996 | |
| AAEL012171 | | 0.0808 | 5.2387 | 0.7376 | 0.9996 | RNA recognition motif protein split ends [Source:VB Community Annotation] |
| AAEL026710 | NA | -0.7255 | -0.1912 | 0.7376 | 0.9996 | NA |
| AAEL023240 | NA | -0.1330 | 3.6541 | 0.7376 | 0.9996 | NA |
| AAEL019658 | NA | -0.2344 | 4.9407 | 0.7378 | 0.9996 | NA |
| AAEL010821 | RpLP0 | -0.1026 | 10.6088 | 0.7378 | 0.9996 | 60S ribosomal protein LP0 [Source:VB Community Annotation] |
| AAEL001293 | | -0.2988 | 4.6571 | 0.7379 | 0.9996 | |
| AAEL005921 | | -0.1490 | 3.7162 | 0.7380 | 0.9996 | D-lactate dehydrognease 2, [Source:VB Community Annotation] |
| AAEL006070 | | 0.1444 | 8.2710 | 0.7381 | 0.9996 | Phosphoglycerate mutase [Source:UniProtKB/TrEMBL;Acc:Q177P3] |
| AAEL019953 | NA | 0.4071 | -1.3725 | 0.7381 | 0.9996 | NA |
| AAEL003259 | | -0.2993 | 10.0301 | 0.7383 | 0.9996 | pupal cuticle protein 78E, putative [Source:VB Community Annotation] |
| AAEL023007 | NA | -0.1088 | 5.5046 | 0.7384 | 0.9996 | NA |
| AAEL024318 | NA | 0.2278 | 0.0218 | 0.7384 | 0.9996 | NA |
| AAEL010698 | | 0.5814 | 0.6590 | 0.7384 | 0.9996 | importin beta-4 [Source:VB Community Annotation] |
| AAEL007040 | | -0.1643 | 1.7359 | 0.7384 | 0.9996 | lozenge [Source:VB Community Annotation] |
| AAEL026002 | NA | 0.3004 | 0.7019 | 0.7386 | 0.9996 | NA |
| AAEL020513 | NA | -0.2459 | 4.2021 | 0.7386 | 0.9996 | NA |
| AAEL003388 | | -0.5448 | -1.2034 | 0.7387 | 0.9996 | axis inhibition protein, axin [Source:VB Community Annotation] |
| AAEL017302 | SDR-1 | 0.1526 | 8.1842 | 0.7387 | 0.9996 | Farnesol dehydrogenase [Source:UniProtKB/Swiss-Prot;Acc:D2WKD9] |
| AAEL007857 | | -0.1387 | 3.4324 | 0.7389 | 0.9996 | |
| AAEL011803 | | -0.1024 | 6.5124 | 0.7390 | 0.9996 | prohibitin, putative [Source:VB Community Annotation] |
| AAEL002176 | | 0.1799 | 3.3873 | 0.7390 | 0.9996 | inosine-uridine preferring nucleoside hydrolase [Source:VB Community Annotation] |
| AAEL012010 | | -0.1146 | 8.5504 | 0.7390 | 0.9996 | |
| AAEL017144 | | -0.3367 | 10.6365 | 0.7392 | 0.9996 | |
| AAEL001218 | Aats-ala | -0.1252 | 5.1179 | 0.7392 | 0.9996 | alanyl-tRNA synthetase [Source:VB Community Annotation] |
| AAEL009072 | | -0.2504 | 7.6658 | 0.7392 | 0.9996 | ribonucleoprotein [Source:VB Community Annotation] |
| AAEL011111 | | -0.2081 | 3.1837 | 0.7393 | 0.9996 | |
| AAEL004933 | | -0.4801 | -2.1488 | 0.7394 | 0.9996 | lysosomal acid lipase, putative [Source:VB Community Annotation] |
| AAEL011815 | | 0.1141 | 4.4985 | 0.7395 | 0.9996 | Poly [ADP-ribose] polymerase [Source:UniProtKB/TrEMBL;Acc:Q16NZ4] |
| AAEL011362 | | -0.1546 | 2.7445 | 0.7395 | 0.9996 | |

| | | | | | | |
|---|---|---|---|---|---|---|
| AAEL019428 | NA | -0.6512 | 4.1461 | 0.7395 | 0.9996 | NA |
| AAEL004914 | | 0.1164 | 6.4539 | 0.7396 | 0.9996 | |
| AAEL018329 | | -0.2158 | 8.1560 | 0.7398 | 0.9996 | |
| AAEL001324 | | -0.0714 | 4.7343 | 0.7398 | 0.9996 | replication factor C large subunit, putative [Source:VB Community Annotation] |
| AAEL012731 | Adk2 | 0.1068 | 5.6184 | 0.7398 | 0.9996 | Adenylate kinase 2, mitochondrial (AK 2)(EC 2.7.4.3)(ATP-AMP transphosphorylase 2) [Source:VB Community Annotation] |
| AAEL022632 | NA | -0.3328 | 7.9144 | 0.7401 | 0.9996 | NA |
| AAEL003648 | | 0.1985 | 0.8342 | 0.7402 | 0.9996 | |
| AAEL008653 | | 0.0833 | 5.2028 | 0.7402 | 0.9996 | engulfment and cell motility protein [Source:VB Community Annotation] |
| AAEL002816 | | 0.1081 | 5.0214 | 0.7403 | 0.9996 | |
| AAEL014886 | | -0.0998 | 8.6048 | 0.7403 | 0.9996 | 4-aminobutyrate aminotransferase [Source:VB Community Annotation] |
| AAEL019717 | NA | -0.1102 | 10.1901 | 0.7403 | 0.9996 | NA |
| AAEL000227 | SCRB8 | 0.1528 | 5.6146 | 0.7406 | 0.9996 | Class B Scavenger Receptor (CD36 domain). [Source:VB Community Annotation |
| AAEL007312 | | -0.1569 | 1.2896 | 0.7406 | 0.9996 | |
| AAEL004862 | | 0.1531 | 4.2389 | 0.7406 | 0.9996 | |
| AAEL009671 | | 0.0928 | 5.0351 | 0.7406 | 0.9996 | snrnp sm protein [Source:VB Community Annotation] |
| AAEL001473 | | -0.0830 | 5.5342 | 0.7407 | 0.9996 | dynamin-associated protein [Source:VB Community Annotation] |
| AAEL020761 | NA | -0.0941 | 3.9472 | 0.7407 | 0.9996 | NA |
| AAEL028048 | NA | 0.3919 | 1.2758 | 0.7408 | 0.9996 | NA |
| AAEL019611 | NA | -0.0945 | 5.3653 | 0.7409 | 0.9996 | NA |
| AAEL003754 | | -0.6034 | 0.4640 | 0.7410 | 0.9996 | actin binding [Source:VB Community Annotation] |
| AAEL004726 | | -0.1658 | 5.1458 | 0.7411 | 0.9996 | |
| AAEL000382 | | 0.1647 | 2.2721 | 0.7412 | 0.9996 | myosin motor, putative [Source:VB Community Annotation] |
| AAEL000124 | | -0.1514 | 7.4697 | 0.7412 | 0.9996 | |
| AAEL019863 | NA | -0.3463 | 5.0439 | 0.7414 | 0.9996 | NA |
| AAEL011635 | | 0.1860 | 2.8990 | 0.7415 | 0.9996 | |
| AAEL014594 | CYP301A1 | 0.2836 | -2.1987 | 0.7416 | 0.9996 | cytochrome P450 [Source:VB Community Annotation] |
| AAEL021713 | NA | 0.3856 | -0.6323 | 0.7416 | 0.9996 | NA |
| AAEL013894 | | 0.1358 | 5.7364 | 0.7417 | 0.9996 | |
| AAEL011240 | | 0.0814 | 4.6991 | 0.7417 | 0.9996 | |
| AAEL005458 | whd | 0.1068 | 6.9603 | 0.7418 | 0.9996 | protein withered, carnitine O-palmitoyltransferase [Source:VB Community Annotation] |
| AAEL003803 | | -0.2944 | 4.7133 | 0.7418 | 0.9996 | |
| AAEL010970 | | -0.2200 | 3.3439 | 0.7419 | 0.9996 | |
| AAEL003605 | | 0.1139 | 5.1886 | 0.7419 | 0.9996 | U2 small nuclear ribonucleoprotein, putative [Source:VB Community Annotation] |
| AAEL019518 | NA | -0.5719 | 1.4178 | 0.7421 | 0.9996 | NA |
| AAEL003418 | | 0.1566 | 8.3484 | 0.7421 | 0.9996 | microsomal signal peptidase 25 kda subunit [Source:VB Community Annotation] |
| AAEL000016 | | -0.0821 | 5.6768 | 0.7421 | 0.9996 | Esterase AAEL000016 [Source:UniProtKB/Swiss-Prot;Acc:Q0C7C4] |
| AAEL022240 | NA | -0.1275 | 4.9565 | 0.7423 | 0.9996 | NA |
| AAEL013390 | | -0.1120 | 2.9939 | 0.7423 | 0.9996 | serine/threonine protein kinase [Source:VB Community Annotation] |
| AAEL019752 | NA | -0.2817 | 5.5009 | 0.7424 | 0.9996 | NA |
| AAEL012987 | | 0.3338 | 3.7311 | 0.7424 | 0.9996 | |

| | | | | | | |
|---|---|---|---|---|---|---|
| AAEL009382 | | -0.1356 | 3.3085 | 0.7425 | 0.9996 | lysine-specific demethylase NO66 (EC 1.14.11.27)(Nucleolar protein 66) [Source:VB Community Annotation] |
| AAEL018139 | | -0.6336 | -0.7741 | 0.7425 | 0.9996 | |
| AAEL008398 | | -0.1190 | 6.2298 | 0.7426 | 0.9996 | |
| AAEL026161 | NA | 0.2588 | 6.4622 | 0.7427 | 0.9996 | NA |
| AAEL003656 | | -0.2231 | 5.3771 | 0.7427 | 0.9996 | |
| AAEL006587 | | 0.1062 | 3.9702 | 0.7428 | 0.9996 | |
| AAEL012212 | | -0.4626 | 0.2631 | 0.7428 | 0.9996 | |
| AAEL000642 | | -0.1236 | 5.1928 | 0.7430 | 0.9996 | alpha-amylase [Source:VB Community Annotation] |
| AAEL020456 | NA | -0.5489 | 2.1475 | 0.7430 | 0.9996 | NA |
| AAEL009150 | | -0.1258 | 6.4179 | 0.7430 | 0.9996 | |
| AAEL004466 | LRIM23 | -0.2398 | 3.4340 | 0.7432 | 0.9996 | leucine-rich immune protein (Coil-less) [Source:VB Community Annotation] |
| AAEL017400 | | 0.1204 | 3.5223 | 0.7433 | 0.9996 | |
| AAEL008593 | | 0.1059 | 4.7858 | 0.7434 | 0.9996 | NAD dependent epimerase/dehydratase [Source:VB Community Annotation] |
| AAEL012998 | | 0.1242 | 3.0052 | 0.7435 | 0.9996 | |
| AAEL007064 | GNBPB6 | -0.5381 | 9.4453 | 0.7435 | 0.9996 | Gram-Negative Binding Protein (GNBP)  or Beta-1 3-Glucan Binding Protein (BGBP). [Source:VB Community Annotation] |
| AAEL003211 | | 0.1554 | 8.0178 | 0.7435 | 0.9996 | beta-carotene dioxygenase [Source:VB Community Annotation] |
| AAEL002900 | | -0.1771 | 6.1207 | 0.7435 | 0.9996 | |
| AAEL006158 | | 0.0634 | 7.7852 | 0.7435 | 0.9996 | histone H3.3 [Source:VB Community Annotation] |
| AAEL015016 | | 0.1715 | 3.9667 | 0.7435 | 0.9996 | importin alpha 1a, putative [Source:VB Community Annotation] |
| AAEL008850 | | -0.1071 | 3.3377 | 0.7436 | 0.9996 | cak assembly factor [Source:VB Community Annotation] |
| AAEL011655 | | 0.1483 | 3.2689 | 0.7437 | 0.9996 | aspartyl-tRNA synthetase [Source:VB Community Annotation] |
| AAEL008061 | | -0.1144 | 3.6232 | 0.7438 | 0.9996 | phosphatidylinositolglycan class N, putative [Source:VB Community Annotation] |
| AAEL002163 | | 0.1080 | 5.1592 | 0.7438 | 0.9996 | |
| AAEL023985 | NA | -0.5402 | 0.6185 | 0.7440 | 0.9996 | NA |
| AAEL004768 | | -0.1073 | 8.3638 | 0.7441 | 0.9996 | |
| AAEL010437 | | -0.1235 | 4.4709 | 0.7442 | 0.9996 | heparan n-sulfatase [Source:VB Community Annotation] |
| AAEL019785 | NA | -0.0976 | 5.8280 | 0.7443 | 0.9996 | NA |
| AAEL001544 | | -0.1187 | 7.8005 | 0.7443 | 0.9996 | |
| AAEL005256 | | -0.3341 | 3.0168 | 0.7444 | 0.9996 | |
| AAEL003052 | | -0.4572 | -0.1533 | 0.7445 | 0.9996 | |
| AAEL018323 | | -0.2574 | 4.8083 | 0.7445 | 0.9996 | |
| AAEL000127 | | -0.1914 | 3.0345 | 0.7445 | 0.9996 | AMP dependent coa ligase [Source:VB Community Annotation] |
| AAEL012722 | | -0.3354 | -0.4460 | 0.7446 | 0.9996 | |
| AAEL010647 | | 0.3211 | -0.6226 | 0.7447 | 0.9996 | |
| AAEL004572 | | -0.4715 | 4.3815 | 0.7447 | 0.9996 | |
| AAEL007360 | | 0.1250 | 4.9416 | 0.7449 | 0.9996 | lethal(3)malignant brain tumor [Source:VB Community Annotation] |
| AAEL006718 | | 0.1418 | 4.9552 | 0.7449 | 0.9996 | Sugar transporter SWEET [Source:UniProtKB/TrEMBL;Acc:Q174X0] |
| AAEL004126 | | -0.2151 | 4.2445 | 0.7449 | 0.9996 | sterol desaturase [Source:VB Community Annotation] |
| AAEL013267 | | -0.2587 | 1.5632 | 0.7449 | 0.9996 | |
| AAEL010344 | | -0.5389 | 2.6568 | 0.7450 | 0.9996 | SEC14, putative [Source:VB Community Annotation] |

| | | | | | | |
|---|---|---|---|---|---|---|
| AAEL008213 | | -0.1566 | 2.3437 | 0.7450 | 0.9996 | mitochondrial carrier protein [Source:VB Community Annotation] |
| AAEL027179 | NA | 0.3301 | 2.5629 | 0.7451 | 0.9996 | NA |
| AAEL013323 | | 0.3025 | 0.2277 | 0.7452 | 0.9996 | |
| AAEL003534 | | 0.2354 | 4.6171 | 0.7452 | 0.9996 | |
| AAEL011900 | | 0.5033 | 2.8658 | 0.7454 | 0.9996 | N-acetyllactosaminide beta-1,3-N-acetylglucosaminyltransferase, putative [Source:VB Community Annotation] |
| AAEL008869 | | -0.2245 | 0.4542 | 0.7455 | 0.9996 | pupal cuticle protein 78E, putative [Source:VB Community Annotation] |
| AAEL007555 | | -0.1636 | 6.0077 | 0.7456 | 0.9996 | acyl-coa dehydrogenase [Source:VB Community Annotation] |
| AAEL027575 | NA | 0.3277 | 7.7905 | 0.7456 | 0.9996 | NA |
| AAEL004213 | | 0.7328 | -0.1818 | 0.7457 | 0.9996 | monocarboxylate transporter [Source:VB Community Annotation] |
| AAEL010496 | | 0.0902 | 5.1379 | 0.7457 | 0.9996 | |
| AAEL019426 | NA | -0.0924 | 5.3636 | 0.7459 | 0.9996 | NA |
| AAEL005779 | | -0.5492 | 1.1167 | 0.7459 | 0.9996 | CRAL/TRIO domain-containing protein [Source:VB Community Annotation] |
| AAEL014216 | | -0.5470 | -1.0024 | 0.7459 | 0.9996 | |
| AAEL022638 | NA | 0.4512 | 5.8428 | 0.7460 | 0.9996 | NA |
| AAEL010753 | | 0.1160 | 3.0593 | 0.7460 | 0.9996 | |
| AAEL010256 | | 0.1401 | 5.5680 | 0.7461 | 0.9996 | E3 ubiquitin ligase [Source:VB Community Annotation] |
| AAEL018029 | | 0.4612 | -0.8769 | 0.7462 | 0.9996 | |
| AAEL013227 | PIWI6 | 0.1421 | 5.0841 | 0.7464 | 0.9996 | PIWI [Source:VB Community Annotation] |
| AAEL006625 | | -0.1033 | 6.4987 | 0.7464 | 0.9996 | |
| AAEL025453 | NA | 0.4897 | 2.3859 | 0.7465 | 0.9996 | NA |
| AAEL004300 | | 0.0847 | 4.5318 | 0.7465 | 0.9996 | |
| AAEL011911 | | 0.0807 | 3.5726 | 0.7466 | 0.9996 | |
| AAEL002645 | | 0.1500 | 3.0546 | 0.7467 | 0.9996 | mannosyl-oligosaccharide alpha-1,2-mannosidase, putative [Source:VB Community Annotation] |
| AAEL025796 | NA | -0.4940 | 1.5901 | 0.7467 | 0.9996 | NA |
| AAEL009401 | | 0.1996 | 1.2734 | 0.7469 | 0.9996 | Methyltransferase-like protein [Source:UniProtKB/TrEMBL;Acc:Q16VX3] |
| AAEL008841 | | 0.1437 | 4.5180 | 0.7470 | 0.9996 | acyl-CoA oxidase [Source:VB Community Annotation] |
| AAEL005612 | | 0.4127 | -1.3261 | 0.7472 | 0.9996 | |
| AAEL019461 | NA | 0.1963 | 4.8165 | 0.7473 | 0.9996 | NA |
| AAEL007711 | | -0.4329 | 0.2710 | 0.7474 | 0.9996 | |
| AAEL019921 | NA | -0.1097 | 5.1699 | 0.7474 | 0.9996 | NA |
| AAEL026519 | NA | -0.2973 | 6.4692 | 0.7474 | 0.9996 | NA |
| AAEL014868 | | 0.1931 | 3.6656 | 0.7475 | 0.9996 | diaphanous [Source:VB Community Annotation] |
| AAEL013182 | | 0.4145 | 2.6613 | 0.7475 | 0.9996 | ATP-dependent RNA helicase [Source:VB Community Annotation] |
| AAEL026535 | NA | 0.0996 | 4.7213 | 0.7475 | 0.9996 | NA |
| AAEL020317 | NA | 0.1720 | 6.2845 | 0.7476 | 0.9996 | NA |
| AAEL005656 | | 0.5422 | 1.5331 | 0.7477 | 0.9996 | myosin heavy chain, nonmuscle or smooth muscle [Source:VB Community Annotation] |
| AAEL021072 | NA | 0.4334 | -0.1654 | 0.7478 | 0.9996 | NA |
| AAEL014227 | shakB | -0.2267 | 5.5985 | 0.7478 | 0.9996 | Innexin shaking-B [Source:UniProtKB/Swiss-Prot;Acc:Q1DH70] |
| AAEL018946 | tRNA-Asp | -0.2005 | 1.6350 | 0.7479 | 0.9996 | |
| AAEL000200 | | -0.2804 | 9.2943 | 0.7479 | 0.9996 | |

| | | | | | | |
|---|---|---|---|---|---|---|
| AAEL014152 | | 0.0898 | 5.5285 | 0.7481 | 0.9996 | |
| AAEL026551 | NA | -0.4660 | 5.3072 | 0.7481 | 0.9996 | NA |
| AAEL023967 | NA | -0.1448 | 6.2097 | 0.7482 | 0.9996 | NA |
| AAEL007386 | | 0.1305 | 3.6466 | 0.7482 | 0.9996 | |
| AAEL006197 | | 0.1581 | 3.1902 | 0.7483 | 0.9996 | |
| AAEL008941 | | -0.1380 | 3.3575 | 0.7483 | 0.9996 | |
| AAEL001114 | | 0.0914 | 4.6589 | 0.7484 | 0.9996 | amino acid transporter [Source:VB Community Annotation] |
| AAEL004474 | | 0.1520 | 5.9787 | 0.7485 | 0.9996 | non-receptor serine/threonine protein kinase [Source:VB Community Annotation] |
| AAEL009761 | | -0.6460 | -0.1362 | 0.7485 | 0.9996 | |
| AAEL019853 | NA | -0.2000 | 5.5099 | 0.7487 | 0.9996 | NA |
| AAEL012312 | | -0.1021 | 5.3949 | 0.7491 | 0.9996 | proliferation-associated 2g4 (pa2g4/ebp1) [Source:VB Community Annotation] |
| AAEL007716 | | 0.1350 | 3.3552 | 0.7492 | 0.9996 | Anoctamin [Source:UniProtKB/TrEMBL;Acc:Q0IEX5] |
| AAEL004869 | | 0.1414 | 2.8697 | 0.7494 | 0.9996 | |
| AAEL004125 | | -0.2746 | 3.6598 | 0.7495 | 0.9996 | signal transduction protein lnk-realted [Source:VB Community Annotation] |
| AAEL001319 | | 0.9161 | 3.7219 | 0.7496 | 0.9996 | |
| AAEL006275 | | -0.1177 | 3.4609 | 0.7497 | 0.9996 | |
| AAEL012840 | | -0.4390 | 4.9160 | 0.7497 | 0.9996 | stathmin [Source:VB Community Annotation] |
| AAEL025449 | NA | -0.3859 | -1.8999 | 0.7498 | 0.9996 | NA |
| AAEL020702 | NA | -0.4199 | 4.6018 | 0.7499 | 0.9996 | NA |
| AAEL010032 | | -0.0817 | 6.3813 | 0.7500 | 0.9996 | translocon-associated protein, beta subunit precursor (trap-beta) (signal sequence receptor beta subunit) [Source:VB Community Annota |
| AAEL026565 | NA | -0.1099 | 11.2542 | 0.7501 | 0.9996 | NA |
| AAEL012060 | PBAN | 0.2014 | 6.8864 | 0.7502 | 0.9996 | PBAN-type neuropeptides Precursor (Pheromone/pyrokinin biosynthesis-activating neuropeptide) [Source:VB Community Annotation] |
| AAEL008038 | | 0.1196 | 3.0503 | 0.7502 | 0.9996 | |
| AAEL005018 | | -0.0955 | 4.3700 | 0.7504 | 0.9996 | |
| AAEL005855 | | -0.2607 | 1.6797 | 0.7504 | 0.9996 | amino acid transporter [Source:VB Community Annotation] |
| AAEL004793 | | -0.0889 | 4.7039 | 0.7504 | 0.9996 | dipeptidyl-peptidase [Source:VB Community Annotation] |
| AAEL002967 | | -0.6395 | -0.6765 | 0.7504 | 0.9996 | |
| AAEL003582 | RpS13 | 0.0805 | 10.9475 | 0.7504 | 0.9996 | 40S ribosomal protein S13 [Source:UniProtKB/TrEMBL;Acc:Q5QC94] |
| AAEL004582 | | 0.1998 | 3.9379 | 0.7505 | 0.9996 | beta-galactosidase [Source:VB Community Annotation] |
| AAEL012397 | | 0.1353 | 2.5425 | 0.7506 | 0.9996 | ng,ng-dimethylarginine dimethylaminohydrolase [Source:VB Community Annotation] |
| AAEL028235 | NA | 0.1313 | 3.9953 | 0.7508 | 0.9996 | NA |
| AAEL019587 | NA | 0.5180 | -0.9900 | 0.7508 | 0.9996 | NA |
| AAEL009074 | IAP1 | 0.4003 | 3.8086 | 0.7508 | 0.9996 | Inhibitor of Apoptosis (IAP) containing Baculoviral IAP Repeat(s) (BIR domains). [Source:VB Community Annotation] |
| AAEL016599 | tRNA-Leu | -0.2460 | 0.2268 | 0.7508 | 0.9996 | |
| AAEL027698 | NA | -0.3301 | 0.0899 | 0.7509 | 0.9996 | NA |
| AAEL024005 | NA | -0.1623 | 4.2586 | 0.7510 | 0.9996 | NA |
| AAEL006469 | | -0.0979 | 4.2607 | 0.7510 | 0.9996 | RNA cytidine acetyltransferase [Source:UniProtKB/TrEMBL;Acc:Q176B3] |
| AAEL001747 | | 0.5048 | -0.9123 | 0.7511 | 0.9996 | Fatty acyl-CoA reductase (Fragment) [Source:UniProtKB/TrEMBL;Acc:Q17KB3] |
| AAEL020332 | NA | -0.1591 | 2.4748 | 0.7513 | 0.9996 | NA |
| AAEL023280 | NA | 0.1279 | 3.1028 | 0.7513 | 0.9996 | NA |

| | | | | | | |
|---|---|---|---|---|---|---|
| AAEL005093 | CLIPB46 | 0.3374 | 5.2429 | 0.7514 | 0.9996 | Clip-Domain Serine Protease family B. [Source:VB Community Annotation] |
| AAEL009191 | | 0.3263 | -0.3274 | 0.7516 | 0.9996 | adenosine deaminase [Source:VB Community Annotation] |
| AAEL008243 | | 0.1205 | 4.0597 | 0.7518 | 0.9996 | |
| AAEL005069 | | -0.1415 | 7.1349 | 0.7519 | 0.9996 | ras-related protein Rab-1A, putative [Source:VB Community Annotation] |
| AAEL020875 | NA | -0.1959 | 1.4369 | 0.7520 | 0.9996 | NA |
| AAEL012809 | | 0.1291 | 3.3393 | 0.7522 | 0.9996 | peptidylprolyl isomerase [Source:VB Community Annotation] |
| AAEL019885 | NA | -0.1569 | 7.3383 | 0.7522 | 0.9996 | NA |
| AAEL001878 | | -0.1875 | 1.6493 | 0.7523 | 0.9996 | lipase [Source:VB Community Annotation] |
| AAEL027143 | NA | 0.3402 | -0.0790 | 0.7524 | 0.9996 | NA |
| AAEL010117 | | -0.2833 | 1.0734 | 0.7525 | 0.9996 | fibrinogen and fibronectin [Source:VB Community Annotation] |
| AAEL007573 | | -0.1287 | 4.1652 | 0.7525 | 0.9996 | |
| AAEL009129 | CYP6Z9 | 0.1066 | 7.6360 | 0.7526 | 0.9996 | cytochrome P450 [Source:VB Community Annotation] |
| AAEL008100 | | 0.1164 | 5.8548 | 0.7526 | 0.9996 | |
| AAEL021180 | NA | -0.2587 | 2.5321 | 0.7528 | 0.9996 | NA |
| AAEL023289 | NA | 0.3214 | -0.4187 | 0.7528 | 0.9996 | NA |
| AAEL000923 | | 0.4281 | -0.1649 | 0.7529 | 0.9996 | |
| AAEL011156 | | -0.0842 | 6.8908 | 0.7529 | 0.9996 | |
| AAEL003679 | | 0.2115 | 0.9538 | 0.7529 | 0.9996 | |
| AAEL025264 | NA | -0.2722 | 0.0115 | 0.7530 | 0.9996 | NA |
| AAEL009125 | CYP6M10 | 0.0995 | 4.1073 | 0.7530 | 0.9996 | cytochrome P450 [Source:VB Community Annotation] |
| AAEL015062 | | -0.0888 | 4.7164 | 0.7531 | 0.9996 | |
| AAEL005663 | | -0.0760 | 5.3568 | 0.7533 | 0.9996 | centrin [Source:VB Community Annotation] |
| AAEL014098 | | -0.1122 | 5.2875 | 0.7534 | 0.9996 | polycomb protein [Source:VB Community Annotation] |
| AAEL014237 | | -0.1150 | 3.2295 | 0.7535 | 0.9996 | |
| AAEL012178 | | -0.4680 | 1.3888 | 0.7536 | 0.9996 | |
| AAEL026785 | NA | 0.3509 | -1.3806 | 0.7536 | 0.9996 | NA |
| AAEL000176 | | -0.3752 | 1.1677 | 0.7537 | 0.9996 | |
| AAEL013102 | | -0.4100 | 4.9457 | 0.7538 | 0.9996 | |
| AAEL019616 | NA | -0.2994 | 5.1397 | 0.7538 | 0.9996 | NA |
| AAEL003309 | | 0.5046 | 2.2282 | 0.7539 | 0.9996 | Alkaline phosphatase [Source:UniProtKB/TrEMBL;Acc:Q17FS5] |
| AAEL000509 | | -0.1103 | 3.7067 | 0.7540 | 0.9996 | rho GTPase activating protein [Source:VB Community Annotation] |
| AAEL025782 | NA | -0.1594 | 6.1290 | 0.7541 | 0.9996 | NA |
| AAEL021853 | NA | 0.4084 | -1.7952 | 0.7541 | 0.9996 | NA |
| AAEL006654 | | -0.1826 | 5.5968 | 0.7541 | 0.9996 | |
| AAEL020671 | NA | 0.1461 | 3.8284 | 0.7542 | 0.9996 | NA |
| AAEL005592 | | 0.1858 | 1.5865 | 0.7542 | 0.9996 | |
| AAEL001416 | | -0.2173 | 3.6387 | 0.7543 | 0.9996 | |
| AAEL013513 | | 0.4372 | 1.3430 | 0.7543 | 0.9996 | |
| AAEL013334 | | 0.1506 | 6.7146 | 0.7543 | 0.9996 | |
| AAEL003404 | | 0.3891 | -0.5210 | 0.7543 | 0.9996 | |

| | | | | | | |
|---|---|---|---|---|---|---|
| AAEL021119 | NA | -0.2197 | 0.5398 | 0.7543 | 0.9996 | NA |
| AAEL019475 | NA | -0.4863 | 2.2653 | 0.7543 | 0.9996 | NA |
| AAEL006175 | | -0.0908 | 4.9142 | 0.7544 | 0.9996 | |
| AAEL019504 | NA | 0.2416 | 4.1522 | 0.7546 | 0.9996 | NA |
| AAEL014393 | | 0.1273 | 7.5878 | 0.7546 | 0.9996 | lactoylglutathione lyase [Source:VB Community Annotation] |
| AAEL001463 | | 0.1651 | 1.9063 | 0.7546 | 0.9996 | |
| AAEL006522 | | -0.2695 | 6.2059 | 0.7547 | 0.9996 | turtle protein, isoform [Source:VB Community Annotation] |
| AAEL003554 | | 0.1063 | 4.7168 | 0.7548 | 0.9996 | leucine rich repeat protein [Source:VB Community Annotation] |
| AAEL002550 | | -0.4908 | 2.9986 | 0.7550 | 0.9996 | polyA-binding protein interacting protein, putative [Source:VB Community Annotation] |
| AAEL002559 | | 0.1709 | 5.9220 | 0.7550 | 0.9996 | |
| AAEL015412 | | 0.2545 | 0.0788 | 0.7551 | 0.9996 | metalloproteinase, putative [Source:VB Community Annotation] |
| AAEL014435 | | 0.2736 | 0.6247 | 0.7551 | 0.9996 | juvenile hormone-inducible protein, putative [Source:VB Community Annotation] |
| AAEL009754 | | 0.0677 | 5.2340 | 0.7551 | 0.9996 | endophilin b [Source:VB Community Annotation] |
| AAEL007321 | | 0.4196 | -1.8822 | 0.7551 | 0.9996 | |
| AAEL025083 | NA | -0.3743 | -0.2550 | 0.7551 | 0.9996 | NA |
| AAEL015136 | | 0.7149 | 1.9061 | 0.7551 | 0.9996 | Niemann-Pick Type C-2, putative [Source:VB Community Annotation] |
| AAEL019795 | NA | 0.1662 | 2.9553 | 0.7551 | 0.9996 | NA |
| AAEL011389 | | 0.1167 | 3.6942 | 0.7552 | 0.9996 | |
| AAEL005237 | | -0.1069 | 5.1420 | 0.7553 | 0.9996 | |
| AAEL002228 | | 0.5919 | 1.2281 | 0.7553 | 0.9996 | fatty acid synthase [Source:VB Community Annotation] |
| AAEL013205 | | 0.0899 | 3.7220 | 0.7553 | 0.9996 | rad25/xp-b DNA repair helicase [Source:VB Community Annotation] |
| AAEL025198 | NA | -0.1982 | 1.2687 | 0.7554 | 0.9996 | NA |
| AAEL013526 | | 0.1503 | 3.6005 | 0.7556 | 0.9996 | |
| AAEL007543 | | -0.3178 | 1.8665 | 0.7557 | 0.9996 | |
| AAEL019626 | NA | -0.5296 | 2.4619 | 0.7557 | 0.9996 | NA |
| AAEL020486 | NA | -0.5798 | -0.5310 | 0.7559 | 0.9996 | NA |
| AAEL005035 | | 0.3941 | -1.9102 | 0.7561 | 0.9996 | 1,4-dihydroxy-2-naphthoate octaprenyltransferase [Source:VB Community Annotation] |
| AAEL007446 | | -0.1766 | 0.5311 | 0.7561 | 0.9996 | |
| AAEL008397 | GPXH2 | 0.1172 | 8.7060 | 0.7565 | 0.9996 | Glutathione peroxidase [Source:UniProtKB/TrEMBL;Acc:Q16YX1] |
| AAEL013885 | | 0.5656 | 5.6978 | 0.7565 | 0.9996 | |
| AAEL027371 | NA | 0.3513 | 1.8696 | 0.7565 | 0.9996 | NA |
| AAEL009703 | | 0.1202 | 5.0925 | 0.7566 | 0.9996 | |
| AAEL002145 | | 0.0990 | 5.5894 | 0.7568 | 0.9996 | gonadotropin inducible transcription factor [Source:VB Community Annotation] |
| AAEL020812 | NA | -0.3248 | -0.6496 | 0.7568 | 0.9996 | NA |
| AAEL019438 | NA | -0.5561 | 2.0150 | 0.7569 | 0.9996 | NA |
| AAEL022309 | NA | 0.1141 | 4.0744 | 0.7569 | 0.9996 | NA |
| AAEL012360 | | 0.1043 | 4.8822 | 0.7570 | 0.9996 | |
| AAEL009048 | | 0.1289 | 3.9857 | 0.7571 | 0.9996 | |
| AAEL007975 | | -0.0907 | 5.7161 | 0.7572 | 0.9996 | |
| AAEL014958 | | -0.1150 | 4.7950 | 0.7573 | 0.9996 | galactokinase [Source:VB Community Annotation] |

| | | | | | | |
|---|---|---|---|---|---|---|
| AAEL004417 | | 0.0848 | 6.3731 | 0.7573 | 0.9996 | |
| AAEL012538 | LRIM6 | -0.3768 | 3.1464 | 0.7573 | 0.9996 | leucine-rich immune protein (Short) [Source:VB Community Annotation] |
| AAEL006042 | | 0.1998 | 1.3897 | 0.7574 | 0.9996 | arginine or creatine kinase [Source:VB Community Annotation] |
| AAEL008848 | | 0.1084 | 9.9550 | 0.7575 | 0.9996 | ATP synthase gamma subunit [Source:VB Community Annotation] |
| AAEL007245 | | -0.4832 | 2.9861 | 0.7576 | 0.9996 | WD40 protein [Source:VB Community Annotation] |
| AAEL010057 | | 0.0843 | 4.9543 | 0.7576 | 0.9996 | |
| AAEL003466 | | -0.1147 | 3.8493 | 0.7577 | 0.9996 | cullin [Source:VB Community Annotation] |
| AAEL006435 | | -0.1800 | 3.3730 | 0.7578 | 0.9996 | Mevalonate kinase [Source:UniProtKB/TrEMBL;Acc:Q176C1] |
| AAEL012578 | | 0.2038 | 6.2817 | 0.7579 | 0.9996 | Phosphoserine aminotransferase [Source:UniProtKB/TrEMBL;Acc:Q16LP7] |
| AAEL022315 | NA | 0.3130 | 0.3965 | 0.7579 | 0.9996 | NA |
| AAEL000035 | OBP57 | 0.1555 | 7.4441 | 0.7580 | 0.9996 | odorant binding protein (Obp57) [Source:VB Community Annotation] |
| AAEL012950 | | -0.1349 | 9.3745 | 0.7582 | 0.9996 | NADH-ubiquinone oxidoreductase 42 kda subunit [Source:VB Community Annotation] |
| AAEL011366 | | 0.1232 | 6.4342 | 0.7582 | 0.9996 | |
| AAEL005638 | | -0.1364 | 7.1052 | 0.7582 | 0.9996 | |
| AAEL012157 | | -0.2290 | 1.4397 | 0.7582 | 0.9996 | |
| AAEL004114 | | 0.2738 | 3.7723 | 0.7583 | 0.9996 | UNC93A protein, putative [Source:VB Community Annotation] |
| AAEL022113 | NA | 0.1254 | 5.2397 | 0.7585 | 0.9996 | NA |
| AAEL013072 | | -0.2292 | 3.9410 | 0.7586 | 0.9996 | |
| AAEL009495 | | -0.0881 | 5.3431 | 0.7586 | 0.9996 | rab6-interacting [Source:VB Community Annotation] |
| AAEL019868 | NA | 0.2346 | 5.7427 | 0.7586 | 0.9996 | NA |
| AAEL011985 | | -0.0994 | 6.6095 | 0.7587 | 0.9996 | |
| AAEL021963 | NA | -0.4034 | 2.6283 | 0.7587 | 0.9996 | NA |
| AAEL022373 | NA | 0.0931 | 4.1777 | 0.7590 | 0.9996 | NA |
| AAEL009399 | | -0.1031 | 3.8760 | 0.7590 | 0.9996 | |
| AAEL019521 | NA | 0.1283 | 3.4378 | 0.7590 | 0.9996 | NA |
| AAEL010661 | | 0.0850 | 4.5123 | 0.7590 | 0.9996 | phospholipid scramblase 1, [Source:VB Community Annotation] |
| AAEL024570 | NA | -0.4023 | 3.9097 | 0.7591 | 0.9996 | NA |
| AAEL011379 | | 0.1127 | 6.8546 | 0.7592 | 0.9996 | coenzyme q10 biosynthesis protein [Source:VB Community Annotation] |
| AAEL008789 | | -0.1736 | 9.5655 | 0.7592 | 0.9996 | apolipophorin-III, putative [Source:VB Community Annotation] |
| AAEL020581 | NA | -0.2935 | -0.1792 | 0.7595 | 0.9996 | NA |
| AAEL019867 | NA | -0.4019 | 4.1881 | 0.7596 | 0.9996 | NA |
| AAEL012523 | | 0.1302 | 2.7413 | 0.7597 | 0.9996 | transcription factor TFIIH-subunit, putative [Source:VB Community Annotation] |
| AAEL003387 | | -0.1644 | 5.6375 | 0.7599 | 0.9996 | cationic amino acid transporter [Source:VB Community Annotation] |
| AAEL022479 | NA | -0.4838 | -1.3896 | 0.7599 | 0.9996 | NA |
| AAEL012438 | | -0.0921 | 4.0630 | 0.7599 | 0.9996 | |
| AAEL004990 | | -0.0913 | 3.9932 | 0.7601 | 0.9996 | |
| AAEL010064 | | -0.5002 | 1.2029 | 0.7601 | 0.9996 | |
| AAEL005388 | | -0.4759 | -0.7019 | 0.7603 | 0.9996 | |
| AAEL008072 | | 0.3804 | -2.0822 | 0.7604 | 0.9996 | NADH-plastoquinone oxidoreductase [Source:VB Community Annotation] |
| AAEL002684 | | -0.6094 | 0.9292 | 0.7604 | 0.9996 | dachshund, [Source:VB Community Annotation] |

| | | | | | | |
|---|---|---|---|---|---|---|
| AAEL006684 | | -0.1386 | 4.9795 | 0.7604 | 0.9996 | Putative oxidoreductase GLYR1 homolog (EC 1.-.-.-)(Glyoxylate reductase 1 homolog)(Nuclear protein NP60 homolog) [Source:VB Commu |
| AAEL005025 | | 0.0988 | 6.1386 | 0.7606 | 0.9996 | U1 small nuclear ribonucleoprotein, putative [Source:VB Community Annotation] |
| AAEL021066 | NA | -0.3553 | 3.8059 | 0.7606 | 0.9996 | NA |
| AAEL000417 | | -0.1249 | 7.1201 | 0.7607 | 0.9996 | monocarboxylate transporter [Source:VB Community Annotation] |
| AAEL009078 | | -0.1518 | 8.8932 | 0.7607 | 0.9996 | NADH-ubiquinone oxidoreductase sgdh subunit [Source:VB Community Annotation] |
| AAEL020497 | NA | 0.3965 | -1.3283 | 0.7607 | 0.9996 | NA |
| AAEL025983 | NA | -0.1828 | 1.2683 | 0.7611 | 0.9996 | NA |
| AAEL025532 | NA | 0.3560 | 2.7757 | 0.7611 | 0.9996 | NA |
| AAEL023858 | NA | 0.2700 | 0.1743 | 0.7611 | 0.9996 | NA |
| AAEL005422 | | 0.1275 | 8.2171 | 0.7611 | 0.9996 | pyrroline-5-carboxylate dehydrogenase [Source:VB Community Annotation] |
| AAEL007278 | | -0.7001 | 0.5858 | 0.7612 | 0.9996 | defective proboscis extension response, putative [Source:VB Community Annotation] |
| AAEL004832 | | 0.2277 | 2.9441 | 0.7613 | 0.9996 | |
| AAEL026260 | NA | 0.3781 | 0.1070 | 0.7614 | 0.9996 | NA |
| AAEL012208 | | 0.0804 | 4.4827 | 0.7614 | 0.9996 | |
| AAEL000418 | | -0.0899 | 6.5762 | 0.7616 | 0.9996 | |
| AAEL005293 | GALE8A | 0.4214 | 0.6355 | 0.7616 | 0.9996 | Galectin [Source:UniProtKB/TrEMBL;Acc:Q16ND5] |
| AAEL024788 | NA | 0.1638 | 0.5683 | 0.7617 | 0.9996 | NA |
| AAEL024981 | NA | -0.4443 | -0.2128 | 0.7617 | 0.9996 | NA |
| AAEL012515 | | -0.0821 | 6.0178 | 0.7617 | 0.9996 | tumor suppressor protein, putative [Source:VB Community Annotation] |
| AAEL024447 | NA | 0.0893 | 3.1889 | 0.7618 | 0.9996 | NA |
| AAEL003104 | | 0.1535 | 5.3493 | 0.7619 | 0.9996 | tripartite motif protein trim2,3 [Source:VB Community Annotation] |
| AAEL019748 | NA | -0.0758 | 8.0888 | 0.7620 | 0.9996 | NA |
| AAEL005619 | | 0.1004 | 4.8565 | 0.7620 | 0.9996 | defective proboscis extension response, putative [Source:VB Community Annotation] |
| AAEL004482 | | 0.1503 | 3.2296 | 0.7621 | 0.9996 | |
| AAEL009586 | | -0.0797 | 4.5735 | 0.7625 | 0.9996 | |
| AAEL007831 | | -0.1396 | 1.5519 | 0.7625 | 0.9996 | |
| AAEL009379 | mRpL33 | -0.1322 | 7.0970 | 0.7625 | 0.9996 | mitochondrial ribosomal protein, L33, putative [Source:VB Community Annotation] |
| AAEL002518 | | 0.1189 | 3.9123 | 0.7626 | 0.9996 | glutamate receptor, ionotropic kainate 1, 2, 3 (glur5, glur6, glur7) [Source:VB Community Annotation] |
| AAEL012671 | | -0.0713 | 9.8117 | 0.7629 | 0.9996 | |
| AAEL006633 | IAP2 | 0.0849 | 4.1097 | 0.7630 | 0.9996 | Inhibitor of Apoptosis (IAP)  containing Baculoviral IAP Repeat(s) (BIR domains). [Source:VB Community Annotation] |
| AAEL015631 | | 0.1819 | 5.1323 | 0.7630 | 0.9996 | asparagine synthetase [Source:VB Community Annotation] |
| AAEL005468 | | -0.1877 | 2.7226 | 0.7631 | 0.9996 | glycosyltransferase [Source:VB Community Annotation] |
| AAEL004496 | | -0.2937 | 3.6915 | 0.7632 | 0.9996 | glutamate transporter [Source:VB Community Annotation] |
| AAEL002880 | | -0.1997 | 3.1411 | 0.7632 | 0.9996 | |
| AAEL000221 | | 0.0923 | 4.9480 | 0.7634 | 0.9996 | mediator complex, subunit, putative [Source:VB Community Annotation] |
| AAEL005446 | | -0.1002 | 5.1508 | 0.7634 | 0.9996 | 6-phosphofructo-2-kinase/fructose-2,6-bisphosphatase [Source:VB Community Annotation] |
| AAEL028094 | NA | -0.4998 | -0.6125 | 0.7635 | 0.9996 | NA |
| AAEL004613 | | 0.1183 | 6.5134 | 0.7635 | 0.9996 | phenylalanyl-tRNA synthetase beta chain [Source:VB Community Annotation] |
| AAEL001175 | | -0.3663 | -0.8486 | 0.7635 | 0.9996 | DNA photolyase [Source:VB Community Annotation] |
| AAEL012766 | CYP325G2 | -0.1715 | 7.0682 | 0.7636 | 0.9996 | cytochrome P450 [Source:VB Community Annotation] |

| | | | | | | |
|---|---|---|---|---|---|---|
| AAEL005822 | | -0.1571 | 2.7029 | 0.7637 | 0.9996 | |
| AAEL018133 | | -0.1573 | 4.2310 | 0.7637 | 0.9996 | |
| AAEL027215 | NA | 0.2744 | 3.0833 | 0.7639 | 0.9996 | NA |
| AAEL006265 | | -0.3804 | 4.6372 | 0.7639 | 0.9996 | |
| AAEL008012 | | 0.1564 | 5.5689 | 0.7641 | 0.9996 | |
| AAEL025514 | NA | 0.0948 | 5.9147 | 0.7642 | 0.9996 | NA |
| AAEL003011 | | -0.1150 | 8.2230 | 0.7642 | 0.9996 | NADH dehydrogenase, putative [Source:VB Community Annotation] |
| AAEL014102 | | 0.3755 | 2.9412 | 0.7643 | 0.9996 | |
| AAEL023122 | NA | -0.2215 | 0.1675 | 0.7643 | 0.9996 | NA |
| AAEL005070 | | 0.1283 | 5.0167 | 0.7644 | 0.9996 | |
| AAEL004436 | | -0.0723 | 6.1587 | 0.7644 | 0.9996 | |
| AAEL024479 | NA | -0.1226 | 5.3701 | 0.7645 | 0.9996 | NA |
| AAEL005187 | | 0.2983 | 2.0299 | 0.7646 | 0.9996 | laminin gamma 1 chain [Source:VB Community Annotation] |
| AAEL006704 | | 0.6363 | 4.8696 | 0.7646 | 0.9996 | fibrinogen and fibronectin [Source:VB Community Annotation] |
| AAEL018354 | OSBP | -0.1364 | 3.2574 | 0.7647 | 0.9996 | oxysterol-binding protein [Source:VB Community Annotation] |
| AAEL013042 | | -0.5677 | 2.5782 | 0.7647 | 0.9996 | |
| AAEL020778 | NA | 0.1567 | 3.1816 | 0.7647 | 0.9996 | NA |
| AAEL026118 | NA | 0.1798 | 3.9876 | 0.7648 | 0.9996 | NA |
| AAEL022775 | NA | -0.1440 | 4.0873 | 0.7649 | 0.9996 | NA |
| AAEL009167 | | -0.1121 | 4.5613 | 0.7649 | 0.9996 | bone morphogenetic protein 5/7, bmp5/7 [Source:VB Community Annotation] |
| AAEL005793 | | -0.1050 | 7.3450 | 0.7649 | 0.9996 | AMP dependent ligase [Source:VB Community Annotation] |
| AAEL003957 | | 0.1048 | 7.6680 | 0.7649 | 0.9996 | |
| AAEL011359 | | -0.3498 | 4.2815 | 0.7650 | 0.9996 | nfat [Source:VB Community Annotation] |
| AAEL019564 | NA | -0.1736 | 5.1541 | 0.7651 | 0.9996 | NA |
| AAEL013424 | | 0.0824 | 4.9155 | 0.7651 | 0.9996 | |
| AAEL011561 | | -0.1647 | 2.2040 | 0.7651 | 0.9996 | |
| AAEL010007 | | 0.2032 | 3.6828 | 0.7651 | 0.9996 | |
| AAEL014265 | | 0.1828 | 3.4569 | 0.7652 | 0.9996 | |
| AAEL016711 | tRNA-Gly | -0.2909 | -1.2324 | 0.7652 | 0.9996 | |
| AAEL006417 | | -0.4929 | 9.1148 | 0.7652 | 0.9996 | D7 protein, putative [Source:VB Community Annotation] |
| AAEL025762 | NA | -0.1150 | 4.1806 | 0.7654 | 0.9996 | NA |
| AAEL005492 | | -0.0864 | 6.6675 | 0.7654 | 0.9996 | protein-tyrosine phosphatase n9 [Source:VB Community Annotation] |
| AAEL001963 | | -0.1150 | 6.2483 | 0.7654 | 0.9996 | protein serine/threonine kinase, putative [Source:VB Community Annotation] |
| AAEL020444 | NA | 0.3158 | -1.3347 | 0.7655 | 0.9996 | NA |
| AAEL001657 | | 0.0946 | 4.9210 | 0.7655 | 0.9996 | DEAD box ATP-dependent RNA helicase [Source:VB Community Annotation] |
| AAEL020599 | NA | 0.3888 | -0.1208 | 0.7655 | 0.9996 | NA |
| AAEL012585 | RpL7 | 0.0897 | 10.9382 | 0.7657 | 0.9996 | 60S ribosomal protein L7 [Source:VB Community Annotation] |
| AAEL012071 | | -0.0750 | 6.2212 | 0.7657 | 0.9996 | ras [Source:VB Community Annotation] |
| AAEL020432 | NA | 0.1447 | 3.1819 | 0.7657 | 0.9996 | NA |
| AAEL019681 | NA | 0.0914 | 6.3114 | 0.7658 | 0.9996 | NA |

| | | | | | | |
|---|---|---|---|---|---|---|
| AAEL009695 | | -0.1496 | 7.8327 | 0.7658 | 0.9996 | |
| AAEL026398 | NA | 0.2169 | 3.7345 | 0.7659 | 0.9996 | NA |
| AAEL009427 | | 0.1409 | 3.0685 | 0.7659 | 0.9996 | |
| AAEL001827 | | 0.0804 | 4.0690 | 0.7659 | 0.9996 | ubiquitin specific protease 39 and snrnp assembly factor [Source:VB Community Annotation] |
| AAEL007994 | | 0.2562 | 0.8336 | 0.7660 | 0.9996 | |
| AAEL010488 | | -0.2454 | 3.7861 | 0.7663 | 0.9996 | ets [Source:VB Community Annotation] |
| AAEL007011 | | -0.1746 | 4.9981 | 0.7663 | 0.9996 | |
| AAEL011714 | | -0.2079 | 1.2200 | 0.7664 | 0.9996 | |
| AAEL000187 | | -0.5268 | 0.8984 | 0.7664 | 0.9996 | lim-kinase1 [Source:VB Community Annotation] |
| AAEL007884 | | 0.1557 | 3.1314 | 0.7664 | 0.9996 | conserved membrane protein at 44E, putative [Source:VB Community Annotation] |
| AAEL006067 | | -0.0998 | 3.3374 | 0.7664 | 0.9996 | |
| AAEL019705 | NA | -0.1029 | 3.9104 | 0.7665 | 0.9996 | NA |
| AAEL022308 | NA | -0.4517 | -0.0556 | 0.7667 | 0.9996 | NA |
| AAEL011368 | | -0.2309 | 3.7728 | 0.7668 | 0.9996 | sugar transporter [Source:VB Community Annotation] |
| AAEL006617 | | 0.1751 | 4.4591 | 0.7668 | 0.9996 | |
| AAEL019779 | NA | 0.2839 | 3.0113 | 0.7669 | 0.9996 | NA |
| AAEL010118 | | 0.1145 | 4.2552 | 0.7671 | 0.9996 | kelch repeat protein [Source:VB Community Annotation] |
| AAEL002335 | | 0.2370 | 2.6003 | 0.7672 | 0.9996 | atbf1 [Source:VB Community Annotation] |
| AAEL014523 | | -0.5544 | 1.9812 | 0.7672 | 0.9996 | neuroendocrine convertase [Source:VB Community Annotation] |
| AAEL013389 | | -0.1281 | 4.0230 | 0.7675 | 0.9996 | coproporphyrinogen iii oxidase [Source:VB Community Annotation] |
| AAEL002412 | | 0.1834 | 5.0024 | 0.7675 | 0.9996 | monocarboxylate transporter [Source:VB Community Annotation] |
| AAEL002789 | | -0.0751 | 6.2248 | 0.7675 | 0.9996 | 26S proteasome non-ATPase regulatory subunit [Source:VB Community Annotation] |
| AAEL006812 | | 0.1112 | 2.8664 | 0.7677 | 0.9996 | |
| AAEL002455 | | -0.1971 | 3.8171 | 0.7678 | 0.9996 | nucleoporin, Nup153, putative [Source:VB Community Annotation] |
| AAEL023907 | NA | -0.1232 | 2.8687 | 0.7679 | 0.9996 | NA |
| AAEL011562 | Dronc | 0.1050 | 4.5680 | 0.7680 | 0.9996 | caspase (long) [Source:VB Community Annotation] |
| AAEL004492 | | 0.1872 | 0.9518 | 0.7680 | 0.9996 | cyclin-dependent kinases regulatory subunit. putative [Source:VB Community Annotation] |
| AAEL020617 | NA | 0.4333 | -1.2986 | 0.7681 | 0.9996 | NA |
| AAEL009517 | | 0.3852 | 0.6626 | 0.7682 | 0.9996 | lumican, putative [Source:VB Community Annotation] |
| AAEL002306 | | -0.1158 | 6.6798 | 0.7683 | 0.9996 | hect E3 ubiquitin ligase [Source:VB Community Annotation] |
| AAEL004997 | | 0.1274 | 4.8216 | 0.7683 | 0.9996 | U3 small nucleolar ribonucleoprotein protein imp4 [Source:VB Community Annotation] |
| AAEL001201 | | -0.1603 | 4.3451 | 0.7684 | 0.9996 | |
| AAEL023651 | NA | -0.3126 | 6.0308 | 0.7684 | 0.9996 | NA |
| AAEL003047 | | -0.1960 | 3.0354 | 0.7684 | 0.9996 | netrin [Source:VB Community Annotation] |
| AAEL010592 | | 0.1498 | 5.2180 | 0.7684 | 0.9996 | esterase, putative [Source:VB Community Annotation] |
| AAEL025013 | NA | -0.3879 | -0.4024 | 0.7685 | 0.9996 | NA |
| AAEL013645 | | -0.4998 | -1.3712 | 0.7685 | 0.9996 | |
| AAEL001638 | | 0.0646 | 4.8946 | 0.7685 | 0.9996 | |
| AAEL012960 | | -0.2219 | 0.1780 | 0.7685 | 0.9996 | importin alpha [Source:VB Community Annotation] |
| AAEL026353 | NA | -0.4776 | 1.4969 | 0.7686 | 0.9996 | NA |

| | | | | | | |
|---|---|---|---|---|---|---|
| AAEL001915 | | -0.4189 | 0.1255 | 0.7686 | 0.9996 | tyrosine protein kinase [Source:VB Community Annotation] |
| AAEL012645 | | 0.3185 | -2.8535 | 0.7686 | 0.9996 | |
| AAEL004392 | IMP | 0.1709 | 3.3952 | 0.7687 | 0.9996 | IAP-antagonist Michelob_x-like Protein [Source:VB Community Annotation] |
| AAEL003026 | | -0.5780 | 3.9569 | 0.7687 | 0.9996 | regulator of g protein signaling [Source:VB Community Annotation] |
| AAEL005772 | OBP22 | -0.2108 | 10.2274 | 0.7688 | 0.9996 | odorant binding protein OBP22 [Source:VB Community Annotation] |
| AAEL011746 | skap | 0.0851 | 8.4177 | 0.7688 | 0.9996 | succinyl-CoA ligase beta subunit [Source:VB Community Annotation] |
| AAEL013440 | | -0.0983 | 4.8359 | 0.7688 | 0.9996 | |
| AAEL023141 | NA | -0.5553 | 0.9964 | 0.7691 | 0.9996 | NA |
| AAEL013844 | | -0.1177 | 5.7346 | 0.7691 | 0.9996 | diazepam binding inhibitor, putative [Source:VB Community Annotation] |
| AAEL001818 | | 0.1822 | 3.6218 | 0.7693 | 0.9996 | |
| AAEL008811 | | -0.1095 | 5.3744 | 0.7693 | 0.9996 | |
| AAEL001997 | | -0.1234 | 3.2171 | 0.7694 | 0.9996 | |
| AAEL009955 | | -0.1228 | 8.3077 | 0.7694 | 0.9996 | |
| AAEL018208 | | -0.1275 | 3.9430 | 0.7695 | 0.9996 | |
| AAEL007230 | Med30 | -0.0812 | 4.1128 | 0.7695 | 0.9996 | Mediator of RNA polymerase II transcription subunit 30 (Med30) [Source:VB Community Annotation] |
| AAEL010010 | | 0.1460 | 2.9389 | 0.7695 | 0.9996 | |
| AAEL019755 | NA | -0.3878 | 4.4609 | 0.7696 | 0.9996 | NA |
| AAEL006283 | GPRDMS | 0.4588 | 1.0968 | 0.7698 | 0.9996 | GPCR Myosuppressin Family [Source:VB Community Annotation] |
| AAEL013505 | GPRNPY7 | -0.4375 | 1.0749 | 0.7698 | 0.9996 | GPCR Neuropeptide Y Family [Source:VB Community Annotation] |
| AAEL009895 | | -0.0690 | 5.5968 | 0.7700 | 0.9996 | neprilysin [Source:VB Community Annotation] |
| AAEL011054 | | 0.0867 | 4.7811 | 0.7701 | 0.9996 | |
| AAEL010826 | | -0.0998 | 7.9058 | 0.7702 | 0.9996 | histone-lysine n-methyltransferase [Source:VB Community Annotation] |
| AAEL005736 | | 0.2968 | 4.8076 | 0.7703 | 0.9996 | |
| AAEL025334 | NA | -0.3182 | -0.7019 | 0.7703 | 0.9996 | NA |
| AAEL025076 | NA | 0.6533 | 1.4732 | 0.7704 | 0.9996 | NA |
| AAEL007248 | | -0.1047 | 3.1121 | 0.7705 | 0.9996 | |
| AAEL018183 | | 0.1453 | 3.9055 | 0.7706 | 0.9996 | |
| AAEL002569 | | -0.1971 | 1.4119 | 0.7706 | 0.9996 | serine/threonine kinase [Source:VB Community Annotation] |
| AAEL019758 | NA | -0.0756 | 5.2091 | 0.7706 | 0.9996 | NA |
| AAEL011330 | | -0.1879 | 0.9519 | 0.7706 | 0.9996 | |
| AAEL006640 | | 0.0910 | 4.1225 | 0.7706 | 0.9996 | DEAD box ATP-dependent RNA helicase [Source:VB Community Annotation] |
| AAEL004561 | | 0.1898 | 0.6624 | 0.7707 | 0.9996 | |
| AAEL017135 | | -0.2950 | 4.1333 | 0.7710 | 0.9996 | |
| AAEL013636 | | 0.1254 | 4.6177 | 0.7710 | 0.9996 | translation elongation factor g [Source:VB Community Annotation] |
| AAEL007129 | | -0.1380 | 3.0935 | 0.7713 | 0.9996 | defective proboscis extension response, putative [Source:VB Community Annotation] |
| AAEL006723 | | 0.1043 | 3.7639 | 0.7714 | 0.9996 | |
| AAEL010060 | | 0.0754 | 6.0610 | 0.7714 | 0.9996 | signal recognition particle 68 kda protein [Source:VB Community Annotation] |
| AAEL017564 | Or71 | 0.1700 | 0.4675 | 0.7715 | 0.9996 | Odorant receptor [Source:UniProtKB/TrEMBL;Acc:J9HRY1] |
| AAEL009957 | | -0.1062 | 3.4793 | 0.7716 | 0.9996 | lipocalin-1 interacting membrane receptor (limr) [Source:VB Community Annotation] |
| AAEL004942 | | -0.0946 | 4.8033 | 0.7719 | 0.9996 | helicase [Source:VB Community Annotation] |

| | | | | | | |
|---|---|---|---|---|---|---|
| AAEL011048 | | -0.0789 | 5.4436 | 0.7720 | 0.9996 | |
| AAEL007659 | | -0.0605 | 8.2398 | 0.7720 | 0.9996 | |
| AAEL006824 | | -0.5964 | 2.8716 | 0.7722 | 0.9996 | cytochrome P450 [Source:VB Community Annotation] |
| AAEL001943 | mRpS34 | 0.0923 | 5.6022 | 0.7722 | 0.9996 | mitochondrial ribosomal protein, S34, putative [Source:VB Community Annotation] |
| AAEL010494 | | -0.1834 | 1.2421 | 0.7722 | 0.9996 | |
| AAEL010514 | | -0.1040 | 6.0464 | 0.7723 | 0.9996 | aminoacyl-tRNA synthetase auxiliary protein, 43kD, putative [Source:VB Community Annotation] |
| AAEL024925 | NA | 0.1211 | 3.1385 | 0.7724 | 0.9996 | NA |
| AAEL005868 | | -0.5550 | 0.9781 | 0.7724 | 0.9996 | |
| AAEL018232 | | -0.5036 | 3.7191 | 0.7725 | 0.9996 | |
| AAEL004368 | | -0.1204 | 3.4123 | 0.7725 | 0.9996 | nucleolar RNA-associated protein [Source:VB Community Annotation] |
| AAEL005450 | | 0.2128 | 1.8013 | 0.7725 | 0.9996 | |
| AAEL013380 | | -0.3469 | -1.2681 | 0.7727 | 0.9996 | adult cuticle protein, putative [Source:VB Community Annotation] |
| AAEL022711 | NA | -0.6883 | -1.8367 | 0.7728 | 0.9996 | NA |
| AAEL021395 | NA | -0.0868 | 4.3017 | 0.7729 | 0.9996 | NA |
| AAEL011155 | | -0.1211 | 2.8696 | 0.7731 | 0.9996 | |
| AAEL013449 | | 0.3887 | -2.7583 | 0.7732 | 0.9996 | metalloproteinase, putative [Source:VB Community Annotation] |
| AAEL013857 | | 0.2550 | 4.4622 | 0.7732 | 0.9996 | |
| AAEL004338 | | 0.0936 | 6.4406 | 0.7732 | 0.9996 | pyruvate dehydrogenase [Source:VB Community Annotation] |
| AAEL022033 | NA | 0.2739 | 1.7175 | 0.7732 | 0.9996 | NA |
| AAEL006359 | | 0.2600 | 1.6105 | 0.7734 | 0.9996 | sulfotransferase (sult) [Source:VB Community Annotation] |
| AAEL011287 | | -0.1981 | 4.7025 | 0.7734 | 0.9996 | ubiquitin specific protease [Source:VB Community Annotation] |
| AAEL021084 | NA | -0.5209 | 1.9415 | 0.7735 | 0.9996 | NA |
| AAEL020586 | NA | -0.3554 | 3.5198 | 0.7735 | 0.9996 | NA |
| AAEL001457 | | 0.0799 | 6.5854 | 0.7736 | 0.9996 | guanylatte cyclase alpha 1 subunit [Source:VB Community Annotation] |
| AAEL004488 | | -0.1681 | 5.8871 | 0.7737 | 0.9996 | |
| AAEL027944 | NA | 0.3887 | -1.8381 | 0.7738 | 0.9996 | NA |
| AAEL014302 | | -0.1017 | 4.3954 | 0.7738 | 0.9996 | Fatty acyl-CoA reductase [Source:UniProtKB/TrEMBL;Acc:Q16GQ4] |
| AAEL026044 | NA | -0.1012 | 8.5810 | 0.7739 | 0.9996 | NA |
| AAEL000217 | | -0.4333 | 2.8032 | 0.7740 | 0.9996 | serine/threonine protein kinase [Source:VB Community Annotation] |
| AAEL019424 | NA | -0.2691 | 6.1534 | 0.7741 | 0.9996 | NA |
| AAEL013044 | | -0.1252 | 1.9038 | 0.7741 | 0.9996 | |
| AAEL004589 | | -0.0682 | 6.5831 | 0.7741 | 0.9996 | small calcium-binding mitochondrial carrier, putative [Source:VB Community Annotation] |
| AAEL019772 | NA | -0.1102 | 4.4625 | 0.7746 | 0.9996 | NA |
| AAEL026817 | NA | -0.0947 | 3.8547 | 0.7747 | 0.9996 | NA |
| AAEL012990 | | 0.1850 | 1.1535 | 0.7747 | 0.9996 | mrg-binding protein [Source:VB Community Annotation] |
| AAEL011575 | | 0.0626 | 5.7783 | 0.7747 | 0.9996 | |
| AAEL009706 | | -0.0858 | 7.9615 | 0.7748 | 0.9996 | ns1 binding protein [Source:VB Community Annotation] |
| AAEL020002 | NA | -0.3560 | 2.6265 | 0.7748 | 0.9996 | NA |
| AAEL009666 | | -0.1347 | 3.4733 | 0.7750 | 0.9996 | set domain protein [Source:VB Community Annotation] |
| AAEL017053 | | 0.1084 | 3.3970 | 0.7750 | 0.9996 | |

| | | | | | | |
|---|---|---|---|---|---|---|
| AAEL012610 | | 0.0800 | 6.0347 | 0.7750 | 0.9996 | |
| AAEL021746 | NA | 0.1212 | 7.8206 | 0.7751 | 0.9996 | NA |
| AAEL004763 | | 0.0913 | 3.5313 | 0.7751 | 0.9996 | |
| AAEL004576 | | 0.1416 | 2.3262 | 0.7752 | 0.9996 | |
| AAEL004106 | | -0.2087 | 1.7378 | 0.7754 | 0.9996 | hairy protein [Source:VB Community Annotation] |
| AAEL022427 | NA | 0.1651 | 2.1735 | 0.7755 | 0.9996 | NA |
| AAEL006902 | | 0.3750 | -1.3455 | 0.7755 | 0.9996 | serine-type enodpeptidase, [Source:VB Community Annotation] |
| AAEL012930 | | 0.1791 | 5.3176 | 0.7755 | 0.9996 | |
| AAEL007805 | | -0.4625 | 2.2859 | 0.7756 | 0.9996 | |
| AAEL006661 | | 0.0889 | 6.5237 | 0.7756 | 0.9996 | histone acetyltransferase, putative [Source:VB Community Annotation] |
| AAEL022282 | NA | -0.0972 | 5.8363 | 0.7756 | 0.9996 | NA |
| AAEL004231 | | -0.1769 | 1.2002 | 0.7757 | 0.9996 | M12 mutant protein precursor, putative [Source:VB Community Annotation] |
| AAEL024295 | NA | 0.2660 | -0.6470 | 0.7758 | 0.9996 | NA |
| AAEL014719 | | -0.1271 | 4.3874 | 0.7758 | 0.9996 | inosine-uridine preferring nucleoside hydrolase [Source:VB Community Annotation] |
| AAEL008394 | | 0.0903 | 7.5979 | 0.7758 | 0.9996 | |
| AAEL000057 | TOLL5B | 0.3490 | -1.8040 | 0.7759 | 0.9996 | Toll-like receptor [Source:VB Community Annotation] |
| AAEL006294 | | -0.0985 | 3.9761 | 0.7759 | 0.9996 | |
| AAEL011499 | OBP47 | 0.1420 | 5.6606 | 0.7760 | 0.9996 | odorant binding protein OBP47 [Source:VB Community Annotation] |
| AAEL017805 | U5 | -0.3206 | -0.7256 | 0.7760 | 0.9996 | U5 spliceosomal RNA [Source:RFAM;Acc:RF00020] |
| AAEL008118 | mab-21 | -0.2644 | 4.1001 | 0.7761 | 0.9996 | Protein mab-21 [Source:UniProtKB/Swiss-Prot;Acc:Q0IES8] |
| AAEL019656 | NA | 0.0808 | 6.6097 | 0.7761 | 0.9996 | NA |
| AAEL001220 | ck | 0.1355 | 3.4662 | 0.7761 | 0.9996 | myosin-VIIa (Protein crinkled) [Source:VB Community Annotation] |
| AAEL009195 | | -0.0785 | 3.5090 | 0.7762 | 0.9996 | |
| AAEL024291 | NA | -0.1346 | 3.3110 | 0.7762 | 0.9996 | NA |
| AAEL001857 | | 0.2209 | 2.1437 | 0.7762 | 0.9996 | |
| AAEL014635 | | 0.3016 | -0.5503 | 0.7763 | 0.9996 | engrailed [Source:VB Community Annotation] |
| AAEL002211 | | -0.3366 | -2.4460 | 0.7763 | 0.9996 | cuticle protein, putative [Source:VB Community Annotation] |
| AAEL017013 | | 0.2599 | 1.4042 | 0.7764 | 0.9996 | |
| AAEL004634 | | 0.0907 | 3.0480 | 0.7764 | 0.9996 | Condensin complex subunit 1 [Source:UniProtKB/TrEMBL;Acc:Q17CA8] |
| AAEL004559 | | -0.2905 | 5.3968 | 0.7765 | 0.9996 | synaptosomal associated protein [Source:VB Community Annotation] |
| AAEL018351 | | -0.1713 | 2.4978 | 0.7769 | 0.9996 | |
| AAEL007794 | | -0.5257 | 3.0256 | 0.7771 | 0.9996 | defective proboscis extension response [Source:VB Community Annotation] |
| AAEL021010 | NA | 0.1250 | 3.6829 | 0.7772 | 0.9996 | NA |
| AAEL025709 | NA | -0.3328 | -0.4547 | 0.7773 | 0.9996 | NA |
| AAEL001173 | | 0.0642 | 5.3647 | 0.7773 | 0.9996 | amidase [Source:VB Community Annotation] |
| AAEL009120 | CYP6S3 | 0.1310 | 3.6205 | 0.7773 | 0.9996 | cytochrome P450 [Source:VB Community Annotation] |
| AAEL012370 | | -0.1893 | 5.9500 | 0.7775 | 0.9996 | brain chitinase and chia [Source:VB Community Annotation] |
| AAEL011807 | | 0.0938 | 2.8251 | 0.7776 | 0.9996 | |
| AAEL005212 | | 0.1871 | 3.4342 | 0.7779 | 0.9996 | |
| AAEL010321 | | 0.0798 | 4.2291 | 0.7781 | 0.9996 | porphobilinogen deaminase [Source:VB Community Annotation] |

| | | | | | | |
|---|---|---|---|---|---|---|
| AAEL001024 | | 0.0906 | 3.3732 | 0.7781 | 0.9996 | mitochondrial carrier protein [Source:VB Community Annotation] |
| AAEL008185 | | -0.1794 | 2.8034 | 0.7781 | 0.9996 | |
| AAEL003901 | | -0.3448 | 3.7165 | 0.7783 | 0.9996 | |
| AAEL027955 | NA | -0.4095 | -1.8359 | 0.7783 | 0.9996 | NA |
| AAEL021834 | NA | -0.3404 | 0.8761 | 0.7783 | 0.9996 | NA |
| AAEL025929 | NA | -0.1674 | 5.0843 | 0.7784 | 0.9996 | NA |
| AAEL019702 | NA | -0.4198 | -0.0771 | 0.7785 | 0.9996 | NA |
| AAEL020636 | NA | -0.1726 | 0.4345 | 0.7786 | 0.9996 | NA |
| AAEL024396 | NA | -0.4043 | 3.4796 | 0.7787 | 0.9996 | NA |
| AAEL026094 | NA | -0.4395 | -2.1823 | 0.7787 | 0.9996 | NA |
| AAEL021132 | NA | 0.2343 | 0.1711 | 0.7787 | 0.9996 | NA |
| AAEL004247 | | 0.1075 | 5.6230 | 0.7787 | 0.9996 | Sialin, Sodium/sialic acid cotransporter, putative [Source:VB Community Annotation] |
| AAEL009182 | | 0.1198 | 3.7194 | 0.7788 | 0.9996 | zinc finger protein, putative [Source:VB Community Annotation] |
| AAEL006234 | | -0.1961 | 1.2934 | 0.7789 | 0.9996 | beta 1,3-galactosyltransferase [Source:VB Community Annotation] |
| AAEL005006 | CYP6CD1 | 0.3245 | -1.7306 | 0.7790 | 0.9996 | cytochrome P450 [Source:VB Community Annotation] |
| AAEL003436 | | -0.1450 | 1.7135 | 0.7790 | 0.9996 | transcription factor coe3 [Source:VB Community Annotation] |
| AAEL023877 | NA | -0.3757 | -0.6204 | 0.7791 | 0.9996 | NA |
| AAEL027078 | NA | 0.4473 | -1.1945 | 0.7793 | 0.9996 | NA |
| AAEL017543 | | 0.0977 | 4.1432 | 0.7794 | 0.9996 | |
| AAEL012945 | | -0.0543 | 6.0150 | 0.7794 | 0.9996 | |
| AAEL003756 | | 0.1496 | 2.2548 | 0.7798 | 0.9996 | |
| AAEL002135 | | 0.0888 | 4.8309 | 0.7799 | 0.9996 | tubulin-specific chaperone b (tubulin folding cofactor b) [Source:VB Community Annotatioı |
| AAEL018302 | | 0.1316 | 3.5866 | 0.7799 | 0.9996 | |
| AAEL019450 | NA | 0.4030 | 1.0056 | 0.7799 | 0.9996 | NA |
| AAEL025170 | NA | -0.3238 | -0.5873 | 0.7799 | 0.9996 | NA |
| AAEL014564 | | 0.1317 | 3.6779 | 0.7802 | 0.9996 | |
| AAEL001795 | | -0.0818 | 5.9467 | 0.7802 | 0.9996 | orfY, putative [Source:VB Community Annotation] |
| AAEL002994 | | 0.2092 | 0.7860 | 0.7802 | 0.9996 | |
| AAEL010637 | | 0.0909 | 2.3876 | 0.7803 | 0.9996 | zinc finger protein, putative [Source:VB Community Annotation] |
| AAEL011084 | | 0.1283 | 4.9564 | 0.7803 | 0.9996 | |
| AAEL008578 | | 0.1844 | 2.4959 | 0.7803 | 0.9996 | |
| AAEL007642 | TUBE | 0.1102 | 3.8606 | 0.7806 | 0.9996 | TOLL pathway signalling. [Source:VB Community Annotation] |
| AAEL003774 | | 0.0963 | 7.6247 | 0.7807 | 0.9996 | |
| AAEL024973 | NA | -0.0978 | 3.2523 | 0.7809 | 0.9996 | NA |
| AAEL010860 | | 0.3711 | 1.0785 | 0.7809 | 0.9996 | |
| AAEL006568 | | 0.3974 | 6.8314 | 0.7809 | 0.9996 | serine protease [Source:VB Community Annotation] |
| AAEL009993 | | -0.4761 | 6.4956 | 0.7809 | 0.9996 | |
| AAEL005808 | | 0.3332 | -0.4796 | 0.7810 | 0.9996 | alanyl aminopeptidase [Source:VB Community Annotation] |
| AAEL004097 | | 0.2833 | 5.2667 | 0.7810 | 0.9996 | enhancer of split protein, putative [Source:VB Community Annotation] |
| AAEL000543 | CTLMA11 | 0.1686 | 6.1730 | 0.7810 | 0.9996 | C-Type Lectin (CTLMA11) - mannose binding. [Source:VB Community Annotation] |

| | | | | | | |
|---|---|---|---|---|---|---|
| AAEL000148 | | 0.1079 | 1.6961 | 0.7811 | 0.9996 | |
| AAEL006808 | | -0.0676 | 5.1617 | 0.7811 | 0.9996 | |
| AAEL005817 | RpL26 | -0.0776 | 10.5239 | 0.7813 | 0.9996 | 60S ribosomal protein L26 [Source:VB Community Annotation] |
| AAEL013511 | | -0.3797 | 7.9280 | 0.7813 | 0.9996 | |
| AAEL024454 | NA | -0.3483 | 5.2745 | 0.7814 | 0.9996 | NA |
| AAEL022136 | NA | -0.0874 | 5.9484 | 0.7814 | 0.9996 | NA |
| AAEL011962 | | 0.0975 | 3.1104 | 0.7816 | 0.9996 | |
| AAEL001449 | | -0.1524 | 0.7026 | 0.7818 | 0.9996 | |
| AAEL027393 | NA | 0.4064 | 4.7749 | 0.7818 | 0.9996 | NA |
| AAEL000594 | | 0.0983 | 4.5887 | 0.7819 | 0.9996 | |
| AAEL022257 | NA | -0.4264 | -1.2905 | 0.7820 | 0.9996 | NA |
| AAEL010471 | ChAT | -0.4844 | 2.6845 | 0.7821 | 0.9996 | choline O-acetyltransferase [Source:VB Community Annotation] |
| AAEL019460 | NA | 0.0967 | 5.0825 | 0.7822 | 0.9996 | NA |
| AAEL001625 | | -0.1530 | 4.6509 | 0.7822 | 0.9996 | |
| AAEL010464 | | 0.0627 | 7.2003 | 0.7822 | 0.9996 | glutamate dehydrogenase [Source:VB Community Annotation] |
| AAEL007281 | | 0.4263 | 2.0963 | 0.7824 | 0.9996 | stretchin-mlck [Source:VB Community Annotation] |
| AAEL025645 | NA | 0.4588 | 0.0569 | 0.7825 | 0.9996 | NA |
| AAEL006647 | | -0.0839 | 6.2145 | 0.7826 | 0.9996 | dynein light chain, putative [Source:VB Community Annotation] |
| AAEL011259 | | 0.3425 | -2.6071 | 0.7826 | 0.9996 | phosphatidylethanolamine-binding protein [Source:VB Community Annotation] |
| AAEL025406 | NA | -0.3786 | -0.8605 | 0.7827 | 0.9996 | NA |
| AAEL002703 | | 0.0858 | 6.6456 | 0.7828 | 0.9996 | pyridoxamine 5'-phosphate oxidase [Source:VB Community Annotation] |
| AAEL022454 | NA | -0.2424 | 4.6003 | 0.7828 | 0.9996 | NA |
| AAEL007301 | | 0.0974 | 3.8240 | 0.7828 | 0.9996 | |
| AAEL003842 | | -0.2386 | 2.8935 | 0.7829 | 0.9996 | |
| AAEL019595 | NA | -0.6157 | 2.7068 | 0.7829 | 0.9996 | NA |
| AAEL012450 | | 0.1055 | 4.0792 | 0.7829 | 0.9996 | serine/threonine-protein kinase [Source:VB Community Annotation] |
| AAEL027096 | NA | -0.6417 | 1.0272 | 0.7830 | 0.9996 | NA |
| AAEL006667 | | 0.1371 | 6.4940 | 0.7830 | 0.9996 | phosphatidyltransferase [Source:VB Community Annotation] |
| AAEL018131 | | -0.0698 | 6.7618 | 0.7831 | 0.9996 | |
| AAEL013490 | | 0.0776 | 5.8656 | 0.7832 | 0.9996 | |
| AAEL001294 | | 0.1589 | 6.1656 | 0.7836 | 0.9996 | |
| AAEL012361 | | -0.1774 | 0.9433 | 0.7836 | 0.9996 | |
| AAEL022010 | NA | 0.0846 | 5.7852 | 0.7837 | 0.9996 | NA |
| AAEL019444 | NA | -0.3996 | 5.3598 | 0.7837 | 0.9996 | NA |
| AAEL006351 | | -0.5252 | 5.8574 | 0.7840 | 0.9996 | |
| AAEL000791 | | 0.1358 | 6.5981 | 0.7841 | 0.9996 | |
| AAEL025672 | NA | 0.1274 | 1.8028 | 0.7842 | 0.9996 | NA |
| AAEL005464 | | 0.1047 | 5.3014 | 0.7843 | 0.9996 | |
| AAEL004326 | | -0.2861 | 1.6558 | 0.7844 | 0.9996 | |
| AAEL019960 | NA | -0.5775 | -0.2842 | 0.7845 | 0.9996 | NA |

| | | | | | | |
|---|---|---|---|---|---|---|
| AAEL021930 | NA | 0.6674 | 4.3842 | 0.7846 | 0.9996 | NA |
| AAEL004236 | | -0.2039 | 5.6102 | 0.7847 | 0.9996 | arf6 guanine nucleotide exchange factor [Source:VB Community Annotation] |
| AAEL026478 | NA | -0.1063 | 3.4059 | 0.7848 | 0.9996 | NA |
| AAEL005218 | | 0.1784 | 2.0865 | 0.7849 | 0.9996 | |
| AAEL019743 | NA | -0.2773 | 3.2456 | 0.7849 | 0.9996 | NA |
| AAEL009733 | | -0.3150 | 3.6390 | 0.7849 | 0.9996 | |
| AAEL003829 | | 0.0761 | 6.0610 | 0.7850 | 0.9996 | dynamin [Source:VB Community Annotation] |
| AAEL007019 | | -0.0644 | 8.6771 | 0.7853 | 0.9996 | Phosphate transporter [Source:UniProtKB/TrEMBL;Acc:Q173U8] |
| AAEL002487 | | -0.0668 | 5.7043 | 0.7853 | 0.9996 | P53 regulated pa26 nuclear protein sestrin [Source:VB Community Annotation] |
| AAEL022397 | NA | 0.1001 | 3.2699 | 0.7854 | 0.9996 | NA |
| AAEL013462 | | 0.2680 | 1.0392 | 0.7854 | 0.9996 | |
| AAEL023699 | NA | -0.2627 | 0.0832 | 0.7858 | 0.9996 | NA |
| AAEL024064 | NA | 0.1347 | 1.8681 | 0.7859 | 0.9996 | NA |
| AAEL001754 | | 0.1288 | 6.0160 | 0.7861 | 0.9996 | |
| AAEL014840 | | -0.1068 | 3.4881 | 0.7861 | 0.9996 | short-chain dehydrogenase [Source:VB Community Annotation] |
| AAEL005744 | | -0.1143 | 6.2285 | 0.7862 | 0.9996 | DEAD box ATP-dependent RNA helicase [Source:VB Community Annotation] |
| AAEL011447 | RpL14 | -0.0990 | 11.5030 | 0.7863 | 0.9996 | 60S ribosomal protein L14 [Source:UniProtKB/TrEMBL;Acc:Q1HRP1] |
| AAEL000970 | | 0.3249 | 3.3144 | 0.7866 | 0.9996 | |
| AAEL014368 | | 0.0829 | 4.9931 | 0.7867 | 0.9996 | sap18 [Source:VB Community Annotation] |
| AAEL004881 | | -0.5827 | 1.5075 | 0.7868 | 0.9996 | adam [Source:VB Community Annotation] |
| AAEL002299 | | 0.0772 | 4.3865 | 0.7868 | 0.9996 | high affinity copper transporter, putative [Source:VB Community Annotation] |
| AAEL006752 | | -0.1468 | 2.1295 | 0.7869 | 0.9996 | Misexpression suppressor of ras, putative [Source:VB Community Annotation] |
| AAEL023112 | NA | 0.3503 | -2.2465 | 0.7869 | 0.9996 | NA |
| AAEL023658 | NA | -0.5490 | 1.9624 | 0.7869 | 0.9996 | NA |
| AAEL021677 | NA | -0.2865 | -0.6527 | 0.7869 | 0.9996 | NA |
| AAEL013275 | | 0.3141 | 4.6868 | 0.7870 | 0.9996 | importin beta-1 [Source:VB Community Annotation] |
| AAEL015404 | LYSC7B | -0.1765 | 6.5881 | 0.7870 | 0.9996 | C-Type Lysozyme (Lys-B). [Source:VB Community Annotation] |
| AAEL006844 | GPRTYR | -0.3646 | 1.2527 | 0.7871 | 0.9996 | GPCR Octopamine/Tyramine Family [Source:VB Community Annotation] |
| AAEL018322 | | 0.1235 | 2.0761 | 0.7871 | 0.9996 | |
| AAEL022948 | NA | -0.3461 | 0.5543 | 0.7871 | 0.9996 | NA |
| AAEL006496 | | -0.1213 | 2.4226 | 0.7873 | 0.9996 | steroid dehydrogenase [Source:VB Community Annotation] |
| AAEL019848 | NA | -0.2988 | 5.5937 | 0.7873 | 0.9996 | NA |
| AAEL004586 | | 0.0730 | 5.1007 | 0.7873 | 0.9996 | Histone deacetylase [Source:UniProtKB/TrEMBL;Acc:Q17CF0] |
| AAEL012031 | | 0.1371 | 2.6096 | 0.7874 | 0.9996 | |
| AAEL010334 | | -0.1495 | 3.2789 | 0.7875 | 0.9996 | |
| AAEL001709 | | -0.0927 | 4.5707 | 0.7877 | 0.9996 | |
| AAEL002057 | | 0.1262 | 2.6275 | 0.7879 | 0.9996 | |
| AAEL000987 | RpL8 | 0.0792 | 11.9563 | 0.7880 | 0.9996 | 60S ribosomal protein L8 [Source:VB Community Annotation] |
| AAEL002437 | | 0.0726 | 4.7723 | 0.7880 | 0.9996 | symplekin [Source:VB Community Annotation] |
| AAEL008602 | | -0.1749 | 1.3585 | 0.7880 | 0.9996 | |

| | | | | | | |
|---|---|---|---|---|---|---|
| AAEL001231 | | -0.2821 | 4.4983 | 0.7881 | 0.9996 | MIND-MELD/ADAM [Source:VB Community Annotation] |
| AAEL004200 | | 0.4145 | 3.6593 | 0.7881 | 0.9996 | DNA replication licensing factor MCM8 [Source:VB Community Annotation] |
| AAEL018178 | | 0.0937 | 2.8619 | 0.7881 | 0.9996 | Amine oxidase [Source:UniProtKB/TrEMBL;Acc:A0A1S4G756] |
| AAEL004918 | | 0.5015 | 2.7705 | 0.7881 | 0.9996 | brat protein [Source:VB Community Annotation] |
| AAEL013222 | | 0.1306 | 5.0620 | 0.7882 | 0.9996 | |
| AAEL020512 | NA | 0.4318 | 10.5152 | 0.7884 | 0.9996 | NA |
| AAEL012520 | | 0.1415 | 1.6956 | 0.7884 | 0.9996 | Nop2p, putative [Source:VB Community Annotation] |
| AAEL010266 | | -0.3181 | -1.4223 | 0.7885 | 0.9996 | |
| AAEL012823 | | -0.3328 | 2.8088 | 0.7887 | 0.9996 | n-acetylgalactosaminyltransferase [Source:VB Community Annotation] |
| AAEL003742 | | -0.0894 | 3.0199 | 0.7887 | 0.9996 | Glucosylceramidase [Source:UniProtKB/TrEMBL;Acc:Q0IG10] |
| AAEL006061 | Prosalpha2 | 0.0755 | 6.9016 | 0.7888 | 0.9996 | 26S proteasome alpha 2 subunit [Source:VB Community Annotation] |
| AAEL023047 | NA | 0.2620 | 5.4601 | 0.7888 | 0.9996 | NA |
| AAEL019476 | NA | 0.1039 | 3.0567 | 0.7888 | 0.9996 | NA |
| AAEL023077 | NA | 0.0819 | 5.4567 | 0.7889 | 0.9996 | NA |
| AAEL007289 | | -0.1464 | 3.1463 | 0.7889 | 0.9996 | NBP2b protein, putative [Source:VB Community Annotation] |
| AAEL002304 | | -0.0974 | 6.0194 | 0.7889 | 0.9996 | porphobilinogen synthase [Source:VB Community Annotation] |
| AAEL011910 | | -0.2985 | 2.9692 | 0.7890 | 0.9996 | |
| AAEL009828 | | 0.0860 | 6.3122 | 0.7890 | 0.9996 | |
| AAEL001565 | | 0.1263 | 4.6206 | 0.7891 | 0.9996 | peptidyl-glycine alpha-amidating monooxygenase [Source:VB Community Annotation] |
| AAEL014531 | | 0.0984 | 4.3129 | 0.7892 | 0.9996 | arsenite inducuble RNA associated protein aip-1 [Source:VB Community Annotation] |
| AAEL002817 | | -0.5287 | 3.1359 | 0.7892 | 0.9996 | |
| AAEL027823 | NA | 0.3172 | -0.6324 | 0.7892 | 0.9996 | NA |
| AAEL007662 | | 0.1231 | 7.6904 | 0.7894 | 0.9996 | casein kinase [Source:VB Community Annotation] |
| AAEL005368 | | 0.0812 | 3.5280 | 0.7894 | 0.9996 | |
| AAEL027623 | NA | -0.2986 | -0.4142 | 0.7896 | 0.9996 | NA |
| AAEL020519 | NA | -0.4052 | -1.1277 | 0.7898 | 0.9996 | NA |
| AAEL012238 | | 0.0748 | 5.3904 | 0.7899 | 0.9996 | glutaredoxin, putative [Source:VB Community Annotation] |
| AAEL020823 | NA | -0.1637 | 8.1149 | 0.7902 | 0.9996 | NA |
| AAEL008638 | CYP49A1 | 0.1402 | 4.0531 | 0.7904 | 0.9996 | cytochrome P450 [Source:VB Community Annotation] |
| AAEL014031 | | 0.0622 | 5.4936 | 0.7904 | 0.9996 | protein phosphatase 2a, regulatory subunit [Source:VB Community Annotation] |
| AAEL019542 | NA | 0.2558 | 2.7281 | 0.7905 | 0.9996 | NA |
| AAEL004416 | | 0.1725 | 1.1940 | 0.7906 | 0.9996 | histone deacetylase [Source:VB Community Annotation] |
| AAEL027323 | NA | 0.3861 | -0.8757 | 0.7907 | 0.9996 | NA |
| AAEL011798 | | 0.2567 | 3.3267 | 0.7907 | 0.9996 | allergen, putative [Source:VB Community Annotation] |
| AAEL002606 | OBP35 | -0.1901 | 9.9541 | 0.7908 | 0.9996 | odorant binding protein OBP35 [Source:VB Community Annotation] |
| AAEL003141 | | -0.0957 | 2.5629 | 0.7908 | 0.9996 | |
| AAEL010756 | RpS19 | 0.0980 | 11.2460 | 0.7910 | 0.9996 | 40S ribosomal protein S19 [Source:UniProtKB/TrEMBL;Acc:Q1HRR8] |
| AAEL009278 | | 0.1242 | 7.1525 | 0.7910 | 0.9996 | Mitochondrial pyruvate carrier [Source:UniProtKB/TrEMBL;Acc:Q16WA8] |
| AAEL007863 | | -0.0746 | 4.9830 | 0.7910 | 0.9996 | |
| AAEL023745 | NA | -0.4165 | -1.5210 | 0.7911 | 0.9996 | NA |

| | | | | | | |
|---|---|---|---|---|---|---|
| AAEL023854 | NA | -0.2123 | 7.1963 | 0.7911 | 0.9996 | NA |
| AAEL002728 | | -0.1073 | 5.3046 | 0.7912 | 0.9996 | |
| AAEL001401 | LRIM10A | -0.1809 | 7.5604 | 0.7913 | 0.9996 | leucine-rich immune protein (Short) [Source:VB Community Annotation] |
| AAEL012176 | | 0.1177 | 2.9267 | 0.7915 | 0.9996 | |
| AAEL000354 | | -0.3512 | -0.9007 | 0.7915 | 0.9996 | dimeric dihydrodiol dehydrogenase [Source:VB Community Annotation] |
| AAEL013998 | | 0.3646 | -1.4375 | 0.7916 | 0.9996 | General transcription factor IIF subunit 2 [Source:UniProtKB/TrEMBL;Acc:Q16HK5] |
| AAEL006725 | | 0.1081 | 5.6987 | 0.7917 | 0.9996 | U2 small nuclear ribonucleoprotein, putative [Source:VB Community Annotation] |
| AAEL006450 | | -0.0781 | 4.7884 | 0.7918 | 0.9996 | integral membrane protein, putative [Source:VB Community Annotation] |
| AAEL008879 | kh | -0.1003 | 5.6704 | 0.7919 | 0.9996 | Kynurenine 3-monooxygenase (EC 1.14.13.9)(Kynurenine 3-hydroxylase) [Source:VB Community Annotation] |
| AAEL013489 | | -0.3964 | 0.7325 | 0.7919 | 0.9996 | sugar transporter [Source:VB Community Annotation] |
| AAEL005408 | | 0.0680 | 5.5998 | 0.7919 | 0.9996 | annexin x [Source:VB Community Annotation] |
| AAEL020195 | NA | -0.1934 | 0.4028 | 0.7921 | 0.9996 | NA |
| AAEL012510 | IKK2 | -0.3852 | 0.8479 | 0.7921 | 0.9996 | IMD pathway signalling I-Kappa-B Kinase 2 (IKK2 IKK-gamma). [Source:VB Community Annotation] |
| AAEL004574 | Sgf11 | 0.4228 | -0.6658 | 0.7922 | 0.9996 | SAGA-associated factor 11 homolog [Source:UniProtKB/Swiss-Prot;Acc:Q17CJ5] |
| AAEL011424 | | 0.0963 | 3.8289 | 0.7922 | 0.9996 | histone H3 [Source:VB Community Annotation] |
| AAEL015007 | | -0.0969 | 5.3770 | 0.7925 | 0.9996 | Proteasome subunit alpha type [Source:UniProtKB/TrEMBL;Acc:Q16EV7] |
| AAEL025547 | NA | -0.1301 | 3.0415 | 0.7925 | 0.9996 | NA |
| AAEL022500 | NA | -0.2086 | 0.8424 | 0.7927 | 0.9996 | NA |
| AAEL003117 | | 0.1314 | 4.7619 | 0.7927 | 0.9996 | peroxisomal targeting signal 2 receptor [Source:VB Community Annotation] |
| AAEL003183 | | 0.0970 | 4.4806 | 0.7927 | 0.9996 | oxidoreductase [Source:VB Community Annotation] |
| AAEL013309 | | 0.0865 | 6.0147 | 0.7929 | 0.9996 | high-affinity copper uptake protein [Source:VB Community Annotation] |
| AAEL022941 | NA | -0.0987 | 6.7036 | 0.7929 | 0.9996 | NA |
| AAEL007016 | | 0.2501 | 0.2449 | 0.7931 | 0.9996 | sodium/solute symporter [Source:VB Community Annotation] |
| AAEL005649 | | 0.4417 | -0.0957 | 0.7932 | 0.9996 | beat protein [Source:VB Community Annotation] |
| AAEL026920 | NA | 0.1444 | 3.2177 | 0.7934 | 0.9996 | NA |
| AAEL002591 | OBP13 | -0.2006 | 7.1871 | 0.7936 | 0.9996 | odorant binding protein OBP13 [Source:VB Community Annotation] |
| AAEL017931 | U1 | -0.1322 | 5.7217 | 0.7936 | 0.9996 | U1 spliceosomal RNA [Source:RFAM;Acc:RF00003] |
| AAEL024098 | NA | -0.0734 | 6.1210 | 0.7937 | 0.9996 | NA |
| AAEL023753 | NA | -0.1846 | 2.6617 | 0.7939 | 0.9996 | NA |
| AAEL025277 | NA | 0.1181 | 4.8453 | 0.7941 | 0.9996 | NA |
| AAEL003813 | | 0.1349 | 1.9869 | 0.7941 | 0.9996 | Mitochondrial GTPase 1 [Source:UniProtKB/Swiss-Prot;Acc:Q17EJ1] |
| AAEL006964 | | -0.1230 | 4.8146 | 0.7941 | 0.9996 | |
| AAEL011158 | | 0.0911 | 4.3173 | 0.7941 | 0.9996 | disulfide oxidoreductase [Source:VB Community Annotation] |
| AAEL009181 | | -0.1727 | 5.4937 | 0.7942 | 0.9996 | |
| AAEL006377 | LRIM31 | -0.1088 | 7.1770 | 0.7942 | 0.9996 | leucine-rich immune protein (Coil-less) [Source:VB Community Annotation] |
| AAEL023669 | NA | 0.4366 | 1.3586 | 0.7942 | 0.9996 | NA |
| AAEL021206 | NA | -0.1475 | 2.0566 | 0.7943 | 0.9996 | NA |
| AAEL011280 | | -0.2121 | 5.7459 | 0.7944 | 0.9996 | voltage-dependent p/q type calcium channel [Source:VB Community Annotation] |
| AAEL017674 | U5 | -0.2497 | 3.3006 | 0.7945 | 0.9996 | U5 spliceosomal RNA [Source:RFAM;Acc:RF00020] |
| AAEL002722 | | 0.0845 | 6.1720 | 0.7947 | 0.9996 | protein kinase C inhibitor, putative [Source:VB Community Annotation] |

| | | | | | | |
|---|---|---|---|---|---|---|
| AAEL013460 | | 0.4137 | 3.9197 | 0.7948 | 0.9996 | ecotropic viral integration site [Source:VB Community Annotation] |
| AAEL019422 | NA | -0.3550 | 4.7231 | 0.7948 | 0.9996 | NA |
| AAEL002526 | | -0.1040 | 9.0634 | 0.7949 | 0.9996 | MICOS complex subunit MIC10 [Source:UniProtKB/TrEMBL;Acc:Q17I05] |
| AAEL002733 | npf | 0.1131 | 4.1828 | 0.7949 | 0.9996 | neuropeptide F Precursor (NPF) [Source:VB Community Annotation] |
| AAEL006560 | | -0.0915 | 4.4194 | 0.7949 | 0.9996 | cop-coated vesicle membrane protein P24 [Source:VB Community Annotation] |
| AAEL022105 | NA | -0.1013 | 4.1264 | 0.7950 | 0.9996 | NA |
| AAEL020972 | NA | -0.3035 | 3.4412 | 0.7951 | 0.9996 | NA |
| AAEL007556 | | -0.0750 | 4.4428 | 0.7951 | 0.9996 | |
| AAEL003055 | | 0.0978 | 5.3230 | 0.7951 | 0.9996 | |
| AAEL024387 | NA | 0.1827 | 6.1624 | 0.7951 | 0.9996 | NA |
| AAEL026939 | NA | -0.2021 | 5.3848 | 0.7952 | 0.9996 | NA |
| AAEL008331 | | -0.3859 | -1.6437 | 0.7952 | 0.9996 | |
| AAEL019662 | NA | 0.2398 | 0.8339 | 0.7953 | 0.9996 | NA |
| AAEL008785 | | 0.1675 | 11.3539 | 0.7953 | 0.9996 | |
| AAEL027318 | NA | 0.1918 | 0.5963 | 0.7954 | 0.9996 | NA |
| AAEL007258 | | -0.1281 | 5.0488 | 0.7956 | 0.9996 | |
| AAEL020250 | NA | -0.3417 | -0.8529 | 0.7956 | 0.9996 | NA |
| AAEL004643 | mRpL1 | -0.1287 | 6.1498 | 0.7958 | 0.9996 | mitochondrial ribosomal protein L1 [Source:VB Community Annotation] |
| AAEL007317 | | -0.0930 | 2.8093 | 0.7958 | 0.9996 | |
| AAEL020221 | NA | 0.2123 | 7.7122 | 0.7959 | 0.9996 | NA |
| AAEL025268 | NA | 0.1816 | 5.6680 | 0.7961 | 0.9996 | NA |
| AAEL000180 | mRpL17 | -0.0967 | 6.0257 | 0.7962 | 0.9996 | mitochondrial ribosomal protein, L17 [Source:VB Community Annotation] |
| AAEL008137 | | -0.1913 | 0.5780 | 0.7963 | 0.9996 | |
| AAEL010097 | | -0.5260 | 0.2609 | 0.7964 | 0.9996 | |
| AAEL006254 | GlcAT-P | 0.0952 | 6.0704 | 0.7966 | 0.9996 | beta-1,3-glucuronyltransferase s, p [Source:VB Community Annotation] |
| AAEL002730 | SRPN21 | -0.1694 | 3.7884 | 0.7967 | 0.9996 | Serine Protease Inhibitor (serpin)  likely cleavage at R/V. [Source:VB Community Annotation] |
| AAEL020362 | NA | -0.2109 | 5.5290 | 0.7968 | 0.9996 | NA |
| AAEL004188 | | -0.5073 | 1.4889 | 0.7968 | 0.9996 | |
| AAEL025866 | NA | 0.3782 | -2.3525 | 0.7969 | 0.9996 | NA |
| AAEL023850 | NA | 0.0743 | 5.3025 | 0.7969 | 0.9996 | NA |
| AAEL001426 | | -0.0857 | 3.3370 | 0.7969 | 0.9996 | poly(a)-specific ribonuclease (deadenylation nuclease) [Source:VB Community Annotation] |
| AAEL006890 | | -0.2427 | 6.9630 | 0.7970 | 0.9996 | |
| AAEL003244 | | 0.3172 | -0.2069 | 0.7971 | 0.9996 | |
| AAEL028163 | NA | -0.1936 | 0.9486 | 0.7972 | 0.9996 | NA |
| AAEL004351 | | -0.1363 | 7.0062 | 0.7972 | 0.9996 | casein kinase [Source:VB Community Annotation] |
| AAEL024520 | NA | 0.1183 | 5.1557 | 0.7973 | 0.9996 | NA |
| AAEL018265 | CTL9 | 0.1941 | 0.9802 | 0.7973 | 0.9996 | C-Type lectin (CTL) [Source:VB Community Annotation] |
| AAEL022886 | NA | 0.1356 | 3.9443 | 0.7974 | 0.9996 | NA |
| AAEL017395 | | -0.0624 | 6.2452 | 0.7975 | 0.9996 | |
| AAEL027380 | NA | -0.1437 | 1.6179 | 0.7975 | 0.9996 | NA |

| | | | | | | |
|---|---|---|---|---|---|---|
| AAEL005032 | | -0.1798 | 8.1092 | 0.7975 | 0.9996 | |
| AAEL020351 | NA | -0.5114 | 0.7374 | 0.7976 | 0.9996 | NA |
| AAEL005028 | | 0.0957 | 3.0331 | 0.7978 | 0.9996 | |
| AAEL000706 | | 0.1098 | 4.4878 | 0.7978 | 0.9996 | serine palmitoyltransferase [Source:VB Community Annotation] |
| AAEL007246 | | 0.1011 | 3.4695 | 0.7979 | 0.9996 | |
| AAEL012943 | | 0.0674 | 6.8460 | 0.7979 | 0.9996 | 26S protease regulatory subunit [Source:VB Community Annotation] |
| AAEL007970 | | -0.1775 | 1.3505 | 0.7980 | 0.9996 | |
| AAEL009189 | | -0.3069 | 5.2360 | 0.7980 | 0.9996 | encore protein [Source:VB Community Annotation] |
| AAEL024751 | NA | -0.1687 | 1.3234 | 0.7980 | 0.9996 | NA |
| AAEL009041 | | -0.0667 | 5.1214 | 0.7982 | 0.9996 | arp2/3 complex 20 kd subunit [Source:VB Community Annotation] |
| AAEL004245 | GPRNNA1[illegible] | -0.3897 | -1.1361 | 0.7982 | 0.9996 | GPCR Orphan/Putative Class A Family [Source:VB Community Annotation] |
| AAEL003777 | | -0.0675 | 5.8850 | 0.7982 | 0.9996 | |
| AAEL004292 | | 0.9461 | 2.3819 | 0.7983 | 0.9996 | |
| AAEL000898 | | 0.2894 | 0.6090 | 0.7984 | 0.9996 | Carboxylic ester hydrolase [Source:UniProtKB/TrEMBL;Acc:A0A1S4EX69] |
| AAEL005749 | | -0.1121 | 5.3046 | 0.7984 | 0.9996 | lysosomal alpha-mannosidase (mannosidase alpha class 2b member 1) [Source:VB Community Annotation] |
| AAEL013959 | | -0.0738 | 5.2000 | 0.7984 | 0.9996 | |
| AAEL017101 | | -0.0663 | 3.8331 | 0.7985 | 0.9996 | Ribonuclease H1 [Source:UniProtKB/TrEMBL;Acc:J9HZ50 |
| AAEL007357 | | 0.0782 | 3.4916 | 0.7985 | 0.9996 | |
| AAEL000049 | | -0.2468 | 2.3267 | 0.7986 | 0.9996 | three prime repair exonuclease 1, putative [Source:VB Community Annotation] |
| AAEL014576 | | 0.1130 | 4.5873 | 0.7987 | 0.9996 | |
| AAEL027400 | NA | 0.4385 | -0.5977 | 0.7987 | 0.9996 | NA |
| AAEL023659 | NA | -0.1445 | 1.5441 | 0.7989 | 0.9996 | NA |
| AAEL000947 | spase22-2[illegible] | -0.0943 | 5.5375 | 0.7990 | 0.9996 | microsomal signal peptidase 23 kd subunit (spc22/23) [Source:VB Community Annotation] |
| AAEL010316 | | 0.0627 | 5.9012 | 0.7991 | 0.9996 | |
| AAEL014554 | | -0.2011 | 0.7957 | 0.7992 | 0.9996 | |
| AAEL024525 | NA | -0.1368 | 2.6378 | 0.7992 | 0.9996 | NA |
| AAEL006832 | GPRFZ3 | 0.1955 | 1.1864 | 0.7993 | 0.9996 | GPCR Frizzled/Smoothened Family [Source:VB Community Annotation] |
| AAEL014844 | | 0.1770 | 0.8105 | 0.7995 | 0.9996 | |
| AAEL006314 | | 0.2364 | 3.7252 | 0.7995 | 0.9996 | upstream transcription factor [Source:VB Community Annotation] |
| AAEL011251 | | -0.1476 | 1.7765 | 0.7995 | 0.9996 | RNA binding motif protein [Source:VB Community Annotation] |
| AAEL027449 | NA | 0.2728 | 0.5985 | 0.7996 | 0.9996 | NA |
| AAEL005221 | | 0.1078 | 7.5781 | 0.7997 | 0.9996 | internalin A, putative [Source:VB Community Annotation] |
| AAEL000822 | | 0.0850 | 4.8333 | 0.7998 | 0.9996 | kinesin-like protein KLP68D [Source:VB Community Annotation] |
| AAEL004479 | | -0.5053 | -0.8218 | 0.7998 | 0.9996 | organic cation transporter [Source:VB Community Annotation] |
| AAEL000672 | | -0.0894 | 3.6431 | 0.7999 | 0.9996 | cyclin a [Source:VB Community Annotation] |
| AAEL023480 | NA | -0.3157 | -1.2230 | 0.7999 | 0.9996 | NA |
| AAEL012007 | | -0.4048 | -0.3612 | 0.8000 | 0.9996 | |
| AAEL004718 | | -0.1750 | 2.2557 | 0.8000 | 0.9996 | |
| AAEL020819 | NA | -0.1358 | 3.0285 | 0.8001 | 0.9996 | NA |
| AAEL009114 | | -0.3786 | 2.5073 | 0.8002 | 0.9996 | |

| | | | | | | |
|---|---|---|---|---|---|---|
| AAEL011391 | mRpS9 | -0.0748 | 5.7594 | 0.8003 | 0.9996 | mitochondrial ribosomal protein, S9, putative [Source:VB Community Annotation] |
| AAEL003135 | | 0.1089 | 4.0526 | 0.8004 | 0.9996 | |
| AAEL028185 | NA | 0.0822 | 5.6671 | 0.8004 | 0.9996 | NA |
| AAEL004343 | OBP19 | 0.2779 | 5.7898 | 0.8004 | 0.9996 | odorant binding protein OBP19 [Source:VB Community Annotation] |
| AAEL009725 | | -0.1934 | 1.7061 | 0.8005 | 0.9996 | |
| AAEL007319 | | 0.2207 | 2.1412 | 0.8005 | 0.9996 | |
| AAEL009627 | | -0.1326 | 8.3100 | 0.8005 | 0.9996 | |
| AAEL022221 | NA | 0.4311 | 1.9013 | 0.8006 | 0.9996 | NA |
| AAEL003506 | | 0.0863 | 3.8448 | 0.8007 | 0.9996 | emx homeobox protein [Source:VB Community Annotation] |
| AAEL010300 | | -0.1007 | 4.5000 | 0.8007 | 0.9996 | |
| AAEL007363 | | 0.0835 | 4.3817 | 0.8008 | 0.9996 | leucine-rich transmembrane protein [Source:VB Community Annotation] |
| AAEL010341 | rept | 0.0950 | 4.0148 | 0.8009 | 0.9996 | RuvB-like helicase 2 (EC 3.6.4.12)(Reptin) [Source:VB Community Annotation] |
| AAEL013984 | | -0.1306 | 6.1617 | 0.8009 | 0.9996 | |
| AAEL013845 | | -0.0548 | 5.5905 | 0.8010 | 0.9996 | endoplasmic reticulum resident protein (ERp44), putative [Source:VB Community Annotation] |
| AAEL008756 | | -0.1319 | 3.0897 | 0.8011 | 0.9996 | |
| AAEL001940 | | 0.2963 | 3.2942 | 0.8011 | 0.9996 | Phosphatidate cytidylyltransferase [Source:UniProtKB/TrEMBL;Acc:Q17JP6] |
| AAEL001843 | | -0.3092 | 4.5641 | 0.8012 | 0.9996 | ski oncogene [Source:VB Community Annotation] |
| AAEL002375 | | -0.0854 | 5.3575 | 0.8012 | 0.9996 | NBP2b protein, putative [Source:VB Community Annotation] |
| AAEL005211 | | 0.0667 | 6.0875 | 0.8012 | 0.9996 | microtubule associated-protein orbit [Source:VB Community Annotation] |
| AAEL013252 | | 0.0770 | 7.0687 | 0.8013 | 0.9996 | |
| AAEL011019 | | 0.0744 | 6.1227 | 0.8013 | 0.9996 | dynein light intermediate chain [Source:VB Community Annotation] |
| AAEL020340 | NA | 0.1340 | 7.6143 | 0.8014 | 0.9996 | NA |
| AAEL000271 | | -0.1077 | 6.0750 | 0.8015 | 0.9996 | gamma-glutamyl hydrolase [Source:VB Community Annotation] |
| AAEL019778 | NA | 0.3926 | -0.9202 | 0.8016 | 0.9996 | NA |
| AAEL002308 | | -0.2052 | 6.5046 | 0.8016 | 0.9996 | butyrate response factor 1 (TIS11B protein) [Source:VB Community Annotation] |
| AAEL000823 | RpL35 | -0.0673 | 10.8230 | 0.8016 | 0.9996 | 60S ribosomal protein L35A, putative [Source:VB Community Annotation] |
| AAEL007780 | | -0.3550 | 5.9859 | 0.8017 | 0.9996 | |
| AAEL001628 | | -0.1014 | 2.1377 | 0.8017 | 0.9996 | |
| AAEL008533 | | 0.1052 | 2.0257 | 0.8018 | 0.9996 | |
| AAEL015326 | | 0.0958 | 6.9648 | 0.8018 | 0.9996 | lipase 1 precursor [Source:VB Community Annotation] |
| AAEL007226 | | -0.2868 | 3.0822 | 0.8019 | 0.9996 | nidogen [Source:VB Community Annotation] |
| AAEL021239 | NA | -0.1082 | 2.9208 | 0.8020 | 0.9996 | NA |
| AAEL005413 | mRpS11 | -0.0973 | 6.2517 | 0.8021 | 0.9996 | mitochondrial ribosomal protein, S11, putative [Source:VB Community Annotation] |
| AAEL001803 | | -0.3435 | -0.7794 | 0.8022 | 0.9996 | |
| AAEL003067 | | 0.1179 | 8.4719 | 0.8022 | 0.9996 | |
| AAEL001920 | | 0.1927 | 3.4800 | 0.8022 | 0.9996 | |
| AAEL006520 | | 0.0586 | 5.4606 | 0.8022 | 0.9996 | |
| AAEL003287 | Ars2 | 0.0854 | 4.7987 | 0.8023 | 0.9996 | Serrate RNA effector molecule homolog (Arsenite-resistance protein 2 homolog) [Source:VB Community Annotation] |
| AAEL021151 | NA | -0.1280 | 1.4327 | 0.8023 | 0.9996 | NA |
| AAEL021284 | NA | 0.0690 | 5.2989 | 0.8024 | 0.9996 | NA |

| | | | | | | |
|---|---|---|---|---|---|---|
| AAEL000290 | | 0.0750 | 3.3574 | 0.8024 | 0.9996 | |
| AAEL005977 | | 0.0697 | 5.1587 | 0.8024 | 0.9996 | chondroitin 4-sulfotransferase [Source:VB Community Annotation] |
| AAEL003383 | | -0.0848 | 5.1740 | 0.8026 | 0.9996 | actin [Source:VB Community Annotation] |
| AAEL020936 | NA | 0.0844 | 5.9273 | 0.8026 | 0.9996 | NA |
| AAEL002153 | | 0.1589 | 1.5101 | 0.8027 | 0.9996 | |
| AAEL018025 | | -0.1467 | 3.3394 | 0.8028 | 0.9996 | |
| AAEL009590 | | -0.1173 | 7.8867 | 0.8030 | 0.9996 | outer mitochondrial translocase subunit, putative [Source:VB Community Annotation] |
| AAEL009266 | | -0.2542 | 4.1222 | 0.8031 | 0.9996 | c4b-binding protein beta chain [Source:VB Community Annotation] |
| AAEL003391 | | -0.3037 | -0.6159 | 0.8031 | 0.9996 | tankyrase [Source:VB Community Annotation] |
| AAEL021107 | NA | 0.2698 | 1.2621 | 0.8031 | 0.9996 | NA |
| AAEL008807 | | 0.1040 | 2.9445 | 0.8032 | 0.9996 | inositol 5-phosphatase [Source:VB Community Annotation] |
| AAEL025903 | NA | -0.3262 | 0.6974 | 0.8033 | 0.9996 | NA |
| AAEL020192 | NA | -0.2602 | 5.3703 | 0.8034 | 0.9996 | NA |
| AAEL024153 | NA | -0.3459 | 4.2264 | 0.8034 | 0.9996 | NA |
| AAEL010789 | | -0.0616 | 5.9565 | 0.8034 | 0.9996 | |
| AAEL001847 | | 0.0897 | 6.2056 | 0.8035 | 0.9996 | DNA-directed RNA polymerase [Source:UniProtKB/TrEMBL;Acc:Q17K00] |
| AAEL004056 | | -0.0642 | 5.4362 | 0.8036 | 0.9996 | |
| AAEL001817 | | -0.1195 | 2.1337 | 0.8037 | 0.9996 | |
| AAEL014382 | CTLMA14 | -0.2080 | 5.9669 | 0.8037 | 0.9996 | C-Type Lectin (CTL) - mannose binding. [Source:VB Community Annotation] |
| AAEL003953 | | -0.1277 | 6.6032 | 0.8038 | 0.9996 | map/microtubule affinity-regulating kinase 2,4 [Source:VB Community Annotation] |
| AAEL008452 | | -0.1215 | 4.3897 | 0.8038 | 0.9996 | Alpha-amylase [Source:UniProtKB/TrEMBL;Acc:Q16YR0] |
| AAEL028996 | NA | 0.6067 | -0.3323 | 0.8038 | 0.9996 | NA |
| AAEL005768 | | -0.0931 | 10.9349 | 0.8039 | 0.9996 | |
| AAEL001950 | | 0.1408 | 3.5645 | 0.8039 | 0.9996 | |
| AAEL010540 | | -0.2324 | 0.3058 | 0.8039 | 0.9996 | alpha-amylase [Source:VB Community Annotation] |
| AAEL020457 | NA | 0.0682 | 4.9312 | 0.8040 | 0.9996 | NA |
| AAEL011939 | | -0.0962 | 4.2513 | 0.8040 | 0.9996 | |
| AAEL007943 | | -0.0754 | 3.7835 | 0.8041 | 0.9996 | |
| AAEL009043 | | -0.2604 | 3.4999 | 0.8041 | 0.9996 | |
| AAEL023471 | NA | -0.1346 | 2.9163 | 0.8042 | 0.9996 | NA |
| AAEL011471 | RpL17 | 0.0805 | 11.9295 | 0.8044 | 0.9996 | 60S ribosomal protein L17 [Source:UniProtKB/Swiss-Prot;Acc:Q1HR65] |
| AAEL013027 | | 0.4751 | -1.6015 | 0.8046 | 0.9996 | |
| AAEL005718 | CLIPA3 | 0.1104 | 6.0592 | 0.8046 | 0.9996 | Clip-Domain Serine Protease family A. Protease homologue [Source:VB Community Annotation] |
| AAEL008063 | | -0.1916 | 4.9007 | 0.8046 | 0.9996 | |
| AAEL001171 | | -0.0677 | 4.3782 | 0.8048 | 0.9996 | tRNA-dihydrouridine synthase [Source:VB Community Annotation] |
| AAEL004992 | | 0.4177 | 1.5864 | 0.8049 | 0.9996 | lachesin, putative [Source:VB Community Annotation] |
| AAEL005583 | | 0.0646 | 6.4224 | 0.8049 | 0.9996 | |
| AAEL011234 | | -0.0956 | 5.2552 | 0.8052 | 0.9996 | reticulon/nogo receptor [Source:VB Community Annotation] |
| AAEL003806 | | -0.2809 | 4.4538 | 0.8052 | 0.9996 | |
| AAEL018154 | | -0.1732 | 2.2514 | 0.8053 | 0.9996 | |

| | | | | | | |
|---|---|---|---|---|---|---|
| AAEL008588 | inx7 | 0.2997 | -1.2306 | 0.8053 | 0.9996 | Innexin [Source:UniProtKB/TrEMBL;Acc:Q16YE3] |
| AAEL014064 | | 0.0883 | 7.6081 | 0.8057 | 0.9996 | glutaredoxin, putative [Source:VB Community Annotation] |
| AAEL012624 | | 0.0896 | 4.3115 | 0.8057 | 0.9996 | |
| AAEL009280 | | -0.2597 | -0.2042 | 0.8057 | 0.9996 | |
| AAEL006248 | | 0.1863 | 4.3123 | 0.8059 | 0.9996 | p37NB protein, putative [Source:VB Community Annotation] |
| AAEL001493 | | -0.1742 | 1.7478 | 0.8059 | 0.9996 | |
| AAEL014909 | | -0.1214 | 6.9244 | 0.8061 | 0.9996 | |
| AAEL014089 | | 0.2176 | 0.4051 | 0.8061 | 0.9996 | ionotropic glutamate receptor-invertebrate [Source:VB Community Annotation] |
| AAEL026040 | NA | -0.0762 | 4.0359 | 0.8062 | 0.9996 | NA |
| AAEL011506 | | 0.0703 | 3.8942 | 0.8063 | 0.9996 | |
| AAEL022415 | NA | 0.1431 | 1.7814 | 0.8063 | 0.9996 | NA |
| AAEL003524 | | 0.0751 | 5.4767 | 0.8063 | 0.9996 | monocarboxylate transporter [Source:VB Community Annotation] |
| AAEL002341 | | -0.1744 | 0.7414 | 0.8063 | 0.9996 | |
| AAEL009487 | | 0.2106 | 2.9289 | 0.8065 | 0.9996 | |
| AAEL014641 | | -0.0914 | 3.2268 | 0.8065 | 0.9996 | |
| AAEL000692 | | 0.0930 | 2.7964 | 0.8066 | 0.9996 | partner of sld5 [Source:VB Community Annotation] |
| AAEL005992 | | -0.1407 | 2.0603 | 0.8067 | 0.9996 | adam (a disintegrin and metalloprotease) [Source:VB Community Annotation] |
| AAEL010798 | | 0.0895 | 6.8529 | 0.8068 | 0.9996 | ubiquitin-conjugating enzyme E2 g [Source:VB Community Annotation] |
| AAEL012702 | | 0.3058 | 2.0206 | 0.8068 | 0.9996 | ATP-binding cassette sub-family A member 3, putative [Source:VB Community Annotation] |
| AAEL009759 | | -0.0767 | 3.3636 | 0.8069 | 0.9996 | |
| AAEL009026 | | 0.0792 | 5.7575 | 0.8069 | 0.9996 | ubiquitin-conjugating enzyme m [Source:VB Community Annotation] |
| AAEL013816 | | 0.0769 | 3.6917 | 0.8070 | 0.9996 | |
| AAEL011371 | | -0.4873 | 2.8789 | 0.8070 | 0.9996 | |
| AAEL001888 | | -0.0668 | 5.6182 | 0.8071 | 0.9996 | |
| AAEL008096 | | -0.1505 | 4.3001 | 0.8071 | 0.9996 | mitochondrial ornithine transporter [Source:VB Community Annotation] |
| AAEL002668 | | 0.1233 | 3.6226 | 0.8072 | 0.9996 | AMP dependent ligase [Source:VB Community Annotation] |
| AAEL019458 | NA | -0.3046 | 6.1253 | 0.8073 | 0.9996 | NA |
| AAEL023481 | NA | -0.0836 | 7.9042 | 0.8073 | 0.9996 | NA |
| AAEL000433 | | -0.2914 | -0.0292 | 0.8074 | 0.9996 | glypican [Source:VB Community Annotation] |
| AAEL006235 | | -0.1211 | 3.4306 | 0.8074 | 0.9996 | |
| AAEL021388 | NA | -0.1303 | 0.8429 | 0.8075 | 0.9996 | NA |
| AAEL007378 | | 0.3805 | -0.2032 | 0.8075 | 0.9996 | |
| AAEL000140 | | 0.0840 | 3.8243 | 0.8075 | 0.9996 | |
| AAEL020127 | NA | -0.1119 | 5.4037 | 0.8076 | 0.9996 | NA |
| AAEL026488 | NA | 0.0889 | 3.5876 | 0.8077 | 0.9996 | NA |
| AAEL014002 | | 0.1489 | 2.0568 | 0.8078 | 0.9996 | |
| AAEL012083 | | 0.0716 | 4.9251 | 0.8079 | 0.9996 | receptor protein-tyrosine phosphatase 10d [Source:VB Community Annotation] |
| AAEL024171 | NA | 0.3118 | -1.1846 | 0.8080 | 0.9996 | NA |
| AAEL015092 | | 0.2457 | 2.0445 | 0.8082 | 0.9996 | |
| AAEL003516 | | -0.1969 | -0.1018 | 0.8084 | 0.9996 | |

| | | | | | | |
|---|---|---|---|---|---|---|
| AAEL023475 | NA | -0.1070 | 2.6528 | 0.8084 | 0.9996 | NA |
| AAEL005026 | | 0.1653 | 0.8726 | 0.8084 | 0.9996 | ATP-dependent bile acid permease [Source:VB Community Annotation] |
| AAEL002583 | TOLL7 | -0.2366 | 4.5973 | 0.8084 | 0.9996 | Toll-like receptor [Source:VB Community Annotation] |
| AAEL019493 | NA | -0.1493 | 2.4595 | 0.8085 | 0.9996 | NA |
| AAEL004120 | | -0.2275 | 7.4928 | 0.8085 | 0.9996 | Niemann-Pick Type C-2, putative [Source:VB Community Annotation] |
| AAEL010511 | | -0.0820 | 4.1510 | 0.8086 | 0.9996 | |
| AAEL007303 | | 0.0659 | 3.6581 | 0.8087 | 0.9996 | |
| AAEL018151 | | -0.0799 | 4.6841 | 0.8088 | 0.9996 | |
| AAEL012932 | | -0.1186 | 3.3780 | 0.8088 | 0.9996 | |
| AAEL006453 | | 0.1266 | 2.2058 | 0.8088 | 0.9996 | B-cell lymphoma/leukaemia 11A extra long form, putative [Source:VB Community Annotation] |
| AAEL019823 | NA | 0.0679 | 7.4896 | 0.8089 | 0.9996 | NA |
| AAEL002848 | | 0.1906 | 2.7266 | 0.8089 | 0.9996 | Tubulin beta chain (Fragment) [Source:UniProtKB/TrEMBL;Acc:Q17GX8] |
| AAEL008765 | | 0.3941 | 0.8899 | 0.8089 | 0.9996 | cuticle protein, putative [Source:VB Community Annotation] |
| AAEL002723 | | 0.0981 | 4.1253 | 0.8089 | 0.9996 | peroxisomal membrane protein pmp34 [Source:VB Community Annotation] |
| AAEL004446 | | -0.2141 | 1.1056 | 0.8090 | 0.9996 | |
| AAEL004237 | | 0.0627 | 4.3188 | 0.8090 | 0.9996 | vacuolar protein sorting 18 (deep orange protein) [Source:VB Community Annotation] |
| AAEL008422 | | -0.2641 | 4.1725 | 0.8092 | 0.9996 | |
| AAEL014600 | | 0.2012 | 7.6505 | 0.8093 | 0.9996 | 4-hydroxyphenylpyruvate dioxygenase [Source:UniProtKB/TrEMBL;Acc:Q16FX9 |
| AAEL003563 | | -0.1207 | 1.8141 | 0.8094 | 0.9996 | |
| AAEL004699 | | -0.4258 | 1.1449 | 0.8094 | 0.9996 | |
| AAEL025785 | NA | -0.5561 | 1.7162 | 0.8095 | 0.9996 | NA |
| AAEL011664 | | -0.1414 | 2.1871 | 0.8095 | 0.9996 | |
| AAEL005049 | | -0.2128 | 1.1866 | 0.8095 | 0.9996 | heterogeneous nuclear ribonucleoprotein [Source:VB Community Annotation] |
| AAEL009291 | | -0.0851 | 8.9812 | 0.8096 | 0.9996 | retinoid-inducible serine carboxypeptidase (serine carboxypeptidase [Source:VB Community Annotation] |
| AAEL003622 | | 0.1567 | 4.3892 | 0.8096 | 0.9996 | delta(9)-desaturase, putative [Source:VB Community Annotation] |
| AAEL008590 | | -0.0826 | 4.9193 | 0.8097 | 0.9996 | |
| AAEL006790 | | -0.0980 | 3.4239 | 0.8098 | 0.9996 | |
| AAEL007125 | | 0.0831 | 4.1006 | 0.8099 | 0.9996 | PET117 polypeptide, putative [Source:VB Community Annotation] |
| AAEL008278 | | -0.3706 | 0.2672 | 0.8101 | 0.9996 | |
| AAEL023434 | NA | 0.3350 | -0.1704 | 0.8103 | 0.9996 | NA |
| AAEL019710 | NA | 0.3101 | 4.8222 | 0.8103 | 0.9996 | NA |
| AAEL014406 | | -0.0771 | 4.1701 | 0.8104 | 0.9996 | |
| AAEL007403 | | -0.1330 | 2.5344 | 0.8105 | 0.9996 | |
| AAEL007404 | | 0.0566 | 3.9121 | 0.8106 | 0.9996 | |
| AAEL001374 | | 0.1411 | 2.9363 | 0.8107 | 0.9996 | flagellar radial spoke protein [Source:VB Community Annotation] |
| AAEL001029 | | 0.0711 | 5.0324 | 0.8107 | 0.9996 | |
| AAEL000793 | | -0.4362 | 9.3686 | 0.8109 | 0.9996 | venom allergen [Source:VB Community Annotation] |
| AAEL009833 | mRpL46 | -0.0711 | 4.4684 | 0.8109 | 0.9996 | mitochondrial ribosomal protein, L46, putative [Source:VB Community Annotation] |
| AAEL017098 | | -0.0957 | 6.5000 | 0.8110 | 0.9996 | |
| AAEL003955 | | -0.1720 | 5.2200 | 0.8111 | 0.9996 | |

| | | | | | | |
|---|---|---|---|---|---|---|
| AAEL021513 | NA | -0.0767 | 4.0301 | 0.8111 | 0.9996 | NA |
| AAEL017560 | | 0.2264 | 3.0108 | 0.8112 | 0.9996 | |
| AAEL019619 | NA | -0.1445 | 4.7040 | 0.8112 | 0.9996 | NA |
| AAEL009531 | | 0.3568 | -1.7042 | 0.8113 | 0.9996 | niemann-pick C1 [Source:VB Community Annotation] |
| AAEL012387 | | 0.1367 | 3.6612 | 0.8114 | 0.9996 | |
| AAEL019449 | NA | -0.1300 | 5.4197 | 0.8114 | 0.9996 | NA |
| AAEL006187 | | -0.0745 | 4.9708 | 0.8116 | 0.9996 | translational activator gcn1 [Source:VB Community Annotation] |
| AAEL006508 | | 0.1473 | 1.1505 | 0.8116 | 0.9996 | |
| AAEL010861 | Med8 | 0.0791 | 4.3761 | 0.8117 | 0.9996 | Mediator of RNA polymerase II transcription subunit 8 (Med8) [Source:VB Community Annotation] |
| AAEL002918 | | -0.4346 | 1.2627 | 0.8118 | 0.9996 | centaurin beta [Source:VB Community Annotation] |
| AAEL009680 | | -0.7713 | 2.6925 | 0.8118 | 0.9996 | chymotrypsin, putative [Source:VB Community Annotation] |
| AAEL018244 | | 0.0931 | 6.5842 | 0.8119 | 0.9996 | DNA topoisomerase 2 [Source:UniProtKB/TrEMBL;Acc:A0A1S4G770 |
| AAEL011603 | | 0.0548 | 4.4879 | 0.8120 | 0.9996 | DNA topoisomerase [Source:UniProtKB/TrEMBL;Acc:Q16PL8] |
| AAEL009387 | | -0.1216 | 6.9733 | 0.8120 | 0.9996 | hexokinase [Source:VB Community Annotation] |
| AAEL008853 | | 0.3620 | 0.9988 | 0.8121 | 0.9996 | choline/ethanolamine kinase [Source:VB Community Annotation] |
| AAEL017402 | | 0.5837 | 3.5086 | 0.8122 | 0.9996 | |
| AAEL017177 | | 0.1836 | 3.9692 | 0.8123 | 0.9996 | |
| AAEL009789 | | 0.2865 | -0.8664 | 0.8123 | 0.9996 | |
| AAEL019685 | NA | -0.1567 | 4.0420 | 0.8123 | 0.9996 | NA |
| AAEL028103 | NA | -0.1540 | 0.8432 | 0.8123 | 0.9996 | NA |
| AAEL005945 | GPRDOP4 | 0.2827 | -0.6215 | 0.8124 | 0.9996 | GPCR Dopamine Family [Source:VB Community Annotation] |
| AAEL027554 | NA | -0.0931 | 6.0823 | 0.8124 | 0.9996 | NA |
| AAEL013086 | | 0.0972 | 4.0354 | 0.8125 | 0.9996 | |
| AAEL009635 | | 0.0805 | 3.9656 | 0.8128 | 0.9996 | |
| AAEL022514 | NA | 0.1684 | 1.1295 | 0.8130 | 0.9996 | NA |
| AAEL018514 | Arthropod_ | -0.1738 | 6.1066 | 0.8130 | 0.9996 | Arthropod 7SK RNA [Source:RFAM;Acc:RF01052] |
| AAEL022176 | NA | -0.2688 | 4.6822 | 0.8131 | 0.9996 | NA |
| AAEL019644 | NA | 0.0910 | 3.1948 | 0.8131 | 0.9996 | NA |
| AAEL021554 | NA | 0.2225 | 4.0986 | 0.8132 | 0.9996 | NA |
| AAEL024332 | NA | -0.5069 | 1.8431 | 0.8132 | 0.9996 | NA |
| AAEL013718 | | 0.2782 | 0.1834 | 0.8132 | 0.9996 | |
| AAEL024936 | NA | -0.1241 | 4.8192 | 0.8133 | 0.9996 | NA |
| AAEL022267 | NA | 0.2047 | 2.7163 | 0.8134 | 0.9996 | NA |
| AAEL006416 | | -0.1181 | 3.0446 | 0.8135 | 0.9996 | glycine rich RNA binding protein, putative [Source:VB Community Annotation] |
| AAEL005915 | | -0.3954 | 2.8614 | 0.8135 | 0.9996 | monocarboxylate transporter [Source:VB Community Annotation] |
| AAEL001134 | | 0.0785 | 8.0227 | 0.8136 | 0.9996 | Probable methylmalonate-semialdehyde dehydrogenase [acylating], mitochondrial Precursor (Malonate-semialdehyde dehydrogenase [a |
| AAEL019744 | NA | -0.3652 | -1.1840 | 0.8137 | 0.9996 | NA |
| AAEL011442 | | 0.0624 | 3.5075 | 0.8137 | 0.9996 | translation initiation factor eif-2b gamma subunit [Source:VB Community Annotation] |
| AAEL011788 | | -0.1228 | 2.0443 | 0.8138 | 0.9996 | |
| AAEL007942 | | -0.6663 | 0.5310 | 0.8138 | 0.9996 | fibrinogen and fibronectin [Source:VB Community Annotation] |

| | | | | | | |
|---|---|---|---|---|---|---|
| AAEL001836 | | -0.1614 | 2.0537 | 0.8138 | 0.9996 | odorant-binding protein 56a, putative [Source:VB Community Annotation] |
| AAEL000157 | | -0.1053 | 2.2083 | 0.8138 | 0.9996 | |
| AAEL007189 | | 0.0799 | 5.9139 | 0.8138 | 0.9996 | nocturnin [Source:VB Community Annotation] |
| AAEL025355 | NA | -0.2684 | 0.8385 | 0.8139 | 0.9996 | NA |
| AAEL008777 | | -0.1210 | 3.6126 | 0.8139 | 0.9996 | proto-oncogene tyrosine-protein kinase abl1 [Source:VB Community Annotation] |
| AAEL020477 | NA | 0.1218 | 4.2625 | 0.8140 | 0.9996 | NA |
| AAEL005769 | | -0.4233 | 1.4019 | 0.8141 | 0.9996 | glucose dehydrogenase [Source:VB Community Annotation] |
| AAEL019713 | NA | -0.3303 | 1.5819 | 0.8141 | 0.9996 | NA |
| AAEL014303 | | 0.0798 | 3.6517 | 0.8141 | 0.9996 | neuroligin, [Source:VB Community Annotation] |
| AAEL010797 | Med21 | 0.0752 | 4.3593 | 0.8142 | 0.9996 | Mediator of RNA polymerase II transcription subunit 21 (Med21) [Source:VB Community Annotation] |
| AAEL020003 | NA | 0.1974 | 5.8176 | 0.8143 | 0.9996 | NA |
| AAEL003006 | | -0.0689 | 6.3800 | 0.8144 | 0.9996 | 2-deoxyglucose-6-phosphate phosphatase [Source:VB Community Annotation] |
| AAEL001951 | Act-4 | 0.2898 | 2.3071 | 0.8146 | 0.9996 | actin [Source:VB Community Annotation] |
| AAEL021008 | NA | 0.1845 | 1.9864 | 0.8146 | 0.9996 | NA |
| AAEL027589 | NA | -0.0798 | 2.8539 | 0.8146 | 0.9996 | NA |
| AAEL003658 | | -0.1642 | 4.3221 | 0.8146 | 0.9996 | laminin beta-2 chain [Source:VB Community Annotation] |
| AAEL011183 | | -0.4129 | 4.3729 | 0.8147 | 0.9996 | |
| AAEL010047 | | -0.1104 | 4.3966 | 0.8147 | 0.9996 | |
| AAEL014377 | | -0.1440 | 1.0402 | 0.8149 | 0.9996 | Nibrin [Source:UniProtKB/TrEMBL;Acc:A0A1S4G1J0] |
| AAEL007624 | REL2 | -0.2435 | 3.1530 | 0.8151 | 0.9996 | IMD pathway signalling NF-kappaB Relish-like transcription factor [Source:VB Community Annotation] |
| AAEL023107 | NA | -0.0849 | 5.2716 | 0.8151 | 0.9996 | NA |
| AAEL012474 | | 0.1722 | 0.2478 | 0.8152 | 0.9996 | |
| AAEL013757 | | 0.2250 | -0.3586 | 0.8152 | 0.9996 | hexamerin 2 beta [Source:VB Community Annotation] |
| AAEL011052 | | 0.0878 | 3.0856 | 0.8153 | 0.9996 | |
| AAEL003131 | | -0.0862 | 5.4139 | 0.8154 | 0.9996 | |
| AAEL027970 | NA | -0.1304 | 3.5334 | 0.8156 | 0.9996 | NA |
| AAEL009080 | | -0.0811 | 5.0556 | 0.8160 | 0.9996 | importin 7, [Source:VB Community Annotation] |
| AAEL010465 | | -0.0598 | 4.4024 | 0.8160 | 0.9996 | gpi-anchor transamidase [Source:VB Community Annotation] |
| AAEL004844 | | -0.4513 | 1.4415 | 0.8160 | 0.9996 | nervous wreck, putative [Source:VB Community Annotation] |
| AAEL003600 | | -0.4281 | 8.2791 | 0.8161 | 0.9996 | |
| AAEL006172 | | -0.0779 | 6.4443 | 0.8162 | 0.9996 | |
| AAEL007401 | | -0.4329 | 3.6494 | 0.8164 | 0.9996 | roundabout, putative [Source:VB Community Annotation] |
| AAEL007374 | | 0.1732 | 7.8412 | 0.8165 | 0.9996 | yellow protein precursor, putative [Source:VB Community Annotation] |
| AAEL019918 | NA | -0.3251 | 4.9159 | 0.8166 | 0.9996 | NA |
| AAEL005966 | | -0.1111 | 7.5046 | 0.8167 | 0.9996 | |
| AAEL002912 | ciao1 | 0.0825 | 4.6400 | 0.8168 | 0.9996 | Probable cytosolic iron-sulfur protein assembly protein Ciao1 [Source:UniProtKB/Swiss-Prot;Acc:Q17GR9] |
| AAEL012120 | | 0.1066 | 5.7109 | 0.8168 | 0.9996 | fad oxidoreductase [Source:VB Community Annotation] |
| AAEL008395 | | 0.1484 | 1.3685 | 0.8168 | 0.9996 | |
| AAEL002115 | | 0.0916 | 3.2656 | 0.8168 | 0.9996 | |
| AAEL017320 | | 0.1044 | 6.8803 | 0.8168 | 0.9996 | |

| | | | | | | |
|---|---|---|---|---|---|---|
| AAEL000094 | | -0.2467 | 0.7205 | 0.8169 | 0.9996 | |
| AAEL007273 | | -0.0897 | 3.0005 | 0.8171 | 0.9996 | peptidyl-prolyl cis-trans isomerase e, ppie [Source:VB Community Annotation] |
| AAEL027986 | NA | -0.3349 | -1.8454 | 0.8172 | 0.9996 | NA |
| AAEL010403 | | 0.0711 | 5.9776 | 0.8172 | 0.9996 | past-1 [Source:VB Community Annotation] |
| AAEL027090 | NA | -0.0775 | 4.5219 | 0.8172 | 0.9996 | NA |
| AAEL020696 | NA | -0.0578 | 5.3577 | 0.8173 | 0.9996 | NA |
| AAEL001129 | | -0.0695 | 3.6276 | 0.8175 | 0.9996 | |
| AAEL010401 | disp | -0.1718 | 1.8728 | 0.8176 | 0.9996 | protein dispatched (segment polarity protein) [Source:VB Community Annotation] |
| AAEL002986 | | 0.0629 | 5.4542 | 0.8176 | 0.9996 | |
| AAEL002426 | | -0.0794 | 3.1483 | 0.8177 | 0.9996 | |
| AAEL023085 | NA | 0.0928 | 4.0917 | 0.8179 | 0.9996 | NA |
| AAEL008686 | | -0.2268 | 3.2506 | 0.8180 | 0.9996 | |
| AAEL013656 | | 0.1224 | 8.7149 | 0.8180 | 0.9996 | bm-40 precursor [Source:VB Community Annotation] |
| AAEL004713 | | 0.1993 | 0.0416 | 0.8180 | 0.9996 | |
| AAEL009428 | | 0.0700 | 4.0277 | 0.8180 | 0.9996 | |
| AAEL012448 | | -0.0689 | 4.0641 | 0.8181 | 0.9996 | |
| AAEL001775 | | 0.0732 | 3.5832 | 0.8181 | 0.9996 | |
| AAEL004616 | | 0.3983 | 1.3085 | 0.8183 | 0.9996 | actin [Source:VB Community Annotation] |
| AAEL010793 | | 0.1187 | 4.9212 | 0.8184 | 0.9996 | F-box/leucine rich repeat protein [Source:VB Community Annotation] |
| AAEL021012 | NA | -0.1814 | 1.6286 | 0.8186 | 0.9996 | NA |
| AAEL013371 | | -0.1600 | 5.8414 | 0.8186 | 0.9996 | |
| AAEL007508 | | 0.3294 | 0.8756 | 0.8188 | 0.9996 | oviductin [Source:VB Community Annotation] |
| AAEL007342 | | 0.0814 | 5.5290 | 0.8188 | 0.9996 | |
| AAEL000170 | | 0.2856 | 1.7577 | 0.8189 | 0.9996 | |
| AAEL019631 | NA | 0.4691 | 0.9443 | 0.8190 | 0.9996 | NA |
| AAEL024793 | NA | 0.0782 | 4.5284 | 0.8190 | 0.9996 | NA |
| AAEL004427 | | 0.0627 | 8.2046 | 0.8190 | 0.9996 | Prefoldin subunit 3 [Source:UniProtKB/TrEMBL;Acc:Q17CV4] |
| AAEL001207 | | -0.2855 | 4.2906 | 0.8191 | 0.9996 | |
| AAEL020302 | NA | -0.0707 | 5.5707 | 0.8192 | 0.9996 | NA |
| AAEL000660 | | 0.0991 | 3.4870 | 0.8192 | 0.9996 | peptide methionine sulfoxide reductase, putative [Source:VB Community Annotation] |
| AAEL008183 | | -0.2210 | -0.0476 | 0.8192 | 0.9996 | t complex protein [Source:VB Community Annotation] |
| AAEL026419 | NA | -0.2124 | 0.3575 | 0.8192 | 0.9996 | NA |
| AAEL007206 | | -0.1675 | 5.0541 | 0.8194 | 0.9996 | |
| AAEL027242 | NA | 0.3177 | 2.4335 | 0.8196 | 0.9996 | NA |
| AAEL006342 | | 0.0849 | 6.1729 | 0.8196 | 0.9996 | epoxide hydrolase [Source:VB Community Annotation] |
| AAEL026023 | NA | 0.2629 | 3.2581 | 0.8197 | 0.9996 | NA |
| AAEL011837 | | -0.1453 | 2.5008 | 0.8197 | 0.9996 | cAMP-dependent protein kinase catalytic subunit [Source:VB Community Annotation] |
| AAEL022320 | NA | -0.1627 | 2.3535 | 0.8197 | 0.9996 | NA |
| AAEL012209 | | -0.1257 | 1.6200 | 0.8198 | 0.9996 | ring finger protein [Source:VB Community Annotation] |
| AAEL001236 | | -0.0631 | 5.5506 | 0.8200 | 0.9996 | |

| | | | | | | |
|---|---|---|---|---|---|---|
| AAEL002179 | | -0.0888 | 4.5750 | 0.8201 | 0.9996 | |
| AAEL013473 | | 0.3751 | -1.0178 | 0.8201 | 0.9996 | cAMP-specific 3,5-cyclic phosphodiesterase [Source:VB Community Annotation] |
| AAEL000291 | | 0.0731 | 9.0017 | 0.8202 | 0.9996 | V-type proton ATPase 16 kDa proteolipid subunit [Source:UniProtKB/Swiss-Prot;Acc:O16110] |
| AAEL009066 | | 0.1187 | 8.6076 | 0.8202 | 0.9996 | symbol, putative [Source:VB Community Annotation] |
| AAEL026297 | NA | 0.0764 | 3.1070 | 0.8203 | 0.9996 | NA |
| AAEL021175 | NA | -0.1020 | 2.6718 | 0.8204 | 0.9996 | NA |
| AAEL011627 | | -0.0826 | 5.9037 | 0.8205 | 0.9996 | ribose-5-phosphate isomerase [Source:VB Community Annotation] |
| AAEL014295 | | 0.0677 | 5.0341 | 0.8205 | 0.9996 | |
| AAEL011338 | | -0.0999 | 3.4238 | 0.8206 | 0.9996 | |
| AAEL025806 | NA | 0.4560 | 2.5552 | 0.8206 | 0.9996 | NA |
| AAEL009334 | | 0.0760 | 3.4630 | 0.8206 | 0.9996 | |
| AAEL022689 | NA | -0.3415 | 2.3002 | 0.8208 | 0.9996 | NA |
| AAEL025472 | NA | 0.0933 | 2.6389 | 0.8208 | 0.9996 | NA |
| AAEL013952 | | 0.0828 | 6.6545 | 0.8208 | 0.9996 | prohibitin [Source:VB Community Annotation] |
| AAEL020597 | NA | -0.0643 | 6.6972 | 0.8208 | 0.9996 | NA |
| AAEL002070 | | -0.3475 | 1.0177 | 0.8209 | 0.9996 | |
| AAEL000270 | | -0.0893 | 5.7224 | 0.8210 | 0.9996 | 26S proteasome regulatory subunit 7, psd7 [Source:VB Community Annotation] |
| AAEL010412 | | 0.1147 | 1.5156 | 0.8210 | 0.9996 | |
| AAEL006782 | Carm1 | -0.0657 | 4.3426 | 0.8211 | 0.9996 | Histone-arginine methyltransferase CARMER [Source:UniProtKB/Swiss-Prot;Acc:Q174R2] |
| AAEL000284 | | 0.0785 | 3.8254 | 0.8211 | 0.9996 | Protein-lysine N-methyltransferase AAEL000284 [Source:UniProtKB/TrEMBL;Acc:Q17PM4] |
| AAEL013532 | | -0.3671 | 4.2641 | 0.8211 | 0.9996 | |
| AAEL021191 | NA | 0.3166 | -1.1164 | 0.8212 | 0.9996 | NA |
| AAEL019996 | NA | -0.3720 | 5.6577 | 0.8215 | 0.9996 | NA |
| AAEL014622 | | -0.1872 | 4.7024 | 0.8216 | 0.9996 | |
| AAEL018103 | | -0.1224 | 4.9004 | 0.8216 | 0.9996 | |
| AAEL025836 | NA | -0.0661 | 3.9400 | 0.8216 | 0.9996 | NA |
| AAEL024628 | NA | -0.1124 | 1.4680 | 0.8218 | 0.9996 | NA |
| AAEL018320 | | -0.3262 | 2.3931 | 0.8218 | 0.9996 | |
| AAEL023994 | NA | -0.3561 | -1.3273 | 0.8219 | 0.9996 | NA |
| AAEL004172 | | 0.3133 | -1.1623 | 0.8219 | 0.9996 | Tubulin alpha chain [Source:UniProtKB/TrEMBL;Acc:Q0IFT7] |
| AAEL019879 | NA | 0.0587 | 4.7814 | 0.8220 | 0.9996 | NA |
| AAEL022786 | NA | -0.0682 | 3.2611 | 0.8221 | 0.9996 | NA |
| AAEL028119 | NA | -0.0602 | 4.5166 | 0.8222 | 0.9996 | NA |
| AAEL023810 | NA | -0.5304 | 1.6951 | 0.8222 | 0.9996 | NA |
| AAEL014913 | | 0.0765 | 9.8706 | 0.8223 | 0.9996 | Pyruvate kinase [Source:UniProtKB/TrEMBL;Acc:Q16F38] |
| AAEL010772 | | -0.1107 | 3.1033 | 0.8223 | 0.9996 | Leucine-rich repeat-containing protein 50 homolog [Source:VB Community Annotation] |
| AAEL014054 | | 0.1158 | 1.8254 | 0.8223 | 0.9996 | |
| AAEL006054 | | -0.2136 | 3.0840 | 0.8224 | 0.9996 | peptidyl-prolyl cis-trans isomerase (cyclophilin) [Source:VB Community Annotation] |
| AAEL023543 | NA | 0.0525 | 4.8321 | 0.8224 | 0.9996 | NA |
| AAEL014254 | | 0.1460 | 1.3716 | 0.8225 | 0.9996 | |

| | | | | | | |
|---|---|---|---|---|---|---|
| AAEL020306 | NA | -0.1385 | 2.0521 | 0.8226 | 0.9996 | NA |
| AAEL014170 | | 0.1241 | 2.2159 | 0.8226 | 0.9996 | |
| AAEL018353 | | -0.1589 | 1.9980 | 0.8227 | 0.9996 | |
| AAEL025237 | NA | 0.0701 | 4.1335 | 0.8228 | 0.9996 | NA |
| AAEL025684 | NA | -0.2737 | -0.4441 | 0.8229 | 0.9996 | NA |
| AAEL012633 | | -0.4814 | 1.2033 | 0.8230 | 0.9996 | |
| AAEL023188 | NA | -0.0507 | 6.3520 | 0.8230 | 0.9996 | NA |
| AAEL014644 | | -0.0670 | 5.4176 | 0.8230 | 0.9996 | UDP-sugar transporter UST74c (fringe connection protein), putative [Source:VB Community Annotation] |
| AAEL003180 | | 0.0597 | 5.4648 | 0.8231 | 0.9996 | prefoldin subunit [Source:VB Community Annotation] |
| AAEL023492 | NA | 0.3246 | 0.7789 | 0.8232 | 0.9996 | NA |
| AAEL005507 | | -0.3488 | -0.2477 | 0.8232 | 0.9996 | inhibitory pou [Source:VB Community Annotation] |
| AAEL020287 | NA | -0.3413 | -0.6319 | 0.8234 | 0.9996 | NA |
| AAEL019726 | NA | -0.1276 | 5.6725 | 0.8235 | 0.9996 | NA |
| AAEL002757 | | 0.1560 | 2.0490 | 0.8235 | 0.9996 | |
| AAEL003934 | | 0.1135 | 9.6546 | 0.8235 | 0.9996 | |
| AAEL007523 | | -0.0645 | 3.6747 | 0.8237 | 0.9996 | peroxisomal n1-acetyl-spermine/spermidine oxidase [Source:VB Community Annotation] |
| AAEL010107 | | 0.0856 | 4.2274 | 0.8239 | 0.9996 | |
| AAEL009124 | CYP6N12 | -0.1124 | 3.5565 | 0.8239 | 0.9996 | cytochrome P450 [Source:VB Community Annotation] |
| AAEL023395 | NA | -0.3681 | -1.0423 | 0.8242 | 0.9996 | NA |
| AAEL004136 | | 0.0922 | 3.5887 | 0.8244 | 0.9996 | |
| AAEL024930 | NA | -0.3125 | -0.3868 | 0.8244 | 0.9996 | NA |
| AAEL021520 | NA | -0.1757 | 7.8403 | 0.8244 | 0.9996 | NA |
| AAEL017300 | | -0.2300 | 2.1947 | 0.8245 | 0.9996 | |
| AAEL002739 | | 0.0722 | 2.5286 | 0.8246 | 0.9996 | |
| AAEL009375 | | -0.0665 | 5.5836 | 0.8246 | 0.9996 | plekhh1 [Source:VB Community Annotation] |
| AAEL001832 | | 0.0974 | 2.9980 | 0.8246 | 0.9996 | |
| AAEL004951 | | -0.1362 | 6.0242 | 0.8247 | 0.9996 | |
| AAEL004930 | | 0.0550 | 7.6175 | 0.8248 | 0.9996 | carbonic anhydrase [Source:VB Community Annotation] |
| AAEL026214 | NA | 0.0967 | 3.0533 | 0.8250 | 0.9996 | NA |
| AAEL009822 | GPRMGL5 | -0.1773 | 0.3500 | 0.8250 | 0.9996 | GPCR Metabotropic glutamate Family [Source:VB Community Annotation] |
| AAEL018226 | | -0.1032 | 3.4684 | 0.8252 | 0.9996 | |
| AAEL013871 | | 0.0942 | 5.3954 | 0.8252 | 0.9996 | |
| AAEL013613 | | -0.1316 | 7.8265 | 0.8253 | 0.9996 | Pyruvate dehydrogenase E1 component subunit alpha [Source:UniProtKB/TrEMBL;Acc:Q16IL3] |
| AAEL018146 | | 0.2351 | 0.7986 | 0.8253 | 0.9996 | |
| AAEL010287 | | -0.0852 | 2.1456 | 0.8256 | 0.9996 | |
| AAEL007784 | | -0.1007 | 4.4401 | 0.8256 | 0.9996 | |
| AAEL013260 | | -0.0933 | 2.6709 | 0.8256 | 0.9996 | alpha methylacyl-coa racemase [Source:VB Community Annotation] |
| AAEL005694 | | -0.1571 | 0.6963 | 0.8256 | 0.9996 | |
| AAEL009013 | | -0.3087 | -1.6998 | 0.8256 | 0.9996 | adult cuticle protein, putative [Source:VB Community Annotation] |
| AAEL000854 | | -0.0786 | 5.5508 | 0.8257 | 0.9996 | fumarylacetoacetate hydrolase [Source:VB Community Annotation] |

| | | | | | | |
|---|---|---|---|---|---|---|
| AAEL025569 | NA | -0.3820 | 2.1937 | 0.8258 | 0.9996 | NA |
| AAEL003752 | | -0.0825 | 5.3435 | 0.8258 | 0.9996 | |
| AAEL011082 | | 0.0592 | 4.7182 | 0.8260 | 0.9996 | |
| AAEL019839 | NA | 0.1277 | 1.1521 | 0.8260 | 0.9996 | NA |
| AAEL026517 | NA | 0.1012 | 4.6573 | 0.8261 | 0.9996 | NA |
| AAEL023194 | NA | -0.0763 | 3.4433 | 0.8261 | 0.9996 | NA |
| AAEL022490 | NA | -0.0674 | 6.1072 | 0.8261 | 0.9996 | NA |
| AAEL004307 | | -0.4744 | 2.3567 | 0.8262 | 0.9996 | n-myc downstream regulated [Source:VB Community Annotation] |
| AAEL015275 | | 0.0568 | 4.0474 | 0.8262 | 0.9996 | |
| AAEL006589 | | 0.0713 | 3.9482 | 0.8263 | 0.9996 | |
| AAEL002925 | | -0.1111 | 3.0897 | 0.8264 | 0.9996 | |
| AAEL014410 | | 0.0888 | 2.9277 | 0.8265 | 0.9996 | |
| AAEL008131 | | -0.0625 | 7.9392 | 0.8266 | 0.9996 | cytidylate kinase [Source:VB Community Annotation] |
| AAEL003123 | | -0.6122 | 1.2465 | 0.8266 | 0.9996 | deoxyribonuclease I, putative [Source:VB Community Annotation] |
| AAEL010428 | Or26 | -0.2429 | -0.1482 | 0.8266 | 0.9996 | odorant receptor (Or26) [Source:VB Community Annotation] |
| AAEL007139 | | 0.3923 | 1.2378 | 0.8267 | 0.9996 | sugar transporter [Source:VB Community Annotation] |
| AAEL010896 | | 0.0894 | 5.1448 | 0.8267 | 0.9996 | WD-repeat protein [Source:VB Community Annotation] |
| AAEL026736 | NA | -0.1557 | 3.1125 | 0.8267 | 0.9996 | NA |
| AAEL008195 | | 0.0696 | 6.4641 | 0.8267 | 0.9996 | |
| AAEL015394 | GPRMGL3_ | -0.4296 | 1.4784 | 0.8267 | 0.9996 | GPCR Metabotropic glutamate Family [Source:VB Community Annotation] |
| AAEL025513 | NA | -0.2290 | 0.3461 | 0.8267 | 0.9996 | NA |
| AAEL010337 | | 0.1226 | 6.3041 | 0.8268 | 0.9996 | CRAL/TRIO domain-containing protein [Source:VB Community Annotation] |
| AAEL002295 | | -0.0922 | 5.7886 | 0.8268 | 0.9996 | leucine-rich transmembrane protein [Source:VB Community Annotation] |
| AAEL013420 | Or79 | 0.2406 | -0.0817 | 0.8269 | 0.9996 | Odorant receptor [Source:UniProtKB/TrEMBL;Acc:Q16J75] |
| AAEL010327 | | -0.1387 | 0.7747 | 0.8273 | 0.9996 | six/sine homebox transcription factors [Source:VB Community Annotation] |
| AAEL004113 | | 0.1055 | 5.6695 | 0.8274 | 0.9996 | |
| AAEL000666 | | 0.0522 | 4.6134 | 0.8274 | 0.9996 | pmp22 peroxisomal membrane protein, putative [Source:VB Community Annotation] |
| AAEL007871 | | -0.0686 | 4.2984 | 0.8275 | 0.9996 | Anaphase-promoting complex subunit 10 [Source:UniProtKB/TrEMBL;Acc:Q170M2] |
| AAEL020969 | NA | -0.3713 | 0.7208 | 0.8276 | 0.9996 | NA |
| AAEL019739 | NA | -0.2436 | 2.1706 | 0.8277 | 0.9996 | NA |
| AAEL021067 | NA | 0.2704 | -1.8234 | 0.8277 | 0.9996 | NA |
| AAEL007420 | SRPN25 | -0.3976 | 5.0556 | 0.8277 | 0.9996 | Serine Protease Inhibitor (serpin) homologue - unlikely to be inhibitory [Source:VB Community Annotation] |
| AAEL008594 | | 0.0505 | 6.3883 | 0.8279 | 0.9996 | |
| AAEL005003 | | -0.0849 | 4.2637 | 0.8281 | 0.9996 | serine/threonine-protein kinase, putative [Source:VB Community Annotation] |
| AAEL014821 | | 0.0598 | 6.5003 | 0.8281 | 0.9996 | |
| AAEL026914 | NA | 0.3910 | -0.9071 | 0.8282 | 0.9996 | NA |
| AAEL020573 | NA | 0.0834 | 3.7386 | 0.8282 | 0.9996 | NA |
| AAEL004127 | | 0.1244 | 3.2878 | 0.8283 | 0.9996 | acyl-coa dehydrogenase [Source:VB Community Annotation] |
| AAEL003137 | | -0.0641 | 7.4508 | 0.8284 | 0.9996 | |
| AAEL025658 | NA | 0.0658 | 4.1489 | 0.8285 | 0.9996 | NA |

| | | | | | | |
|---|---|---|---|---|---|---|
| AAEL023839 | NA | 0.1235 | 2.7164 | 0.8285 | 0.9996 | NA |
| AAEL027655 | NA | -0.3550 | 1.5755 | 0.8287 | 0.9996 | NA |
| AAEL018235 | | 0.0781 | 5.6205 | 0.8288 | 0.9996 | |
| AAEL011184 | | -0.0937 | 9.8515 | 0.8289 | 0.9996 | mitochondrial phosphate carrier protein [Source:VB Community Annotation] |
| AAEL005405 | | 0.0484 | 5.5756 | 0.8289 | 0.9996 | |
| AAEL009009 | | 0.2182 | 4.2006 | 0.8290 | 0.9996 | |
| AAEL008561 | | -0.0786 | 4.7381 | 0.8291 | 0.9996 | |
| AAEL022462 | NA | -0.0712 | 5.9372 | 0.8291 | 0.9996 | NA |
| AAEL021244 | NA | -0.3643 | 2.1555 | 0.8292 | 0.9996 | NA |
| AAEL000243 | | -0.2509 | -0.2513 | 0.8292 | 0.9996 | leucine-rich transmembrane protein [Source:VB Community Annotation] |
| AAEL003722 | | -0.1526 | 2.5331 | 0.8292 | 0.9996 | ribonuclease UK114, putative [Source:VB Community Annotation] |
| AAEL011950 | | -0.3766 | -0.7572 | 0.8294 | 0.9996 | |
| AAEL019536 | NA | -0.0663 | 5.1786 | 0.8297 | 0.9996 | NA |
| AAEL004948 | | 0.1237 | 1.1008 | 0.8297 | 0.9996 | |
| AAEL007069 | | 0.0714 | 3.7528 | 0.8297 | 0.9996 | |
| AAEL018267 | | -0.1727 | 2.0763 | 0.8298 | 0.9996 | |
| AAEL005401 | | 0.1093 | 6.4812 | 0.8300 | 0.9996 | |
| AAEL013997 | | -0.1530 | 7.5594 | 0.8300 | 0.9996 | guanine nucleotide-binding protein beta 3 (g protein beta3) [Source:VB Community Annotation] |
| AAEL005354 | | -0.0934 | 6.7678 | 0.8300 | 0.9996 | dynein light chain, putative [Source:VB Community Annotation] |
| AAEL006133 | | 0.0822 | 5.5719 | 0.8302 | 0.9996 | cofactor A, putative [Source:VB Community Annotation] |
| AAEL026265 | NA | 0.2048 | 6.1734 | 0.8302 | 0.9996 | NA |
| AAEL008084 | | -0.0779 | 4.1546 | 0.8302 | 0.9996 | phosphatidylserine receptor [Source:VB Community Annotation] |
| AAEL006320 | | 0.0856 | 5.0915 | 0.8302 | 0.9996 | protein tyrosine phosphatase 69d, drome [Source:VB Community Annotation] |
| AAEL018251 | | -0.3354 | 1.6844 | 0.8303 | 0.9996 | |
| AAEL012855 | | -0.2302 | 3.5482 | 0.8303 | 0.9996 | |
| AAEL022729 | NA | 0.0636 | 6.7213 | 0.8304 | 0.9996 | NA |
| AAEL004281 | | -0.2781 | 0.6264 | 0.8304 | 0.9996 | short-chain dehydrogenase [Source:VB Community Annotation] |
| AAEL018039 | | 0.3328 | 2.9588 | 0.8305 | 0.9996 | |
| AAEL017826 | U1 | -0.1250 | 5.6860 | 0.8306 | 0.9996 | U1 spliceosomal RNA [Source:RFAM;Acc:RF00003] |
| AAEL002726 | | -0.0991 | 2.3609 | 0.8306 | 0.9996 | D7 protein, putative [Source:VB Community Annotation] |
| AAEL001896 | | -0.0592 | 6.4024 | 0.8309 | 0.9996 | UDP-N-acetylglucosamine: polypeptide-N-acetylglucosaminyl transferase, putative [Source:VB Community Annotation] |
| AAEL008840 | | -0.1243 | 3.5016 | 0.8310 | 0.9996 | Bardet-Biedl syndrome 2 protein homolog [Source:UniProtKB/TrEMBL;Acc:Q16XJ9] |
| AAEL025362 | NA | 0.0564 | 10.9386 | 0.8310 | 0.9996 | NA |
| AAEL015527 | | -0.1091 | 4.5416 | 0.8312 | 0.9996 | |
| AAEL026339 | NA | -0.2105 | 0.2877 | 0.8312 | 0.9996 | NA |
| AAEL022232 | NA | -0.1246 | 3.1458 | 0.8313 | 0.9996 | NA |
| AAEL013858 | | 0.0656 | 5.0171 | 0.8314 | 0.9996 | raf [Source:VB Community Annotation] |
| AAEL013085 | | 0.0536 | 5.3108 | 0.8315 | 0.9996 | |
| AAEL022919 | NA | -0.0678 | 3.8691 | 0.8318 | 0.9996 | NA |
| AAEL021288 | NA | -0.2915 | 3.2359 | 0.8318 | 0.9996 | NA |

| | | | | | | |
|---|---|---|---|---|---|---|
| AAEL012343 | | -0.0897 | 2.7517 | 0.8319 | 0.9996 | lysosomal acid lipase, putative [Source:VB Community Annotation] |
| AAEL026179 | NA | -0.4057 | 1.1756 | 0.8319 | 0.9996 | NA |
| AAEL007526 | | -0.0867 | 6.8280 | 0.8319 | 0.9996 | electron transfer flavoprotein-ubiquinone oxidoreductase [Source:VB Community Annotation] |
| AAEL005648 | CLIPB16 | -0.0872 | 3.2181 | 0.8321 | 0.9996 | Clip-Domain Serine Protease  family B. Protease homologue. [Source:VB Community Annotation] |
| AAEL019572 | NA | 0.3199 | 2.3973 | 0.8321 | 0.9996 | NA |
| AAEL010591 | GSTD6 | 0.1167 | 2.3602 | 0.8322 | 0.9996 | glutathione transferase [Source:VB Community Annotation] |
| AAEL000669 | | 0.5207 | 0.7054 | 0.8323 | 0.9996 | |
| AAEL026075 | NA | 0.3459 | 3.5593 | 0.8323 | 0.9996 | NA |
| AAEL018059 | | -0.2728 | 2.3891 | 0.8327 | 0.9996 | |
| AAEL007017 | | 0.2393 | 0.1875 | 0.8328 | 0.9996 | |
| AAEL011159 | | -0.0895 | 2.3028 | 0.8328 | 0.9996 | cartilage associated protein [Source:VB Community Annotation] |
| AAEL003363 | | 0.0719 | 4.5417 | 0.8329 | 0.9996 | phosphatidic acid phosphatase [Source:VB Community Annotation] |
| AAEL004853 | | 0.0846 | 3.8535 | 0.8331 | 0.9996 | glycerol kinase [Source:VB Community Annotation] |
| AAEL001816 | | -0.1856 | 2.1812 | 0.8331 | 0.9996 | glucosyl/glucuronosyl transferases [Source:VB Community Annotation] |
| AAEL022304 | NA | -0.2190 | 2.4730 | 0.8332 | 0.9996 | NA |
| AAEL027661 | NA | -0.4749 | 1.1292 | 0.8332 | 0.9996 | NA |
| AAEL027912 | NA | -0.0983 | 4.0868 | 0.8332 | 0.9996 | NA |
| AAEL013382 | | 0.0823 | 6.6904 | 0.8332 | 0.9996 | nudix hydrolase 6 [Source:VB Community Annotation] |
| AAEL008527 | | 0.1090 | 4.2926 | 0.8332 | 0.9996 | zinc metalloprotease [Source:VB Community Annotation] |
| AAEL025731 | NA | -0.0961 | 2.5510 | 0.8333 | 0.9996 | NA |
| AAEL017659 | U3 | -0.1034 | 3.9902 | 0.8334 | 0.9996 | Small nucleolar RNA U3 [Source:RFAM;Acc:RF00012] |
| AAEL021626 | NA | -0.2679 | -2.4119 | 0.8335 | 0.9996 | NA |
| AAEL002109 | | 0.2532 | 0.1021 | 0.8335 | 0.9996 | |
| AAEL001205 | | -0.0767 | 5.5653 | 0.8335 | 0.9996 | ubiquitin protein ligase [Source:VB Community Annotation] |
| AAEL010284 | | -0.0756 | 3.8293 | 0.8338 | 0.9996 | aliphatic nitrilase, putative [Source:VB Community Annotation] |
| AAEL010840 | | 0.0824 | 8.2520 | 0.8339 | 0.9996 | |
| AAEL013353 | | -0.1292 | 6.8278 | 0.8339 | 0.9996 | Profilin [Source:UniProtKB/TrEMBL;Acc:Q16JE9] |
| AAEL004060 | | -0.1067 | 10.8627 | 0.8341 | 0.9996 | |
| AAEL013623 | | -0.2830 | 3.6670 | 0.8341 | 0.9996 | trypsin [Source:VB Community Annotation] |
| AAEL018164 | | -0.0627 | 6.4898 | 0.8341 | 0.9996 | |
| AAEL019881 | NA | -0.0938 | 3.4896 | 0.8341 | 0.9996 | NA |
| AAEL013967 | | -0.0944 | 4.7348 | 0.8342 | 0.9996 | Methylmalonyl-CoA carboxyltransferase 12S subunit, putative [Source:VB Community Annotation] |
| AAEL026018 | NA | 0.1641 | 3.5079 | 0.8343 | 0.9996 | NA |
| AAEL009821 | | -0.2740 | 2.0372 | 0.8343 | 0.9996 | |
| AAEL012778 | APN1 | 0.3710 | 0.7419 | 0.8345 | 0.9996 | protease m1 zinc metalloprotease [Source:VB Community Annotation] |
| AAEL025730 | NA | -0.3504 | -1.8286 | 0.8345 | 0.9996 | NA |
| AAEL028907 | NA | -0.3444 | -1.2579 | 0.8346 | 0.9996 | NA |
| AAEL001778 | | 0.0590 | 3.9827 | 0.8347 | 0.9996 | zinc finger protein, putative [Source:VB Community Annotation] |
| AAEL012588 | | 0.0826 | 7.1085 | 0.8348 | 0.9996 | Hsp70-interacting protein, putative [Source:VB Community Annotation] |
| AAEL008219 | | -0.0949 | 2.1166 | 0.8348 | 0.9996 | elongase, putative [Source:VB Community Annotation] |

| | | | | | | |
|---|---|---|---|---|---|---|
| AAEL002422 | | -0.3346 | 1.0897 | 0.8348 | 0.9996 | cytoplasmic polyadenylation element binding protein (cpeb) [Source:VB Community Annotation] |
| AAEL027868 | NA | -0.2468 | 0.1316 | 0.8349 | 0.9996 | NA |
| AAEL007156 | | -0.0915 | 3.1544 | 0.8349 | 0.9996 | |
| AAEL019864 | NA | -0.0497 | 5.3051 | 0.8350 | 0.9996 | NA |
| AAEL018317 | | -0.3560 | 3.6110 | 0.8351 | 0.9996 | |
| AAEL004006 | | -0.3750 | 2.5111 | 0.8351 | 0.9996 | acetylcholine receptor protein alpha 1, 2, 3, 4 invertebrate [Source:VB Community Annotation] |
| AAEL001517 | | -0.1599 | 0.9369 | 0.8353 | 0.9996 | carboxylesterase [Source:VB Community Annotation] |
| AAEL012818 | | -0.0706 | 3.9822 | 0.8354 | 0.9996 | |
| AAEL026466 | NA | -0.2629 | 4.4797 | 0.8354 | 0.9996 | NA |
| AAEL023332 | NA | -0.0832 | 6.1117 | 0.8355 | 0.9996 | NA |
| AAEL020303 | NA | -0.1040 | 2.4462 | 0.8356 | 0.9996 | NA |
| AAEL014019 | CYP4J16 | -0.1325 | 3.2964 | 0.8357 | 0.9996 | cytochrome P450 [Source:VB Community Annotation] |
| AAEL012369 | | 0.4128 | 2.0573 | 0.8358 | 0.9996 | |
| AAEL008769 | | -0.4042 | 2.5923 | 0.8358 | 0.9996 | serine-type enodpeptidase, [Source:VB Community Annotation] |
| AAEL009422 | | 0.0787 | 10.2861 | 0.8358 | 0.9996 | |
| AAEL000689 | | 0.0943 | 2.1702 | 0.8359 | 0.9996 | steroid dehydrogenase [Source:VB Community Annotation] |
| AAEL017010 | | -0.0775 | 3.0341 | 0.8361 | 0.9996 | DNA-directed RNA polymerase subunit beta [Source:UniProtKB/TrEMBL;Acc:J9E9J9] |
| AAEL009142 | | 0.0506 | 6.3139 | 0.8362 | 0.9996 | prolyl endopeptidase (prolyl oligopeptidase) [Source:VB Community Annotation] |
| AAEL012029 | | 0.1172 | 5.7680 | 0.8362 | 0.9996 | |
| AAEL019983 | NA | -0.1782 | 6.6516 | 0.8362 | 0.9996 | NA |
| AAEL001389 | | -0.0496 | 5.1618 | 0.8363 | 0.9996 | Palmitoyltransferase [Source:UniProtKB/TrEMBL;Acc:Q17LD8] |
| AAEL020236 | NA | 0.0946 | 6.6666 | 0.8363 | 0.9996 | NA |
| AAEL012185 | | 0.1263 | 5.0802 | 0.8365 | 0.9996 | ribosome biogenesis regulatory protein [Source:VB Community Annotation] |
| AAEL027341 | NA | -0.1723 | 0.2221 | 0.8365 | 0.9996 | NA |
| AAEL013244 | | 0.0846 | 2.5770 | 0.8369 | 0.9996 | |
| AAEL011953 | ORP9 | -0.0761 | 5.7870 | 0.8370 | 0.9996 | oxysterol-binding protein related protein (ORP9) [Source:VB Community Annotation] |
| AAEL025259 | NA | 0.0888 | 2.1732 | 0.8371 | 0.9996 | NA |
| AAEL005206 | | -0.1294 | 3.1461 | 0.8373 | 0.9996 | c-terminal pdz ligand of neuronal nitric oxide synthase protein [Source:VB Community Annotation] |
| AAEL013870 | | -0.1108 | 3.9504 | 0.8375 | 0.9996 | |
| AAEL001427 | | -0.1069 | 2.1931 | 0.8375 | 0.9996 | short-chain dehydrogenase [Source:VB Community Annotation] |
| AAEL009639 | | -0.3185 | 2.7353 | 0.8376 | 0.9996 | Ubiquitin-like protein [Source:UniProtKB/TrEMBL;Acc:Q16V89] |
| AAEL001112 | | -0.0646 | 5.4493 | 0.8377 | 0.9996 | Ubiquitinyl hydrolase 1 [Source:UniProtKB/TrEMBL;Acc:Q17M51] |
| AAEL007462 | | -0.0612 | 4.4967 | 0.8378 | 0.9996 | |
| AAEL000809 | | -0.0891 | 2.2371 | 0.8378 | 0.9996 | |
| AAEL012772 | CYP325G3 | 0.2014 | 1.7205 | 0.8381 | 0.9996 | cytochrome P450 [Source:VB Community Annotation] |
| AAEL019977 | NA | -0.2784 | 4.5691 | 0.8383 | 0.9996 | NA |
| AAEL001779 | | 0.1826 | 5.1290 | 0.8383 | 0.9996 | bax inhibitor [Source:VB Community Annotation] |
| AAEL008167 | | 0.1161 | 7.4222 | 0.8383 | 0.9996 | aspartate ammonia lyase [Source:VB Community Annotation] |
| AAEL025824 | NA | 0.3707 | 1.1785 | 0.8385 | 0.9996 | NA |
| AAEL013090 | | 0.0761 | 4.2929 | 0.8386 | 0.9996 | |

| | | | | | | |
|---|---|---|---|---|---|---|
| AAEL005349 | | -0.1925 | 2.2727 | 0.8386 | 0.9996 | Sugar transporter SWEET [Source:UniProtKB/TrEMBL;Acc:Q17A98] |
| AAEL021615 | NA | -0.1205 | 4.5139 | 0.8387 | 0.9996 | NA |
| AAEL011356 | | -0.0704 | 2.6133 | 0.8390 | 0.9996 | alcohol dehydrogenase [Source:VB Community Annotation] |
| AAEL021909 | NA | 0.1911 | 0.7393 | 0.8390 | 0.9996 | NA |
| AAEL026055 | NA | 0.1009 | 2.6714 | 0.8390 | 0.9996 | NA |
| AAEL004121 | | 0.1837 | 4.0848 | 0.8392 | 0.9996 | ubiquitin-conjugating enzyme E2 q [Source:VB Community Annotation] |
| AAEL009879 | | 0.0725 | 4.4525 | 0.8394 | 0.9996 | |
| AAEL022060 | NA | 0.0512 | 5.2587 | 0.8397 | 0.9996 | NA |
| AAEL008514 | | -0.0579 | 6.1501 | 0.8397 | 0.9996 | ubiquitin conjugating enzyme E2, putative [Source:VB Community Annotation] |
| AAEL002411 | | 0.0461 | 8.3844 | 0.8398 | 0.9996 | |
| AAEL017458 | | 0.1158 | 2.1026 | 0.8398 | 0.9996 | |
| AAEL012536 | | -0.0839 | 5.7246 | 0.8398 | 0.9996 | |
| AAEL026211 | NA | 0.1916 | 0.0480 | 0.8399 | 0.9996 | NA |
| AAEL014250 | | -0.0927 | 4.1920 | 0.8399 | 0.9996 | insect replication protein a [Source:VB Community Annotation] |
| AAEL018036 | | -0.0876 | 3.7293 | 0.8399 | 0.9996 | Putative somatomedin-b and thrombospondin type-1 domain-containing protein [Source:UniProtKB/TrEMBL;Acc:A0A0P6JRX5] |
| AAEL001211 | | 0.0572 | 4.1461 | 0.8399 | 0.9996 | COMPASS component SWD2, putative [Source:VB Community Annotation] |
| AAEL007632 | | -0.1726 | 0.8998 | 0.8400 | 0.9996 | myosin light chain kinase [Source:VB Community Annotation] |
| AAEL009912 | | 0.1243 | 1.9852 | 0.8401 | 0.9996 | |
| AAEL009742 | abd-A | -0.2849 | -0.7979 | 0.8401 | 0.9996 | Homeobox protein abdominal-A homolog [Source:UniProtKB/Swiss-Prot;Acc:P29552] |
| AAEL012782 | | 0.0855 | 4.1968 | 0.8403 | 0.9996 | |
| AAEL008662 | | 0.3240 | 1.0209 | 0.8403 | 0.9996 | mixed-lineage leukemia protein [Source:VB Community Annotation] |
| AAEL019972 | NA | -0.0810 | 5.8062 | 0.8404 | 0.9996 | NA |
| AAEL009499 | | 0.0904 | 2.1060 | 0.8404 | 0.9996 | |
| AAEL004107 | | 0.0561 | 5.4609 | 0.8405 | 0.9996 | nucleoside diphosphate kinase, putative [Source:VB Community Annotation] |
| AAEL009629 | | -0.0763 | 9.9841 | 0.8406 | 0.9996 | endoU protein, putative [Source:VB Community Annotation] |
| AAEL010248 | | 0.1321 | 4.5410 | 0.8407 | 0.9996 | fibrillarin [Source:VB Community Annotation] |
| AAEL019609 | NA | -0.2252 | 4.8572 | 0.8407 | 0.9996 | NA |
| AAEL010370 | | -0.1310 | 2.6330 | 0.8408 | 0.9996 | aldehyde oxidase [Source:VB Community Annotation] |
| AAEL023251 | NA | 0.2284 | -0.0154 | 0.8409 | 0.9996 | NA |
| AAEL026843 | NA | 0.2139 | 4.9714 | 0.8409 | 0.9996 | NA |
| AAEL001856 | | -0.0503 | 5.4017 | 0.8410 | 0.9996 | adenosine kinase [Source:VB Community Annotation] |
| AAEL007713 | | 0.0574 | 4.2633 | 0.8410 | 0.9996 | viral IAP-associated factor, putative [Source:VB Community Annotation] |
| AAEL009667 | | -0.1470 | 4.6049 | 0.8411 | 0.9996 | |
| AAEL016212 | tRNA-Lys | -0.1017 | 1.9794 | 0.8411 | 0.9996 | |
| AAEL005170 | | -0.0684 | 11.3311 | 0.8412 | 0.9996 | cytochrome c oxidase subunit iv [Source:VB Community Annotation |
| AAEL022631 | NA | 0.1307 | 3.9427 | 0.8413 | 0.9996 | NA |
| AAEL001593 | | -0.0987 | 6.7197 | 0.8413 | 0.9996 | Glycerol-3-phosphate dehydrogenase [NAD(+)] [Source:UniProtKB/TrEMBL;Acc:Q17KS4] |
| AAEL022551 | NA | 0.3668 | -0.6846 | 0.8414 | 0.9996 | NA |
| AAEL014141 | SRPN5 | 0.1320 | 3.9543 | 0.8416 | 0.9996 | Serine Protease Inhibitor (serpin) likely cleavage at S/M. [Source:VB Community Annotation] |
| AAEL019715 | NA | 0.2935 | 2.1345 | 0.8416 | 0.9996 | NA |

| | | | | | | |
|---|---|---|---|---|---|---|
| AAEL004141 | | 0.0482 | 7.3413 | 0.8418 | 0.9996 | phosphatidylinositol transfer protein/retinal degeneration b protein [Source:VB Community Annotation] |
| AAEL009850 | GALE14 | -0.2020 | -0.3265 | 0.8418 | 0.9996 | Galectin [Source:UniProtKB/TrEMBL;Acc:Q16UP0] |
| AAEL004569 | | 0.0699 | 6.6247 | 0.8418 | 0.9996 | |
| AAEL003394 | | -0.1061 | 3.7373 | 0.8419 | 0.9996 | |
| AAEL001090 | GSTD7 | -0.0906 | 5.7274 | 0.8419 | 0.9996 | glutathione S-transferase (GSTD7) [Source:VB Community Annotation] |
| AAEL011564 | | -0.0555 | 7.5190 | 0.8419 | 0.9996 | choline-phosphate cytidylyltransferase a, b [Source:VB Community Annotation] |
| AAEL027699 | NA | -0.3923 | 1.4336 | 0.8420 | 0.9996 | NA |
| AAEL004055 | | 0.1106 | 2.3199 | 0.8422 | 0.9996 | |
| AAEL005336 | | -0.0813 | 7.6421 | 0.8422 | 0.9996 | d-3-phosphoglycerate dehydrogenase [Source:VB Community Annotation] |
| AAEL007939 | | -0.0962 | 1.8526 | 0.8425 | 0.9996 | |
| AAEL025460 | NA | 0.1293 | 3.8323 | 0.8426 | 0.9996 | NA |
| AAEL024468 | NA | -0.2679 | -2.6242 | 0.8426 | 0.9996 | NA |
| AAEL002141 | | -0.0691 | 6.0615 | 0.8426 | 0.9996 | endothelin-converting enzyme [Source:VB Community Annotation] |
| AAEL010513 | | -0.3071 | 0.8847 | 0.8427 | 0.9996 | class b basic helix-loop-helix protein (bhlhb) (differentially expressed in chondrocytes) (mdec) (sharp) [Source:VB Community Annotation |
| AAEL005476 | | 0.1966 | 1.1493 | 0.8427 | 0.9996 | |
| AAEL008569 | | 0.0483 | 5.2743 | 0.8428 | 0.9996 | |
| AAEL006703 | | -0.2816 | 0.5296 | 0.8428 | 0.9996 | lumbrokinase-3(1) precursor, putative [Source:VB Community Annotation] |
| AAEL013724 | | 0.0643 | 3.5565 | 0.8429 | 0.9996 | |
| AAEL022990 | NA | 0.0668 | 3.3372 | 0.8430 | 0.9996 | NA |
| AAEL009474 | PGRPS1 | 0.1733 | 5.2456 | 0.8430 | 0.9996 | Peptidoglycan Recognition Protein (Short) [Source:VB Community Annotation] |
| AAEL005040 | | 0.1185 | 4.1467 | 0.8431 | 0.9996 | E3 ubiquitin-protein ligase [Source:UniProtKB/TrEMBL;Acc:Q17BB8] |
| AAEL005356 | | -0.2007 | 5.3185 | 0.8432 | 0.9996 | poly a polymerase [Source:VB Community Annotation] |
| AAEL025216 | NA | 0.0938 | 4.7980 | 0.8433 | 0.9996 | NA |
| AAEL021977 | NA | 0.1616 | 0.6021 | 0.8435 | 0.9996 | NA |
| AAEL018173 | | 0.1629 | 0.3726 | 0.8435 | 0.9996 | |
| AAEL018119 | | -0.0558 | 4.9892 | 0.8436 | 0.9996 | |
| AAEL003182 | SRPN26 | -0.3430 | 7.1838 | 0.8436 | 0.9996 | Serine Protease Inhibitor (serpin) homologue - unlikely to be inhibitory. [Source:VB Community Annotation] |
| AAEL000256 | SCRB9 | -0.2059 | 4.5974 | 0.8436 | 0.9996 | Class B Scavenger Receptor (CD36 domain). [Source:VB Community Annotation |
| AAEL001343 | | 0.3822 | 0.6804 | 0.8437 | 0.9996 | |
| AAEL008303 | | -0.0926 | 8.0789 | 0.8437 | 0.9996 | calponin/transgelin [Source:VB Community Annotation] |
| AAEL022241 | NA | -0.3824 | -0.6151 | 0.8437 | 0.9996 | NA |
| AAEL012475 | | -0.0459 | 4.7710 | 0.8439 | 0.9996 | jnk/sapk-associated protein [Source:VB Community Annotation] |
| AAEL005147 | | 0.0613 | 4.9050 | 0.8440 | 0.9996 | |
| AAEL000654 | | 0.0662 | 4.3163 | 0.8440 | 0.9996 | n-acetylgalactosaminyltransferase [Source:VB Community Annotation] |
| AAEL002549 | sosie | 0.0819 | 4.7942 | 0.8440 | 0.9996 | protein sosie [Source:VB Community Annotation] |
| AAEL006400 | | 0.0431 | 4.9604 | 0.8440 | 0.9996 | elongator component, putative [Source:VB Community Annotation] |
| AAEL007187 | | -0.1908 | 2.8344 | 0.8441 | 0.9996 | |
| AAEL021510 | NA | -0.1472 | 2.9752 | 0.8442 | 0.9996 | NA |
| AAEL013209 | | 0.1023 | 1.4989 | 0.8442 | 0.9996 | |
| AAEL009381 | | -0.3210 | 3.0598 | 0.8442 | 0.9996 | |

| | | | | | | |
|---|---|---|---|---|---|---|
| AAEL022234 | NA | -0.2762 | -0.9031 | 0.8443 | 0.9996 | NA |
| AAEL018231 | | 0.0620 | 4.5046 | 0.8443 | 0.9996 | |
| AAEL017547 | | -0.1883 | -0.4802 | 0.8443 | 0.9996 | |
| AAEL005160 | | 0.2761 | -0.9682 | 0.8445 | 0.9996 | sulphate transporter [Source:VB Community Annotation] |
| AAEL021452 | NA | -0.0862 | 2.4687 | 0.8445 | 0.9996 | NA |
| AAEL020775 | NA | -0.1690 | 0.8953 | 0.8445 | 0.9996 | NA |
| AAEL015411 | | 0.1105 | 1.7833 | 0.8446 | 0.9996 | Glutamine-dependent NAD(+) synthetase [Source:UniProtKB/TrEMBL;Acc:Q16E59] |
| AAEL021728 | NA | -0.0641 | 4.7007 | 0.8446 | 0.9996 | NA |
| AAEL015099 | | 0.3374 | -1.2683 | 0.8447 | 0.9996 | sumo ligase [Source:VB Community Annotation] |
| AAEL001000 | | 0.1083 | 5.8322 | 0.8447 | 0.9996 | 1-acyl-sn-glycerol-3-phosphate acyltransferase [Source:VB Community Annotation] |
| AAEL014505 | | 0.0709 | 9.2637 | 0.8447 | 0.9996 | succinate dehydrogenase, putative [Source:VB Community Annotation] |
| AAEL005482 | CTL18 | -0.2553 | 5.4140 | 0.8448 | 0.9996 | C-Type Lectin (CTL). [Source:VB Community Annotation] |
| AAEL001831 | | -0.0776 | 4.7946 | 0.8449 | 0.9996 | DNA-directed RNA polymerase II [Source:VB Community Annotation] |
| AAEL000378 | | 0.1245 | 2.9818 | 0.8449 | 0.9996 | integrin-linked protein kinase 2 (ilk-2) [Source:VB Community Annotation] |
| AAEL020186 | NA | 0.0736 | 3.9772 | 0.8449 | 0.9996 | NA |
| AAEL013406 | | 0.2523 | -1.5133 | 0.8450 | 0.9996 | venom allergen [Source:VB Community Annotation] |
| AAEL025525 | NA | 0.1155 | 1.7737 | 0.8452 | 0.9996 | NA |
| AAEL027439 | NA | 0.3528 | 4.4238 | 0.8453 | 0.9996 | NA |
| AAEL005064 | CLIPB5 | -0.1125 | 7.4634 | 0.8454 | 0.9996 | Clip-Domain Serine Protease  family B. [Source:VB Community Annotation] |
| AAEL026634 | NA | -0.3289 | 2.1308 | 0.8454 | 0.9996 | NA |
| AAEL021736 | NA | 0.0919 | 3.7293 | 0.8455 | 0.9996 | NA |
| AAEL020015 | NA | 0.0582 | 3.7546 | 0.8456 | 0.9996 | NA |
| AAEL027631 | NA | -0.1034 | 8.3718 | 0.8458 | 0.9996 | NA |
| AAEL008039 | | -0.1119 | 4.4115 | 0.8459 | 0.9996 | |
| AAEL001080 | | -0.1038 | 3.5184 | 0.8460 | 0.9996 | RNA 3' terminal phosphate cyclase [Source:VB Community Annotation] |
| AAEL003697 | SRPN17 | -0.0794 | 6.3216 | 0.8460 | 0.9996 | Serine Protease Inhibitor (serpin) homologue - unlikely to be inhibitory. [Source:VB Community Annotation] |
| AAEL010671 | | -0.0624 | 4.0879 | 0.8460 | 0.9996 | oxidoreductase [Source:VB Community Annotation] |
| AAEL011053 | | -0.0842 | 2.3145 | 0.8464 | 0.9996 | |
| AAEL002617 | OBP12 | -0.1059 | 7.9134 | 0.8464 | 0.9996 | odorant binding protein OBP12 [Source:VB Community Annotation] |
| AAEL001710 | | 0.2761 | -1.4809 | 0.8464 | 0.9996 | |
| AAEL008551 | | 0.0639 | 5.7461 | 0.8464 | 0.9996 | |
| AAEL021693 | NA | -0.3018 | -1.5054 | 0.8465 | 0.9996 | NA |
| AAEL007602 | | 0.1996 | -1.2043 | 0.8466 | 0.9996 | trypsin, putative [Source:VB Community Annotation] |
| AAEL013277 | | -0.2914 | 3.7762 | 0.8466 | 0.9996 | voltage-gated sodium channel [Source:VB Community Annotation] |
| AAEL006740 | | 0.0506 | 4.7031 | 0.8466 | 0.9996 | |
| AAEL009700 | | 0.1218 | 5.2677 | 0.8467 | 0.9996 | beta lactamase domain [Source:VB Community Annotation] |
| AAEL002161 | | -0.0683 | 5.4832 | 0.8468 | 0.9996 | UPF0184 protein AAEL002161 [Source:UniProtKB/Swiss-Prot;Acc:Q17J49] |
| AAEL011986 | | -0.2749 | -0.4460 | 0.8468 | 0.9996 | |
| AAEL025633 | NA | 0.2914 | -1.6688 | 0.8469 | 0.9996 | NA |
| AAEL027494 | NA | -0.1177 | 4.7312 | 0.8469 | 0.9996 | NA |

| | | | | | | |
|---|---|---|---|---|---|---|
| AAEL003529 | | -0.0702 | 3.6015 | 0.8470 | 0.9996 | zinc finger protein [Source:VB Community Annotation] |
| AAEL026356 | NA | 0.1853 | 1.1729 | 0.8471 | 0.9996 | NA |
| AAEL023002 | NA | -0.2020 | -1.7475 | 0.8471 | 0.9996 | NA |
| AAEL027239 | NA | -0.0763 | 2.1253 | 0.8471 | 0.9996 | NA |
| AAEL006317 | | 0.0508 | 6.1066 | 0.8471 | 0.9996 | short-chain dehydrogenase [Source:VB Community Annotation] |
| AAEL017029 | | -0.1545 | 7.3181 | 0.8472 | 0.9996 | |
| AAEL013172 | | 0.0919 | 3.5404 | 0.8474 | 0.9996 | zinc finger protein [Source:VB Community Annotation] |
| AAEL013407 | Cat | -0.0688 | 7.6196 | 0.8475 | 0.9996 | Catalase [Source:UniProtKB/TrEMBL;Acc:Q16J86] |
| AAEL006026 | | -0.1110 | 4.5639 | 0.8476 | 0.9996 | leucine rich protein, putative [Source:VB Community Annotation] |
| AAEL024346 | NA | 0.1119 | 3.8485 | 0.8477 | 0.9996 | NA |
| AAEL019534 | NA | 0.1034 | 2.5067 | 0.8479 | 0.9996 | NA |
| AAEL000605 | | -0.0593 | 4.5352 | 0.8479 | 0.9996 | rab geranylgeranyl transferase alpha subunit [Source:VB Community Annotation] |
| AAEL011973 | | -0.1093 | 7.0145 | 0.8480 | 0.9996 | fumarylacetoacetate hydrolase [Source:VB Community Annotation] |
| AAEL022076 | NA | 0.1355 | 2.3105 | 0.8482 | 0.9996 | NA |
| AAEL002440 | | -0.2830 | -1.5777 | 0.8482 | 0.9996 | larval cuticle protein, putative [Source:VB Community Annotation] |
| AAEL007445 | | -0.0969 | 1.5735 | 0.8483 | 0.9996 | |
| AAEL006712 | | 0.1819 | -0.4504 | 0.8483 | 0.9996 | serine/threonine protein kinase [Source:VB Community Annotation] |
| AAEL006822 | | -0.1883 | 5.7154 | 0.8484 | 0.9996 | otopetrin [Source:VB Community Annotation] |
| AAEL009213 | | 0.1002 | 2.4478 | 0.8485 | 0.9996 | |
| AAEL003019 | | 0.0705 | 5.6293 | 0.8485 | 0.9996 | |
| AAEL003530 | RpLP1 | 0.0582 | 11.2247 | 0.8486 | 0.9996 | acidic ribosomal protein P1, putative [Source:VB Community Annotation] |
| AAEL003532 | | -0.2185 | 5.1508 | 0.8487 | 0.9996 | |
| AAEL000867 | | -0.3050 | 1.7806 | 0.8487 | 0.9996 | F-box protein [Source:VB Community Annotation] |
| AAEL004131 | | -0.0958 | 4.8204 | 0.8488 | 0.9996 | gh regulated tbc protein-1 [Source:VB Community Annotation] |
| AAEL024923 | NA | -0.1086 | 3.2547 | 0.8488 | 0.9996 | NA |
| AAEL005143 | | -0.0793 | 9.7382 | 0.8489 | 0.9996 | ubiquinone binding protein, putative [Source:VB Community Annotation] |
| AAEL019798 | NA | -0.1097 | 8.3983 | 0.8490 | 0.9996 | NA |
| AAEL006652 | | 0.0829 | 6.6671 | 0.8490 | 0.9996 | |
| AAEL008481 | RpL18 | 0.0515 | 11.8583 | 0.8492 | 0.9996 | 60S ribosomal protein L18 [Source:UniProtKB/Swiss-Prot;Acc:Q1HR62] |
| AAEL009951 | | 0.1545 | 3.7304 | 0.8492 | 0.9996 | dimeric dihydrodiol dehydrogenase [Source:VB Community Annotation] |
| AAEL001819 | | -0.3422 | 0.3523 | 0.8493 | 0.9996 | |
| AAEL002056 | | -0.0659 | 2.1234 | 0.8494 | 0.9996 | Cytoplasmic tRNA 2-thiolation protein 2 [Source:UniProtKB/Swiss-Prot;Acc:Q17JB7] |
| AAEL000993 | | -0.2441 | 3.8785 | 0.8494 | 0.9996 | dally [Source:VB Community Annotation] |
| AAEL002890 | | -0.0466 | 4.6393 | 0.8494 | 0.9996 | |
| AAEL027135 | NA | -0.1572 | 2.0788 | 0.8494 | 0.9996 | NA |
| AAEL024623 | NA | -0.1417 | 0.4193 | 0.8495 | 0.9996 | NA |
| AAEL027814 | NA | 0.2032 | -0.4544 | 0.8498 | 0.9996 | NA |
| AAEL008392 | | -0.0952 | 2.4149 | 0.8499 | 0.9996 | |
| AAEL011458 | | 0.1674 | 0.9763 | 0.8499 | 0.9996 | |
| AAEL005754 | | -0.0714 | 2.7924 | 0.8500 | 0.9996 | cgmp-dependent protein kinase [Source:VB Community Annotation |

| | | | | | | |
|---|---|---|---|---|---|---|
| AAEL009604 | | -0.0731 | 8.2984 | 0.8501 | 0.9996 | Receptor expression-enhancing protein [Source:UniProtKB/TrEMBL;Acc:Q16VD7] |
| AAEL015607 | | -0.0498 | 4.3324 | 0.8501 | 0.9996 | |
| AAEL022855 | NA | 0.0635 | 3.5378 | 0.8501 | 0.9996 | NA |
| AAEL011150 | Sxl | 0.0877 | 4.4270 | 0.8505 | 0.9996 | sex-lethal [Source:VB Community Annotation] |
| AAEL007514 | | 0.2346 | -1.2413 | 0.8506 | 0.9996 | oviductin [Source:VB Community Annotation] |
| AAEL009642 | | 0.1625 | 3.3868 | 0.8507 | 0.9996 | cathepsin b [Source:VB Community Annotation] |
| AAEL009753 | | 0.1118 | 2.0497 | 0.8508 | 0.9996 | sodium-dependent phosphate transporter [Source:VB Community Annotation] |
| AAEL006043 | | 0.0624 | 3.1117 | 0.8508 | 0.9996 | gc-rich sequence DNA-binding factor [Source:VB Community Annotation] |
| AAEL027322 | NA | -0.0544 | 3.8130 | 0.8508 | 0.9996 | NA |
| AAEL005084 | | -0.1218 | 4.9498 | 0.8508 | 0.9996 | tubulin beta chain [Source:VB Community Annotation] |
| AAEL013501 | PPO4 | 0.1587 | 2.7585 | 0.8509 | 0.9996 | prophenoloxidase [Source:VB Community Annotation] |
| AAEL003491 | | 0.0653 | 5.8660 | 0.8509 | 0.9996 | proline synthetase associated protein [Source:VB Community Annotation] |
| AAEL009634 | | 0.0689 | 6.6653 | 0.8509 | 0.9996 | steroid dehydrogenase [Source:VB Community Annotation] |
| AAEL004313 | | -0.1347 | 5.7945 | 0.8509 | 0.9996 | fk506-binding protein [Source:VB Community Annotation] |
| AAEL009179 | | 0.1206 | 2.7589 | 0.8511 | 0.9996 | molybdopterin biosynthesis protein [Source:VB Community Annotation] |
| AAEL003299 | | -0.1560 | 7.3974 | 0.8511 | 0.9996 | |
| AAEL000076 | | -0.0670 | 3.4333 | 0.8512 | 0.9996 | dimethyladenosine transferase [Source:VB Community Annotation] |
| AAEL005849 | | 0.1210 | 3.9907 | 0.8513 | 0.9996 | synaptic vesicle protein [Source:VB Community Annotation] |
| AAEL004805 | | -0.0745 | 6.5395 | 0.8514 | 0.9996 | potassium-dependent sodium-calcium exchanger, putative [Source:VB Community Annotation] |
| AAEL006160 | | 0.0803 | 2.5450 | 0.8514 | 0.9996 | runx1 (aml1) [Source:VB Community Annotation] |
| AAEL019418 | NA | 0.1888 | 3.6888 | 0.8515 | 0.9996 | NA |
| AAEL008743 | | 0.0633 | 3.3076 | 0.8516 | 0.9996 | tetratricopeptide repeat protein, putative [Source:VB Community Annotation] |
| AAEL009551 | TOLL11 | -0.2793 | 1.8350 | 0.8516 | 0.9996 | Toll-like receptor [Source:VB Community Annotation] |
| AAEL010132 | LRIM3 | -0.1111 | 2.8634 | 0.8517 | 0.9996 | leucine-rich immune protein (Long) [Source:VB Community Annotation] |
| AAEL004098 | | 0.0534 | 5.8889 | 0.8520 | 0.9996 | neuromusculin [Source:VB Community Annotation] |
| AAEL001084 | CLIPB21 | 0.1145 | 2.9669 | 0.8520 | 0.9996 | Clip-Domain Serine Protease family B. Protease homologue. [Source:VB Community Annotation] |
| AAEL025017 | NA | 0.0410 | 5.6923 | 0.8520 | 0.9996 | NA |
| AAEL019515 | NA | -0.0889 | 2.5948 | 0.8522 | 0.9996 | NA |
| AAEL004419 | | -0.0484 | 5.5303 | 0.8523 | 0.9996 | ATP-dependent RNA helicase [Source:VB Community Annotation] |
| AAEL001543 | | 0.2162 | -0.4954 | 0.8523 | 0.9996 | |
| AAEL005454 | | 0.1073 | 4.3940 | 0.8524 | 0.9996 | AAA ATPase [Source:VB Community Annotation] |
| AAEL012476 | | 0.0591 | 5.0799 | 0.8524 | 0.9996 | |
| AAEL001102 | | -0.0728 | 6.5176 | 0.8524 | 0.9996 | adenosine kinase [Source:VB Community Annotation] |
| AAEL021545 | NA | 0.2997 | -1.1381 | 0.8525 | 0.9996 | NA |
| AAEL021785 | NA | 0.2041 | 0.3887 | 0.8526 | 0.9996 | NA |
| AAEL000519 | | -0.2283 | 3.9356 | 0.8527 | 0.9996 | |
| AAEL020007 | NA | -0.2080 | -1.2536 | 0.8528 | 0.9996 | NA |
| AAEL018876 | tRNA-Val | -0.1310 | 1.0097 | 0.8529 | 0.9996 | |
| AAEL001749 | | 0.3001 | 2.9601 | 0.8529 | 0.9996 | ventrhoid transmembrane protein, putative [Source:VB Community Annotation] |
| AAEL004423 | | -0.0641 | 10.1386 | 0.8530 | 0.9996 | mitochondrial F0 ATP synthase D chain, putative [Source:VB Community Annotation] |

| | | | | | | |
|---|---|---|---|---|---|---|
| AAEL019556 | NA | 0.0769 | 5.4488 | 0.8530 | 0.9996 | NA |
| AAEL007963 | | -0.0874 | 5.7091 | 0.8530 | 0.9996 | |
| AAEL011274 | | -0.0586 | 6.2023 | 0.8530 | 0.9996 | 1-phosphatidylinositol-4-phosphate 5-kinase, putative [Source:VB Community Annotation] |
| AAEL011263 | | -0.1186 | 6.9076 | 0.8532 | 0.9996 | phosphatidylethanolamine-binding protein [Source:VB Community Annotation] |
| AAEL019577 | NA | 0.0845 | 3.9220 | 0.8533 | 0.9996 | NA |
| AAEL019528 | NA | 0.0615 | 4.1745 | 0.8536 | 0.9996 | NA |
| AAEL003456 | | -0.0748 | 2.7923 | 0.8537 | 0.9996 | |
| AAEL004689 | | 0.0533 | 3.7891 | 0.8539 | 0.9996 | |
| AAEL024517 | NA | 0.0926 | 1.3307 | 0.8539 | 0.9996 | NA |
| AAEL006087 | | -0.0499 | 4.6867 | 0.8539 | 0.9996 | |
| AAEL012052 | | 0.0773 | 5.5575 | 0.8542 | 0.9996 | |
| AAEL001281 | | -0.0885 | 6.8986 | 0.8542 | 0.9996 | |
| AAEL011283 | | -0.0676 | 3.1680 | 0.8544 | 0.9996 | Cytoplasmic tRNA 2-thiolation protein 1 (EC 2.7.7.-)(Cytoplasmic tRNA adenylyltransferase 1) [Source:VB Community Annotation] |
| AAEL006848 | | 0.2063 | 3.0494 | 0.8544 | 0.9996 | |
| AAEL009872 | | -0.0702 | 7.5120 | 0.8545 | 0.9996 | alanine aminotransferase [Source:VB Community Annotation] |
| AAEL021173 | NA | 0.2189 | -1.2428 | 0.8546 | 0.9996 | NA |
| AAEL025578 | NA | 0.0528 | 5.5330 | 0.8546 | 0.9996 | NA |
| AAEL002800 | | -0.1237 | 3.0529 | 0.8548 | 0.9996 | DNA polymerase epsilon, catalytic subunit [Source:VB Community Annotation] |
| AAEL014171 | | 0.1003 | 3.6346 | 0.8548 | 0.9996 | |
| AAEL000596 | | 0.1083 | 9.6491 | 0.8548 | 0.9996 | myosin [Source:VB Community Annotation] |
| AAEL024174 | NA | 0.1636 | -0.4854 | 0.8549 | 0.9996 | NA |
| AAEL000938 | | 0.0637 | 3.1216 | 0.8549 | 0.9996 | |
| AAEL006549 | | 0.0791 | 3.6974 | 0.8550 | 0.9996 | |
| AAEL027067 | NA | -0.1608 | 1.6219 | 0.8550 | 0.9996 | NA |
| AAEL003847 | | 0.0641 | 3.7843 | 0.8550 | 0.9996 | |
| AAEL005130 | | -0.1860 | 3.9538 | 0.8551 | 0.9996 | zinc finger protein [Source:VB Community Annotation] |
| AAEL004870 | CYP18A1 | 0.2885 | -0.9348 | 0.8552 | 0.9996 | cytochrome P450 [Source:VB Community Annotation] |
| AAEL003822 | | -0.0750 | 4.0169 | 0.8552 | 0.9996 | |
| AAEL010190 | | 0.1269 | 3.3049 | 0.8553 | 0.9996 | |
| AAEL002263 | | 0.3263 | 3.3098 | 0.8553 | 0.9996 | |
| AAEL002771 | | 0.0621 | 4.7113 | 0.8554 | 0.9996 | microtubule binding protein, putative [Source:VB Community Annotation] |
| AAEL011818 | | -0.1002 | 3.9158 | 0.8555 | 0.9996 | |
| AAEL000973 | | 0.3276 | 0.3821 | 0.8556 | 0.9996 | |
| AAEL000439 | | 0.0905 | 0.9540 | 0.8557 | 0.9996 | |
| AAEL026304 | NA | 0.3613 | 7.7550 | 0.8557 | 0.9996 | NA |
| AAEL022809 | NA | -0.4172 | 0.0657 | 0.8557 | 0.9996 | NA |
| AAEL010938 | | 0.0692 | 2.1214 | 0.8558 | 0.9996 | l-asparaginase [Source:VB Community Annotation] |
| AAEL020745 | NA | -0.0619 | 2.8957 | 0.8558 | 0.9996 | NA |
| AAEL005190 | mRpL42 | -0.0602 | 5.4896 | 0.8559 | 0.9996 | mitochondrial ribosomal protein, L42, putative [Source:VB Community Annotation] |
| AAEL012243 | | 0.0464 | 8.1100 | 0.8559 | 0.9996 | |

| | | | | | | |
|---|---|---|---|---|---|---|
| AAEL014340 | | 0.0807 | 1.9494 | 0.8560 | 0.9996 | |
| AAEL002380 | Gr1 | 0.1327 | 1.4631 | 0.8561 | 0.9996 | gustatory receptor (Gr1) [Source:VB Community Annotation] |
| AAEL006919 | | -0.2558 | -1.4396 | 0.8561 | 0.9996 | serine-type enodpeptidase, [Source:VB Community Annotation] |
| AAEL015003 | | -0.0919 | 2.8539 | 0.8561 | 0.9996 | |
| AAEL017335 | GPRGHP2 | -0.3006 | -0.3743 | 0.8562 | 0.9996 | GPCR Growth Hormone Releasing Hormone Family [Source:VB Community Annotation] |
| AAEL006968 | | -0.1486 | 9.1124 | 0.8564 | 0.9996 | |
| AAEL003285 | TRAM | -0.0532 | 6.5190 | 0.8564 | 0.9996 | translocation associated membrane protein [Source:VB Community Annotation] |
| AAEL006360 | | 0.1833 | 2.3474 | 0.8564 | 0.9996 | |
| AAEL006012 | | -0.2344 | 2.7767 | 0.8566 | 0.9996 | factor for adipocyte differentiation [Source:VB Community Annotation] |
| AAEL019468 | NA | -0.1136 | 4.6538 | 0.8566 | 0.9996 | NA |
| AAEL007184 | | -0.0392 | 6.7080 | 0.8567 | 0.9996 | vacuolar ATP synthase subunit g [Source:VB Community Annotation] |
| AAEL007592 | | -0.2318 | -1.4502 | 0.8568 | 0.9996 | |
| AAEL004646 | | 0.2814 | -0.9929 | 0.8568 | 0.9996 | actin [Source:VB Community Annotation] |
| AAEL025520 | NA | 0.1402 | 3.2318 | 0.8570 | 0.9996 | NA |
| AAEL023591 | NA | 0.1161 | 4.5437 | 0.8572 | 0.9996 | NA |
| AAEL019988 | NA | -0.0677 | 5.8947 | 0.8573 | 0.9996 | NA |
| AAEL000897 | | -0.1227 | 5.6196 | 0.8574 | 0.9996 | |
| AAEL007877 | | 0.0748 | 3.9225 | 0.8574 | 0.9996 | |
| AAEL005131 | | -0.2133 | 4.9030 | 0.8575 | 0.9996 | protein kinase c-binding protein nell1 [Source:VB Community Annotation] |
| AAEL021862 | NA | -0.1899 | -0.6302 | 0.8575 | 0.9996 | NA |
| AAEL011836 | | -0.2312 | -1.1153 | 0.8576 | 0.9996 | |
| AAEL001897 | | -0.2128 | 3.3826 | 0.8577 | 0.9996 | |
| AAEL013504 | | 0.2237 | -0.0834 | 0.8578 | 0.9996 | T-box transcription factor tbx6 [Source:VB Community Annotation] |
| AAEL012556 | | -0.1663 | 2.3133 | 0.8578 | 0.9996 | Ofd1 protein, putative [Source:VB Community Annotation] |
| AAEL013722 | | -0.2277 | -1.5170 | 0.8579 | 0.9996 | partner of burs, putative [Source:VB Community Annotation] |
| AAEL025552 | NA | -0.2775 | 4.8605 | 0.8582 | 0.9996 | NA |
| AAEL004407 | | 0.2058 | 0.4185 | 0.8582 | 0.9996 | allergen, putative [Source:VB Community Annotation] |
| AAEL001734 | | -0.2111 | 4.1889 | 0.8583 | 0.9996 | bric-a-brac [Source:VB Community Annotation] |
| AAEL024560 | NA | -0.2332 | 3.0232 | 0.8583 | 0.9996 | NA |
| AAEL000788 | | 0.1621 | 4.5893 | 0.8583 | 0.9996 | matrix metalloproteinase [Source:VB Community Annotation] |
| AAEL003944 | | -0.0729 | 3.3568 | 0.8583 | 0.9996 | |
| AAEL026060 | NA | -0.1396 | 0.6250 | 0.8584 | 0.9996 | NA |
| AAEL000301 | | 0.0789 | 6.5130 | 0.8584 | 0.9996 | heat shock protein [Source:VB Community Annotation] |
| AAEL017272 | GPR5HT3 | -0.2941 | 1.4021 | 0.8585 | 0.9996 | GPCR Serotonin Family [Source:VB Community Annotation] |
| AAEL003116 | | 0.1087 | 14.6469 | 0.8586 | 0.9996 | phosrestin i (arrestin b) (arrestin 2) [Source:VB Community Annotation] |
| AAEL008948 | | -0.1990 | -2.7133 | 0.8587 | 0.9996 | |
| AAEL007680 | | 0.0700 | 4.2420 | 0.8588 | 0.9996 | |
| AAEL002676 | | 0.2056 | -0.1383 | 0.8589 | 0.9996 | angiotensin-converting enzyme [Source:VB Community Annotation] |
| AAEL008466 | | 0.0801 | 2.7373 | 0.8589 | 0.9996 | homeobox protein b [Source:VB Community Annotation] |
| AAEL019719 | NA | -0.0886 | 10.4612 | 0.8590 | 0.9996 | NA |

| | | | | | | |
|---|---|---|---|---|---|---|
| AAEL026846 | NA | 0.2674 | 2.5183 | 0.8590 | 0.9996 | NA |
| AAEL011080 | | 0.1366 | 0.8979 | 0.8592 | 0.9996 | intraflagellar transport 80 homolog (WD-repeat protein 56, putative) [Source:VB Community Annotation] |
| AAEL007838 | | 0.0511 | 6.2121 | 0.8593 | 0.9996 | |
| AAEL001308 | | 0.0466 | 7.7413 | 0.8595 | 0.9996 | CRAL/TRIO domain-containing protein [Source:VB Community Annotation] |
| AAEL026244 | NA | -0.1182 | 0.7704 | 0.8597 | 0.9996 | NA |
| AAEL021658 | NA | -0.1665 | 1.6604 | 0.8597 | 0.9996 | NA |
| AAEL000294 | | 0.1483 | 3.0861 | 0.8597 | 0.9996 | |
| AAEL022183 | NA | -0.0454 | 5.3798 | 0.8600 | 0.9996 | NA |
| AAEL010263 | | -0.1079 | 2.7115 | 0.8600 | 0.9996 | |
| AAEL001350 | | 0.0706 | 4.1120 | 0.8601 | 0.9996 | |
| AAEL013962 | | 0.0761 | 2.4437 | 0.8602 | 0.9996 | mutagen-sensitive, putative [Source:VB Community Annotation] |
| AAEL010635 | | 0.0746 | 2.5517 | 0.8602 | 0.9996 | zinc finger protein [Source:VB Community Annotation] |
| AAEL007006 | CLIPA17 | -0.1235 | 4.2519 | 0.8603 | 0.9996 | Clip-Domain Serine Protease  family A. [Source:VB Community Annotation] |
| AAEL003540 | | 0.0768 | 3.2969 | 0.8604 | 0.9996 | |
| AAEL007146 | | 0.0559 | 3.3822 | 0.8605 | 0.9996 | voltage-gated potassium channel [Source:VB Community Annotation] |
| AAEL004278 | | -0.1248 | 6.8384 | 0.8607 | 0.9996 | |
| AAEL003346 | | 0.0920 | 3.3016 | 0.8608 | 0.9996 | heparan sulphate 2-o-sulfotransferase [Source:VB Community Annotation] |
| AAEL013639 | | -0.0598 | 3.4715 | 0.8609 | 0.9996 | |
| AAEL023757 | NA | -0.1267 | 1.9754 | 0.8610 | 0.9996 | NA |
| AAEL027408 | NA | 0.0678 | 7.4299 | 0.8610 | 0.9996 | NA |
| AAEL024675 | NA | -0.1156 | 4.3997 | 0.8611 | 0.9996 | NA |
| AAEL019745 | NA | -0.0732 | 5.3699 | 0.8612 | 0.9996 | NA |
| AAEL013483 | | 0.0725 | 3.3057 | 0.8613 | 0.9996 | short-chain dehydrogenase [Source:VB Community Annotation] |
| AAEL019854 | NA | 0.0991 | 4.0707 | 0.8613 | 0.9996 | NA |
| AAEL011293 | | 0.0600 | 6.0806 | 0.8614 | 0.9996 | vitamin-K-epoxide reductase (warfarin-sensitive), putative [Source:VB Community Annotation] |
| AAEL026276 | NA | -0.1360 | 4.7569 | 0.8614 | 0.9996 | NA |
| AAEL009257 | | -0.0615 | 10.5911 | 0.8616 | 0.9996 | |
| AAEL017851 | U3 | -0.1167 | 2.8284 | 0.8618 | 0.9996 | Small nucleolar RNA U3 [Source:RFAM;Acc:RF00012] |
| AAEL025606 | NA | -0.1847 | 1.9470 | 0.8618 | 0.9996 | NA |
| AAEL009801 | | -0.1825 | -0.9723 | 0.8620 | 0.9996 | cuticle protein, putative [Source:VB Community Annotation] |
| AAEL021227 | NA | -0.3110 | 2.1623 | 0.8622 | 0.9996 | NA |
| AAEL023494 | NA | 0.0545 | 4.9750 | 0.8622 | 0.9996 | NA |
| AAEL000715 | | 0.1179 | 0.7370 | 0.8623 | 0.9996 | zinc finger protein [Source:VB Community Annotation] |
| AAEL004509 | | 0.1752 | 2.3481 | 0.8625 | 0.9996 | Kynurenine formamidase [Source:UniProtKB/TrEMBL;Acc:Q0IFP1] |
| AAEL005259 | | 0.2420 | 1.1064 | 0.8626 | 0.9996 | |
| AAEL027212 | NA | 0.0776 | 2.8795 | 0.8627 | 0.9996 | NA |
| AAEL011416 | | 0.0761 | 4.6512 | 0.8628 | 0.9996 | |
| AAEL010941 | | 0.2738 | 2.1366 | 0.8628 | 0.9996 | Protein tweety homolog [Source:UniProtKB/TrEMBL;Acc:Q16RK1] |
| AAEL009924 | | -0.1448 | -0.0790 | 0.8628 | 0.9996 | type II keratin, putative [Source:VB Community Annotation] |
| AAEL000064 | | -0.1227 | 5.6921 | 0.8629 | 0.9996 | dopachrome-conversion enzyme (DCE) isoenzyme, putative [Source:VB Community Annotation] |

| | | | | | | |
|---|---|---|---|---|---|---|
| AAEL009314 | | 0.0978 | 1.7678 | 0.8629 | 0.9996 | adenylate cyclase [Source:VB Community Annotation] |
| AAEL027157 | NA | -0.1137 | 5.4643 | 0.8630 | 0.9996 | NA |
| AAEL003825 | | -0.0519 | 4.1487 | 0.8630 | 0.9996 | |
| AAEL019851 | NA | 0.1314 | 5.4069 | 0.8630 | 0.9996 | NA |
| AAEL006033 | | -0.0722 | 4.3771 | 0.8631 | 0.9996 | Dihydroorotate dehydrogenase (quinone), mitochondrial [Source:UniProtKB/TrEMBL;Acc:Q177S6] |
| AAEL005951 | | -0.1190 | 6.9975 | 0.8632 | 0.9996 | lipid storage droplets surface binding protein [Source:VB Community Annotation] |
| AAEL019995 | NA | 0.0676 | 3.0490 | 0.8633 | 0.9996 | NA |
| AAEL026671 | NA | -0.0908 | 2.1515 | 0.8634 | 0.9996 | NA |
| AAEL009569 | | -0.0847 | 4.9630 | 0.8635 | 0.9996 | apolipoprotein D, putative [Source:VB Community Annotation] |
| AAEL007718 | eIF3-S9 | -0.0362 | 7.4655 | 0.8636 | 0.9996 | Eukaryotic translation initiation factor 3 subunit B (eIF3b)(Eukaryotic translation initiation factor 3 subunit 9) [Source:VB Community Ann |
| AAEL014511 | | -0.0938 | 7.8142 | 0.8636 | 0.9996 | |
| AAEL007033 | | -0.1747 | 5.3911 | 0.8636 | 0.9996 | Pyrroline-5-carboxylate reductase [Source:UniProtKB/TrEMBL;Acc:Q173T1] |
| AAEL000716 | | 0.0495 | 5.5061 | 0.8636 | 0.9996 | chondroitin 4-sulfotransferase [Source:VB Community Annotation] |
| AAEL000020 | | -0.0469 | 5.5616 | 0.8636 | 0.9996 | |
| AAEL023012 | NA | -0.2937 | 0.3571 | 0.8637 | 0.9996 | NA |
| AAEL020188 | NA | 0.2914 | -2.2460 | 0.8638 | 0.9996 | NA |
| AAEL008824 | | 0.2275 | 2.7115 | 0.8640 | 0.9996 | |
| AAEL008024 | | 0.0673 | 5.9494 | 0.8641 | 0.9996 | |
| AAEL026448 | NA | -0.0834 | 2.7618 | 0.8641 | 0.9996 | NA |
| AAEL006138 | | -0.4946 | -0.9175 | 0.8642 | 0.9996 | |
| AAEL005052 | | -0.0830 | 6.7924 | 0.8642 | 0.9996 | Tubulin beta chain [Source:UniProtKB/TrEMBL;Acc:Q17B46] |
| AAEL006910 | | -0.0489 | 5.5886 | 0.8643 | 0.9996 | ubiquitination factor E4 [Source:VB Community Annotation] |
| AAEL020592 | NA | -0.2265 | 1.1477 | 0.8644 | 0.9996 | NA |
| AAEL012258 | | 0.0467 | 4.9547 | 0.8645 | 0.9996 | platelet-activating factor acetylhydrolase ib [Source:VB Community Annotation] |
| AAEL013875 | | 0.0357 | 8.4399 | 0.8646 | 0.9996 | tetraspanin, putative [Source:VB Community Annotation] |
| AAEL013487 | | 0.1647 | 0.0439 | 0.8647 | 0.9996 | RHO guanyl-nucleotide exchange factor, putative [Source:VB Community Annotation] |
| AAEL009806 | | -0.0914 | 1.6943 | 0.8647 | 0.9996 | low-density lipoprotein receptor (ldl) [Source:VB Community Annotation] |
| AAEL021532 | NA | 0.1483 | 4.4809 | 0.8647 | 0.9996 | NA |
| AAEL021995 | NA | 0.3480 | 3.6617 | 0.8647 | 0.9996 | NA |
| AAEL022363 | NA | 0.1865 | -0.2183 | 0.8648 | 0.9996 | NA |
| AAEL006989 | CYP6AG7 | -0.0824 | 4.2801 | 0.8648 | 0.9996 | cytochrome P450 [Source:VB Community Annotation] |
| AAEL002246 | | -0.1038 | 2.3227 | 0.8648 | 0.9996 | cuticle protein, putative [Source:VB Community Annotation] |
| AAEL019966 | NA | -0.3549 | 0.9802 | 0.8648 | 0.9996 | NA |
| AAEL026237 | NA | 0.1818 | -0.8467 | 0.8650 | 0.9996 | NA |
| AAEL003273 | | 0.0616 | 4.3595 | 0.8651 | 0.9996 | cell division cycle [Source:VB Community Annotation] |
| AAEL027829 | NA | 0.1599 | 3.1729 | 0.8651 | 0.9996 | NA |
| AAEL005524 | | 0.0801 | 5.7724 | 0.8651 | 0.9996 | Adenosylhomocysteinase [Source:UniProtKB/TrEMBL;Acc:Q179S1] |
| AAEL002417 | | -0.2542 | 3.6635 | 0.8651 | 0.9996 | troponin t, invertebrate [Source:VB Community Annotation] |
| AAEL010993 | | -0.0899 | 2.8030 | 0.8652 | 0.9996 | queuine tRNA-ribosyltransferase [Source:VB Community Annotation] |
| AAEL018194 | | 0.0550 | 3.6169 | 0.8652 | 0.9996 | |

| | | | | | | |
|---|---|---|---|---|---|---|
| AAEL013731 | | -0.0752 | 4.9930 | 0.8653 | 0.9996 | |
| AAEL013488 | | -0.2540 | -1.1265 | 0.8653 | 0.9996 | glucose inhibited division protein a [Source:VB Community Annotation] |
| AAEL019478 | NA | -0.1253 | 3.5274 | 0.8655 | 0.9996 | NA |
| AAEL002244 | | -0.0767 | 2.3566 | 0.8656 | 0.9996 | |
| AAEL003444 | CASPS19 | 0.1867 | 0.2334 | 0.8657 | 0.9996 | caspase (short) [Source:VB Community Annotation] |
| AAEL025759 | NA | -0.0963 | 2.1294 | 0.8657 | 0.9996 | NA |
| AAEL019827 | NA | -0.0936 | 4.8903 | 0.8657 | 0.9996 | NA |
| AAEL014713 | | -0.0683 | 4.4516 | 0.8658 | 0.9996 | |
| AAEL005626 | | 0.0928 | 6.7418 | 0.8658 | 0.9996 | exostosin-2 [Source:VB Community Annotation] |
| AAEL011907 | | 0.0562 | 3.9714 | 0.8659 | 0.9996 | |
| AAEL007698 | PIWI4 | -0.0824 | 3.5799 | 0.8659 | 0.9996 | PIWI [Source:VB Community Annotation] |
| AAEL021479 | NA | -0.2356 | -1.3254 | 0.8662 | 0.9996 | NA |
| AAEL007521 | | -0.2058 | 1.6928 | 0.8663 | 0.9996 | importin beta-2 [Source:VB Community Annotation] |
| AAEL007343 | | -0.1545 | 5.1803 | 0.8664 | 0.9996 | |
| AAEL002850 | | -0.0744 | 2.7827 | 0.8665 | 0.9996 | patched 1, [Source:VB Community Annotation] |
| AAEL011433 | | -0.0815 | 6.4567 | 0.8669 | 0.9996 | small nuclear ribonucleoprotein sm d3 [Source:VB Community Annotation] |
| AAEL002690 | | 0.2178 | 0.1992 | 0.8669 | 0.9996 | beat protein [Source:VB Community Annotation] |
| AAEL019707 | NA | 0.0969 | 3.8405 | 0.8669 | 0.9996 | NA |
| AAEL006795 | CYP9J15 | 0.2232 | 0.1181 | 0.8670 | 0.9996 | cytochrome P450 [Source:VB Community Annotation] |
| AAEL019693 | NA | -0.1226 | 0.2386 | 0.8670 | 0.9996 | NA |
| AAEL007789 | | -0.0959 | 3.6994 | 0.8671 | 0.9996 | alkyldihydroxyacetonephosphate synthase [Source:VB Community Annotation] |
| AAEL027907 | NA | -0.2534 | -1.8182 | 0.8672 | 0.9996 | NA |
| AAEL008839 | | -0.0818 | 0.5722 | 0.8673 | 0.9996 | |
| AAEL021431 | NA | -0.1467 | -0.1231 | 0.8676 | 0.9996 | NA |
| AAEL020259 | NA | -0.0741 | 4.3593 | 0.8676 | 0.9996 | NA |
| AAEL026976 | NA | -0.0861 | 3.0469 | 0.8677 | 0.9996 | NA |
| AAEL025228 | NA | -0.0814 | 6.1211 | 0.8678 | 0.9996 | NA |
| AAEL004332 | | -0.0661 | 2.7481 | 0.8679 | 0.9996 | |
| AAEL006627 | | -0.2251 | -0.5356 | 0.8682 | 0.9996 | serine-type enodpeptidase, [Source:VB Community Annotation] |
| AAEL002377 | | 0.0635 | 6.3270 | 0.8682 | 0.9996 | Small nuclear ribonucleoprotein-associated protein [Source:UniProtKB/TrEMBL;Acc:Q1HQD9] |
| AAEL013991 | | -0.4563 | 1.1043 | 0.8684 | 0.9996 | guanine nucleotide-binding protein beta 3 (g protein beta3) [Source:VB Community Annotation] |
| AAEL004521 | | 0.0514 | 6.2874 | 0.8685 | 0.9996 | |
| AAEL022214 | NA | 0.0654 | 3.8447 | 0.8685 | 0.9996 | NA |
| AAEL011636 | | -0.0681 | 2.6214 | 0.8685 | 0.9996 | |
| AAEL003769 | | 0.0505 | 4.9526 | 0.8688 | 0.9996 | Methionine aminopeptidase [Source:UniProtKB/TrEMBL;Acc:Q0IFY6] |
| AAEL019751 | NA | -0.1910 | 3.7395 | 0.8688 | 0.9996 | NA |
| AAEL011740 | | -0.0571 | 6.7622 | 0.8691 | 0.9996 | |
| AAEL023601 | NA | -0.0679 | 4.7040 | 0.8692 | 0.9996 | NA |
| AAEL009275 | | -0.0512 | 7.6174 | 0.8693 | 0.9996 | protein phosphatase-1 [Source:VB Community Annotation] |
| AAEL017280 | | -0.1316 | 6.1525 | 0.8693 | 0.9996 | |

| | | | | | | |
|---|---|---|---|---|---|---|
| AAEL008766 | | -0.2456 | 5.0991 | 0.8694 | 0.9996 | |
| AAEL014347 | | 0.1831 | -0.2960 | 0.8694 | 0.9996 | |
| AAEL019566 | NA | -0.2330 | 3.7875 | 0.8694 | 0.9996 | NA |
| AAEL002565 | | -0.1257 | 6.9243 | 0.8694 | 0.9996 | titin [Source:VB Community Annotation] |
| AAEL027331 | NA | -0.0938 | 4.2057 | 0.8694 | 0.9996 | NA |
| AAEL004486 | | -0.0692 | 4.0771 | 0.8695 | 0.9996 | valacyclovir hydrolase [Source:VB Community Annotation] |
| AAEL001988 | | -0.1099 | 2.9711 | 0.8696 | 0.9996 | protein serine/threonine kinase, putative [Source:VB Community Annotation] |
| AAEL008473 | | 0.1571 | 4.8164 | 0.8697 | 0.9996 | cysteine-rich venom protein, putative [Source:VB Community Annotation] |
| AAEL014830 | | -0.1395 | 5.0686 | 0.8697 | 0.9996 | cytochrome P450 [Source:VB Community Annotation] |
| AAEL009264 | | -0.0757 | 2.3874 | 0.8700 | 0.9996 | |
| AAEL004433 | mRpL14 | -0.0610 | 6.3176 | 0.8701 | 0.9996 | mitochondrial ribosomal protein, L14, putative [Source:VB Community Annotation] |
| AAEL023962 | NA | -0.2099 | -2.7906 | 0.8701 | 0.9996 | NA |
| AAEL013829 | | -0.3391 | -0.9904 | 0.8702 | 0.9996 | cuticle protein, putative [Source:VB Community Annotation] |
| AAEL001740 | | -0.0494 | 3.8383 | 0.8702 | 0.9996 | candidate tumor suppressor protein [Source:VB Community Annotation] |
| AAEL019502 | NA | 0.0558 | 3.2387 | 0.8703 | 0.9996 | NA |
| AAEL007282 | | -0.1185 | 5.8272 | 0.8704 | 0.9996 | syntaxin binding protein-1,2,3 [Source:VB Community Annotation] |
| AAEL019646 | NA | -0.1974 | 3.5797 | 0.8704 | 0.9996 | NA |
| AAEL006028 | | 0.0637 | 5.4802 | 0.8704 | 0.9996 | |
| AAEL009585 | | -0.0992 | 4.7010 | 0.8704 | 0.9996 | |
| AAEL008684 | | -0.0692 | 3.0961 | 0.8706 | 0.9996 | serrano protein [Source:VB Community Annotation] |
| AAEL028161 | NA | -0.0579 | 5.0260 | 0.8708 | 0.9996 | NA |
| AAEL000530 | | 0.0925 | 3.5312 | 0.8709 | 0.9996 | Carboxylic ester hydrolase [Source:UniProtKB/TrEMBL;Acc:A0A1S4EW81] |
| AAEL010011 | | -0.0745 | 2.1137 | 0.8711 | 0.9996 | |
| AAEL011730 | | 0.1264 | 3.9428 | 0.8711 | 0.9996 | odorant-binding protein 99c, putative [Source:VB Community Annotation] |
| AAEL025416 | NA | -0.0441 | 5.1070 | 0.8713 | 0.9996 | NA |
| AAEL000950 | | 0.0656 | 6.8856 | 0.8714 | 0.9996 | |
| AAEL003868 | | -0.0519 | 4.3872 | 0.8714 | 0.9996 | DNA repair protein xp-c / rad4 [Source:VB Community Annotation] |
| AAEL019806 | NA | 0.0535 | 5.2687 | 0.8716 | 0.9996 | NA |
| AAEL005277 | | 0.0991 | 4.9111 | 0.8717 | 0.9996 | |
| AAEL009068 | | 0.0408 | 4.3119 | 0.8718 | 0.9996 | dynamin [Source:VB Community Annotation] |
| AAEL002105 | | 0.0815 | 1.3828 | 0.8720 | 0.9996 | |
| AAEL022774 | NA | 0.0865 | 5.9031 | 0.8720 | 0.9996 | NA |
| AAEL010364 | | 0.1677 | -0.2776 | 0.8720 | 0.9996 | |
| AAEL014369 | | 0.3453 | 1.4547 | 0.8721 | 0.9996 | |
| AAEL008801 | | -0.1676 | 1.5149 | 0.8721 | 0.9996 | |
| AAEL000411 | | 0.1386 | 0.7971 | 0.8722 | 0.9996 | |
| AAEL001483 | | -0.0720 | 3.8055 | 0.8723 | 0.9996 | |
| AAEL006659 | | 0.0456 | 4.3429 | 0.8725 | 0.9996 | apyrase [Source:VB Community Annotation] |
| AAEL018273 | | 0.0911 | 3.0160 | 0.8725 | 0.9996 | |
| AAEL019601 | NA | 0.2546 | 0.8552 | 0.8725 | 0.9996 | NA |

| | | | | | | |
|---|---|---|---|---|---|---|
| AAEL010611 | | -0.0921 | 10.7386 | 0.8728 | 0.9996 | Acyl carrier protein [Source:UniProtKB/TrEMBL;Acc:Q16PC9] |
| AAEL008010 | | 0.3245 | 3.8169 | 0.8729 | 0.9996 | sidestep protein [Source:VB Community Annotation] |
| AAEL023113 | NA | -0.3382 | -0.9043 | 0.8729 | 0.9996 | NA |
| AAEL026720 | NA | 0.1996 | 1.5113 | 0.8729 | 0.9996 | NA |
| AAEL022980 | NA | -0.1954 | 1.6623 | 0.8729 | 0.9996 | NA |
| AAEL025818 | NA | -0.0593 | 4.9395 | 0.8731 | 0.9996 | NA |
| AAEL006115 | | 0.0516 | 8.3940 | 0.8731 | 0.9996 | Eukaryotic translation initiation factor 3 subunit K (eIF3k)(eIF-3 p25) [Source:VB Community Annotation] |
| AAEL003703 | | 0.1763 | -0.4227 | 0.8731 | 0.9996 | scarlet protein [Source:VB Community Annotation] |
| AAEL022082 | NA | -0.1955 | 4.1145 | 0.8733 | 0.9996 | NA |
| AAEL026835 | NA | -0.0666 | 1.8820 | 0.8733 | 0.9996 | NA |
| AAEL014262 | | -0.0449 | 5.0423 | 0.8734 | 0.9996 | translation initiation factor eif-2b alpha subunit [Source:VB Community Annotation] |
| AAEL028675 | NA | -0.1481 | -0.3054 | 0.8736 | 0.9996 | NA |
| AAEL017315 | | -0.0465 | 6.1604 | 0.8737 | 0.9996 | |
| AAEL015335 | | 0.1585 | -0.1972 | 0.8737 | 0.9996 | |
| AAEL000500 | | 0.2152 | 0.7785 | 0.8738 | 0.9996 | |
| AAEL014645 | | -0.0568 | 3.7517 | 0.8738 | 0.9996 | |
| AAEL004669 | | -0.2560 | 2.3562 | 0.8739 | 0.9996 | |
| AAEL022564 | NA | 0.1584 | 2.8486 | 0.8739 | 0.9996 | NA |
| AAEL001588 | | -0.0600 | 5.8141 | 0.8741 | 0.9996 | glutamate carboxypeptidase [Source:VB Community Annotation] |
| AAEL008697 | | -0.0511 | 10.5335 | 0.8741 | 0.9996 | cytochrome c oxidase, subunit VB, putative [Source:VB Community Annotation] |
| AAEL013603 | | 0.1022 | 0.2843 | 0.8742 | 0.9996 | short-chain dehydrogenase [Source:VB Community Annotation] |
| AAEL006815 | CYP9J16 | -0.0614 | 4.2713 | 0.8743 | 0.9996 | cytochrome P450 [Source:VB Community Annotation] |
| AAEL024747 | NA | -0.1440 | -0.5747 | 0.8743 | 0.9996 | NA |
| AAEL007372 | GPRGBB1 | -0.2501 | 2.3474 | 0.8745 | 0.9996 | GPCR GABA B Family [Source:VB Community Annotation] |
| AAEL027956 | NA | -0.1475 | -2.1474 | 0.8746 | 0.9996 | NA |
| AAEL003824 | | -0.1430 | 5.2677 | 0.8746 | 0.9996 | |
| AAEL022225 | NA | 0.0802 | 1.6527 | 0.8746 | 0.9996 | NA |
| AAEL025373 | NA | -0.0697 | 3.5468 | 0.8748 | 0.9996 | NA |
| AAEL008403 | | 0.0722 | 2.8372 | 0.8749 | 0.9996 | |
| AAEL000241 | | 0.0373 | 3.6288 | 0.8750 | 0.9996 | |
| AAEL010688 | | -0.1887 | 3.7957 | 0.8752 | 0.9996 | MRAS2, putative [Source:VB Community Annotation] |
| AAEL024630 | NA | -0.3693 | -2.0381 | 0.8753 | 0.9996 | NA |
| AAEL015314 | | -0.1627 | 6.6142 | 0.8754 | 0.9996 | cAMP-dependent protein kinase type ii regulatory subunit [Source:VB Community Annotation] |
| AAEL017025 | | 0.1651 | -2.3713 | 0.8754 | 0.9996 | |
| AAEL024852 | NA | -0.0611 | 4.2327 | 0.8755 | 0.9996 | NA |
| AAEL006333 | | -0.2681 | 6.8811 | 0.8755 | 0.9996 | salivary apyrase, putative [Source:VB Community Annotation] |
| AAEL019814 | NA | 0.1048 | 4.4837 | 0.8757 | 0.9996 | NA |
| AAEL009325 | | -0.0835 | 1.8853 | 0.8757 | 0.9996 | |
| AAEL014999 | | 0.2975 | 1.6450 | 0.8759 | 0.9996 | |
| AAEL019648 | NA | -0.0915 | 3.5929 | 0.8759 | 0.9996 | NA |

| | | | | | | |
|---|---|---|---|---|---|---|
| AAEL021433 | NA | -0.0536 | 4.1491 | 0.8759 | 0.9996 | NA |
| AAEL017244 | | -0.0730 | 3.6735 | 0.8759 | 0.9996 | |
| AAEL004784 | | 0.0644 | 2.9598 | 0.8759 | 0.9996 | 3-2trans-enoyl-CoA isomerase, putative [Source:VB Community Annotation] |
| AAEL014630 | | 0.0521 | 6.5485 | 0.8761 | 0.9996 | cytochrome C1 heme lyase [Source:VB Community Annotation] |
| AAEL002049 | CYC | 0.1560 | 6.7807 | 0.8762 | 0.9996 | circadian protein clock/arnt/bmal/pas [Source:VB Community Annotation] |
| AAEL012687 | | 0.1453 | 3.7368 | 0.8762 | 0.9996 | juvenile hormone-inducible protein, putative [Source:VB Community Annotation] |
| AAEL018200 | | 0.0452 | 4.3045 | 0.8763 | 0.9996 | |
| AAEL005851 | | 0.0730 | 3.3179 | 0.8764 | 0.9996 | exosome complex exonuclease rrp42 (ribosomal RNA processing protein 42) [Source:VB Community Annotation] |
| AAEL013014 | | 0.0532 | 3.5429 | 0.8764 | 0.9996 | zinc finger protein [Source:VB Community Annotation] |
| AAEL001118 | | 0.1137 | 1.0427 | 0.8764 | 0.9996 | |
| AAEL014142 | | -0.2215 | 2.0920 | 0.8765 | 0.9996 | phosphatidylinositol synthase [Source:VB Community Annotation] |
| AAEL018350 | | 0.0832 | 4.9401 | 0.8768 | 0.9996 | |
| AAEL007092 | | 0.0702 | 5.2703 | 0.8768 | 0.9996 | |
| AAEL001240 | GPRNNB1 | 0.2358 | 1.4769 | 0.8768 | 0.9996 | GPCR Orphan/Putative Class B Family [Source:VB Community Annotation] |
| AAEL021789 | NA | -0.2490 | 3.4494 | 0.8770 | 0.9996 | NA |
| AAEL024891 | NA | -0.1521 | -2.9834 | 0.8770 | 0.9996 | NA |
| AAEL008001 | | -0.0453 | 4.4213 | 0.8771 | 0.9996 | |
| AAEL010470 | | 0.0364 | 7.4111 | 0.8772 | 0.9996 | calcineurin b subunit [Source:VB Community Annotation] |
| AAEL022794 | NA | 0.1103 | 1.1594 | 0.8772 | 0.9996 | NA |
| AAEL009524 | | 0.2575 | 11.9842 | 0.8772 | 0.9996 | alpha-amylase [Source:VB Community Annotation] |
| AAEL019732 | NA | -0.2956 | 1.2425 | 0.8773 | 0.9996 | NA |
| AAEL005455 | CTPsyn | -0.0511 | 5.2799 | 0.8773 | 0.9996 | CTP synthase [Source:UniProtKB/TrEMBL;Acc:Q17A05] |
| AAEL029006 | NA | 0.1122 | 4.5144 | 0.8773 | 0.9996 | NA |
| AAEL006961 | | -0.1637 | 0.0293 | 0.8774 | 0.9996 | lipase [Source:VB Community Annotation] |
| AAEL010775 | | -0.0909 | 1.1803 | 0.8775 | 0.9996 | |
| AAEL011872 | | 0.2778 | 0.2927 | 0.8775 | 0.9996 | |
| AAEL019508 | NA | -0.1710 | 1.0144 | 0.8776 | 0.9996 | NA |
| AAEL005742 | | 0.0612 | 7.5424 | 0.8776 | 0.9996 | transcription initiation factor TFIID subunit 10, putative [Source:VB Community Annotation] |
| AAEL022107 | NA | -0.1520 | 6.9518 | 0.8777 | 0.9996 | NA |
| AAEL022979 | NA | -0.0619 | 5.1486 | 0.8778 | 0.9996 | NA |
| AAEL000440 | | 0.0623 | 3.6651 | 0.8778 | 0.9996 | DNA repair/transcription protein met18/mms19 [Source:VB Community Annotation] |
| AAEL013367 | | -0.1936 | 2.5766 | 0.8780 | 0.9996 | |
| AAEL002276 | | 0.1128 | 2.4192 | 0.8781 | 0.9996 | serine protease, putative [Source:VB Community Annotation] |
| AAEL010417 | | -0.0493 | 3.8393 | 0.8783 | 0.9996 | |
| AAEL005000 | | -0.0861 | 1.7657 | 0.8783 | 0.9996 | |
| AAEL002827 | | -0.0768 | 10.7220 | 0.8784 | 0.9996 | ATP synthase beta subunit [Source:VB Community Annotation] |
| AAEL001875 | | 0.0708 | 5.4948 | 0.8786 | 0.9996 | |
| AAEL019605 | NA | -0.2843 | 0.9257 | 0.8786 | 0.9996 | NA |
| AAEL019606 | NA | 0.1323 | 4.7099 | 0.8788 | 0.9996 | NA |
| AAEL028064 | NA | 0.2967 | 3.8606 | 0.8789 | 0.9996 | NA |

| | | | | | | |
|---|---|---|---|---|---|---|
| AAEL014343 | | -0.2061 | 3.5776 | 0.8790 | 0.9996 | |
| AAEL021943 | NA | 0.1407 | -0.7681 | 0.8790 | 0.9996 | NA |
| AAEL024394 | NA | 0.2933 | 1.8841 | 0.8790 | 0.9996 | NA |
| AAEL014596 | | -0.0935 | 1.5024 | 0.8791 | 0.9996 | |
| AAEL003269 | | -0.1646 | 6.1513 | 0.8792 | 0.9996 | |
| AAEL000360 | | 0.1951 | -0.5704 | 0.8793 | 0.9996 | DNAJ homolog subfamily B member, putative [Source:VB Community Annotation] |
| AAEL010201 | | -0.1352 | 1.6085 | 0.8793 | 0.9996 | axonemal dynein intermediate chain [Source:VB Community Annotation] |
| AAEL026781 | NA | -0.1521 | -3.0310 | 0.8793 | 0.9996 | NA |
| AAEL012250 | | -0.1586 | -1.3408 | 0.8793 | 0.9996 | developmentally regulated GTP-binding protein 2 (drg 2) [Source:VB Community Annotation] |
| AAEL019928 | NA | 0.2782 | 2.2101 | 0.8794 | 0.9996 | NA |
| AAEL026215 | NA | 0.1038 | 2.5500 | 0.8794 | 0.9996 | NA |
| AAEL025531 | NA | 0.3153 | 1.6283 | 0.8794 | 0.9996 | NA |
| AAEL012539 | | -0.0659 | 4.5893 | 0.8795 | 0.9996 | heparan sulphate n-deacetylase/n-sulfotransferase [Source:VB Community Annotation] |
| AAEL009783 | | -0.2190 | -1.8642 | 0.8795 | 0.9996 | cuticle protein, putative [Source:VB Community Annotation] |
| AAEL000604 | | -0.2098 | -0.1236 | 0.8795 | 0.9996 | |
| AAEL000674 | | 0.0642 | 3.0201 | 0.8795 | 0.9996 | RNA m5u methyltransferase [Source:VB Community Annotation] |
| AAEL011466 | | 0.0856 | 2.1001 | 0.8797 | 0.9996 | Biogenesis of lysosome-related organelles complex 1 subunit 6 [Source:UniProtKB/TrEMBL;Acc:Q16Q01] |
| AAEL000844 | | -0.0559 | 4.4103 | 0.8797 | 0.9996 | ad-003 [Source:VB Community Annotation] |
| AAEL016123 | tRNA-Lys | 0.1166 | 1.3374 | 0.8797 | 0.9996 | |
| AAEL010588 | | 0.0562 | 4.0460 | 0.8797 | 0.9996 | striatin, putative [Source:VB Community Annotation] |
| AAEL026074 | NA | -0.2598 | -0.1801 | 0.8797 | 0.9996 | NA |
| AAEL006514 | | -0.0832 | 6.4335 | 0.8797 | 0.9996 | sodium-dependent phosphate transporter [Source:VB Community Annotation] |
| AAEL006498 | GPROP1 | -0.1014 | 4.8058 | 0.8800 | 0.9996 | long wavelength sensitive opsin [Source:VB Community Annotation] |
| AAEL007719 | | -0.2124 | 1.4907 | 0.8800 | 0.9996 | rhombotin [Source:VB Community Annotation] |
| AAEL024429 | NA | 0.3882 | 2.6533 | 0.8800 | 0.9996 | NA |
| AAEL003237 | | 0.0447 | 4.6217 | 0.8801 | 0.9996 | low molecular weight protein-tyrosine-phosphatase [Source:VB Community Annotation] |
| AAEL020992 | NA | 0.0650 | 6.0322 | 0.8801 | 0.9996 | NA |
| AAEL004802 | | 0.0519 | 5.2055 | 0.8802 | 0.9996 | |
| AAEL004284 | | 0.0596 | 7.0052 | 0.8802 | 0.9996 | mitochondrial ATPase inhibitor, putative [Source:VB Community Annotation] |
| AAEL019453 | NA | 0.2611 | 0.8106 | 0.8803 | 0.9996 | NA |
| AAEL021605 | NA | 0.0690 | 3.8341 | 0.8805 | 0.9996 | NA |
| AAEL007126 | | 0.2665 | 0.5578 | 0.8806 | 0.9996 | sugar transporter [Source:VB Community Annotation] |
| AAEL010239 | | -0.0929 | 1.4676 | 0.8806 | 0.9996 | |
| AAEL000316 | | 0.1500 | 4.9236 | 0.8806 | 0.9996 | |
| AAEL020156 | NA | -0.1380 | 4.7734 | 0.8807 | 0.9996 | NA |
| AAEL005485 | | 0.0589 | 3.7087 | 0.8807 | 0.9996 | adamts-7 [Source:VB Community Annotation] |
| AAEL000279 | | 0.0718 | 3.7775 | 0.8808 | 0.9996 | N-formylmethionylaminoacyl-tRNA deformylase, putative [Source:VB Community Annotation] |
| AAEL006923 | | -0.0548 | 4.9697 | 0.8808 | 0.9996 | |
| AAEL021664 | NA | -0.2001 | -1.6359 | 0.8809 | 0.9996 | NA |
| AAEL010232 | | -0.1242 | 0.0889 | 0.8810 | 0.9996 | |

| | | | | | | |
|---|---|---|---|---|---|---|
| AAEL013640 | | 0.0923 | 5.4412 | 0.8811 | 0.9996 | lung carbonyl reductase [Source:VB Community Annotation] |
| AAEL022087 | NA | -0.1394 | 2.9725 | 0.8811 | 0.9996 | NA |
| AAEL007200 | | 0.1898 | -0.9275 | 0.8811 | 0.9996 | amino acid transporter [Source:VB Community Annotation] |
| AAEL006298 | | -0.0789 | 1.2531 | 0.8813 | 0.9996 | |
| AAEL001095 | | 0.1695 | 1.6964 | 0.8813 | 0.9996 | |
| AAEL009368 | | -0.0951 | 4.3543 | 0.8814 | 0.9996 | lipoic acid synthetase [Source:VB Community Annotation] |
| AAEL003229 | | -0.0509 | 4.8495 | 0.8814 | 0.9996 | |
| AAEL013661 | | 0.0409 | 6.6570 | 0.8815 | 0.9996 | actin binding protein, putative [Source:VB Community Annotation] |
| AAEL005753 | | 0.0558 | 5.0953 | 0.8816 | 0.9996 | serine protease [Source:VB Community Annotation] |
| AAEL025041 | NA | 0.1742 | 2.8636 | 0.8818 | 0.9996 | NA |
| AAEL010451 | | 0.2395 | -0.2138 | 0.8821 | 0.9996 | rabaptin-5, putative [Source:VB Community Annotation] |
| AAEL025618 | NA | -0.0859 | 7.2838 | 0.8822 | 0.9996 | NA |
| AAEL006557 | | -0.0541 | 7.2098 | 0.8822 | 0.9996 | |
| AAEL010557 | | 0.0751 | 0.8991 | 0.8823 | 0.9996 | |
| AAEL019799 | NA | -0.0728 | 9.3583 | 0.8823 | 0.9996 | NA |
| AAEL004987 | | -0.0669 | 9.8127 | 0.8823 | 0.9996 | |
| AAEL017184 | | 0.1584 | -0.5144 | 0.8824 | 0.9996 | |
| AAEL026334 | NA | -0.1194 | 1.4691 | 0.8824 | 0.9996 | NA |
| AAEL019531 | NA | -0.3563 | 2.0487 | 0.8825 | 0.9996 | NA |
| AAEL014215 | | -0.0433 | 6.7588 | 0.8825 | 0.9996 | nicotinate phosphoribosyltransferase [Source:VB Community Annotation] |
| AAEL004554 | | 0.0639 | 4.7845 | 0.8826 | 0.9996 | |
| AAEL028145 | NA | 0.2547 | 0.8541 | 0.8826 | 0.9996 | NA |
| AAEL025187 | NA | -0.3091 | -0.5891 | 0.8828 | 0.9996 | NA |
| AAEL013863 | | 0.2912 | 0.5204 | 0.8828 | 0.9996 | phosphatidylinositol-binding clathrin assembly protein [Source:VB Community Annotation] |
| AAEL023508 | NA | 0.0642 | 4.0540 | 0.8828 | 0.9996 | NA |
| AAEL012545 | | -0.0712 | 3.0848 | 0.8829 | 0.9996 | Proliferating cell nuclear antigen [Source:UniProtKB/TrEMBL;Acc:Q4PKD7] |
| AAEL007465 | | -0.2876 | 0.6842 | 0.8829 | 0.9996 | |
| AAEL014497 | | 0.0617 | 2.7552 | 0.8830 | 0.9996 | |
| AAEL022930 | NA | -0.0647 | 5.3639 | 0.8831 | 0.9996 | NA |
| AAEL019464 | NA | -0.2039 | -0.0957 | 0.8831 | 0.9996 | NA |
| AAEL019550 | NA | 0.0940 | 7.1480 | 0.8831 | 0.9996 | NA |
| AAEL000511 | | 0.2121 | 5.2007 | 0.8831 | 0.9996 | Carboxylic ester hydrolase [Source:UniProtKB/TrEMBL;Acc:A0A1S4EW26] |
| AAEL010848 | | -0.1521 | -2.8793 | 0.8832 | 0.9996 | |
| AAEL026141 | NA | 0.0574 | 4.3847 | 0.8833 | 0.9996 | NA |
| AAEL017085 | GSTO1 | -0.0366 | 4.7843 | 0.8833 | 0.9996 | glutathione transferase [Source:VB Community Annotation] |
| AAEL021682 | NA | -0.1521 | -3.0234 | 0.8834 | 0.9996 | NA |
| AAEL015247 | | 0.2245 | -0.4543 | 0.8835 | 0.9996 | |
| AAEL005118 | | -0.1256 | 2.4116 | 0.8837 | 0.9996 | |
| AAEL020194 | NA | 0.1126 | 1.6011 | 0.8839 | 0.9996 | NA |
| AAEL021255 | NA | 0.0543 | 5.7854 | 0.8840 | 0.9996 | NA |

| | | | | | | |
|---|---|---|---|---|---|---|
| AAEL012103 | | -0.0446 | 5.0034 | 0.8840 | 0.9996 | |
| AAEL000454 | | -0.0614 | 8.5381 | 0.8843 | 0.9996 | Isocitrate dehydrogenase [NAD] subunit, mitochondrial [Source:UniProtKB/TrEMBL;Acc:Q17P79] |
| AAEL025886 | NA | -0.1521 | -2.9178 | 0.8844 | 0.9996 | NA |
| AAEL000467 | mRpL49 | -0.0545 | 5.3734 | 0.8844 | 0.9996 | mitochondrial ribosomal protein, L49, putative [Source:VB Community Annotation] |
| AAEL014522 | | 0.0613 | 2.3593 | 0.8846 | 0.9996 | |
| AAEL005276 | | -0.3017 | 0.5214 | 0.8846 | 0.9996 | target of myb1 (tom1) [Source:VB Community Annotation] |
| AAEL022610 | NA | -0.2523 | 1.4726 | 0.8846 | 0.9996 | NA |
| AAEL020062 | NA | -0.1263 | 4.2685 | 0.8847 | 0.9996 | NA |
| AAEL006390 | | -0.2254 | 1.8806 | 0.8849 | 0.9996 | vacuolar proton ATPases [Source:VB Community Annotation] |
| AAEL003430 | | 0.0631 | 6.5353 | 0.8849 | 0.9996 | |
| AAEL011453 | CTL14 | -0.0837 | 3.5830 | 0.8849 | 0.9996 | C-Type Lectin (CTL14) [Source:VB Community Annotation] |
| AAEL006983 | | 0.0620 | 2.2838 | 0.8850 | 0.9996 | |
| AAEL004813 | | 0.0509 | 5.2776 | 0.8850 | 0.9996 | M-phase phosphoprotein, putative [Source:VB Community Annotation] |
| AAEL018972 | tRNA-Pro | 0.1344 | -0.2297 | 0.8851 | 0.9996 | |
| AAEL000235 | | -0.0868 | 3.7443 | 0.8854 | 0.9996 | |
| AAEL007391 | | -0.0676 | 6.8233 | 0.8855 | 0.9996 | |
| AAEL027779 | NA | -0.1349 | -0.0093 | 0.8856 | 0.9996 | NA |
| AAEL014258 | | -0.0499 | 6.6422 | 0.8857 | 0.9996 | monocarboxylate transporter [Source:VB Community Annotation] |
| AAEL004854 | | -0.1119 | 3.5886 | 0.8857 | 0.9996 | |
| AAEL015465 | | 0.0756 | 5.2492 | 0.8858 | 0.9996 | clip-domain serine protease, putative [Source:VB Community Annotation] |
| AAEL000435 | | 0.0558 | 3.3122 | 0.8859 | 0.9996 | THO complex, putative [Source:VB Community Annotation] |
| AAEL006668 | | -0.0705 | 4.0266 | 0.8860 | 0.9996 | |
| AAEL023040 | NA | 0.0875 | 4.1964 | 0.8862 | 0.9996 | NA |
| AAEL004221 | | -0.0679 | 5.2122 | 0.8862 | 0.9996 | glycogen synthase [Source:VB Community Annotation] |
| AAEL017042 | | 0.0806 | 5.0902 | 0.8863 | 0.9996 | |
| AAEL018336 | | 0.0316 | 6.9633 | 0.8863 | 0.9996 | |
| AAEL021235 | NA | -0.1521 | -2.7255 | 0.8864 | 0.9996 | NA |
| AAEL012447 | | -0.1831 | 1.0926 | 0.8867 | 0.9996 | elastase, putative [Source:VB Community Annotation] |
| AAEL025425 | NA | 0.0396 | 3.6100 | 0.8867 | 0.9996 | NA |
| AAEL006409 | | 0.0414 | 4.7358 | 0.8868 | 0.9996 | sialin, sodium/sialic acid cotransporter, putative [Source:VB Community Annotation] |
| AAEL001619 | | -0.0476 | 3.5315 | 0.8872 | 0.9996 | |
| AAEL012519 | | -0.2111 | 0.5174 | 0.8873 | 0.9996 | actin binding protein, putative [Source:VB Community Annotation] |
| AAEL006563 | Vcp | 0.3054 | -1.5801 | 0.8873 | 0.9996 | Vitellogenic carboxypeptidase Precursor (EC 3.4.16.-) [Source:VB Community Annotation] |
| AAEL008760 | | 0.1858 | -0.3491 | 0.8875 | 0.9996 | |
| AAEL004891 | | 0.0566 | 4.2350 | 0.8876 | 0.9996 | |
| AAEL011504 | | -0.1927 | 0.5006 | 0.8876 | 0.9996 | pupal cuticle protein, putative [Source:VB Community Annotation] |
| AAEL022812 | NA | -0.0520 | 3.8180 | 0.8877 | 0.9996 | NA |
| AAEL004526 | | 0.0829 | 1.9906 | 0.8877 | 0.9996 | |
| AAEL026470 | NA | -0.0421 | 3.8761 | 0.8878 | 0.9996 | NA |
| AAEL000073 | OBP4 | -0.0731 | 7.7221 | 0.8879 | 0.9996 | odorant binding protein OBP4 [Source:VB Community Annotation] |

| | | | | | | |
|---|---|---|---|---|---|---|
| AAEL000342 | | -0.2299 | 2.7772 | 0.8879 | 0.9996 | peroxidasin [Source:VB Community Annotation] |
| AAEL001423 | | 0.0839 | 5.4878 | 0.8880 | 0.9996 | acid phosphatase-1 [Source:VB Community Annotation] |
| AAEL019703 | NA | -0.2672 | 1.2794 | 0.8880 | 0.9996 | NA |
| AAEL001682 | | 0.1312 | 2.1883 | 0.8880 | 0.9996 | nuclear movement protein nudc [Source:VB Community Annotation] |
| AAEL013281 | | 0.1756 | -1.5338 | 0.8881 | 0.9996 | serine-type enodpeptidase [Source:VB Community Annotation] |
| AAEL002102 | | -0.0758 | 3.5547 | 0.8882 | 0.9996 | |
| AAEL001726 | | -0.2066 | -1.1697 | 0.8883 | 0.9996 | |
| AAEL009712 | | 0.2191 | 2.8831 | 0.8885 | 0.9996 | |
| AAEL020555 | NA | 0.1474 | -2.1162 | 0.8885 | 0.9996 | NA |
| AAEL018333 | | 0.0414 | 4.9929 | 0.8886 | 0.9996 | |
| AAEL006050 | | -0.1236 | 2.5525 | 0.8888 | 0.9996 | nuclear RNA export factor 2 (NXF2), putative [Source:VB Community Annotation] |
| AAEL000630 | | -0.0915 | 3.7731 | 0.8888 | 0.9996 | cardioacceleratory peptide 2a, putative [Source:VB Community Annotation] |
| AAEL006825 | | -0.0922 | 4.4550 | 0.8889 | 0.9996 | |
| AAEL027166 | NA | -0.0720 | 3.8180 | 0.8890 | 0.9996 | NA |
| AAEL011842 | | 0.0502 | 3.4537 | 0.8890 | 0.9996 | mitochondrial carrier protein [Source:VB Community Annotation] |
| AAEL007203 | | -0.0457 | 3.9424 | 0.8892 | 0.9996 | |
| AAEL003925 | | -0.0767 | 7.6310 | 0.8892 | 0.9996 | |
| AAEL016972 | | -0.0457 | 10.5185 | 0.8892 | 0.9996 | |
| AAEL011248 | inx3 | -0.2156 | 1.4848 | 0.8893 | 0.9996 | Innexin [Source:UniProtKB/TrEMBL;Acc:Q16QK9] |
| AAEL001867 | | -0.2793 | 1.4777 | 0.8893 | 0.9996 | sodium-dependent phosphate transporter [Source:VB Community Annotation] |
| AAEL002125 | | 0.0898 | 5.3849 | 0.8894 | 0.9996 | |
| AAEL014297 | | 0.1225 | 1.1992 | 0.8895 | 0.9996 | xenotropic and polytropic murine leukemia virus receptor xpr1 [Source:VB Community Annotation] |
| AAEL026479 | NA | -0.1940 | -0.6781 | 0.8895 | 0.9996 | NA |
| AAEL000174 | | 0.2322 | 0.9419 | 0.8896 | 0.9996 | retinal protein [Source:VB Community Annotation] |
| AAEL002258 | | 0.0463 | 3.6332 | 0.8896 | 0.9996 | |
| AAEL013709 | | -0.0405 | 8.4175 | 0.8898 | 0.9996 | Histone H4 [Source:UniProtKB/TrEMBL;Acc:Q16IE4] |
| AAEL021555 | NA | 0.1576 | 0.5681 | 0.8898 | 0.9996 | NA |
| AAEL016608 | tRNA-Pro | -0.0872 | 1.9210 | 0.8899 | 0.9996 | |
| AAEL013506 | | 0.1582 | -1.1885 | 0.8900 | 0.9996 | fibrinogen and fibronectin [Source:VB Community Annotation] |
| AAEL005838 | | 0.0736 | 2.2028 | 0.8901 | 0.9996 | |
| AAEL019525 | NA | -0.1064 | 5.4274 | 0.8902 | 0.9996 | NA |
| AAEL005312 | | 0.0689 | 6.4533 | 0.8902 | 0.9996 | |
| AAEL022750 | NA | 0.0909 | 4.5354 | 0.8903 | 0.9996 | NA |
| AAEL020180 | NA | 0.0373 | 5.0262 | 0.8904 | 0.9996 | NA |
| AAEL000006 | | 0.1276 | 5.2659 | 0.8905 | 0.9996 | phosphoenolpyruvate carboxykinase [Source:VB Community Annotation] |
| AAEL018699 | | 0.0538 | 5.5218 | 0.8907 | 0.9996 | |
| AAEL021838 | NA | -0.1475 | -2.6957 | 0.8909 | 0.9996 | NA |
| AAEL008156 | | -0.0526 | 3.4136 | 0.8909 | 0.9996 | |
| AAEL027881 | NA | 0.1818 | -1.3731 | 0.8909 | 0.9996 | NA |
| AAEL017096 | | 0.0628 | 9.7872 | 0.8909 | 0.9996 | Elongation factor 1-alpha [Source:UniProtKB/TrEMBL;Acc:Q1HR88] |

| | | | | | | |
|---|---|---|---|---|---|---|
| AAEL003871 | Prosalpha7 | 0.0523 | 6.9560 | 0.8910 | 0.9996 | 26S proteasome alpha 7 subunit [Source:VB Community Annotation] |
| AAEL009630 | | -0.1635 | 5.5545 | 0.8910 | 0.9996 | high-affinity cgmp-specific 3,5-cyclic phosphodiesterase [Source:VB Community Annotatio |
| AAEL009554 | | -0.1380 | 2.6800 | 0.8910 | 0.9996 | |
| AAEL002943 | | -0.0684 | 2.2871 | 0.8914 | 0.9996 | che-11 [Source:VB Community Annotation] |
| AAEL012859 | | -0.1089 | 2.0710 | 0.8915 | 0.9996 | |
| AAEL010245 | | 0.0519 | 5.5844 | 0.8916 | 0.9996 | chloride channel, putative [Source:VB Community Annotation] |
| AAEL017116 | | 0.0706 | 6.7678 | 0.8921 | 0.9996 | |
| AAEL010711 | | 0.1852 | 4.1118 | 0.8922 | 0.9996 | eph receptor tyrosine kinase [Source:VB Community Annotation] |
| AAEL006433 | | 0.0346 | 5.7169 | 0.8924 | 0.9996 | Piezo-type mechanosensitive ion channel component (Fragment) [Source:UniProtKB/TrEMBL;Acc:Q176C8] |
| AAEL006277 | | -0.0622 | 3.8357 | 0.8924 | 0.9996 | |
| AAEL026540 | NA | -0.0868 | 6.2369 | 0.8925 | 0.9996 | NA |
| AAEL009948 | ALDH9948 | 0.0858 | 4.9464 | 0.8926 | 0.9996 | aldehyde dehydrogenase [Source:VB Community Annotation] |
| AAEL019810 | NA | -0.2165 | 1.3575 | 0.8926 | 0.9996 | NA |
| AAEL026392 | NA | -0.2087 | 1.8460 | 0.8927 | 0.9996 | NA |
| AAEL020327 | NA | 0.1730 | -0.7014 | 0.8928 | 0.9996 | NA |
| AAEL001442 | | 0.1557 | -0.1306 | 0.8929 | 0.9996 | map-kinase activating death domain protein (madd)/denn/aex-3(c.elegans) [Source:VB Community Annotation] |
| AAEL019654 | NA | 0.0676 | 5.5274 | 0.8929 | 0.9996 | NA |
| AAEL002812 | | 0.0486 | 5.3239 | 0.8930 | 0.9996 | |
| AAEL019529 | NA | 0.0505 | 3.8168 | 0.8931 | 0.9996 | NA |
| AAEL026602 | NA | 0.0757 | 4.4061 | 0.8931 | 0.9996 | NA |
| AAEL018137 | | 0.0539 | 4.5685 | 0.8932 | 0.9996 | |
| AAEL010186 | | -0.2492 | 2.4142 | 0.8936 | 0.9996 | |
| AAEL024141 | NA | 0.0264 | 4.9778 | 0.8936 | 0.9996 | NA |
| AAEL009151 | RpS15a | -0.0352 | 11.4723 | 0.8936 | 0.9996 | 40S ribosomal protein S15a [Source:VB Community Annotation] |
| AAEL000247 | | 0.0743 | 1.5209 | 0.8938 | 0.9996 | |
| AAEL010584 | | -0.0302 | 4.8048 | 0.8939 | 0.9996 | vesicular mannose-binding lectin [Source:VB Community Annotation] |
| AAEL017212 | | 0.0436 | 8.9560 | 0.8940 | 0.9996 | |
| AAEL010342 | | 0.0969 | 0.0123 | 0.8941 | 0.9996 | racGTPase-activating protein [Source:VB Community Annotation] |
| AAEL006462 | | -0.0367 | 4.0091 | 0.8941 | 0.9996 | TFIID subunit TAFII55, putative [Source:VB Community Annotation] |
| AAEL008510 | | -0.0895 | 1.1449 | 0.8942 | 0.9996 | sphingosine kinase a, b [Source:VB Community Annotation] |
| AAEL019625 | NA | -0.1936 | -0.4864 | 0.8942 | 0.9996 | NA |
| AAEL006932 | | -0.1451 | 3.8879 | 0.8944 | 0.9996 | transcription factor ap-2 [Source:VB Community Annotation] |
| AAEL001300 | | -0.0638 | 2.4534 | 0.8944 | 0.9996 | |
| AAEL020570 | NA | 0.2514 | 1.3676 | 0.8944 | 0.9996 | NA |
| AAEL013553 | | -0.0305 | 4.4255 | 0.8945 | 0.9996 | |
| AAEL003402 | | 0.0498 | 5.1284 | 0.8945 | 0.9996 | sphingomyelin phosphodiesterase [Source:VB Community Annotation] |
| AAEL007679 | | 0.0609 | 5.1111 | 0.8946 | 0.9996 | |
| AAEL000633 | TOLL8 | 0.2299 | 0.7811 | 0.8947 | 0.9996 | Toll-like receptor [Source:VB Community Annotation] |
| AAEL022231 | NA | 0.1750 | -0.6790 | 0.8948 | 0.9996 | NA |
| AAEL022280 | NA | 0.1538 | -0.3193 | 0.8949 | 0.9996 | NA |

| | | | | | | |
|---|---|---|---|---|---|---|
| AAEL011488 | | 0.1584 | -1.1730 | 0.8949 | 0.9996 | short-chain dehydrogenase [Source:VB Community Annotation] |
| AAEL019445 | NA | -0.0814 | 3.9039 | 0.8949 | 0.9996 | NA |
| AAEL006962 | | -0.0847 | 6.3397 | 0.8951 | 0.9996 | |
| AAEL002192 | | -0.0484 | 4.5520 | 0.8952 | 0.9996 | M12 mutant protein precursor, putative [Source:VB Community Annotation] |
| AAEL005508 | | 0.0464 | 8.8081 | 0.8953 | 0.9996 | NADH-ubiquinone oxidoreductase 24 kda subunit [Source:VB Community Annotation] |
| AAEL000903 | e(y)2 | -0.0406 | 4.6154 | 0.8954 | 0.9996 | Enhancer of yellow 2 transcription factor [Source:UniProtKB/Swiss-Prot;Acc:Q17MZ8] |
| AAEL012398 | | -0.1224 | 0.3184 | 0.8955 | 0.9996 | |
| AAEL009702 | | -0.0471 | 3.3939 | 0.8956 | 0.9996 | Ubiquitinyl hydrolase 1 [Source:UniProtKB/TrEMBL;Acc:Q16V37] |
| AAEL028983 | NA | 0.1405 | 0.3854 | 0.8957 | 0.9996 | NA |
| AAEL001185 | | -0.0406 | 4.3279 | 0.8957 | 0.9996 | |
| AAEL011057 | | -0.0531 | 5.4648 | 0.8957 | 0.9996 | DNA-repair protein complementing XP-A cells homolog [Source:VB Community Annotation] |
| AAEL025977 | NA | -0.0366 | 5.7341 | 0.8958 | 0.9996 | NA |
| AAEL002148 | | 0.0561 | 5.6294 | 0.8959 | 0.9996 | |
| AAEL007056 | | -0.0432 | 2.4876 | 0.8960 | 0.9996 | btf [Source:VB Community Annotation] |
| AAEL015430 | | -0.1368 | 5.1017 | 0.8960 | 0.9996 | serine protease, putative [Source:VB Community Annotation] |
| AAEL006267 | | 0.0821 | 4.7567 | 0.8962 | 0.9996 | ras-related protein Rab-3C, putative [Source:VB Community Annotation] |
| AAEL012683 | | 0.1576 | 0.0503 | 0.8963 | 0.9996 | |
| AAEL020932 | NA | -0.1521 | -2.6740 | 0.8963 | 0.9996 | NA |
| AAEL009367 | | 0.0473 | 5.1838 | 0.8965 | 0.9996 | myelin proteolipid [Source:VB Community Annotation] |
| AAEL014080 | ALDH1408( | 0.0422 | 3.8792 | 0.8967 | 0.9996 | aldehyde dehydrogenase [Source:VB Community Annotation] |
| AAEL004717 | | -0.1887 | 4.3487 | 0.8967 | 0.9996 | cyclin-dependent kinase 5 activator [Source:VB Community Annotation] |
| AAEL009265 | | 0.2502 | 1.8154 | 0.8968 | 0.9996 | |
| AAEL005653 | | 0.0680 | 5.1211 | 0.8969 | 0.9996 | leukocyte receptor cluster (lrc) member 4 protein [Source:VB Community Annotation] |
| AAEL027311 | NA | -0.4752 | 0.1571 | 0.8970 | 0.9996 | NA |
| AAEL001853 | | 0.0454 | 4.3800 | 0.8971 | 0.9996 | rac-GTP binding protein [Source:VB Community Annotation] |
| AAEL013539 | | 0.2871 | 2.0325 | 0.8973 | 0.9996 | SH2/SH3 adaptor protein [Source:VB Community Annotation] |
| AAEL003913 | | -0.0806 | 1.6796 | 0.8973 | 0.9996 | methionine-tRNA synthetase [Source:VB Community Annotation] |
| AAEL000396 | | 0.0640 | 1.7722 | 0.8974 | 0.9996 | |
| AAEL000123 | | 0.1987 | 1.9967 | 0.8974 | 0.9996 | |
| AAEL001835 | | -0.0578 | 4.3994 | 0.8974 | 0.9996 | sodium/solute symporter [Source:VB Community Annotation] |
| AAEL015180 | | 0.1550 | -1.7033 | 0.8975 | 0.9996 | smooth muscle caldesmon, putative [Source:VB Community Annotation] |
| AAEL025517 | NA | -0.0334 | 4.9024 | 0.8975 | 0.9996 | NA |
| AAEL012102 | | -0.0392 | 4.0348 | 0.8976 | 0.9996 | |
| AAEL009961 | | -0.0547 | 4.8326 | 0.8976 | 0.9996 | |
| AAEL027621 | NA | -0.0676 | 1.3625 | 0.8976 | 0.9996 | NA |
| AAEL025130 | NA | -0.0481 | 4.0473 | 0.8977 | 0.9996 | NA |
| AAEL000685 | | 0.0370 | 3.8970 | 0.8977 | 0.9996 | |
| AAEL004674 | | -0.1377 | 0.8698 | 0.8977 | 0.9996 | pupal cuticle protein 78E, putative [Source:VB Community Annotation] |
| AAEL005183 | | 0.0376 | 5.1010 | 0.8978 | 0.9996 | |
| AAEL002740 | | -0.1887 | 1.0114 | 0.8978 | 0.9996 | homeobox protein otx [Source:VB Community Annotation] |

| | | | | | | |
|---|---|---|---|---|---|---|
| AAEL011965 | | -0.0957 | 0.9527 | 0.8978 | 0.9996 | nuclear lamin L1 alpha, putative [Source:VB Community Annotation] |
| AAEL022352 | NA | 0.0957 | 4.0349 | 0.8980 | 0.9996 | NA |
| AAEL010679 | | -0.0495 | 4.1635 | 0.8980 | 0.9996 | monocyte to macrophage differentiation protein [Source:VB Community Annotation] |
| AAEL007247 | | -0.0468 | 5.7148 | 0.8980 | 0.9996 | |
| AAEL027821 | NA | 0.0377 | 4.8189 | 0.8980 | 0.9996 | NA |
| AAEL022416 | NA | -0.1397 | -2.6110 | 0.8981 | 0.9996 | NA |
| AAEL017883 | U5 | 0.0989 | 2.0306 | 0.8981 | 0.9996 | U5 spliceosomal RNA [Source:RFAM;Acc:RF00020] |
| AAEL007512 | | 0.0358 | 4.0827 | 0.8981 | 0.9996 | ATP-dependent RNA and DNA helicase [Source:VB Community Annotation] |
| AAEL005874 | | -0.0436 | 6.5037 | 0.8982 | 0.9996 | |
| AAEL027802 | NA | -0.2679 | -0.4554 | 0.8982 | 0.9996 | NA |
| AAEL018227 | | -0.2083 | -0.1614 | 0.8983 | 0.9996 | |
| AAEL005944 | mRpS23 | 0.0391 | 4.7771 | 0.8984 | 0.9996 | mitochondrial ribosomal protein, S23, putative [Source:VB Community Annotation] |
| AAEL022035 | NA | -0.1521 | -2.6810 | 0.8984 | 0.9996 | NA |
| AAEL014251 | IAP5 | 0.0704 | 1.7320 | 0.8985 | 0.9996 | Inhibitor of Apoptosis (IAP)  containing Baculoviral IAP Repeat(s) (BIR domains). [Source:VB Community Annotation] |
| AAEL013778 | | 0.1166 | 3.7053 | 0.8986 | 0.9996 | F-actin capping protein alpha [Source:VB Community Annotation] |
| AAEL006697 | | -0.0589 | 3.0435 | 0.8986 | 0.9996 | |
| AAEL008490 | | 0.0414 | 7.9802 | 0.8987 | 0.9996 | NADH dehydrogenase, putative [Source:VB Community Annotation] |
| AAEL010299 | RpS12 | -0.0350 | 10.6155 | 0.8988 | 0.9996 | 40S ribosomal protein S12 [Source:UniProtKB/TrEMBL;Acc:Q1HRM3] |
| AAEL003408 | | 0.0698 | 1.1803 | 0.8989 | 0.9996 | leucine-rich transmembrane protein [Source:VB Community Annotation] |
| AAEL002229 | | -0.1407 | 1.7048 | 0.8989 | 0.9996 | cuticle protein, putative [Source:VB Community Annotation] |
| AAEL001524 | | -0.1521 | -2.1531 | 0.8990 | 0.9996 | rab23 [Source:VB Community Annotation] |
| AAEL013915 | | 0.0468 | 3.8888 | 0.8990 | 0.9996 | monocarboxylate transporter [Source:VB Community Annotation] |
| AAEL009345 | | -0.0323 | 7.2445 | 0.8990 | 0.9996 | prohibitin [Source:VB Community Annotation] |
| AAEL010419 | | 0.0440 | 5.3756 | 0.8991 | 0.9996 | translin associated factor x [Source:VB Community Annotation] |
| AAEL027833 | NA | 0.1492 | -0.9041 | 0.8991 | 0.9996 | NA |
| AAEL007954 | GSTE1 | 0.0485 | 4.2314 | 0.8993 | 0.9996 | glutathione transferase [Source:VB Community Annotation] |
| AAEL007218 | | -0.0361 | 5.2887 | 0.8995 | 0.9996 | Fatty acyl-CoA reductase [Source:UniProtKB/TrEMBL;Acc:Q173B8] |
| AAEL005980 | | 0.0618 | 2.1351 | 0.8995 | 0.9996 | |
| AAEL010721 | | -0.1521 | -2.0390 | 0.8995 | 0.9996 | reticulon/nogo receptor [Source:VB Community Annotation] |
| AAEL004342 | OBP18 | -0.1578 | 6.2701 | 0.8997 | 0.9996 | odorant binding protein OBP18 [Source:VB Community Annotation] |
| AAEL020476 | NA | -0.0356 | 7.2213 | 0.8997 | 0.9996 | NA |
| AAEL008721 | | 0.0612 | 1.6307 | 0.8997 | 0.9996 | zinc finger protein [Source:VB Community Annotation] |
| AAEL003970 | | -0.0902 | 2.4065 | 0.8998 | 0.9996 | amino acid transporter [Source:VB Community Annotation] |
| AAEL002142 | | -0.1262 | 3.2826 | 0.8999 | 0.9996 | |
| AAEL020008 | NA | -0.1531 | -0.0069 | 0.8999 | 0.9996 | NA |
| AAEL014589 | | 0.0547 | 3.4620 | 0.9001 | 0.9996 | reticulocalbin [Source:VB Community Annotation] |
| AAEL021533 | NA | -0.2203 | 0.0257 | 0.9001 | 0.9996 | NA |
| AAEL011871 | | 0.0525 | 10.0734 | 0.9002 | 0.9996 | cytochrome C1 [Source:VB Community Annotation] |
| AAEL004841 | | -0.0499 | 3.0067 | 0.9002 | 0.9996 | |
| AAEL007899 | | 0.0574 | 2.1703 | 0.9003 | 0.9996 | spermatogenesis associated factor [Source:VB Community Annotation] |

| | | | | | | |
|---|---|---|---|---|---|---|
| AAEL007315 | | -0.1397 | -2.1771 | 0.9004 | 0.9996 | symbol, putative [Source:VB Community Annotation] |
| AAEL001704 | | -0.3601 | 3.6715 | 0.9004 | 0.9996 | |
| AAEL008371 | | -0.0415 | 4.2154 | 0.9006 | 0.9996 | neurofibromin [Source:VB Community Annotation] |
| AAEL025258 | NA | -0.0540 | 2.7508 | 0.9006 | 0.9996 | NA |
| AAEL007776 | | -0.2268 | 8.2058 | 0.9006 | 0.9996 | |
| AAEL022367 | NA | -0.1278 | -0.3195 | 0.9008 | 0.9996 | NA |
| AAEL018236 | | -0.1521 | -1.9600 | 0.9009 | 0.9996 | |
| AAEL018211 | | -0.0862 | 7.1483 | 0.9010 | 0.9996 | |
| AAEL000661 | | 0.2075 | -0.5325 | 0.9012 | 0.9996 | t-cell specific transcription factor, tcf [Source:VB Community Annotation] |
| AAEL000713 | | -0.0481 | 7.4130 | 0.9012 | 0.9996 | reticulon/nogo [Source:VB Community Annotation] |
| AAEL010397 | | 0.0970 | 2.7647 | 0.9012 | 0.9996 | |
| AAEL014201 | | 0.0391 | 6.7982 | 0.9013 | 0.9996 | chaperone protein, putative [Source:VB Community Annotation] |
| AAEL021019 | NA | 0.1545 | -0.6274 | 0.9013 | 0.9996 | NA |
| AAEL026401 | NA | 0.0832 | 1.8487 | 0.9014 | 0.9996 | NA |
| AAEL010524 | | -0.0309 | 4.8736 | 0.9014 | 0.9996 | |
| AAEL004269 | | -0.2323 | 0.9997 | 0.9015 | 0.9996 | hippocalcin [Source:VB Community Annotation] |
| AAEL022351 | NA | 0.0378 | 5.0537 | 0.9018 | 0.9996 | NA |
| AAEL005155 | | 0.0456 | 3.2576 | 0.9019 | 0.9996 | |
| AAEL008095 | | 0.0533 | 3.2143 | 0.9019 | 0.9996 | |
| AAEL000663 | | -0.0489 | 7.1878 | 0.9019 | 0.9996 | |
| AAEL007509 | | 0.1170 | 3.4427 | 0.9020 | 0.9996 | neuroendocrine differentiation factor [Source:VB Community Annotation] |
| AAEL012746 | | -0.0390 | 6.2429 | 0.9020 | 0.9996 | chaperonin [Source:VB Community Annotation] |
| AAEL013862 | | 0.0387 | 2.6385 | 0.9020 | 0.9996 | arginyl-tRNA synthetase [Source:VB Community Annotation] |
| AAEL006403 | | -0.1521 | -2.9851 | 0.9022 | 0.9996 | trypsin-beta, putative [Source:VB Community Annotation] |
| AAEL024709 | NA | 0.0246 | 6.1859 | 0.9023 | 0.9996 | NA |
| AAEL002061 | | 0.1147 | 2.0545 | 0.9023 | 0.9996 | cation-transporting ATPase 13a1 (g-box binding protein) [Source:VB Community Annotation] |
| AAEL004426 | | -0.0302 | 5.0694 | 0.9024 | 0.9996 | Syntaxin 18, putative [Source:VB Community Annotation] |
| AAEL021056 | NA | 0.2189 | 4.0977 | 0.9024 | 0.9996 | NA |
| AAEL002786 | | 0.0569 | 2.6473 | 0.9024 | 0.9996 | |
| AAEL026570 | NA | -0.0784 | 5.8232 | 0.9024 | 0.9996 | NA |
| AAEL025226 | NA | -0.2676 | 2.8802 | 0.9025 | 0.9996 | NA |
| AAEL004053 | | -0.1248 | 0.6530 | 0.9026 | 0.9996 | Nucleoporin NUP53 [Source:UniProtKB/TrEMBL;Acc:Q17DT3] |
| AAEL006421 | | -0.1521 | -2.8982 | 0.9026 | 0.9996 | trypsin, putative [Source:VB Community Annotation] |
| AAEL003483 | | 0.0573 | 7.5370 | 0.9028 | 0.9996 | |
| AAEL011553 | | -0.1521 | -2.6248 | 0.9028 | 0.9996 | trypsin [Source:VB Community Annotation] |
| AAEL019535 | NA | -0.1625 | 2.3841 | 0.9028 | 0.9996 | NA |
| AAEL012264 | mRpL24 | -0.0542 | 6.6362 | 0.9028 | 0.9996 | mitochondrial ribosomal protein L24 [Source:VB Community Annotation] |
| AAEL000498 | | 0.2097 | -0.3777 | 0.9029 | 0.9996 | defective proboscis extension response, putative [Source:VB Community Annotation] |
| AAEL028108 | NA | 0.1783 | 0.2055 | 0.9029 | 0.9996 | NA |
| AAEL023560 | NA | -0.2047 | 2.3733 | 0.9030 | 0.9996 | NA |

| | | | | | | |
|---|---|---|---|---|---|---|
| AAEL009108 | | -0.2632 | 0.3339 | 0.9030 | 0.9996 | protease m1 zinc metalloprotease [Source:VB Community Annotation] |
| AAEL008367 | | 0.0405 | 7.1776 | 0.9030 | 0.9996 | fk506-binding protein [Source:VB Community Annotation] |
| AAEL003447 | | -0.0479 | 3.6704 | 0.9031 | 0.9996 | |
| AAEL019336 | U1 | -0.1521 | -2.7783 | 0.9032 | 0.9996 | U1 spliceosomal RNA [Source:RFAM;Acc:RF00003] |
| AAEL003293 | | -0.0438 | 4.1526 | 0.9035 | 0.9996 | GTP-binding protein [Source:VB Community Annotation] |
| AAEL000982 | Med7 | 0.0507 | 5.6383 | 0.9036 | 0.9996 | mediator of RNA polymerase II transcription subunit 7 (Med7) [Source:VB Community Annotation] |
| AAEL003449 | | 0.0352 | 3.6143 | 0.9037 | 0.9996 | structural maintenance of chromosomes 2 smc2 [Source:VB Community Annotation] |
| AAEL009610 | | -0.0463 | 2.7837 | 0.9038 | 0.9996 | |
| AAEL022770 | NA | 0.0631 | 2.3660 | 0.9040 | 0.9996 | NA |
| AAEL025872 | NA | -0.0346 | 7.9374 | 0.9042 | 0.9996 | NA |
| AAEL020205 | NA | 0.0441 | 6.3458 | 0.9043 | 0.9996 | NA |
| AAEL014603 | CYP9J30 | 0.1490 | -0.8719 | 0.9043 | 0.9996 | cytochrome P450 [Source:VB Community Annotation] |
| AAEL013207 | | -0.0342 | 5.5792 | 0.9043 | 0.9996 | |
| AAEL019865 | NA | -0.1910 | 2.3676 | 0.9043 | 0.9996 | NA |
| AAEL007830 | CYP4H29 | 0.0458 | 5.1977 | 0.9045 | 0.9996 | cytochrome P450 [Source:VB Community Annotation] |
| AAEL008401 | | 0.2041 | 0.8779 | 0.9046 | 0.9996 | |
| AAEL000088 | | 0.0434 | 3.7256 | 0.9046 | 0.9996 | brefeldin A-sensitive peripheral Golgi protein, putative [Source:VB Community Annotation] |
| AAEL020808 | NA | -0.1521 | -2.2573 | 0.9047 | 0.9996 | NA |
| AAEL001196 | | -0.1557 | 4.1600 | 0.9047 | 0.9996 | cadherin [Source:VB Community Annotation] |
| AAEL020824 | NA | -0.1381 | -2.1942 | 0.9048 | 0.9996 | NA |
| AAEL012057 | | -0.1211 | 3.8333 | 0.9049 | 0.9996 | enhancer of polycomb [Source:VB Community Annotation] |
| AAEL002514 | | -0.0473 | 2.5226 | 0.9049 | 0.9996 | |
| AAEL019495 | NA | -0.1805 | 1.2539 | 0.9050 | 0.9996 | NA |
| AAEL001787 | | -0.0475 | 4.1243 | 0.9051 | 0.9996 | DNA photolyase [Source:VB Community Annotation] |
| AAEL002783 | mRpL37 | -0.0463 | 5.1720 | 0.9053 | 0.9996 | 39S mitochondrial ribosomal protein L37 [Source:VB Community Annotation] |
| AAEL021698 | NA | -0.2567 | -0.6337 | 0.9053 | 0.9996 | NA |
| AAEL002704 | SRPN23 | -0.1910 | 5.8870 | 0.9056 | 0.9996 | Serine Protease Inhibitor (serpin) homologue [Source:VB Community Annotation] |
| AAEL010676 | | 0.0458 | 4.9611 | 0.9057 | 0.9996 | regulator of g protein signaling [Source:VB Community Annotation] |
| AAEL011510 | | 0.1870 | 5.7776 | 0.9058 | 0.9996 | multiple inositol polyphosphate phosphatase [Source:VB Community Annotation] |
| AAEL009269 | | 0.2206 | 0.0023 | 0.9059 | 0.9996 | |
| AAEL004441 | | -0.0384 | 4.1988 | 0.9060 | 0.9996 | importin 9 (imp9) (ran-binding protein 9) [Source:VB Community Annotation] |
| AAEL009338 | CTL10 | 0.1328 | -0.6562 | 0.9060 | 0.9996 | C-Type Lectin (CTL). [Source:VB Community Annotation] |
| AAEL008705 | | 0.0551 | 4.4565 | 0.9061 | 0.9996 | |
| AAEL023413 | NA | -0.1561 | 0.8616 | 0.9061 | 0.9996 | NA |
| AAEL005991 | | -0.0471 | 5.8302 | 0.9061 | 0.9996 | tricarboxylate transport protein [Source:VB Community Annotation] |
| AAEL023052 | NA | -0.1521 | -2.9221 | 0.9064 | 0.9996 | NA |
| AAEL014965 | | -0.0799 | 8.2097 | 0.9064 | 0.9996 | nova [Source:VB Community Annotation] |
| AAEL026799 | NA | 0.0330 | 4.1374 | 0.9065 | 0.9996 | NA |
| AAEL026706 | NA | -0.0703 | 1.4518 | 0.9066 | 0.9996 | NA |
| AAEL023574 | NA | 0.0409 | 4.0994 | 0.9066 | 0.9996 | NA |

| | | | | | | |
|---|---|---|---|---|---|---|
| AAEL003872 | Tctp | 0.0340 | 11.0710 | 0.9067 | 0.9996 | translationally-controlled tumor protein homolog (TCTP) [Source:VB Community Annotation] |
| AAEL022600 | NA | -0.1607 | 0.6661 | 0.9067 | 0.9996 | NA |
| AAEL015345 | | -0.1521 | -2.6932 | 0.9068 | 0.9996 | |
| AAEL007018 | | -0.0420 | 3.3429 | 0.9070 | 0.9996 | udp-glucose 4-epimerase [Source:VB Community Annotation] |
| AAEL005655 | | 0.0796 | 3.5284 | 0.9072 | 0.9996 | sorting nexin [Source:VB Community Annotation] |
| AAEL009185 | | 0.0418 | 10.4062 | 0.9073 | 0.9996 | arginine or creatine kinase [Source:VB Community Annotation] |
| AAEL021015 | NA | 0.0475 | 1.7267 | 0.9073 | 0.9996 | NA |
| AAEL028752 | NA | -0.0697 | 1.2260 | 0.9073 | 0.9996 | NA |
| AAEL001105 | | 0.0327 | 4.8779 | 0.9073 | 0.9996 | von Hippel-Lindau disease tumor suppressor, putative [Source:VB Community Annotation] |
| AAEL019934 | NA | -0.1265 | 5.1018 | 0.9073 | 0.9996 | NA |
| AAEL020508 | NA | -0.0710 | 5.3651 | 0.9073 | 0.9996 | NA |
| AAEL026701 | NA | 0.0499 | 2.9331 | 0.9074 | 0.9996 | NA |
| AAEL001187 | | 0.0339 | 4.7631 | 0.9075 | 0.9996 | |
| AAEL026960 | NA | -0.2785 | 2.3200 | 0.9076 | 0.9996 | NA |
| AAEL011177 | | 0.0851 | 5.1200 | 0.9077 | 0.9996 | |
| AAEL025123 | NA | -0.1590 | 3.7527 | 0.9079 | 0.9996 | NA |
| AAEL004301 | | 0.0621 | 10.2892 | 0.9080 | 0.9996 | |
| AAEL000536 | | -0.0598 | 4.6965 | 0.9081 | 0.9996 | |
| AAEL012156 | | -0.0395 | 2.9455 | 0.9081 | 0.9996 | n6-adenosine-methyltransferase ime4 [Source:VB Community Annotation] |
| AAEL003654 | | 0.0485 | 2.6478 | 0.9081 | 0.9996 | |
| AAEL027545 | NA | 0.1015 | 4.0241 | 0.9082 | 0.9996 | NA |
| AAEL011657 | | -0.0455 | 7.0192 | 0.9082 | 0.9996 | importin alpha [Source:VB Community Annotation] |
| AAEL010479 | | -0.0780 | 7.1927 | 0.9083 | 0.9996 | sugar transporter [Source:VB Community Annotation] |
| AAEL003076 | | -0.0586 | 7.9696 | 0.9083 | 0.9996 | glucosyl/glucuronosyl transferases [Source:VB Community Annotation] |
| AAEL009802 | | 0.1627 | -1.3616 | 0.9083 | 0.9996 | cuticle protein, putative [Source:VB Community Annotation] |
| AAEL006323 | | -0.1102 | 6.6498 | 0.9085 | 0.9996 | |
| AAEL013001 | | 0.0778 | 3.5130 | 0.9087 | 0.9996 | |
| AAEL003184 | | 0.0491 | 7.1830 | 0.9088 | 0.9996 | mfs transporter [Source:VB Community Annotation] |
| AAEL018703 | | 0.0502 | 3.4717 | 0.9088 | 0.9996 | |
| AAEL009075 | | -0.0386 | 3.7901 | 0.9090 | 0.9996 | |
| AAEL008580 | | 0.1129 | -0.4728 | 0.9091 | 0.9996 | |
| AAEL020135 | NA | 0.0854 | 2.3442 | 0.9091 | 0.9996 | NA |
| AAEL014140 | CLIPB24 | 0.0801 | 4.2739 | 0.9091 | 0.9996 | Clip-Domain Serine Protease  family B. [Source:VB Community Annotation] |
| AAEL015059 | | -0.0371 | 4.5563 | 0.9091 | 0.9996 | |
| AAEL014990 | | 0.0430 | 6.5197 | 0.9092 | 0.9996 | |
| AAEL010068 | | 0.1504 | 3.8849 | 0.9093 | 0.9996 | |
| AAEL012273 | | 0.0388 | 3.7601 | 0.9094 | 0.9996 | |
| AAEL007897 | SPZ4 | -0.1152 | 0.8707 | 0.9094 | 0.9996 | spaetzle-like cytokine [Source:VB Community Annotation] |
| AAEL025030 | NA | -0.0442 | 3.0059 | 0.9095 | 0.9996 | NA |
| AAEL018306 | | 0.1035 | 3.7353 | 0.9095 | 0.9996 | |

| | | | | | | |
|---|---|---|---|---|---|---|
| AAEL002588 | | -0.1758 | -1.7983 | 0.9101 | 0.9996 | |
| AAEL019841 | NA | -0.2009 | 5.1358 | 0.9102 | 0.9996 | NA |
| AAEL010021 | | 0.1605 | 2.9284 | 0.9102 | 0.9996 | serine/threonine-protein kinase 38 (ndr2 protein kinase) [Source:VB Community Annotation] |
| AAEL008454 | | -0.0252 | 6.5411 | 0.9102 | 0.9996 | |
| AAEL025582 | NA | -0.1383 | -1.5111 | 0.9103 | 0.9996 | NA |
| AAEL012835 | | 0.0417 | 3.7742 | 0.9104 | 0.9996 | 85 kda calcium-independent phospholipase A2 (ipla2) [Source:VB Community Annotation] |
| AAEL002219 | | -0.1347 | -0.2076 | 0.9104 | 0.9996 | zinc finger protein, putative [Source:VB Community Annotation] |
| AAEL022912 | NA | -0.1521 | -2.7133 | 0.9104 | 0.9996 | NA |
| AAEL003396 | RpL32 | 0.0326 | 10.1572 | 0.9105 | 0.9996 | 60S ribosomal protein L32 [Source:UniProtKB/TrEMBL;Acc:Q1HR34] |
| AAEL014158 | | 0.0392 | 7.3822 | 0.9106 | 0.9996 | |
| AAEL000649 | | 0.1840 | 3.0576 | 0.9106 | 0.9996 | |
| AAEL020727 | NA | -0.1521 | -2.1677 | 0.9108 | 0.9996 | NA |
| AAEL028237 | NA | -0.0453 | 4.2972 | 0.9108 | 0.9996 | NA |
| AAEL018120 | | 0.1788 | 3.1976 | 0.9109 | 0.9996 | Ribosomal protein S6 kinase [Source:UniProtKB/TrEMBL;Acc:Q17ND6] |
| AAEL001724 | GPRNNB3 | 0.0872 | 1.6406 | 0.9112 | 0.9996 | GPCR Orphan/Putative Class B Family [Source:VB Community Annotation] |
| AAEL012344 | | 0.1484 | -1.2754 | 0.9114 | 0.9996 | lipase 1 precursor [Source:VB Community Annotation] |
| AAEL012583 | | -0.0482 | 3.4922 | 0.9116 | 0.9996 | tuberin [Source:VB Community Annotation] |
| AAEL007476 | | -0.0319 | 4.4088 | 0.9116 | 0.9996 | makorin [Source:VB Community Annotation] |
| AAEL011405 | | 0.1560 | -1.1780 | 0.9118 | 0.9996 | |
| AAEL010270 | | 0.0916 | 1.8733 | 0.9119 | 0.9996 | |
| AAEL011271 | | 0.1425 | -1.5285 | 0.9120 | 0.9996 | programmed cell death 6-interacting protein [Source:VB Community Annotation] |
| AAEL014453 | | 0.0595 | 3.9040 | 0.9121 | 0.9996 | |
| AAEL013229 | | 0.0468 | 8.7574 | 0.9123 | 0.9996 | Tubulin alpha chain [Source:UniProtKB/TrEMBL;Acc:Q16JS3] |
| AAEL027978 | NA | -0.1784 | 2.7787 | 0.9124 | 0.9996 | NA |
| AAEL021982 | NA | 0.1621 | 3.9077 | 0.9126 | 0.9996 | NA |
| AAEL023347 | NA | 0.0316 | 6.4665 | 0.9126 | 0.9996 | NA |
| AAEL025802 | NA | 0.0590 | 3.5212 | 0.9127 | 0.9996 | NA |
| AAEL012431 | | 0.1416 | -0.2660 | 0.9127 | 0.9996 | AMP dependent ligase [Source:VB Community Annotation] |
| AAEL003613 | sage | 0.1114 | 2.1828 | 0.9127 | 0.9996 | |
| AAEL000191 | | -0.0297 | 5.4822 | 0.9127 | 0.9996 | |
| AAEL026740 | NA | 0.0596 | 4.5111 | 0.9127 | 0.9996 | NA |
| AAEL026070 | NA | 0.0507 | 1.8173 | 0.9128 | 0.9996 | NA |
| AAEL021278 | NA | 0.1304 | -0.6259 | 0.9128 | 0.9996 | NA |
| AAEL014754 | | -0.0567 | 2.4988 | 0.9128 | 0.9996 | |
| AAEL025678 | NA | -0.1572 | 4.2755 | 0.9129 | 0.9996 | NA |
| AAEL011991 | CLIPC1 | -0.0558 | 4.4393 | 0.9129 | 0.9996 | Clip-Domain Serine Protease family C. [Source:VB Community Annotation] |
| AAEL008107 | | 0.0350 | 5.9656 | 0.9129 | 0.9996 | f14p3.9 protein (auxin transport protein) [Source:VB Community Annotation] |
| AAEL004167 | | 0.0377 | 5.3099 | 0.9130 | 0.9996 | zinc finger protein [Source:VB Community Annotation] |
| AAEL006702 | | 0.2216 | 1.6086 | 0.9130 | 0.9996 | fibrinogen and fibronectin [Source:VB Community Annotation] |
| AAEL023587 | NA | -0.0414 | 7.2610 | 0.9131 | 0.9996 | NA |

| | | | | | | |
|---|---|---|---|---|---|---|
| AAEL008152 | | -0.0636 | 3.9966 | 0.9132 | 0.9996 | short-chain dehydrogenase [Source:VB Community Annotation] |
| AAEL021618 | NA | -0.1731 | 3.8467 | 0.9134 | 0.9996 | NA |
| AAEL000046 | | 0.0480 | 2.3053 | 0.9134 | 0.9996 | |
| AAEL017120 | | 0.0339 | 3.0902 | 0.9136 | 0.9996 | |
| AAEL008426 | | -0.1663 | 3.1134 | 0.9136 | 0.9996 | broad-complex core-protein [Source:VB Community Annotation] |
| AAEL009330 | | 0.1720 | 2.9649 | 0.9139 | 0.9996 | carbonic anhydrase II, putative [Source:VB Community Annotation] |
| AAEL014501 | Med10 | -0.0493 | 6.4662 | 0.9140 | 0.9996 | Mediator of RNA polymerase II transcription subunit 10 (Med10) [Source:VB Community Annotation] |
| AAEL002903 | p110 | 0.1833 | 3.6130 | 0.9141 | 0.9996 | phosphatidylinositol 3-kinase catalytic subunit alpha, beta, delta [Source:VB Community Annotation] |
| AAEL019801 | NA | -0.0283 | 4.8105 | 0.9141 | 0.9996 | NA |
| AAEL002737 | | 0.0503 | 11.8612 | 0.9141 | 0.9996 | cytochrome c oxidase, subunit VIIC, putative [Source:VB Community Annotation] |
| AAEL008293 | | -0.1004 | -0.1521 | 0.9141 | 0.9996 | pupal cuticle protein 78E, putative [Source:VB Community Annotation] |
| AAEL001407 | | 0.0535 | 2.8674 | 0.9143 | 0.9996 | cdk4/6 [Source:VB Community Annotation] |
| AAEL008482 | | 0.0569 | 2.5241 | 0.9143 | 0.9996 | |
| AAEL024490 | NA | -0.1622 | 2.3977 | 0.9143 | 0.9996 | NA |
| AAEL007812 | CYP4H32 | 0.1824 | -1.4817 | 0.9145 | 0.9996 | cytochrome P450 [Source:VB Community Annotation] |
| AAEL025155 | NA | 0.1084 | -1.0841 | 0.9146 | 0.9996 | NA |
| AAEL010445 | | 0.1974 | 2.0633 | 0.9146 | 0.9996 | |
| AAEL004010 | | 0.0211 | 5.9331 | 0.9150 | 0.9996 | splicing factor, putative [Source:VB Community Annotation] |
| AAEL020579 | NA | -0.1237 | 3.7138 | 0.9150 | 0.9996 | NA |
| AAEL022568 | NA | 0.1458 | -0.2367 | 0.9150 | 0.9996 | NA |
| AAEL006118 | mRpL55 | 0.0317 | 5.1735 | 0.9151 | 0.9996 | mitochondrial ribosomal protein, L55 [Source:VB Community Annotation] |
| AAEL007205 | | -0.1097 | 1.8784 | 0.9151 | 0.9996 | gata-binding factor-c (transcription factor gata-c) [Source:VB Community Annotation] |
| AAEL008945 | | 0.0398 | 3.4048 | 0.9152 | 0.9996 | |
| AAEL012989 | | -0.0361 | 3.6037 | 0.9153 | 0.9996 | |
| AAEL011211 | | -0.1586 | -2.1097 | 0.9154 | 0.9996 | |
| AAEL019782 | NA | 0.0748 | 1.2668 | 0.9154 | 0.9996 | NA |
| AAEL011198 | mRpL38 | -0.0518 | 5.3493 | 0.9154 | 0.9996 | mitochondrial ribosomal protein, L38, putative [Source:VB Community Annotation] |
| AAEL007314 | | -0.1475 | -1.2790 | 0.9154 | 0.9996 | |
| AAEL004339 | OBP17 | -0.0629 | 5.6910 | 0.9154 | 0.9996 | odorant binding protein OBP17 [Source:VB Community Annotation] |
| AAEL004524 | CLIPC5B | 0.0658 | 5.8700 | 0.9154 | 0.9996 | Clip-Domain Serine Protease family C. [Source:VB Community Annotation] |
| AAEL007763 | | -0.0294 | 3.9825 | 0.9155 | 0.9996 | vacuole membrane protein [Source:VB Community Annotation] |
| AAEL004282 | | -0.0286 | 6.0381 | 0.9155 | 0.9996 | protein-(glutamine-N5) methyl transferase, putative [Source:VB Community Annotation] |
| AAEL019484 | NA | 0.1121 | -2.1238 | 0.9155 | 0.9996 | NA |
| AAEL006945 | | 0.0399 | 4.1670 | 0.9155 | 0.9996 | golgi SNAP receptor complex member, putative [Source:VB Community Annotation] |
| AAEL002866 | | -0.0583 | 5.6693 | 0.9155 | 0.9996 | cyclin l [Source:VB Community Annotation] |
| AAEL018864 | tRNA-Lys | -0.1521 | -2.0892 | 0.9156 | 0.9996 | |
| AAEL027601 | NA | 0.1434 | -1.2450 | 0.9157 | 0.9996 | NA |
| AAEL010710 | | -0.1499 | 3.0422 | 0.9157 | 0.9996 | gamma-aminobutyric-acid receptor a beta subunit 1, 2, 3, [Source:VB Community Annotation] |
| AAEL024303 | NA | 0.1779 | 5.0104 | 0.9159 | 0.9996 | NA |
| AAEL012095 | | -0.0237 | 6.9743 | 0.9159 | 0.9996 | 26S protease regulatory subunit [Source:VB Community Annotation] |

| | | | | | | |
|---|---|---|---|---|---|---|
| AAEL000581 | | -0.0589 | 2.4764 | 0.9159 | 0.9996 | |
| AAEL001582 | | 0.1663 | 3.0824 | 0.9159 | 0.9996 | kinesin [Source:VB Community Annotation] |
| AAEL014773 | | 0.1680 | -0.9995 | 0.9161 | 0.9996 | |
| AAEL020560 | NA | 0.1199 | -1.3132 | 0.9162 | 0.9996 | NA |
| AAEL008962 | | -0.0344 | 6.6996 | 0.9163 | 0.9996 | |
| AAEL019533 | NA | 0.0331 | 6.1643 | 0.9163 | 0.9996 | NA |
| AAEL000840 | | -0.0454 | 7.1528 | 0.9164 | 0.9996 | skeletal muscle/kidney enriched inositol 5-phosphatase [Source:VB Community Annotation] |
| AAEL000516 | | -0.0474 | 6.0654 | 0.9166 | 0.9996 | juvenile hormone-inducible protein, putative [Source:VB Community Annotation] |
| AAEL026441 | NA | -0.1071 | -0.5310 | 0.9166 | 0.9996 | NA |
| AAEL020561 | NA | 0.1308 | -2.3311 | 0.9168 | 0.9996 | NA |
| AAEL007332 | | 0.0310 | 5.8447 | 0.9169 | 0.9996 | cornichon protein, putative [Source:VB Community Annotation] |
| AAEL002023 | | -0.0608 | 9.8804 | 0.9169 | 0.9996 | imaginal disc growth factor [Source:VB Community Annotation] |
| AAEL025729 | NA | 0.1126 | 1.4888 | 0.9169 | 0.9996 | NA |
| AAEL006566 | | -0.0312 | 5.0555 | 0.9169 | 0.9996 | suppressor of ty [Source:VB Community Annotation] |
| AAEL025463 | NA | -0.0429 | 3.9039 | 0.9169 | 0.9996 | NA |
| AAEL007130 | | 0.0488 | 4.3916 | 0.9170 | 0.9996 | leucyl-tRNA synthetase [Source:VB Community Annotation] |
| AAEL001677 | | 0.1686 | 3.8027 | 0.9171 | 0.9996 | |
| AAEL013256 | | 0.0214 | 6.4538 | 0.9171 | 0.9996 | |
| AAEL022797 | NA | 0.1788 | -1.0579 | 0.9172 | 0.9996 | NA |
| AAEL024837 | NA | 0.0415 | 6.1076 | 0.9173 | 0.9996 | NA |
| AAEL007104 | | -0.0822 | 3.3600 | 0.9173 | 0.9996 | voltage-gated potassium channel [Source:VB Community Annotation] |
| AAEL019671 | NA | -0.0699 | 5.2536 | 0.9175 | 0.9996 | NA |
| AAEL025109 | NA | 0.0545 | 1.1052 | 0.9176 | 0.9996 | NA |
| AAEL003403 | | -0.1139 | 0.0471 | 0.9176 | 0.9996 | |
| AAEL023657 | NA | 0.0668 | 3.3741 | 0.9176 | 0.9996 | NA |
| AAEL007926 | | 0.1830 | -0.1870 | 0.9177 | 0.9996 | retinoid-inducible serine carboxypeptidase (serine carboxypeptidase [Source:VB Community Annotation] |
| AAEL005353 | | 0.0324 | 7.5486 | 0.9177 | 0.9996 | Sugar transporter SWEET [Source:UniProtKB/TrEMBL;Acc:Q17A97] |
| AAEL026365 | NA | -0.1397 | -1.9919 | 0.9178 | 0.9996 | NA |
| AAEL014296 | | 0.0302 | 6.1830 | 0.9179 | 0.9996 | major sperm protein [Source:VB Community Annotation] |
| AAEL007290 | | -0.0959 | 4.7033 | 0.9179 | 0.9996 | |
| AAEL010269 | | -0.0561 | 7.6399 | 0.9181 | 0.9996 | venom allergen [Source:VB Community Annotation] |
| AAEL010669 | | -0.0786 | 0.7839 | 0.9182 | 0.9996 | olfactory receptor, putative [Source:VB Community Annotation] |
| AAEL017000 | Or97 | -0.1005 | -0.8356 | 0.9182 | 0.9996 | odorant receptor - partial [Source:VB Community Annotation] |
| AAEL000563 | mosGCTL-1 | -0.0688 | 3.5550 | 0.9183 | 0.9996 | C-Type Lectin (CTL) - mannose binding. [Source:VB Community Annotation] |
| AAEL003794 | | -0.0427 | 4.3394 | 0.9183 | 0.9996 | homeobox protein nk-2 [Source:VB Community Annotation] |
| AAEL008056 | | -0.0345 | 4.1713 | 0.9184 | 0.9996 | hyperpolarization activated cyclic nucleotide-gated potassium channel [Source:VB Community Annotation] |
| AAEL019431 | NA | -0.1678 | 3.6750 | 0.9186 | 0.9996 | NA |
| AAEL026262 | NA | -0.1397 | -1.9364 | 0.9187 | 0.9996 | NA |
| AAEL022894 | NA | -0.1521 | -2.7118 | 0.9188 | 0.9996 | NA |
| AAEL007518 | | -0.0873 | 9.5373 | 0.9191 | 0.9996 | complexin, putative [Source:VB Community Annotation] |

| | | | | | | |
|---|---|---|---|---|---|---|
| AAEL013552 | | -0.0428 | 1.6750 | 0.9192 | 0.9996 | |
| AAEL025638 | NA | 0.1695 | 3.2404 | 0.9192 | 0.9996 | NA |
| AAEL018342 | | -0.1011 | 5.2282 | 0.9192 | 0.9996 | |
| AAEL017866 | U11 | -0.0715 | -0.0537 | 0.9193 | 0.9996 | U11 spliceosomal RNA [Source:RFAM;Acc:RF00548] |
| AAEL001830 | | -0.0299 | 4.9205 | 0.9194 | 0.9996 | geranylgeranyl transferase type i beta subunit [Source:VB Community Annotation] |
| AAEL009194 | | 0.1344 | 2.0483 | 0.9195 | 0.9996 | |
| AAEL014441 | | 0.1504 | 1.9799 | 0.9195 | 0.9996 | |
| AAEL003393 | | 0.0466 | 9.6581 | 0.9196 | 0.9996 | ATP synthase beta subunit [Source:VB Community Annotation] |
| AAEL022570 | NA | -0.1110 | 5.1055 | 0.9196 | 0.9996 | NA |
| AAEL025092 | NA | -0.1288 | -1.9202 | 0.9196 | 0.9996 | NA |
| AAEL025317 | NA | 0.0838 | 6.2690 | 0.9196 | 0.9996 | NA |
| AAEL001580 | | 0.0400 | 3.5360 | 0.9197 | 0.9996 | otefin, putative [Source:VB Community Annotation] |
| AAEL000533 | CTL16 | 0.1856 | 9.8334 | 0.9198 | 0.9996 | C-Type Lectin (CTL16) [Source:VB Community Annotation] |
| AAEL004709 | | 0.0306 | 6.2404 | 0.9199 | 0.9996 | protein phosphatase type 2c [Source:VB Community Annotation] |
| AAEL024161 | NA | 0.0931 | 9.1000 | 0.9200 | 0.9996 | NA |
| AAEL010119 | | 0.0775 | 2.9601 | 0.9204 | 0.9996 | ER-derived vesicles protein ERV14, putative [Source:VB Community Annotation] |
| AAEL024434 | NA | 0.0313 | 10.2986 | 0.9205 | 0.9996 | NA |
| AAEL008013 | OBP38 | -0.0509 | 6.7957 | 0.9205 | 0.9996 | odorant binding protein OBP38 [Source:VB Community Annotation] |
| AAEL009660 | | -0.1475 | -2.0617 | 0.9206 | 0.9996 | |
| AAEL018255 | | 0.1026 | -0.3146 | 0.9206 | 0.9996 | |
| AAEL024588 | NA | -0.0437 | 3.0627 | 0.9207 | 0.9996 | NA |
| AAEL021439 | NA | -0.1288 | -2.6914 | 0.9208 | 0.9996 | NA |
| AAEL012025 | mRpL45 | 0.0359 | 5.0972 | 0.9209 | 0.9996 | mitochondrial ribosomal protein, L45 [Source:VB Community Annotation] |
| AAEL008623 | | -0.0705 | 2.8886 | 0.9209 | 0.9996 | |
| AAEL003641 | | 0.0662 | 1.1804 | 0.9210 | 0.9996 | sodium/chloride dependent amino acid transporter [Source:VB Community Annotation] |
| AAEL003832 | DEFC | -0.1364 | 7.4887 | 0.9210 | 0.9996 | defensin anti-microbial peptide [Source:VB Community Annotation] |
| AAEL003490 | | 0.0432 | 2.4771 | 0.9210 | 0.9996 | |
| AAEL002642 | | -0.1838 | 2.0411 | 0.9211 | 0.9996 | tripartite motif protein trim9 [Source:VB Community Annotation] |
| AAEL013653 | | -0.0605 | 5.7253 | 0.9212 | 0.9996 | tata-box binding protein [Source:VB Community Annotation] |
| AAEL006693 | | 0.0256 | 6.4518 | 0.9213 | 0.9996 | Uroporphyrinogen decarboxylase [Source:UniProtKB/TrEMBL;Acc:Q175D5] |
| AAEL001946 | | -0.0713 | 6.5011 | 0.9214 | 0.9996 | four and a half lim domains [Source:VB Community Annotation] |
| AAEL019708 | NA | 0.0704 | 4.5412 | 0.9214 | 0.9996 | NA |
| AAEL010859 | | 0.0652 | 1.0628 | 0.9215 | 0.9996 | |
| AAEL010541 | | 0.0439 | 5.9823 | 0.9216 | 0.9996 | |
| AAEL000163 | | -0.0403 | 6.5847 | 0.9217 | 0.9996 | mitochondrial inner membrane protein translocase, 22kD-subunit, putative [Source:VB Community Annotation] |
| AAEL006262 | | -0.0257 | 4.3193 | 0.9217 | 0.9996 | mitochondrial carrier protein [Source:VB Community Annotation] |
| AAEL026833 | NA | 0.0461 | 5.2727 | 0.9219 | 0.9996 | NA |
| AAEL014541 | | -0.0727 | 0.9076 | 0.9220 | 0.9996 | maltose phosphorylase [Source:VB Community Annotation] |
| AAEL005605 | paps | -0.0675 | 3.3405 | 0.9225 | 0.9996 | adenylsulphate kinase [Source:VB Community Annotation] |
| AAEL001155 | | -0.0538 | 1.8566 | 0.9227 | 0.9996 | |

| | | | | | | |
|---|---|---|---|---|---|---|
| AAEL019472 | NA | 0.0918 | 0.8373 | 0.9228 | 0.9996 | NA |
| AAEL019560 | NA | 0.0278 | 5.4475 | 0.9228 | 0.9996 | NA |
| AAEL001691 | | 0.0385 | 5.2090 | 0.9229 | 0.9996 | adenylate cyclase [Source:VB Community Annotation] |
| AAEL001742 | | 0.0303 | 4.7722 | 0.9229 | 0.9996 | |
| AAEL020593 | NA | 0.2151 | 3.9253 | 0.9231 | 0.9996 | NA |
| AAEL003713 | | 0.0240 | 5.0353 | 0.9232 | 0.9996 | leucine-rich transmembrane protein [Source:VB Community Annotation] |
| AAEL024015 | NA | -0.1638 | -1.1013 | 0.9233 | 0.9996 | NA |
| AAEL010387 | | 0.0312 | 5.2409 | 0.9233 | 0.9996 | pre-mRNA splicing factor [Source:VB Community Annotation] |
| AAEL018166 | | 0.0421 | 4.2149 | 0.9233 | 0.9996 | |
| AAEL004645 | | -0.0518 | 1.9429 | 0.9234 | 0.9996 | |
| AAEL004003 | | -0.1439 | -1.2941 | 0.9235 | 0.9996 | glucose dehydrogenase [Source:VB Community Annotation] |
| AAEL025020 | NA | 0.0819 | 0.4462 | 0.9237 | 0.9996 | NA |
| AAEL022126 | NA | -0.0507 | 3.2690 | 0.9237 | 0.9996 | NA |
| AAEL022098 | NA | -0.0285 | 5.3261 | 0.9237 | 0.9996 | NA |
| AAEL021608 | NA | -0.1159 | -0.9117 | 0.9239 | 0.9996 | NA |
| AAEL028071 | NA | 0.1635 | -2.3200 | 0.9239 | 0.9996 | NA |
| AAEL022913 | NA | -0.1882 | 0.6638 | 0.9240 | 0.9996 | NA |
| AAEL011390 | mRPL9 | 0.0379 | 5.2503 | 0.9240 | 0.9996 | mitochondrial ribosomal protein, L9, putative [Source:VB Community Annotation] |
| AAEL001379 | | -0.0382 | 4.4712 | 0.9241 | 0.9996 | |
| AAEL018343 | | 0.0631 | 3.2368 | 0.9242 | 0.9996 | |
| AAEL019440 | NA | -0.1716 | 2.0579 | 0.9244 | 0.9996 | NA |
| AAEL000797 | | 0.0682 | 3.3138 | 0.9244 | 0.9996 | dimethylaniline monooxygenase [Source:VB Community Annotation] |
| AAEL018290 | | 0.1563 | -0.1868 | 0.9245 | 0.9996 | |
| AAEL014146 | | -0.0402 | 4.5564 | 0.9247 | 0.9996 | |
| AAEL020826 | NA | -0.0798 | -0.3766 | 0.9248 | 0.9996 | NA |
| AAEL026043 | NA | -0.0255 | 4.4472 | 0.9249 | 0.9996 | NA |
| AAEL020005 | NA | 0.0840 | 0.2072 | 0.9250 | 0.9996 | NA |
| AAEL011399 | | -0.0355 | 3.9290 | 0.9251 | 0.9996 | |
| AAEL003731 | | -0.1103 | 3.0534 | 0.9251 | 0.9996 | nuclear body associated kinase, putative [Source:VB Community Annotation] |
| AAEL009729 | | 0.1021 | 2.0839 | 0.9253 | 0.9996 | |
| AAEL003750 | | -0.0340 | 8.7379 | 0.9253 | 0.9996 | |
| AAEL011320 | | -0.1589 | -0.3046 | 0.9254 | 0.9996 | G-protein, gamma-subunit, putative [Source:VB Community Annotation] |
| AAEL007720 | | -0.1030 | -1.0598 | 0.9254 | 0.9996 | |
| AAEL000081 | | 0.0505 | 4.0915 | 0.9254 | 0.9996 | peroxisome assembly factor-2 (peroxisomal-type ATPase 1) [Source:VB Community Annotation] |
| AAEL010082 | | 0.0844 | 0.2658 | 0.9255 | 0.9996 | |
| AAEL026670 | NA | -0.0388 | 4.7962 | 0.9256 | 0.9996 | NA |
| AAEL012306 | APG7B | -0.0259 | 4.4587 | 0.9257 | 0.9996 | autophagy related gene [Source:VB Community Annotation] |
| AAEL001812 | | -0.0377 | 4.0212 | 0.9257 | 0.9996 | organic anion transporter [Source:VB Community Annotation] |
| AAEL006860 | RpS28 | -0.0349 | 11.5814 | 0.9257 | 0.9996 | 40S ribosomal protein S28 [Source:VB Community Annotation] |
| AAEL014243 | | 0.0634 | 2.5812 | 0.9258 | 0.9996 | |

| | | | | | | |
|---|---|---|---|---|---|---|
| AAEL001965 | | 0.0436 | 10.3564 | 0.9259 | 0.9996 | imaginal disc growth factor [Source:VB Community Annotation] |
| AAEL008793 | | -0.0757 | 4.1247 | 0.9259 | 0.9996 | |
| AAEL013154 | | -0.0725 | 1.3650 | 0.9260 | 0.9996 | |
| AAEL004069 | mRpS14 | 0.0377 | 6.8802 | 0.9261 | 0.9996 | mitochondrial 28S ribosomal protein S14 (s14mt) [Source:VB Community Annotation] |
| AAEL025006 | NA | -0.0747 | 4.0944 | 0.9262 | 0.9996 | NA |
| AAEL000474 | | -0.0324 | 5.9545 | 0.9263 | 0.9996 | |
| AAEL012514 | | 0.0299 | 3.3047 | 0.9263 | 0.9996 | translation initiation factor 2b, delta subunit [Source:VB Community Annotation] |
| AAEL027068 | NA | -0.1428 | 2.9840 | 0.9264 | 0.9996 | NA |
| AAEL007387 | | -0.0454 | 3.8233 | 0.9265 | 0.9996 | |
| AAEL012135 | GALE2 | -0.1433 | 2.3425 | 0.9265 | 0.9996 | Galectin [Source:UniProtKB/TrEMBL;Acc:Q16MZ7] |
| AAEL008097 | | 0.1063 | 3.7972 | 0.9265 | 0.9996 | trypsin-eta, putative [Source:VB Community Annotation] |
| AAEL021775 | NA | 0.0928 | -0.4523 | 0.9266 | 0.9996 | NA |
| AAEL024889 | NA | -0.1397 | -1.7577 | 0.9267 | 0.9996 | NA |
| AAEL010108 | | -0.0723 | 3.7168 | 0.9267 | 0.9996 | sex-determining protein fem-1 [Source:VB Community Annotation] |
| AAEL007752 | | -0.0224 | 9.4444 | 0.9267 | 0.9996 | cytochrome c oxidase, subunit VIIA, putative [Source:VB Community Annotation] |
| AAEL010407 | | 0.0364 | 3.3125 | 0.9268 | 0.9996 | |
| AAEL007962 | GSTE4 | -0.0411 | 7.1378 | 0.9269 | 0.9996 | glutathione transferase [Source:VB Community Annotation] |
| AAEL018339 | | 0.0192 | 5.4026 | 0.9271 | 0.9996 | |
| AAEL010308 | | 0.0440 | 3.4360 | 0.9271 | 0.9996 | |
| AAEL022574 | NA | -0.0432 | 2.9078 | 0.9273 | 0.9996 | NA |
| AAEL001845 | | 0.0296 | 6.3421 | 0.9274 | 0.9996 | short-chain dehydrogenase [Source:VB Community Annotation] |
| AAEL027228 | NA | -0.1178 | -0.0506 | 0.9275 | 0.9996 | NA |
| AAEL010134 | | -0.1729 | 2.4921 | 0.9276 | 0.9996 | pupal cuticle protein, putative [Source:VB Community Annotation] |
| AAEL024448 | NA | -0.0519 | 1.0257 | 0.9276 | 0.9996 | NA |
| AAEL020167 | NA | -0.1263 | -1.8669 | 0.9278 | 0.9996 | NA |
| AAEL008887 | | -0.0337 | 10.8658 | 0.9278 | 0.9996 | cytochrome c oxidase,-subunit VIb [Source:VB Community Annotation] |
| AAEL001865 | | -0.0382 | 8.9092 | 0.9279 | 0.9996 | |
| AAEL008619 | | 0.1586 | 8.7928 | 0.9279 | 0.9996 | |
| AAEL012582 | | 0.0377 | 4.5503 | 0.9280 | 0.9996 | RrnaAD, ribosomal RNA adenine dimethylase, putative [Source:VB Community Annotation] |
| AAEL001371 | | -0.0280 | 4.6380 | 0.9280 | 0.9996 | pre-mRNA cleavage factor im, 25kD subunit [Source:VB Community Annotation] |
| AAEL003857 | DEFD | -0.1453 | 7.3132 | 0.9281 | 0.9996 | defensin anti-microbial peptide [Source:VB Community Annotation] |
| AAEL012819 | | 0.0358 | 6.4815 | 0.9281 | 0.9996 | vacuolar ATP synthase subunit g [Source:VB Community Annotation] |
| AAEL010164 | | -0.1947 | 0.6338 | 0.9281 | 0.9996 | |
| AAEL011022 | | 0.0336 | 5.6610 | 0.9282 | 0.9996 | cysteine-rich protein, putative [Source:VB Community Annotation] |
| AAEL004982 | | 0.1602 | 1.7464 | 0.9282 | 0.9996 | Anoctamin [Source:UniProtKB/TrEMBL;Acc:Q17BF8] |
| AAEL002185 | | 0.1210 | 10.1703 | 0.9284 | 0.9996 | cuticle protein, putative [Source:VB Community Annotation] |
| AAEL006476 | | -0.0748 | 3.1537 | 0.9285 | 0.9996 | symbol, putative [Source:VB Community Annotation] |
| AAEL006004 | | -0.0226 | 5.1481 | 0.9285 | 0.9996 | |
| AAEL011588 | | -0.1386 | 0.7745 | 0.9286 | 0.9996 | |
| AAEL006161 | CLIPB31 | 0.0421 | 3.1275 | 0.9287 | 0.9996 | Clip-Domain Serine Protease  family B [Source:VB Community Annotation] |

| | | | | | | |
|---|---|---|---|---|---|---|
| AAEL002347 | | 0.1192 | 0.1801 | 0.9288 | 0.9996 | serine-type enodpeptidase, [Source:VB Community Annotation] |
| AAEL004341 | CCEAE1O | -0.1619 | -0.3544 | 0.9289 | 0.9996 | Carboxy/choline esterase Alpha Esterase [Source:VB Community Annotation] |
| AAEL001837 | | -0.1138 | 0.9713 | 0.9289 | 0.9996 | lipase [Source:VB Community Annotation] |
| AAEL026782 | NA | 0.0432 | 3.3219 | 0.9291 | 0.9996 | NA |
| AAEL002731 | SRPN14 | -0.0428 | 2.7302 | 0.9292 | 0.9996 | Serine Protease Inhibitor (serpin) homologue - unlikely to be inhibitory. [Source:VB Community Annotation] |
| AAEL004991 | | -0.1540 | 1.7334 | 0.9293 | 0.9996 | protein 4.1 (band 4.1) (EPB4.1) [Source:VB Community Annotation] |
| AAEL001699 | | 0.0344 | 4.1177 | 0.9293 | 0.9996 | adenylate cyclase [Source:VB Community Annotation] |
| AAEL001178 | | -0.0902 | 0.1837 | 0.9294 | 0.9996 | serine protease [Source:VB Community Annotation] |
| AAEL012128 | | -0.0442 | 4.7130 | 0.9294 | 0.9996 | cationic amino acid transporter [Source:VB Community Annotation] |
| AAEL013549 | | -0.0360 | 2.4011 | 0.9294 | 0.9996 | |
| AAEL021950 | NA | 0.0273 | 4.3703 | 0.9294 | 0.9996 | NA |
| AAEL022307 | NA | 0.0997 | 1.4277 | 0.9295 | 0.9996 | NA |
| AAEL005518 | | 0.0683 | 0.9413 | 0.9295 | 0.9996 | |
| AAEL004777 | GPRGRK | -0.1273 | 0.2368 | 0.9295 | 0.9996 | Glycoprotein Hormone Family [Source:VB Community Annotation] |
| AAEL022906 | NA | -0.1521 | -2.7628 | 0.9296 | 0.9996 | NA |
| AAEL000229 | | -0.1726 | 9.3393 | 0.9297 | 0.9996 | prosialokinin precursor [Source:VB Community Annotation] |
| AAEL005848 | | -0.0265 | 3.7998 | 0.9297 | 0.9996 | |
| AAEL008536 | | -0.0344 | 3.0945 | 0.9298 | 0.9996 | UCR-motif DNA binding protein, putative [Source:VB Community Annotation] |
| AAEL013018 | OBP56 | 0.0418 | 9.1889 | 0.9298 | 0.9996 | odorant binding protein OBP56 [Source:VB Community Annotation] |
| AAEL021756 | NA | -0.0396 | 4.3608 | 0.9299 | 0.9996 | NA |
| AAEL017282 | | 0.0327 | 1.8034 | 0.9299 | 0.9996 | |
| AAEL011343 | | -0.1179 | -0.6175 | 0.9302 | 0.9996 | |
| AAEL010663 | | 0.0305 | 4.0545 | 0.9302 | 0.9996 | cleavage and polyadenylation specificity factor [Source:VB Community Annotation] |
| AAEL020311 | NA | 0.0463 | 4.2257 | 0.9304 | 0.9996 | NA |
| AAEL025444 | NA | 0.0439 | 6.3985 | 0.9304 | 0.9996 | NA |
| AAEL010505 | | 0.0534 | 6.5624 | 0.9305 | 0.9996 | ribulose-5-phosphate-3-epimerase [Source:VB Community Annotation] |
| AAEL002814 | | 0.0509 | 4.7776 | 0.9306 | 0.9996 | |
| AAEL022394 | NA | 0.0757 | 2.1423 | 0.9306 | 0.9996 | NA |
| AAEL011756 | | -0.1055 | 0.2623 | 0.9307 | 0.9996 | aldehyde dehydrogenase [Source:VB Community Annotation] |
| AAEL007568 | | -0.0303 | 4.2615 | 0.9307 | 0.9996 | DNA-directed RNA polymerase [Source:VB Community Annotation] |
| AAEL012455 | | 0.0814 | 2.0503 | 0.9307 | 0.9996 | alcohol dehydrogenase [Source:VB Community Annotation] |
| AAEL009755 | | 0.0733 | 5.0671 | 0.9308 | 0.9996 | |
| AAEL007701 | | -0.0311 | 3.6008 | 0.9308 | 0.9996 | |
| AAEL005208 | | 0.0199 | 4.8020 | 0.9309 | 0.9996 | |
| AAEL027936 | NA | 0.1454 | 10.4629 | 0.9310 | 0.9996 | NA |
| AAEL001559 | | -0.1096 | -2.5994 | 0.9311 | 0.9996 | |
| AAEL016840 | tRNA-Leu | -0.0574 | 1.7322 | 0.9313 | 0.9996 | |
| AAEL000709 | cact | 0.0433 | 6.0909 | 0.9314 | 0.9996 | protein cactus (TOLL pathway signalling) [Source:VB Community Annotation] |
| AAEL007707 | | 0.0363 | 7.4034 | 0.9314 | 0.9996 | Malate dehydrogenase [Source:UniProtKB/TrEMBL;Acc:Q171B2] |
| AAEL007706 | | 0.1068 | -0.0342 | 0.9314 | 0.9996 | |

| | | | | | | |
|---|---|---|---|---|---|---|
| AAEL012014 | | -0.0380 | 2.2724 | 0.9315 | 0.9996 | L-lactate dehydrogenase [Source:UniProtKB/TrEMBL;Acc:Q16ND1] |
| AAEL006507 | | -0.0328 | 3.4853 | 0.9315 | 0.9996 | |
| AAEL008643 | | -0.0304 | 3.4710 | 0.9315 | 0.9996 | RNA lariat debranching enzyme, putative [Source:VB Community Annotation] |
| AAEL004071 | | -0.0272 | 9.2944 | 0.9319 | 0.9996 | nitrogen fixation protein nifu [Source:VB Community Annotation] |
| AAEL028846 | NA | -0.0600 | 1.7439 | 0.9319 | 0.9996 | NA |
| AAEL012751 | | 0.0402 | 6.9893 | 0.9320 | 0.9996 | |
| AAEL024249 | NA | -0.0882 | -1.2847 | 0.9321 | 0.9996 | NA |
| AAEL023534 | NA | -0.0412 | 3.1461 | 0.9321 | 0.9996 | NA |
| AAEL000394 | | -0.0270 | 5.2606 | 0.9321 | 0.9996 | U2 small nuclear ribonucleoprotein, putative [Source:VB Community Annotation] |
| AAEL010755 | | 0.0287 | 5.5254 | 0.9323 | 0.9996 | |
| AAEL003906 | | 0.0490 | 2.1949 | 0.9323 | 0.9996 | Endonuclease III homolog [Source:UniProtKB/TrEMBL;Acc:Q17E62] |
| AAEL001807 | CYP9M9 | -0.1288 | -1.7440 | 0.9324 | 0.9996 | cytochrome P450 [Source:VB Community Annotation] |
| AAEL007432 | | -0.1521 | 0.1235 | 0.9326 | 0.9996 | serine collagenase 1 precursor, putative [Source:VB Community Annotation] |
| AAEL022806 | NA | -0.0291 | 4.9277 | 0.9326 | 0.9996 | NA |
| AAEL017297 | CYP6M9 | 0.0377 | 3.7333 | 0.9328 | 0.9996 | cytochrome P450 [Source:VB Community Annotation] |
| AAEL003129 | | 0.0409 | 3.2581 | 0.9328 | 0.9996 | neuroligin, [Source:VB Community Annotation] |
| AAEL004843 | | -0.1079 | -0.8766 | 0.9329 | 0.9996 | actin [Source:VB Community Annotation] |
| AAEL010608 | | 0.0305 | 7.8986 | 0.9330 | 0.9996 | Succinate dehydrogenase (quinone) (Fragment) [Source:UniProtKB/TrEMBL;Acc:Q16SE4] |
| AAEL008462 | | -0.0737 | 3.3362 | 0.9330 | 0.9996 | apl5 protein (spac144.06 protein) [Source:VB Community Annotation] |
| AAEL015124 | | 0.0482 | 2.2548 | 0.9331 | 0.9996 | |
| AAEL014021 | Med23 | -0.0440 | 2.4392 | 0.9331 | 0.9996 | mediator of RNA polymerase II transcription subunit 23 (Med23) [Source:VB Community Annotation] |
| AAEL007100 | | 0.0257 | 5.2999 | 0.9334 | 0.9996 | |
| AAEL023306 | NA | -0.0355 | 4.1179 | 0.9337 | 0.9996 | NA |
| AAEL002997 | CLIPD3 | 0.1019 | -1.1676 | 0.9337 | 0.9996 | Clip-Domain Serine Protease family D. [Source:VB Community Annotation] |
| AAEL003443 | | -0.0688 | 3.9589 | 0.9338 | 0.9996 | threonine dehydrogenase [Source:VB Community Annotation] |
| AAEL000208 | | 0.0272 | 7.9596 | 0.9339 | 0.9996 | copii-coated vesicle membrane protein P24 [Source:VB Community Annotation |
| AAEL003494 | | -0.0257 | 4.7366 | 0.9340 | 0.9996 | goodpasture antigen-binding protein [Source:VB Community Annotation] |
| AAEL006394 | | -0.1754 | 1.6360 | 0.9340 | 0.9996 | |
| AAEL014704 | | -0.0258 | 4.5428 | 0.9340 | 0.9996 | U3 small nucleolar RNA (U3 snoRNA) associated protein [Source:VB Community Annotation] |
| AAEL020830 | NA | 0.0918 | 4.2514 | 0.9341 | 0.9996 | NA |
| AAEL020368 | NA | 0.0730 | -1.0379 | 0.9341 | 0.9996 | NA |
| AAEL008733 | | 0.0206 | 5.1462 | 0.9342 | 0.9996 | histidine triad (hit) protein member [Source:VB Community Annotation] |
| AAEL001176 | | 0.1512 | 1.3900 | 0.9342 | 0.9996 | S-adenosylmethionine decarboxylase proenzyme [Source:UniProtKB/TrEMBL;Acc:Q17M10] |
| AAEL022379 | NA | 0.1110 | 1.5709 | 0.9345 | 0.9996 | NA |
| AAEL015644 | | -0.0852 | 1.2539 | 0.9347 | 0.9996 | |
| AAEL003923 | | -0.0237 | 3.9936 | 0.9347 | 0.9996 | |
| AAEL005699 | | 0.0344 | 8.2832 | 0.9348 | 0.9996 | membrane protein tms1d [Source:VB Community Annotation] |
| AAEL009178 | GNBPB4 | -0.0427 | 6.7341 | 0.9350 | 0.9996 | Gram-Negative Binding Protein (GNBP) or Beta-1 3-Glucan Binding Protein (BGBP). [Source:VB Community Annotation] |
| AAEL009327 | | -0.1227 | 3.0329 | 0.9350 | 0.9996 | |
| AAEL002892 | | -0.0498 | 2.0511 | 0.9351 | 0.9996 | protein kinase c, mu [Source:VB Community Annotation] |

| | | | | | | |
|---|---|---|---|---|---|---|
| AAEL007502 | | 0.0799 | 3.3170 | 0.9351 | 0.9996 | iroquois-class homeodomain protein irx [Source:VB Community Annotation] |
| AAEL029005 | NA | 0.1777 | 1.4513 | 0.9351 | 0.9996 | NA |
| AAEL017224 | | -0.0548 | 4.7731 | 0.9352 | 0.9996 | |
| AAEL025295 | NA | -0.0862 | 2.9700 | 0.9353 | 0.9996 | NA |
| AAEL021257 | NA | 0.0712 | 1.7959 | 0.9353 | 0.9996 | NA |
| AAEL017054 | | -0.0547 | 4.9566 | 0.9356 | 0.9996 | |
| AAEL005041 | | 0.0336 | 3.5374 | 0.9356 | 0.9996 | Ribosome biogenesis protein WDR12 homolog [Source:UniProtKB/Swiss-Prot;Acc:Q17BB0] |
| AAEL007839 | | 0.0539 | 4.6779 | 0.9356 | 0.9996 | Beta-hexosaminidase [Source:UniProtKB/TrEMBL;Acc:Q170Q1] |
| AAEL025133 | NA | 0.0870 | 1.7711 | 0.9357 | 0.9996 | NA |
| AAEL014318 | | 0.1686 | 1.5703 | 0.9357 | 0.9996 | |
| AAEL009018 | | -0.0525 | 8.4433 | 0.9357 | 0.9996 | cytochrome P450 [Source:VB Community Annotation] |
| AAEL002436 | | 0.0358 | 8.2081 | 0.9357 | 0.9996 | |
| AAEL003695 | | 0.0250 | 5.3958 | 0.9358 | 0.9996 | |
| AAEL021171 | NA | -0.0183 | 5.5030 | 0.9361 | 0.9996 | NA |
| AAEL011253 | | -0.1160 | 3.7104 | 0.9362 | 0.9996 | rho-GTPase-activating protein [Source:VB Community Annotation] |
| AAEL007601 | | -0.1288 | -2.2797 | 0.9362 | 0.9996 | trypsin [Source:VB Community Annotation] |
| AAEL023414 | NA | -0.0435 | 2.4877 | 0.9362 | 0.9996 | NA |
| AAEL004511 | | 0.0258 | 5.3063 | 0.9363 | 0.9996 | |
| AAEL010968 | | -0.0302 | 3.0392 | 0.9363 | 0.9996 | Queuine tRNA-ribosyltransferase subunit QTRTD1 homolog (EC 2.4.2.29)(Queuine tRNA-ribosyltransferase domain-containing protein 1) |
| AAEL027933 | NA | -0.0309 | 2.4137 | 0.9364 | 0.9996 | NA |
| AAEL002075 | | 0.2283 | 2.0534 | 0.9364 | 0.9996 | |
| AAEL003746 | | -0.0244 | 7.1550 | 0.9365 | 0.9996 | 4-Hydroxybutyrate CoA-transferase, putative [Source:VB Community Annotation] |
| AAEL019406 | NA | -0.0574 | 4.1440 | 0.9366 | 0.9996 | NA |
| AAEL008990 | | 0.0843 | 3.5239 | 0.9366 | 0.9996 | |
| AAEL023382 | NA | 0.0421 | 4.9230 | 0.9367 | 0.9996 | NA |
| AAEL011606 | | 0.0412 | 3.8987 | 0.9367 | 0.9996 | |
| AAEL011817 | | 0.0308 | 5.4998 | 0.9369 | 0.9996 | nonsense-mediated mRNA decay protein 1 (rent1) [Source:VB Community Annotation] |
| AAEL026432 | NA | -0.0749 | 4.0278 | 0.9370 | 0.9996 | NA |
| AAEL008545 | | 0.0554 | 1.6568 | 0.9370 | 0.9996 | |
| AAEL007211 | | -0.0701 | 0.3046 | 0.9370 | 0.9996 | |
| AAEL027899 | NA | -0.0472 | 0.2037 | 0.9371 | 0.9996 | NA |
| AAEL003171 | | 0.0295 | 2.7906 | 0.9373 | 0.9996 | |
| AAEL004035 | | -0.1014 | 0.8199 | 0.9374 | 0.9996 | importin 11 (imp11) (ran-binding protein 11) [Source:VB Community Annotation] |
| AAEL003069 | | -0.0533 | 0.7372 | 0.9375 | 0.9996 | |
| AAEL003701 | | 0.0972 | 1.5772 | 0.9377 | 0.9996 | actin-binding protein ipp [Source:VB Community Annotation] |
| AAEL005054 | | -0.0209 | 4.8663 | 0.9378 | 0.9996 | pap-inositol-1,4-phosphatase [Source:VB Community Annotation] |
| AAEL000924 | | 0.0312 | 2.5127 | 0.9378 | 0.9996 | eukaryotic translation initiation factor 2-alpha kinase [Source:VB Community Annotation] |
| AAEL000422 | | -0.0365 | 3.2915 | 0.9378 | 0.9996 | U3 small nucleolar RNA-associated protein homolog, putative [Source:VB Community Annotation] |
| AAEL003140 | | -0.0309 | 3.2494 | 0.9379 | 0.9996 | retinoblastoma binding protein [Source:VB Community Annotation] |
| AAEL007889 | | 0.0348 | 7.6398 | 0.9379 | 0.9996 | F-spondin [Source:VB Community Annotation] |

| | | | | | | |
|---|---|---|---|---|---|---|
| AAEL007684 | | -0.0404 | 1.6658 | 0.9380 | 0.9996 | tektin, putative [Source:VB Community Annotation] |
| AAEL005342 | | -0.0339 | 5.3216 | 0.9380 | 0.9996 | |
| AAEL007815 | CYP4D24 | -0.0497 | 3.6203 | 0.9382 | 0.9996 | cytochrome P450 [Source:VB Community Annotation] |
| AAEL025245 | NA | -0.1356 | 3.6051 | 0.9384 | 0.9996 | NA |
| AAEL020809 | NA | -0.0383 | 2.3178 | 0.9384 | 0.9996 | NA |
| AAEL010444 | | 0.0911 | 3.3103 | 0.9384 | 0.9996 | |
| AAEL005826 | | 0.0558 | 3.9884 | 0.9385 | 0.9996 | E3 ubiquitin-protein ligase [Source:UniProtKB/TrEMBL;Acc:Q178N6] |
| AAEL022936 | NA | -0.0226 | 3.4881 | 0.9385 | 0.9996 | NA |
| AAEL023350 | NA | -0.1459 | 0.8749 | 0.9386 | 0.9996 | NA |
| AAEL021748 | NA | -0.1280 | 3.1301 | 0.9386 | 0.9996 | NA |
| AAEL004353 | | 0.1739 | 6.5895 | 0.9387 | 0.9996 | |
| AAEL022996 | NA | 0.0388 | 3.7928 | 0.9389 | 0.9996 | NA |
| AAEL008517 | | 0.0276 | 6.9656 | 0.9389 | 0.9996 | elongation factor tu (ef-tu) [Source:VB Community Annotation] |
| AAEL020736 | NA | 0.0494 | 0.1388 | 0.9390 | 0.9996 | NA |
| AAEL010148 | | -0.0337 | 5.9873 | 0.9390 | 0.9996 | sodium/potassium-dependent ATPase beta-2 subunit [Source:VB Community Annotation] |
| AAEL021824 | NA | -0.1032 | -1.4828 | 0.9390 | 0.9996 | NA |
| AAEL001038 | | -0.0301 | 3.4632 | 0.9391 | 0.9996 | cak1 [Source:VB Community Annotation] |
| AAEL027514 | NA | 0.1165 | 1.7288 | 0.9391 | 0.9996 | NA |
| AAEL001934 | | 0.0374 | 3.5592 | 0.9394 | 0.9996 | |
| AAEL005573 | | 0.0268 | 4.5713 | 0.9394 | 0.9996 | GTP-binding protein era [Source:VB Community Annotation] |
| AAEL007394 | | -0.1363 | 5.4920 | 0.9394 | 0.9996 | |
| AAEL013341 | | 0.0293 | 7.1983 | 0.9394 | 0.9996 | lethal(2)essential for life protein, l2efl [Source:VB Community Annotation] |
| AAEL016392 | tRNA-Glu | 0.0686 | 1.4499 | 0.9395 | 0.9996 | |
| AAEL001076 | | 0.1180 | 3.0901 | 0.9395 | 0.9996 | lipase [Source:VB Community Annotation] |
| AAEL002860 | | 0.0350 | 7.2058 | 0.9398 | 0.9996 | Mitochondrial pyruvate carrier [Source:UniProtKB/TrEMBL;Acc:Q17GV8] |
| AAEL026903 | NA | -0.1415 | 0.4738 | 0.9399 | 0.9996 | NA |
| AAEL018205 | | -0.0231 | 7.2152 | 0.9400 | 0.9996 | |
| AAEL025574 | NA | -0.0504 | 5.2608 | 0.9401 | 0.9996 | NA |
| AAEL000434 | | -0.0248 | 4.8196 | 0.9401 | 0.9996 | lipid a export ATP-binding/permease protein msba [Source:VB Community Annotation] |
| AAEL024661 | NA | 0.0874 | -1.0079 | 0.9402 | 0.9996 | NA |
| AAEL012949 | | 0.0343 | 3.8610 | 0.9403 | 0.9996 | transferrin [Source:VB Community Annotation] |
| AAEL021735 | NA | -0.1521 | -2.3242 | 0.9405 | 0.9996 | NA |
| AAEL001227 | | 0.1139 | -1.4222 | 0.9407 | 0.9996 | sidestep protein [Source:VB Community Annotation] |
| AAEL003631 | CLIPB41 | -0.1038 | -0.7639 | 0.9407 | 0.9996 | Clip-Domain Serine Protease family B. [Source:VB Community Annotation] |
| AAEL010953 | | -0.0201 | 5.4066 | 0.9409 | 0.9996 | Protein OPI10 homolog [Source:UniProtKB/Swiss-Prot;Acc:Q16RI1] |
| AAEL015424 | | -0.0483 | 6.6782 | 0.9409 | 0.9996 | adult cuticle protein, putative [Source:VB Community Annotation] |
| AAEL003735 | | 0.0315 | 4.8606 | 0.9409 | 0.9996 | |
| AAEL001392 | | 0.0881 | 3.8585 | 0.9409 | 0.9996 | |
| AAEL018055 | | -0.0455 | 1.9686 | 0.9410 | 0.9996 | |
| AAEL005688 | | -0.0270 | 3.6327 | 0.9410 | 0.9996 | glutamyl-tRNA synthetase [Source:VB Community Annotation] |

| | | | | | | |
|---|---|---|---|---|---|---|
| AAEL005412 | | -0.0455 | 1.8513 | 0.9411 | 0.9996 | annexin x [Source:VB Community Annotation] |
| AAEL005890 | | 0.0267 | 3.7817 | 0.9411 | 0.9996 | |
| AAEL007956 | | -0.0383 | 4.0257 | 0.9411 | 0.9996 | |
| AAEL008080 | | 0.1134 | 1.8287 | 0.9412 | 0.9996 | trypsin-eta, putative [Source:VB Community Annotation] |
| AAEL006328 | | 0.0613 | 2.5300 | 0.9412 | 0.9996 | |
| AAEL014365 | | 0.0296 | 4.2734 | 0.9412 | 0.9996 | chromaffin granule amine transporter, putative [Source:VB Community Annotation] |
| AAEL028895 | NA | 0.2105 | 0.6975 | 0.9412 | 0.9996 | NA |
| AAEL005406 | | 0.0207 | 3.4665 | 0.9412 | 0.9996 | |
| AAEL004733 | | 0.0241 | 5.8760 | 0.9413 | 0.9996 | jun activation domain binding protein [Source:VB Community Annotation] |
| AAEL007767 | | -0.0249 | 4.5264 | 0.9414 | 0.9996 | Protein kintoun [Source:UniProtKB/Swiss-Prot;Acc:Q0IEW8] |
| AAEL021432 | NA | 0.0755 | 4.2533 | 0.9417 | 0.9996 | NA |
| AAEL019776 | NA | -0.0342 | 5.8449 | 0.9417 | 0.9996 | NA |
| AAEL002585 | | -0.0432 | 4.0498 | 0.9418 | 0.9996 | serine protease [Source:VB Community Annotation] |
| AAEL023756 | NA | -0.0227 | 5.9298 | 0.9418 | 0.9996 | NA |
| AAEL006143 | | 0.0320 | 7.1777 | 0.9421 | 0.9996 | |
| AAEL005086 | | 0.0200 | 3.4405 | 0.9421 | 0.9996 | |
| AAEL023884 | NA | -0.0495 | 0.8460 | 0.9421 | 0.9996 | NA |
| AAEL019819 | NA | 0.0485 | 3.9085 | 0.9421 | 0.9996 | NA |
| AAEL004820 | | -0.0218 | 3.3288 | 0.9421 | 0.9996 | zinc finger protein [Source:VB Community Annotation] |
| AAEL009994 | RpL4 | -0.0236 | 12.0436 | 0.9422 | 0.9996 | 60S ribosomal protein L4 [Source:VB Community Annotation] |
| AAEL006361 | SCRC2 | -0.0585 | 2.0257 | 0.9423 | 0.9996 | Class C Scavenger Receptor (Sushi/SCR/CCP MAM and Somatomedin B domains). [Source:VB Community Annotation] |
| AAEL007251 | | -0.0229 | 3.1141 | 0.9423 | 0.9996 | zinc finger protein, putative [Source:VB Community Annotation] |
| AAEL002656 | | -0.1212 | 6.0009 | 0.9423 | 0.9996 | |
| AAEL006953 | | -0.3216 | 3.1485 | 0.9424 | 0.9996 | |
| AAEL023521 | NA | -0.0458 | 4.9793 | 0.9425 | 0.9996 | NA |
| AAEL019519 | NA | 0.1342 | 0.0397 | 0.9426 | 0.9996 | NA |
| AAEL001068 | | -0.0590 | 1.7876 | 0.9427 | 0.9996 | myosin light chain 2V, putative [Source:VB Community Annotation] |
| AAEL013140 | | 0.0438 | 3.4569 | 0.9427 | 0.9996 | |
| AAEL004585 | | 0.1244 | 10.5926 | 0.9429 | 0.9996 | |
| AAEL021419 | NA | 0.1388 | 0.0330 | 0.9429 | 0.9996 | NA |
| AAEL014000 | | 0.0263 | 2.2603 | 0.9430 | 0.9996 | |
| AAEL010176 | | 0.0269 | 2.7482 | 0.9430 | 0.9996 | |
| AAEL003332 | | 0.0223 | 3.4902 | 0.9431 | 0.9996 | |
| AAEL013109 | | -0.0918 | 0.0815 | 0.9434 | 0.9996 | glutamate transporter [Source:VB Community Annotation] |
| AAEL027973 | NA | 0.0266 | 3.8767 | 0.9435 | 0.9996 | NA |
| AAEL000569 | | 0.0234 | 3.3421 | 0.9436 | 0.9996 | omega-crystallin, putative [Source:VB Community Annotation] |
| AAEL008260 | | 0.1463 | -0.4226 | 0.9436 | 0.9996 | |
| AAEL001210 | | -0.0294 | 8.7776 | 0.9437 | 0.9996 | NADH ubiquinone oxidoreductase subunit, putative [Source:VB Community Annotation] |
| AAEL027788 | NA | 0.0483 | 1.2883 | 0.9437 | 0.9996 | NA |
| AAEL011328 | | -0.0308 | 3.4710 | 0.9437 | 0.9996 | zinc finger protein [Source:VB Community Annotation] |

| | | | | | | |
|---|---|---|---|---|---|---|
| AAEL000269 | | -0.0260 | 8.6642 | 0.9437 | 0.9996 | |
| AAEL024660 | NA | -0.0728 | 3.1974 | 0.9439 | 0.9996 | NA |
| AAEL004746 | | 0.0214 | 3.4002 | 0.9441 | 0.9996 | structural contituent of cuticle [Source:VB Community Annotation] |
| AAEL006871 | | 0.0255 | 2.8867 | 0.9441 | 0.9996 | |
| AAEL006174 | | -0.0186 | 6.5243 | 0.9442 | 0.9996 | Proteasome subunit beta type [Source:UniProtKB/TrEMBL;Acc:Q177C2] |
| AAEL013114 | | 0.0248 | 3.8255 | 0.9444 | 0.9996 | DNA-J, putative [Source:VB Community Annotation] |
| AAEL013258 | | 0.0907 | -0.3760 | 0.9444 | 0.9996 | |
| AAEL012613 | | -0.0354 | 4.2206 | 0.9444 | 0.9996 | |
| AAEL009944 | | -0.0266 | 3.6283 | 0.9444 | 0.9996 | |
| AAEL004981 | | -0.0166 | 6.1553 | 0.9445 | 0.9996 | cation-transporting ATPase [Source:VB Community Annotation] |
| AAEL009601 | | -0.0441 | 4.9052 | 0.9447 | 0.9996 | pyridoxine kinase [Source:VB Community Annotation] |
| AAEL005960 | | 0.0988 | 0.3036 | 0.9448 | 0.9996 | |
| AAEL008347 | | 0.0241 | 5.3240 | 0.9449 | 0.9996 | monocarboxylate transporter [Source:VB Community Annotation] |
| AAEL003343 | | 0.1191 | -0.8953 | 0.9451 | 0.9996 | |
| AAEL022506 | NA | 0.0717 | 1.5367 | 0.9451 | 0.9996 | NA |
| AAEL000201 | | 0.0524 | 0.7442 | 0.9452 | 0.9996 | DNA replication helicase dna2 [Source:VB Community Annotation] |
| AAEL014636 | | -0.0279 | 6.2898 | 0.9452 | 0.9996 | rhoGTPase [Source:VB Community Annotation] |
| AAEL011056 | | 0.1275 | 0.1547 | 0.9452 | 0.9996 | transcription factor HES-1 (Hairy and enhancer of split 1), putative [Source:VB Community Annotation] |
| AAEL021945 | NA | 0.0603 | 0.0428 | 0.9453 | 0.9996 | NA |
| AAEL011349 | | -0.0318 | 4.4223 | 0.9454 | 0.9996 | serine protease [Source:VB Community Annotation] |
| AAEL003451 | | -0.0345 | 1.6604 | 0.9454 | 0.9996 | |
| AAEL019543 | NA | -0.0504 | 4.7850 | 0.9457 | 0.9996 | NA |
| AAEL019651 | NA | 0.0239 | 3.2632 | 0.9457 | 0.9996 | NA |
| AAEL014053 | | -0.0466 | 3.3008 | 0.9457 | 0.9996 | vacuolar proton ATPases [Source:VB Community Annotation] |
| AAEL000097 | | 0.0421 | 1.4653 | 0.9458 | 0.9996 | n-acetylneuraminate synthetase [Source:VB Community Annotation] |
| AAEL018102 | | 0.0447 | 8.6284 | 0.9461 | 0.9996 | |
| AAEL017403 | TMOF | -0.1521 | -2.4275 | 0.9461 | 0.9996 | Trypsin Modulating Oostatic Factor [Source:VB Community Annotation] |
| AAEL011563 | | -0.0924 | 0.4030 | 0.9462 | 0.9996 | |
| AAEL007354 | | -0.0199 | 4.8183 | 0.9462 | 0.9996 | pseudouridylate synthase [Source:VB Community Annotation] |
| AAEL007353 | | -0.0175 | 4.2911 | 0.9464 | 0.9996 | cullin [Source:VB Community Annotation] |
| AAEL007370 | | 0.0293 | 4.2379 | 0.9464 | 0.9996 | |
| AAEL008078 | | 0.0846 | 4.2620 | 0.9466 | 0.9996 | clk2 [Source:VB Community Annotation] |
| AAEL024460 | NA | 0.0531 | 4.8128 | 0.9467 | 0.9996 | NA |
| AAEL007876 | | 0.1046 | -0.2783 | 0.9468 | 0.9996 | tyrosine protein kinase with ig domain [Source:VB Community Annotation] |
| AAEL000857 | | -0.0282 | 4.0862 | 0.9468 | 0.9996 | |
| AAEL004142 | psidin | 0.0213 | 2.5903 | 0.9469 | 0.9996 | Phagocyte signaling-impaired protein (N-terminal acetyltransferase B complex subunit NAA25 homolog)(N-terminal acetyltransferase B c |
| AAEL019920 | NA | -0.1521 | -1.4066 | 0.9469 | 0.9996 | NA |
| AAEL020020 | NA | -0.0546 | -0.1206 | 0.9469 | 0.9996 | NA |
| AAEL001642 | | 0.0240 | 5.2694 | 0.9470 | 0.9996 | 6-pyruvoyltetrahydropterin synthase, putative [Source:VB Community Annotation] |
| AAEL022876 | NA | 0.1140 | -0.2448 | 0.9471 | 0.9996 | NA |

| | | | | | | |
|---|---|---|---|---|---|---|
| AAEL002267 | | 0.0611 | 1.9635 | 0.9473 | 0.9996 | |
| AAEL002253 | | 0.0181 | 3.6003 | 0.9474 | 0.9996 | beta-1,3-n-acetylglucosaminyltransferase radical fringe (o-fucosylpeptide 3-beta-n-acetylglucosaminyltransferase) [Source:VB Communit |
| AAEL001500 | | -0.0403 | 2.1682 | 0.9474 | 0.9996 | insulinprotein enhancer protein isl [Source:VB Community Annotation] |
| AAEL018214 | | 0.0524 | 5.4316 | 0.9474 | 0.9996 | |
| AAEL013593 | | -0.0253 | 3.2120 | 0.9474 | 0.9996 | |
| AAEL021217 | NA | 0.0957 | -1.5770 | 0.9475 | 0.9996 | NA |
| AAEL000165 | | 0.0631 | 6.8445 | 0.9475 | 0.9996 | |
| AAEL027975 | NA | -0.1870 | 1.9516 | 0.9476 | 0.9996 | NA |
| AAEL019714 | NA | -0.0993 | 1.9244 | 0.9477 | 0.9996 | NA |
| AAEL011903 | | -0.0319 | 1.9034 | 0.9477 | 0.9996 | zinc finger protein [Source:VB Community Annotation] |
| AAEL007712 | Tsc2 | -0.0180 | 5.9455 | 0.9477 | 0.9996 | tuberous sclerosis 2 [Source:VB Community Annotation] |
| AAEL006401 | | -0.0287 | 3.5073 | 0.9477 | 0.9996 | mitosis protein dim1 [Source:VB Community Annotation] |
| AAEL001586 | | 0.0228 | 5.3102 | 0.9478 | 0.9996 | glucosyl/glucuronosyl transferases [Source:VB Community Annotation] |
| AAEL013542 | | -0.1521 | -1.4755 | 0.9480 | 0.9996 | elongase, putative [Source:VB Community Annotation] |
| AAEL000758 | | 0.0211 | 6.1057 | 0.9480 | 0.9996 | ubiquitin-activating enzyme E1 [Source:VB Community Annotation] |
| AAEL011386 | | 0.0280 | 3.2549 | 0.9481 | 0.9996 | |
| AAEL002779 | | -0.0356 | 2.9182 | 0.9482 | 0.9996 | ribokinase [Source:VB Community Annotation] |
| AAEL005520 | | 0.0303 | 6.2874 | 0.9482 | 0.9996 | carbonic anhydrase [Source:VB Community Annotation] |
| AAEL004180 | | -0.0207 | 4.3015 | 0.9482 | 0.9996 | Ubiquinone biosynthesis monooxygenase COQ6, mitochondrial [Source:UniProtKB/TrEMBL;Acc:Q0IFQ5] |
| AAEL009778 | | 0.0349 | 1.3917 | 0.9482 | 0.9996 | |
| AAEL010311 | | -0.0368 | 2.3859 | 0.9483 | 0.9996 | |
| AAEL018182 | | 0.0219 | 5.7682 | 0.9484 | 0.9996 | |
| AAEL011622 | | -0.0435 | 5.5500 | 0.9486 | 0.9996 | serine protease, putative [Source:VB Community Annotation] |
| AAEL003649 | | 0.0224 | 4.5363 | 0.9486 | 0.9996 | |
| AAEL027977 | NA | 0.0191 | 7.8859 | 0.9487 | 0.9996 | NA |
| AAEL003452 | | 0.0724 | 0.0643 | 0.9487 | 0.9996 | |
| AAEL014519 | | 0.0460 | 2.4290 | 0.9487 | 0.9996 | cbl-d [Source:VB Community Annotation] |
| AAEL008921 | | -0.0167 | 6.5646 | 0.9490 | 0.9996 | myosin regulatory light chain 2 smooth muscle [Source:VB Community Annotation] |
| AAEL010506 | | 0.0413 | 9.5911 | 0.9491 | 0.9996 | GTP-binding  protein alpha subunit, gna [Source:VB Community Annotation] |
| AAEL000071 | OBP2 | -0.0281 | 5.3748 | 0.9491 | 0.9996 | odorant binding protein OBP2 [Source:VB Community Annotation] |
| AAEL011858 | | -0.0265 | 1.9077 | 0.9492 | 0.9996 | transcription factor IIIA, putative [Source:VB Community Annotation] |
| AAEL021195 | NA | 0.0473 | 1.9518 | 0.9492 | 0.9996 | NA |
| AAEL006715 | | -0.0385 | 2.2475 | 0.9494 | 0.9996 | DNA polymerase iota [Source:VB Community Annotation] |
| AAEL001600 | | -0.0252 | 2.2314 | 0.9495 | 0.9996 | |
| AAEL012307 | | 0.0244 | 4.7589 | 0.9495 | 0.9996 | |
| AAEL001822 | | -0.0918 | -1.0348 | 0.9497 | 0.9996 | glucosyl/glucuronosyl transferases [Source:VB Community Annotation] |
| AAEL006799 | | 0.0236 | 2.9523 | 0.9497 | 0.9996 | |
| AAEL010964 | | 0.0281 | 2.8322 | 0.9497 | 0.9996 | |
| AAEL004462 | | -0.0462 | 0.4008 | 0.9497 | 0.9996 | |
| AAEL003762 | | 0.0297 | 4.2400 | 0.9498 | 0.9996 | Protoporphyrinogen IX oxidase, putative [Source:VB Community Annotation] |

| | | | | | | |
|---|---|---|---|---|---|---|
| AAEL005245 | | -0.0577 | 0.7299 | 0.9498 | 0.9996 | DNA repair protein rad50 [Source:VB Community Annotation] |
| AAEL006454 | OBP1 | -0.0605 | 6.3222 | 0.9498 | 0.9996 | odorant binding protein (OBP1) [Source:VB Community Annotation] |
| AAEL009067 | | 0.0244 | 3.5469 | 0.9499 | 0.9996 | helicase [Source:VB Community Annotation] |
| AAEL004478 | | -0.0989 | -0.9394 | 0.9499 | 0.9996 | |
| AAEL007239 | | -0.0238 | 5.5345 | 0.9500 | 0.9996 | |
| AAEL011866 | | 0.0265 | 4.7779 | 0.9500 | 0.9996 | DNA-directed RNA polymerase III 25 kDa polypeptide [Source:VB Community Annotation] |
| AAEL024236 | NA | 0.0941 | 0.7051 | 0.9500 | 0.9996 | NA |
| AAEL006207 | | 0.0237 | 4.3271 | 0.9501 | 0.9996 | |
| AAEL000445 | | -0.0767 | 1.3864 | 0.9502 | 0.9996 | |
| AAEL025999 | NA | -0.0258 | 8.3147 | 0.9502 | 0.9996 | NA |
| AAEL006887 | | -0.0416 | 4.6574 | 0.9503 | 0.9996 | mRNA turnover protein 4 mrt4 [Source:VB Community Annotation] |
| AAEL003991 | | 0.0154 | 3.8506 | 0.9503 | 0.9996 | alcohol dehydrogenase [Source:VB Community Annotation] |
| AAEL013046 | | 0.0216 | 4.0681 | 0.9503 | 0.9996 | glucose-methanol-choline (gmc) oxidoreductase [Source:VB Community Annotation] |
| AAEL011898 | | 0.0196 | 6.0934 | 0.9504 | 0.9996 | 1-acyl-sn-glycerol-3-phosphate acyltransferase [Source:UniProtKB/TrEMBL;Acc:Q16NQ5] |
| AAEL010762 | Arp8 | 0.0330 | 1.7963 | 0.9504 | 0.9996 | Actin-related protein 8 [Source:UniProtKB/Swiss-Prot;Acc:Q0IEG8] |
| AAEL001655 | | 0.0208 | 3.6384 | 0.9506 | 0.9996 | structural maintenance of chromosomes 4 smc4 [Source:VB Community Annotation] |
| AAEL009206 | | 0.0207 | 5.4932 | 0.9507 | 0.9996 | organic cation transporter [Source:VB Community Annotation] |
| AAEL012371 | | 0.0672 | -0.6964 | 0.9507 | 0.9996 | |
| AAEL019704 | NA | -0.1085 | -0.2425 | 0.9507 | 0.9996 | NA |
| AAEL004818 | | -0.0243 | 3.9418 | 0.9508 | 0.9996 | |
| AAEL013236 | | 0.0122 | 6.7256 | 0.9508 | 0.9996 | proteasome subunit beta type 5,8 [Source:VB Community Annotation] |
| AAEL012681 | RpL24 | -0.0263 | 9.5964 | 0.9508 | 0.9996 | 60S ribosomal protein L24 [Source:VB Community Annotation] |
| AAEL026922 | NA | -0.0137 | 6.1869 | 0.9509 | 0.9996 | NA |
| AAEL001077 | CLIPB45 | 0.0412 | 6.7019 | 0.9509 | 0.9996 | Clip-Domain Serine Protease family B. Protease homologue. [Source:VB Community Annotation] |
| AAEL021996 | NA | 0.0248 | 4.7687 | 0.9509 | 0.9996 | NA |
| AAEL009244 | | 0.1065 | 3.5295 | 0.9511 | 0.9996 | serine-type enodpeptidase, [Source:VB Community Annotation] |
| AAEL006630 | | -0.0394 | 2.3248 | 0.9512 | 0.9996 | |
| AAEL010393 | | -0.0475 | 0.2191 | 0.9515 | 0.9996 | ferritin subunit, putative [Source:VB Community Annotation] |
| AAEL027939 | NA | 0.1092 | 1.5546 | 0.9515 | 0.9996 | NA |
| AAEL000762 | LRIM19 | 0.0242 | 4.5196 | 0.9516 | 0.9996 | leucine-rich immune protein (Coil-less) [Source:VB Community Annotation] |
| AAEL002271 | | 0.0218 | 5.5578 | 0.9516 | 0.9996 | 26S proteasome regulatory subunit S3 [Source:VB Community Annotation] |
| AAEL019661 | NA | -0.0379 | 0.7977 | 0.9516 | 0.9996 | NA |
| AAEL010276 | | -0.0343 | 6.1538 | 0.9517 | 0.9996 | Aminomethyltransferase [Source:UniProtKB/TrEMBL;Acc:Q16TD5] |
| AAEL019880 | NA | 0.0460 | 1.3289 | 0.9517 | 0.9996 | NA |
| AAEL001864 | | 0.0861 | 3.2481 | 0.9518 | 0.9996 | eukaryotic translation initiation factor 4E binding protein (4EBP) [Source:VB Community Annotation] |
| AAEL004899 | | -0.1087 | 5.3965 | 0.9522 | 0.9996 | |
| AAEL013631 | | 0.0169 | 4.7863 | 0.9524 | 0.9996 | mmr1/hsr1 GTP binding protein [Source:VB Community Annotation] |
| AAEL019546 | NA | -0.0212 | 6.7099 | 0.9524 | 0.9996 | NA |
| AAEL006843 | | 0.0184 | 3.2384 | 0.9524 | 0.9996 | |
| AAEL006663 | | 0.0240 | 5.3979 | 0.9524 | 0.9996 | |

| | | | | | | |
|---|---|---|---|---|---|---|
| AAEL023829 | NA | -0.0286 | 7.0692 | 0.9526 | 0.9996 | NA |
| AAEL014495 | | 0.0215 | 3.4216 | 0.9526 | 0.9996 | |
| AAEL017270 | | -0.0236 | 4.3795 | 0.9528 | 0.9996 | |
| AAEL019757 | NA | 0.0264 | 2.7565 | 0.9528 | 0.9996 | NA |
| AAEL026269 | NA | -0.1071 | 0.3512 | 0.9529 | 0.9996 | NA |
| AAEL006211 | | -0.0738 | -0.5582 | 0.9529 | 0.9996 | |
| AAEL027493 | NA | 0.0307 | 4.0796 | 0.9530 | 0.9996 | NA |
| AAEL027732 | NA | 0.1163 | -0.7855 | 0.9530 | 0.9996 | NA |
| AAEL010081 | | 0.0152 | 4.2599 | 0.9530 | 0.9996 | |
| AAEL014376 | | -0.0298 | 1.6362 | 0.9531 | 0.9996 | |
| AAEL011436 | | -0.0712 | 2.2463 | 0.9533 | 0.9996 | myosin xv [Source:VB Community Annotation] |
| AAEL012764 | | 0.0602 | 6.2556 | 0.9534 | 0.9996 | |
| AAEL009640 | | -0.0395 | 2.2001 | 0.9534 | 0.9996 | |
| AAEL025438 | NA | -0.0182 | 3.4245 | 0.9537 | 0.9996 | NA |
| AAEL005531 | | 0.0309 | 5.5125 | 0.9538 | 0.9996 | venom allergen [Source:VB Community Annotation] |
| AAEL006539 | | -0.0237 | 4.2237 | 0.9538 | 0.9996 | serine/threonine protein kinase [Source:VB Community Annotation] |
| AAEL011369 | | -0.0736 | 2.6906 | 0.9538 | 0.9996 | endothelin-converting enzyme [Source:VB Community Annotation] |
| AAEL012975 | | -0.0319 | 2.3908 | 0.9540 | 0.9996 | |
| AAEL011693 | | 0.0217 | 3.0709 | 0.9541 | 0.9996 | mitotic control protein dis3 [Source:VB Community Annotation] |
| AAEL014379 | | -0.0197 | 6.5723 | 0.9542 | 0.9996 | adrenodoxin [Source:VB Community Annotation] |
| AAEL021679 | NA | 0.1051 | 1.5989 | 0.9543 | 0.9996 | NA |
| AAEL008027 | | 0.0465 | 2.9328 | 0.9543 | 0.9996 | Histone-lysine N-methyltransferase [Source:UniProtKB/TrEMBL;Acc:Q16ZX8] |
| AAEL000850 | | -0.0275 | 4.1090 | 0.9544 | 0.9996 | tyrosine kinase receptor [Source:VB Community Annotation] |
| AAEL008443 | | 0.0583 | 1.9928 | 0.9544 | 0.9996 | |
| AAEL025386 | NA | -0.0816 | -0.7176 | 0.9545 | 0.9996 | NA |
| AAEL024615 | NA | -0.1042 | 1.8113 | 0.9545 | 0.9996 | NA |
| AAEL010907 | | 0.0940 | 0.1374 | 0.9545 | 0.9996 | |
| AAEL007002 | | -0.0995 | 0.0692 | 0.9545 | 0.9996 | calsenilin [Source:VB Community Annotation] |
| AAEL002950 | | -0.0241 | 4.8871 | 0.9546 | 0.9996 | |
| AAEL012708 | | -0.0697 | 1.9325 | 0.9546 | 0.9996 | |
| AAEL003495 | | -0.0225 | 2.4098 | 0.9547 | 0.9996 | |
| AAEL019499 | NA | -0.0768 | 2.7094 | 0.9547 | 0.9996 | NA |
| AAEL010998 | | -0.0649 | -1.7254 | 0.9548 | 0.9996 | |
| AAEL006439 | | -0.0333 | 6.4201 | 0.9549 | 0.9996 | testis/ seletal muscle  dual specificty phosphatase [Source:VB Community Annotation] |
| AAEL006872 | | -0.0223 | 6.6317 | 0.9549 | 0.9996 | calponin/transgelin [Source:VB Community Annotation] |
| AAEL014445 | | 0.0573 | 1.9870 | 0.9549 | 0.9996 | |
| AAEL009927 | | 0.0378 | 5.3554 | 0.9549 | 0.9996 | |
| AAEL028170 | NA | -0.0896 | 2.0243 | 0.9550 | 0.9996 | NA |
| AAEL006467 | | 0.0571 | 1.2241 | 0.9551 | 0.9996 | alcohol dehydrogenase [Source:VB Community Annotation] |
| AAEL014153 | | -0.0321 | 3.1513 | 0.9551 | 0.9996 | |

| | | | | | | |
|---|---|---|---|---|---|---|
| AAEL011519 | | 0.0758 | -0.5721 | 0.9552 | 0.9996 | sucrose transport protein [Source:VB Community Annotation] |
| AAEL025303 | NA | -0.1303 | -1.5912 | 0.9552 | 0.9996 | NA |
| AAEL022851 | NA | -0.0527 | 0.0357 | 0.9553 | 0.9996 | NA |
| AAEL001397 | | 0.0387 | 3.7802 | 0.9554 | 0.9996 | ribonuclease [Source:VB Community Annotation] |
| AAEL005903 | | 0.0365 | 5.5857 | 0.9555 | 0.9996 | UDP-glucuronosyltransferase, putative [Source:VB Community Annotation] |
| AAEL021372 | NA | -0.1521 | -0.1383 | 0.9555 | 0.9996 | NA |
| AAEL007655 | | 0.0249 | 2.3726 | 0.9555 | 0.9996 | |
| AAEL021694 | NA | -0.0187 | 5.8317 | 0.9556 | 0.9996 | NA |
| AAEL027560 | NA | -0.0180 | 3.9868 | 0.9561 | 0.9996 | NA |
| AAEL026688 | NA | -0.0284 | 2.9604 | 0.9563 | 0.9996 | NA |
| AAEL001001 | | 0.0206 | 3.6000 | 0.9565 | 0.9996 | |
| AAEL004905 | | -0.0157 | 5.0595 | 0.9565 | 0.9996 | monocarboxylate transporter [Source:VB Community Annotation] |
| AAEL025716 | NA | 0.0736 | -1.7983 | 0.9565 | 0.9996 | NA |
| AAEL024365 | NA | -0.0618 | -0.5665 | 0.9566 | 0.9996 | NA |
| AAEL006352 | | 0.0284 | 4.6628 | 0.9566 | 0.9996 | |
| AAEL023999 | NA | -0.0677 | -0.5966 | 0.9566 | 0.9996 | NA |
| AAEL022409 | NA | -0.0457 | 2.7595 | 0.9567 | 0.9996 | NA |
| AAEL006482 | | -0.0242 | 4.4634 | 0.9568 | 0.9996 | sugar transporter [Source:VB Community Annotation] |
| AAEL024371 | NA | 0.0711 | -1.2986 | 0.9568 | 0.9996 | NA |
| AAEL006650 | | 0.0857 | 2.0962 | 0.9570 | 0.9996 | potassium channel beta [Source:VB Community Annotation] |
| AAEL006791 | | -0.0252 | 5.6918 | 0.9570 | 0.9996 | rab6 GTPase activating protein, gapcena (rabgap1 protein) [Source:VB Community Annotation] |
| AAEL013251 | | -0.0688 | 4.4475 | 0.9571 | 0.9996 | Protein quiver (Fragment) [Source:UniProtKB/TrEMBL;Acc:Q16JQ1] |
| AAEL019457 | NA | 0.0599 | 5.3191 | 0.9572 | 0.9996 | NA |
| AAEL001549 | | -0.0160 | 5.2352 | 0.9574 | 0.9996 | protein kinase c [Source:VB Community Annotation] |
| AAEL011612 | CTLMA6 | -0.0197 | 4.9393 | 0.9575 | 0.9996 | C-Type Lectin (CTL) - mannose binding. Transcript A [Source:VB Community Annotation] |
| AAEL021979 | NA | 0.0182 | 4.9806 | 0.9575 | 0.9996 | NA |
| AAEL003097 | | -0.0382 | 4.7679 | 0.9576 | 0.9996 | calmodulin-binding transcription activator (camta), drome [Source:VB Community Annotation] |
| AAEL011365 | | -0.0231 | 2.7802 | 0.9576 | 0.9996 | |
| AAEL004469 | | -0.0138 | 5.7171 | 0.9578 | 0.9996 | adaptin, alpha/gamma/epsilon [Source:VB Community Annotation] |
| AAEL001672 | | -0.0373 | 2.3478 | 0.9579 | 0.9996 | multicopper oxidase [Source:VB Community Annotation] |
| AAEL009070 | | -0.0498 | 2.0564 | 0.9579 | 0.9996 | |
| AAEL024283 | NA | 0.0525 | 6.0577 | 0.9579 | 0.9996 | NA |
| AAEL010027 | | -0.0214 | 4.8358 | 0.9580 | 0.9996 | CRAL/TRIO domain-containing protein [Source:VB Community Annotation] |
| AAEL001198 | | -0.0175 | 5.8135 | 0.9581 | 0.9996 | sodium/solute symporter [Source:VB Community Annotation] |
| AAEL017515 | | 0.0753 | 2.1645 | 0.9581 | 0.9996 | |
| AAEL013197 | | 0.0525 | 0.0949 | 0.9582 | 0.9996 | |
| AAEL014516 | | 0.0879 | 5.5015 | 0.9582 | 0.9996 | metalloproteinase, putative [Source:VB Community Annotation] |
| AAEL005230 | | -0.0434 | -1.1391 | 0.9584 | 0.9996 | |
| AAEL000644 | | -0.0158 | 5.1541 | 0.9584 | 0.9996 | |
| AAEL009406 | | -0.0155 | 6.6589 | 0.9584 | 0.9996 | n(4)-(beta-n-acetylglucosaminyl)-l-asparaginase [Source:VB Community Annotation] |

| | | | | | | |
|---|---|---|---|---|---|---|
| AAEL008673 | | -0.0451 | -0.1482 | 0.9585 | 0.9996 | ras-related protein Rab-9, putative [Source:VB Community Annotation] |
| AAEL027580 | NA | -0.0611 | 0.1674 | 0.9586 | 0.9996 | NA |
| AAEL012511 | | 0.0111 | 6.7465 | 0.9587 | 0.9996 | |
| AAEL010673 | | 0.0297 | 10.3320 | 0.9588 | 0.9996 | NADH dehydrogenase, putative [Source:VB Community Annotation] |
| AAEL011658 | | -0.0184 | 8.2197 | 0.9588 | 0.9996 | plasma glutamate carboxypeptidase [Source:VB Community Annotation] |
| AAEL006737 | | -0.0293 | 2.9685 | 0.9589 | 0.9996 | |
| AAEL022665 | NA | -0.1421 | 2.7153 | 0.9589 | 0.9996 | NA |
| AAEL011276 | | 0.0104 | 5.7844 | 0.9589 | 0.9996 | mitochondrial glutamate carrier protein [Source:VB Community Annotation] |
| AAEL001624 | | 0.0221 | 1.5499 | 0.9590 | 0.9996 | |
| AAEL008794 | | 0.0238 | 3.9543 | 0.9592 | 0.9996 | |
| AAEL011688 | | 0.0221 | 3.3221 | 0.9593 | 0.9996 | cpg binding protein [Source:VB Community Annotation] |
| AAEL015014 | | 0.0197 | 6.3716 | 0.9593 | 0.9996 | |
| AAEL018031 | | 0.0592 | -0.5223 | 0.9594 | 0.9996 | |
| AAEL004889 | | -0.0282 | 4.9815 | 0.9594 | 0.9996 | |
| AAEL000230 | | 0.0223 | 4.4640 | 0.9595 | 0.9996 | |
| AAEL000951 | | -0.0183 | 9.1052 | 0.9595 | 0.9996 | elongation factor 1-beta2 [Source:VB Community Annotation] |
| AAEL005691 | | -0.0429 | 3.8689 | 0.9596 | 0.9996 | protein serine/threonine kinase, putative [Source:VB Community Annotation] |
| AAEL020536 | NA | -0.0179 | 3.1674 | 0.9597 | 0.9996 | NA |
| AAEL005043 | | -0.0606 | -0.9532 | 0.9598 | 0.9996 | ATP-dependent bile acid permease [Source:VB Community Annotation] |
| AAEL023335 | NA | 0.0760 | 2.2499 | 0.9599 | 0.9996 | NA |
| AAEL024181 | NA | -0.0643 | 3.9817 | 0.9599 | 0.9996 | NA |
| AAEL004290 | | 0.0215 | 5.0702 | 0.9601 | 0.9996 | histone-lysine n-methyltransferase [Source:VB Community Annotation] |
| AAEL003317 | | -0.0891 | 0.8931 | 0.9604 | 0.9996 | alkaline phosphatase [Source:VB Community Annotation] |
| AAEL002742 | | -0.0593 | -0.8162 | 0.9604 | 0.9996 | |
| AAEL011452 | | -0.0378 | 1.0744 | 0.9604 | 0.9996 | |
| AAEL019473 | NA | 0.0540 | 3.4996 | 0.9604 | 0.9996 | NA |
| AAEL020804 | NA | 0.0817 | -0.9533 | 0.9605 | 0.9996 | NA |
| AAEL014551 | | -0.0279 | 3.1391 | 0.9605 | 0.9996 | triacylglycerol lipase, pancreatic [Source:VB Community Annotation] |
| AAEL001260 | Med20 | 0.0194 | 3.3137 | 0.9606 | 0.9996 | mediator of RNA polymerase II transcription subunit 20 (Med20) [Source:VB Community Annotation] |
| AAEL013768 | | 0.0127 | 5.5759 | 0.9608 | 0.9996 | glial maturation factor [Source:VB Community Annotation] |
| AAEL009763 | | 0.1019 | 0.0660 | 0.9609 | 0.9996 | |
| AAEL019931 | NA | -0.0239 | 2.4641 | 0.9610 | 0.9996 | NA |
| AAEL006918 | | -0.0145 | 4.6786 | 0.9613 | 0.9996 | |
| AAEL011578 | | 0.0253 | 1.9203 | 0.9613 | 0.9996 | serine/threonine protein kinase [Source:VB Community Annotation] |
| AAEL005628 | | 0.0154 | 5.2716 | 0.9614 | 0.9996 | |
| AAEL007279 | | 0.0267 | 2.0022 | 0.9614 | 0.9996 | |
| AAEL013887 | | -0.0165 | 4.1896 | 0.9614 | 0.9996 | |
| AAEL000504 | | -0.0987 | 0.5744 | 0.9614 | 0.9996 | |
| AAEL000726 | | -0.0809 | 8.9511 | 0.9614 | 0.9996 | fibrinogen and fibronectin [Source:VB Community Annotation] |
| AAEL011125 | | -0.0365 | 0.8870 | 0.9614 | 0.9996 | |

| | | | | | | |
|---|---|---|---|---|---|---|
| AAEL026896 | NA | 0.0883 | 4.0651 | 0.9615 | 0.9996 | NA |
| AAEL013771 | mRpL54 | -0.0194 | 5.6102 | 0.9615 | 0.9996 | mitochondrial ribosomal protein, L54, putative [Source:VB Community Annotation] |
| AAEL006146 | | -0.0378 | 0.7741 | 0.9615 | 0.9996 | |
| AAEL010563 | | -0.0232 | 4.0060 | 0.9615 | 0.9996 | tyrosyl-tRNA synthetase [Source:VB Community Annotation] |
| AAEL023429 | NA | 0.0515 | -0.6794 | 0.9616 | 0.9996 | NA |
| AAEL008469 | | 0.0220 | 4.6997 | 0.9617 | 0.9996 | |
| AAEL005747 | | -0.0185 | 4.6906 | 0.9618 | 0.9996 | |
| AAEL013637 | | -0.0360 | 5.1430 | 0.9618 | 0.9996 | homogentisate 1,2-dioxygenase [Source:VB Community Annotation] |
| AAEL017888 | U5 | 0.0344 | 1.5731 | 0.9618 | 0.9996 | U5 spliceosomal RNA [Source:RFAM;Acc:RF00020] |
| AAEL003791 | | -0.0195 | 4.1948 | 0.9619 | 0.9996 | |
| AAEL014068 | | -0.0123 | 4.7477 | 0.9619 | 0.9996 | |
| AAEL003239 | | -0.0417 | -0.2835 | 0.9619 | 0.9996 | pupal cuticle protein, putative [Source:VB Community Annotation] |
| AAEL003071 | | 0.0194 | 4.9825 | 0.9620 | 0.9996 | tRNA pseudouridine synthase D [Source:VB Community Annotation] |
| AAEL012916 | | -0.0471 | -0.2084 | 0.9620 | 0.9996 | |
| AAEL009448 | | -0.0188 | 4.0207 | 0.9621 | 0.9996 | |
| AAEL012693 | | 0.0319 | 1.0414 | 0.9621 | 0.9996 | tripartite motif protein [Source:VB Community Annotation] |
| AAEL009881 | | 0.0131 | 5.9222 | 0.9623 | 0.9996 | dynein heavy chain [Source:VB Community Annotation] |
| AAEL001925 | | -0.0236 | 5.7743 | 0.9623 | 0.9996 | |
| AAEL023863 | NA | 0.0304 | 2.1006 | 0.9624 | 0.9996 | NA |
| AAEL023945 | NA | 0.0646 | 3.5409 | 0.9624 | 0.9996 | NA |
| AAEL008680 | | 0.0335 | 3.0326 | 0.9625 | 0.9996 | Ubiquitin-related modifier 1 homolog [Source:UniProtKB/Swiss-Prot;Acc:Q16Y28] |
| AAEL022771 | NA | 0.0889 | 1.1764 | 0.9625 | 0.9996 | NA |
| AAEL020279 | NA | -0.1003 | 2.1855 | 0.9626 | 0.9996 | NA |
| AAEL001978 | | 0.0193 | 3.7144 | 0.9627 | 0.9996 | uroporphyrinogen iii synthase [Source:VB Community Annotation] |
| AAEL020238 | NA | 0.0268 | 11.6739 | 0.9627 | 0.9996 | NA |
| AAEL012679 | | -0.0451 | 0.8749 | 0.9628 | 0.9996 | juvenile hormone-inducible protein, putative [Source:VB Community Annotation] |
| AAEL025979 | NA | -0.0188 | 4.5403 | 0.9628 | 0.9996 | NA |
| AAEL004719 | | -0.0442 | 4.3694 | 0.9629 | 0.9996 | |
| AAEL009131 | CYP6Z8 | -0.0864 | 0.5741 | 0.9630 | 0.9996 | cytochrome P450 [Source:VB Community Annotation] |
| AAEL027257 | NA | -0.0503 | 2.6066 | 0.9630 | 0.9996 | NA |
| AAEL006221 | | -0.0180 | 5.2135 | 0.9631 | 0.9996 | o-sialoglycoprotein endopeptidase [Source:VB Community Annotation] |
| AAEL006095 | | -0.0187 | 4.7494 | 0.9632 | 0.9996 | Gelsolin precursor [Source:VB Community Annotation] |
| AAEL017385 | | 0.0198 | 5.4933 | 0.9632 | 0.9996 | |
| AAEL027697 | NA | 0.0262 | 3.4799 | 0.9633 | 0.9996 | NA |
| AAEL005928 | | -0.0152 | 6.1660 | 0.9634 | 0.9996 | |
| AAEL007637 | | -0.0649 | 0.6532 | 0.9635 | 0.9996 | AAA ATPase [Source:VB Community Annotation] |
| AAEL001139 | | -0.0844 | 0.7119 | 0.9635 | 0.9996 | br serine/threonine-protein kinase [Source:VB Community Annotation] |
| AAEL003036 | | -0.0916 | 0.7252 | 0.9636 | 0.9996 | |
| AAEL013825 | | 0.0113 | 4.1451 | 0.9637 | 0.9996 | helicase [Source:VB Community Annotation] |
| AAEL012261 | | -0.0152 | 3.5894 | 0.9637 | 0.9996 | Probable cytosolic Fe-S cluster assembly factor AAEL012261 [Source:UniProtKB/Swiss-Prot;Acc:Q16ML2] |

| | | | | | | |
|---|---|---|---|---|---|---|
| AAEL019467 | NA | -0.0616 | 1.7609 | 0.9637 | 0.9996 | NA |
| AAEL019788 | NA | 0.0190 | 6.7428 | 0.9638 | 0.9996 | NA |
| AAEL023444 | NA | 0.0333 | 2.7767 | 0.9638 | 0.9996 | NA |
| AAEL009201 | | -0.0165 | 11.0223 | 0.9638 | 0.9996 | |
| AAEL001168 | | 0.0675 | 2.5292 | 0.9638 | 0.9996 | |
| AAEL014255 | | 0.0255 | 4.7945 | 0.9640 | 0.9996 | aquaporin, putative [Source:VB Community Annotation] |
| AAEL024828 | NA | 0.0570 | 4.1370 | 0.9642 | 0.9996 | NA |
| AAEL024123 | NA | 0.0414 | -0.3933 | 0.9642 | 0.9996 | NA |
| AAEL009180 | | -0.0423 | 4.5053 | 0.9642 | 0.9996 | GTP-binding protein rit [Source:VB Community Annotation] |
| AAEL014419 | | 0.0425 | 7.6049 | 0.9643 | 0.9996 | |
| AAEL013068 | | 0.0104 | 8.1760 | 0.9644 | 0.9996 | protein phsophatase-2a [Source:VB Community Annotation] |
| AAEL013159 | | -0.0629 | -1.1577 | 0.9645 | 0.9996 | tartan [Source:VB Community Annotation] |
| AAEL010572 | | 0.0174 | 3.7631 | 0.9645 | 0.9996 | late endosomal/lysosomal MP1 interacting protein, putative [Source:VB Community Annotation] |
| AAEL003909 | | 0.0164 | 5.7735 | 0.9646 | 0.9996 | |
| AAEL005552 | | 0.0307 | 6.8845 | 0.9647 | 0.9996 | succinyl-CoA synthetase beta chain [Source:VB Community Annotation] |
| AAEL003881 | | 0.1091 | 3.8715 | 0.9647 | 0.9996 | ubiquitin, putative [Source:VB Community Annotation] |
| AAEL004143 | | 0.0130 | 3.8927 | 0.9648 | 0.9996 | n-acetylglucosaminyl-phosphatidylinositol de-n-acetylase [Source:VB Community Annotation] |
| AAEL011996 | AKHR-II | -0.0392 | 5.6720 | 0.9649 | 0.9996 | |
| AAEL005961 | | -0.0357 | 7.9636 | 0.9649 | 0.9996 | actin [Source:VB Community Annotation] |
| AAEL026531 | NA | 0.0233 | 2.6611 | 0.9650 | 0.9996 | NA |
| AAEL012962 | | 0.0159 | 3.4706 | 0.9651 | 0.9996 | DEAD box ATP-dependent RNA helicase [Source:VB Community Annotation] |
| AAEL009980 | | 0.0246 | 5.1289 | 0.9652 | 0.9996 | |
| AAEL006313 | | -0.0115 | 4.7694 | 0.9653 | 0.9996 | |
| AAEL019834 | NA | -0.0265 | 2.4361 | 0.9654 | 0.9996 | NA |
| AAEL013075 | | 0.0190 | 5.0769 | 0.9655 | 0.9996 | |
| AAEL002874 | | -0.0179 | 2.7589 | 0.9656 | 0.9996 | apolipoprotein a binding protein [Source:VB Community Annotation] |
| AAEL000915 | | 0.0787 | 0.8201 | 0.9656 | 0.9996 | |
| AAEL008134 | | -0.0130 | 6.7826 | 0.9658 | 0.9996 | ABC transporter [Source:VB Community Annotation] |
| AAEL007758 | | -0.0167 | 3.5605 | 0.9658 | 0.9996 | |
| AAEL027786 | NA | 0.0167 | 6.6858 | 0.9658 | 0.9996 | NA |
| AAEL000419 | | -0.0171 | 5.1747 | 0.9659 | 0.9996 | |
| AAEL027106 | NA | -0.0123 | 4.2393 | 0.9659 | 0.9996 | NA |
| AAEL012856 | | 0.0251 | 6.4260 | 0.9659 | 0.9996 | |
| AAEL019741 | NA | 0.0529 | 5.9064 | 0.9660 | 0.9996 | NA |
| AAEL009515 | | 0.0591 | 2.6202 | 0.9661 | 0.9996 | |
| AAEL002501 | | 0.0147 | 7.6692 | 0.9661 | 0.9996 | protein disulfide isomerase [Source:VB Community Annotation] |
| AAEL003640 | | -0.0317 | 3.0915 | 0.9662 | 0.9996 | sodium/chloride dependent amino acid transporter [Source:VB Community Annotation] |
| AAEL001532 | | -0.0217 | 5.7813 | 0.9663 | 0.9996 | FAD NAD binding oxidoreductases [Source:VB Community Annotation] |
| AAEL013015 | | 0.0509 | -0.1098 | 0.9664 | 0.9996 | |
| AAEL019586 | NA | -0.0134 | 5.8155 | 0.9665 | 0.9996 | NA |

| | | | | | | |
|---|---|---|---|---|---|---|
| AAEL005002 | | 0.0151 | 3.5545 | 0.9667 | 0.9996 | roundabout [Source:VB Community Annotation] |
| AAEL006480 | | 0.0102 | 6.9847 | 0.9670 | 0.9996 | |
| AAEL021417 | NA | 0.0613 | 2.5554 | 0.9672 | 0.9996 | NA |
| AAEL010536 | | -0.0223 | 2.1728 | 0.9676 | 0.9996 | alpha-amylase [Source:VB Community Annotation] |
| AAEL008953 | | -0.0140 | 6.9856 | 0.9676 | 0.9996 | |
| AAEL022731 | NA | -0.0406 | 7.1708 | 0.9676 | 0.9996 | NA |
| AAEL014831 | | -0.0134 | 6.6241 | 0.9677 | 0.9996 | RNA polymerase II subunit Rpb10, putative [Source:VB Community Annotation] |
| AAEL000523 | | -0.0406 | 4.0993 | 0.9678 | 0.9996 | ring finger protein [Source:VB Community Annotation] |
| AAEL025024 | NA | -0.0462 | -1.4243 | 0.9678 | 0.9996 | NA |
| AAEL019903 | NA | -0.0214 | 4.3683 | 0.9678 | 0.9996 | NA |
| AAEL007845 | | -0.0119 | 8.9745 | 0.9678 | 0.9996 | rab5 [Source:VB Community Annotation] |
| AAEL010835 | | 0.0574 | 2.0312 | 0.9679 | 0.9996 | mitogen-activated protein kinase kinase kinase [Source:VB Community Annotation] |
| AAEL010977 | | -0.0105 | 9.6653 | 0.9682 | 0.9996 | ATP-dependent transporter [Source:VB Community Annotation] |
| AAEL001764 | | -0.0130 | 4.7090 | 0.9682 | 0.9996 | histone-fold protein CHRAC subunit, putative [Source:VB Community Annotation] |
| AAEL013278 | | 0.0449 | 2.3539 | 0.9682 | 0.9996 | ga binding protein beta chain (transcription factor e4tf1-47) [Source:VB Community Annotation] |
| AAEL019506 | NA | -0.0232 | 3.7337 | 0.9682 | 0.9996 | NA |
| AAEL014016 | | 0.0106 | 5.1341 | 0.9683 | 0.9996 | breast cancer metastasis-suppressor [Source:VB Community Annotation] |
| AAEL003515 | | -0.0121 | 2.9677 | 0.9683 | 0.9996 | |
| AAEL003853 | | -0.0438 | 4.3590 | 0.9683 | 0.9996 | nephrin [Source:VB Community Annotation] |
| AAEL006980 | | -0.0137 | 5.1718 | 0.9683 | 0.9996 | lipase [Source:VB Community Annotation] |
| AAEL005263 | | 0.0198 | 5.3327 | 0.9683 | 0.9996 | U3 small nucleolar ribonucleoprotein protein IMP3 (U3 snoRNP protein IMP3), putative [Source:VB Community Annotation] |
| AAEL016607 | tRNA-Pro | 0.0379 | 0.0655 | 0.9684 | 0.9996 | |
| AAEL010776 | | -0.0831 | 0.8164 | 0.9685 | 0.9996 | carboxypeptidase [Source:VB Community Annotation] |
| AAEL003469 | | 0.0178 | 5.2132 | 0.9686 | 0.9996 | NHP2 protein, putative [Source:VB Community Annotation] |
| AAEL000757 | | -0.0392 | 5.8029 | 0.9686 | 0.9996 | anterior fat body protein [Source:VB Community Annotation] |
| AAEL006792 | | 0.0105 | 5.2140 | 0.9686 | 0.9996 | |
| AAEL004835 | | 0.0152 | 3.9509 | 0.9686 | 0.9996 | |
| AAEL019887 | NA | -0.0531 | -0.2169 | 0.9688 | 0.9996 | NA |
| AAEL008719 | | 0.0120 | 7.2695 | 0.9688 | 0.9996 | Sm protein G, putative [Source:VB Community Annotation] |
| AAEL009863 | | 0.0207 | 6.2669 | 0.9689 | 0.9996 | sodium/dicarboxylate cotransporter, putative [Source:VB Community Annotation] |
| AAEL011319 | | -0.0240 | 6.4423 | 0.9690 | 0.9996 | |
| AAEL005659 | | 0.0141 | 5.4275 | 0.9690 | 0.9996 | |
| AAEL002859 | | 0.0281 | 5.1390 | 0.9691 | 0.9996 | |
| AAEL015503 | | -0.0360 | -0.7486 | 0.9694 | 0.9996 | |
| AAEL021023 | NA | -0.1031 | 2.9999 | 0.9695 | 0.9996 | NA |
| AAEL003705 | | 0.0120 | 4.2644 | 0.9696 | 0.9996 | seven transmembrane protein, putative [Source:VB Community Annotation] |
| AAEL003509 | | 0.0147 | 4.9551 | 0.9697 | 0.9996 | smap1 [Source:VB Community Annotation] |
| AAEL003730 | mRpS28 | -0.0116 | 4.9469 | 0.9697 | 0.9996 | mitochondrial ribosomal protein, S28, putative [Source:VB Community Annotation] |
| AAEL014447 | | 0.0238 | 2.8299 | 0.9698 | 0.9996 | |
| AAEL008015 | | 0.0102 | 5.7295 | 0.9698 | 0.9996 | |

| | | | | | | |
|---|---|---|---|---|---|---|
| AAEL004110 | | -0.0103 | 4.2297 | 0.9698 | 0.9996 | cdk10/11 [Source:VB Community Annotation] |
| AAEL003041 | | 0.0414 | 4.4616 | 0.9699 | 0.9996 | |
| AAEL004382 | | -0.0646 | 9.7195 | 0.9699 | 0.9996 | |
| AAEL024838 | NA | -0.0191 | 3.3295 | 0.9700 | 0.9996 | NA |
| AAEL010950 | | 0.0254 | 6.8556 | 0.9701 | 0.9996 | |
| AAEL011255 | | 0.0207 | 7.0433 | 0.9701 | 0.9996 | GTP-binding protein-invertebrate [Source:VB Community Annotation] |
| AAEL011289 | | -0.0194 | 5.8247 | 0.9701 | 0.9996 | |
| AAEL013774 | | -0.0464 | 3.0161 | 0.9701 | 0.9996 | |
| AAEL014852 | | 0.0249 | 1.8073 | 0.9702 | 0.9996 | |
| AAEL010885 | | -0.0131 | 2.9813 | 0.9702 | 0.9996 | |
| AAEL006059 | | -0.0097 | 5.0820 | 0.9702 | 0.9996 | |
| AAEL024645 | NA | -0.0449 | -0.6488 | 0.9703 | 0.9996 | NA |
| AAEL001113 | | 0.0119 | 7.8885 | 0.9703 | 0.9996 | inorganic phosphate cotransporter, putative [Source:VB Community Annotation] |
| AAEL014640 | PGRPLC | 0.0175 | 6.8134 | 0.9705 | 0.9996 | Peptidoglycan Recognition Protein (Long) [Source:VB Community Annotation] |
| AAEL011186 | | 0.0156 | 2.1101 | 0.9705 | 0.9996 | |
| AAEL011992 | | -0.0165 | 9.9756 | 0.9707 | 0.9996 | NADH:ubiquinone dehydrogenase, putative [Source:VB Community Annotation] |
| AAEL003282 | | -0.0099 | 5.7378 | 0.9707 | 0.9996 | ezrin-binding protein pace-1 [Source:VB Community Annotation] |
| AAEL007158 | | 0.0160 | 3.7582 | 0.9708 | 0.9996 | nnp-1 protein (novel nuclear protein 1) (nop52) [Source:VB Community Annotation] |
| AAEL012342 | | -0.0319 | 0.4937 | 0.9708 | 0.9996 | lysosomal acid lipase, putative [Source:VB Community Annotation] |
| AAEL007920 | | -0.0133 | 8.4692 | 0.9710 | 0.9996 | |
| AAEL006062 | | -0.0139 | 4.0410 | 0.9713 | 0.9996 | neuralized [Source:VB Community Annotation] |
| AAEL000090 | | -0.0080 | 6.9236 | 0.9714 | 0.9996 | secretory carrier-associated membrane protein (scamp) [Source:VB Community Annotation] |
| AAEL004731 | | -0.0431 | 1.5677 | 0.9714 | 0.9996 | |
| AAEL026737 | NA | 0.0156 | 2.2815 | 0.9715 | 0.9996 | NA |
| AAEL020543 | NA | -0.0119 | 3.2657 | 0.9715 | 0.9996 | NA |
| AAEL009320 | | -0.0142 | 6.6465 | 0.9715 | 0.9996 | chaperonin [Source:VB Community Annotation] |
| AAEL004972 | | -0.0274 | 5.1673 | 0.9715 | 0.9996 | |
| AAEL016989 | | -0.0125 | 2.9832 | 0.9716 | 0.9996 | |
| AAEL002442 | | 0.0211 | 0.9634 | 0.9716 | 0.9996 | |
| AAEL009871 | | -0.0131 | 3.6178 | 0.9718 | 0.9996 | 80 kda MCM3-associated protein [Source:VB Community Annotation] |
| AAEL003478 | | -0.0140 | 3.1785 | 0.9718 | 0.9996 | |
| AAEL017397 | | 0.0276 | 5.5939 | 0.9720 | 0.9996 | |
| AAEL011357 | | 0.0153 | 4.4818 | 0.9721 | 0.9996 | maintenance of killer 16 (mak16) protein [Source:VB Community Annotation] |
| AAEL003493 | | 0.0285 | 3.1421 | 0.9721 | 0.9996 | GDI interacting protein, putative [Source:VB Community Annotation] |
| AAEL005687 | | -0.0183 | 7.8908 | 0.9721 | 0.9996 | protein serine/threonine kinase, putative [Source:VB Community Annotation] |
| AAEL002392 | | 0.0569 | 2.4034 | 0.9722 | 0.9996 | |
| AAEL013764 | | -0.0171 | 1.9885 | 0.9722 | 0.9996 | WD-repeat protein [Source:VB Community Annotation] |
| AAEL003519 | | -0.0126 | 4.3397 | 0.9723 | 0.9996 | |
| AAEL029016 | NA | -0.0127 | 4.6302 | 0.9725 | 0.9996 | NA |
| AAEL008427 | | 0.0103 | 3.9871 | 0.9728 | 0.9996 | smad nuclear interacting protein [Source:VB Community Annotation] |

| | | | | | | |
|---|---|---|---|---|---|---|
| AAEL009274 | | 0.0117 | 5.2257 | 0.9729 | 0.9996 | |
| AAEL002349 | | -0.0125 | 4.0005 | 0.9730 | 0.9996 | |
| AAEL014196 | | -0.0137 | 2.8356 | 0.9732 | 0.9996 | |
| AAEL010462 | | -0.0548 | 4.3882 | 0.9732 | 0.9996 | vesamicol binding protein, putative [Source:VB Community Annotation] |
| AAEL017563 | | 0.0569 | 8.2305 | 0.9734 | 0.9996 | |
| AAEL012478 | | 0.0226 | -0.7072 | 0.9738 | 0.9996 | glucose transporter, putative [Source:VB Community Annotation] |
| AAEL016638 | tRNA-Ala | 0.0341 | -0.8197 | 0.9740 | 0.9996 | |
| AAEL019738 | NA | 0.0452 | -1.4605 | 0.9740 | 0.9996 | NA |
| AAEL003855 | | 0.0091 | 4.1400 | 0.9741 | 0.9996 | flightless-I, putative [Source:VB Community Annotation] |
| AAEL006341 | | 0.0128 | 2.6547 | 0.9742 | 0.9996 | |
| AAEL014455 | | 0.0237 | 0.8736 | 0.9742 | 0.9996 | |
| AAEL002343 | | -0.0204 | 0.9147 | 0.9744 | 0.9996 | neuronal pas domain protein [Source:VB Community Annotation] |
| AAEL020061 | NA | -0.0558 | 0.8915 | 0.9744 | 0.9996 | NA |
| AAEL008269 | | -0.0121 | 3.3923 | 0.9745 | 0.9996 | |
| AAEL009157 | | -0.0167 | 5.2220 | 0.9745 | 0.9996 | RHO GTPase activator, putative [Source:VB Community Annotation] |
| AAEL021274 | NA | -0.0373 | -1.3505 | 0.9747 | 0.9996 | NA |
| AAEL011449 | | -0.0249 | 1.5451 | 0.9747 | 0.9996 | |
| AAEL026364 | NA | 0.0550 | -0.9558 | 0.9748 | 0.9996 | NA |
| AAEL000986 | | -0.0119 | 8.9766 | 0.9756 | 0.9996 | NADH-ubiquinone oxidoreductase ashi subunit [Source:VB Community Annotation] |
| AAEL007657 | | 0.0382 | -1.9993 | 0.9757 | 0.9996 | low-density lipoprotein receptor (ldl) [Source:VB Community Annotation] |
| AAEL021077 | NA | -0.0209 | 2.1331 | 0.9757 | 0.9996 | NA |
| AAEL023512 | NA | 0.0376 | 4.2122 | 0.9759 | 0.9996 | NA |
| AAEL023746 | NA | -0.0152 | 1.9352 | 0.9760 | 0.9996 | NA |
| AAEL006180 | CCC1 | -0.0128 | 3.2131 | 0.9761 | 0.9996 | sodium-coupled cation-chloride cotransporter [Source:VB Community Annotation] |
| AAEL000166 | | -0.0406 | 4.2944 | 0.9762 | 0.9996 | |
| AAEL021230 | NA | 0.0116 | 6.5416 | 0.9762 | 0.9996 | NA |
| AAEL007450 | | -0.0199 | 1.3823 | 0.9763 | 0.9996 | odd skipped, putative [Source:VB Community Annotation] |
| AAEL009047 | | 0.0353 | 1.1455 | 0.9766 | 0.9996 | |
| AAEL014605 | CYP9J9 | -0.0102 | 7.6760 | 0.9766 | 0.9996 | cytochrome P450 [Source:VB Community Annotation] |
| AAEL019976 | NA | -0.0098 | 3.1153 | 0.9767 | 0.9996 | NA |
| AAEL013077 | | 0.0334 | 7.8204 | 0.9768 | 0.9996 | |
| AAEL013171 | HPX2 | 0.0119 | 6.6850 | 0.9770 | 0.9996 | heme peroxidase [Source:VB Community Annotation] |
| AAEL009769 | | 0.0106 | 3.2306 | 0.9770 | 0.9996 | |
| AAEL009045 | | -0.0224 | 2.0315 | 0.9771 | 0.9996 | amine oxidase [Source:VB Community Annotation] |
| AAEL012440 | | -0.0083 | 5.0985 | 0.9771 | 0.9996 | sodium-bile acid cotransporter [Source:VB Community Annotation] |
| AAEL000395 | USP | 0.0158 | 4.7943 | 0.9772 | 0.9996 | Ultra spiracleisoform A  nuclear receptor [Source:VB Community Annotation] |
| AAEL002170 | | -0.0080 | 5.4566 | 0.9772 | 0.9996 | phosphatidylinositol-phosphatidylcholine transfer protein, putative [Source:VB Community Annotation] |
| AAEL005225 | | 0.0100 | 3.1547 | 0.9772 | 0.9996 | |
| AAEL005402 | | -0.0177 | 2.6504 | 0.9773 | 0.9996 | |
| AAEL000918 | | -0.0136 | 1.8336 | 0.9773 | 0.9996 | Carboxylic ester hydrolase (Fragment) [Source:UniProtKB/TrEMBL;Acc:Q17MV7] |

| | | | | | | |
|---|---|---|---|---|---|---|
| AAEL022734 | NA | 0.0273 | 0.8484 | 0.9774 | 0.9996 | NA |
| AAEL019686 | NA | -0.0101 | 4.9745 | 0.9775 | 0.9996 | NA |
| AAEL006353 | | -0.0162 | 3.2973 | 0.9776 | 0.9996 | sulfotransferase (sult) [Source:VB Community Annotation] |
| AAEL025454 | NA | -0.0139 | 2.9910 | 0.9776 | 0.9996 | NA |
| AAEL003766 | | 0.0208 | 3.9015 | 0.9779 | 0.9996 | |
| AAEL015100 | | 0.0079 | 8.4665 | 0.9779 | 0.9996 | calnexin [Source:VB Community Annotation] |
| AAEL023037 | NA | 0.0180 | 6.9514 | 0.9779 | 0.9996 | NA |
| AAEL008873 | | 0.0349 | -1.7917 | 0.9780 | 0.9996 | pupal cuticle protein 78E, putative [Source:VB Community Annotation] |
| AAEL002785 | | -0.0216 | -1.0531 | 0.9780 | 0.9996 | DNA polymerase epsilon subunit b [Source:VB Community Annotation] |
| AAEL019597 | NA | -0.0221 | 4.6976 | 0.9781 | 0.9996 | NA |
| AAEL001054 | GSTD4 | -0.0301 | 3.0403 | 0.9783 | 0.9996 | glutathione S-transferase (GSTD4) [Source:VB Community Annotation] |
| AAEL010228 | SAAG-4 | -0.0438 | 8.0808 | 0.9783 | 0.9996 | |
| AAEL010933 | | -0.0361 | 3.0156 | 0.9784 | 0.9996 | |
| AAEL006542 | | 0.0117 | 4.9898 | 0.9785 | 0.9996 | retinoid-inducible serine carboxypeptidase (serine carboxypeptidase [Source:VB Community Annotation] |
| AAEL012119 | | 0.0204 | 4.4416 | 0.9785 | 0.9996 | |
| AAEL000223 | | 0.0406 | -0.0214 | 0.9785 | 0.9996 | alpha-glucosidase [Source:VB Community Annotation] |
| AAEL001459 | | -0.0333 | -0.0676 | 0.9785 | 0.9996 | deltex [Source:VB Community Annotation] |
| AAEL021100 | NA | -0.0690 | 0.5401 | 0.9786 | 0.9996 | NA |
| AAEL006490 | GPRCAL2 | -0.0106 | 3.7896 | 0.9786 | 0.9996 | GPCR Calcitonin/Diuretic Hormone Family [Source:VB Community Annotation] |
| AAEL018300 | | 0.0132 | 2.5785 | 0.9787 | 0.9996 | |
| AAEL003162 | | 0.0291 | 0.7639 | 0.9788 | 0.9996 | formin 1,2/cappuccino [Source:VB Community Annotation] |
| AAEL013702 | | -0.0077 | 5.4686 | 0.9788 | 0.9996 | |
| AAEL008894 | | -0.0470 | 2.3450 | 0.9788 | 0.9996 | |
| AAEL006543 | | 0.0068 | 4.9580 | 0.9790 | 0.9996 | |
| AAEL000723 | | 0.0139 | 1.9258 | 0.9790 | 0.9996 | |
| AAEL022286 | NA | -0.0071 | 11.4589 | 0.9790 | 0.9996 | NA |
| AAEL027287 | NA | -0.0115 | 3.1561 | 0.9791 | 0.9996 | NA |
| AAEL004974 | | -0.0096 | 3.2043 | 0.9791 | 0.9996 | beta-1,3-glucuronyltransferase s, p [Source:VB Community Annotation] |
| AAEL024390 | NA | 0.0406 | 1.0637 | 0.9791 | 0.9996 | NA |
| AAEL010598 | | -0.0075 | 3.0277 | 0.9791 | 0.9996 | |
| AAEL007316 | | 0.0160 | 4.1224 | 0.9791 | 0.9996 | |
| AAEL027343 | NA | -0.0225 | 0.3493 | 0.9792 | 0.9996 | NA |
| AAEL015199 | | 0.0087 | 4.6517 | 0.9792 | 0.9996 | WD-repeat protein [Source:VB Community Annotation] |
| AAEL013177 | | -0.0110 | 3.4451 | 0.9793 | 0.9996 | nucleotide-binding protein, putative [Source:VB Community Annotation] |
| AAEL008886 | | -0.0110 | 4.5281 | 0.9793 | 0.9996 | |
| AAEL006653 | | 0.0105 | 3.9884 | 0.9794 | 0.9996 | Gamma-tubulin complex component [Source:UniProtKB/TrEMBL;Acc:A0A1S4FE93] |
| AAEL004299 | | -0.0281 | 0.6922 | 0.9795 | 0.9996 | angiotensin-converting-relatedenzyme [Source:VB Community Annotation] |
| AAEL012783 | | -0.0518 | 2.1098 | 0.9795 | 0.9996 | protease m1 zinc metalloprotease [Source:VB Community Annotation] |
| AAEL008636 | | -0.0073 | 4.2405 | 0.9796 | 0.9996 | |
| AAEL004829 | | -0.0088 | 8.9267 | 0.9798 | 0.9996 | NADH dehydrogenase, putative [Source:VB Community Annotation] |

| | | | | | | |
|---|---|---|---|---|---|---|
| AAEL003946 | mRpS33 | 0.0085 | 5.7048 | 0.9798 | 0.9996 | mitochondrial ribosomal protein S33, putative [Source:VB Community Annotation] |
| AAEL011538 | | 0.0087 | 3.3065 | 0.9799 | 0.9996 | |
| AAEL009676 | | 0.0099 | 5.2504 | 0.9799 | 0.9996 | glyoxylate/hydroxypyruvate reductase [Source:VB Community Annotation] |
| AAEL019728 | NA | 0.0093 | 5.6194 | 0.9801 | 0.9996 | NA |
| AAEL006001 | | -0.0079 | 5.0230 | 0.9803 | 0.9996 | |
| AAEL007128 | | 0.0445 | 1.9563 | 0.9803 | 0.9996 | sugar transporter [Source:VB Community Annotation] |
| AAEL009596 | | 0.0149 | 5.1866 | 0.9804 | 0.9996 | sterol o-acyltransferase [Source:VB Community Annotation] |
| AAEL006065 | | -0.0084 | 5.9722 | 0.9805 | 0.9996 | s-adenosyl-methyltransferase mraw [Source:VB Community Annotation] |
| AAEL013625 | RpS5 | 0.0072 | 11.8929 | 0.9805 | 0.9996 | 40S ribosomal protein S5 [Source:VB Community Annotation] |
| AAEL003680 | | -0.0124 | 3.0318 | 0.9805 | 0.9996 | ring finger protein [Source:VB Community Annotation] |
| AAEL013842 | | -0.0118 | 1.4756 | 0.9807 | 0.9996 | |
| AAEL002483 | | -0.0085 | 4.0324 | 0.9809 | 0.9996 | |
| AAEL002790 | | -0.0104 | 3.2523 | 0.9809 | 0.9996 | |
| AAEL001367 | | -0.0087 | 2.8556 | 0.9810 | 0.9996 | type IV inositol 5-phosphatase [Source:VB Community Annotation] |
| AAEL004345 | | 0.0064 | 4.5297 | 0.9812 | 0.9996 | cysteinyl-tRNA synthetase [Source:VB Community Annotation] |
| AAEL010942 | | 0.0110 | 3.7507 | 0.9814 | 0.9996 | kinesin-like protein KIF3A [Source:VB Community Annotation] |
| AAEL007631 | | -0.0072 | 4.8681 | 0.9814 | 0.9996 | |
| AAEL013963 | | -0.0080 | 4.8740 | 0.9814 | 0.9996 | tumor necrosis factor induced protein [Source:VB Community Annotation] |
| AAEL019420 | NA | -0.0050 | 5.3087 | 0.9815 | 0.9996 | NA |
| AAEL015095 | | -0.0116 | 1.1973 | 0.9815 | 0.9996 | |
| AAEL002863 | | -0.0105 | 2.6328 | 0.9816 | 0.9996 | zinc finger protein [Source:VB Community Annotation] |
| AAEL008434 | | 0.0058 | 5.5289 | 0.9816 | 0.9996 | ER lumen protein retaining receptor [Source:VB Community Annotation] |
| AAEL008188 | RpL6 | 0.0066 | 10.6235 | 0.9817 | 0.9996 | 60S ribosomal protein L6 [Source:VB Community Annotation] |
| AAEL007998 | | -0.0286 | -0.4018 | 0.9817 | 0.9996 | |
| AAEL008485 | | -0.0098 | 9.5974 | 0.9818 | 0.9996 | |
| AAEL007138 | | 0.0511 | 0.8274 | 0.9821 | 0.9996 | sugar transporter [Source:VB Community Annotation] |
| AAEL002669 | | -0.0138 | 5.1792 | 0.9821 | 0.9996 | AMP dependent ligase [Source:VB Community Annotation] |
| AAEL002106 | | -0.0094 | 3.4247 | 0.9822 | 0.9996 | |
| AAEL013557 | | -0.0060 | 3.8039 | 0.9823 | 0.9996 | |
| AAEL008535 | | 0.0213 | 0.4358 | 0.9824 | 0.9996 | |
| AAEL019804 | NA | -0.0091 | 2.8096 | 0.9824 | 0.9996 | NA |
| AAEL014267 | | 0.0172 | -0.1410 | 0.9824 | 0.9996 | |
| AAEL001715 | | -0.0059 | 6.3599 | 0.9824 | 0.9996 | chaperonin [Source:VB Community Annotation] |
| AAEL010739 | | -0.0189 | -0.1547 | 0.9825 | 0.9996 | WNT4 precursor, putative [Source:VB Community Annotation] |
| AAEL011758 | | -0.0251 | -0.8699 | 0.9825 | 0.9996 | peptidyl-prolyl cis-trans isomerase f, ppif [Source:VB Community Annotation] |
| AAEL000719 | | -0.0078 | 5.4034 | 0.9825 | 0.9996 | pak-interacting exchange factor, beta-pix/cool-1 [Source:VB Community Annotation] |
| AAEL025250 | NA | 0.0282 | 2.2135 | 0.9826 | 0.9996 | NA |
| AAEL002700 | | 0.0239 | 1.6631 | 0.9828 | 0.9996 | |
| AAEL004953 | | -0.0201 | 0.4526 | 0.9828 | 0.9996 | elongase, putative [Source:VB Community Annotation] |
| AAEL002242 | | -0.0182 | 2.0176 | 0.9829 | 0.9996 | |

| | | | | | | |
|---|---|---|---|---|---|---|
| AAEL028011 | NA | 0.0115 | 6.1015 | 0.9831 | 0.9996 | NA |
| AAEL005596 | | 0.0332 | 6.7054 | 0.9832 | 0.9996 | trypsin-epsilon, putative [Source:VB Community Annotation] |
| AAEL012579 | | 0.0075 | 6.4801 | 0.9833 | 0.9996 | Aspartate aminotransferase [Source:UniProtKB/TrEMBL;Acc:Q16LN3] |
| AAEL008170 | | 0.0061 | 4.2504 | 0.9834 | 0.9996 | |
| AAEL003966 | | -0.0136 | 5.6649 | 0.9835 | 0.9996 | lachesin, putative [Source:VB Community Annotation] |
| AAEL006537 | | 0.0070 | 6.9490 | 0.9835 | 0.9996 | |
| AAEL006510 | | 0.0174 | 4.4205 | 0.9835 | 0.9996 | |
| AAEL002906 | | 0.0057 | 5.9078 | 0.9835 | 0.9996 | 26S proteasome regulatory subunit rpn2 [Source:VB Community Annotation] |
| AAEL010960 | | 0.0080 | 4.0407 | 0.9835 | 0.9996 | xaa-pro dipeptidase app(e.coli) [Source:VB Community Annotation] |
| AAEL027598 | NA | 0.0104 | 5.9891 | 0.9836 | 0.9996 | NA |
| AAEL004086 | | -0.0074 | 4.5192 | 0.9836 | 0.9996 | aldo-keto reductase [Source:VB Community Annotation] |
| AAEL005242 | | 0.0072 | 4.0455 | 0.9837 | 0.9996 | |
| AAEL010677 | | 0.0103 | 2.9346 | 0.9837 | 0.9996 | oxidoreductase [Source:VB Community Annotation] |
| AAEL027921 | NA | 0.0095 | 0.5702 | 0.9837 | 0.9996 | NA |
| AAEL012389 | | 0.0062 | 4.1693 | 0.9838 | 0.9996 | |
| AAEL019677 | NA | -0.0074 | 3.9257 | 0.9838 | 0.9996 | NA |
| AAEL019522 | NA | 0.0136 | 1.2120 | 0.9839 | 0.9996 | NA |
| AAEL003379 | | 0.0239 | -1.2632 | 0.9840 | 0.9996 | GTPase-activating protein gyp2 [Source:VB Community Annotation] |
| AAEL000589 | | -0.0065 | 6.6597 | 0.9841 | 0.9996 | serine/threonine protein kinase [Source:VB Community Annotation] |
| AAEL025471 | NA | 0.0230 | -1.5734 | 0.9841 | 0.9996 | NA |
| AAEL013445 | | -0.0078 | 4.1664 | 0.9842 | 0.9996 | |
| AAEL008916 | | -0.0214 | 3.1746 | 0.9842 | 0.9996 | |
| AAEL003764 | | 0.0375 | 0.5734 | 0.9842 | 0.9996 | |
| AAEL024583 | NA | 0.0152 | 3.6523 | 0.9843 | 0.9996 | NA |
| AAEL014577 | | -0.0083 | 2.1848 | 0.9843 | 0.9996 | |
| AAEL001451 | | 0.0084 | 3.5214 | 0.9843 | 0.9996 | DNA repair protein Rad62, putative [Source:VB Community Annotation] |
| AAEL012701 | | -0.0073 | 5.5653 | 0.9846 | 0.9996 | ATP-binding cassette sub-family A member 3, putative [Source:VB Community Annotation] |
| AAEL008006 | | -0.0110 | 5.8516 | 0.9846 | 0.9996 | 3-hydroxyacyl-coa dehyrogenase [Source:VB Community Annotation] |
| AAEL023787 | NA | 0.0065 | 4.9769 | 0.9847 | 0.9996 | NA |
| AAEL003284 | | -0.0248 | -0.4255 | 0.9847 | 0.9996 | |
| AAEL024528 | NA | 0.0057 | 4.1125 | 0.9848 | 0.9996 | NA |
| AAEL015598 | | -0.0082 | 2.7455 | 0.9848 | 0.9996 | |
| AAEL019496 | NA | -0.0129 | 8.9794 | 0.9849 | 0.9996 | NA |
| AAEL014529 | | 0.0048 | 5.3915 | 0.9850 | 0.9996 | |
| AAEL005972 | | 0.0156 | 1.6500 | 0.9851 | 0.9996 | |
| AAEL014944 | | -0.0070 | 11.0567 | 0.9851 | 0.9996 | cytochrome c oxidase polypeptide [Source:VB Community Annotation] |
| AAEL019479 | NA | 0.0053 | 4.5428 | 0.9851 | 0.9996 | NA |
| AAEL001536 | | 0.0222 | 3.6827 | 0.9853 | 0.9996 | homeobox protein abdominal-A, putative [Source:VB Community Annotation] |
| AAEL010373 | | 0.0072 | 5.1821 | 0.9853 | 0.9996 | dullard protein [Source:VB Community Annotation] |
| AAEL010077 | | 0.0075 | 3.7981 | 0.9854 | 0.9996 | xpa-binding protein 1 (mbdin) [Source:VB Community Annotation] |

| | | | | | | |
|---|---|---|---|---|---|---|
| AAEL003858 | | -0.0168 | 1.3384 | 0.9855 | 0.9996 | UPF0466 protein AAEL003858, mitochondrial Precursor [Source:VB Community Annotation] |
| AAEL017825 | U2 | 0.0180 | -0.6041 | 0.9855 | 0.9996 | U2 spliceosomal RNA [Source:RFAM;Acc:RF00004] |
| AAEL002120 | | 0.0138 | 0.6743 | 0.9855 | 0.9996 | zinc finger protein [Source:VB Community Annotation] |
| AAEL003831 | | 0.0109 | 1.5329 | 0.9856 | 0.9996 | fatty acid hydroxylase [Source:VB Community Annotation] |
| AAEL013935 | | -0.0069 | 4.2808 | 0.9859 | 0.9996 | |
| AAEL003173 | | -0.0357 | -0.6822 | 0.9859 | 0.9996 | forkhead protein/ forkhead protein domain [Source:VB Community Annotation] |
| AAEL026008 | NA | 0.0080 | 8.3148 | 0.9860 | 0.9996 | NA |
| AAEL011980 | | 0.0190 | 3.0058 | 0.9860 | 0.9996 | |
| AAEL013888 | | 0.0048 | 5.7942 | 0.9863 | 0.9996 | |
| AAEL002687 | | -0.0063 | 7.1650 | 0.9863 | 0.9996 | sterol carrier protein-2, putative [Source:VB Community Annotation] |
| AAEL007257 | | 0.0077 | 2.6080 | 0.9863 | 0.9996 | |
| AAEL012313 | | 0.0063 | 6.9040 | 0.9864 | 0.9996 | charged multivesicular body protein 5 [Source:VB Community Annotation] |
| AAEL002312 | | -0.0058 | 4.4314 | 0.9864 | 0.9996 | |
| AAEL022693 | NA | -0.0111 | 1.6365 | 0.9864 | 0.9996 | NA |
| AAEL012868 | | -0.0072 | 6.1578 | 0.9864 | 0.9996 | cmp-n-acetylneuraminic acid synthase [Source:VB Community Annotation] |
| AAEL020986 | NA | -0.0093 | 4.0076 | 0.9866 | 0.9996 | NA |
| AAEL008701 | | -0.0140 | 7.6840 | 0.9866 | 0.9996 | myoinositol oxygenase [Source:VB Community Annotation] |
| AAEL005493 | | -0.0083 | 4.3602 | 0.9869 | 0.9996 | septin [Source:VB Community Annotation] |
| AAEL001390 | | 0.0131 | 10.6663 | 0.9870 | 0.9996 | |
| AAEL001520 | | 0.0325 | 0.7471 | 0.9870 | 0.9996 | |
| AAEL004379 | | 0.0180 | -1.1379 | 0.9872 | 0.9996 | phosphatidylinositol 4-kinase type-ii [Source:VB Community Annotation] |
| AAEL003739 | | 0.0055 | 3.4519 | 0.9872 | 0.9996 | M-type 9 protein, putative [Source:VB Community Annotation] |
| AAEL006071 | | 0.0047 | 6.3879 | 0.9872 | 0.9996 | |
| AAEL019567 | NA | -0.0148 | 5.8517 | 0.9874 | 0.9996 | NA |
| AAEL014863 | | 0.0050 | 6.3518 | 0.9875 | 0.9996 | glycogenin [Source:VB Community Annotation] |
| AAEL012056 | ORP1 | -0.0139 | 5.0630 | 0.9876 | 0.9996 | oxysterol-binding protein related protein (ORP1) [Source:VB Community Annotation] |
| AAEL021578 | NA | -0.0047 | 5.8883 | 0.9876 | 0.9996 | NA |
| AAEL018266 | | 0.0089 | 2.4245 | 0.9877 | 0.9996 | |
| AAEL003899 | | 0.0093 | 2.4108 | 0.9878 | 0.9996 | sugar transporter [Source:VB Community Annotation] |
| AAEL006370 | | -0.0051 | 3.4945 | 0.9880 | 0.9996 | amsh [Source:VB Community Annotation] |
| AAEL011381 | | -0.0055 | 9.8442 | 0.9880 | 0.9996 | NADH-ubiquinone oxidoreductase fe-s protein 2 (ndufs2) [Source:VB Community Annotation] |
| AAEL013950 | | 0.0058 | 6.4074 | 0.9882 | 0.9996 | DEAD box ATP-dependent RNA helicase [Source:VB Community Annotation] |
| AAEL020644 | NA | -0.0126 | 0.1962 | 0.9883 | 0.9996 | NA |
| AAEL000460 | | -0.0057 | 3.9948 | 0.9883 | 0.9996 | |
| AAEL001631 | | -0.0067 | 2.0159 | 0.9886 | 0.9996 | |
| AAEL008910 | | -0.0092 | 1.3774 | 0.9887 | 0.9996 | |
| AAEL008576 | | 0.0082 | 1.7275 | 0.9888 | 0.9996 | tRNA-splicing endonuclease subunit Sen34 [Source:UniProtKB/TrEMBL;Acc:Q16YD2] |
| AAEL006982 | | -0.0041 | 3.4495 | 0.9888 | 0.9996 | lipase [Source:VB Community Annotation] |
| AAEL004667 | | -0.0092 | 4.1412 | 0.9888 | 0.9996 | |
| AAEL008707 | | 0.0039 | 4.9796 | 0.9889 | 0.9996 | |

| | | | | | | |
|---|---|---|---|---|---|---|
| AAEL003678 | | 0.0125 | 4.3650 | 0.9890 | 0.9996 | diphosphoinositol polyphosphate phosphohydrolase, putative [Source:VB Community Annotation] |
| AAEL021365 | NA | -0.0221 | 4.2717 | 0.9892 | 0.9996 | NA |
| AAEL009908 | | 0.0054 | 4.2299 | 0.9893 | 0.9996 | glutamyl-tRNA(Gln) amidotransferase, subunit-B, putative [Source:VB Community Annotation] |
| AAEL020637 | NA | 0.0117 | 0.3251 | 0.9893 | 0.9996 | NA |
| AAEL021860 | NA | 0.0065 | 2.8119 | 0.9894 | 0.9996 | NA |
| AAEL000575 | | -0.0078 | 5.0324 | 0.9894 | 0.9996 | apyrase, putative [Source:VB Community Annotation] |
| AAEL010738 | | 0.0092 | 3.7720 | 0.9895 | 0.9996 | sodium bicarbonate cotransporter [Source:VB Community Annotation] |
| AAEL023258 | NA | 0.0066 | 3.7956 | 0.9898 | 0.9996 | NA |
| AAEL019627 | NA | 0.0053 | 6.1457 | 0.9898 | 0.9996 | NA |
| AAEL008389 | | -0.0040 | 6.1528 | 0.9899 | 0.9996 | ankyrin repeat-rich membrane-spanning protein [Source:VB Community Annotation] |
| AAEL000717 | | -0.0210 | -0.6171 | 0.9899 | 0.9996 | protocadherin [Source:VB Community Annotation] |
| AAEL006917 | | -0.0037 | 4.3681 | 0.9899 | 0.9996 | MG-160, putative [Source:VB Community Annotation] |
| AAEL002334 | eIF3-S6 | 0.0036 | 7.2254 | 0.9900 | 0.9996 | eukaryotic translation initiation factor 3 subunit E (eIF3e) [Source:VB Community Annotation] |
| AAEL013766 | | -0.0171 | 3.5846 | 0.9902 | 0.9996 | |
| AAEL026170 | NA | -0.0074 | 2.9468 | 0.9902 | 0.9996 | NA |
| AAEL007180 | | -0.0084 | 1.2186 | 0.9902 | 0.9996 | |
| AAEL020261 | NA | 0.0053 | 4.0358 | 0.9902 | 0.9996 | NA |
| AAEL014672 | | 0.0107 | 0.3949 | 0.9902 | 0.9996 | |
| AAEL000697 | | 0.0051 | 2.3734 | 0.9903 | 0.9996 | |
| AAEL024334 | NA | 0.0099 | 1.5370 | 0.9903 | 0.9996 | NA |
| AAEL008005 | | -0.0143 | -1.3326 | 0.9905 | 0.9996 | |
| AAEL001928 | Act1 | -0.0084 | 6.2908 | 0.9906 | 0.9996 | Actin-1 [Source:UniProtKB/Swiss-Prot;Acc:P49128] |
| AAEL019668 | NA | -0.0098 | 2.8360 | 0.9909 | 0.9996 | NA |
| AAEL021515 | NA | 0.0074 | 0.2310 | 0.9909 | 0.9996 | NA |
| AAEL003837 | | -0.0056 | 5.0798 | 0.9909 | 0.9996 | ryanodine receptor 3, brain [Source:VB Community Annotation] |
| AAEL020359 | NA | 0.0040 | 2.9595 | 0.9910 | 0.9996 | NA |
| AAEL023019 | NA | 0.0207 | 2.3308 | 0.9911 | 0.9996 | NA |
| AAEL005097 | | 0.0039 | 11.1564 | 0.9911 | 0.9996 | cold induced protein (BnC24A), putative [Source:VB Community Annotation] |
| AAEL002870 | | -0.0029 | 5.1224 | 0.9912 | 0.9996 | dipeptidyl peptidase iii [Source:VB Community Annotation] |
| AAEL018129 | | -0.0117 | 0.4930 | 0.9912 | 0.9996 | |
| AAEL021212 | NA | 0.0109 | 0.6421 | 0.9913 | 0.9996 | NA |
| AAEL005593 | | -0.0029 | 8.0561 | 0.9914 | 0.9996 | |
| AAEL024952 | NA | -0.0140 | -0.7508 | 0.9915 | 0.9996 | NA |
| AAEL014520 | | 0.0050 | 2.8584 | 0.9915 | 0.9996 | follistatin [Source:VB Community Annotation] |
| AAEL002898 | | 0.0052 | 7.3882 | 0.9916 | 0.9996 | |
| AAEL001840 | | -0.0092 | 2.7901 | 0.9917 | 0.9996 | zinc carboxypeptidase [Source:VB Community Annotation] |
| AAEL018334 | | -0.0087 | 2.4583 | 0.9918 | 0.9996 | |
| AAEL014765 | | 0.0034 | 5.9673 | 0.9918 | 0.9996 | |
| AAEL015294 | | -0.0168 | 7.9201 | 0.9918 | 0.9996 | serine-type enodpeptidase, [Source:VB Community Annotation] |
| AAEL004032 | | -0.0032 | 4.2683 | 0.9919 | 0.9996 | acetylcholine receptor protein alpha 1, 2, 3, 4 invertebrate [Source:VB Community Annotation] |

| | | | | | | |
|---|---|---|---|---|---|---|
| AAEL009911 | | 0.0035 | 6.8725 | 0.9919 | 0.9996 | rotamase [Source:VB Community Annotation] |
| AAEL011459 | | 0.0064 | 4.0068 | 0.9920 | 0.9996 | |
| AAEL004696 | | -0.0081 | 0.3674 | 0.9921 | 0.9996 | |
| AAEL009786 | | 0.0055 | 1.0660 | 0.9922 | 0.9996 | |
| AAEL012340 | | 0.0124 | -2.2266 | 0.9923 | 0.9996 | lipase 1 precursor [Source:VB Community Annotation] |
| AAEL022695 | NA | 0.0179 | 0.9370 | 0.9923 | 0.9996 | NA |
| AAEL021221 | NA | -0.0110 | 0.4413 | 0.9925 | 0.9996 | NA |
| AAEL010819 | | -0.0033 | 8.6298 | 0.9926 | 0.9996 | vacuolar ATP synthase subunit H [Source:VB Community Annotation] |
| AAEL009684 | | -0.0083 | 6.5690 | 0.9926 | 0.9996 | |
| AAEL006344 | | 0.0072 | 4.1018 | 0.9926 | 0.9996 | sulfotransferase (sult) [Source:VB Community Annotation] |
| AAEL018149 | | 0.0050 | 4.4599 | 0.9928 | 0.9996 | |
| AAEL011502 | | 0.0024 | 5.9690 | 0.9929 | 0.9996 | |
| AAEL007730 | | 0.0043 | 1.9550 | 0.9930 | 0.9996 | tetratricopeptide repeat protein 10, tpr10 [Source:VB Community Annotation] |
| AAEL010330 | | 0.0027 | 8.8249 | 0.9932 | 0.9996 | Succinate dehydrogenase [ubiquinone] iron-sulfur subunit, mitochondrial [Source:UniProtKB/TrEMBL;Acc:Q16TA7] |
| AAEL021595 | NA | 0.0024 | 4.3499 | 0.9932 | 0.9996 | NA |
| AAEL009914 | | -0.0028 | 4.4227 | 0.9933 | 0.9996 | small nuclear ribonucleoprotein sm d2 [Source:VB Community Annotation] |
| AAEL004728 | | 0.0055 | 2.4699 | 0.9934 | 0.9996 | 5-methyltetrahydrofolate:homocysteine methyltransferase [Source:VB Community Annotation] |
| AAEL001498 | | -0.0027 | 4.7483 | 0.9935 | 0.9996 | |
| AAEL017043 | Or84 | -0.0056 | 1.6141 | 0.9935 | 0.9996 | Odorant receptor [Source:UniProtKB/TrEMBL;Acc:J9HYG1] |
| AAEL005706 | | -0.0032 | 4.6679 | 0.9937 | 0.9996 | triacylglycerol lipase [Source:VB Community Annotation] |
| AAEL011789 | | -0.0035 | 7.3349 | 0.9937 | 0.9996 | Probable citrate synthase 2, mitochondrial Precursor (EC 2.3.3.1) [Source:VB Community Annotation] |
| AAEL000729 | | 0.0038 | 9.0492 | 0.9938 | 0.9996 | |
| AAEL000956 | | 0.0028 | 3.7150 | 0.9938 | 0.9996 | |
| AAEL010333 | | 0.0052 | 1.9238 | 0.9938 | 0.9996 | Probable ribosome biogenesis protein AAEL010333 [Source:UniProtKB/TrEMBL;Acc:Q16T92] |
| AAEL000556 | CTL25 | 0.0138 | 7.8731 | 0.9939 | 0.9996 | C-Type Lectin (CTL25) [Source:VB Community Annotation] |
| AAEL011031 | | 0.0081 | -0.2547 | 0.9939 | 0.9996 | |
| AAEL005451 | RpL38 | -0.0022 | 12.3393 | 0.9939 | 0.9996 | 60S ribosomal protein L38 [Source:UniProtKB/Swiss-Prot;Acc:Q1HRT4] |
| AAEL000464 | | -0.0026 | 3.5338 | 0.9940 | 0.9996 | |
| AAEL006719 | AMY1 | -0.0127 | 8.9420 | 0.9940 | 0.9996 | Alpha-amylase I Precursor (EC 3.2.1.1)(1,4-alpha-D-glucan glucanohydrolase) [Source:VB Community Annotation] |
| AAEL025955 | NA | 0.0068 | 0.7465 | 0.9942 | 0.9996 | NA |
| AAEL025975 | NA | 0.0119 | 6.0529 | 0.9943 | 0.9996 | NA |
| AAEL019843 | NA | -0.0046 | 4.8953 | 0.9944 | 0.9996 | NA |
| AAEL006785 | RpL18a | -0.0035 | 2.2857 | 0.9944 | 0.9996 | 60S ribosomal protein L18a [Source:UniProtKB/TrEMBL;Acc:Q174U3] |
| AAEL014079 | | -0.0020 | 4.3989 | 0.9944 | 0.9996 | serine protease inhibitor, serpin [Source:VB Community Annotation] |
| AAEL028056 | NA | -0.0091 | -0.3231 | 0.9947 | 0.9996 | NA |
| AAEL014050 | | -0.0031 | 3.8233 | 0.9948 | 0.9996 | testosterone-regulated protein rp2 [Source:VB Community Annotation] |
| AAEL005527 | | 0.0027 | 4.5324 | 0.9949 | 0.9996 | Probable exonuclease mut-7 homolog (EC 3.1.-.-)(Exonuclease 3'-5' domain-containing protein 3 homolog) [Source:VB Community Annot |
| AAEL001188 | | 0.0022 | 3.8104 | 0.9951 | 0.9996 | |
| AAEL006700 | | 0.0098 | 2.1455 | 0.9951 | 0.9996 | trypsin, putative [Source:VB Community Annotation] |
| AAEL013782 | | 0.0021 | 5.6419 | 0.9951 | 0.9996 | |

| | | | | | | |
|---|---|---|---|---|---|---|
| AAEL025171 | NA | -0.0017 | 4.2131 | 0.9953 | 0.9996 | NA |
| AAEL004319 | | 0.0020 | 4.4397 | 0.9954 | 0.9996 | epidermal growth factor receptor [Source:VB Community Annotation] |
| AAEL018042 | | 0.0033 | 2.9806 | 0.9958 | 0.9996 | |
| AAEL017678 | U4 | 0.0045 | -0.4582 | 0.9959 | 0.9996 | U4 spliceosomal RNA [Source:RFAM;Acc:RF00015] |
| AAEL012750 | | 0.0023 | 5.1029 | 0.9959 | 0.9996 | transcription factor TFIIH-subunit, putative [Source:VB Community Annotation] |
| AAEL004916 | | 0.0022 | 4.3755 | 0.9959 | 0.9996 | |
| AAEL022310 | NA | -0.0113 | 0.4572 | 0.9959 | 0.9996 | NA |
| AAEL002241 | | -0.0078 | 0.9635 | 0.9960 | 0.9996 | cuticle protein, putative [Source:VB Community Annotation] |
| AAEL012940 | | 0.0071 | 0.4342 | 0.9961 | 0.9996 | |
| AAEL014412 | CYP304B2 | 0.0068 | -0.1898 | 0.9961 | 0.9996 | cytochrome P450 [Source:VB Community Annotation] |
| AAEL003437 | PrBP | 0.0012 | 4.7977 | 0.9962 | 0.9996 | cGMP 3',5'-cyclic phosphodiesterase subunit delta [Source:VB Community Annotation] |
| AAEL025165 | NA | 0.0025 | 1.3669 | 0.9962 | 0.9996 | NA |
| AAEL011774 | | 0.0022 | 1.4474 | 0.9962 | 0.9996 | sarcolemmal associated protein, putative [Source:VB Community Annotation] |
| AAEL026747 | NA | 0.0065 | 0.1779 | 0.9963 | 0.9996 | NA |
| AAEL025004 | NA | 0.0032 | 0.3441 | 0.9964 | 0.9996 | NA |
| AAEL001172 | | 0.0020 | 3.5414 | 0.9964 | 0.9996 | |
| AAEL008410 | | 0.0027 | 1.6694 | 0.9964 | 0.9996 | helix-loop-helix transcription factor [Source:VB Community Annotation] |
| AAEL019733 | NA | 0.0097 | 1.7639 | 0.9964 | 0.9996 | NA |
| AAEL020217 | NA | -0.0017 | 3.1438 | 0.9965 | 0.9996 | NA |
| AAEL002877 | | 0.0018 | 3.4160 | 0.9966 | 0.9996 | kinesin-like protein KIF17 [Source:VB Community Annotation] |
| AAEL002407 | | 0.0010 | 6.4877 | 0.9967 | 0.9996 | DNA repair protein xp-e [Source:VB Community Annotation] |
| AAEL009089 | APG12 | 0.0013 | 4.1157 | 0.9968 | 0.9996 | autophagy related gene [Source:VB Community Annotation] |
| AAEL008538 | | 0.0014 | 2.5728 | 0.9968 | 0.9996 | |
| AAEL014614 | | 0.0017 | 3.8936 | 0.9968 | 0.9996 | cytochrome P450 [Source:VB Community Annotation] |
| AAEL003555 | | 0.0018 | 3.7104 | 0.9971 | 0.9996 | ribosome biogenesis protein [Source:VB Community Annotation] |
| AAEL010787 | | -0.0011 | 9.8549 | 0.9972 | 0.9996 | DEAD box ATP-dependent RNA helicase [Source:VB Community Annotation] |
| AAEL010815 | | 0.0011 | 4.5964 | 0.9972 | 0.9996 | |
| AAEL005910 | | 0.0015 | 3.5199 | 0.9973 | 0.9996 | programmed cell death protein [Source:VB Community Annotation] |
| AAEL020372 | NA | -0.0055 | 0.4059 | 0.9974 | 0.9996 | NA |
| AAEL019981 | NA | -0.0017 | 5.2956 | 0.9974 | 0.9996 | NA |
| AAEL019985 | NA | -0.0044 | 1.1908 | 0.9975 | 0.9996 | NA |
| AAEL002692 | | -0.0017 | 5.6254 | 0.9975 | 0.9996 | beat protein [Source:VB Community Annotation] |
| AAEL027959 | NA | 0.0026 | 1.4966 | 0.9976 | 0.9996 | NA |
| AAEL006381 | | -0.0046 | -0.0772 | 0.9976 | 0.9996 | Sphingomyelin phosphodiesterase [Source:UniProtKB/TrEMBL;Acc:Q176G6] |
| AAEL006483 | | 0.0050 | 1.3203 | 0.9976 | 0.9996 | |
| AAEL009421 | | 0.0011 | 7.4219 | 0.9978 | 0.9996 | cyclophilin-r [Source:VB Community Annotation] |
| AAEL003431 | | 0.0009 | 7.3762 | 0.9978 | 0.9996 | proteasome subunit beta type 7,10 [Source:VB Community Annotation] |
| AAEL013763 | | -0.0015 | 2.0472 | 0.9979 | 0.9996 | arrowhead [Source:VB Community Annotation] |
| AAEL007355 | mRpS18A | 0.0012 | 5.8102 | 0.9979 | 0.9996 | mitochondrial ribosomal protein, S18A, putative [Source:VB Community Annotation] |
| AAEL009364 | | -0.0011 | 3.8938 | 0.9979 | 0.9996 | cdc2l5 [Source:VB Community Annotation] |

| | | | | | | |
|---|---|---|---|---|---|---|
| AAEL008306 | | 0.0006 | 4.6106 | 0.9980 | 0.9996 | mitogen activated protein kinase kinase kinase 5, mapkkk5, mekk5 [Source:VB Community Annotation] |
| AAEL017075 | | 0.0010 | 9.1769 | 0.9981 | 0.9996 | |
| AAEL017051 | | -0.0033 | -1.3989 | 0.9981 | 0.9996 | |
| AAEL006154 | | 0.0023 | -0.2689 | 0.9981 | 0.9996 | |
| AAEL002666 | | -0.0018 | 0.4377 | 0.9981 | 0.9996 | AMP dependent ligase [Source:VB Community Annotation] |
| AAEL009311 | | -0.0011 | 3.2196 | 0.9983 | 0.9996 | 3-hydroxy-3-methylglutaryl-coenzyme A reductase [Source:VB Community Annotation] |
| AAEL013987 | | 0.0010 | 7.0267 | 0.9983 | 0.9996 | |
| AAEL009061 | | 0.0010 | 4.9529 | 0.9984 | 0.9996 | rrm/rnp domain [Source:VB Community Annotation] |
| AAEL007349 | | 0.0009 | 3.1480 | 0.9986 | 0.9997 | |
| AAEL020587 | NA | 0.0028 | -0.2118 | 0.9988 | 0.9998 | NA |
| AAEL013503 | | -0.0005 | 2.8764 | 0.9990 | 0.9999 | |
| AAEL028141 | NA | 0.0004 | 7.1850 | 0.9992 | 0.9999 | NA |
| AAEL010366 | | 0.0007 | 6.3233 | 0.9993 | 0.9999 | glucosyl/glucuronosyl transferases [Source:VB Community Annotation] |
| AAEL026243 | NA | -0.0003 | 4.3564 | 0.9993 | 0.9999 | NA |
| AAEL005765 | | -0.0004 | 1.6841 | 0.9994 | 0.9999 | |
| AAEL005515 | | 0.0002 | 8.0121 | 0.9995 | 0.9999 | heterogeneous nuclear ribonucleoprotein [Source:VB Community Annotation] |
| AAEL004732 | GPRNPR2 | -0.0007 | -1.7420 | 0.9996 | 0.9999 | GPCR Growth Hormone Secretagogue/Neurotensin/Neuromedin/Thyrotropin Releasing Hormone Family [Source:VB Community Annota |
| AAEL012106 | | -0.0012 | 2.0591 | 0.9996 | 0.9999 | acetylcholine receptor protein alpha 1, 2, 3, 4 invertebrate [Source:VB Community Annotation] |
| AAEL002225 | | 0.0001 | 5.8679 | 0.9998 | 0.9999 | 5-oxoprolyl-peptidase, putative [Source:VB Community Annotation] |
| AAEL020264 | NA | -0.0003 | 1.3897 | 0.9999 | 0.9999 | NA |
| AAEL021082 | NA | 0.0001 | 4.3756 | 0.9999 | 0.9999 | NA |
